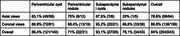# Poster Session 2: Wednesday, February 11, 2026 3:30 PM – 5:00 PM

**DOI:** 10.1002/pmf2.70175

**Published:** 2026-02-09

**3:30 PM – 5:00 PM**

## 398 | Iron Deficiency Without Anemia is Highly Prevalent at 24–28 Weeks’ Gestation: A Prospective Cohort

Abbey P. Donahue^1^; Angelina H. Wiater^2^; Mackenzie Wood^1^; Lexi Frankel^1^; Matthew Zuber^3^; Melissa Kozakiewicz^4^; Chad Grotegut^1^

^1^Wake Forest University, Winston‐Salem, NC; ^2^Wake Forest University School of Medicine, Winston‐Salem, NC; ^3^Wake Forest University School of Medicine, Winston Salem, NC; ^4^Wake Forest School of Medicine, Winston‐Salem, NC

**Objective**: To determine the prevalence of iron deficiency without anemia (IDWA) at 24–28 weeks’ gestation among non‐anemic pregnant patients.

**Study Design**: We conducted a prospective cohort study of pregnant patients with hemoglobin ≥ 11 g/dL at their initial prenatal visit who underwent ferritin testing between 24w0d–28w6d across three outpatient clinics. Patients with prior anemia, known iron deficiency, malabsorptive disorders, or multifetal gestation were excluded. Ferritin results were blinded to providers and not used to guide clinical care. IDWA was defined as ferritin <30 µg/L in the setting of normal hemoglobin (Hgb ≥ 11 g/dL). Baseline characteristics were compared between patients with and without IDWA.

**Results**: Among 321 patients with ferritin levels drawn during the study period, 66 (20%) were anemic (Hgb <11 g/dL) and excluded. Of the remaining 251 non‐anemic patients, 94% (*N* = 240) met criteria for IDWA (Figure 1). Baseline characteristics were largely similar between ferritin groups. Patients with ferritin ≥ 30 µg/L trended toward higher rates of nulliparity (53% vs 34%, *p* = 0.13) and tobacco use during pregnancy (13% vs 5%, *p* = 0.2), though these differences were not statistically significant. Maternal age, hemoglobin and MCV values, and rates of hypertensive disorders were comparable between groups (Table 1).

**Conclusion**: Iron deficiency in pregnancy is exceedingly common, even among those with normal hemoglobin levels based on current ferritin criteria. These findings suggest that routine screening or empiric iron supplementation may be warranted to prevent the later development of anemia. An updated ferritin cutoff to define IDWA in pregnancy may be warranted given the distribution we observed. Once the cohort has completed delivery, we plan to evaluate associations between midtrimester ferritin levels and delivery outcomes; specifically whether midtrimester ferritin levels can predict hemoglobin at birth admission and other perinatal outcomes.

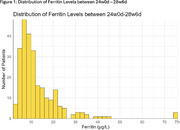

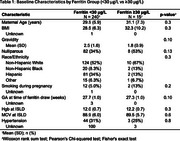

**3:30 PM – 5:00 PM**

## 399 | **Limited** Diagnostic **Value of Maternal Serum AFP Screening for Gastroschisis Detection**

Abigail E. Rex^1^; Christian M. Parobek^2^; Morgan Motakef^2^; Brian A. Burnett^2^; KC Sutherland^2^; Rebecca M. Johnson^2^; Ahmed A. Nassr^3^

^1^Baylor College of Medicine, Houst, TX; ^2^Baylor College of Medicine, Houston, TX; ^3^Baylor College of Medicine, Baylor College of Medicine, TX

**Objective**: With advances in prenatal ultrasound and the shift from serum aneuploidy screening to cell‐free DNA testing, the role of maternal serum AFP (MSAFP) in detecting fetal body‐wall defects is unclear. We evaluated the diagnostic utility of MSAFP screening for prenatal gastroschisis (GS) in a modern cohort.

**Study Design**: We conducted an IRB‐approved retrospective cohort study of prenatally diagnosed GS cases at a single fetal center, from 3/2012 to 3/2024. Maternal, fetal, and neonatal data were analyzed using bivariate statistics and linear regression to assess associations between MSAFP screening, diagnostic timing, and neonatal outcomes.

**Results**: Among 149 prenatally‐detected GS cases, 69 (46%) had MSAFP screening. MSAFP screening was not associated with earlier gestational age (GA) at GS suspicion or diagnosis (*p* = 0.3 and *p* = 0.4, respectively; Table 1). In contrast, early anatomy or NT ultrasound (US) led to significantly earlier diagnosis compared to MSAFP (median 14.5 vs. 19.1 weeks; *p* < 0.001). Among patients with timely prenatal care (*n* = 136) but no early anatomy US, MSAFP screening was linked to a minor improvement in diagnostic timing (19.6 vs. 20.0 weeks; *p* = 0.05). Regardless of MSAFP screening, those with early anatomy US had the earliest diagnoses of GS (Figure 1). Furthermore, MSAFP MoM values were not associated with neonatal morbidity composite (*p* = 0.4).

**Conclusion**: In the comparison of all GS cases, MSAFP screening was not associated with earlier diagnosis. In cases with timely prenatal care and no early anatomy ultrasound, MSAFP screening was associated with slightly earlier detection (∼3 days). The level of MSAFP elevation was not predictive of adverse neonatal outcomes. These findings support prioritizing early ultrasound for prenatal diagnosis of gastroschisis and suggest that the diagnostic value of MSAFP is limited in current prenatal care.

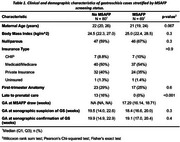

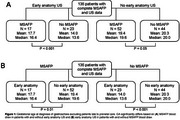

**3:30 PM – 5:00 PM**

## 400 | **Characteristics of Psychotropic Medication Prescribing Before, During, and After Pregnancy**

Adina R. Kern‐Goldberger^1^; Hannah E. Bowers^2^; Stacey Ehrenberg^3^; Cara D. Dolin^4^; Sarah Nazeer^1^

^1^Cleveland Clinic Lerner College of Medicine, Cleveland Clinic Lerner College of Medicine, OH; ^2^Cleveland Clinic Foundation, Cleveland Clinic Foundation, Cleveland, OH; ^3^Cleveland Clinic Foundation, Cleveland Clinic Foundation, OH; ^4^Cleveland Clinic, Clevelend, OH

**Objective**: Mental health conditions like depression and anxiety are common in reproductive age individuals, yet antidepressants and anxiolytics are often discontinued in pregnancy. This study evaluates trends in these prescriptions before and throughout pregnancy.

**Study Design**: This is a retrospective cohort study of individuals who delivered in a single health system from 1/1/2021–6/30/2025. Prescriptions were evaluated at 4 time points: 6 months prior to delivery, the initial prenatal visit, 32 weeks gestation, and discharge from delivery admission. The frequency of antidepressant and anxiolytic prescriptions at each time point was compared between individuals who were prescribed these medications prior to pregnancy and those who initiated them during pregnancy. Characteristics of individuals who continuously prescribed psychotropic medications were compared to those who initiated medication during pregnancy at any time point in bivariate analyses.

**Results**: Among 52,778 individuals, 6421 (12.2%) had an antidepressant or anxiolytic prescription in the 6 months before pregnancy. Of these, 1499 (23.3%) continued their prescriptions throughout pregnancy and at discharge. There were 5048 (9.6%) individuals that were initiated on medication during or immediately postpartum [Figure]. Those with continuous prescriptions were older, had higher pre gravid and delivery BMI, and were less like likely to be Black or Hispanic. They were also less likely to have Medicaid insurance, more likely to be multiparous and initiate prenatal care earlier [Table]. Preterm birth rates did not differ between groups; however, individuals with continuous prescription use had a higher rate of NICU admission (12.2% vs. 8.4%, *p* < 0.01).

**Conclusion**: Affective disorders are a major comorbidity in pregnancy, with a large number of individuals taking psychotropic medication during this time. Understanding prescribing patterns and identifying disparities related to social determinants of health can inform strategies to optimize perinatal mental health care.

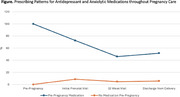

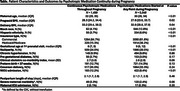

**3:30 PM – 5:00 PM**

## 401 | **Laser Ablation of Type II vs Type III Vasa Previa**

Adriana S. Mockler^1^; Randy Mohtar^1^; Arlyn S. Llanes^2^; Lisa M. Korst^3^; Alireza A. Shamshirsaz^4^; Ramen H. Chmait^2^

^1^Keck School of Medicine of USC, University of Southern California, Los Angeles, CA; ^2^Keck School of Medicine of USC, University of Southern California, Keck School of Medicine of USC, University Of Southern California, CA; ^3^Childbirth Research Associates, North Hollywood, CA; ^4^Boston Children's Hospital, Harvard Medical School, Boston, MA

**Objective**: Fetoscopic laser ablation is a novel therapeutic treatment for Types II and III vasa previa (VP), a condition where fetal blood vessels traverse near the internal cervical os, unsupported by underlying placenta. VP connecting the main placenta to an accessory lobe is classified as Type II, and a VP that exits and reenters the same placenta is classified as Type III. We compared patient characteristics and outcomes after laser ablation of Type II vs Type III VP.

**Study Design**: This was a retrospective cohort study (2013–2025) comparing pregnancy, surgical, and neonatal outcomes of singleton pregnancies with Types II and III VP after laser ablation treatment at ≥ 31 gestational weeks (range 31.1–33.7 weeks). Bivariate analyses were used to compare outcomes.

**Results**: Of 32 patients who underwent VP laser ablation during the study period, 14 (43.8%) had Type II and 18 (56.2%) had Type III VP. Preoperative characteristics (Table 1) and operative and perinatal outcomes (Table 2) were similar for VP Types. For Type II vs III, the mean (SD) gestational age (GA) at procedure was 32.0 ± 0.7 vs 32.0 ± 0.4 weeks, *p* = .731 and mean vessel distance to the os was 0.73 ± 0.65 vs 0.84 ± 0.62 cm, *p* = .893. The mean laser energy to achieve occlusion for Type II vs III was 7498 ± 5865 vs 4370 ± 2805 joules, *p* = .262. All patients had successful VP occlusion (1 required a second attempt) and all were managed as outpatients. The mean (SD) delivery GA was similar (36.7 ± 1.6 vs 37.0 ± 2.2 weeks, *p* = .608), and the vaginal delivery rates did not differ (71.4% vs 55.6%, *p* = .471). All patients had a live birth and survived at least 30 days. There were 2 cases requiring hyperbilirubinemia treatment, 1 for each Type, and 1 Type III case of respiratory distress syndrome (RDS). The neonatal intensive care unit length of stay (LOS) was 6.7 ± 3.1 (*n* = 3) vs 26.8 ± 18.9 (*n* = 4) days, *p* = .034; one outlier in the Type III group had a LOS of 53 days due to RDS. There were no cases of intraventricular hemorrhage, neonatal blood transfusion, or sepsis.

**Conclusion**: Laser ablation treatment outcomes for Type II vs Type III VP were similar.

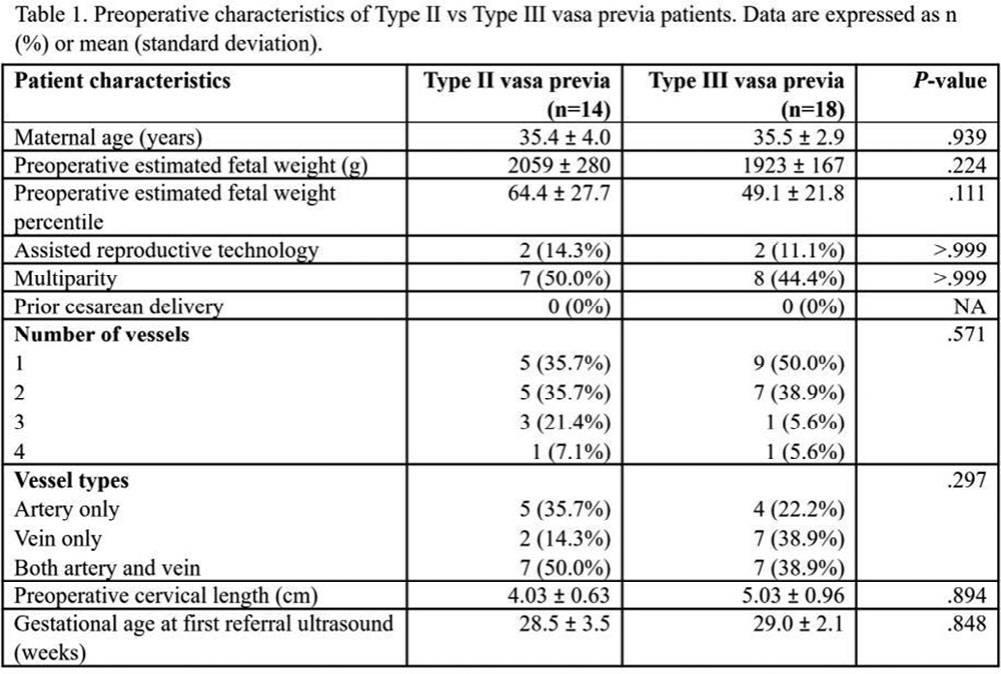

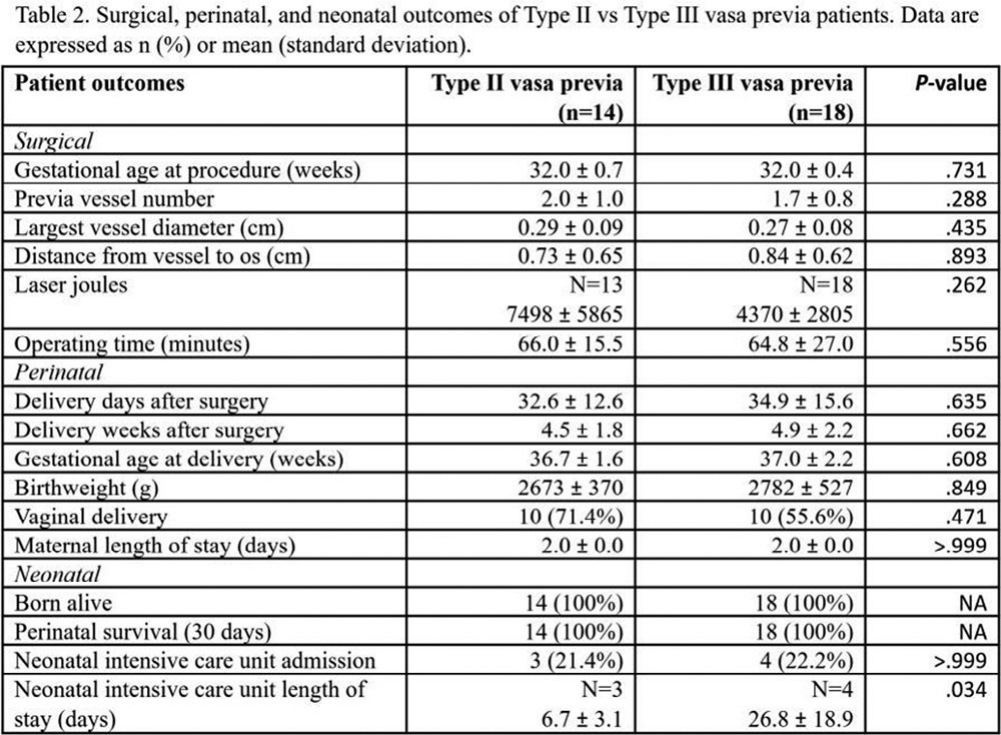

**3:30 PM – 5:00 PM**

## 403 | **Comparing CGM** Fasting **Blood Glucose Versus Time in Range as Predictors of Neonatal Hypoglycemia**

Alesandra Rau^1^; Genevieve Mazza^1^; Megan C. Oakes^2^

^1^UC Irvine Medical Center, Orange, CA; ^2^MemorialCare Miller Children's and Women's Hospital, Long Beach, CA

**Objective**: Neonatal hypoglycemia is a common manifestation of sub‐optimally controlled gestational diabetes requiring medication (A2GDM) and type 2 diabetes mellitus (T2DM). Traditional guidance for strict glycemic control in pregnancy, with a fasting blood glucose (FBG) goal <95 mg/dL, reduces this complication. A continuous glucose meter (CGM) time in range (TIR) >70% is also associated with improved neonatal outcomes in patients with T1DM. As continuous glucose meters (CGM) are utilized more frequently in patients with A2GDM and T2DM, it is unclear which parameter is the strongest predictor of adverse outcomes. We aimed to compare predictive characteristics of mean FBG >95 mg/dL to TIR <70% for neonatal hypoglycemia in patients using CGM.

**Study Design**: This was a retrospective cohort study of patients with T2DM or A2GDM using a CGM in the 3rd trimester between January 2021 and May 2025. Mean 3^rd^ trimester BG at 6AM was used as a proxy for FBG. The cohort was grouped by FBG <95 or >95 mg/dL and TIR >70% or <70%. Area under the receiver operator curves (AUROC) were generated to compare the predictive abilities of FBG >95 versus TIR <70% as predictors of neonatal hypoglycemia (defined as neonatal BG <40 mg/dL within the first 24 h of life). Predictive characteristics were compared between the groups.

**Results**: Of 133 patients in the cohort, 108 (82.4%) had mean FBG >95 mg/dL and 47 (35.6%) had TIR <70%. The AUROC for FBG >95 mg/dL was not significantly different compared to TIR <70% for the prediction of neonatal hypoglycemia (0.56 (95% CI 0.54–0.72) and 0.63 (95% CI 0.50–0.63), respectively; *p* = 0.15) (Figure 1). Compared to TIR <70%, FBG >95 had higher positive predictive value and accuracy; however, the positive likelihood ratio was weak (Table 1).

**Conclusion**: Among those with A2GDM and T2DM using a CGM, 3^rd^ trimester mean FBG >95 mg/dL and TIR <70% are poor predictors of neonatal hypoglycemia. As utilization of CGMs increases for this patient population, larger studies are needed to identify which parameters most strongly reduce adverse neonatal outcomes.

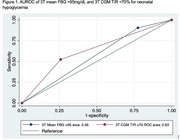

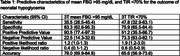

**3:30 PM – 5:00 PM**

## 404 | **Long Term (3 Year) Follow‐Up for Patients With Placenta Accreta Spectrum**

Alesha M. White^1^; Hee Jae Hong^2^; Jessica E. Pruszynski^2^; Breean Dakin^3^; Catherine Y. Spong^2^; Christina L. Herrera^2^

^1^University of Texas Southwestern Medical Center, Grand Prairie, TX; ^2^University of Texas Southwestern Medical Center, Dallas, TX; ^3^Parkland Health, Dallas, TX

**Objective**: The impact of placenta accreta spectrum (PAS) on long‐term mental and physical health is unknown. The objective of this study was to describe long‐term follow‐up for patients with PAS, including the impact of hysterectomy.

**Study Design**: Retrospective cohort study including postpartum patients complicated by PAS at a single urban hospital between 2009 and 2025. Maternal demographics and health visits for up to three years after PAS delivery were studied and compared based on hysterectomy status.

**Results**: Of 325 PAS patients, 2 were lost to follow up; of the remaining 323, 255 (79%) had a hysterectomy. Within 3 years post‐delivery, 18% (*n* = 58) developed a mood disorder, 12%(*n* = 38) a substance use disorder, 11% (*n* = 35) urinary dysfunction, and 4% (*n* = 13) sexual dysfunction. These complications were not different for women with and without hysterectomy. Health visits were more common in the 1st year (median: 2 (1–5)) than years 2–3 (median: 0 (0–2)) and were significantly higher in women with hysterectomy. 157 (49%) were seen in the emergency department with 14% (*n* = 44) leading to an admission; this was higher in women with hysterectomy. The location of health visits was significantly different between those who did and did not have a hysterectomy (Table).

**Conclusion**: More than 90% of patients with PAS interacted with follow‐up care within our health care system, with the majority of visits immediately postpartum. Nearly one fifth of women developed a mood disorder and 10% developed a substance use disorder. Hysterectomy status did not affect these rates. Our findings support integrated post‐partum care to limit the burden of clinic visits and to better screen and evaluate these long‐term complications.

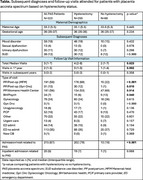

**3:30 PM – 5:00 PM**

## 405 | **Does Transfusion Differ in Placenta Accreta Spectrum Compared to Postpartum Hemorrhage Secondary to Other Causes**

Alesha M. White^1^; Jessica E. Pruszynski^2^; Anne M. Ambia^2^; Catherine Y. Spong^2^; Christina L. Herrera^2^

^1^University of Texas Southwestern Medical Center, Grand Prairie, TX; ^2^University of Texas Southwestern Medical Center, Dallas, TX

**Objective**: The objective of this study is to compare transfusion requirements between patients with placenta accreta spectrum (PAS) who undergo scheduled cesarean delivery (CD) with or without hysterectomy to patients who undergo scheduled CD and experienced PPH.

**Study Design**: This is a retrospective cohort study of patients with clinical diagnosis of PAS based on FIGO classification who delivered at a single institution with a multidisciplinary team between 2019 to 2025. Maternal demographics and delivery characteristics were compared between patients with PAS who underwent scheduled delivery to patients who underwent scheduled CD for other reasons but experienced PPH with an EBL of 1500cc or greater.

**Results**: Of 564 patients, 68 had PAS and 496 did not. Patients who underwent delivery for PAS delivered at earlier gestational ages (36 (36–36) vs 39 (38–39), *p* < 0.001) and were more likely to undergo hysterectomy (93% vs 4%, *p* < 0.001) with higher median EBLs (2000 (1500–2500cc) vs 1500 (1500–1750cc), *p* < 0.001). They were more likely to receive a transfusion (79% vs 30%, *p* < 0.001) and to receive packed red blood cells (pRBCs) (RR 2.76 (95% CI 2.23–3.33)) and fresh frozen plasma (FFP) (RR 5.97 (95% CI 4.13–8.48)). Rates of massive transfusion were also higher (26% vs 2%, *p* < 0.001) (Table 1). Of those transfused, PAS patients received a higher median number of total products (4.5 (1–55) vs 2 (1–34), *p* < 0.001) with higher median packed red blood cells and FFP transfused (both *p* < 0.001). Timing of transfusion also differed with immediate transfusion (0–2 hours from delivery) more common in the PAS cohort (*p* < 0.001).

**Conclusion**: Transfusion requirements are higher in patients with PAS than those who experience PPH after cesarean. The type of product required was significantly different with an increased use of FFP in cases of PAS. In preoperative planning for cases of PAS, blood products to include pRBCs and FFP should be readily available.

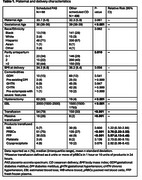

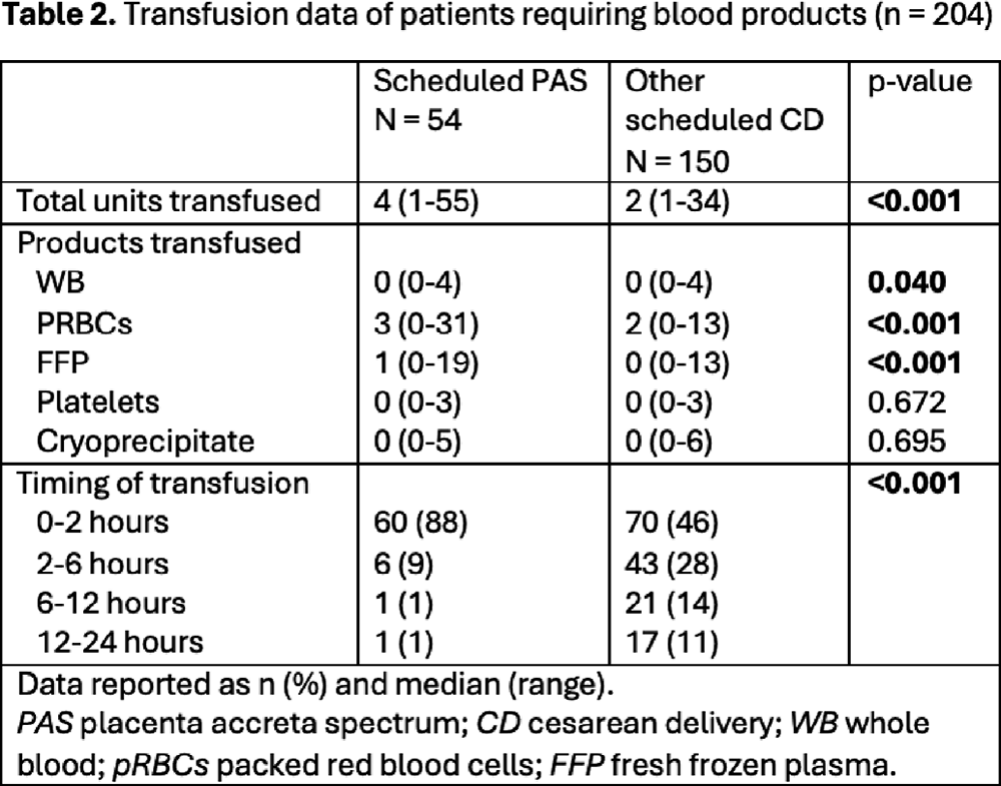

**3:30 PM – 5:00 PM**

## 406 | **Metabolomic** Profiles **in Patients With Postpartum Hemorrhage**

Alesha M. White^1^; Andrea Woods^2^; Ashley Solmonson^2^; David B. Nelson^2^

^1^University of Texas Southwestern Medical Center, Grand Prairie, TX; ^2^University of Texas Southwestern Medical Center, Dallas, TX

**Objective**: Serum metabolic profiles from patients who experience postpartum hemorrhage (PPH) are potential therapeutic targets but are largely unknown. The purpose of this study is to describe metabolomic profiles as they relate to PPH.

**Study Design**: This is a prospective observational study of patients who experienced PPH or who were at risk of experiencing PPH at the time of delivery. This data is a subgroup from a single‐center within a larger, multicenter industry sponsored study to validate a point of care viscoelastic assay device, the Quantra^®^ sonorheometry (SEER) system. Metabolites were extracted from maternal serum using 80% acetonitrile. Metabolomic analysis was performed using a Vanquish UHPLC coupled to a Thermo Scientific Exploris 240 hybrid quadrupole orbitrap high‐resolution mass spectrometer. Relative metabolite abundance was compared between patients experiencing PPH and controls. Metabolites that were increased or decreased by 20% were compared between patients with PPH and those without. Metabolite data were tested for significance using unpaired parametric t‐test or Mann‐Whitney test.

**Results**: Metabolomics data was collected from 41 samples and 33 (80%) experienced PPH. A supervised PLS‐DA analysis demonstrated that the metabolic profile for patients with PPH was distinct from patients without PPH (Figure 1). A total of 166 metabolites were identified and 29 were significantly different between PPH and controls. From the total list, 39 metabolites were increased by 20% (14 significant) and 56 were decreased by 20% (15 significant). Among all cases of PPH, the metabolites that were significantly and consistently decreased were amnio acids. This was driven by three non‐essential amino acids, theronine, L‐lysine and leucine (Figure 2).

**Conclusion**: Serum metabolomic profiles differ in patients with and without PPH. Patients who experience PPH specifically lack the metabolites related to amnio acid metabolism, specific to non‐essential amnio acids available via diet. This suggests a nutritional role in risk of PPH. Future large prospective studies are needed to confirm these findings.

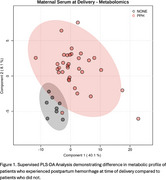

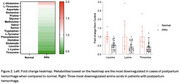

**3:30 PM – 5:00 PM**

## 407 | **Transvaginal** Ultrasound**‐Guided Treatment of Live Cesarean Scar Pregnancies**

Alexa L. Cohen^1^; Leeann Dar^2^; Fatima Estrada Trejo^3^; Ohad Rotenberg^2^; Pe'er Dar^2^; Georgios Doulaveris^2^

^1^University of Miami, Miami, FL; ^2^Montefiore Medical Center, Bronx, NY; ^3^Montefiore Medical Center, Montefiore Medical Center, NY

**Objective**: Despite a variety of interventions proposed for managing live cesarean scar pregnancies (CSP), there is a paucity of data on outcomes, particularly in the late first trimester. Our study aimed to assess the safety and efficacy of transvaginal ultrasound (TVU)‐guided feticide in the treatment of live CSP.

**Study Design**: A retrospective analysis of all live CSP cases treated with TVU‐guided feticide at a single academic institution from 2010–2023. Patients with prior intervention or who received systemic methotrexate (MTX) were excluded. TVU‐guided feticide, with administration of intracardiac potassium chloride and/or intrasac MTX was performed in‐office for all cases using a 20‐gauge needle under local anesthesia. Interval suction curettage (D&C) was offered to all patients after a negative bHCG. Primary outcome was successful treatment defined as complete pregnancy resolution not requiring further unplanned intervention. Outcomes were compared between early (5–8 weeks) and late (9–13 weeks) first trimester. Secondary outcomes included complications, rate of bHCG decrease a week following treatment, time to negative bHCG, time to sonographic resolution, and rate of interval D&C.

**Results**: 95 patients with CSP presented to our institution: 55 live CSP and 51 without prior intervention. Successful treatment was achieved in 47 patients (92.2%) with four requiring hysterectomy: two due to bleeding, and two due to infection. Overall, 35 patients (68.6%) were diagnosed in the early first trimester and 16 patients (31.4%) in the late first trimester. Demographics were comparable between groups. Complete resolution rates were 33/35 (94.3%) for early versus 14/16 (87.5%, *p* = 0.5) for late first trimester. Groups were similar for rate of complications, decrease of bHCG a week after treatment, time to negative bHCG, time to sonographic resolution, and rate of interval D&C.

**Conclusion**: TVU‐guided feticide emerges as a safe and efficacious first‐line treatment for live CSP throughout the first trimester.

**3:30 PM – 5:00 PM**

## 408 | **Outcomes of** Intrauterine **Transfusion in Hemolytic Disease of the Fetus and Newborn in the US**

Alexis Krumme^1^; Clair Blacketer^1^; Rupa Makadia^1^; Robert Suruki^1^; Lisa Schwartz^2^; May Lee Tjoa^3^

^1^Johnson & Johnson, Titusville, NJ; ^2^Johnson & Johnson, Raritan, NJ; ^3^Johnson & Johnson, Cambridge, MA

**Objective**: To evaluate complications of intrauterine transfusion (IUT) for the treatment of severe Hemolytic Disease of the Fetus and Newborn (HDFN) in the United States in the last twenty years.

**Study Design**: This study was conducted in pregnant individuals age 18–49 with commercial insurance in the Merative MarketScan Commercial Claims and Encounters (CCAE) database or government assistance insurance in the Merative MarketScan Multi‐State Medicaid (Medicaid) database from 2000–2024. Cohorts were defined by an ICD alloimmunization diagnosis code and IUT procedure code and a pregnancy outcome of live or still birth. IUT complications and pregnancy outcomes were compared between databases.

**Results**: In CCAE, 234 births (96.6% liveborn) occurred in 230 individuals. On average, each pregnancy underwent 2.5 (SD = 1.7) IUTs with the first occurring at gestational week 28 (SD = 5); 17% were delivered by Cesarean section within one day of an IUT. Maternal infections requiring hospitalization after the start of IUTs occurred in 3.0% of pregnancies. In Medicaid, 180 births (97.2% liveborn) occurred in 168 individuals. Mean number of IUTs (2.4; SD = 1.7), gestational age at first IUT (27 weeks; SD = 6), and deliveries by Cesarean within one day of an IUT (19%) were similar to CCAE, while maternal infections requiring hospitalization occurred more often in Medicaid (11.1%). Prevalence of preterm birth (30.8% CCAE; 31.1% Medicaid) and Cesarean section delivery (61.5% CCAE; 61.7% Medicaid) were higher compared to the general pregnant population (9.8% CCAE, 16.9% Medicaid for preterm birth; 34.6% CCAE, 30.0% Medicaid for Cesarean delivery) while the prevalence of preterm premature rupture of membranes was 2–3 times higher in pregnancies with IUTs versus the general pregnant population.

**Conclusion**: Although IUTs were performed at a similar gestational age in the two populations, both subsequent infection and Cesarean section rates were higher in the Medicaid population. A higher rate of premature delivery in patients with IUT than in the general pregnant population is a potential additional source of neonatal comorbidity.

**3:30 PM – 5:00 PM**

## 409 | **The Obstetrical** Transport **Score: A Novel Tool for Guiding Transport Decisions and Predicting Maternal‐Fetal Acuity**

Alixandria F. Pfeiffer^1^; Mikaela Simmang^2^; Sarah A. T. Poor^2^; Michaela M. Kelly^2^; Leslie C. Omeire^3^; Arianna Braddom^2^; Allison Moreno^4^; Patrick S. Ramsey^5^; John J. Byrne^6^

^1^University of Texas Health San Antonio, University of Texas health San Antonio, TX; ^2^University of Texas Health San Antonio, San Antonio, TX; ^3^University of Texas San Antonio, Long School of Medicine, San Antonio, TX; ^4^University Health System, San Antonio, TX; ^5^Department of Obstetrics and Gynecology University of Texas Health Science Center in San Antonio, San Antonio, TX; ^6^University of Texas Health Science Center in San Antonio, San Antonio, TX

**Objective**: To develop and evaluate a novel scoring system designed to predict maternal acuity and optimize triage during interfacility transport to a tertiary center.

**Study Design**: We conducted a retrospective cohort study of pregnant patients transported by a maternal transport team to a tertiary care center between 6/2020 and 7/2024. Patients were included if they were transported by our institutional maternal transport team and were excluded if they had incomplete pre‐transport records. The Obstetrical Transport Score (OTS) was developed using eight maternal‐fetal acuity domains: vaginal bleeding, cervical dilation, rupture of membranes, contraction frequency, intensive care unit (ICU) status, pre‐transport medications, EFM category, and MEWS triggers. Each domain was scored 0–3. Patients were stratified into OTS Levels 1–5: triage, antepartum, L&D, ICU/operating room (OR) readiness, and contraindicated transport. A non‐blinded reviewer retrospectively assigned OTS scores. The primary outcome was escalation of care, defined as L&D or ICU admission and/or delivery within 24 hours. Secondary outcomes included delivery within 4 hours and concordance (“match rate”) between OTS level and actual location of admission. Statistical analyses included Chi‐Square testing and receiver operating characteristic (ROC) curve analysis.

**Results**: Of 936 maternal transports, 304 met inclusion criteria. An OTS ≥3 was significantly associated with escalation of care (sensitivity 94.0%, specificity 73.0%, PPV 89.5%, NPV 81.0%; *p* < 0.001). ROC analysis showed excellent discrimination (AUC 0.91). Higher OTS levels correlated with shorter time to delivery (*p* < 0.01). Delivery within 4 hours occurred in 12.8% of cases and was more likely at OTS Level 4 vs. Levels 1–2 (*p* = 0.004). Match rate between assigned OTS level and actual admitting unit (e.g., L&D, antepartum, ICU) was 84.5%.

**Conclusion**: The OTS demonstrated excellent predictive performance for maternal‐fetal acuity and appropriate unit triage. Its structured scoring of maternal‐fetal indicators can support triage decision‐making during obstetrical transport.

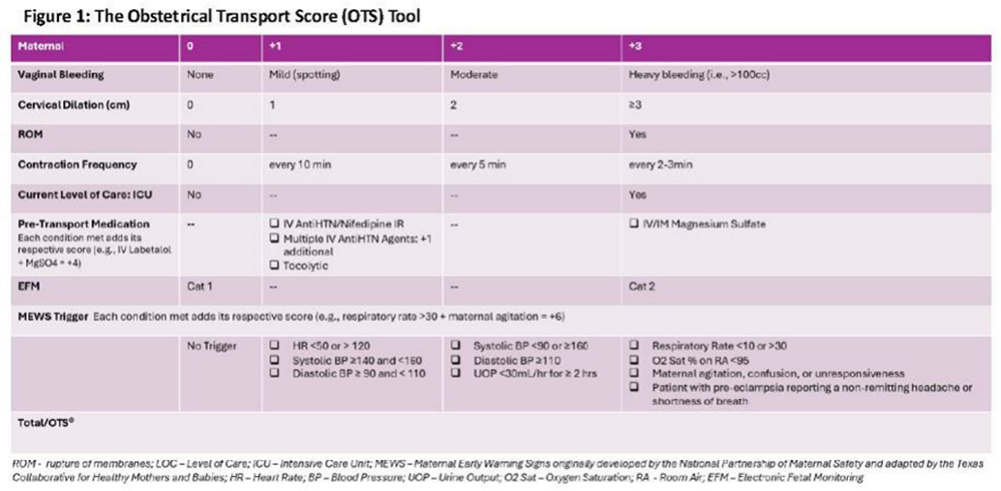

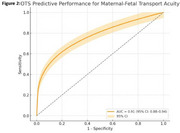

**3:30 PM – 5:00 PM**

## 410 | **Implementation of Universal Hepatitis C Screening**

Allison Kurzeja^1^; Anna Buford^1^; Joyce Miller^2^; Lorre MacDonald^2^; Anne M. Ambia^1^; Jessica E. Pruszynski^1^; Emily H. Adhikari^1^

^1^University of Texas Southwestern Medical Center, Dallas, TX; ^2^Parkland Health, Dallas, TX

**Objective**: To investigate the continuum of care, specifically postpartum follow‐up and treatment, for patients with chronic, untreated hepatitis C after establishment of universal hepatitis C screening and a standardized management protocol.

**Study Design**: This was a retrospective cohort study of patients who delivered at a large public hospital and were screened for hepatitis C. The standardized protocol included evaluation by a maternal fetal medicine specialist for a reactive hepatitis C antibody, a quantitative viral load, liver enzymes, and referral to hepatology in the third trimester if viremic. Electronic medical records were reviewed for laboratory results, hepatology referrals, follow‐up, and treatment.

**Results**: From March 1, 2024 through March 10, 2025, 8971 individuals delivered and were screened for hepatitis C. Of these, 106 (1.2%) had positive hepatitis C antibody testing with 13 (0.14%) with detectable viremia. Ten (0.11%) were newly diagnosed with hepatitis C. Of viremic patients, 12 (92.3%) were referred to hepatology and 8 (66.7%) of referred patients presented for follow‐up by 115 days postpartum. Of the 8 individuals seen by hepatology, 5 initiated treatment between 39 and 357 days postpartum. Ultimately, 3 completed a treatment course and the fourth remains on treatment. The fifth patient discontinued prior to completion due to loss of insurance. Of the 3 who did not initiate treatment, one deferred until breastfeeding completion and has not yet returned to care. Another patient was unable to start due to difficulties with prior authorization, and the last declined to start despite initially filling the prescription.

**Conclusion**: Following universal screening for hepatitis C at a safety‐net hospital, 1.4 per 1000 individuals had detectable viremia. Despite increased screening and detection of this treatable infection, significant barriers remain in place for treatment postpartum. These findings suggest a need to consider available safety data and opportunities for treatment during pregnancy in a high‐risk patient population with significant barriers for postpartum treatment completion.

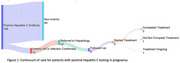

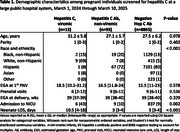

**3:30 PM – 5:00 PM**

## 411 | **Characteristics of Progression of Hypertensive Disorders of Pregnancy**

Allison E. Payne^1^; Laurie B. Griffin^2^; Judith H. Chung^3^; Lynn M. Yee^4^; George R. Saade^5^; Lisa D. Levine^6^; Uma Reddy^7^; C. Noel Bairey Merz^8^; Janet Catov^9^; William A. Grobman^10^; Alisse Hauspurg^11^

^1^Alpert Medical School of Brown University / Women & Infants Hospital of Rhode Island, Providence, RI; ^2^University of Utah, Cottonwood Heights, UT; ^3^University of California Irvine Medical Center, Irvine, CA; ^4^Northwestern University Feinberg School of Medicine, Chicago, IL; ^5^Eastern Virginia Medical School at Old Dominion University, Norfolk, VA; ^6^Perelman School of Medicine, University of Pennsylvania, Philadelphia, PA; ^7^Yale School of Medicine, Yale University, CT; ^8^Cedars‐Sinai Medical Center, Los Angeles, CA; ^9^University of Pittsburgh, Pittsburgh, PA; ^10^Warren Alpert Medical School of Brown University, Providence, RI; ^11^Brown University, Providence, RI

**Objective**: Hypertensive disorders in pregnancy (HDP) occur along a spectrum, and can progress from mild to severe forms. Clinical differences and outcomes between patients with stable versus progressive disease are not well characterized. We sought to explore rates of progression of HDP, and patient characteristics and outcomes associated with progression.

**Study Design**: This is a secondary analysis of the NuMoM2b‐Heart Health Study, a multi‐center cohort study of nulliparous individuals with singleton pregnancies followed during pregnancy through 2–7 years postpartum. We evaluated individuals with no history of hypertension (HTN) and new diagnoses of non‐severe HDP (gestational HTN (GHTN) or preeclampsia without severe features). Stable disease was defined as no change from non‐severe HDP, and progression was defined as progression from non‐severe HDP to preeclampsia with severe features/eclampsia. We compared early pregnancy blood pressures (BP), metabolic biomarkers, obstetric complications, and rates of incident HTN 2–7 years after pregnancy for individuals with stable versus progressive HDP.

**Results**: 2019 individuals met inclusion criteria: 1851 (92%) had no progression of disease and 168 (8%) had progression. Demographic characteristics were similar for individuals with stable versus progressive HDP. Compared to those with stable HDP, individuals with progressive HDP had higher early pregnancy BP and triglycerides, lower NT‐proBNP, and were more likely to have gestational diabetes. Among those with follow up at 2–7 years after delivery, individuals with progressive HDP were significantly more likely than those with stable HDP to have an incident diagnosis of Stage 1 hypertension or greater and to be prescribed anti‐HTN medications.

**Conclusion**: In this multi‐site cohort, 8% of individuals with non‐severe HDP progressed to severe. Progression is associated with differences in baseline pregnancy biometrics, as well as an increased risk of antenatal and postpartum complications, suggesting that knowledge of progression may potentially be informative for peri‐natal and longer lifespan risk stratification.

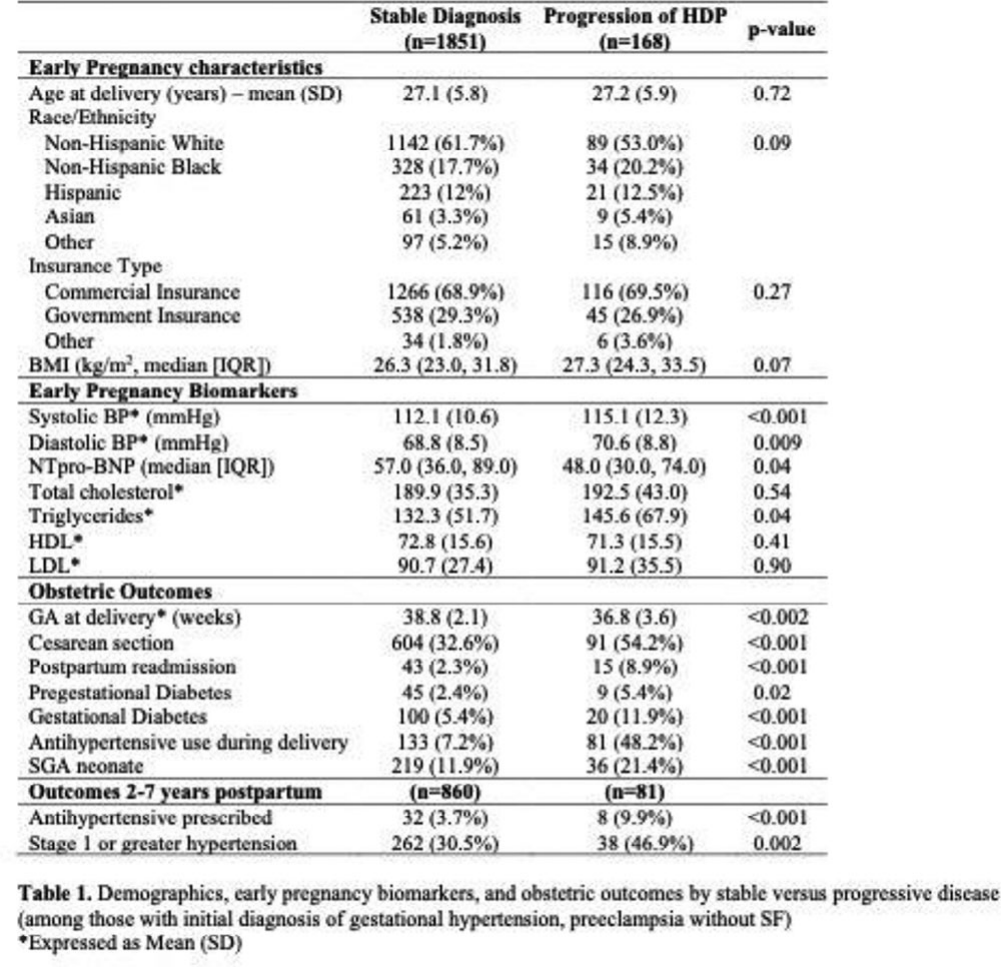

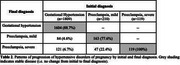

**3:30 PM – 5:00 PM**

## 412 | **Trends in** Hypertensive **Disorders of Pregnancy Highlight Persistent Racial and Rural‐Urban Disparities: A Statewide Analysis**

Alyssa M. Solberg^1^; Kara K. Hoppe^2^; Ronald E. Gangnon^3^; Jacqueline M. Powell^2^

^1^University of Wisconsin‐Madison School of Medicine and Public Health, Madison, WI; ^2^University of Wisconsin School of Medicine and Public Health, Department of Obstetrics and Gynecology, Madison, WI; ^3^University of Wisconsin‐Madison School of Medicine and Public Health, Department of Population Health Sciences, Madison, WI

**Objective**: Hypertensive disorders of pregnancy (HDP) complicate 10% of pregnancies in the U.S. and are associated with an increased risk of morbidity and mortality. This study aims to characterize HDP incidence across Wisconsin (WI) by demographic variables and assess its contribution to trends in severe maternal morbidity (SMM).

**Study Design**: We conducted a retrospective cohort study of all births in Wisconsin from 2017 to 2023 using Wisconsin Hospital Association data. HDP and its subtypes including chronic hypertension, gestational hypertension, pre‐eclampsia, pre‐eclampsia with severe features, and HELLP syndrome were identified using ICD‐10 codes. Incidence rates of HDP per 10,000 births and average annual percent changes (AAPC) were estimated using Poisson regression, both overall and stratified by disease subtype, region, and race/ethnicity. SMM was defined using CDC criteria. We applied the above methods to assess SMM incidence, AAPCs, and the most prevalent SMM indicators by HDP status.

**Results**: Among 388,250 pregnancies analyzed, the incidence of nearly all subtypes of HDP increased from 2017 to 2023. Severe pre‐eclampsia had the highest AAPC at 11.8% (95% CI, 10.6–13%). HDP incidence was highest in urban areas, though the AAPC in pre‐eclampsia was greater in rural versus urban areas at 8.7% (95% CI, 7.1–10.3%, *p* = 0.004). Non‐Hispanic Black patients experienced higher incidences of nearly all HDP subtypes, with significant differences in AAPC for HDP overall, chronic hypertension, and severe pre‐eclampsia across racial/ethnic groups (*p* < 0.001). Patients with HDP had higher rates of SMM compared to those without, though the AAPC in SMM did not significantly differ by HDP status.

**Conclusion**: Our findings highlight a growing burden of HDP in Wisconsin, with a notable rise in severe preeclampsia and disparities by geography and race. Targeted interventions in rural areas and for non‐Hispanic Black patients are needed to reduce preeclampsia risk and SMM across WI. By shedding light on state‐level disparities, studies like this can offer insight into the overall national landscape of maternal health.

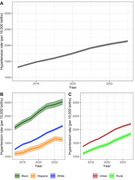

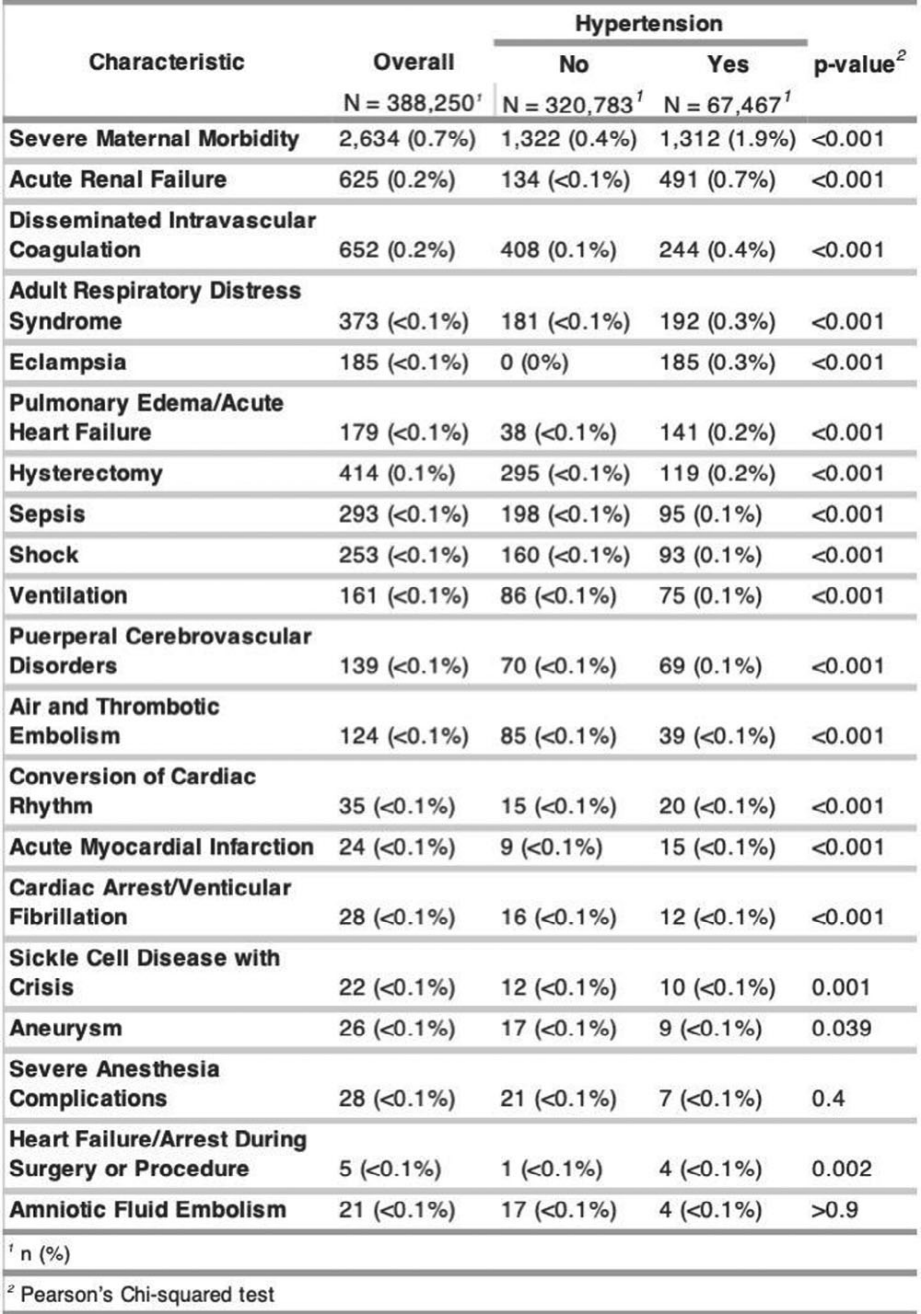

**3:30 PM – 5:00 PM**

## 413 | **A Novel Ultrafast Doppler‐Based Technique for Differentiating Maternal and Fetal Circulations Within the Placenta**

Megan D. Whitham^1^; Amanda DeGraaf^1^; Maegan Gabby^1^; Sofia Issel^2^; Nicholas Joseph^3^; Allison Durica^1^; Yuanbin Zhu^2^; Aiguo Han^2^

^1^Virginia Tech Carilion School of Medicine, Roanoke, VA; ^2^Virginia Tech, Blacksburg, VA; ^3^Carilion Clinic, 24014, VA

**Objective**: To describe a novel imaging method using ultrafast Doppler ultrasound combined with advanced algorithmic analysis to noninvasively differentiate maternal and fetal blood flow within the placenta.

**Study Design**: We developed and tested a placental imaging technique that applies ultrafast Doppler to capture high‐sensitivity perfusion data from the intervillous and fetal vascular spaces using a Verasonics Vantage 256 ultrasound. Ultrafast power Doppler imaging (uPDI) enabled visualization of the primary, secondary and tertiary branches patterns of the placental villous trees. Custom algorithms were applied to pixel‐by‐pixel waveform data to classify flow signals as either maternal or fetal based on three physiological principles: (1) heart rate (2) pulsatility index patterns and (3) localized waveform morphology and flow disturbance features. Composite flow maps were generated to distinguish and visualize the two circulatory systems in vivo.

**Results**: We demonstrate that when paired with ultrafast Doppler data acquisition and uPDI techniques, branching patterns of chorionic villi can be visualized in vivo. We applied an algorithmic approach to separate maternal and fetal signals, producing visually distinct perfusion maps. Our technique identifies fetal circulation based on higher heart rate and low‐resistance flow, and maternal flow by lower‐frequency, higher‐resistance waveforms and greater turbulence near spiral artery entry points. The resulting spatial flow profiles reveal regional heterogeneity.

**Conclusion**: This novel ultrafast Doppler‐based method enables in vivo distinction of villous branching patterns and differentiation of maternal and fetal blood flow within the placenta using a noninvasive, algorithmic approach based on physiologic principles. This technique demonstrates great promise in direct visualization of the microvascular environment. Future work will focus on correlation with pregnancy outcomes and placental histopathology.

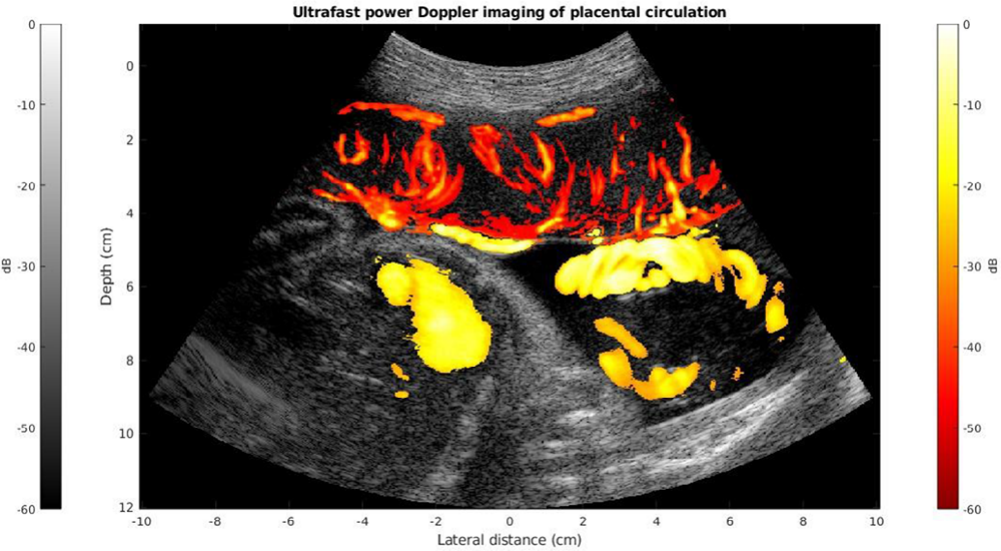

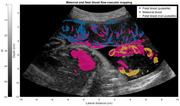

**3:30 PM – 5:00 PM**

## 414 | **Antiseizure** Medication **Use in Pregnancy and Risk of Seizure in Patients With Epilepsy**

Amelie Pham^1^; Chaochen You^2^; Andrew D. Wiese^2^; Andrew J. Spieker^2^; Ashley A. Leech^2^; Margaret A. Adgent^2^; Alexandra Sundermann^2^; Carlos G. Grijalva^2^; Sarah O. Osmundson^2^

^1^Vanderbilt University Medical Center, Nashville, TN; ^2^Vanderbilt University Medical Center, Vanderbilt University Medical Center, TN

**Objective**: Antiseizure medications (ASM) are the primary method for epileptic seizure control in pregnancy. We examined the association between ASM adherence and the risk of seizure in pregnant individuals with epilepsy.

**Study Design**: This nested case‐control study examined a retrospective cohort of pregnant individuals continuously enrolled in Tennessee Medicaid (2007–2019). Linked maternal‐infant data included healthcare claims and discharge data, prescription fills, and birth certificates. Pregnancies with ≥ 20 weeks’ gestation among individuals aged 15–44 with epilepsy diagnosis prior to conception and ASM exposure were included. Seizures during pregnancy were identified using ICD 9/10 and CPT code algorithms (Table 1). Each case was matched to up to 10 controls with replacement by gestational age at first seizure (index date, ±2 days). ASM adherence was measured as the proportion of days covered during a 60‐day window before the index date. We used conditional logistic regression to estimate the association between ASM adherence prior to seizure onset and seizure risk in pregnancy, adjusting for a‐priori selected confounders and using cluster‐robust standard errors.

**Results**: Among 1491 eligible pregnancies, we examined 354 cases (24%) and 1468 matched controls (Table 1). Most seizures occurred in the first trimester (47%, 168/354). Mean ASM adherence was low in both cases and controls. Each 10% increase in ASM adherence was associated with 5.5% higher odds of seizure (95% CI: 1.30%–9.87%, Table 2). Findings were similar when excluding cases of index seizure date within 60 days of conception.

**Conclusion**: Among pregnant patients with epilepsy, ASM adherence was low, and seizures were common. Preliminary analysis noted that a small increase in ASM adherence was associated with higher odds for seizure events. Further analyses should examine whether pre‐pregnancy epilepsy severity impacts ASM adherence and seizure control during pregnancy.

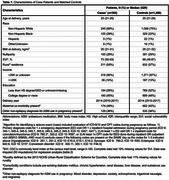

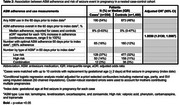

**3:30 PM – 5:00 PM**

## 415 | **Effect of** Intravenous **Fluid Restriction on Nulliparous, Term, Singleton, Vertex Cesarean Delivery Rates**

Amin Tavakoli^1^; Catriona Lewis^2^; Naomi H. Greene^3^; Gabriela Dellapiana^4^

^1^Cedars Sinai Medical Center, Los Angeles, CA; ^2^Cedars‐Sinai Health Sciences University, Los Angeles, CA; ^3^Department of Obstetrics & Gynecology, Cedars‐Sinai Medical Center, Los Angeles, CA; ^4^Cedars‐Sinai Medical Center, Los Angeles, CA

**Objective**: During the 2024 nationwide intravenous (IV) fluid shortage, our hospital implemented a restricted protocol for laboring patients. Previously, patients received 125 mL/hr of maintenance IV fluids; during the shortage, this was reduced to 10 mL/hr to keep the line open while encouraging oral hydration. Given limited data on optimal IV fluid rate in labor, we evaluated whether IV fluid restriction in labor affected cesarean delivery (CD) rates among nulliparous, term, singleton, vertex (NTSV) pregnancies.

**Study Design**: This was a retrospective cohort study of all NTSV patients at a quaternary care hospital. Patients laboring during the IV fluid shortage (minimal fluid group; 10/2024–2/2025) were compared to those laboring during the same months in the two preceding years (standard fluid group; 10/2022–2/2023 and 10/2023–2/2024). Data on demographics, labor characteristics, and delivery outcomes were abstracted from medical records. Our primary outcome was NTSV CD rate and our secondary outcomes included total IV fluid intake in labor and total labor time. T‐test, chi‐square, and Wilcoxon rank‐sum were used as appropriate.

**Results**: Of 2506 NTSV patients, 832 were in the minimal IV fluid group and 1674 were in the standard IV fluid group. The minimal IV fluid group had higher birth weights and was more likely to have public insurance. The minimal IV fluid group received less total IV fluids during labor (median [IQR] 873 mL [1124] vs. 2365 mL [1997], *p* < 0.01). There was no difference in NTSV CD rates (28.4% vs 30.7%, *p* = 0.23), indication for cesarean delivery, or in total labor time (median [IQR] 16.7 hours [13.7] vs. 16.4 hours [14.0], *p* = 0.23) between the groups. However, Terbutaline was administered more in the minimal fluid group (12.9% vs. 8.8%, *p* < 0.01).

**Conclusion**: Cesarean delivery rates in NTSV patients were not negatively impacted by a minimal IV fluid protocol, suggesting that oral hydration may be sufficient in resource‐limited settings. However, increased need for terbutaline in this group does suggest higher rates of tachysystole, so attention to fetal monitoring should be emphasized.

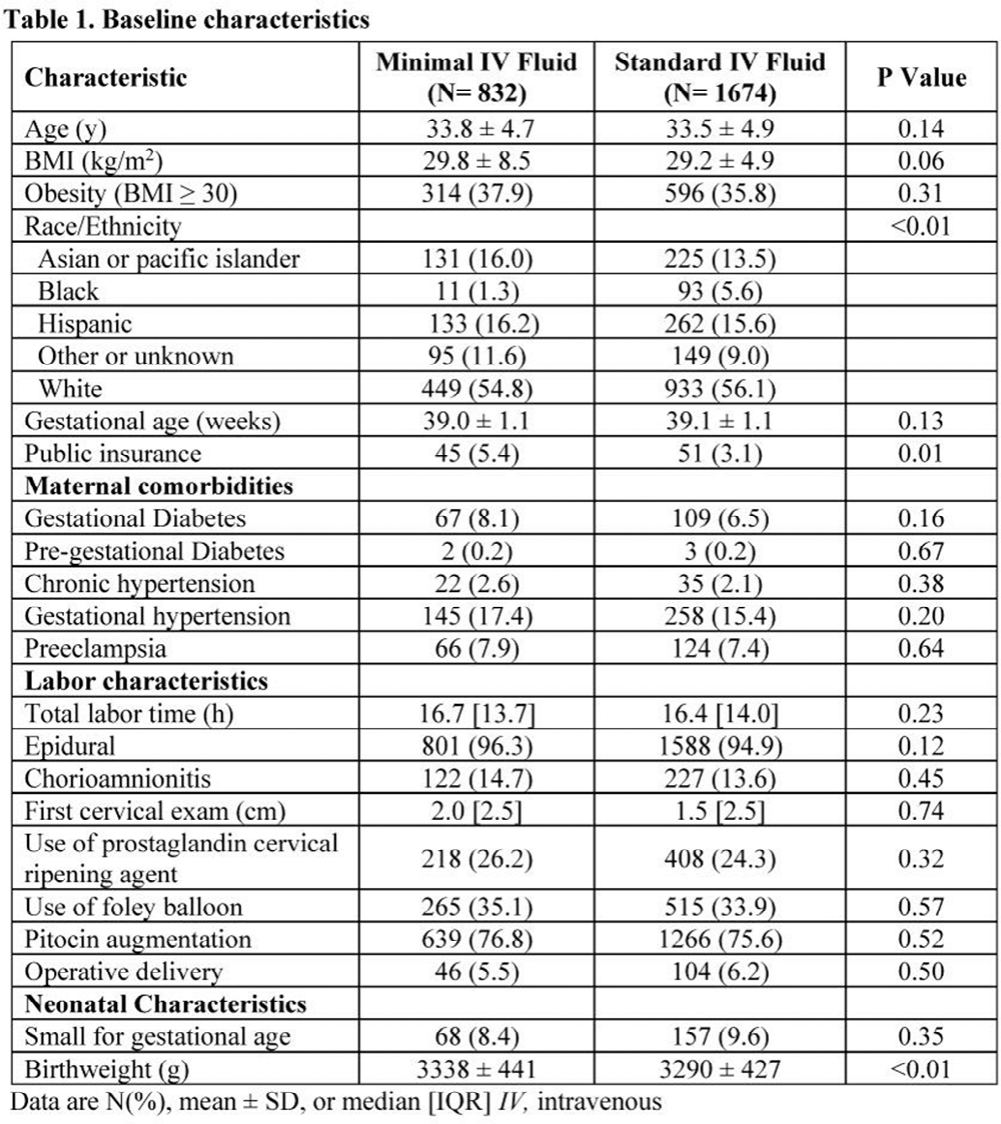

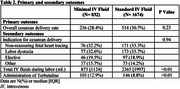

**3:30 PM – 5:00 PM**

## 416 | **Preterm Birth** After **Antenatal Corticosteroid Treatment: Associations With Long‐Term Infectious Diseases**

Amir Snir^1^; Gali Pariente^2^; Tamar Wainstock^3^; Eyal Sheiner^4^

^1^Soroka University Medical Center, omer, HaDarom; ^2^Department of Obstetrics and Gynecology, Soroka University Medical Center, Ben‐Gurion University, beer sheva, HaDarom; ^3^Department of Epidemiology, Biostatistics and Community Health Sciences, Faculty of Health Sciences, Ben‐Gurion University of the Negev, Beer Sheva, HaDarom; ^4^Soroka University Medical Center, Faculty of Health Sciences, Ben‐Gurion University of the Negev, beer sheva, HaDarom

**Objective**: Antenatal corticosteroids (ACS) are routinely administered to pregnancies at risk for preterm birth due to their proven benefits in reducing neonatal morbidity and mortality. However, the long‐term effects of ACS in this population are not fully understood. We aimed to evaluate the risk of childhood infectious morbidity among premature infants born following ACS exposure before 34 weeks.

**Study Design**: A population based cohort study was conducted at a tertiary referral center, including singleton preterm births (<37 weeks gestation) between 1991 and 2021. Long‐term infectious morbidity of preterm offspring who were exposed to ACS prior to 34 weeks gestation was compared with unexposed children, based on combined databases from both community and hospitalization records. Cumulative incidence was estimated using Kaplan–Meier survival curves and Cox proportional hazards models were employed to adjust for potential confounders.

**Results**: Among 13,580 preterm births, 1538 neonates (11.3%) were exposed to ACS before 34 weeks gestation. The incidence of infectious morbidities was significantly higher in the exposed group (387.6 vs. 177.5 per 1000 person years, Table; Kaplan–Meier log‐rank *p* < 0.001; Figure). In a multivariable Cox model, adjusting for maternal age, mode of delivery, hypertensive disorders, diabetes mellitus, and gestational age, ACS exposure remained independently associated with increased risk for long term infectious morbidity of the offspring (aHR = 1.15; 95% CI 1.11–1.19; *p* < 0.001). Subgroup analyses by gestational age demonstrated consistent findings, with the strongest association observed among extremely preterm infants (<28 weeks, Kaplan–Meier log‐rank *p* < 0.001; Figure).

**Conclusion**: ACS exposure before 34 weeks of gestation was associated with increased risk of infectious morbidity in children born preterm. While ACS remains essential in managing threatened preterm birth, this association may partly reflect indication bias, as more vulnerable fetuses are more likely to receive treatment. Further research is warranted to clarify long‐term outcomes and guide clinical decision‐making.

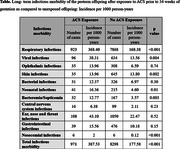

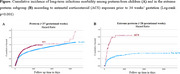

**3:30 PM – 5:00 PM**

## 417 | **Temporal** Changes **in Injury Patterns and Outcomes in Obstetric Trauma at One Academic Center (1994–2023)**

Ana Collins‐Smith; Megan Zachariah; Celeste Traub; Aylia Rizvi; Paloma Rodriguez Paramo; Leah Saylor; Sourabh Sharma; Micheal Erickson

University of Texas Medical Branch, Galveston, TX

**Objective**: Trauma is the leading cause of nonobstetric death among pregnant women. But management of obstetrical traumas can vary from the general population and require special attention. Trauma centers with readily available obstetrical services are ideal for the management of obstetrical patients with traumatic injuries. The objective of this study was to investigate long‐term trends in mortality, mechanism of trauma, and rate of admissions in pregnant patients.

**Study Design**: We conducted a retrospective analysis of obstetrical traumas at a single academic level one trauma center over 29 years (1994–2023). Demographic factors, injury characteristics, admitting service, age at time of trauma, and admitting service were examined for temporal trends over ten‐year increments. Temporal changes in mechanism of injury, rate of admission, and admitting service were analyzed using logistic regression analysis in ten‐year increments. Factors associated with independent temporal trends were analyzed using ordinal logistic regression modeling.

**Results**: During the study period, there were 1688 encounters for obstetrical trauma, with a predominance of motor vehicle collisions (50.5%) and falls (29.7%). Trends were observed with increases in motor vehicle accidents (MVA) (*p* < 0.001) & all‐terrain vehicle accidents (*p* < 0.003). Additional trends were noted with decreases in assaults (*p* < 0.002), falls (*p* < 0.00001), and deaths (*p* = 0.3).

Over the study period in ten‐year increments, lower rates of admission to the hospital and admission to the obstetrical service was observed.

**Conclusion**: Throughout the decades, with advancement in science and changes in medical society guidelines; there has been a decrease in the amount of posttraumatic pregnant patients admitted and a statistically significant decrease in gravid patients admitted to the surgical service (general surgery or acute trauma care).

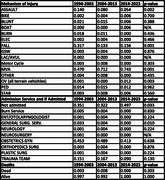

**3:30 PM – 5:00 PM**

## 418 | **Perinatal** Outcomes **of Fgr‐Infants ≥26 Weeks and <500G Compared to <26 Weeks Neonates With Paired Birthweight**

Ambrogio Pietro Londero^1^; Enrico Corno^2^; Chiara Melito^3^; Maria Grazia Capurso^3^; Carolina Scala^4^; Federico Prefumo^4^; Andrea Dall'Asta^3^

^1^Department of Neuroscience, Rehabilitation, Ophtalmology, Genetics, Maternal and Child Health–DINOGMI, University of Genoa, University of Genova, Liguria; ^2^Department of Medicine and Surgery, Obstetrics and Gynecology Unit, University of Parma, Parma, Italy, Parma, Emilia‐Romagna; ^3^Department of Medicine and Surgery, Obstetrics and Gynecology Unit, University of Parma, Parma, Italy, University of Parma, Emilia‐Romagna; ^4^Unit of Obstetrics and Gynecology IRCCS Giannina Gaslini Institute, Genova, Italy, IRCCS Giannina Gaslini Institute, Genova, Liguria

**Objective**: To investigate the perinatal survival of fetal growth restriction (FGR) with birthweight (BW) <500 grams beyond 26^+0^ weeks and compare their outcome with non‐FGR neonates paired for BW born prior to 26^+0^ weeks.

**Study Design**: Retrospective study on data from the U.S. National Center for Health Statistics birth certificate data 2011‐to‐2023. Singleton pregnancies with delivery of a neonate weighting <500 grams between 22^+0^ and 31^+6^ weeks were included. For each infant, the corresponding birthweight percentile was calculated according to the Fenton growth curve. Fetuses born prior to 26^+0^ weeks with BW < 3rd percentile were excluded.

**Results**: 18230 cases were included, of whom 5752 were FGR with BW <500 grams born beyond 26^+0^ weeks. Their perinatal survival rate declined with advancing gestation from 26 to 31 weeks (66.8% vs 44.5%, respectively, *p* < 0.01) (Figure 1). Outcome comparison between cases of FGR with BW <500 grams born >26^+0^ weeks and non‐FGR with BW <500 grams <26^+0^ weeks showed a lower rate of chorioamnionitis (2.1% vs 6.9%, *p* = 0.01) and vaginal delivery (28% vs 68%, *p* < 0.01) and a higher frequency of antenatal corticosteroids administration (33.7% vs 21.5%, *p* < 0.01) and preeclampsia (23.9% vs 0.8%, *p* < 0.01) in FGR neonates born beyond 26^+0^ weeks. The overall survival rate was higher for FGR neonates born beyond 26^+0^ weeks compared to non‐FGR neonates born prior to 26^+0^ weeks (63.4% vs 38.1%, *p* < 0.01).

**Conclusion**: In FGR with BW <500 grams beyond 26^+0^ weeks, the perinatal survival rate is inversely correlated with the gestational age. However, this group shows an overall higher survival rate compared to non‐FGR neonates born below 26 weeks with paired birthweight.

**3:30 PM – 5:00 PM**

## 419 | **Social** Vulnerability **and Racial Disparities in Preterm Birth: A National Cohort Study**

Anita Pershad^1^; Gabriella Adams^2^; Tetsuya Kawakita^3^

^1^Eastern Virginia Medical School, silver Spring, MD; ^2^Eastern Virginia Medical School Macon & Joan Brock Virginia Health Sciences, Norfolk, VA; ^3^Eastern Virginia Medical School Department of Obstetrics and Gynecology, Macon & Joan Brock Virginia Health Sciences at Old Dominion University, Norfolk, VA

**Objective**: To evaluate the association between county‐level social vulnerability and preterm birth, and assess whether racial disparities vary across levels of social vulnerability.

**Study Design**: This cross‐sectional study used restricted Centers for Disease Control and Prevention natality data from 2016–2021. We included Black or White individuals aged 15–44 years with singleton pregnancies across 3114 U.S. counties. Participants were grouped into quartiles based on the county‐level Social Vulnerability Index (SVI) (Figure 1). The primary outcome was preterm birth, defined as delivery before 37 weeks. Secondary outcomes included small for gestational age (SGA) (birth weight <10th percentile), pregnancy‐related hypertension (HTN), and no prenatal care. We used mixed‐effects generalized linear models with Poisson distribution to calculate average marginal effects (AMEs) with 95% confidence intervals (95% CI), adjusted for confounders. Difference‐in‐differences (DID) was calculated for the difference in Black‐White disparity across SVI quartiles, with quartile 1 as a reference.

**Results**: A total of 2,198,560 individuals were included: 368,256 in quartile 1, 554,767 in quartile 2, 632,717 in quartile 3, and 642,820 in quartile 4. Black individuals had higher adjusted rates of preterm birth than White individuals in all quartiles. Black and White preterm birth rates were 12.1% vs. 9.6% (AME 2.5%, 95% CI 1.9–3.0) in quartile 1; 13.2% vs. 9.7% (AME 3.5%; 95% CI 3.1–3.9) in quartile 2, 13.6% vs. 10.1% (AME 3.5; 95% CI 3.1–3.8) in quartile 3; and 14.6% vs. 10.4% (AME 4.24%, 95% CI 3.89–4.58) in quartile 4 (Table 1). The DID as a disparity indicator increased as social vulnerability increased; DID was statistically significant in quartiles 2–4 compared to quartile 1. Secondary outcomes had similar trends, with widening disparities in pregnancy‐related HTN, SGA, and prenatal care.

**Conclusion**: Increasing social vulnerability is associated with widening racial disparities in preterm birth and other adverse pregnancy outcomes. These findings underscore the need to address community‐level influences on perinatal inequities.

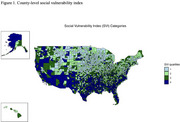

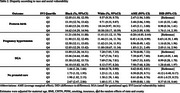

**3:30 PM – 5:00 PM**

## 420 | **Adverse** Perinatal **Outcomes Associated With Anemia in Early and Late Pregnancy**

Anna Booman^1^; Brian T. Bateman^1^; Sara Siadat^1^; Caroline Berube^1^; Irogue I. Igbinosa^1^; Cecilia Leggett^2^; Deirdre J. Lyell^3^; Elliott K. Main^4^; Stephanie A. Leonard^5^

^1^Stanford University, Stanford, CA; ^2^Stanford University School of Medicine, Palo Alto, CA; ^3^Division of Maternal‐Fetal Medicine, Department of Obstetrics and Gynecology, Stanford School of Medicine, Stanford, CA; ^4^Stanford University School of Medicine, Stanford, CA; ^5^Stanford University, Stanford University, CA

**Objective**: Anemia in early pregnancy may lead to adverse perinatal outcomes, but evidence is limited. Further, it is unknown if resolution of anemia during pregnancy mitigates the risk. We evaluated perinatal outcomes of those with anemia in the first trimester and of those with resolved and persistent anemia by late pregnancy, compared with those without anemia in the first trimester.

**Study Design**: We used the Merative™ Marketscan^®^ Commercial Database of nationwide insurance claims data during 2018–2023. We identified anemia with trimester‐specific thresholds for hemoglobin (Hgb) and hematocrit (Hct). We classified anemia as resolved if ≥1 normal Hg/Hct value in late pregnancy (≥24 weeks’ gestation) and as persistent otherwise. We defined perinatal outcomes using diagnosis and procedure codes. We used inverse probability of treatment weights and modified Poisson regression to estimate associations between anemia and anemia resolution with perinatal outcomes.

**Results**: Among 73,586 pregnancies, 4.4% had anemia in the first trimester. Of those with ≥1 Hgb/Hct value in late pregnancy (72.1%), 53.4% had persistent and 46.6% had resolved anemia. Compared with those without anemia in the first trimester, those with anemia, regardless of resolution, had at least twofold higher risk of blood products transfusion and severe postpartum hemorrhage and 46% higher risk of non‐transfusion severe maternal morbidity (ntSMM) (adjusted risk ratio [aRR] 2.45, 95% confidence interval [CI]: 1.91, 3.13; aRR 1.99, 95% CI: 1.39, 2.85; and aRR 1.46, 95% CI: 1.13, 1.89) (Figure 1). Risk of ntSMM was increased among those with persistent anemia (aRR 1.64, 95% CI: 1.13, 2.37), but not among those with resolved anemia (aRR 1.07, 95% CI: 0.65, 1.74) (Figure 2). Risk of other outcomes was similar for those with persistent and resolved anemia.

**Conclusion**: Findings suggest that anemia in the first trimester increases risk of adverse perinatal outcomes, and resolution of anemia by late pregnancy reduces but does not eliminate risk. This research emphasizes the clinical importance of anemia in early pregnancy and the need for adequate treatment.

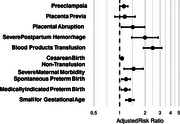

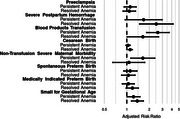

**3:30 PM – 5:00 PM**

## 421 | **Histopathologic** Changes **Associated With Treponema Pallidum Detection in Placental Tissues**

Anna Buford^1^; Sophie Gao^1^; Kristen A. Warncke^1^; Shreya Battu^1^; Jeffrey SoRelle^1^; Daniel Montelongo Jauregui^1^; Jessica E. Pruszynski^1^; Rebecca Collins^2^; Emily H. Adhikari^1^

^1^University of Texas Southwestern Medical Center, Dallas, TX; ^2^Parkland Health, Dallas, TX

**Objective**: Characterize histopathologic changes in syphilis‐exposed placentas with and without immunohistochemical (IHC) detection of *Treponema pallidum* in placental tissues.

**Study Design**: This is a retrospective cohort study of patients delivered after syphilis diagnosis at a large public hospital. Among placentas for which placental pathologic evaluation was available and IHC stain on umbilical cord or placental tissues was performed for detection of *T. pallidum*, we compared maternal syphilis characteristics and histopathologic changes among IHC+ and IHC‐ placentas.

**Results**: From January 1, 2010, through June 30, 2025, 126 placentas from gravidas with syphilis in pregnancy had IHC stain, including 22 IHC(+) and 104 IHC(‐). Early syphilis, higher maternal RPR, later EGA at diagnosis, lack of benzathine penicillin G (BPG) exposure before delivery, and stillbirth were more common among IHC+ placentas. Congenital syphilis was diagnosed among 100% of IHC+ and 18(18%) of IHC‐ groups. Among gravidas treated prenatally, interval (d) to delivery was shorter (6 [3–17] vs 136[66–180], *p* < 0.001] with odds of IHC+ decreased by 97% if the BPG‐delivery interval was more than 17 days. IHC positivity was associated with LGA placentas, acute maternal and fetal inflammatory responses, and specific villous changes including increased histiocytic villitis with Hofbauer cells and increased fetal RBCs. Maternal vascular lesions suggestive of malperfusion were not different. Among fetal villous or vascular lesions, villous karyorrhexis and vascular occlusion or involution in chorionic plate vessels were more common in IHC+ placentas.

**Conclusion**: Immunohistochemical staining for *Treponema pallidum* assists with characterization of congenital syphilis‐associated placental histopathologic changes. Earlier diagnosis and treatment of gravidas with syphilis decreases the acute maternal and fetal inflammatory response in the placenta linked to prematurity, fetal distress and stillbirth.

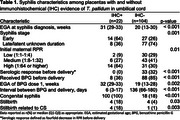

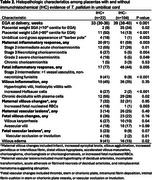

**3:30 PM – 5:00 PM**

## 422 | **Maternal** Hypoalbuminemia **And Complications of Hypertensive Disorders of Pregnancy**

Anna K. Daoud^1^; Mirely Garcia^2^; Julia Rubin^3^; Ester Sanchez^4^; Kristin M. Voegtline^5^; Moeun Son^6^

^1^New York Presbyterian/ Weill Cornell Medicine, New York, NY; ^2^NewYork‐Presbyterian/Weill Cornell Medicine, NewYork‐Presbyterian/Weill Cornell Medical Center, NY; ^3^Weill Cornell Medical College, Weill Cornell Medical College, NY; ^4^NewYork‐Presbyterian/Weill Cornell Medicine, NewYork‐Presbyterian/Weill Cornell Medicine, NY; ^5^NewYork‐Presbyterian/ Weill Cornell Medicine, New York, NY; ^6^Weill Cornell Medical College, New York, NY

**Objective**: Serum albumin is physiologically decreased during pregnancy, but can be further decreased in individuals with preeclampsia due to increased vascular permeability and endothelial dysfunction. Among patients with hypertensive disorders of pregnancy (HDP) without severe features at delivery admission, we aimed to evaluate if hypoalbuminemia is associated with postpartum complications.

**Study Design**: This is a retrospective cohort study of pregnant persons aged 18–50 years with singleton term gestations who were diagnosed with HDP without severe features and delivered at two NYC hospitals from 2020 to 2024. Those with chronic hypertension, kidney or liver disease, or missing albumin values were excluded. Eligible individuals were identified via a research database, but data were manually extracted from the medical record. Hypoalbuminemia was defined as < = 2.3 g/dL based on prior literature. Study outcomes included development of severe features, magnesium sulfate infusion, antihypertensive use, highest blood pressures, and hospital representation. Patients with low serum albumin were compared to those with values >2.3 g/dL using Fisher's exact and Chi‐square tests with Bonferroni correction for multiple comparisons. Logistic regression was performed.

**Results**: Of 572 eligible patients, 231 (40.4%) had hypoalbuminemia during their delivery encounter. Baseline characteristics are shown in Table 1. Patients with hypoalbuminemia were significantly more likely to develop severe features (OR 3.91 95% CI 2.58–5.94), receive antihypertensives during delivery admission (OR 3.60 95% CI 2.16–6.02) and upon discharge (OR 3.01 95% CI 2.13–4.25), and had higher blood pressures postpartum (*p* < .0001) (Table 2).

**Conclusion**: Among patients with HDP without severe features who delivered at term, hypoalbuminemia at admission was associated with the development of severe features and other HDP complications. Albumin levels are readily available in basic metabolic panels routinely collected for HDP and could be used to identify patients who are at increased risk for HDP complications.

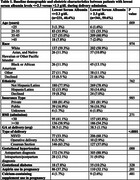

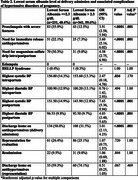

**3:30 PM – 5:00 PM**

## 423 | **A Case‐Control** Study **Of Uterine Rupture in Caluquembe, Angola, 2019–2025: Adverse Obstetric and Perinatal Outcomes**

Anna E. Eberwein^1^; Jasmine J. Su^2^; Priscila Ribeiro Cummings^3^; Wan‐Ju Wu^1^; Julia Andre^2^; Perreira Goa^2^; Kathryn H. Jacobsen^4^

^1^Icahn School of Medicine at Mount Sinai, New York, NY; ^2^Hospital Evangelico de Caluquembe, Caluquembe, Huila; ^3^Hospital Evangelico de Caluquembe, Kalukembe, Huila; ^4^University of Richmond, Richmond, VA

**Objective**: Our rural district hospital serves remote and sometimes inaccessible regions of Huila province, Angola, where two physicians and two technicians provide surgical care for a catchment area of more than half a million people. Although many cases are preventable, uterine rupture remains a leading cause of maternal mortality in Angola. We sought to identify risk factors for poor maternal and fetal outcomes related to uterine rupture.

**Study Design**: We conducted a date‐of‐delivery‐matched case‐control study of 187 uterine ruptures and 187 controls in Hospital Evangélico de Caluquembe in Huila province, Angola in 2019–2025. Odds ratios and logistic regression were used to compare the two populations.

**Results**: Thirteen uterine rupture cases resulted in maternal mortality, and a live birth was documented in only 22 cases. Cases had greater odds of maternal death (OR = 7.8 (2.0, 52.0)) and stillbirth (OR = 89.9 (44.1, 195.1)). Increased time from home to the hospital in hours (OR = 1.2 (1.1, 1,4)) and multiparity (OR = 1.3 (1.1, 1.6)) were both associated with stillbirths. Five surviving uterine ruptures received hysterectomy rather than uterine repair. Additionally, bladder rupture was documented in five cases.

**Conclusion**: Building capacity for surgical delivery at critical access hospitals, strengthening referral networks for women in labor in rural areas, and increasing access to modern contraception among women with higher parity are necessary for reducing burden from uterine rupture.

**3:30 PM – 5:00 PM**

## 424 | **National trends** in **antepartum hospitalization rates in a healthcare claims database, 2011–2021**

Anna C. Jarvis^1^; Alison N. Goulding^2^; Lisa Yanek^1^; Marika Toscano^3^

^1^Johns Hopkins University, Johns Hopkins University, MD; ^2^Baylor College of Medicine, Houston, TX; ^3^Johns Hopkins University, Baltimore, MD

**Objective**: Extended antepartum hospitalizations (EAH) for medical and obstetric complications of pregnancy, such as preterm pre‐eclampsia with severe features, preterm prelabor rupture of membranes, and vasa previa, have significant economic and social costs, as well as demonstrated impacts on maternal mental health. However, epidemiologic estimates of EAH are >20 years outdated. The objective of this study was to describe the national prevalence and temporal trends over time of EAH during pregnancy using administrative data from a large insurance claims database.

**Study Design**: Using the MarketScan Database, a national convenience sample of commercially insured individuals, we conducted a retrospective analysis of EAH from 1/1/2011 to 12/31/2021. Included were women aged 13–64 with continuous insurance enrollment from 9 months before to 6 months after a delivery. Pregnancies were identified using a published validated algorithm based on diagnosis, procedure, and DRG codes. EAH was defined as a pregnancy‐related admission ≥4 days occurring within 259 days before delivery, excluding delivery‐only hospitalizations. The primary outcome was the proportion of individuals with EAH; the secondary outcome was annual percent change (APC) in EAH rates. Data were extracted using SAS v 9.4.

**Results**: There were 2,009,385 unique pregnancies among commercially insured patients in the United States from 2011 to 2021 (Table 1), with a total of 64,868 antepartum hospitalizations (3.2%), 14,684 (0.73%) of which were extended. The average annual percent change in EAH from 2011–2021 was ‐6.59%, with a standard deviation of 8.59% (Figure 1).

**Conclusion**: From 2011–2021, 0.73% of commercially insured pregnancies in the U.S. had an EAH prior to delivery. EAH rates nationally declined by an average of 6.59% over this time period. Findings reflect unweighted data from a commercially insured population and exclude antepartum admissions ending in discharge with childbirth. Further studies are needed to evaluate the financial, social, and care burdens associated with EAH.

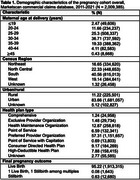

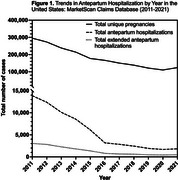

**3:30 PM – 5:00 PM**

## 425 | **Glycemic Control and Pregnancy Outcomes among Individuals Using Hybrid Closed‐Loop Therapy**

Annabeth Brewton^1^; Radhika A. Pandit^2^; Jiqiang Wu^1^; Natasha Santhanam^3^; Ellena Privitera^3^; Shaylyn Vickers^1^; Steven Gabbe^1^; William A. Grobman^4^; Mark B. Landon^5^; Christine P. Field^5^; Elizabeth O. Buschur^1^; Kartik K. Venkatesh^5^

^1^The Ohio State University Wexner Medical Center, Columbus, OH; ^2^The Ohio State University Department of Obstetrics and Gynecology, Columbus, OH; ^3^The Ohio State University College of Medicine, Columbus, OH; ^4^Warren Alpert Medical School of Brown University, Providence, RI; ^5^The Ohio State University Wexner Medical Center, The Ohio State University Wexner Medical Center, OH

**Objective**: Pregnant individuals with type 1 diabetes (T1D) increasingly utilize hybrid closed‐loop (HCL) therapy with continuous glucose monitoring (CGM), which offers two modes for insulin delivery: automated (HCL) and manual (sensor augmented pump therapy, [SAPT]). While international data demonstrate pregnancy‐specific automated HCL is associated with improved glycemic control, such pregnancy‐specific algorithms are not available in the US. Hence, individuals with T1D may use manual SAPT or automated HCL with off‐label use in pregnancy. We compared glycemic management and pregnancy outcomes between those using automated HCL versus manual SAPT.

**Study Design**: A retrospective analysis of pregnant individuals with T1D using HCL enrolled in an integrated prenatal and diabetes care program from 2022–2024. The exposure was intent to use automated HCL (using non‐pregnancy algorithms) versus manual SAPT (reference) at prenatal care initiation. Outcomes included glycemic control (A1c, CGM metrics) and pregnancy outcomes (cesarean delivery, HDP, PTB, LGA, NICU admission, hypoglycemia, hyperbilirubinemia, and RDS). An Inverse‐Probability‐of‐Treatment Weighting analytic approach was used to address confounding by insulin pump type, duration of T1D, and early pregnancy A1c.

**Results**: Among 138 pregnant individuals with T1D using HCL, 67% (*n* = 93) used automated mode and 32.6% (*n* = 45) used manual SAPT (median gestational age: 8 weeks). Individuals with automated HCL were more likely to have an Omnipod or Tandem pump and have a lower early pregnancy A1c, and had less time below range compared with those in manual mode (p<0.10) (Table 1). In unadjusted analyses, individuals using automated HCL were less likely to have a cesarean delivery, PTB, and time below range compared with those using manual SAPT. Following adjustment, glycemic control and pregnancy outcomes did not differ between groups.

**Conclusion**: Glycemic control and pregnancy outcomes were similar for those using automated HCL and manual SAPT. Whether outcomes can be further optimized with pregnancy‐specific HCL algorithms in the US is a research priority.

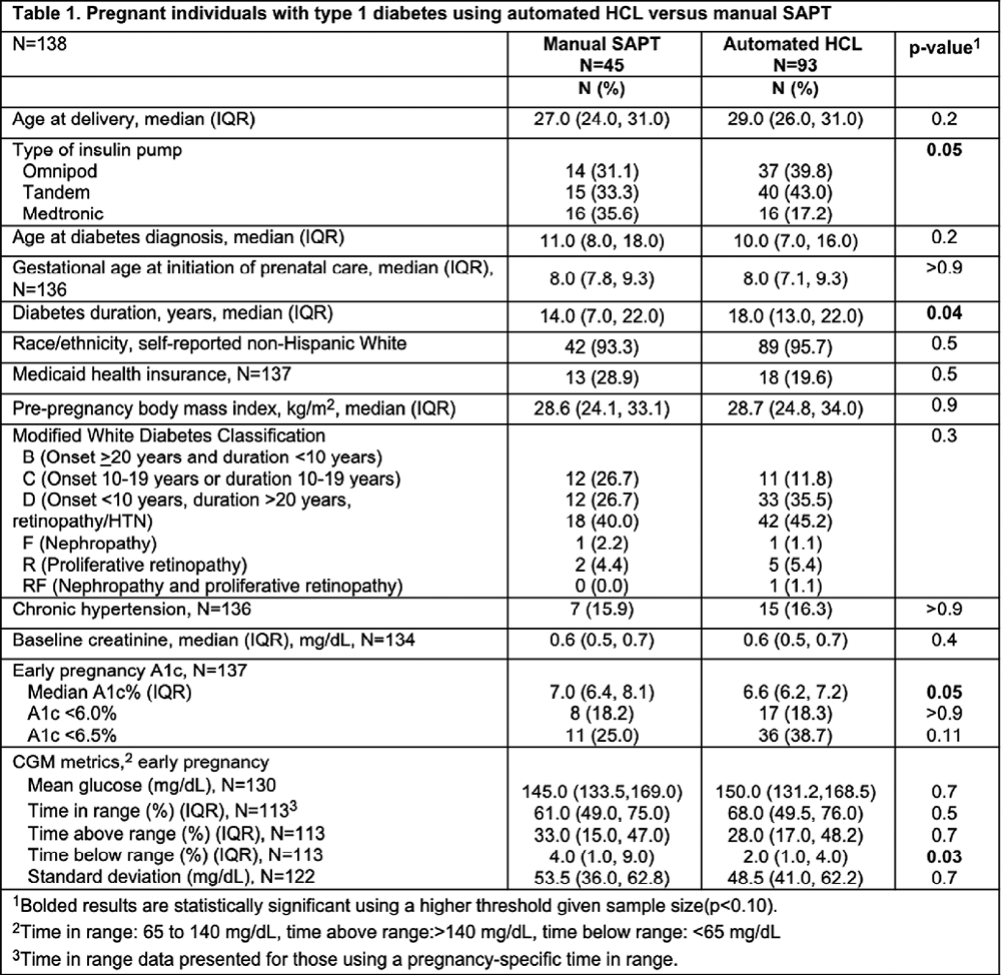

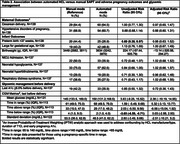

**3:30 PM – 5:00 PM**

## 426 | **Serial Sebum** Sampling **In Intrahepatic Cholestasis of Pregnancy**

Anne M. Ambia^1^; Jeffrey McDonald^2^; Yevgenia Y. Fomina^3^; Tamia Harris‐Tryon^2^; Andrea Rizkallah^2^; C. Edward Wells^1^; Catherine Y. Spong^1^; Donald D. McIntire^1^; David B. Nelson^1^

^1^University of Texas Southwestern Medical Center, Dallas, TX; ^2^University of Texas Southwestern, Dallas, TX; ^3^University of Texas Southwestern, university of Texas Southwestern, TX

**Objective**: Intrahepatic cholestasis of pregnancy (ICP) is characterized by elevated bile acids as a disease unique to pregnancy. Because our group has previously demonstrated the ability to detect individual bile acids through sebum sampling, we aimed to evaluate serial sebum sampling on patients with ICP receiving ursodiol treatment.

**Study Design**: This was a prospective, observational cohort study of pregnant individuals with confirmed ICP (defined by total serum bile acids ≥10 µmol/L) from December 2023 to July 2024 at a single academic center. At the time of initial evaluation for ICP, sebum sampling was performed after preparation of the skin on the upper back to be sampled with an alcohol wipe cleanse. Three to five strips of sebutape^®^ were placed for 15 minutes and removed. A second sampling was done at approximately 35–38 weeks at least 2 weeks after the initial analysis. Extraction of bile acids were performed through liquid chromatography mass spectrometry methods. Estimations are handled through the model with linear contrasts. *p* values and confidence intervals are adjusted for multiple comparisons through the Tukey‐Kramer method with *p* < 0.05.

**Results**: There were 32 patients with ICP evaluated with sebutape^®^ samples. Patients were predominantly Hispanic white (*N* = 27) with mean gestational age at initial sebum sampling at 34.3 ± 3.6 weeks and 36 ± 1 weeks at subsequent sampling. Mean total serum bile acids was 30.1 ± 20.2 at initial sampling and 26 ± 25.3 at subsequent sampling. When comparing serial samples in those with ICP, a statistically significant decrease in levels of both glycocholic (*p* < 0.001) and taurodeoxycholic acid (*p* = 0.01) were noted between initial and subsequent sampling as shown in the Table.

**Conclusion**: Using a novel method of sebum sampling of the upper back through sebutape^®^, we have demonstrated a reduction in glycocholic acid and taurodeoxycholic acid at the level of the skin in those with ICP after treatment with ursodiol. These findings may inform future diagnosis, management, and treatment strategies for ICP.

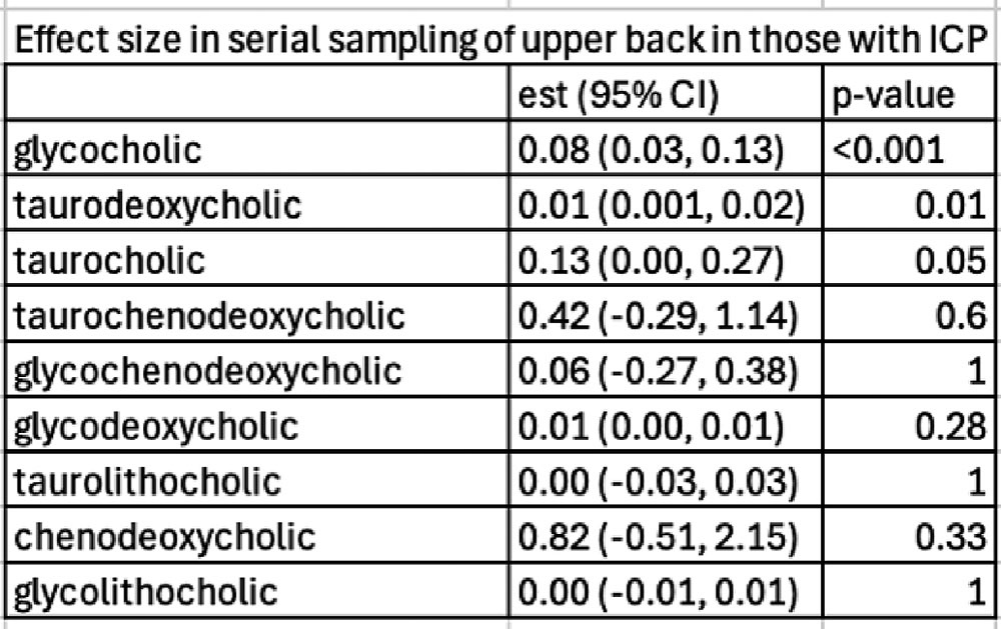

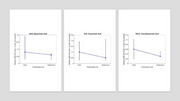

**3:30 PM – 5:00 PM**

## 427 | **Correlating** Placental **Pathology to Clinically Diagnosed Intra‐Amniotic Infection in PPROM**

Anne Stoklosa^1^; May Golenberg^2^; Amber McFerren^2^; Sarah Crimmins^3^; Ponnila S. Marinescu^2^

^1^University of Rochester Medical Center, Rochester, NY; ^2^University of Rochester Medical Center, University of Rochester Medical Center, NY; ^3^University of Rochester Medical Center–Roches, University of Rochester, NY

**Objective**: Preterm prelabor rupture of membranes (PPROM) is a risk factor for the development of intra‐amniotic infection (IAI). Placental histology can provide insight into the intra‐uterine environment that may precede the PPROM and IAI events. There is limited evidence to suggest whether rates of clinically diagnosed IAI (cIAI) are equivalent to rates of histopathologic IAI (hIAI). The objective of this study was to correlate the clinical diagnosis of IAI with placental pathology findings.

**Study Design**: Retrospective cohort study of individuals with PPROM managed at an academic medical center from 2022–2024. Available placental pathology for cases of PPROM with cIAI were compared to cases of PPROM without cIAI. Histopathologic evidence of IAI was defined by the presence of maternal or fetal acute inflammatory responses (MAIR, FAIR). Chi‐square test was used to compare categorical variables.

**Results**: Of 241 total PPROM cases, 81.7% (*n* = 197) of corresponding placentas were available for review. There was clinical and histopathologic congruence (by presence of MAIR) in 81.8% of cases, reflecting sensitivity. There were 18.2% of cases with cIAI without corresponding evidence of hIAI (*p* = 0.004). FAIR was present in 69.7% of cases of cIAI and 30.3% of cases without cIAI (*p* = 0.001). The negative predictive values (NPV) for MAIR and FAIR were 94.3% and 93.3%, respectively. FAIR was more specific than MAIR for corresponding cIAI (63.4% vs. 45.2%). The positive predictive values for both MAIR and FAIR were low (18.2% and 22.1%).

**Conclusion**: We demonstrate a high correlation between clinical and histopathologic IAI. Yet, in the absence of histologic evidence of IAI, the likelihood of a corresponding clinical diagnosis is low. This study uniquely calls attention to the interface between the clinical and in‐utero manifestations of IAI. Future research is needed to understand how placental histopathology could inform antepartum clinical management.

**3:30 PM – 5:00 PM**

## 428 | **Oral Physiologic** vs **Non‐Physiologic Treatment of Blood Pressure in Preeclampsia with Severe Features**

Annika Van Oosbree^1^; Sarayu Parise^1^; Gabriela Carrara^1^; Emalie Houk^2^; Lauren L. Yacobucci^3^; Sarah K. Shea^1^

^1^Medical University of South Carolina, Charleston, SC; ^2^The Medical University of South Carolina, The Medical University of South Carolina, SC; ^3^Medical University of South Carolina, CHARLESTON, SC

**Objective**: Evaluate the impact of physiologic blood pressure treatment in preeclampsia with severe features using pulse pressure as a surrogate for preeclampsia phenotype.

**Study Design**: This retrospective cohort study included patients with preeclampsia with severe features admitted to a tertiary academic medical center from 2014–2024, prior to 34 weeks gestation. Chronic hypertension patients were excluded. Physiologic treatment was defined by vasoconstrictive (MAP <65 mm Hg) or hyperdynamic (MAP ≥ 65 mm Hg) physiology, treated with nifedipine or labetalol, respectively. The primary outcome was latency period from admission to delivery. Secondary outcomes included gestational age at delivery, number of hospital days, delivery method, IV medications, PO medications, classical hysterotomy, and pulmonary edema. Statistical analyses were performed using Chi‐square and Mann‐Whitney tests.

**Results**: Of 156 patients, 78 received physiologic treatment and 77 received non‐physiologic treatment. No differences were found in latency period (5.51 vs. 5.04 days, *p* = 0.204), gestational age at delivery (30w1d vs. 30w2d, *p* = 0.356), hospital days (8.71 vs. 8.27, *p* = 0.381), or IV medications (3.28 vs. 2.73, *p* = 0.182). Patients receiving physiologic treatment took more daily PO medications (1.31 vs. 1.13, *p* = 0.036). No significant differences were observed in delivery method, classical hysterotomy, or pulmonary edema. Even when analyzing patients delivered only for uncontrolled blood pressure, no differences in outcomes were found. Patients with a vasoconstrictive phenotype had more fetal growth restriction (50.5% vs. 36.5%, *p* = 0.044), while those with a hyperdynamic phenotype were more likely to develop pulmonary edema (19.2% vs. 4.9%, *p* = 0.014).

**Conclusion**: Physiologic treatment did not affect latency to delivery or other clinical outcomes. Pulse pressure as a surrogate for blood pressure phenotype did not improve outcomes. The vasoconstrictive phenotype was associated with fetal growth restriction, while the hyperdynamic phenotype was linked to pulmonary edema.

**3:30 PM – 5:00 PM**

## 429 | **Hospital Cesarean Delivery Rate and Risk of Obstetric Anal Sphincter Injury Among Nulliparous Births**

Annika Willy^1^; Bharti Garg^2^; Marie J. Boller^3^; Isha Garg^3^; Aaron B. Caughey^2^

^1^Mayo Clinic, Rochester, MN; ^2^Oregon Health & Science University, Oregon Health & Science University, OR; ^3^Oregon Health and Science University, Portland, OR

**Objective**: Obstetric anal sphincter injuries (OASIS) are associated with both acute and long‐term maternal morbidity and rate of OASIS has been used as a quality measure. Risk for OASIS is increased by operative vaginal delivery, which in turn may prevent cesarean delivery (CD). Thus, as we seek to reduce rate of CD by using OASIS as a quality measure we may be inadvertently disincentivizing hospitals from lowering their CD rate.

Our objective was to evaluate the association between hospital‐level rates of CD and OASIS among nulliparous individuals.

**Study Design**: We conducted a retrospective cohort study using linked California birth certificate and hospital discharge data from 2008–2020.We included nulliparous, term, singleton, vertex‐presenting (NTSV), non‐anomalous births. We further excluded hospitals with less than 50 deliveries per year. Proportions of cesarean deliveries and OASIS were calculated at each hospital. Pearson correlation coefficient was calculated to examine the correlation between cesarean deliveries and lacerations at the hospital level. Multivariable logistic regression model was used to determine association of cesarean deliveries with lacerations at hospital level.

**Results**: The mean rate of primary NTSV cesarean was 23.9% ± 5.0%, and the mean rate of NSTV deliveries with OASIS was 9.7% ± 4.2%. The proportion of primary cesarean deliveries is overall negatively correlated with the proportion of OASIS among NSTV deliveries (correlation coefficient = −0.19, *p* < 0.001). Hospitals with higher cesarean deliveries had lower OASIS (aOR = 0.010 (95% CI: 0.008–0.012).

**Conclusion**: We found that patients delivering vaginally at hospitals with lower CD rates may have higher likelihood of OASIS, and vice versa. These findings suggest a tradeoff between reducing CD and OASIS. As we seek to identify metrics that reflect quality obstetric care, it is critical to understand the tradeoffs between such metrics and other obstetric outcomes.

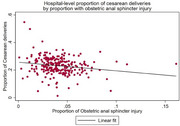

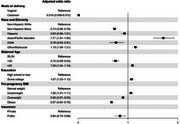

**3:30 PM – 5:00 PM**

## 430 | **Performance Of** Universal **5Ps Screening to Detect Opioid use Disorder on Labor and Delivery**

Anthony M. Kendle^1^; Chaitanya Chaphalkar^2^; Jennifer Marshall^3^; Tanner G. Wright^4^

^1^Department of Obstetrics and Gynecology, University of South Florida, Tampa, FL; ^2^Office of Research, University of South Florida, Tampa, FL; ^3^University of South Florida College of Public Health, Tampa, FL; ^4^Department of Pediatrics, The University of South Florida, Tampa, FL

**Objective**: Screening for opioid use disorder (OUD) in pregnancy should utilize a validated instrument. The 5Ps is a self‐reported instrument assessing substance use in parents, peers, partners, past, and pregnancy. We aimed to describe rates of screening as well as test performance to identify OUD.

**Study Design**: Universal 5Ps screening was implemented for all patients presenting to labor and delivery in January 2020. Data were extracted from electronic medical records from January 2020 through July 2025 for completion of 5Ps screening, responses to individual tool components, and presence OUD using a validated algorithm. Rates of 5Ps screening was stratified by year. To assess clinical utility of 5Ps, we calculated sensitivity, specificity, positive predictive value (PPV), and negative predictive value (NPV) relative to presence of OUD. Receiver operating characteristic (ROC) curve and area under the curve (AUC) was calculated using SPSS to quantify overall test performance.

**Results**: Of the 39,125 deliveries that occurred during the study period, 31,063 (79.4%) were screened with 5Ps. OUD was present in 189 (0.6%) of all screened pregnancies. A positive response to any 5Ps item had a sensitivity of 74.1% and NPV of 99.8%. A positive response to the “past” item demonstrated the highest single‐item sensitive of 70.9% and NPV of 99.8%. The 5Ps demonstrated good discriminatory performance (AUC = 0.86, 95% confidence interval: 0.82–0.89). The optimal cutoff threshold was ≥1 ‘yes’ response.

**Conclusion**: Universal use of 5Ps on labor and delivery is an effective screening tool to identify OUD. Our analysis validate the clinical relevance of a positive response to any single item on the 5Ps screening tool as an indicator warranting further assessment for OUD.

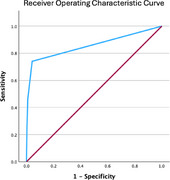

**3:30 PM – 5:00 PM**

## 431 | **Obstetrical** Outcomes **In Patients With Placenta Previa With and Without Antepartum Bleeding**

Anthony E. Melendez Torres^1^; Adwoa A. Baffoe‐Bonnie^2^; Kathleen Chang^2^; Joyce Liu^2^; Jeffrey A. Kuller^2^; Sarah K. Dotters‐Katz^3^

^1^Division of Maternal Fetal Medicine, Department of Obstetrics and Gynecology, Duke University, Durham, NC; ^2^Duke University School of Medicine, Durham, NC; ^3^Division of Maternal Fetal Medicine, Department of Obstetrics and Gynecology, Duke University, Duke University/Durham, NC

**Objective**: Placenta previa is associated with adverse perinatal outcomes. Data on delivery outcomes based on antepartum bleeding patterns are lacking. We describe differences in obstetric outcomes in placenta previa patients based on antepartum (AP) bleeding patterns.

**Study Design**: Retrospective cohort study of pregnant patients with ultrasound‐confirmed placenta previa with and without AP bleeding, delivering at a single healthcare system in 6/2013–3/2025. Patients with resolved placenta previa or low‐lying placenta excluded. Exposure of interest was AP bleeding. Primary outcome was composite of adverse delivery outcomes including cesarean hysterectomy, ICU admission, postpartum hemorrhage (PPH), and blood transfusion. Secondary outcomes included components of composite, unplanned cesarean delivery, EBL >2L, and preterm birth (PTB). Planned secondary analysis comparing above outcomes between subjects who had 1 vs >1 bleed also conducted. Bivariate statistics used to analyze data. Regression models developed to control for confounders.

**Results**: Of 247 patients included, 139 (56%) had antepartum bleeding. Patients without antepartum bleeding were more likely to be age >35 (36% vs 56.5%, *p* = 0.001) and obese (25.9% vs 41.7%, *p* = 0.009). Of those with antepartum bleeding 85 (61%) had >1 bleeding episode. The delivery composite occurred quite frequently, but did not differ based on bleeding status, even when controlling for confounders (Table 1). However, PTB and unplanned delivery were more common in patients with antepartum bleeding, even after controlling for confounders (Table 1). When comparing those with 1 vs >1 AP bleed, the frequency of adverse delivery composite did not differ, even after controlling for confounders (Table 2). The only secondary outcome that was and remained significant in this cohort was PTB (Table 2).

**Conclusion**: No differences in the adverse delivery composite were seen based on antepartum bleeding occurrence or recurrence. However, antepartum bleeding occurrence and recurrence were associated with unplanned cesarean delivery and preterm birth; aspects worth incorporating in counseling.

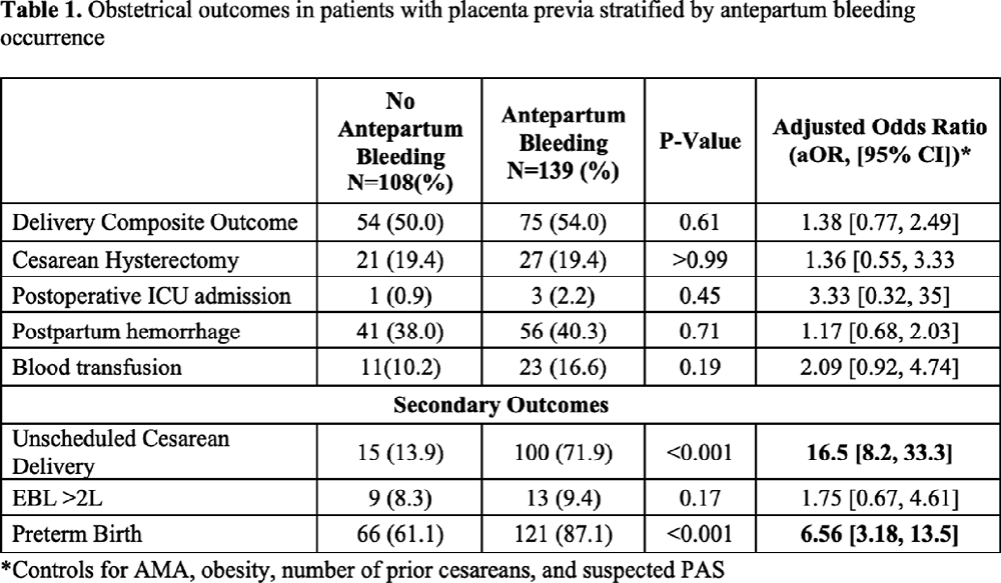

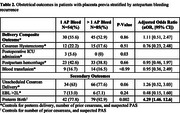

**3:30 PM – 5:00 PM**

## 432 | **Perspectives on** Implementing **Social Screenings in Maternal Substance use Disorder Clinics: A Multicenter Qualitative Study**

April Y. Lewis^1^; Ana Baumann^2^; Nandini Raghuraman^1^; Rachel Paul^3^; Abigail R. Barker^1^; Candice L. Woolfolk^4^; Melissa Mills^5^; Brittaney Vaughn^6^; Courtney Townsel^7^; Tomi Ogungbenle^1^; Abygail Martinez^4^; Jeannie C. Kelly^4^

^1^Washington University School of Medicine, St. Louis, MO; ^2^Washington University School of Medicine, Washington University in St. Louis, MO; ^3^Washington University School of Medicine, Washinton University in St. Louis School of Medicine, MO; ^4^Washington University School of Medicine, Washington University School of Medicine, MO; ^5^Washington University School of Medicine, Saint Louis, MO; ^6^Washington University School of Medicine, Barnes Jewish Hospital/Saint Louis, MO; ^7^University of Maryland School of Medicine, University of Maryland School of Medicine, MD

**Objective**: Screening for social drivers of health (SDoH) is mandated by managed care organizations, however implementation in obstetric settings, particularly for patients with substance use disorders (SUD), remains inconsistent due to a lack of standardized protocols and guidance. This study aims to explore clinician, clinical leader, and payer perspectives on effectively integrating SDoH screening and referral into a maternal SUD clinic.

**Study Design**: Qualitative focus groups were held with 13 participants involved in perinatal SUD care at two urban tertiary care centers. Participants included clinicians and clinical leaders (*n* = 8), social workers (*n* = 2), and managed care payer informants (*n* = 3). Individuals provided consent before participating. We analyzed transcripts using inductive thematic analysis. Thematic saturation guided sample sufficiency, and themes are reported with representative quotes (Table 1).

**Results**: Three primary themes emerged: **(1)** Time and Workflow Constraints: Clinicians reported that competing priorities between complex patient needs and limited appointment time challenge their ability to complete SDoH screening, highlighting the need for dedicated personnel to assist. **(2)** Role Clarity and Training Needs: Participants expressed uncertainty about who was responsible for SDoH screening. Nurses, social workers, and community health workers were identified as key team members, but their training in standardized screening varied. **(3)** Psychological Safety and Patient Trust: Participants noted that patients with SUD may fear disclosing social needs due to stigma or child protective services involvement, emphasizing the need for trauma‐informed training and trust‐based therapeutic relationships.

**Conclusion**: Despite being mandated, SDoH screening in maternal SUD care remains poorly defined in practice. Successful implementation will require clearly delineated clinical roles, adaptable workflows, and efforts to prioritize psychological safety.

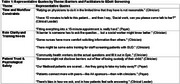

**3:30 PM – 5:00 PM**

## 433 | **Risk Factors for** Preterm **Birth Among Individuals With Spinal Cord Injury: A Retrospective Cohort Analysis**

Areeba Chaudhry^1^; Anne Berndl^2^; Sarah Kosa^3^

^1^University of Toronto, University of Toronto/Toronto, ON; ^2^Department of Obstetrics and Gynecology, Division of Maternal Fetal Medicine, University of Toronto, Toronto, ON, Toronto, ON; ^3^Women's College Hospital, Women's College Hospital/Toronto, ON

**Objective**: People with spinal cord injury (SCI) experience disproportionately high rates of preterm birth, yet contributing factors remain poorly understood. This study aimed to identify clinical, demographic, and social risk factors for preterm birth in individuals with SCI to inform tailored perinatal care strategies.

**Study Design**: We conducted a secondary analysis of the Spinal Cord Injury and Urogenital Health Project, a cross‐sectional survey of 780 individuals assigned female at birth across 33 countries. A total of 386 pregnancies among participants with SCI were analyzed. We evaluated 43 potential risk factors spanning SCI‐specific (e.g., injury level, ASIA score, etiology), biological, behavioral, and social domains. Multivariate logistic regression was used to assess associations with preterm birth (<37 weeks’ gestation), with significance set at *p* < 0.05.

**Results**: Six factors were significantly associated with increased risk of preterm birth: higher‐level SCI, greater neurologic impairment (ASIA score), gun violence as cause of injury, chronic smoking, respiratory complications due to SCI, and history of prior preterm birth. In contrast, several well‐established risk factors in the general obstetric population, including education level, anxiety/depression, and urinary tract infections, did not demonstrate statistical significance in this cohort.

**Conclusion**: This is the first study to identify risk factors for preterm birth specific to individuals with SCI. Our findings suggest that SCI‐related physiological and behavioral factors may outweigh traditional social and biological determinants in this population. These insights support the development of SCI‐informed, interdisciplinary preconception and prenatal care models.

**3:30 PM – 5:00 PM**

## 434 | **Obstetric and** Neonatal **Outcomes Following Pre‐pregnancy Exposure to GLP‐1 Receptor Agonists: A Retrospective Cohort Study**

Ariel Benyaminov^1^; Ziv Ribac^2^; Lior Hassan^2^; Gali Pariente^1^; Ran Abuhasira^2^; Tamar Eshkoli^3^

^1^Soroka University Medical Center, Beer‐Sheva, HaDarom; ^2^Soroka University Medical Center, beer‐sheva, HaDarom; ^3^Soroka Medical Center, Ben Gurion University of the Negev, beer sheba, HaDarom

**Objective**: Glucagon‐like peptide‐1 receptor agonists (GLP‐1 RAs) are increasingly used to manage type 2 diabetes mellitus and promote weight loss in women of reproductive age. While these agents are effective in improving glycemic control and reducing obesity, their safety profile during pregnancy remains uncertain. We aimed to assess whether first‐trimester exposure to GLP‐1 RAs is associated with adverse obstetric and neonatal outcomes.

**Study Design**: This retrospective cohort study included deliveries recorded in the southern district of HMO Services between 2012 and 2024. Women with documented GLP‐1 RA exposure during the first trimester were matched 1:3 to unexposed controls using nearest‐neighbor matching on maternal age, socio‐economic status, gestational age at delivery, calendar year, comorbidity score, and pre‐pregnancy BMI. Outcomes included preeclampsia, cesarean delivery, preterm birth, low birth weight, neonatal hypoglycemia, NICU admissions, and a composite maternal morbidity index.

**Results**: A total of 636 women were included (159 exposed, 477 unexposed). Baseline characteristics were similar aside from lower diabetes prevalence in the GLP‐1 group (47% vs. 100%, *p* < 0.001). No significant differences were observed in preeclampsia (23% vs. 23%), cesarean delivery (45% vs. 47%, *p* = 0.700), preterm birth, neonatal hypoglycemia, low birth weight, NICU admissions, or composite maternal outcomes.

**Conclusion**: Prepregnancy exposure to GLP‐1 RAs was not associated with increased risk of adverse obstetric or neonatal outcomes. These findings support the safety of inadvertent exposure and underscore the need for additional long‐term studies.

**3:30 PM – 5:00 PM**

## 435 | **Vasopressors for Prevention of Postspinal Hypotension During High‐Risk Cesarean Delivery: A Network Meta‐analysis**

Arya Babul^1^; Sohi Ashraf^2^; Jyoti Desai^3^; Leanne Free^4^; Momina Hussain^5^; Najib Babul^6^

^1^West Career & Technical Academy, Las Vegas, NV; ^2^MountainView Hospital, Las Vegas, NV; ^3^Department of Gynecologic Surgery & Obstetrics, Kirk Kerkorian School of Medicine at UNLV, Las Vegas, NV; ^4^Kirk Kerkorian School of Medicine at UNLV, Las Vegas, NV; ^5^Chinese Academy of Tropical Agricultural Sciences, Sanya City, Hainan; ^6^Quadra Therapeutics, Las Vegas, NV.

**Objective**: Although numerous randomized controlled trials (RCTs) have compared the safety and efficacy of norepinephrine (NE), phenylephrine (PE) and ephedrine (EP) for the prevention of postspinal hypotension (PSH) during high‐risk cesarean delivery (CD), findings are discordant. Methodologic limitations include small sample sizes, variable definitions of PSH, and heterogeneity of parturients.

**Study Design**: This systematic review and network meta‐analysis (NMA) compared RCTs of NE, PE and EP administered via bolus or infusion for the prevention of PSH in high‐risk CD (PROSPERO CRD420251050322). Endpoints included maternal hemodynamic outcomes (SBP, DBP, MAP, HR), fetal outcomes (Apgar scores, UA pH), and adverse effects (AE). Pairwise meta‐analyses for direct comparison were conducted using a random‐effects model, and for NMA, using a frequentist approach. Treatment rankings were based on P‐scores. Heterogeneity was assessed using the I^2^ statistic and Cochran's Q test. Subgroup analysis, data quality, sensitivity analysis and publication bias were examined.

**Results**: A majority of the 24 eligible RCTs (*n* = 2,843) included in the NMA showed a low risk of bias. Sensitivity analysis indicated robustness and consistency of the combined outcome. Vasopressors significantly reduced PSH incidence (OR = 1.5, 95% CI: 1.12–1.67) with low heterogeneity (I^2^ = 10.5%). Hemodynamic outcomes also favored vasopressors: SBP (OR = 0.75), DBP (OR = 0.68), and MAP (OR = 0.67). NE and PE were more effective than EP, with NE demonstrating superior cardiac stability and fewer AEs. Subgroup analysis revealed enhanced benefits in preeclamptic patients and with infusion‐based strategies. Fetal outcomes were similar across all groups.

**Conclusion**: This NMA provided strong evidence for vasopressor use in the prevention of PSH during neuraxial anesthesia in high‐risk CD. Both NE and PE were highly effective, with NE having benefits in safety and cardiac stability. EP was associated with more AEs and suboptimal maternal profiles. This evidence supports the preferential use of NE and PE over EP in such clinical settings.

**3:30 PM – 5:00 PM**

## 436 | **Assessing the** Impact **of Tirzepatide Exposure on Human Fetal Neural Stem Cells**

Asha Mada^1^; Morgan Wasickanin^2^; Robert Walton^2^; Nicholas Ieronimakis^2^; Hillary Kinsman^2^; Katie Free^2^; Jennifer Damicis^2^; Emily D. Sheikh^2^; Peter Napolitano^3^

^1^Madigan Army Medical Center, Fullerton, CA; ^2^Madigan Army Medical Center, Tacoma, WA; ^3^University of Washington, Seattle, WA

**Objective**: Glucagon‐like peptide‐1 receptor (GLP‐1R) agonists are now commonly used for weight loss and diabetes management. Semaglutide (Ozempic/Wegovy) targets GLP‐1R, whereas tirzepatide (Mounjaro/Zepbound) is a dual receptor agonist that also activates glucose‐dependent insulinotropic receptor (GIPR). The actions of these drugs on brain development remain poorly understood despite fetal exposures occurring. Previously we modeled the effects of semaglutide on neurodevelopment by exposing human fetal neural stem cells (NSCs) *in vitro* and observed no obvious changes. Due to differences between these drugs, we hypothesize that tirzepatide may alter development and examined its effects on NSC programming and inflammatory responses.

**Study Design**: Commercially available fetal NSCs were exposed to a physiological concentration of tirzepatide or the vehicle. In parallel they were given PBS as a control or Interleukin‐1 beta (IL‐1B) to stimulate inflammatory responses. Cells were harvested 24‐hours post exposure (*n* = 5–6 per condition) and examined for gene expression.

**Results**: The expression of genes related to stem cell programming (*NESTIN*, *SOX2*) and neural differentiation (*TUBBIII*, *GFAP*) was not different with tirzepatide exposure and/or IL‐1B (Figure 1A). Tirzepatide alone significantly lowered the expression of *TNF* but did not impact the upregulation of proinflammatory genes by IL‐1B (Figure 1B). *GLP‐1R* was significantly downregulated with IL‐1B irrespective of tirzepatide exposure, whereas *GIPR* did not change.

**Conclusion**: There was a significant downregulation of *TNF* with tirzepatide, not previously observed with semaglutide. No other changes in NSC programming were observed. *TNF* is essential not only for inflammation but also developmental processes. Lower levels of TNF may alter NSC proliferation and survival during normal brain formation. The downregulation of *GLP1R* with inflammatory stimulation may disrupt metabolic signaling within the developing brain. Further research is warranted to validate the clinical significance of these findings in exposed pregnancies.

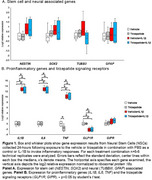

**3:30 PM – 5:00 PM**

## 437 | **Routine use of** Fetal **Brain Mri Following Fetal Intervention**

Asia Gandy^1^; Orit Glenn^1^; Vickie Feldstein^1^; Nasim C. Sobhani^2^

^1^University of California, San Francisco, San Francisco, CA; ^2^UCSF, UCSF/San Francisco, CA

**Objective**: Fetal interventions may be associated with central nervous system (CNS) injuries. CNS injuries can be identified by obstetric ultrasound (US), with fetal brain MRI as an adjunct as needed. Whether fetal brain MRI should be routinely offered following fetal interventions remains an area of clinical equipoise. This study aimed to determine the incidence of CNS anomalies in a cohort that underwent both US and MRI as part of routine postprocedural care following fetal intervention.

**Study Design**: Retrospective single‐center cohort study of all pregnancies that underwent routine postprocedural fetal brain MRI following fetal intervention, from 2014–2016 and 2021–2024. Cases from 2017–2020 were excluded due to a policy change in recommending postprocedural fetal brain MRI. The primary outcome was incidence of new CNS anomalies identified by any imaging modality. Secondary outcomes included incidence of anomalies identified by both US and MRI and by MRI alone.

**Results**: A cohort of 73 patients were included, of which 93% were monochorionic‐diamniotic twin pregnancies. The most common procedures were laser ablation for TTTS (59%) and selective reduction (36%). Median gestational age at time of procedure, post‐procedure US, and post‐procedure MRI was 19.9, 22.3, and 22.6 weeks, respectively. Of the 73 patients, 12% (9) were found to have a new CNS anomaly (Figure). Of those, 33% (3) were identified by US imaging prior to MRI, whereas the remaining 66% (6) were identified only at the time of fetal brain MRI (Table).

**Conclusion**: In this single center cohort, 12% of pregnancies were found to have new CNS anomalies following fetal intervention. Notably, 66% of these were identified only by fetal brain MRI, suggesting that US alone as a routine screening tool following fetal interventions could miss CNS anomalies. Consideration should be given to routine use of fetal brain MRI in this population. More research is needed to determine the long‐term neonatal consequences and clinical significance of the postprocedural CNS anomalies detected by MRI only.

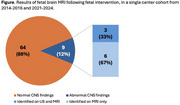

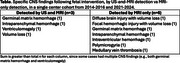

**3:30 PM – 5:00 PM**

## 438 | **Enhanced** Thematic **Analysis of the Focus Group Discussion on Gestational Diabetes and Preeclampsia Postpartum**

Asma Ahmed Fagear Mohamed^1^; Mairead Kennelly^2^; Amy O'Higgins^3^

On behalf of the UCD Center of Human Reproduction and Irish Heart Foundation

^1^UCD Center of Human Reproduction, Co Dublin, Dublin; ^2^UCD Center of Human Reproduction, Dublin, Dublin; ^3^UCD Center of Human Reproduction / Coombe Hospital, Dublin, Dublin

**Objective**: To explore the postnatal follow‐up experiences of women with gestational diabetes mellitus (GDM) and pre‐eclampsia (PET), identify barriers to care, and gather insights to inform the development of more patient‐centered and responsive postpartum follow‐up models.

**Study Design**: This qualitative study involved two online focus group discussions with 13 postpartum women previously enrolled in a structured follow‐up program for GDM or PET. Participants were invited from a larger cohort of 185 women. A semi‐structured discussion guide explored themes of awareness, emotional response, healthcare access, and service preferences. Sessions were audio‐recorded, transcribed verbatim, and analyzed using Braun and Clarke's thematic analysis framework.

**Results**: Participants reported limited knowledge of the long‐term risks associated with GDM and PET. Emotional distress, lack of childcare, scheduling conflicts, and insufficient provider communication were cited as key barriers to follow‐up. Many women prioritized newborn care over their own health. Nevertheless, participants valued health monitoring, appreciated being heard, and supported improvements such as flexible scheduling, early engagement, peer support, and integration of maternal and infant services. Suggestions included personalized health tracking and extending care beyond the traditional six‐week postpartum period

**Conclusion**: Postpartum women with GDM and PET face complex barriers to care. Addressing emotional, logistical, and informational gaps through patient‐centered, integrated services may improve engagement and long‐term outcomes. Findings inform the development of flexible follow‐up models that align with women's lived experiences and caregiving demands.

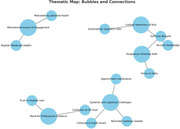

**3:30 PM – 5:00 PM**

## 439 | **Delivery Mode** and **Risk of Atopic Disease: A Population Based Cohort Study**

Leia Kennel^1^; Aude Girault^2^; Blandine De Lauzon Guillain^3^

^1^Assistance Publique–Hôpitaux de Paris, PARIS, Ile‐de‐France; ^2^Department of Obstetrics and Gynecology, Port‐Royal Maternity Hospital, AP‐HP, Cochin Hospital, FHU PREMA, Paris, France, Paris, Ile‐de‐France; ^3^INSERM‐U1153, Paris, Ile‐de‐France

**Objective**: In recent decades, both cesarean delivery rates and the prevalence of childhood atopy have increased. This parallel trend has led to hypotheses about a potential association, possibly mediated by alterations in neonatal microbiota and reduced cortisol exposure at birth following cesarean delivery.

To examine the association between mode of delivery and childhood atopic disease in a large national birth cohort.

**Study Design**: This study used data from the ELFE cohort, a longitudinal study including over 18,000 children born in France in 2011. Analyses focused on approximately 12,837 children with follow‐up data at ages 2, 5, or 10 years. Mode of delivery was obtained from medical records and analysed both as a binary variable and as a three‐category variable reflecting the urgency of the cesarean delivery. Atopic symptoms were reported by parents at each timepoint. Latent class analysis was used to identify distinct atopic profiles, and multinomial logistic regression assessed associations between delivery mode and these profiles.

**Results**: At each follow‐up (2, 5, and 10 years), four distinct atopy profiles were identified. When data from all three time points were analyzed jointly, six longitudinal profiles emerged: asymptomatic, food allergic, exclusive skin involvement, early respiratory atopy, persistent respiratory atopy, and multi‐atopic. At age 2, compared to vaginal delivery, cesarean delivery was associated with an increased risk of respiratory atopy (OR 1.21 [1.04–1.41]), particularly for planned cesarean (OR 1.29 [1.02–1.62]). At age 10, planned cesarean remained associated with respiratory atopy (OR 1.54 [1.01–2.36]). Across all timepoints, cesarean delivery was associated with a higher risk of persistent respiratory atopy (OR 1.37 [1.07–1.74]), especially for emergency cesarean (OR 1.45 [1.09–1.92]).

**Conclusion**: Associations between cesarean birth and long‐term respiratory atopy underscore the importance of considering potential immune consequences when planning delivery mode, and support efforts to minimize unnecessary cesareans.

**3:30 PM – 5:00 PM**

## 440 | **Discharge on** Labetalol **Lowers 6‐Week Postpartum Diastolic Blood Pressure Compared to Nifedipine**

Audra C. Fain^1^; Jenny Y. Mei^2^; Maral Demirjian^3^; Lorna Kwan^3^; Rashmi Rao^4^; Thalia Mok^5^

^1^Department of Obstetrics and Gynecology, David Geffen School of Medicine at University of California, Los Angeles (UCLA), Los Angeles, CA; ^2^Stanford University, Stanford University, CA; ^3^Department of Urology, David Geffen School of Medicine at University of California, Los Angeles (UCLA), Los Angeles, CA; ^4^Division of Maternal Fetal Medicine, Department of Obstetrics and Gynecology, David Geffen School of Medicine at University of California, Los Angeles (UCLA), Los Angeles, CA; ^5^David Geffen School of Medicine at University of California, Los Angeles (UCLA), Los Angeles, CA

**Objective**: Improved blood pressure (BP) control following hypertensive disorders of pregnancy (HDP) may reduce long‐term cardiovascular risk, yet the optimal antihypertensive (anti‐HTN) agent remains unknown. We compared 6‐week postpartum (PP) BP measurements and outcomes among patients with HDP or chronic hypertension (CHTN) discharged on nifedipine or labetalol.

**Study Design**: Retrospective cohort study of pregnancies with HDP or CHTN that delivered at a quaternary care center between April 2022 to April 2025 and were discharged on either nifedipine or labetalol. Those discharged on two or more antihypertensive medications were excluded. Primary outcome was mean 6‐week PP visit systolic and diastolic BP. Secondary outcomes included 6‐week PP BP ascertainment, emergency department (ED) visits, hospital readmission, and hypotension. Primary and secondary outcomes were compared by anti‐HTN agent with Chi‐Square or Fisher's exact and Wilcoxon rank‐sum tests.

**Results**: 785 pregnancies with HDP or CHTN delivered and were discharged home on a single antihypertensive agent: 650 on nifedipine, 135 on labetalol. The labetalol cohort was more likely to be obese, multiparous, and have CHTN (Table 1, *p* < 0.05). Mean 6‐week systolic BP did not differ by anti‐HTN agent (127.60 ± 6.41 vs. 126.79 ± 7.38, *p* = 0.15), but labetalol was associated with significantly lower 6‐week diastolic BP (79.94 ± 5.25 vs. 77.10 ± 5.98, *p* < 0.001). At 6 weeks PP, rates of documented BP measurement (75.1% vs 67.4%, *p* = 0.07) and ED presentations (1.2% vs 2.2%, *p* = 0.41) and hospital readmissions (1.7% vs 4.4%, *p* = 0.10) for elevated BP did not significantly differ between groups.

**Conclusion**: Patients discharged on labetalol for HDP achieved lower diastolic BP at the 6‐week PP visit than those on nifedipine, despite having a higher cardiometabolic risk profile. Our results suggest that labetalol may be the preferred anti‐HTN agent in certain populations. Further studies are needed to guide individualized anti‐HTN selection and evaluate the effect of improved diastolic BP control on long‐term cardiovascular risks.

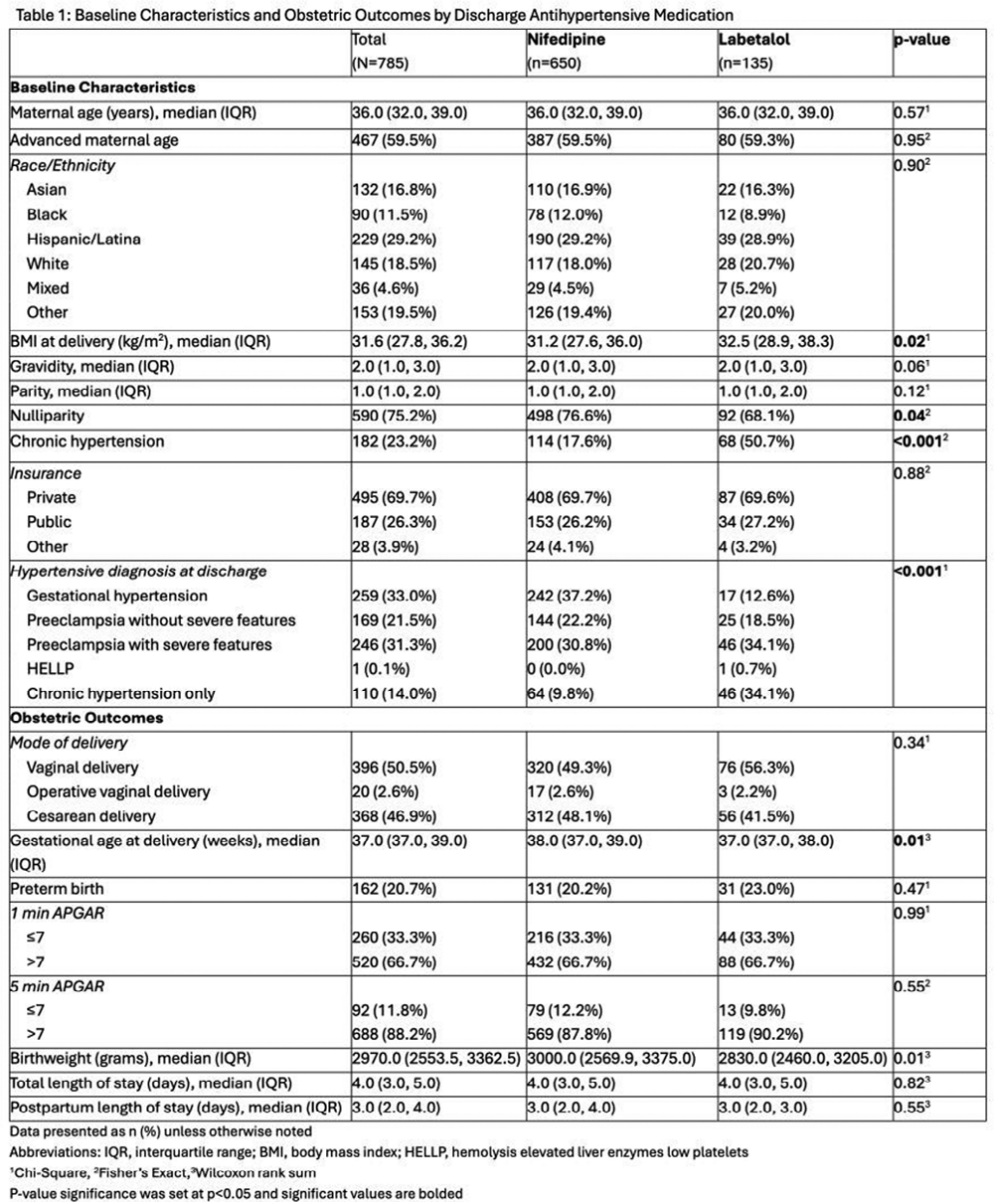

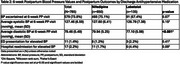

**3:30 PM – 5:00 PM**

## 441 | **Subsequent** Pregnancy **Outcomes After Nulliparous Second‐Stage Cesarean Versus Operative Vaginal Delivery**

Avery Cox^1^; Sarah Martinez^2^; Bridget M. Donovan^3^; Michelle J. Wang^4^; Anna M. Modest^4^; Cassandra R. Duffy^4^

^1^Beth Israel Deaconess Medical Center, Beth Israel Deaconess Medical Center, MA; ^2^Beth Israel Deaconess Medical Center, Beth Israel deaconess Medical Center, MA; ^3^University of Virginia, University of Virginia, VA; ^4^Beth Israel Deaconess Medical Center, Boston, MA

**Objective**: Second‐stage cesarean (CS) is associated with an increased risk of future preterm birth. We compared pregnancy and delivery outcomes in subsequent pregnancies following CS for second‐stage arrest to outcomes in those with operative vaginal deliveries (OVD) in order to control for the second stage of labor.

**Study Design**: This was a retrospective cohort study of nulliparous patients with a singleton term birth from 2017 to 2020 and subsequent pregnancy at our institution. Patients with second‐stage CS and OVD were compared. Data was abstracted from the electronic medical record. Primary outcomes were cervical insufficiency, preterm birth, and gestational age (GA) at delivery in subsequent pregnancy. Secondary outcomes were mode of delivery, birth characteristics, and neonatal outcomes.

**Results**: We identified 180 second‐stage CS and 214 OVD. There was no difference between groups in diagnosis of short cervix, initiation of vaginal progesterone, cerclage placement, or rates of preterm birth in subsequent pregnancies (Table 1). The majority of patients in the OVD group had a subsequent vaginal birth (VB) (87.9%) compared to 22.2% in the second‐stage CS group (*p* < 0.001). Among those with subsequent VB, the second‐stage CS group were more likely to present in spontaneous labor (91.8% vs. 59.5%, *p* = 0.002). The median length of second stage in subsequent VB was longer for prior second‐stage CS vs. OVD (66 vs. 23 min, *p* < 0.0001). Among those with subsequent CS, the OVD group was more likely to have an intrapartum or urgent indication for CS. In the second‐stage CS group, 65% elected or were counseled to have a scheduled subsequent CS. However, only 26% of those attempting secondary VB failed. Neonatal outcomes between groups were overall similar.

**Conclusion**: There were no inter‐group differences in rates of preterm birth or cervical insufficiency. Interestingly, of those with a subsequent VB, those in the second‐stage CS group were more likely to present in spontaneous labor.

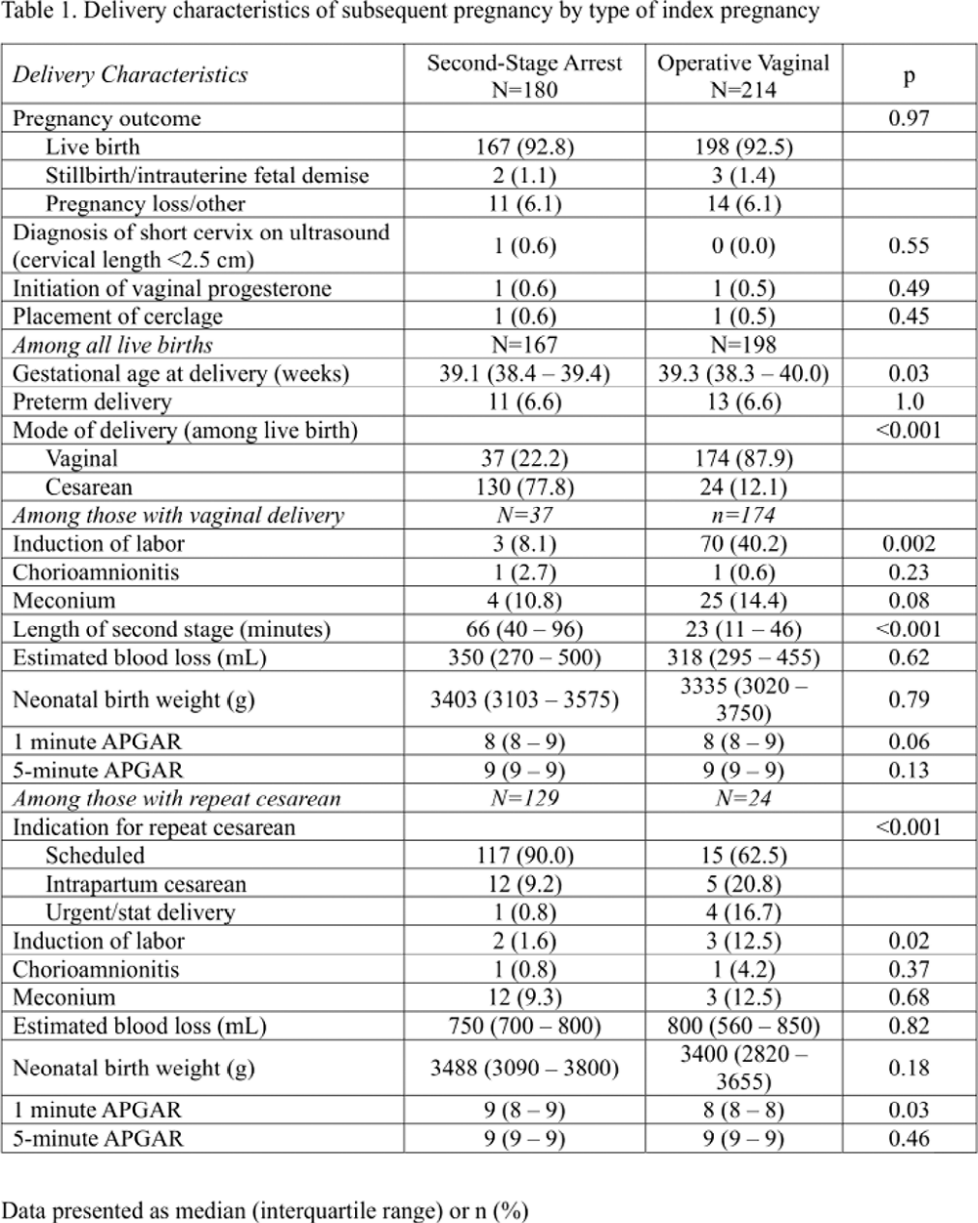

**3:30 PM – 5:00 PM**

## 442 | **Late Preterm** Labor **Induction Outcomes in Nulliparas with Hypertensive Disorders of Pregnancy**

Avihu Krieger^1^; Michal Axelrod^2^; Shiran Bookstein^3^; Michal Fishel Bartal^4^

^1^The Department of Obstetrics and Gynecology, The Chaim Sheba Medical Center, Ramat‐Gan, Israel, Ramat Gan, Tel Aviv; ^2^Sheba Medical Center, Sheba Medical Center, HaMerkaz; ^3^Sheba Medical Center, Ramat Gan, Israel, Ramat Gan, HaMerkaz; ^4^Division of Maternal‐Fetal Medicine, Department of Obstetrics, Gynecology and Reproductive Sciences, Sheba Medical Center, Tel Aviv University, Israel, Ramat Gan, HaMerkaz

**Objective**: The data regarding delivery outcomes among nulliparous individuals with hypertensive disorders of pregnancy (HDP) during the late preterm period is limited. We aimed to evaluate the rate of successful induction of labor (IOL) and risk factors for unplanned cesarean delivery (CD), among nulliparous individuals with HDP undergoing IOL in the late preterm period (34 and 37 weeks).

**Study Design**: This retrospective cohort study included nulliparous pregnancies individuals with HDP who underwent IOL in the late preterm period. Those with a planned caesarean delivery (CD) were excluded. The primary outcome was mode of delivery. Logistic regression was used to identify predictors of vaginal delivery. Adjusted odds ratios (aOR) and 95% confidence intervals (CI) were calculated.

**Results**: Of 6670 individuals diagnosed with HDP during the study period, 537 (8.1%) underwent induction between 34–37 weeks. Among them, 242 (45.1%) nulliparous individuals met inclusion criteria. Of these, 155 (64.0%) had a vaginal delivery, while 87 (36.0%) underwent an unplanned CD.

Vaginal delivery was less likely in patients with severe HDP (43% vs. 70%, *p* < 0.001), older age (29.6 vs. 32.4 years, *p* < 0.001), and higher BMI (28.9 vs. 29.7, *p* = 0.013). Maternal and neonatal outcomes were similar between the groups (Table 1). In multivariable analysis, severe HDP (aOR 3.22, 95% CI 1.72–6.01), maternal age (aOR 1.09 per year, 95% CI 1.03–1.16), and Body Mass Index (BMI) (aOR 1.09 per unit, 95% CI 1.03–1.15) independently predicted unplanned CD (Table 2).

**Conclusion**: Approximately one in three nulliparous individuals with HDP requiring induction in the late preterm period will undergo an unplanned CD. The risk is higher among those with severe disease, advanced maternal age, and elevated BMI. These findings may assist with patient counseling and guide clinical decision‐making regarding induction in this population.

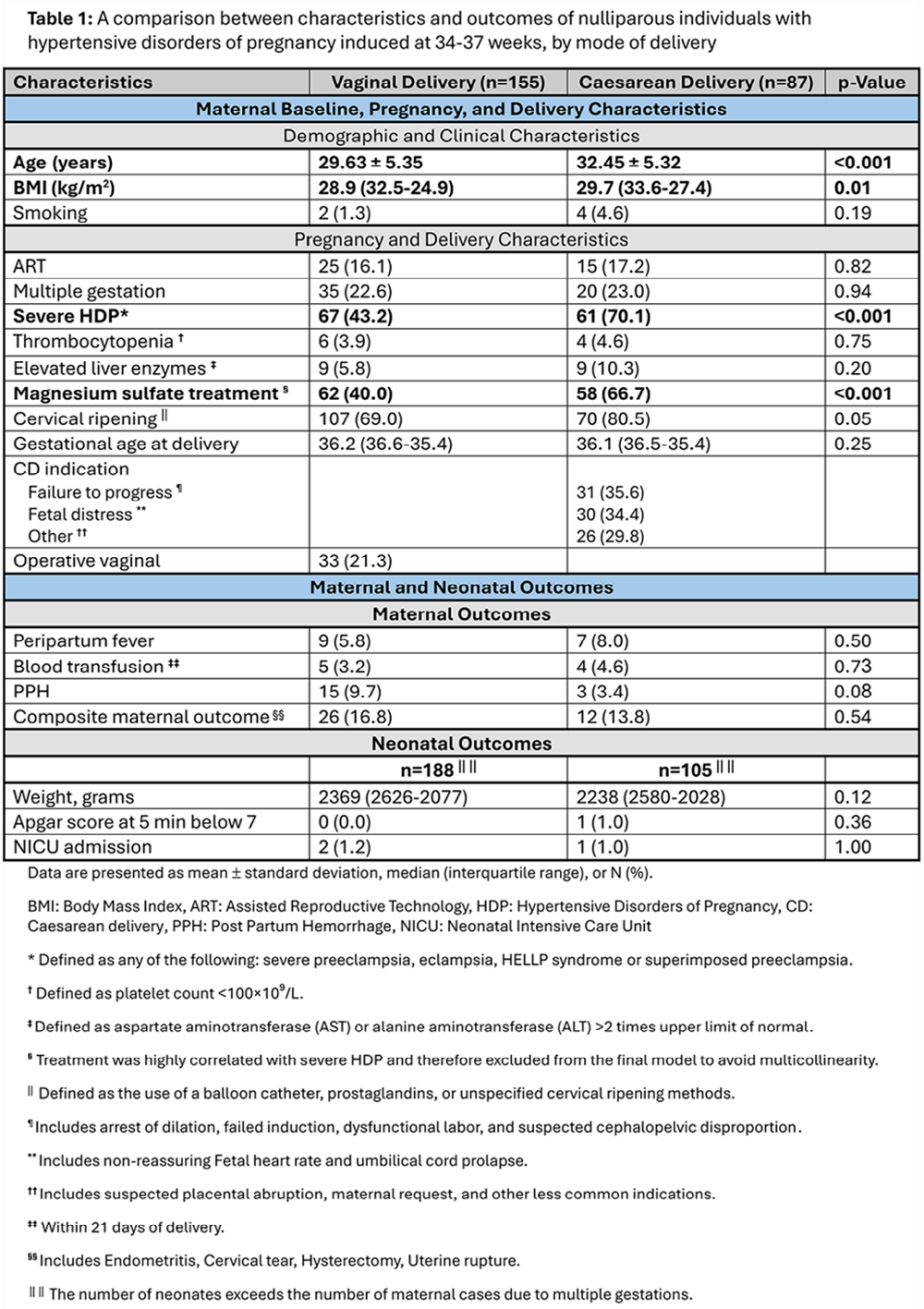

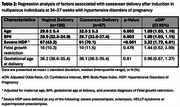

**3:30 PM – 5:00 PM**

## 443 | **Agentic AI in** Gestational **Diabetes Care: An Analysis of Patient Communication and Concerns**

Avish Arora^1^; Pe'er Dar^2^; Georgios Doulaveris^2^

^1^Montefiore/Albert Einstein College of Med., New York, NY; ^2^Montefiore Medical Center/Albert Einstein College of Medicine, Montefiore Medical Center/Albert Einstein College of Medicine, NY

**Objective**: To categorize patient‐initiated messages to a virtual GDM assistant, identifying key concerns and communication patterns.

**Study Design**: This prospective observational study (June 1–July 30, 2025) included ten newly diagnosed diet‐controlled GDM patients. We developed a patient‐facing, agentic AI tool for GDM that received FDA approval as an investigational device to support self‐management. The WhatsApp‐based assistant offered real‐time support, logged glucose readings, provided immediate feedback, answered dietary and general health questions, and escalated concerning symptoms to clinicians. Patient messages were classified using a predefined taxonomy: glucose readings, dietary comments and questions, general health questions, symptom reports, greetings, acknowledgments, logistical inquiries, and “other.”

**Results**: A total of 710 messages were analyzed. Most (50.7%) were glucose readings. Dietary content (17.1%) highlighted nutrition as a key educational need. General health questions comprised 3.2% of messages (e.g., “Does stress raise blood sugar?”). Symptom reports were infrequent (1.5%) but clinically important; two triggered clinician callbacks within 15 minutes. Social interactions (greetings, acknowledgments) totaled 18.7%. Logistical messages accounted for 1.0%, and “other” represented 7.7%. No manual overrides were required for safety alerts.

**Conclusion**: Patients primarily used the agentic AI‐powered assistant for glucose reporting and nutritional queries. This demonstrates an educational opportunity in dietary management and confirms the potential of AI to continuously support and adapt patient education strategies in real‐time GDM management. A randomized controlled trial comparing traditional nutritional counseling with agentic AI–based diabetes management is underway.

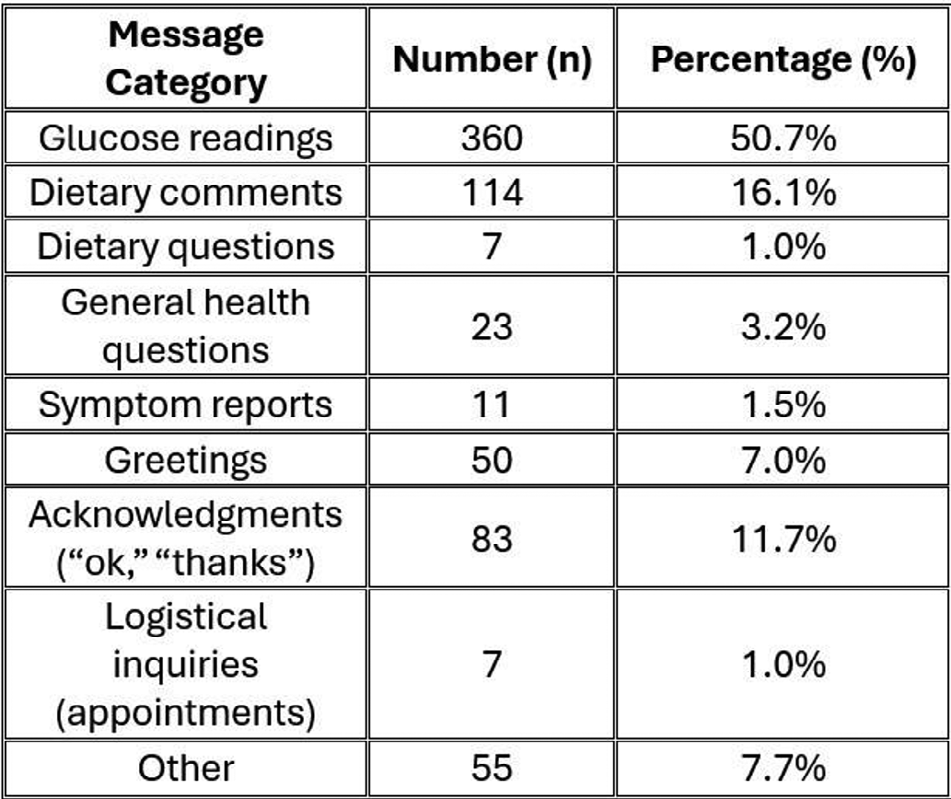

**3:30 PM – 5:00 PM**

## 444 | **Combined** Hormonal **Contraceptives During Lactation: A Network Meta‐Analysis of Breastfeeding, Maternal, and Infant Outcomes**

Ayala Hirsch^1^; Stav Yanko^2^; Shira Shalit^3^; Ilona Lozovsky^2^; Bruria Hirsh Raccah^2^

^1^Shaare Zedek medical hospital, Jerusalem, Yerushalayim; ^2^Shaare Zedek medicalhospital, Jerusalem, Yerushalayim; ^3^Hebrew University of Jerusalem, Jerusalem, Yerushalayim

**Objective**: To assess the safety and efficacy of various contraceptive methods used during breastfeeding, with a focus on estrogen‐based options, using a network meta‐analysis approach.

**Study Design**: Eligible studies included randomized controlled trials and observational studies (cohort and case‐control) that compared different contraceptive methods in postpartum breastfeeding women. Contraceptives were classified into three categories: (1). Progestin‐only (2). Combined estrogen and progestin (3). Non‐hormonal methods (e.g., copper IUD, sterilization, condoms, spermicides. Primary maternal outcomes included breast milk volume, breastfeeding discontinuation, and unplanned pregnancies. Infant outcomes included weight and height. A network meta‐analysis was performed to compare interventions.

**Results**: Of 5728 screened records, 38 studies were included (5 RCTs, 33 observational), comprising a total of 13,122 women. No statistically significant reduction in breast milk volume was observed when comparing combined oral contraceptives (COC) to progestin‐only ([SMD] 13.41ml, 95% CI −29.5 to 56.33; *p* = 0.54) or to non‐hormonal methods ([SMD] 24.5ml, 95% CI −18.95 to 67.95; *p* = 0.27). Similarly, no significant difference was found in breastfeeding discontinuation rates: COC vs. progestin‐only: OR 1.37 (95% CI 0.67–2.81; *p* = 0.4) and COC vs. non‐hormonal: OR 1.13 (95% CI 0.57–2.24; *p* = 0.7). Among 8712 women, 149 unplanned pregnancies were reported. Mechanical barrier methods were associated with a higher pregnancy risk compared to progestin‐only methods (OR 1.92, 95% CI 1.06–3.48; *p* = 0.0308). Infant growth parameters (weight and height) showed no significant differences across groups.

**Conclusion**: Estrogen‐containing contraceptives do not appear to adversely affect milk volume, breastfeeding continuation, or infant growth compared to other methods. These findings support the consideration of a broader range of contraceptive options during lactation, based on individual preference and clinical context. Nonetheless, larger and well‐designed studies are needed to confirm these results and inform practice guidelines.

**3:30 PM – 5:00 PM**

## 445 | **Free thyroxine (**FT4**) assay in pregnancy routinely underestimates T4 compared to the adjusted Total T4**

Bethany T. Waites^1^; Matthew A. Bolt^1^; Virginia Lijewski^1^; Mary D. Sammel^2^; Linda A. Barbour^1^

^1^University of Colorado, Aurora, CO; ^2^Colorado School of Public Health, Aurora, CO

**Objective**: Accurate thyroid function testing in pregnancy is critical to avoid under‐ and over‐treatment. Free thyroxine (FT4) by immunoassay (FT4IA) is commonly used but limited in pregnancy due to marked binding protein changes, a FT4 fraction of <0.05%, and lack of trimester‐specific ranges. The ATA endorses total thyroxine (TT4) adjusted by 1.5x after 16 wks, but many clinicians are unaware. We hypothesized FT4IA and pregnancy‐adjusted TT4 disagree at a rate of 20% after 16 wks. We also compared TT4 to FT4 by equilibrium dialysis, tandem mass spectrometry (FT4ED), the gold standard with trimester‐specific ranges.

**Study Design**: Retrospective study of pregnant individuals 18–45 yrs between 1/2021–6/2025, >16 wks gestation, with paired FT4IA vs TT4 (Siemens ADVIA Centaur XP) samples, or TT4 vs FT4ED (ARUP) samples, within 7 days. Lab results were assigned to quintile groups (Below Normal range (NR); Lower NR; Mid NR; Upper NM; Above NR) as these ranges are targeted clinically. A binomial test assessed whether 20% of FT4IA and TT4 quintiles disagreed.

**Results**:

***FT4IA vs TT4***

FT4IA and TT4 were measured in 78 pregnancies (mean age 32 yrs; Gravity/Parity (G/P) 2.6/1.1). A binomial test of proportions found that the disagreement rate was 89.7% (95% CI: [80.8%‐95.5%], *p* < 0.001). In of all disagreements (∼90%), the FT4 quintile was lower than TT4 (Figure 1).

***TT4 vs FT4ED***

TT4 and FT4ED were measured in 14 pregnancies (mean age 33 yrs; G/P 2.9/1.4) and were discordant in 28.6% (95% CI [8.4%‐58.1%], *p* = 0.50). The TT4 quintile was higher than FT4ED in 3/4 of discordant cases (Figure 2).

**Conclusion**: We found only a ∼10% agreement when FT4IA and adjusted TT4 quintiles were compared, with FT4IA consistently lower than TT4. In contrast, TT4 agreed with FT4ED in ∼71% of quintiles. Clinical implications of using FT4IA include undertreatment with anti‐thyroid meds for hyperthyroidism and overtreatment with LT4 for hypothyroidism. Our data supports an urgent need for trimester‐specific FT4IA ranges and/or improved clinician education on use of TT4 or FT4ED for appropriate thyroid disease management in pregnancy.

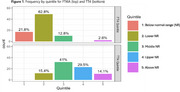

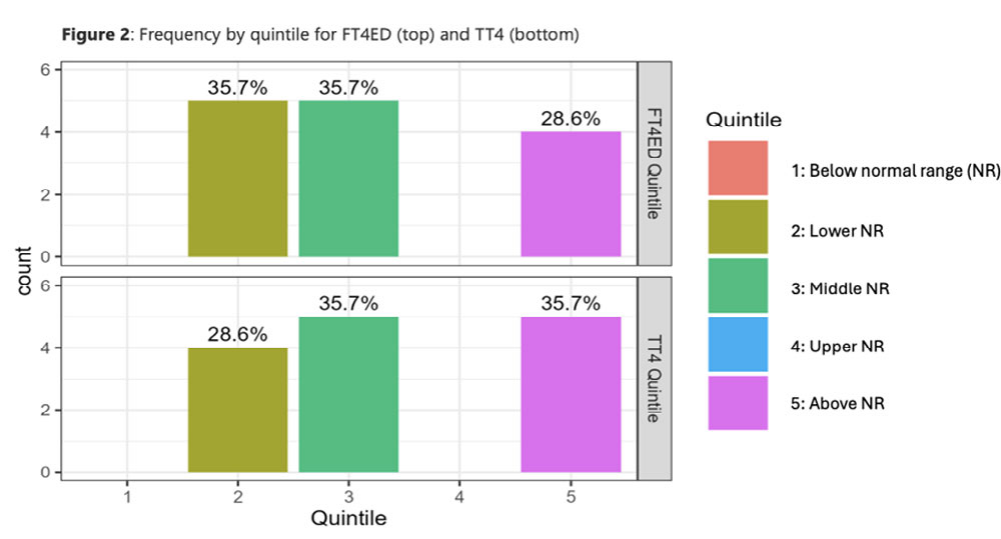

**3:30 PM – 5:00 PM**

## 446 | **Comparing Standard Clinical Evaluation & Immunoassays in Diagnosing of Rupture of Membranes: A Systematic Review**

Bhaskari Burra^1^; Baillie Bronner^2^; Katie Aman^2^; Kathryn Davidson^2^

^1^Mountain Area Health Education Center, Mountain Area Health Education Center, NC; ^2^Mountain Area Health Education Center, Mountain Area Health Education Center, NC, NC

**Objective**: Preterm prelabor rupture of membranes and term prelabor rupture of membranes affect 3% and 8% of births in the United States. Accurate diagnosis prevents adverse outcomes or harmful interventions. Traditionally, rupture of membranes (ROM) is diagnosed by a speculum exam to visualize pooling, nitrazine testing, and/or ferning on microscopy. However, this can be insufficient as individual components are affected by blood, urine, semen, and lubricants. Rapid immunoassays were developed as a more accurate method of diagnosing ROM by detecting biochemical markers highly concentrated in amniotic fluid [placental alpha‐microglobulin‐1(PAMG‐1), insulin like growth factor binding protein‐1(ILGFBP‐1), and alpha‐fetoprotein(AFP)]. Our objective was to consolidate available data comparing standard clinical evaluation (SCE) and immunoassays in diagnosing ROM.

**Study Design**: This systematic review adhered to PRISMA (Preferred Reporting Items for Systematic Reviews and Meta‐analyses) guidelines. Inclusion criteria were prospective/observational cohort studies comparing SCE and immunoassays against a standard reference measure with a gestational age of >20 weeks. Weighed means of the sensitivity, specificity, positive and negative predictive values of each method were calculated.

**Results**: Of 31 abstracts screened, 15 met inclusion criteria. 11 studies compared SCE to PAMG‐1, 2 compared SCE to ILGFBP‐1, and 4 compared SCE with AFP/ILGFBP‐1 duo‐assay. AFP/ILGFBP‐1 had the highest weighted sensitivity and PAMG‐1 had the highest weighted specificity. SCE had the highest weighted PPV but the lowest weighted NPV. All immunoassays were more sensitive and more specific than SCE.

**Conclusion**: Our systematic review demonstrates that immunoassays are highly sensitive, supporting their utility as effective screening tools. While SCE yielded excellent PPV, its NPV was limited, highlighting the need for a more reliable method to rule out ROM. These findings suggest a rationale for employing both SCE and immunoassays in tandem in diagnosing ROM. Future investigations should assess the diagnostic potential of this combined approach.

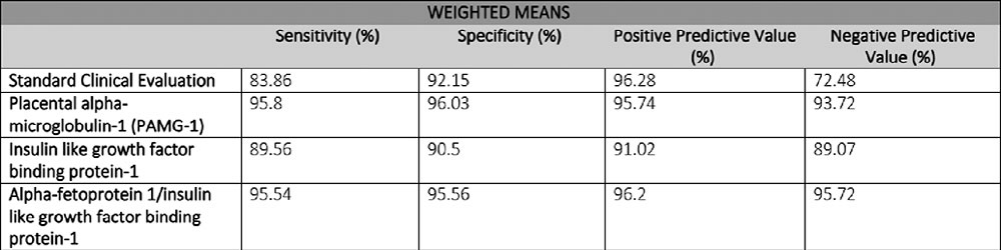

**3:30 PM – 5:00 PM**

## 447 | **Delivery Morbidity and Discrimination Impact Postpartum Follow Up**

Bridget C. Huysman^1^; Adia Woodson^2^; Mia Malcolm^3^; Harry A. Obeng^4^; Paige Wilder^5^; Maya Bussey^3^; Lori Atwood^1^; April Y. Lewis^3^; Monique Gill^3^; Rachel Witt^6^; Eve Colson^3^; Ebony Carter^7^; Candice L. Woolfolk^1^

^1^Washington University School of Medicine, Washington University School of Medicine, MO; ^2^Washington University in St. Louis, Saint Louis, MO; ^3^Washington University School of Medicine, St. Louis, MO; ^4^School of Public Health, Washington University in St. Louis, Washington University in St. Louis, MO; ^5^Washington University School of Medicine, Saint Louis, MO; ^6^Children's Minnesota, Minneapolis, MN; ^7^University of North Carolina, University of North Carolina, NC

**Objective**: Experiencing racism and discrimination during delivery may impact postpartum follow‐up care. This study aims to assess the influence of delivery morbidity and discrimination on postpartum follow up.

**Study Design**: We conducted a prospective survey of a representative convenience sample of patients admitted for delivery at a tertiary care center from 03/2024 to 06/2025. The Discrimination in Medical Settings Scale (DMSS) was administered prior to discharge. Postpartum readmissions, triage, and office visits were compared between people who experienced delivery morbidity and those who did not (Table). We assessed for interaction by reports of any discrimination. Survey responses were compared between groups using Chi‐squared or Mann Whitney U tests as appropriate and multivariable logistic regression was used to estimate adjusted odds ratios (aOR) and 95% confidence intervals (CI).

**Results**: Prior to discharge, 262 birthing people were approached and 83 declined, most of whom declined all research opportunities on admission. Over 50% experienced delivery morbidity. Of the cohort, 2.7% were readmitted, 13.7% visited triage, and 38.2% attended an office visit postpartum. There was a statistically significant difference in those experiencing delivery morbidity and triage visits postpartum (*p* < 0.01, aOR 4.48, 95% CI [1.94, 10.24]). Delivery morbidity increased the odds of a postpartum triage visit (aOR 3.68, 95% CI [1.38, 9.79]), and this risk was further modified by discrimination (*p* interaction = 0.02), but not for postpartum readmission or office visit. Birthing people who did not experience delivery morbidity were more likely to present to their postpartum office visit (aOR 0.59, 95% CI [0.30, 0.99]) (Table). There was no difference in readmissions between the groups (Table).

**Conclusion**: Birthing people who experience delivery morbidity and discrimination are more likely to present to triage, but not their postpartum office visit. Strategies to reduce discrimination could improve continuity of postpartum care for at risk birthing people.

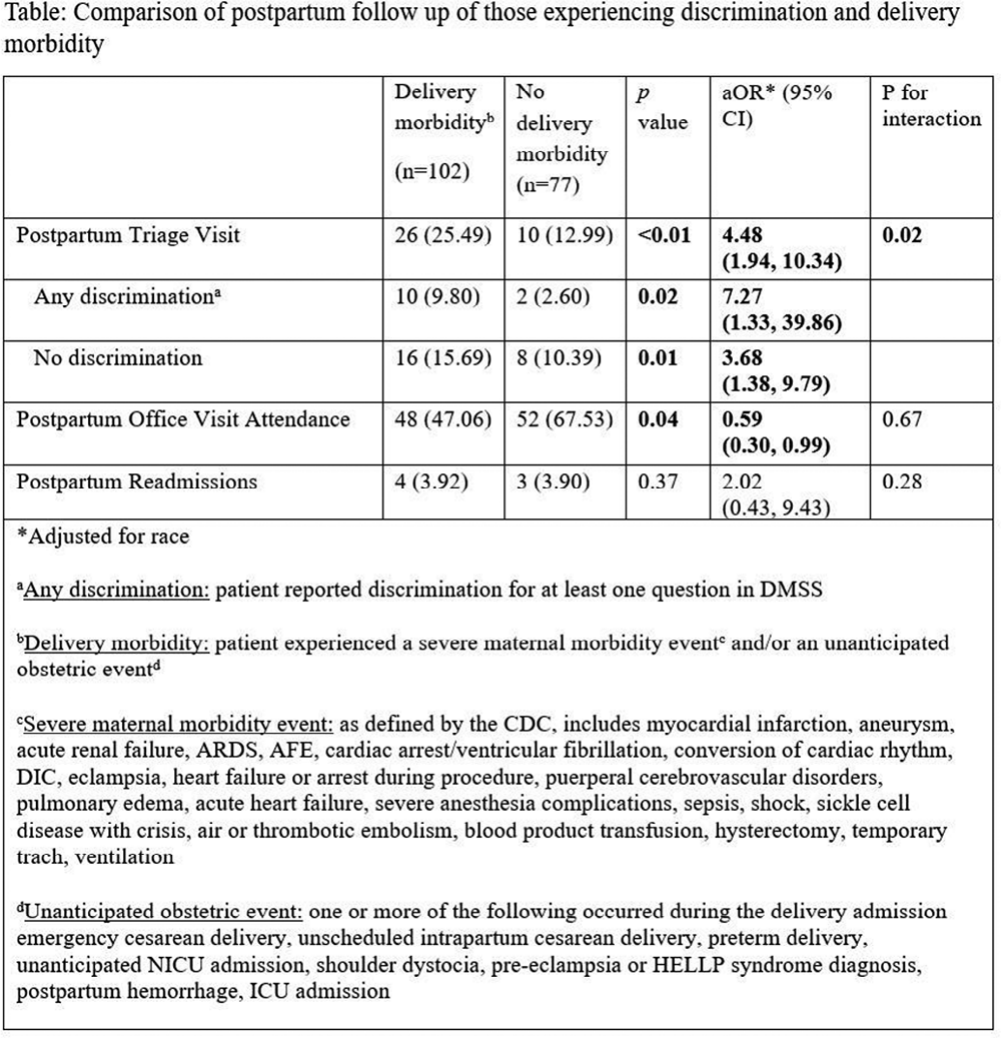

**3:30 PM – 5:00 PM**

## 448 | **Delivery** Outcomes **Among Patients with Marfan Syndrome in the United States: 2016–2022**

Brittany Arditi^1^; Caitlin D. Baptiste^2^; Marie‐Julie Trahan^1^; Timothy Wen^3^

^1^Columbia University Irving Medical Center, New York, NY; ^2^Division of Maternal‐Fetal Medicine, Department of Obstetrics and Gynecology, Columbia University Irving Medical Center, New York, NY; ^3^UCSD, Irvine, CA

**Objective**: Patients with Marfan syndrome (MFS) are known to have increased cardiovascular risk in pregnancy. Our objective was to characterize contemporary maternal and obstetric outcomes in pregnancies complicated by MFS using a nationally representative dataset.

**Study Design**: This cross‐sectional study used the U.S. National Inpatient Sample to identify delivery hospitalizations among patients with MFS from 2016 to 2022. Adjusted logistic regression models were used to compare outcomes between patients with and without MFS, accounting for demographic, clinical, and hospital‐level characteristics. Associations were reported as adjusted odds ratios (aORs) with 95% confidence intervals (CIs).

**Results**: Among more than 25 million delivery hospitalizations, 1435 involved patients with MFS. The prevalence of MFS affected deliveries remained stable over the study period [Figure 1]. Patients with MFS were at significantly increased risk of severe maternal morbidity (aOR 5.79, 95% CI 3.29–10.19) and a composite of cardiovascular complications—including aortic dissection or rupture, cardiac arrest, arrhythmia, and heart failure (aOR 7.76, 95% CI 3.61–16.7). MFS was also associated with higher odds of cesarean delivery (aOR 2.19, 95% CI 1.72–2.77) and operative vaginal delivery (aOR 7.89, 95% CI 5.63–11.07). MFS was not associated with hypertensive disorders of pregnancy (aOR 0.69, 95% CI 0.47–1.01). From a fetal perspective, patients with MFS were more likely to experience preterm delivery at both <37 weeks (aOR 2.19, 95% CI 1.62–2.96) and <34 weeks (aOR 1.74, 95% CI 1.01–2.97). No significant associations were found with fetal growth restriction or intrauterine fetal demise [Table 1].

**Conclusion**: The rate of deliveries affected by MFS did not change significantly over time. Pregnancies complicated by MFS were associated with markedly increased risks of maternal complications, as well as preterm birth. Further research is needed to identify modifiable factors that contribute to these elevated risks.

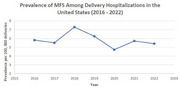

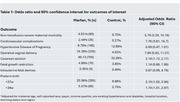

**3:30 PM – 5:00 PM**

## 449 | **Severe Maternal Morbidity and Mortality Among Patients With Hypertensive Disorders of Pregnancy**

Brittany Hood; Elizabeth L. Mirsky; Emily A. DeFranco

University of Kentucky, University of Kentucky/Lexington, KY

**Objective**: Hypertensive disorders of pregnancy (HDP) are a leading cause of severe maternal morbidity (SMM) and mortality in the US. Rates are rising and are higher than other industrialized nations. Although the association between HDP and SMM is established, there is a paucity of data quantifying risk by severity of the subtype of HDP, which we aimed to assess.

**Study Design**: Population‐based retrospective cohort study using US delivery data from the National Inpatient Sample database from 2016–2022. The 10th edition International Classification of Diagnoses codes were used to identify delivery admissions, diagnosis of HDP and indicators of SMM, defined by Centers for Disease Control and Prevention criteria. Generalized linear models estimated crude and adjusted relative risks (aRRs) and 95% confidence intervals for SMM, mortality and individual SMM contributors among HDP subtypes using Stata.

**Results**: We identified 4,992,996 hospital admissions associated with a birth, of which, 760,683 (15.2%) had HDP. All subgroups were associated with a modest increase in SMM, however there was a significant risk increase among patients with preeclampsia (PE) without severe features (aRR [95% CI] 2.35 [2.28–2.42]), PE with severe features (4.71 [4.62–4.81]), chronic hypertension (CHTN) with superimposed preeclampsia (SIP) (3.69 [3.57–3.82]), and HELLP (12.41 [11.98–12.86]) even after adjustment for maternal age, race, pregestational diabetes, and low income. Maternal mortality risk was highest in patients with CHTN (2.57 [1.66–3.99]), CHTN with SIP (3.54 [2.06–6.07]), and HELLP (19.77 [12.28–31.83]) when adjusted for maternal race.

**Conclusion**: Preeclampsia is associated with a 2.3‐ to 4.7‐fold increase in SMM, while HELLP confers a 12.4‐fold increase. Maternal mortality risk is elevated with CHTN and rises nearly 20‐fold with HELLP. Sociodemographic disparities and pre‐existing medical conditions disproportionately increased the risk of SMM and mortality in patients with HDP. These findings highlight the need for targeted clinical interventions and policy efforts to reduce health inequities and improve maternal outcomes.

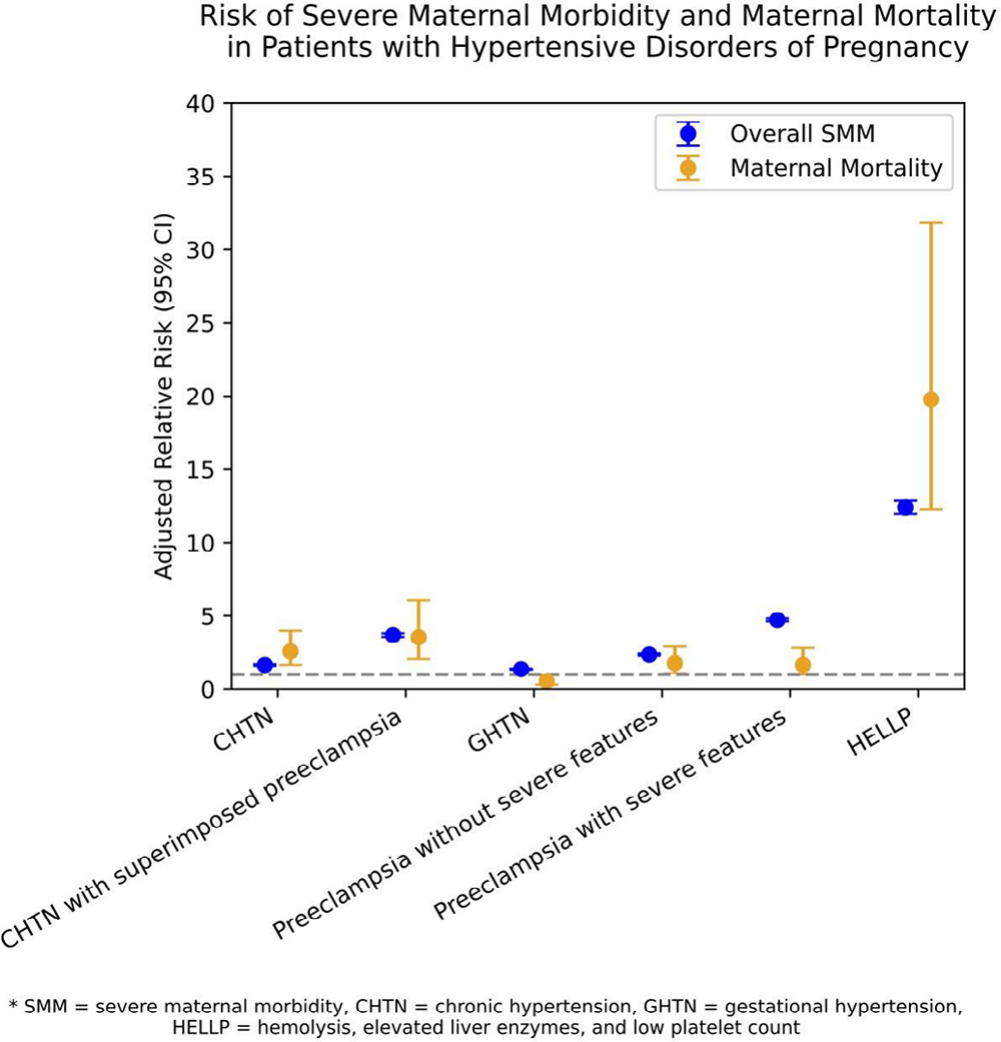

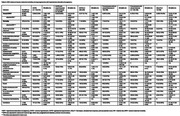

**3:30 PM – 5:00 PM**

## 450 | **Beyond Maternal Indications: Considering Fetal Risk in the Management of Severe Preeclampsia**

Brooke Schroeder^1^; Sally Kuehn^1^; Jaye Boissiere^1^; Madison Calvert^2^; Hannaneh Mirmozaffari^3^; Maya Patel^3^; Ashley Ruhashya^3^; Matt Fuller^1^; Jerome J. Federspiel^1^; Marie‐Louise Meng^1^; Johanna Quist‐Nelson^3^; Kim Boggess^4^

^1^Duke University School of Medicine, Durham, NC; ^2^University of North Carolina School of Medicine, Chapel Hill, NC; ^3^University of North Carolina, Chapel Hill, Chapel Hill, NC; ^4^University of North Carolina at Chapel Hill, Chapel Hill, NC

**Objective**: Preeclampsia with severe features (PEC‐SF) is a major cause of maternal and neonatal morbidity. We aimed to estimate the association between number of and individual severe features (SF) and perinatal morbidity.

**Study Design**: We conducted a retrospective cohort study of patients diagnosed with PEC‐SF who delivered at two tertiary academic medical centers between 12/2015 and 12/2017. Patients were identified using ICD‐10 codes, and clinical data were abstracted through chart review. The primary exposure was the number of SF present at diagnosis, while secondary analyses assessed the association between specific SF, as defined by ACOG criteria, and perinatal outcomes, including gestational age (GA) at delivery, mode of delivery, small for gestational age (SGA), placental abruption, and intrauterine fetal demise (IUFD).

**Results**: 773 patients met criteria for PEC‐SF. Of these, 411 (53.2%) had one SF, 273 (35.3%) had two, 65 (8.4%) had three, and 24 (3.1%) had four to six SF. Increasing number of SF was significantly associated with earlier GA at delivery, both scheduled and intrapartum cesarean delivery (CD), and IUFD (Table 1). SGA also occurred most often in neonates born to patients with 4–6 SF (*p* = 0.04). By individual SF criteria, pulmonary edema had the highest proportion of adverse perinatal outcomes: 50% of neonates were delivered <34 weeks, and mothers with pulmonary edema had the highest rates of placental abruption and IUFD. CD rates were high across all subtypes (38–56%), with the highest intrapartum CD rate observed in patients with renal dysfunction. SGA was most prevalent among patients with thrombocytopenia (Table 2).

**Conclusion**: Although the presence of more than three severe features typically necessitates delivery for maternal indications, our findings demonstrate this level of disease severity also carries substantial fetal risk—particularly heightened likelihood of intrauterine fetal demise.

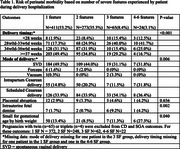

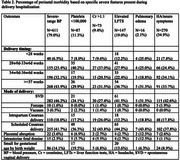

**3:30 PM – 5:00 PM**

## 451 | **Retrospective** Analysis **of AI Alarm Rate in Prenatal Cardiac Screening**

Noah AB Saphier^1^; Carl J. Saphier^2^

^1^Dwight‐Englewood School, Englewood, NJ; ^2^Women's Ultrasound, LLC, Englewood, NJ

**Objective**: Implementing AI to cardiac anomaly screening requires a high sensitivity and low alarm rate to avoid increasing the burden to healthcare systems which need to evaluate cases for second tier evaluations. We aim to evaluate the alarm rate of an AI‐based tool applied to a large and diverse retrospective dataset performed at a single center by a single practitioner.

**Study Design**: We retrospectively analyzed 3925 ultrasound exams of singleton pregnancies at 18–24 weeks performed and interpreted solely by a single MFM physician‐sonographer at a single center in the United States according to AIUM practice parameters from 2014 to 2025. An AI software developed by BrightHeart was applied to first assess view completeness (≥ 2 seconds of 4CH, LVOT and RVOT views in grayscale ultrasound cines). For the 1858 scans meeting view completeness criteria, the AI software analyzed all cines and assessed the presence of 8 morphological findings suspicious for CHD.

The primary outcome was the alarm rate (presence of an AI‐identified flag) in fetuses determined to be morphologically normal during the ultrasound exam and subsequent clinical follow‐up.

**Results**: Of the 1858 ultrasound exams, 1753 had no cardiac abnormality, 100 had minor suspected abnormality and 5 had severe CHD identified. The AI alarm rate in exams without cardiac abnormality was 5.5% (95% CI: 4.6–6.7) and remained low across subgroups including BMI, fetal presentation, and 3 generations of ultrasound devices (see Figure). The AI software identified at least 1 flag in all 5 cases with severe CHD.

**Conclusion**: Retrospective application of an AI tool demonstrated accurate detection of severe CHD, while maintaining a low alarm rate among exams without cardiac abnormality across a diversity of BMI, range of fetal presentations, and several generations of ultrasound devices. The findings suggest the robustness of AI to provide a low alarm rate while also providing a high detection of severe CHD, thereby avoiding an overwhelming burden for a second tier evaluation.

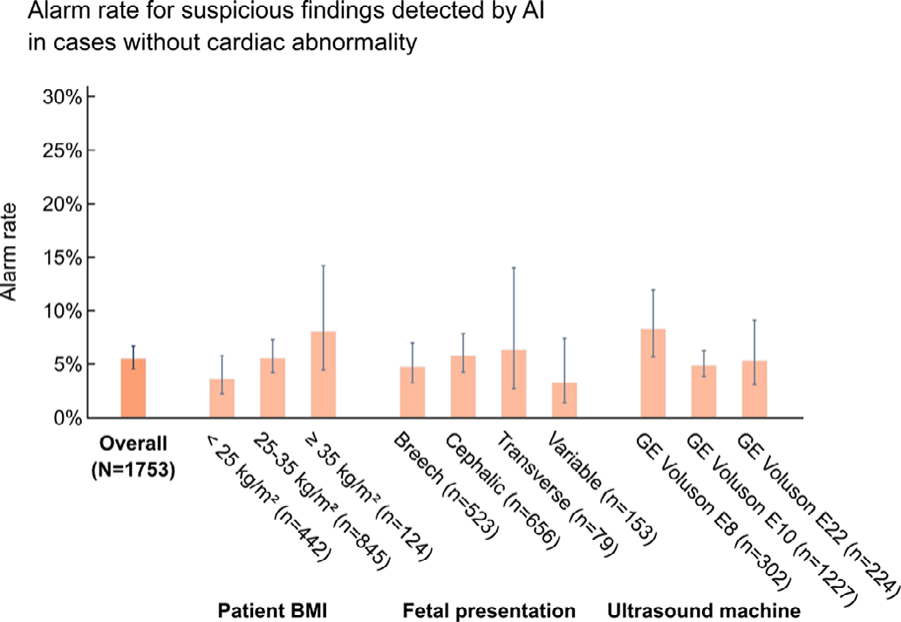

**3:30 PM – 5:00 PM**

## 453 | **Platelet Count** Trends **Across Consecutive Deliveries in Women With Gestational Thrombocytopenia**

Carolyn Sharim^1^; Yael Yekel^2^; Ron Rosenberg^3^; Tzippy Shochat^4^

^1^Laniado Hospital, Kochav Yair, HaMerkaz; ^2^Laniado Hospital, Laniado hospital, Netanya, HaMerkaz; ^3^Laniado Hospital, Laniado hospital Netanya, HaMerkaz; ^4^Israel

**Objective**: To evaluate whether women with gestational thrombocytopenia (GT), defined as a platelet count (PLT) <150,000/µL at the time of delivery, are at increased risk for recurrent thrombocytopenia in subsequent pregnancies, and to identify clinical or hematologic patterns that may predict recurrence.

**Study Design**: A retrospective cohort study conducted at secondary‐level public hospital between 2018 and 2022. Women with at least four deliveries, and with GT identified in one of them, were included. Exclusion criteria included multiple gestation, liver dysfunction, hypertensive disorders, hematologic diseases (including ITP), and viral infections (e.g., COVID‐19). Data included demographic and obstetric variables, and PLT values from each delivery. Statistical analysis included Wilcoxon signed‐rank tests and unadjusted logistic regression.

**Results**:

- A total of **443 women** were included.
- There was **no significant overall trend** in platelet counts between the first delivery with thrombocytopenia and subsequent deliveries (*p* = 0.92 for first vs. second delivery).
- Approximately **35%** of women with platelet counts of **120–150 K/µL** in their first thrombocytopenic delivery had **normalized counts** (>150 K/µL) in subsequent births.
- Women with **PLT <80 K/µL** showed a **tendency toward persistently low counts** across future deliveries.
- **Unadjusted logistic regression** demonstrated **no significant association** between PLT ≤150 K/µL at the second delivery and maternal age, parity, inter‐delivery interval, neonatal sex, birthweight, or Rh status.

**Conclusion**: Most women with GT do not experience a progressively worsening course in subsequent deliveries. While a minority demonstrate persistently low or worsening platelet trends, a considerable proportion recover to normal counts. These findings support a conservative approach to managing GT in the absence of other risk factors and emphasize the need for individualized risk assessment in future pregnancies.

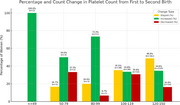

**3:30 PM – 5:00 PM**

## 454 | **Estimated versus Quantitative Blood Loss: Association with Blood Transfusion**

Cassidy A. O'Sullivan^1^; Gary R. Kersbergen^2^; Suneet P. Chauhan^3^; Jessica C. Fields^4^; Matthew K. Hoffman^2^

^1^Christiana Care Health Services, Newark, DE; ^2^Christiana Care Health System, Newark, DE; ^3^Delaware Center for Maternal‐Fetal Medicine of Christiana Care, Newark, DE; ^4^Delaware Center for Maternal Fetal Medicine, Newark, DE

**Objective**: In 2020, Joint Commission endorsed quantitative blood loss assessment (QBL) over estimated blood loss (EBL) as a more accurate assessment of blood loss during delivery. Nonetheless, whether QBL improves outcomes such as appropriate blood transfusion is understudied. This study compares EBL and QBL and their correlation with transfusion outcomes.

**Study Design**: Our retrospective review focused on deliveries at a single academic center, with concurrent EBL and QBL recorded at delivery. We excluded deliveries without both EBL & QBL, home births, and planned cesarean hysterectomy. The primary outcomes were correlation between EBL and QBL, overall blood transfusion rate, and predictive value of each method for transfusion risk. Correlation analysis and Bland‐Altman plotting were used to assess agreement between EBL and QBL. Logistic regression modeling and area under receiver operating characteristic curves (AUC) evaluated the predictive strength of EBL and QBL for transfusion need.

**Results**: There were 3355 deliveries which met the inclusion criteria. Among the cohort, 70 (2.1%) had a blood transfusion. The correlation between EBL and QBL was moderate at 40.3%. Bland‐Altman analysis revealed that QBL consistently estimated higher blood loss compared to EBL, especially as the volume of loss increased (Figure 1). Logistic regression demonstrated that both EBL and QBL similarly predicted transfusion. Both EBL (AUC 0.82, 95% CI 0.76–0.88) and QBL (AUC 0.79, 95% CI 0.72–0.86) had similar (*p* = 0.53; Figure 2) accuracy to identify individuals who were transfused.

**Conclusion**: While EBL and QBL demonstrate only moderate correlation, both are equally effective in identifying individuals who were transfused. QBL may offer a more conservative estimate of blood loss, particularly in high‐volume cases, but EBL remains clinically valid for predicting transfusion risk.

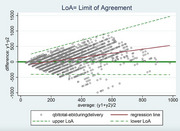

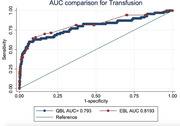

**3:30 PM – 5:00 PM**

## 455 | **Optimizing** Obstetric **Ultrasound Education: A Randomized Trial of Pocket‐Sized Ultrasound Probes Versus Traditional Ultrasound Machines**

Cecilia Leggett^1^; Saumaya Sao^2^; Alexandra C. Gallagher^3^; Hannah Moran^4^; Jane Chueh^4^; Elizabeth B. Sherwin^5^; Irogue I. Igbinosa^5^; Erica Wu^4^; Emi Komatsu^4^; Amy Judy^4^

^1^Stanford University School of Medicine, Palo Alto, CA; ^2^Stanford University School of Medicine, Stanford, CA; ^3^Stanford University, Palo Alto, CA; ^4^Stanford University, Stanford University, CA; ^5^Stanford University, Stanford, CA

**Objective**: Ultrasound curriculum implementation is disparate among obstetrics and gynecology (OBGYN) training programs due to difficulty integrating learning goals into clinical environments. This study evaluates whether providing trainees with handheld ultrasound devices (pocket probes) improves education outcomes compared to traditional ultrasound machines.

**Study Design**: A prospective, randomized controlled trial was conducted at an urban academic center from January‐June 2025. OBGYN trainees on a labor and delivery rotation were randomized to either a pocket probe (Butterfly iQ3) that connected to personal devices for image display or traditional ultrasound machines (GE Voluson P8) available on the unit. All participants received a digital obstetric ultrasound curriculum.

The primary outcome was curriculum completion measured by number and type of scans performed. 7 participants per group were needed to detect a mean difference of 18% (alpha 0.05).

Secondary outcomes included knowledge assessment scores, image quality, and perceived workload assessed by a validated survey (NASA Task Load Index). Mann‐Whitney, paired T‐tests, and chi‐square tests were used for analysis.

**Results**: 23 trainees were enrolled (13 pocket, 10 traditional). Groups were similar in age, training year, pre‐curriculum scores, and shift schedules.

The pocket group had significantly higher curriculum completion (median 67% vs 8.3%, *p* = 0.0016, Figure 1). Image quality scores were significantly higher in the pocket group (4.0 vs 3.0, *p* = 0.0003). Post‐curriculum knowledge scores were not significantly different between groups.

The pocket group more often rated workload as low/moderate, while controls rate

D workload high/very high (*p* = 0.046).Lower mental, physical, temporal demand, and lower frustration were seen among those randomized to pocket probes (Figure 2).

**Conclusion**: Use of pocket‐sized ultrasound probes was associated with significantly greater curriculum

engagement, improved image quality, and lower perceived workload among OBGYN trainees in clinical training environments.

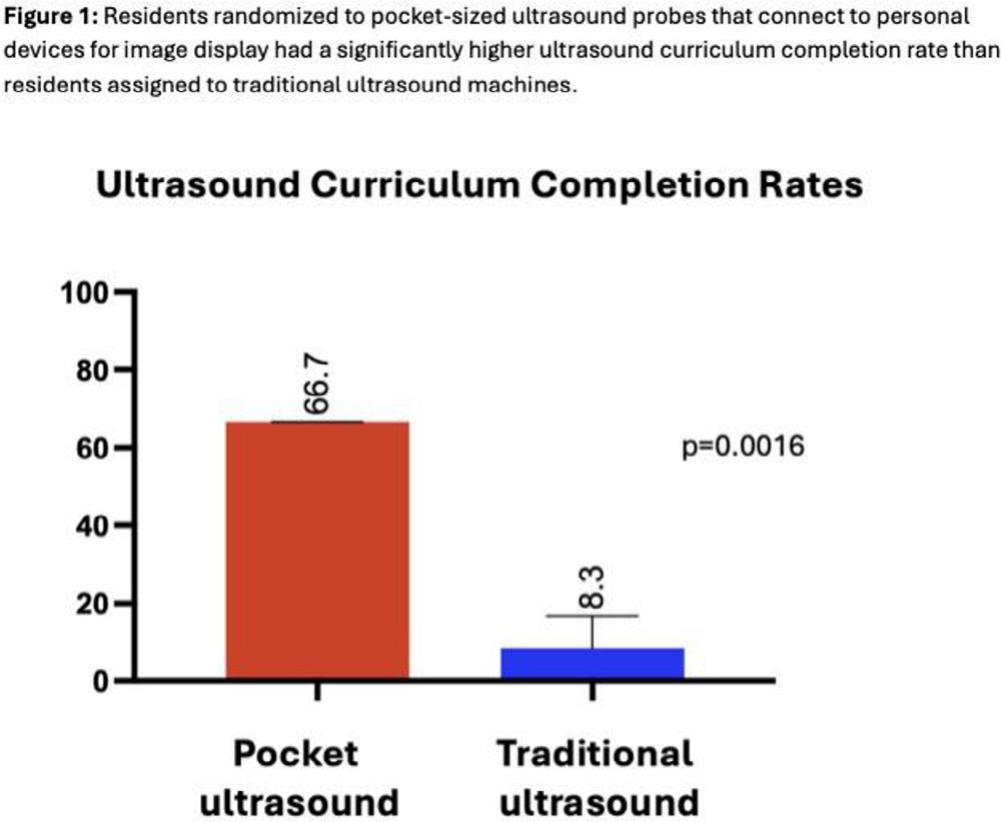

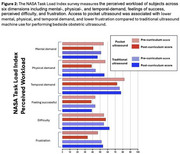

**3:30 PM – 5:00 PM**

## 456 | **A Decision Tree** Model **Including Sonographic Measurement of Head‐Perineum Distance to Predict Difficult Vacuum Delivery**

Maeva Gimaret^1^; Nassima Ramdane^1^; Bertrand Gachon^2^; Charles Garabedian^1^

^1^CHU Lille, Lille, Nord‐Pas‐de‐Calais; ^2^CHU Poitiers, Department of Obstetrics and Gynecology, Poitiers University Hospital, Poitou‐Charentes

**Objective**: Ultrasound use during birth is a relevant and objective method to help in decision‐making in the delivery room. It seems like ultrasound use can predict “difficult birth”. However, there is an absence of consensus concerning the HPD measurement and these studies are essentially single‐centre studies.

Thus the objective was to build a decision tree model including sonographic measurement of head‐perineum distance to predict difficult vacuum delivery.

**Study Design**: This was a secondary analysis of a prospective, multicentric cohort‐based study involving 111 private and public maternities in France (Instrumoda study, Gachon et al., AJOG, 2025). Women included were nulliparous women, aged of 18 years or more, with deliveries at 34 weeks of gestation or more, of singleton fetuses in cephalic presentation. In this present study, only women who had a vacuum delivery with measurement of head‐perineum distance before operative vaginal delivery, whether the final delivery route, were included. HPD was measured in a frontal transperineal scan as the shortest distance from the outer bony limit of the fetal skull to the perineum. Difficult instrumental delivery was defined as a change of instrument or a c‐section.

**Results**: 2644 cases of vacuum delivery with HPD measurement were included of which 343 (13%) were classified as difficult. HPD was higher in the failure group (43,0 +‐ 10,1 vs 38,0 +‐ 11,3 mm, *p* < 0,001). Vacuum failures seem to increase with HPD: there is 1,5% of failure in the 0–20 mm HPD group whilst there is 39,1% in the 40–49 mm HPD group. The decision tree was created to establish thresholds according to the fetal position. In the case of an anterior presentation with a DTP <36.5 mm, the risk of vacuum failure was 6%. On the other hand, in the case of a posterior variety with a DTP >30.5 mm, the risk of vacuum failure was 22%.

**Conclusion**: HPD measurement associated with the fetal position can help predict a difficult vacuum delivery. It will be interesting to evaluate this tree in other populations. (INSTRUMODA Study Group)

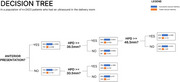

**3:30 PM – 5:00 PM**

## 457 | **Association** Between **Definitions of Fetal Growth Restriction and Placental Insufficiency**

Chinonye Imo^1^; Alexa A. Freedman^2^; Ryan Keltner^3^; Marci Adams^3^; Linda M. Ernst^4^; Sunitha Suresh^5^

^1^Endeavor Health / University of Chicago, Chicago, IL; ^2^Northwestern University, Chicago, IL; ^3^Endeavor Health, Endeavor Health, IL; ^4^Endeavor Health, Evanston, IL; ^5^Endeavor Health, Endeavor Health System, IL

**Objective**: Contemporary definitions of fetal growth restriction (FGR) include diagnosis by AC <10^th^ percentile alone, with controversy if similar perinatal risk exists in this cohort of patients. We aimed to examine the prevalence of placental pathology, a marker of placental insufficiency, in association with classic vs contemporary definition of FGR.

**Study Design**: This is a retrospective study of patients in a large health system from 01/2021 to 01/2025 diagnosed with FGR on ultrasound within 1 month of delivery. The exposure of FGR was classified based on the contemporary (AC <10^th^ percentile and EFW >10^th^ percentile) vs traditional (EFW <10^th^ percentile with or without AC <10^th^ percentile) definitions. Our primary outcome was placental insufficiency, determined by presence of maternal vascular malperfusion (MVM) by a single perinatal pathologist. Secondary outcomes included fetal vascular malformation (FVM), chronic inflammation (CI), and acute inflammation (AI), and a composite of chronic pathology (MVM, FVM, CI). Statistical analyses included chi‐square, Wilcoxon rank‐sum, T‐test for univariate analysis and logistic regression for multivariable analysis, adjusted for age, BMI, race and gestational diabetes.

**Results**: 400 patients had FGR and placental pathology available during the study period, 146 patients with traditional and 254 patients with contemporary definition. There were no demographic differences (Table 1). Across both diagnostic definitions, there was a high prevalence of placental insufficiency. There was no difference in the presence of MVM between the two groups (98 (67.1%) traditional vs 166 (65.4%) contemporary, *p* = 0.72, aOR 1.04 95% CI 0.67–1.62, Table 2). The majority of patients in both cohorts had chronic placental pathology (88.4% traditional vs 88.2% contemporary, *p* = 0.96, aOR 1.05 95% CI 0.55–2.01, Table 2).

**Conclusion**: FGR diagnosed by contemporary and traditional definitions have similarly high rates of placental insufficiency, supporting equal management of patients given the known association of placental insufficiency and adverse perinatal outcomes including stillbirth.

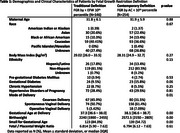

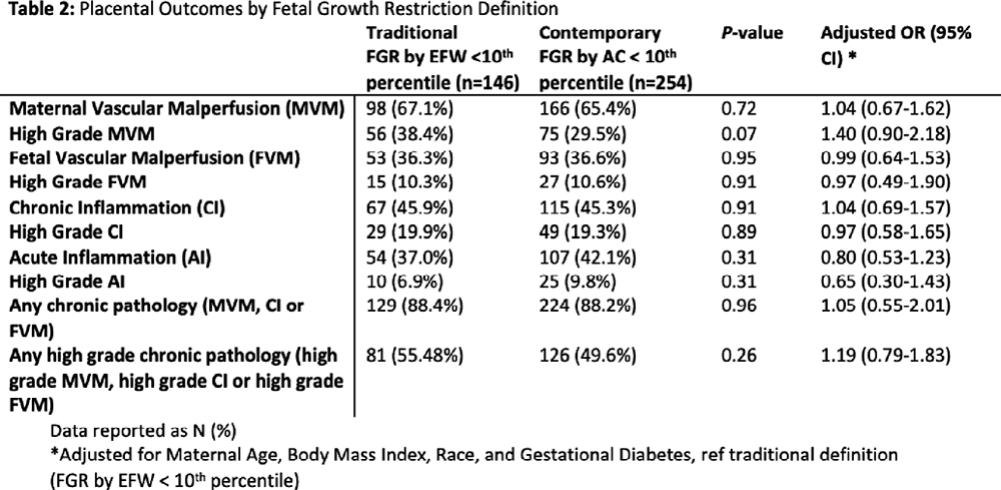

**3:30 PM – 5:00 PM**

## 458 | **Periconception** Hemoglobin **A1c and the Risk of Neurodevelopmental Disorders in the Offspring**

Christina N. Schmidt^1^; Kaitlyn E. James^2^; Sarah Hsu^3^; Siguo Li^4^; Andrea G. Edlow^2^; Mark A. Clapp^5^; Camille E. Powe^6^; Lydia L. Shook^7^

^1^Department of Obstetrics and Gynecology, MassGeneral Brigham, Boston, MA; ^2^Department of Obstetrics and Gynecology, MassGeneral Brigham; Division of Maternal‐Fetal Medicine; Harvard Medical School, Boston, MA; ^3^Department of Medicine, MassGeneral Brigham, Mass General Brigham, MA; ^4^Department of Obstetrics and Gynecology, Mass General Brigham, Boston, MA; ^5^Department of Obstetrics and Gynecology, Mass General Brigham, Massachusetts General Hospital, MA; ^6^Massachusetts General Hospital, Boston, MA; ^7^Department of Obstetrics and Gynecology, MassGeneral Brigham; Division of Maternal‐Fetal Medicine; Harvard Medical School, Massachusetts General Hospital/Boston, MA

**Objective**: Maternal pregestational diabetes is associated with an increased risk of neurodevelopmental disorders in children. The objective of this study is to evaluate whether periconception hemoglobin A1c (A1c) is associated with the diagnosis of a neurodevelopmental disorder (ND) among offspring.

**Study Design**: This retrospective cohort included live singleton births with an observed periconception A1c from 2016–2023 across four hospitals in an integrated healthcare system. Periconception A1c was defined as the nearest A1c within 90 days before or after the estimated date of conception. The outcome of interest was documentation of a ND (ICD‐10 diagnostic codes F80–F84) in the offspring electronic health record. Multiple logistic regression with cluster‐robust standard errors was used to assess the association between A1c and ND. Models were adjusted for maternal age, race/ethnicity, insurance status, pre‐pregnancy BMI, infant sex, and preterm birth.

**Results**: Of the 16,917 included pregnancies, ND was identified in 1013 (6.0%) offspring. The average periconception A1c was 5.2 (SD 0.6). 1452 had a diagnosis of pregestational diabetes (8.6%). In the adjusted model, a 1‐point increase in periconception A1c was associated with 12% increased odds of a ND diagnosis (aOR 1.12; [95% CI 1.02–1.22]; see Table 1). BMI, Hispanic ethnicity, public insurance, male sex, and preterm birth were also associated with increased ND risk.

**Conclusion**: Periconception hyperglycemia is associated with increased risk of ND in offspring in early childhood. These findings highlight the preconception period as a critical intervention window for improving long‐term offspring ND outcomes.

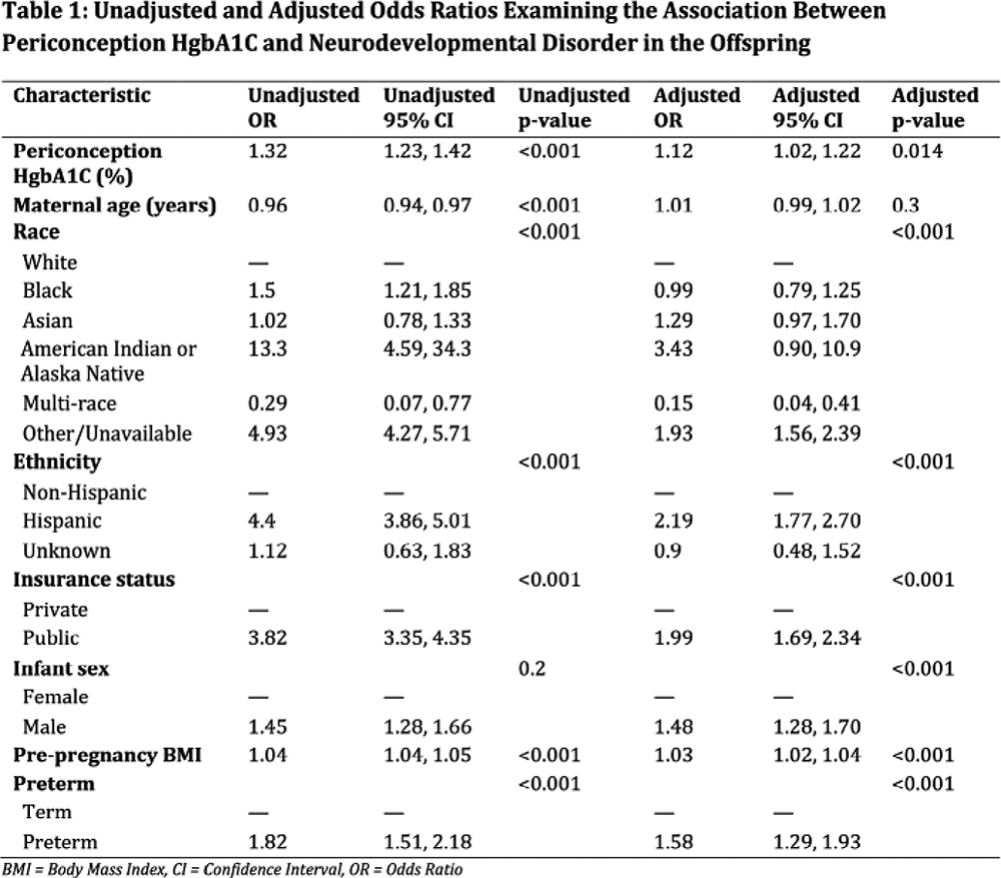

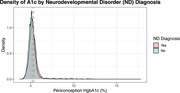

**3:30 PM – 5:00 PM**

## 459 | **Risk of Recurrent Stillbirth in a Subsequent Pregnancy**

Sophi Farid; Christine E. Henricks; Donald D. McIntire; F. Gary Cunningham; David B. Nelson; Elaine L. Duryea

University of Texas Southwestern Medical Center, Dallas, TX

**Objective**: This study aimed to quantify the risk of recurrent stillbirth following an initial stillbirth. A second aim was to evaluate the contribution of placental insufficiency to the recurrence risk.

**Study Design**: This was a retrospective cohort study of 77,689 women with two consecutive singleton deliveries at a single academic institution between 2000–2023. Stillbirth was defined as a fetal demise occurring at ≥20 weeks of gestation or with a birth weight ≥500 g. Women who experienced a stillbirth in their first pregnancy were compared to those who had a live birth, to assess whether stillbirth increased the risk of recurrent fetal demise in the subsequent pregnancy. A multidisciplinary stillbirth review committee routinely studied the etiology of all stillborn infants. Identification of stillbirth secondary to placental insufficiency was based on integrated evaluation of placental pathology, autopsy findings, and genetic testing. A subgroup analysis examined those with prior placental insufficiency. Statistical analysis was performed including adjustment for maternal age as continuous variable with *p* < 0.05 considered significant.

**Results**: A prior stillbirth was associated with a significantly increased risk of stillbirth in a subsequent pregnancy (RR 2.44, 95% CI 1.10–5.45, *p* = 0.025). This relationship remained statistically significant after adjustment (aRR 2.33, 95% CI 1.05–5.20, *p* = 0.04). Among patients whose initial stillbirth was due to placental insufficiency, the risk of recurrence was significantly higher (RR 6.00, 95% CI 1.52–23.59, *p* = <0.01).

**Conclusion**: Women who experience a stillbirth in their first pregnancy have a significantly increased risk of stillbirth in their next pregnancy, with a markedly higher risk observed when the initial loss is due to placental insufficiency. These findings underscore the importance of determining the cause of prior stillbirth and surveillance in subsequent pregnancies.

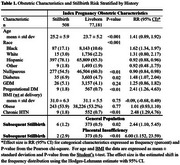

**3:30 PM – 5:00 PM**

## 460 | **Is there an** Association **Between Parity and Neurovascular Complications in Patients With Sickle Cell Disease?**

Chrystelle El‐Khoury^1^; Bi Lan Wo^1^; Mi‐Suk Kang Dufour^2^; Jonathan St‐Onge^1^; Melika Safa^3^; Stéphanie Forté^1^

^1^Centre Hospitalier de l'Université de Montréal, Montreal, PQ; ^2^Centre Hospitalier Universitaire Sainte‐Justine, Montreal, PQ; ^3^Université de Montréal, Montreal, PQ

**Objective**: Both pregnancy and sickle cell disease (SCD) increase neurovascular risk. However, it remains unknown whether pregnancy specifically increases the risk of neurovascular complications (NVCs) in women with SCD. Our objective was to evaluate whether parity is associated with clinical (stroke or transient ischemic attack), functional (neurocognitive disorder or abnormal score on the Montreal Cognitive Assessment, Rowland Universal Dementia Assessment Scale or Modified Rankin Scale for Neurologic Disability) or imaging (cerebrovascular lesions) NVCs in this population.

**Study Design**: We performed a single‐center retrospective cohort study of patients with SCD managed at our neuro‐hematology clinic. Baseline characteristics were obtained between November 2018 and April 2024 through chart review and standardized phone interview. We included all pregnancies that reached a gestational age ≥20 weeks regardless of outcome. Participants were stratified as nulliparous (parity = 0) or multiparous (parity ≥1). The prevalence of each NVC category was compared between groups using Fisher's exact test.

**Results**: Among 148 participants, 68 (45.9%) had at least 1 pregnancy of 20 weeks or more. Overall, 7/148 (4.7%) experienced a clinical NVC, 65/148 (43.9%) met functional NVC criteria, and 103/148 (69.6%) demonstrated cerebrovascular lesions on neuroimaging. The prevalence of clinical (2.5% vs 7.4%, *p* = 0.248), functional (40.0% vs 48.5%, *p* = 0.406) and imaging‐defined (66.3% vs 73.5%, *p* = 0.374) NVCs did not differ significantly when compared for parity.

**Conclusion**: Parity was not associated with clinical, functional or imaging NVCs in our SCD cohort. Larger, multicentric studies and multivariable analyses are warranted to confirm these findings and clarify risk stratification for patients with SCD who wish to become pregnant. Long‐term follow‐up and systematic documentation of obstetrical history are essential to determine whether pregnancy is a risk factor for NVCs.

**3:30 PM – 5:00 PM**

## 461 | **Seasonal** Associations **Between Gastroschisis Rates and Pesticide Application in California**

Citlali I. Lopez^1^; Alejandro Perez^1^; Bharti Garg^2^; Joshua F. Robinson^3^; Aaron B. Caughey^2^; Stephanie L. Gaw^3^

^1^University of California San Francisco, San Francisco, CA; ^2^Oregon Health & Science University, Oregon Health & Science University, OR; ^3^University of California, San Francisco, San Francisco, CA

**Objective**: Gastroschisis is a congenital abdominal wall defect with unclear etiology. We investigated associations between seasonal rates of gastroschisis and pesticide application during the critical window of development.

**Study Design**: This is a retrospective cohort study of CA linked hospital discharge‐vital statistics data from 2016–2020. Gastroschisis rates were determined by zip code of maternal residence. Gastroschisis rates were organized by counties growing commodities with high pesticide application (>4 mill pounds applied or >3 mill acres treated). Data from the CA Department of Pesticide Regulation was used to identify the month of application of the top 100 pesticides applied. County‐wide applications were categorized as “any” or “high” (>25%ile or >75%ile by pounds per land area, respectively). Risk of gastroschisis was analyzed by month, commodity, and amount of application by county for pesticides applied during the critical window. Statistical analysis was performed by chi squared or multivariable logistic regression.

**Results**: Rates of gastroschisis births were higher in May and June (0.04%) and lower in December (0.01%) in California (*p* < 0.001). Analysis by commodity grown confirmed seasonal rates of gastroschisis for 9 of 13 commodities (Figure 1). Counties growing cotton had the greatest seasonal variation in gastroschisis rates (0.08% in May‐June and 0.03% in December, *p* = 0.018). We analyzed risk of gastroschisis for 14 pesticides highly applied during the critical window (Figure 2). Any application of sulphur, paraquat dichloride, and chlorpyrifos was associated with increased risk of gastroschisis. High application of pendimethalin, sulphur, paraquat dichloride, chlorpyrifos, urea dihydrogen sulfate, methoxyfenozide, and azoxystrobin was associated with increased odds of gastroschisis.

**Conclusion**: There are seasonal trends of gastroschisis births in CA that are also associated with specific commodities. Pesticides highly applied during the critical window of development are associated with increased risk of gastroschisis.

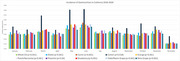

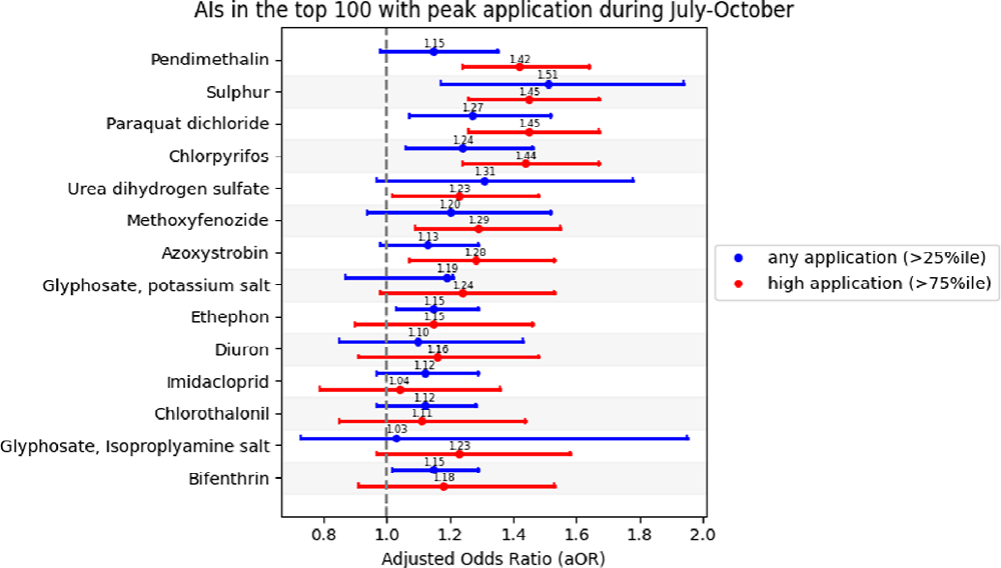

**3:30 PM – 5:00 PM**

## 462 | **Incidence of** Recurrent **Preterm Birth Before and After the PROLONG Trial**

Claire H. Packer^1^; Taylor S. Freret^2^; Kaitlyn E. James^3^; Sarah E. Little^4^; Alexander Melamed^5^; Mark A. Clapp^6^

^1^Mass General Brigham, Boston, MA; ^2^Beth Israel Deaconess Medical Center, Brookline, MA; ^3^Massachusetts General Hospital, Boston, MA; ^4^Beth Israel Deaconess Medical Center, Newton, MA; ^5^Department of Obstetrics and Gynecology, Mass General Brigham, Boston, MA; ^6^Department of Obstetrics and Gynecology, Mass General Brigham, Massachusetts General Hospital, MA

**Objective**: After its initial FDA approval in 2011, 17‐hydroxyprogesterone (17‐OH‐P) was prescribed to patients with a history of spontaneous preterm birth (PTB) to reduce the risk of recurrence. In the confirmatory PROLONG trial, 17‐OH‐P did not decrease recurrent PTB. In a contemporary, US‐based cohort, our objective was to assess if the PROLONG trial changed 17‐OH‐P prescribing practices and to compare the rates of PTB before and after the trial.

**Study Design**: We performed a pre‐post analysis of singleton patients with a history of PTB receiving care within a single, large health care system between July 1, 2016, and February 28, 2023. Pregnancies at 20 weeks between October 2019 and September 2020 were excluded to allow for any potential practice changes to take effect; this also excluded pregnancies delivered during the height of the COVID‐19 pandemic. The primary outcomes were 17‐OH‐P use and PTB <35 weeks. Secondary outcomes were PTB <32 weeks and PTB <37 weeks. Chi‐square tests and logistic regression were used, adjusting for maternal age, race, ethnicity, insurance, and number of prior PTBs.

**Results**: 1719 patients in the pre‐group and 1780 in the post‐group were included. 17‐OH‐P use decreased from 23.6% to 5.7% between the two periods (*p* < 0.001). There was no significant difference in rates of PTB between the time periods (<35 weeks: 10.2 vs 10.7%, *p* = 0.64; <32 weeks: 4.1 vs. 3.8%, *p* = 0.70; <37 weeks: 23.2 vs. 24.5%, *p* = 0.37). In the logistic regression model, there was no significantly increased risk of preterm birth for delivering after the PROLONG trial compared to before (adjusted odds ratio for PTB <32 weeks for delivering in the post vs. pre‐PROLONG period: 1.00 (0.80–1.26)), despite the notable change in 17‐OH‐P use.

**Conclusion**: Within a single health system, 17‐OH‐P use dropped by >75% after the PROLONG trial with no measurable changes in PTB rates. These findings support the conclusions of the PROLONG trial, though additional confirmatory research with larger sample sizes to investigate for potential differential effects among population subgroups.

**3:30 PM – 5:00 PM**

## 463 | **Cardiac** Remodeling **In Fetuses Exposed to Threatened Preterm Labor Delivered Near or at Term**

Clara Murillo Bravo *^1^; Claudia Rueda^2^; Marta Larroya^2^; David Boada^2^; Laia Grau^3^; Júlia Ponce^2^; Ana Herranz^4^; Olga Gomez^4^; Sílvia Ferrero^5^; Vicente Andreu‐Fernández^6^; Eduard Gratacós^2^; Fatima Crispi^4^; Montse Palacio *^2^; Teresa Cobo^7^

^1^Hospital Clínic Barcelona, HOSPITAL CLINIC BARCELONA / BARCELONA, Catalonia; ^2^Hospital Clínic Barcelona, BARCELONA, Catalonia; ^3^Hospital Sant Joan de Deu, BARCELONA, Catalonia; ^4^Hospital clinic Barcelona, BARCELONA, Catalonia; ^5^BCNatal–Barcelona Center for Maternal Fetal and Neonatal Medicine Hospital Sant Joan de Déu and Hospital Clínic, University of Barcelona, BARCELONA, Catalonia; ^6^Biosanitary Research Institute, Valencian International University, VALENCIA, Comunidad Valenciana; ^7^Hospital Clinic Barcelona, Hospital Clinic (Barcelona), Catalonia

**Objective**: To evaluate whether fetuses exposed to threatened preterm labor (PTL) without intra‐amniotic infection or inflammation (IAI) and delivered after 34 weeks show cardiac remodeling and dysfunction at admission, and whether these changes persist one month later.

**Study Design**: This prospective cohort study included singleton pregnancies with PTL between 23–34 weeks, confirmed absence of IAI via amniocentesis, a latency to delivery exceeding four weeks, and birth beyond 34 weeks. Fetal echocardiography was performed at admission and one month later. A gestational age‐matched control group was also assessed. Amniotic fluid levels of Troponin‐I and NT‐proBNP were compared to biobank samples at similar gestational ages.

**Results**: We analyzed 32 fetuses with threatened PTL and 43 controls, with comparable baseline characteristics including fetal sex, gestational age, and fetal growth. 84% of fetuses in the threatened PTL group were born at term. At admission (around 29 weeks of gestation), the threatened PTL group already showed signs of cardiac remodeling, including more globular hearts, enlarged right atrial areas, and subclinical diastolic dysfunction. Systolic function was mildly increased. These changes affected exclusively the right heart. Troponin‐I levels were significantly higher, and NT‐proBNP was detectable in 25% of threatened PTL cases compared to none in controls. One month later (around 34 weeks of gestation), systolic parameters had normalized, but signs of remodeling (lower sphericity index, larger right ventricular area) and diastolic dysfunction (prolonged inflow times) persisted, primarily affecting the right heart.

**Conclusion**: Fetuses exposed to threatened PTL without IAI and delivered near or at term exhibit persistent cardiac remodeling and subclinical diastolic dysfunction. Elevated amniotic fluid Troponin‐I at admission support these early echocardiographic findings. These results help identify a previously unrecognized high‐risk neonatal subgroup, born at or near term, that could benefit from targeted follow‐up and early preventive interventions.

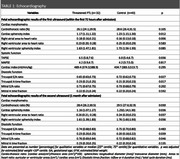

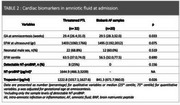

**3:30 PM – 5:00 PM**

## 464 | **Rescue** Antenatal **Corticosteroid Administration in Twin Pregnancies: A Cost‐Effectiveness Analysis**

Clarice G. Zhou^1^; Dana Senderoff Berger^1^; Diana S. Abbas^2^; Aaron B. Caughey^3^; Christina A. Penfield^4^

^1^Division of Maternal‐Fetal Medicine, Department of Obstetrics and Gynecology, NYU Grossman School of Medicine, NYU Grossman School of Medicine, NY; ^2^Department of Obstetrics and Gynecology, NYU Grossman School of Medicine, New York, NY; ^3^Oregon Health & Science University, Oregon Health & Science University, OR; ^4^Division of Maternal‐Fetal Medicine, Department of Obstetrics and Gynecology, NYU Grossman School of Medicine, New York City, NY

**Objective**: A second course of antenatal corticosteroids (ACS), used for “rescue,” has been shown to be beneficial for fetal maturation in singleton pregnancies at risk of preterm delivery <34 weeks, but studies have not evaluated the cost‐effectiveness of rescue ACS in twin pregnancies. This study aims to evaluate the cost‐effectiveness of a rescue course of ACS among twin pregnancies at risk of preterm delivery <34 weeks.

**Study Design**: A cost‐effectiveness model was constructed using TreeAge software to compare exposure to one versus two courses of betamethasone in a theoretical cohort of 14,000 twin pregnancies at risk of preterm delivery <34 weeks in the US per year. The outcomes included in the model were neonatal death and composite respiratory morbidity (including mechanical ventilation, respiratory distress syndrome, and bronchopulmonary dysplasia). Maternal quality‐adjusted life years (QALYs) were assessed. All probabilities, utilities, and costs were derived from published literature. Costs are expressed in 2025 US dollars with a willingness to pay threshold (WTP) set at $100,000/QALY. Deterministic and probabilistic sensitivity analyses were performed to interrogate model assumptions.

**Results**: In our theoretical cohort of 14,000 twin pregnancies at risk of preterm delivery <34 weeks gestation, exposure to rescue ACS resulted in 6 fewer neonatal deaths and 1237 fewer neonates with composite respiratory morbidity. Compared to a single course, rescue ACS was the dominant strategy, resulting in cost savings of $1026 million and greater effectiveness (33 maternal QALYs). In our sensitivity analyses, we found that administration of rescue ACS remained cost‐effective when the probability of respiratory morbidity following a first course was >10%.

**Conclusion**: In a theoretical cohort of twin pregnancies <34 weeks at risk of preterm delivery, a strategy of rescue ACS was associated with improved neonatal outcomes, reduced healthcare costs, and increased maternal QALYs. Rescue ACS, as the dominant strategy, appeared to be a cost‐effective intervention in this population.

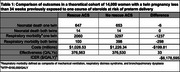

**3:30 PM – 5:00 PM**

## 465 | **Placental** Inflammation**, Angiogenesis and Senescence Markers in Preterm Premature Rupture of Membranes**

Claudia Rueda^1^; Clara Murillo Bravo *^2^; Silvia Ferrero^3^; Ana Paula Dantas^4^; Eduard Gratacós^1^; Francesc Figueras^5^; Laia Grau^6^; David Boada^1^; Ana Herranz^7^; Montse Palacio *^1^; Teresa Cobo^8^

^1^Hospital Clínic Barcelona, BARCELONA, Catalonia; ^2^Hospital Clínic Barcelona, HOSPITAL CLINIC BARCELONA / BARCELONA, Catalonia; ^3^Hospital Sant Joan de Déu, Barcelona, Catalonia; ^4^IDIBAPS, Barcelona, Catalonia; ^5^BCNatal–Barcelona Center for Maternal Fetal and Neonatal Medicine Hospital Sant Joan de Déu and Hospital Clínic, University of Barcelona, Barcelona, Catalonia; ^6^Hospital Sant Joan de Deu, Barcelona, Catalonia; ^7^Hospital clinic Barcelona, BARCELONA, Catalonia; ^8^Hospital Clinic Barcelona, Hospital Clinic (Barcelona), Catalonia

**Objective**: To characterize placental expression of genes involved in apoptosis and senescence, along with biomarkers related to angiogenesis, inflammation, tissue remodeling, and telomere length, in pregnancies complicated by preterm premature rupture of membranes (PPROM) compared to term asymptomatic pregnancies.

**Study Design**: A prospective cohort study including 54 singleton pregnancies: 25 presenting with PPROM between 23+6 and 34+0 weeks of gestation and 29 term pregnancies. Placental tissue samples were analyzed for protein concentrations related to angiogenesis, inflammation, and tissue remodeling via immunoassays. Gene expression of apoptosis and senescence markers, and relative telomere length (TR ratio), were assessed by quantitative PCR. Fold change was used to evaluate group differences. Group comparisons were performed using non‐parametric tests.

**Results**: Baseline maternal characteristics were similar between groups, with expected differences in clinical outcomes: lower gestational age at delivery, higher rates of labor induction, antenatal corticosteroid exposure, and increased neonatal morbidity in the PPROM group.

Placentas from PPROM pregnancies showed significantly higher levels of IL‐8, IL‐1β, MCP‐1, and G‐CSF, indicating an enhanced inflammatory response. Markers related to angiogenesis and tissue remodeling — VEGF, VEGFR1, TIE‐2, and FGF‐1 — were also significantly increased in PPROM cases.

Of the apoptosis and senescence‐related genes evaluated, only SIRT6 showed significantly higher expression in the PPROM group.

No significant differences were found in relative telomere length (TR ratio) between the two groups.

**Conclusion**: PPROM is associated with a distinct placental profile characterized by heightened inflammatory and angiogenic activity. Changes in the expression of apoptosis and senescence markers were limited, and no significant differences in telomere length were observed between groups. These findings emphasize the importance of characterizing the underlying physiopathology of PPROM, with inflammation and angiogenic dysfunction emerging as key features in these patients.

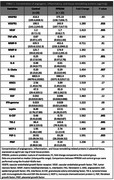

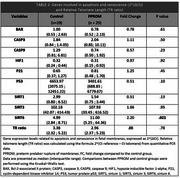

**3:30 PM – 5:00 PM**

## 466 | **Racialized** Disparities **in Adverse Health Outcomes During Second Trimester Medication Abortion**

Courtney N. Sullivan^1^; Vanya Manthena^1^; Jocelyn Wascher^1^; Eryn Wanyonyi^1^; Camille Johnson^1^; Lahari Vuppaladhadiam^1^; Julie Chor^1^; Beth Plunkett^2^; Isa Ryan^2^; Olivert Mbah^2^; Jungeun Lee^2^; Emily Barker^3^; Laura Laursen^4^; Leanne McCloskey^5^; Sloane York^4^; Ashish Premkumar^6^

^1^University of Chicago Medicine, Chicago, IL; ^2^Endeavor Health Evanston, Evanston, IL; ^3^Hope Clinic, Granite City, IL; ^4^Rush University Medical Center, Chicago, IL; ^5^Yale University, New Haven, CT; ^6^University of Chicago, Chicago, IL

**Objective**: To evaluate potential disparities in health outcomes for non‐Hispanic Black (NHB) people undergoing 2nd trimester (2T) medication abortion (MAB) compared to non‐Hispanic White (NHW) people.

**Study Design**: This was a retrospective cohort study of people undergoing 2T MAB at 4 tertiary care centers in Cook County, IL from 2009 to 2019. The primary exposure was self‐reported race. The primary outcome was composite morbidity (i.e., uterine rupture, blood transfusion, chorioamnionitis, intensive care unit admission, and hospital readmission within 6 weeks). The secondary outcomes were the composite morbidity without and all components of the composite. We performed multivariable regression analyses via backwards stepwise selection of covariates, with eligibility based on *p* < 0.10 on bivariate analyses. A priori, we controlled for mifepristone use, the only covariate ultimately selected for inclusion. Subgroup analyses were performed based on uterine scarring and parity. Pairwise comparisons were done based on gestational duration. A post‐hoc sensitivity analysis was conducted excluding preterm prelabor rupture of membranes, preterm labor, and cervical insufficiency.

**Results**: 669 people were included, 51.3% identifying as NHB. NHB people had a higher BMI, lower frequency of prior uterine scarring, and presented 1 week earlier in gestation (Table 1). NHB people had a higher frequency of the primary outcome, which persisted when controlling for mifepristone (aRR 1.50, 95% CI 1.10–2.05). The secondary outcome was not significantly different between groups. The only significant difference in the components of the primary outcome was a higher frequency of chorioamnionitis among NHB people (Table 2). No significant difference was found on subgroup analyses based on uterine scarring or parity. No significant difference was found in NHW people based on gestational duration at presentation. Sensitivity analyses were consistent with the primary findings.

**Conclusion**: NHB people have a higher risk of morbidity during 2T MAB compared to NHW, with the difference driven by clinical chorioamnionitis.

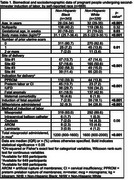

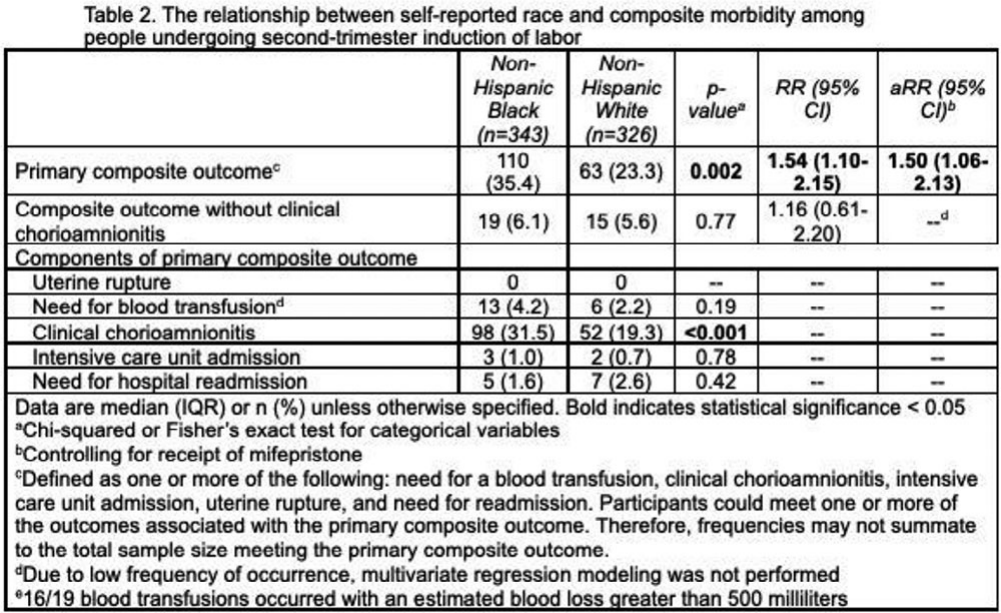

**3:30 PM – 5:00 PM**

## 467 | **Cord Tissue** Analysis **of Prenatal Buprenorphine Exposure and Neonatal Withdrawal**

Courtney Townsel^1^; Makeda Turner^2^; Fatemeh Abdollah Biroon^2^; Vedavalli Govindan^3^; Eric K. Broni^4^; Jeannie C. Kelly^5^

^1^University of Maryland School of Medicine, University of Maryland School of Medicine, MD; ^2^University of Maryland School of Medicine, Baltimore, MD; ^3^Campbell University School of Osteopathic Medicine, Lillington, NC; ^4^Yale University, New Haven, CT; ^5^Washington University School of Medicine, Washington University School of Medicine, MO

**Objective**: Neonatal opioid withdrawal syndrome (NOWS) occurs less frequently in buprenorphine than methadone‐exposed pregnancies. However, maternal buprenorphine dose does not reliably predict NOWS severity. This study assessed whether umbilical cord buprenorphine and norbuprenorphine levels are associated with maternal dose and NOWS severity.

**Study Design**: Secondary analysis of a multi‐site prospective study of pregnancies with opioid use disorder (OUD) between 2021–2025. Patients included if maintained on buprenorphine, delivered ≥34 weeks and had biospecimen collected at delivery. Major fetal anomalies and missing data were exclusions. Maternal and neonatal characteristics were abstracted. Umbilical cord tissue was collected after delivery, drained of blood, stored at ‐80°C and underwent mass spectrometry for quantification of buprenorphine and norbuprenorphine levels. NOWS was defined as any Finnegan score >4; severe NOWS as requiring pharmacotherapy or Finnegan score >24 in 24 hours. Pearson correlations assessed relationships between maternal dose, cord drug levels and NOWS. *p* < 0.05 was significant.

**Results**: Out of 83 patients with OUD, 34 received buprenorphine. Most were non‐Hispanic White (91%), multiparous (77%), and had vaginal deliveries (71%). NOWS was diagnosed in 74% (*n* = 25) of neonates, and 27% (*n* = 9) had severe NOWS. Maternal buprenorphine dose moderately correlated with cord buprenorphine levels (*r* = 0.39, *p* = 0.03), but not with cord norbuprenorphine levels (*r* = 0.10, *p* = 0.59). Cord drug levels did not differ between severe and non‐severe NOWS cases and were not correlated with Finnegan scores.

**Conclusion**: Maternal buprenorphine dose was moderately associated with cord buprenorphine levels but not with norbuprenorphine. Neither cord buprenorphine nor norbuprenorphine levels were linked to NOWS severity. Other factors may influence NOWS beyond umbilical cord drug concentrations.

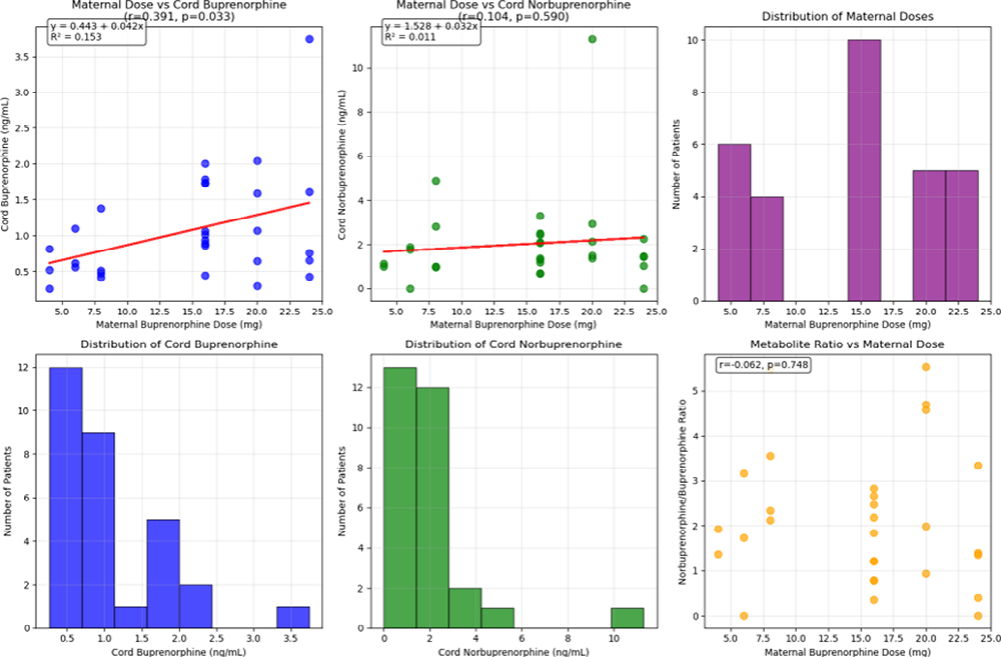

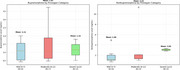

**3:30 PM – 5:00 PM**

## 468 | **Eclampsia** Recurrence **in the Modern Era: A 10‐Year National Cohort Study**

Dana Baraki^1^; Marisa R. Imbroane^2^; Ahmed Ahmed^3^; Amol Malshe^3^

^1^Cleveland Clinic, Cleveland Clinic/Cleveland, OH; ^2^Case Western Reserve University School of Medicine, Case Western Reserve University School of Medicine/Cleveland, OH; ^3^Cleveland Clinic Foundation, Cleveland Clinic Foundation, OH

**Objective**: To determine the recurrence risk of eclampsia in subsequent pregnancies. While the recurrence of preeclampsia is well‐documented, there is limited data regarding the recurrence of eclampsia. Understanding this risk is critical for counseling and management of affected patients.

**Study Design**: This retrospective cohort study utilized the TriNetX research network which includes data from a diverse group of national healthcare organizations. Patients with a diagnosis of eclampsia between 2015 and June 2025 and at least one subsequent pregnancy were included. A total of 12,362 patients met inclusion criteria. Recurrence of eclampsia was assessed and stratified by the number of subsequent episodes. Patients were also screened for development of preeclampsia and HELLP syndrome in future pregnancies.

**Results**: Of the 12,362 patients with an index pregnancy complicated by eclampsia and a subsequent pregnancy, 2641 (21.4%) experienced recurrent eclampsia. Additionally, 886 (7.2%) had three episodes, and 1102 (8.9%) had four or more episodes. In total, 4629 patients (37.4%) experienced recurrent eclampsia. Among the cohort, 6983 patients had no prior diagnosis of preeclampsia; of these, 2298 (32.9%) developed preeclampsia in a subsequent pregnancy. Further, 1016 (14.5%) experienced preeclampsia in two subsequent pregnancies, 471 (6.7%) in three, and 761 (10.9%) in four or more pregnancies. Overall, 4546 patients (65%) developed preeclampsia in at least one future pregnancy. Of 13,359 patients without a prior diagnosis of HELLP syndrome before the index eclampsia event, 718 (5.4%) developed HELLP in one subsequent pregnancy, 378 (2.8%) in two, 175 (1.3%) in three, and 252 (1.9%) in four or more subsequent pregnancies.

**Conclusion**: Recurrent eclampsia occurred in over one‐third of patients with a prior diagnosis. Additionally, two‐thirds experienced preeclampsia and nearly 10% developed recurrent HELLP syndrome in subsequent pregnancies. These findings highlight the need for anticipatory counseling, risk stratification, and close surveillance in patients with a history of eclampsia.

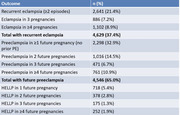

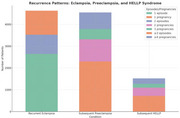

**3:30 PM – 5:00 PM**

## 469 | **Identifying** Culture**‐Positive Intrapartum Fever: Risk Factors from a Retrospective Cohort and CHAID Analysis**

Daniel Gabbai^1^; Itamar Gilboa^1^; Roza Berkovitz Shperling^2^; Itai Atar^3^; Dana Englander^1^; Yariv Yogev^4^; Emmanuel Attali^1^

^1^Lis Hospital for Women's Health, Tel Aviv Sourasky Medical Center, Lis Hospital for Women's Health, Tel Aviv Sourasky Medical Center, Tel Aviv; ^2^Lis Hospital for Women's Health, Tel Aviv Sourasky Medical Center, Tel Aviv, Tel Aviv; ^3^Lis Hospital for Women's Health, Tel Aviv Sourasky Medical Center, Tel Aviv, Israel, Tel Aviv, Tel Aviv; ^4^Lis Hospital for Women's Health, Tel Aviv Sourasky Medical Center Gray Faculty of Medicine, Tel Aviv University, Israel, Lis Hospital for Women's Health, Tel Aviv Sourasky Medical Center, Tel Aviv

**Objective**: To identify clinical and obstetric factors associated with positive bacterial cultures among women with intrapartum fever, and to develop a hierarchical risk stratification model.

**Study Design**: A retrospective cohort study included all deliveries (2011–2024) at a single, university affiliated, tertiary medical center (∼13,000 annual deliveries). Women with intrapartum fever (maternal temperature of ≥38.0°C (100.4°F) during labor) were categorized based on bacterial culture results. Cultures were obtained from blood, urine, and placenta. Univariate analysis was followed by multivariable logistic regression. Moreover, a CHAID (Chi‐squared Automatic Interaction Detector) decision tree was constructed using only variables that remained significant in the multivariable model. Women with intrapartum fever who had missing data regarding bacterial culture sample were excluded from study.

**Results**: 1. During the study period, 2845/146,999 (3.9%) women had intrapartum fever, with 352 (12.4%) yielding at least one positive culture: blood (39%), placenta (37.5%), and urine (23.4%).

2. In the univariate analysis, positive cultures were associated with older maternal age, earlier gestational age, lower gestational weight gain, nulliparity, IVF, multiple gestation, cesarean delivery and maternal fever >39°C (*p* < 0.05).

3. In the multivariable logistic regression model, preterm delivery (OR = 2.47, 95% CI 1.51–4.05, *p* < 0.001), IVF (OR = 1.71, 95% CI 1.16–2.5, *p* = 0.007), multiple gestation (OR = 2.68, 95% CI 1.4–5.1, *p* = 0.003), and maternal fever >39°C (OR = 1.67, 95% CI 1.11–2.51, *p* = 0.013), were found to be independent risk factors for the study outcome.

4. In the CHAID model, preterm birth was the strongest predictor of positive cultures (*p* < 0.001). Among preterm births, age >35 and nulliparity further increased risk. In term births, fever >39°C and weight gain >12 kg were linked to higher positivity rate.

**Conclusion**: Positive cultures in intrapartum fever are associated with identifiable clinical risk factors. The CHAID decision tree offers a practical tool for infection risk stratification and targeted management.

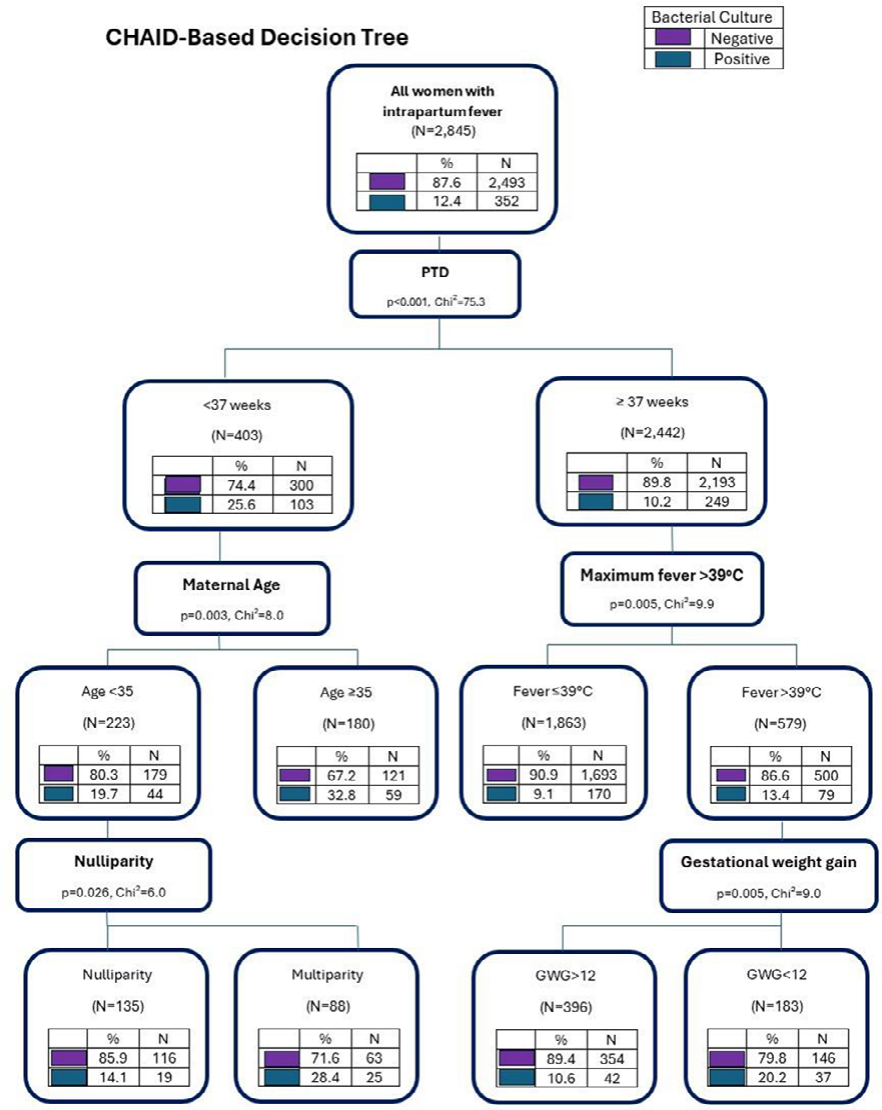

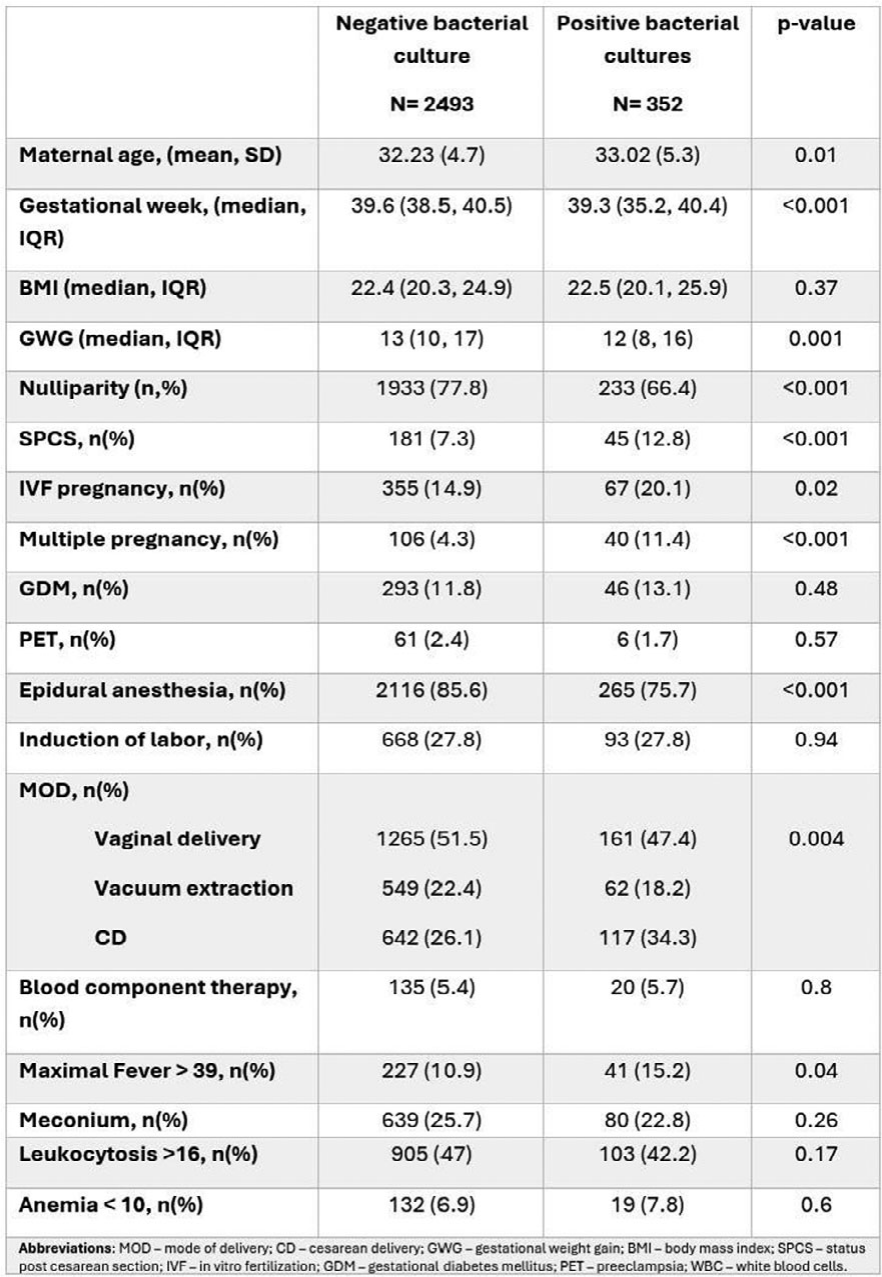

**3:30 PM – 5:00 PM**

## 470 | **Factors** Associated **With Uptake of Prenatal Repair for Fetal Myelomeningocele**

Daniel Liesman^1^; Steven T. Papastefan^2^; Manmeet Singh^3^; Aimen Shaaban^4^; Ashish Premkumar^5^

^1^Ann & Robert H. Lurie Children's Hospital of Chicago, Ann and robert h. lurie children, IL; ^2^Northwestern University Feinberg School of Medicine, Northwestern Memorial Hospital, IL; ^3^The Chicago Institute for Fetal Health, The Chicago Institute for Fetal Health, IL; ^4^The Chicago Institute for Fetal Health (CIFH), Chicago, IL; ^5^University of Chicago, Chicago, IL

**Objective**: Single‐center data from a high‐volume fetal care center (FCC) demonstrate 71% of patients eligible for prenatal fetal myelomeningocele repair (fMMC) ultimately proceed with surgery. However, little is known about the sociodemographic and biomedical factors associated with electing for prenatal fMMC repair.

**Study Design**: We conducted a retrospective cohort study of patients offered prenatal repair for fMMC at an FCC in Illinois between 2017–2025. All included patients were offered prenatal repair based on the criteria established by the Management of Myelomeningocele Study, except for a body mass index cutoff of 40 kg/m^2^, rather than 35 kg/m2. Our center began offering fetoscopic repair in 2020. The primary outcome was uptake of prenatal repair. Unadjusted odds ratios were generated. Significance was set at an alpha of 0.05.

**Results**: Among 107 patients offered prenatal repair, 92 (86%) chose prenatal repair and 15 (14%) chose postnatal repair. Maternal age and gestational age at initial consultation did not differ between groups. Non‐White race, LatinX ethnicity, nulliparity, route of surgery, and governmental payor insurance were not associated with prenatal or postnatal repair choice (Table 1). However, in‐state residence and the presence of talipes equinovarus were associated with a lower likelihood of choosing prenatal repair (OR 0.24; *p*‐value = 0.03; OR 0.29, *p*‐value = 0.03) (Table 2).

**Conclusion**: A majority of patients elected for prenatal repair of fMMC, at a frequency higher than previously reported in the literature. In‐state residents were more likely to opt for postnatal repair despite their proximity to the FCC suggesting that factors beyond geographic access, such as regional referral patterns, inter‐state cultural differences, or patient propensity to seek specialized care, may influence fMMC repair decision‐making. Furthermore, the presence of talipes equinovarus was associated with decreased uptake of prenatal repair, which likely reflects team counseling about the limited benefits of prenatal fMMC repair when motor function is impaired.

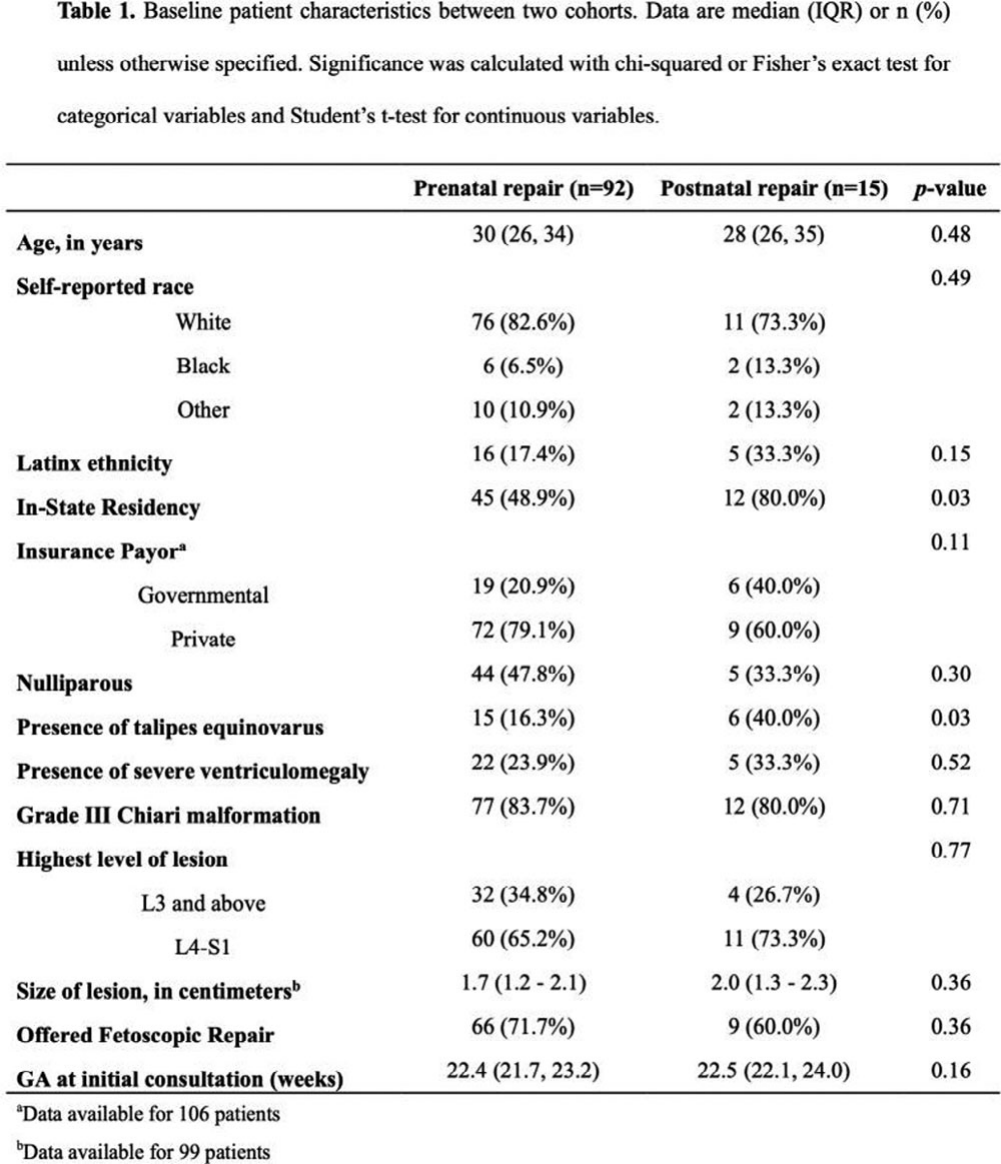

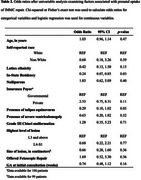

**3:30 PM – 5:00 PM**

## 471 | **Disparities in Low Dose Aspirin Prescription Among Patients at Increased Risk for Preeclampsia**

Daniella Rogerson^1^; Carolina Thorlund‐Díaz^2^; Marni B. Jacobs^3^; Lillian Blank^4^; Leilani Gutierrez‐Palominos^2^; Ayelet Ruppin‐Pham^2^; Maryam Tarsa^3^; E. Nicole Nicole Teal^3^

^1^University of California San Diego Health, San Diego, CA; ^2^University of California San Diego, University of California San Diego, CA; ^3^Division of Maternal Fetal Medicine, Department of Obstetrics, Gynecology and Reproductive Sciences, University of California San Diego, San Diego, CA; ^4^University of California San Diego, San Diego, CA

**Objective**: Low dose aspirin (LDA) reduces preeclampsia (preE) risk, yet prescribing rates among eligible pregnant patients remain low. This study aims to determine whether there are disparities in LDA prescription to eligible patients based on race/ethnicity, primary language, or insurance after implementation of a standardized electronic health record (EHR) preE risk screening tool.

**Study Design**: This retrospective cohort study included all patients ≥ age 18 with births at a single academic center from 9/1/22 to 7/15/25 who had 1 high [multifetal gestation, chronic hypertension (CHTN), diabetes mellitus (DM), renal disease, lupus] or ≥2 moderate (nulliparity, obesity, age ≥35) preE risk factors documented via screening tool or ICD10 code. The primary outcome was LDA recommendation/prescription rate, determined by recommendation documentation in the risk screening tool or prescription in the EHR medication log. Data were extracted from Epic Clinical Data Warehouse. Chi‐square or t‐tests were used to compare LDA prescribing rates by race/ethnicity, primary language, and insurance, and multivariable regression models were used.

**Results**: Of 5737 LDA eligible patients, 3995 (69.6%) were recommended/prescribed LDA and 1,7442 (30.4%) were not. Patients recommended/prescribed LDA were more likely to be older, multiparous, English speaking, and privately insured and/or have DM or CHTN (Table 1). In a multivariable analysis, Black/African American patients had higher odds of being prescribed/recommended LDA (aOR 1.80, 95% CI 1.36–2.39) while non‐English speaking and publicly insured patients had lower odds (Spanish aOR 0.60, 95% CI 0.46–0.78; other language aOR 0.62, 95% CI 0.45–0.85; public insurance aOR 0.80, 95% CI 0.69–0.93) (Table 2).

**Conclusion**: After implementation of a standardized preE risk screening tool, LDA was more often prescribed to eligible Black patients than White, likely reflecting ACOG's inclusion of Black race/racism as a preE risk factor. However, LDA was less often prescribed to publicly insured or non‐English speaking patients, which highlights an opportunity for quality and equity improvement.

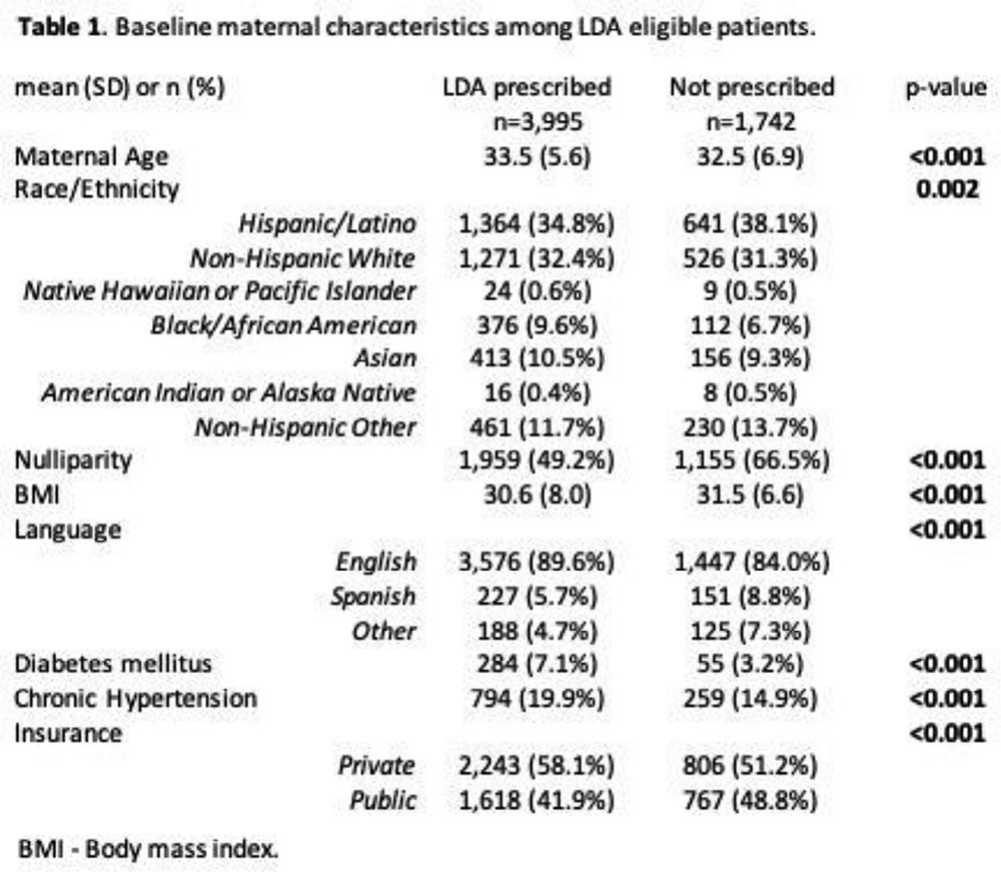

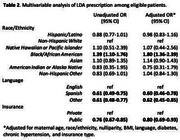

**3:30 PM – 5:00 PM**

## 472 | **Maternal** Transocular **Ultrasound for Prediction of Hypertensive Disorders of Pregnancy**

Danielle L. Chirumbole^1^; Ellen Tillery^1^; Lauren Taylor^1^; Lauren Greenwood^1^; Sarah Cronin^1^; Alexandra H. Latham^2^; Rebecca M. Johnson^1^; Avni Kapadia^1^; Benjamin Frankfort^1^; Karin A. Fox^3^; Llewellyn Padayachy^4^; Michael A. Belfort^1^

^1^Baylor College of Medicine, Houston, TX; ^2^Baylor College of Medicine, Baylor College of Medicine, TX; ^3^Division of Maternal Fetal Medicine, The University of Texas Medical Branch Health, Galveston, TX; ^4^University of Pretoria, Pretoria, Gauteng

**Objective**: Increased optic nerve sheath diameter (ONSD), as measured by transocular ultrasound, correlates with intracranial pressure in hypertensive non‐pregnant and pregnant patients. We aimed to determine whether increased ONSD in normotensive gravidas in the 2^nd^ trimester can predict subsequent development of hypertensive disorders (ACOG criteria).

**Study Design**: Prospective cohort study (IRB#H‐53999) of gravidas undergoing prenatal care/obstetric ultrasound and delivered at an academic institution (2/2024–6/2025). Gravidas with preexisting chronic hypertension, maternal ocular anomalies, fetal growth restriction (FGR) and fetal anomalies were excluded. A handheld 3–12 MHz linear array ultrasound transducer (V‐scan, GE) was used to obtain 10‐second cine clips and still images of both eyes in the transverse plane during the 2^nd^ trimester. ONSD was measured 3mm behind the papilla at two different time points in each clip. One measurement was made for each still image. ONSD for both eyes were averaged to determine mean ONSD for each patient. The Shapiro‐Wilk test and appropriate parametric/non‐parametric tests for continuous variables and proportions were used. ROC curve used to test diagnostic accuracy. *p* < 0.05 denoted significance.

**Results**: Table 1 shows demographic data. 34 patients were included after 9 were excluded for: new diagnosis of glaucoma (*n* = 1), chronic hypertension (*n* = 5), and FGR (*n* = 3). 8/34 (24%) developed a hypertensive disorder of pregnancy (HTN group) per ACOG criteria 18.3 ± 0.7 weeks after measurement. Mean ONSD in the 2nd trimester was significantly higher in the HTN group compared to the normotensive (Control) group: 6.04 ± 0.24 (*n* = 8) vs 5.47 ± 0.13 mm (*n* = 26), *p* = 0.04. 50% of the HTN group had ONSD >6.1 vs 14.8% of Controls, *p* = 0.06. ROC analysis demonstrated an AUC of 0.745 with a PPV of 0.5 (95% CI 0.15–0.85) and NPV of 0.85 (95% CI 0.71–0.98).

**Conclusion**: ONSD in the second trimester may be an easily applied, non‐invasive, inexpensive predictor of the subsequent development of hypertensive disorders of pregnancy though is likely most useful in ruling out hypertension if normal.

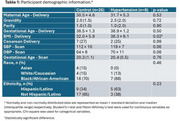

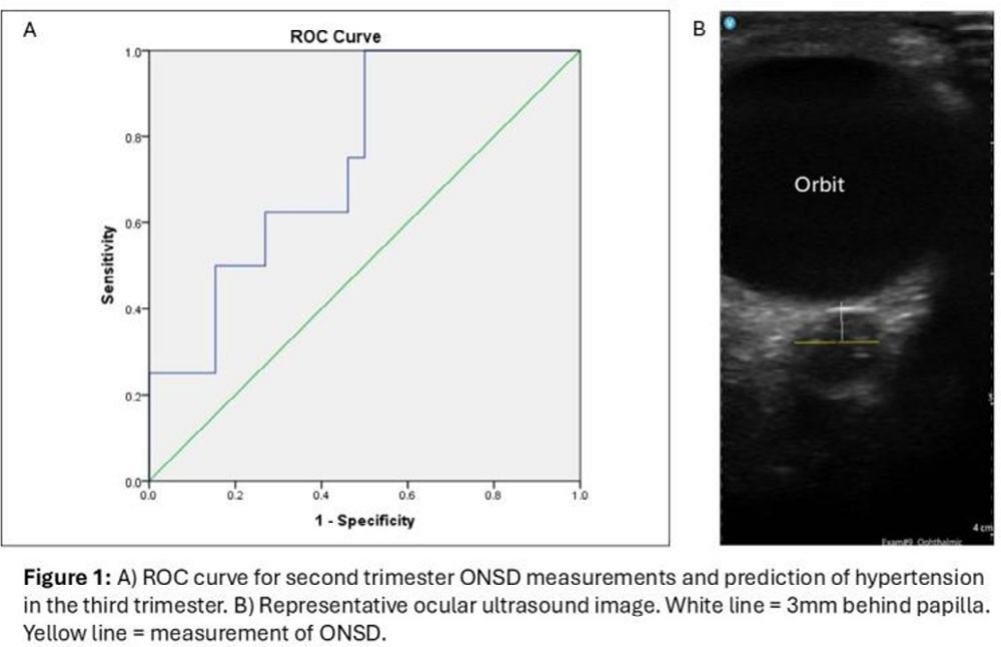

**3:30 PM – 5:00 PM**

## 473 | **Effect of Pre‐**Conception **Bariatric Surgery on Attention‐Deficit Hyperactivity Disorder: Role of Child's Sex and Race/Ethnicity**

Darios Getahun^1^; Michael J. Fassett^2^; Theresa M. Im^1^; Vicki Y. Chiu^1^; Morgan R. Peltier^3^

^1^Department of Research & Evaluation, Kaiser Permanente Southern California, Pasadena, CA; ^2^Department of Obstetrics and Gynecology, Kaiser Permanente West Los Angeles Medical Center, Los Angeles, CA; ^3^Psychiatry and Behavioral Health, Jersey Shore University Medical Center, Neptune City, NJ

**Objective**: Although bariatric surgery (BS) may deprive the fetus of essential nutrients, affecting growth and development, its link to attention deficit hyperactivity disorder (ADHD) risk has received limited attention. We investigated whether preconception BS increases ADHD risk and how gestational age, child's sex, and race/ethnicity may modify this association.

**Study Design**: We conducted a retrospective cohort study of children born between 2011–2022 at Kaiser Permanente Southern California (KPSC) hospitals. BS data were obtained from the KPSC Bariatric Surgery Registry (*n* = 17,254). ADHD diagnoses were made by neurodevelopmental specialists using standardized protocols and validated against gold standards. We assessed the modifying effects of BS type, child's sex, race/ethnicity, and gestational age. Associations between BS and ADHD were evaluated using Cox proportional hazards models, reported as incidence rates and adjusted hazard ratios (aHRs) with 95% confidence intervals (CIs).

**Results**: Children of mothers with preconception BS had higher ASD rates than those without BS (8.3% vs. 6.1%; aHR = 1.52, CI: 1.22–1.90). The association was significant for pregnancies conceived <1 year (aHR 1.59, CI: 1.16–2.17), 1–2 years (aHR 1.48, CI: 1.11–1.97), and ≥2 years (aHR 1.33, CI: 1.08–1.63) post‐surgery. It was significant among Hispanic (aHR 1.67, CI: 1.24–2.27), but not for non‐Hispanic groups, and for Roux‐en‐Y gastric bypass (aHR 1.43, CI: 1.12–1.82) and sleeve (aHR 1.48, CI: 1.20–1.82) procedures. Stratified analyses showed higher ADHD risk in children of mothers with post‐surgery body mass index (BMI) 35–39.9 kg/m^2^, compared to BMI‐matched women without BS. The BS ‐ ADHD link was consistent across race/ethnicity and sex.

**Conclusion**: Preconception BS increases the risk of ADHD in offspring, regardless of the interval between surgery and pregnancy, as well as the child's sex and race/ethnicity.

**3:30 PM – 5:00 PM**

## 474 | **Reducing** Postpartum **Hemorrhage and Blood Transfusion Rates Through Standardized Anemia Management: A Quality Improvement Initiative**

Delphina Maldonado^1^; Marie Russelman^1^; Rosanne Vertichio^1^; Emmanuella Kobara^1^; Katherine Walter^1^; Genevieve Sicuranza^1^; Anju Suhag^2^

^1^NYU Grossman Long Island School of Medicine, Mineola, NY; ^2^NYU Langone Long Island, Mineola, NY

**Objective**: Iron deficiency anemia (IDA) is common in pregnancy due to increased iron demands and is linked to postpartum hemorrhage (PPH), transfusion, and neonatal complications. This quality improvement (QI) initiative aimed to reduce PPH and transfusion through standardized antenatal anemia management— including ferritin screening, oral/IV iron therapy, and structured education—at a single academic center.

**Study Design**: This single‐site interdisciplinary QI project was implemented at two NYU Langone Hospital—Long Island (NYUG‐LI) outpatient practices, targeting pregnant patients with hemoglobin <11 g/dL in the third trimester. The intervention included ferritin screening at 24–32 weeks, oral or IV iron supplementation, and structured provider‐patient education. Outcomes—admission anemia (Hgb <10 g/dL), PPH (≥ 500 mL vaginal or ≥ 1000 mL cesarean), and transfusion—were compared between focus patients receiving protocol‐based care and all other NYUG‐LI deliveries from July 2024 to June 2025. Stratified analysis by race/ethnicity explored potential disparities.

**Results**: Among 905 focus site deliveries, 54 patients received standardized anemia management. Compared to 4791 total NYUG‐LI deliveries, focus patients had lower rates of anemia (4.4% vs. 9.2%), PPH (0.8% vs. 1.0%), and transfusion (0.7% vs. 1.1%). Figure 1 shows favorable trends over time. Table 1 highlights improved outcomes across racial/ethnic groups, with notable benefit among Black and Hispanic patients.

**Conclusion**: Standardized antenatal anemia management was associated with improved maternal outcomes and may help reduce disparities in obstetric complications. Future efforts will focus on expanding the protocol across additional clinical sites, enhancing adherence to IV iron guidelines, and engaging community partners to ensure equitable implementation and sustained impact.

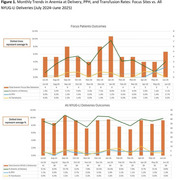

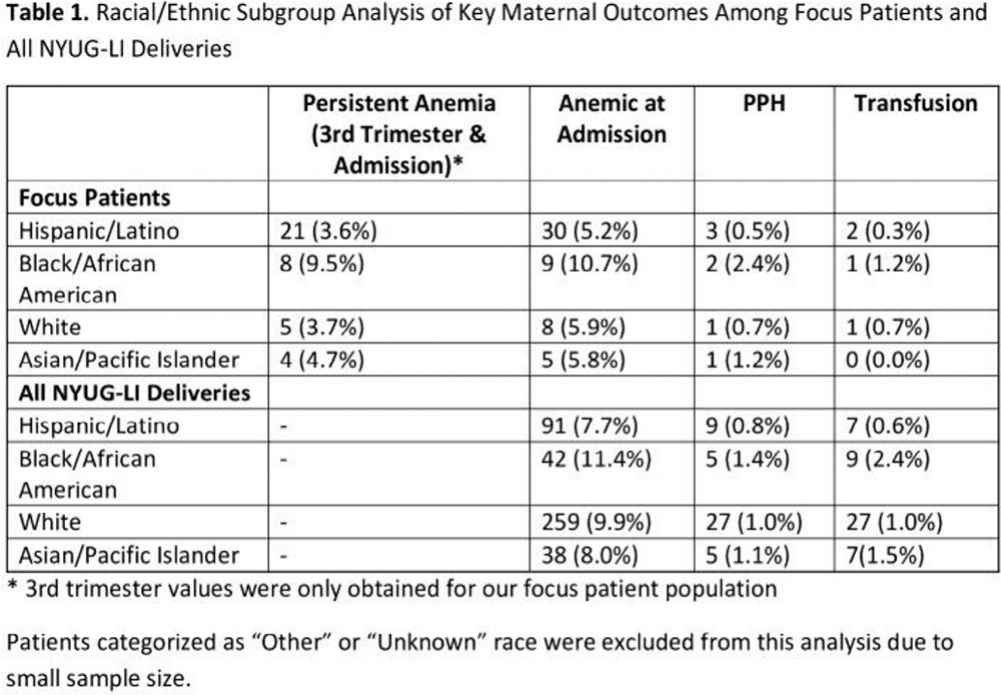

**3:30 PM – 5:00 PM**

## 475 | **The Association** Between **Premature Antenatal Corticosteroid Exposure and Long‐Term Respiratory Morbidity in Offspring**

Dor Marciano^1^; Gali Pariente^2^; Tamar Wainstock^3^; Eyal Sheiner^4^

^1^Soroka university medical center, Soroka, HaDarom; ^2^Department of Obstetrics and Gynecology, Soroka University Medical Center, Ben‐Gurion University, beer sheva, HaDarom; ^3^Ben‐Gurion University of the Negev, Beer Sheva, HaDarom; ^4^Soroka University Medical Center, Faculty of Health Sciences, Ben‐Gurion University of the Negev, beer sheva, HaDarom

**Objective**: Antenatal corticosteroids (ACS) are routinely administered to mothers at risk for preterm delivery to reduce short‐term neonatal complications. While their immediate benefits are well‐established, less is known about long‐term respiratory outcomes. This study aimed to evaluate the association between ACS exposure before 34 weeks, and long‐term respiratory morbidity among preterm‐born offspring (<37 weeks), adjusting for gestational age at birth.

**Study Design**: A retrospective population‐based cohort study of all singleton preterm births at a tertiary medical center was conducted. Offspring were classified by ACS exposure before 34 gestational weeks. Respiratory‐related community and hospital diagnoses were tracked from birth to 18 years. Incidence rates were calculated per 1000 person‐years. Kaplan–Meier curves compared cumulative respiratory morbidity. A Cox proportional hazards model estimated adjusted hazard ratios, controlling for gestational age at birth, maternal age, diabetes mellitus, pregnancy–related hypertensive disorders, and cesarean delivery.

**Results**: Among 13,580 infants, 1538 (11.3%) were ACS‐exposed. These infants had lower mean gestational age (32.6 vs. 34.2 weeks; *p* < 0.001) and higher rates of cesarean delivery and hypertensive disorders. Respiratory morbidity incidence was higher in the exposed group (161.8 vs. 68.8 per 1000 person‐years; *p* < 0.001, Table), including asthma and obstructive sleep apnea. Kaplan–Meier analysis showed significantly higher cumulative incidence in the exposed group (*p* < 0.001; the strongest association was noted in infants born <28 weeks (*p* < 0.001, Figure)). In a Cox model, ACS exposure remained independently associated with long‐term respiratory morbidity (aHR = 1.1; 95% CI, 1.03–1.13; *p* < 0.001).

**Conclusion**: ACS exposure before 34 weeks is associated with increased long‐term respiratory morbidity in preterm offspring, particularly among those born before 28 weeks. These findings underscore the importance of long‐term follow‐up in ACS‐exposed children.

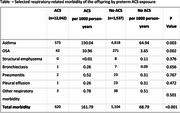

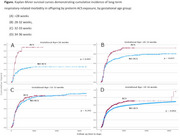

**3:30 PM – 5:00 PM**

## 476 | **Enlarged** Cardiothoracic **Ratio Detection for Ebstein Anomaly Detection using Artificial Intelligence**

Edgar A. Hernandez‐Andrade^1^; Nathan S. Fox^2^; Andrei Rebarber^3^; Jennifer Lam‐Rachlin^4^; Sara Garmel^5^; Nazbanou Heim^6^; Anna‐Maria Coltri^7^; Roger Bessis^6^; Mia Ratsimandresy^8^; Philippe Boukobza^9^; Malo de Boisredon^10^; Eric Askinazi^10^; Valentin Thorey^10^; Christophe Gardella^10^; Bertrand Stos^11^; Marilyne Levy^11^

^1^Division of Maternal‐Fetal Medicine, Department of Obstetrics, Gynecology and Reproductive Sciences, McGovern Medical School at UTHealth Houston, Houston, TX; ^2^Icahn School of Medicine, Mount Sinai West, New York, NY; ^3^Icahn School of Medicine, Mount Sinai West, Mount Sinai West, NY; ^4^Icahn School of Medicine at Mount Sinai, Icahn School of Medicine at Mount Sinai, NY; ^5^Michigan Perinatal Associates and Corewell Health, Dearborn, MI; ^6^Centre d'Echographie de l'Odéon, Paris, Ile‐de‐France; ^7^CHU de Nice, Nice, Provence‐Alpes‐Cote d'Azur; ^8^Clinique Pasteur, Toulouse, Midi‐Pyrenees; ^9^CEDEF ‐ Centre Européen de Diagnostic et d'Exploration de la Femme, Le Chesnay, Ile‐de‐France; ^10^BrightHeart, Paris, Ile‐de‐France; ^11^UE3C ‐ Unité d'explorations cardiologiques ‐ Cardiopathies Congénitales, Paris, Ile‐de‐France

**Objective**: Cardiothoracic ratio (CTR) is a biomarker of altered fetal cardiac hemodynamics and may be useful to screen for Ebstein anomaly, a severe tricuspid valve malformation. However, current guidelines only provide general thresholds for CTR in the 2nd and 3rd trimesters.

We aim to (1) create a nomogram of CTR for normal cases throughout pregnancy, (2) establish a gestational age (GA)‐dependent threshold for enlarged CTR, (3) assess its relevance in screening for Ebstein anomaly, and (4) evaluate an AI software in detecting cases with enlarged CTR.

**Study Design**: We evaluated 646 ultrasound exams of singleton pregnancies at the 2nd and 3rd trimester without cardiac abnormalities, with interpretable 4CH view, from 7 prenatal screening centers. Detailed area‐based CTR were manually measured. A nomogram for CTR of normal cases was developed, and a GA‐dependent threshold was defined as the 99th percentile (Figure 1).

We evaluated this threshold on 59 fetal echocardiograms, including 41 with severe Ebstein anomaly (severe tricuspid regurgitation, functional pulmonary atresia, non functional right ventricle or hydrops) and 18 with mild variants.

We then evaluated an AI software developed by BrightHeart to detect cases above the CTR threshold, based on an automated analysis of all grayscale cines in an exam.

**Results**: Among the 646 normal exams, 5 cases had CTR above threshold (99.2% specificity, 95% CI: 98.2–99.7). CTR was above threshold in 46 of 59 exams with Ebstein anomaly, corresponding to a sensitivity of 78.0% (95% CI: 65.9–86.6) (92.7% (95% CI: 80.6–97.5) and 44.4% (95% CI: 24.6–66.3) for severe and mild cases, respectively).

In the 481 cases with a 4CH view in a cine, the AI had 100% (95% CI: 92.7–100) sensitivity to detect enlarged CTR and 97.9% (95% CI: 96.1–98.9). Results were consistent across subgroups of GA (Figure 2).

**Conclusion**: AI can accurately estimate cases with enlarged CTR, which may be an effective screening tool for Ebstein anomaly, particularly when assessed among additional markers. Such screening is especially impactful for severe cases, which typically warrant immediate post‐delivery evaluation.

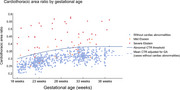

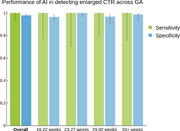

**3:30 PM – 5:00 PM**

## 477 | **Local Growth** Curves **Overestimate Fetal Size and May Miss Pathologic Growth Restriction**

Eliel Kedar Sade^1^; Tamar Shyldkrot^2^; Ohad Mizrachi^2^; Letizia Schreiber^2^; Eran Weiner^3^; Ilia Kleiner^3^; Lior Kashani Ligumsky^2^

^1^Wolfson Medical Center, Department of Obstetrics and Gynecology, Wolfson Medical Center, Tel Aviv; ^2^Wolfson Medical Center, Department of Obstetrics and Gynecology, Wolfson Medical Center, HaMerkaz; ^3^Wolfson Medical Center, Department of Obstetrics and Gynecology, Holon, HaMerkaz

**Objective**: Population‐based fetal growth curves are expected to improve the detection of pathologic fetal growth restriction (FGR). We compared a local curve (Spiegel‐Yagel, SY) and a universal curve (Hadlock, HL) to evaluate differences in overestimation rates and placental lesion prevalence among small‐for‐gestational‐age (SGA) neonates in a retrospective cohort study.

**Study Design**: We retrospectively analyzed 346 singleton pregnancies (2016–2024) with birthweight (BW) <10th percentile. Group 1: BW <3rd percentile (*n* = 243); Group 2: BW 3–10th percentile (*n* = 103). Estimated fetal weight (EFW) percentiles were calculated using SY and HL curves. Overestimation was defined as EFW 3–10% despite BW <3% or EFW >10% despite BW 3–10%. Placental pathology was reviewed per Amsterdam Consensus (2016) and classified as:

- Maternal vascular malperfusion (MVM): distal villous hypoplasia, accelerated villous maturation, decidual arteriopathy, infarction, syncytial knots, villous agglutination.
- Fetal vascular malperfusion (FVM): avascular villi, stromal‐vascular karyorrhexis, thrombosis, villous mineralization.

Lesion prevalence was compared between overestimated and correctly classified SGA cases using chi‐square analysis.

**Results**: SY overestimated fetal size more often than HL, especially in BW 3–10% (46.6% vs 21.4%, *p* = 0.006). In BW <3%, rates were 35.8% (SY) vs 28.4% (HL). Placental lesions were frequent in overestimated cases, with MVM in 52–64% and FVM in 28–42%, meaning over half of fetuses classified as non‐SGA still had placental pathology. No significant difference in lesion prevalence was observed between overestimated and correctly classified cases.

**Conclusion**: Local growth curves classified more SGA neonates as non‐SGA compared with a universal standard, missing many with placental malperfusion. Universal standards may better identify pathologic FGR and improve prenatal risk detection

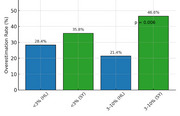

**3:30 PM – 5:00 PM**

## 478 | **Factors** Associated **With Severe Maternal Morbidity Among Patients With Placenta Accreta Spectrum Disorder**

Elizabeth L. Mirsky; Emily A. DeFranco

University of Kentucky, University of Kentucky/Lexington, KY

**Objective**: Although Placenta Accreta Spectrum (PAS) disorder is a recognized risk factor for Severe Maternal Morbidity (SMM), limited evidence exists on which specific risk factors contribute most significantly. This study aimed to identify maternal and pregnancy‐related factors associated with SMM among patients with PAS disorder.

**Study Design**: Population‐based case‐control study querying the National Inpatient Sample (NIS) database for delivery admissions containing an International Classification Diagnosis (ICD) code for PAS from 2016 to 2022. Various demographic, maternal, and pregnancy‐related factors were assessed in those with and without SMM, as defined by the standardized criteria for measuring SMM in PAS disorder (Einerson et al, 2025). Logistic regression estimated the association of each factor with SMM. A receiver operating characteristic (ROC) curve was generated from a model including significant risk factors for SMM. The study population was weighted to approximate the US population.

**Results**: 6370 delivery admissions of patients with PAS were identified, representative of 31,850 in the US during the study period. Of those, 10% experienced SMM. We identified numerous sociodemographic, medical, pregnancy and delivery factors that were significantly associated with increased risk of SMM (Table 1). The factors most robustly associated with SMM, with over 2‐fold increased risk were chronic cardiac or renal disease, bleeding disorder, percreta subtype, chorioamnionitis, stillbirth, and severe preeclampsia. ROC curve generated from the logistic regression model of factors displayed in Figure 1a demonstrated good discriminative performance in predicting SMM, with an AUC of 0.78 (Figure 1b).

**Conclusion**: Our findings demonstrate specific clinical and obstetric factors that reliably predict severe maternal morbidity in pregnancies complicated by placenta accreta. These results underscore the importance of early identification and tailored peripartum management to mitigate maternal risk and provide a foundation for future prospective studies and clinical protocols.

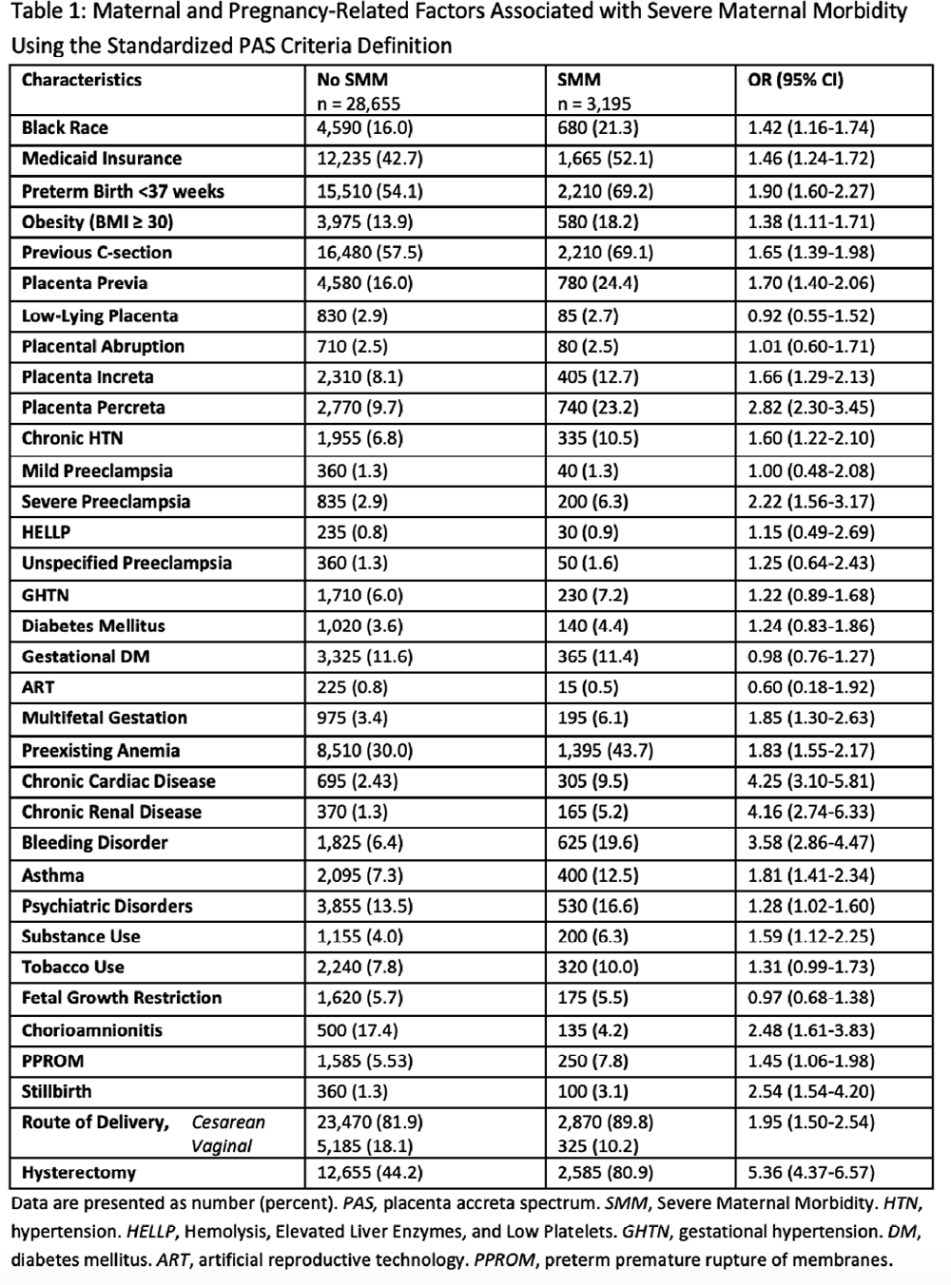

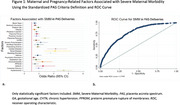

**3:30 PM – 5:00 PM**

## 479 | **Can the Expanded Obstetric Co‐Morbidity Score Predict Intrapartum SMM Using Data Available During Prenatal Care?**

Elliott K. Main^1^; Brielle L. Formanowski^2^; Deirdre Lyell^1^; Katy B. Kozhimannil^3^; Scott A. Lorch^2^; Ciaran S. Phibbs^1^; Molly Passarella^2^; Stephanie A. Leonard^4^

^1^Stanford University School of Medicine, Stanford, CA; ^2^Children's Hospital of Philadelphia, Philadelphia, PA; ^3^University of Minnesota, Minneapolis, MN; ^4^Stanford University, Stanford University, CA

**Objective**: The Expanded Obstetric Comorbidity Score (EOCS) utilizes preexisting medical and obstetric conditions to predict severe maternal morbidity (SMM). We assessed whether a modified EOCS of co‐morbidities limited to those available at mid‐gestation could predict the incidence of SMM at delivery, a prediction that could drive consultations to improve pregnancy outcomes, particularly in low volume and rural settings.

**Study Design**: Linked maternal discharge and vital record data for live‐ and stillbirths (≥20 weeks) from OR, SC (2008–2020) and PA (2008–2018) were analyzed. Modified EOCS was calculated excluding preterm birth, preeclampsia, and abruption. Co‐morbidities and SMM (total and non‐transfusion) were calculated using ICD codes. Modified Poisson regression modelling was used predict SMM rates based on the EOCS, unadjusted and adjusted for age and parity. Established urban‐rural and hospital volume classifications were utilized. Standard NICU levels were used in lieu of maternal levels of care.

**Results**: 1,731,858 records were examined. The population mean for total SMM was 1.4%. Natural cut‐points were observed dividing the population into 5 groups. 79.2% of the population were in the first group (scores of 0–5) and had a baseline SMM rate of 0.7%. The 3^rd^ to 5^th^ groups with scores of 17–24, 25–36, and 37–76 points had adjusted RR for intrapartum SMM of 8.3, 22.3 and 66.0 (Figure 1). Adjustment for maternal age and parity did not alter the results. Similar findings were noted for non‐transfusion SMM. While the majority of the higher risk groups delivered at Level 3 and 4 hospitals, substantial numbers still delivered in Level 1 and 2 and in rural and low‐volume urban hospitals (Table 1).

**Conclusion**: The EOCS could identify prenatal patients at much greater risk for intrapartum SMM within a small percentage of the population. Use of the score could move risk appropriate care to the prenatal period allowing for optimization of care before intrapartum admission. This could be of particular value for obstetric care in small and rural hospitals.

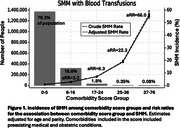

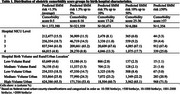

**3:30 PM – 5:00 PM**

## 480 | **Comparing** Adverse **Outcomes of FGR Defined by Abdominal Circumference Versus EFW: A Retrospective Chart Review**

Emily Hahm^1^; Michelle Currie^2^; Ava Karnish^3^; Ankita Kulkani^4^

^1^Cooper University Hospital, Cooper University Hospital, NJ; ^2^The Brooklyn Hospital Center, Brooklyn, NY; ^3^Rowan University at Cooper University Hospital, Camden, NJ; ^4^Cooper University Hospital, Camden, NJ

**Objective**: To compare adverse perinatal outcomes in pregnancies diagnosed with fetal growth restriction (FGR) based on isolated abdominal circumference (AC) versus estimated fetal weight (EFW) <10th percentile.

Limited literature exists on the clinical significance and outcomes of AC‐defined FGR, resulting in variability in practice, particularly regarding surveillance intensity and timing of delivery. This study aims to evaluate our institution's experience to inform decision‐making for pregnancies affected by AC‐defined FGR.

**Study Design**: This was a retrospective chart review of all singleton gestations who underwent growth ultrasounds and delivered at a single health system from 07/02/2021–06/30/2024. The study group comprised of patients with AC <10th percentile and EFW ≥10th percentile. The control group comprised of patients with overall EFW <10th percentile. Data was extracted from electronic medical records. Outcomes evaluated included gestational age at diagnosis and delivery, mode of delivery, maternal and neonatal outcomes. A subgroup analysis was done on perinatal outcomes at delivery by gestational week in the AC group. Data was analyzed using independent T test and Chi‐square tests with significance defined at *p* < 0.05.

**Results**: Of 277 pregnancies, 99 were in the AC group and 178 in the EFW group. The AC group had later gestational age at diagnosis (33.6 vs 31.8 weeks) and delivery (38.2 vs 36.5 weeks). Compared to the EFW group, the AC group had lower rates of preterm delivery (15.2% vs 28.7%), NICU admission (22.2% vs 40.4%), and SGA (60.6% vs 80.9%). Gestational hypertension was more common in the AC group, while preeclampsia was more frequent in the EFW group. In the AC group, an increase in SGA and NICU admissions was observed at 37 weeks compared to other gestational weeks at term.

**Conclusion**: Our findings suggest that isolated AC‐defined FGR is associated with better neonatal outcomes than EFW‐defined FGR, indicating that these two phenotypes may warrant different clinical approaches. Further prospective studies are needed to guide optimal timing of delivery for AC‐defined FGR.

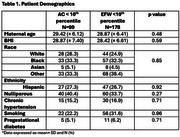

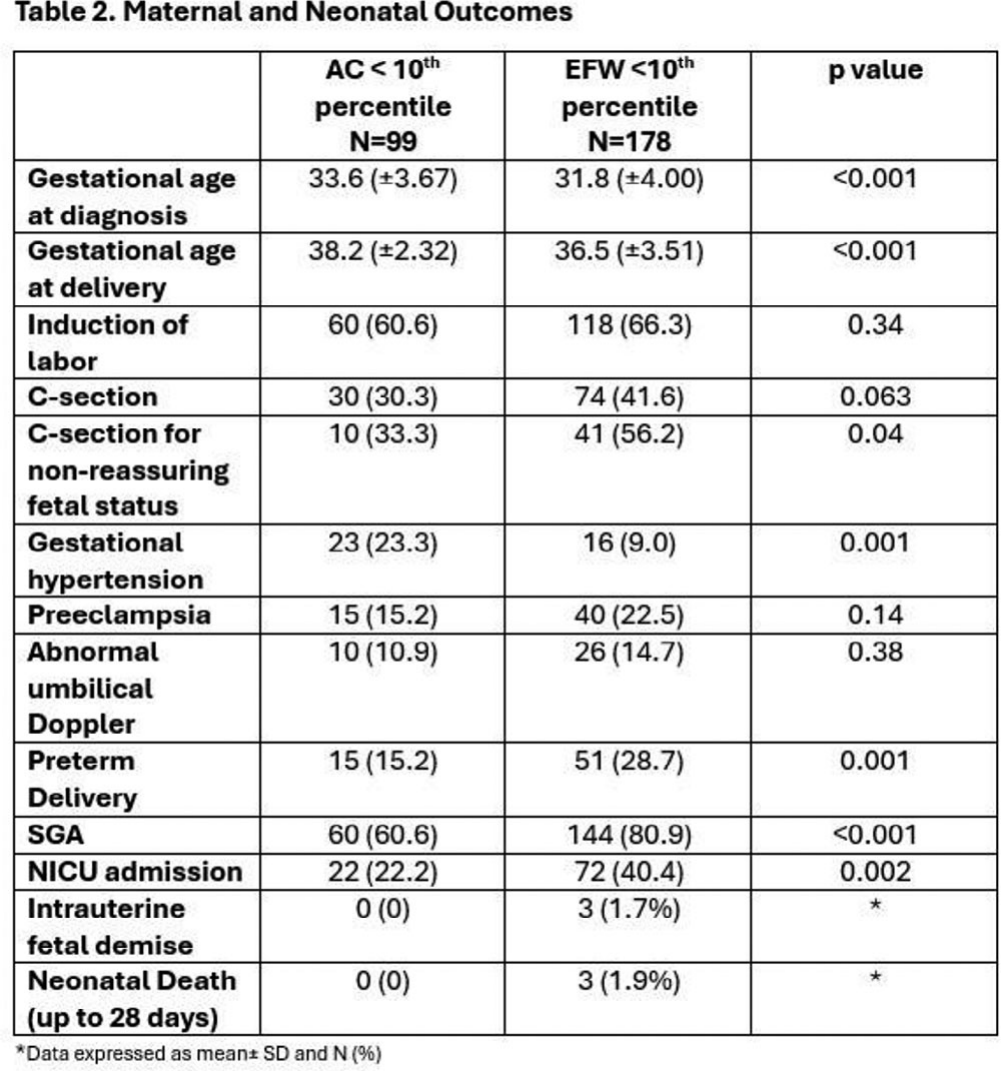

**3:30 PM – 5:00 PM**

## 481 | **Prediction of** Adverse **Neonatal Outcomes by Different Birthweight Standards**

Emily Heideman^1^; Katherine Pressman^2^; Hasan Alhasan^1^; Anthony O. Odibo^3^; Jose R. Duncan^1^

^1^University of South Florida, Tampa, FL; ^2^Department of Obstetrics and Gynecology, University of South Florida, Tampa, Florida, Tampa, FL; ^3^University of Missouri Kansas City, Kansas City, MO

**Objective**: Our objective was to compare the prediction ability for adverse neonatal outcomes of Alexander and Fenton birthweight standards.

**Study Design**: This was a secondary analysis of a previous study that included singleton pregnancies that underwent fetal growth assessment between 26 and 36 weeks of gestation. Exclusion criteria included fetuses with chromosomal or congenital malformations and those without delivery information. The prediction ability of both small for gestational age (SGA) and large for gestational age (LGA) small for gestational age (SGA) by the Fenton and Alexander birthweight charts for a composite of adverse neonatal outcomes (CANO) that included one or more of: neonatal intensive care unit admission, pH <7.1, Apgar score <7 at 5 minutes of life, seizures, respiratory distress syndrome, intraventricular hemorrhage grade III or IV, or neonatal death) was evaluated by comparing the area under the curve of receiver operating curve of clinical characteristics.

**Results**: A total of 1054 neonates were included for analysis, 221 neonates were complicated with CANO, 99 met criteria for SGA and 111 for LGA by the Fenton chart and 139 and 124 by the Alexander standard. For SGA, the Fenton and Alexander standards had similar ability to predict CANO, AUC: 0.524 [0.499 −0.548] vs 0.537 [0.509 −0.565], *p* = 0.16. For LGA, the Fenton standard had a statistically significant better prediction ability for CANO, AUC: 0.534 [0.508 −0.548] vs 0.514 [0.489 −0.540], *p* = 0.026. However, both standards had poor prediction ability for adverse neonatal outcomes in SGA (Figure 1) and LGA neonates (Figure 2).

**Conclusion**: The Fenton birthweight chart may better predictor of adverse neonatal outcomes than the Alexander chart for LGA neonates. Both birthweight standards included in the present study, had poor prediction ability to detect CANO.

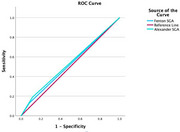

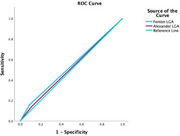

**3:30 PM – 5:00 PM**

## 482 | **The Safety and** Utility **of Attempting an External Cephalic Version After Two Prior Cesarean Deliveries**

Emily Schlussel Markovic; Itamar Futterman; Joselle O'Brien; Nelli Fisher; Tirtza Spiegel Strauss; Rodney A. McLaren, Jr

Maimonides Medical Center, Brooklyn, NY

**Objective**: ACOG considers patients with 2 prior cesarean deliveries (CD) to be candidates for a trial of labor (TOLAC) and separately recommends external cephalic version (ECV) be offered for malpresentation. However, little is known about the safety and utility of ECV among patients with 2 prior CD. This study aimed to evaluate obstetric outcomes after ECV in patients with 2 prior CD to better guide counseling.

**Study Design**: A population study was performed utilizing the US Natality Database from 2018 to 2023 of non‐anomalous, singleton births after 36 weeks gestation in those with 2 prior CD. Adverse maternal and neonatal composite outcomes were compared between those with an ECV attempt and those who had a breech repeat CD (rCD) without ECV attempt and TOLAC. Adverse maternal outcomes included blood transfusion, uterine rupture, unplanned hysterectomy, or ICU admission. Adverse neonatal outcomes included 5‐min Apgar <7, immediate or prolonged assisted ventilation, NICU admission, seizure, or death. ECV success rate and mode of delivery after ECV attempt were also assessed. Univariable and multivariable analyses were performed.

**Results**: Of the 16,293 births that met inclusion criteria, 328 (2%) underwent ECV attempt. Of these ECVs, 223 (68%) were successful. Adverse maternal composite did not differ between births after ECV attempt and those without (1.8% vs. 1.5%, aOR 1.20, 95% CI 0.53–2.71). Those who had an ECV attempt had a lower risk of adverse neonatal composite compared to those who did not (9.5% vs. 18.2%, aOR 0.47, 95% CI 0.32–0.68). Of the 328 ECV attempts, 234 (71.3%) underwent rCD without TOLAC. Of 215 cephalic deliveries, those who had a rCD without TOLAC (*n* = 146) had fewer prior vaginal births than those who had a TOLAC (*n* = 69) (rCD: 1 (1,1) vs 2 (1,3), *p* < 0.001). Of the 94 patients with TOLAC, 52 (55.3%) had a spontaneous vaginal birth, 4 (4.3%) had an operative vaginal birth, and 38 (40.4%) had a rCD.

**Conclusion**: These data suggest that an ECV in patients with 2 prior CD had a similar success rate to the general obstetric population and may be associated with decreased neonatal morbidity.

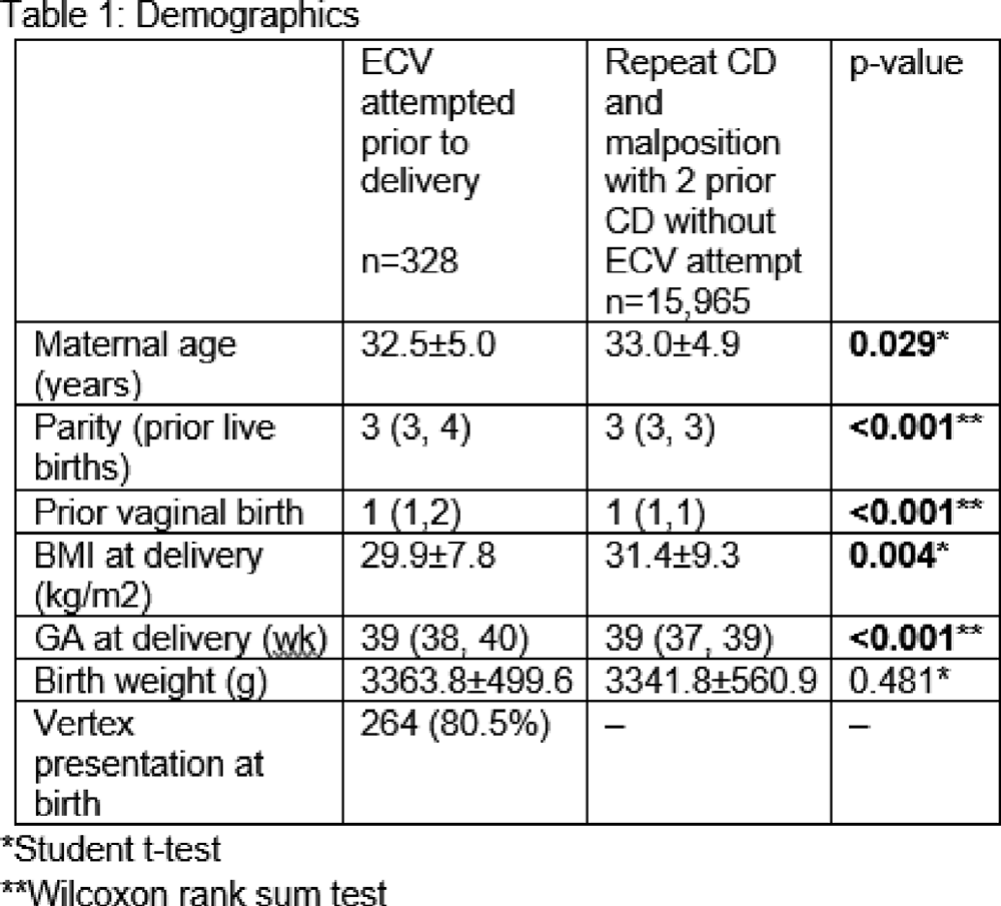

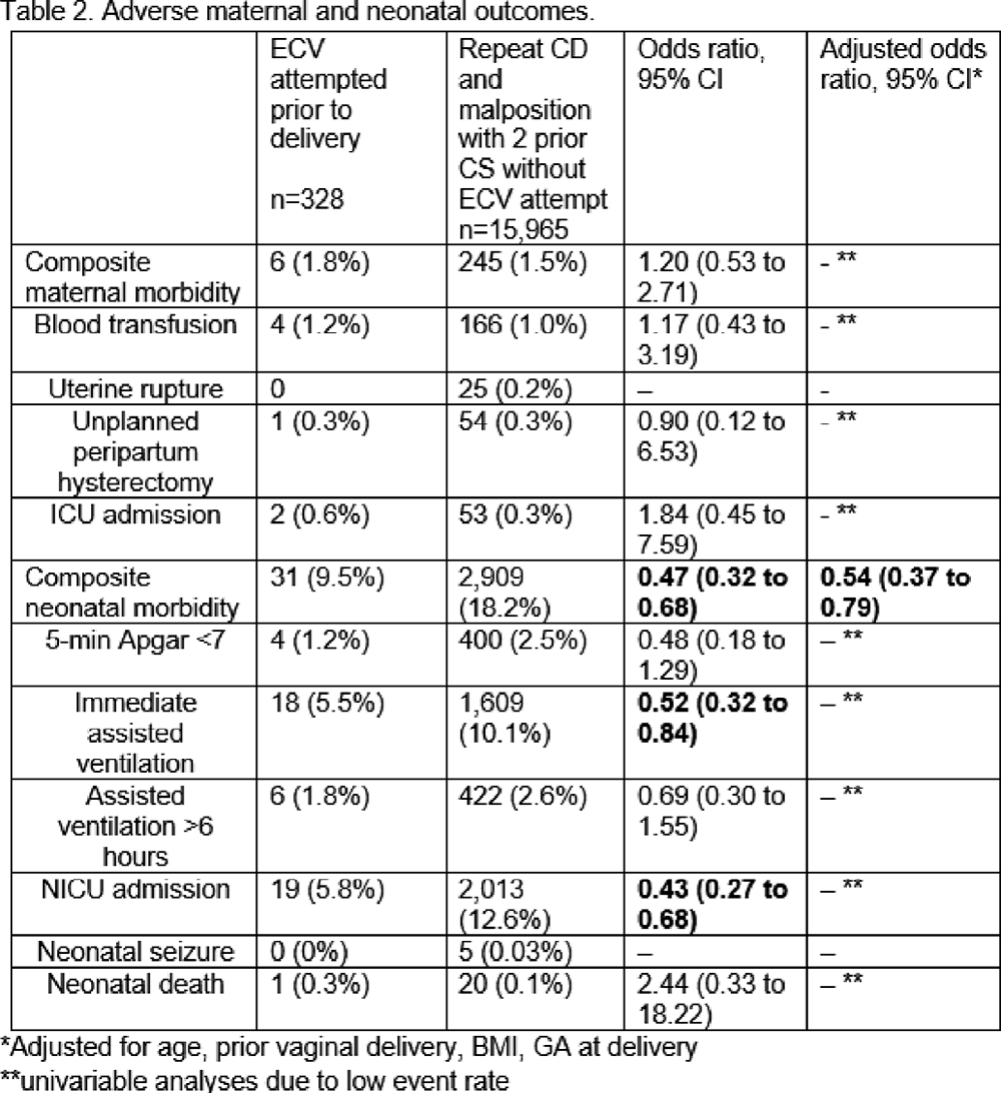

**3:30 PM – 5:00 PM**

## 483 | **The Effect of** Modifiable **Risk Factors on Recurrence of Placental Abruption: A Population‐Based Cohort Study**

Emily B. Rosenfeld^1^; Amanda Tuchler^1^; Rachel Lee^2^; Minxiu Shi^1^; Ruchira Sharma^1^; Cande V. Ananth^2^

^1^Rutgers Robert Wood Johnson Medical School, New Brunswick, NJ; ^2^Rutgers Robert Wood Johnson Medical School, Rutgers Robert Wood Johnson Medical School, NJ

**Objective**: Estimates of abruption recurrence risk originate from outdated studies that may not reflect current risks due to changes in modifiable risk factors and limit the ability to counsel patients about their recurrence risk. The aim of this study is to assess the recurrence risk of patients with placental abruption using a contemporary cohort and to assess the impact of modifiable risk factors on abruption recurrence.

**Study Design**: The study utilizes the Placental Abruption and Cardiovascular Event Risk (PACER) cohort, which includes all births in New Jersey from 1993 to 2020, linked with hospital discharge and mortality records. The cohort was restricted to women with two or more deliveries. Confounder‐adjusted relative risks (aRR) and 95% confidence intervals (CI) were calculated using Poisson regression models to assess the risk of recurrent abruption and joint effects of modifiable risk factors.

**Results**: There were 662,140 women who contributed to at least 1,324,280 deliveries within the cohort, of which 5850 (0.9%) had an abruption in the first delivery and 6287 (0.9%) had an abruption in the second delivery. Of those that had an abruption in the first pregnancy, 209 (3.6%) had recurrent abruption (aRR 3.5, 95% CI 3.4, 3.7). The highest risk of recurrent abruption was seen in those with tobacco use in both pregnancies (RR 6.2, 95% CI 5.7, 6.7), had severe preeclampsia in either pregnancy (RR 5.2, 95% CI 4.6, 5.8), pregestational diabetes in either pregnancy (RR 7.8, 95% CI 7.1, 8.6), and multiples in both pregnancies (RR 22.2, 95% CI 20.9, 23.4) (Table 1). Tobacco users who had an abruption in their first pregnancy but quit smoking before their second pregnancy reduced their risk of abruption in the second pregnancy (RR 0.6, 95% CI 0.2, 1.4) compared to persistent smokers.

**Conclusion**: The overall risk of recurrent abruption is 3.6%, and eliminating modifiable risk factors can further decrease the risk in future pregnancies.

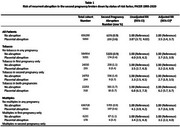

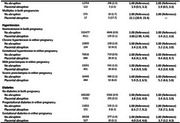

**3:30 PM – 5:00 PM**

## 484 | **Swiss Solutions** to **Pregnant Problems: Evaluating the Ganzoni Formula in Anemic Individuals Receiving IV Iron**

Erik G. Holder^1^; Krystal Li^2^; Julia Kramer^2^; Desiree N. Davis^2^; Raphaela Tchani^2^; Nora M. Doyle^3^

^1^University of Texas Health Science Center at San Antonio, University of Texas Health Science Center at San Antonio, TX; ^2^The Joe R. and Teresa Lozano Long School of Medicine, San Antonio, TX; ^3^Department of Obstetrics and Gynecology, Division of Maternal Fetal Medicine, University of Texas Health Science Center, San Antonio, TX

**Objective**: Iron deficiency anemia (IDA) during pregnancy affects 14–56% of pregnant women globally. In the U.S., IDA affects from 6.9% (1^st^ trimester) ‐ 28.4% (3^rd ^trimester) of pregnancies. Maternal mortality / feto‐maternal morbidity are directly (20%) and indirectly (50%) attributed to IDA. Since 1970, the Ganzoni formula (GF) has been widely used to calculate total iron deficit in other medical specialties but has not yet been applied in pregnancy. We aimed to assess the utility of the GF in pregnant patients with IDA receiving IV iron.

**Study Design**: We conducted a retrospective review of pregnant patients who delivered at our level IV center from 7/2020–7/2025 and received IV iron for IDA. GF was calculated as: body weight (kg) x [target hemoglobin (Hb)–actual Hb (g/dL)] x 2.4 + 500mg (iron stores), with a 2.4 factor derived from assumptions of blood volume (7% of body weight) and Hb iron content of 0.34%. Weight at initial iron transfusion was used. Pre‐ and post‐transfusion Hbs were compared to determine treatment efficacy, with a target Hb of 11g/dL at delivery. Exclusion criteria included hemoglobinopathies. ROC analysis identified an initial GF threshold at which primary outcome was achieved.

**Results**: Of 857 pregnant individuals who received IV iron, 250 delivered > = 6 weeks after initial transfusion (29.2%). Mean index Hb was 8.68 g/dL [range 6.3–12.5] with mean delivery Hb of 11.07 g/dL [range 6.2–14.0]. Patients received an average of 781.3mg [range 200–1600] of IV iron across 1–5 separate, inpatient / outpatient infusions. GF deficits ranged from 372.8–2259.7 mg. ROC analysis identified 1117.28 mg as predictive threshold to achieve target Hb of > = 11 g/dL (*p* = 0.0002), regardless of dose frequency.

**Conclusion**: GF, though previously untested in pregnancy, can be utilized to evaluate iron deficits in patients with known IDA. Patients with a GF deficit of <1117 mg were less likely to achieve a target Hb of 11 g/dL regardless of treatment dosage and frequency. Our findings support the utility of GF in a pregnant population, though further validation is needed.

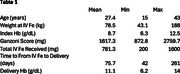

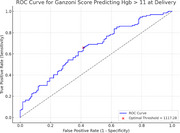

**3:30 PM – 5:00 PM**

## 485 | **Non‐Invasive** Point**‐of‐Care Hemoglobin Resting in an Obstetric Population**

Ethan Litman; Nadiha Chelsea Noor; Shreetoma subrata Datta; Annliz Macharia; Michele R. Hacker; Megha Gupta

Beth Israel Deaconess Medical Center, Boston, MA

**Objective**: To compare the accuracy of a non‐invasive (finger sensor), point‐of‐care hemoglobin (Hb) monitoring device to the gold‐standard blood test among pregnant patients.

**Study Design**: This cross‐sectional study included pregnant patients across all trimesters undergoing routine venipuncture for Hb assessment during prenatal visits at a tertiary care center. We collected two device measurements at time of venipuncture and averaged the values. We also collected self‐reported skin tone using the Monk Skin Tone scale. The primary outcome was the difference between the device and blood Hb. We used the Bland‐Altman plot to visualize data, assessed the independence of the bias, and calculated the Pearson Correlation coefficient overall and stratified by skin tone. Anemia was defined as Hb <11 g/dL in the first and third trimesters and Hb <10.5 g/dL in the second.

**Results**: We enrolled 124 participants, 38 (31%) in the first trimester, 40 (32%) in the second, and 46 (37%) in the third. The median perfusion index was 8 (IQR 5–10), indicating adequate perfusion during testing. Mean Hb was 11.6 g/dL (±1.1) for blood and 13.1 (±1.1) for the device. The device result was 1.5 g/dL (±1.1) higher than the blood result across all Hb levels, as demonstrated by Figure 1 and the test of independence of the bias (*r* = 0.04; *p* = 0.64). The overall Pearson correlation coefficient was 0.54 (*p* < 0.001) and did not vary notably when stratified by skin tone: light tones: *r* = 0.47 (*p* < 0.001), medium tones: *r* = 0.62 (*p* < 0.001), and dark tones: *r* = 0.58, (*p* = 0.01). The blood test identified 25 (20%) patients with anemia. The device identified 2, only one of which had anemia based on the blood test, yielding a sensitivity of 4% and specificity of 99%.

**Conclusion**: Results from the non‐invasive point‐of‐care Hb monitoring device consistently overestimated venipuncture results by 1.5 g/dL. Future work should center on determining whether a device‐specific anemia cutoff could increase device accuracy, this may improve rates of anemia screening.

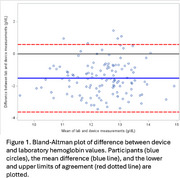

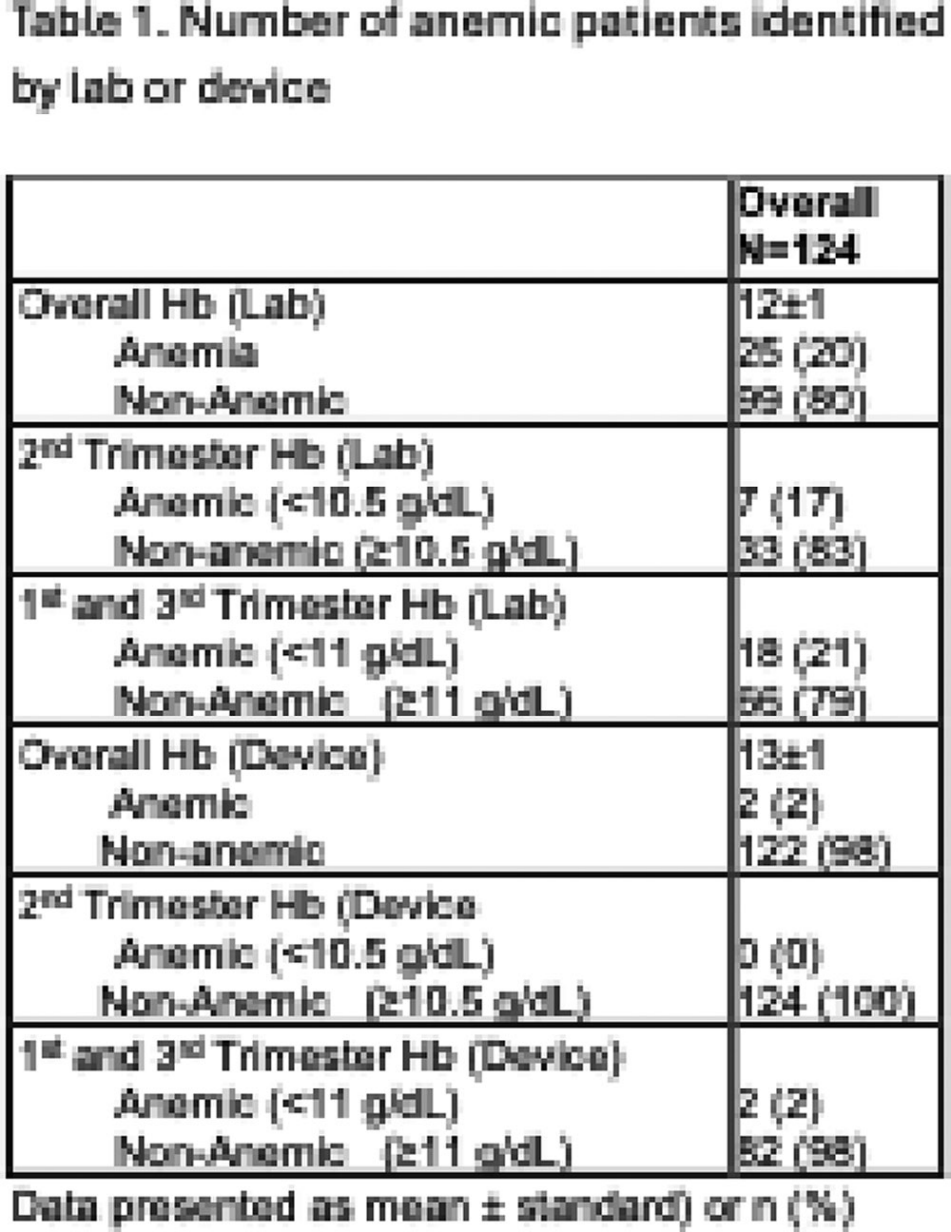

**3:30 PM – 5:00 PM**

## 486 | **Establishing a** Mean **Arterial Pressure Threshold for Severe Hypertension in Labor**

Frank I. Jackson; Sarah H. Abelman; Oladunni Ogundipe; Isabella A. Molina; Luis A. Bracero; Matthew J. Blitz

Northwell, New Hyde Park, NY

**Objective**: Current definitions of severe hypertension in pregnancy rely on different systolic and diastolic thresholds, which may be inconsistently applied in non‐obstetric clinical settings. Unlike these dual cutoffs, mean arterial pressure (MAP) offers a single, integrated value that could streamline recognition and triage. However, no validated MAP threshold currently exists. We sought to identify a MAP cutoff that reliably detects severe‐range blood pressure to improve detection and response in obstetric care.

**Study Design**: We conducted a retrospective cohort study of all deliveries across 7 hospitals from 2019–2024. Among 7,996,632 recorded blood pressures, 6,971,692 physiologic readings were analyzed after excluding extreme or missing values. Severe hypertension was defined per ACOG criteria. We used receiver operating characteristic (ROC) analysis and Youden's Index to determine the optimal MAP threshold and assessed diagnostic performance across clinically relevant cutoffs.

**Results**: Of the 6.97 million readings, 146,581 (2.1%) met criteria for severe hypertension. The optimal MAP threshold was 103.2 mmHg (AUC 0.981; sensitivity 94.7%; specificity 91.0%). For clinical use, a MAP ≥100 mmHg had sensitivity 98.1% and specificity 85.8%, missing only 1.9% of severe cases. A more conservative threshold of MAP ≥110 mmHg improved specificity (97.4%) but reduced sensitivity (79.1%). Mean MAP was 119 mmHg (SD 13.0) in severe cases vs 86.3 mmHg (SD 11.9) in non‐severe cases (*p* < 0.001).

**Conclusion**: A MAP threshold of ≥100 mmHg reliably detects severe‐range hypertension and may simplify recognition across diverse clinical settings, particularly where non‐obstetric providers may be less familiar with dual systolic/diastolic criteria requiring urgent treatment. Adoption of a single, memorable MAP cutoff could improve timely identification and treatment of severe hypertension in pregnancy

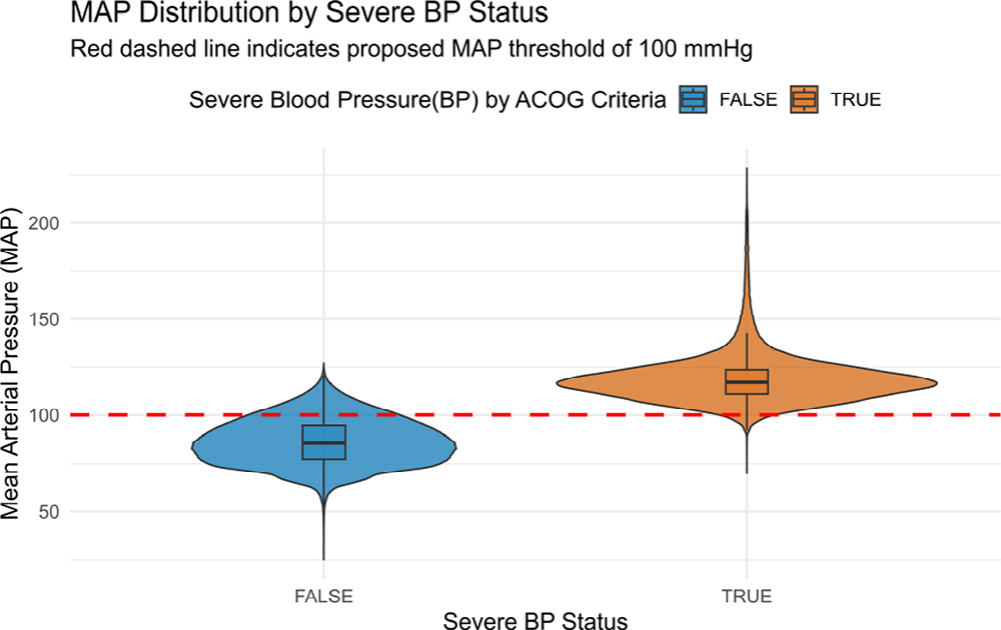

**3:30 PM – 5:00 PM**

## 487 | **Quantitative** Assessment **Of Biomechanical Changes During Cervical Ripening in Labor Induction**

Rachana Gavara^1^; Gabriela F. Tessler^1^; Abigail Laughlin^2^; Erin Louwagie^3^; Ruddys A. Pena Recio^4^; Kristin M. Myers^5^

^1^Columbia University Medical Center, New York, NY; ^2^Columbia University Department of Mechanical Engineering, New York, NY; ^3^Columbia University Medical Center, Columbia University, NY; ^4^Columbia University, New York, NY; ^5^Columbia University, Columbia University, NY

**Objective**: Labor induction is one of the most common obstetric procedures, used in 30% of deliveries, but little is known about the physiology and mechanics of cervical ripening. This study aimed to record quantitative measurements of the biomechanical changes in the cervix during cervical ripening with commonly used mechanical and pharmacological ripening methods.

**Study Design**: This was a prospective, exploratory study evaluating the feasibility of serial, quantitative cervical stiffness measurements during cervical ripening. Seventeen patients undergoing labor induction were enrolled. Participants were assigned to receive either pharmacologic (*n* = 5 Misoprostol) or mechanical (*n* = 7 Dilapan and *n* = 5 Foley Catheter) methods. Bishop score and cervical stiffness measurements (using the Pregnolia System) were obtained in all study subjects. Cervical stiffness was assessed every 4 hours for a period of 12 hours.

**Results**: This study describes one of the first attempts to measure the biomechanical changes of the cervix during the process of cervical ripening. 14 out of 17 (82.4%) patients had a vaginal delivery. 6 out of 17 (35.3%) were nulliparous (Table 1). There did not appear to be a meaningful trend in cervical stiffness changes during the process of cervical ripening across any methods (Chart 1). Bishop scores increased for all but 1 patient. Pre‐induction cervical stiffness was not associated with change in bishop score or with mode of delivery.

**Conclusion**: It is feasible to measure cervical stiffness before, during, and after cervical ripening. There were not identifiable trends in cervical stiffness measurements or correlations with bishop score, though this was a very small, limited sample size. Additionally, a possible factor that may have altered measurements is the presence of a mechanical method during some aspiration measurements, which may have artificially increased the stiffness measurement. Future studies are needed to detect the impact of different ripening agents on cervical stiffness and to understand if a quantitative measurement of cervical stiffness is associated with labor outcomes.

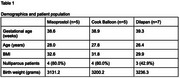

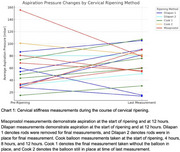

**3:30 PM – 5:00 PM**

## 488 | **Short** Interpregnancy **Interval After Fetal Growth Restriction: Impact on Subsequent Pregnancy Outcomes**

Efrat Leibowitz^1^; Gal Bachar^2^; Yuval Ginsberg^3^; Naphtali Justman^4^; Ron Beloosesky^5^; Yaniv Zipori^6^; Zeev Weiner^7^; Nizar Khatib^2^

^1^Rambam Health Care Campus, D.N. Emek Hamayanot, HaZafon; ^2^Rambam Health Care Campus, Haifa, HaZafon; ^3^Rambam health care campus, Haifa, Hefa; ^4^Rambam Medical Health Center, Haifa, Hefa; ^5^Rambam Health Care Campus, Rambam Health Care Campus, Hefa; ^6^Rambam Health Care campus, Haifa, HaZafon; ^7^Rambam Health Care campus, Haifa, Hefa

**Objective**: Fetal growth restriction (FGR) is a leading cause of perinatal morbidity and mortality. Counseling regarding optimal interpregnancy interval (IPI) after FGR is challenging due to limited data. This study evaluates whether a short IPI (≤ 18 months) increases maternal or neonatal risks in subsequent pregnancies.

**Study Design**: This study is a retrospective cohort study of women delivering at a tertiary medical center between 2008 and 2024. The cohort included women with a prior FGR delivery (birthweight <10th percentile) and a subsequent live birth. Women were allocated by IPI into two groups: short IPI (≤18 months) and long IPI (>18 months). Maternal and neonatal outcomes were compared between groups. Primary outcomes were recurrent FGR and hypertensive disorders; secondary outcomes included a composite of maternal and neonatal complications.

**Results**: A total of 984 women were included; 413 (42%) had a short IPI and 571 (58%) had a long IPI. Rates of recurrent FGR were similar in both groups (26.2%, *p* = 0.99). No significant differences were observed in hypertensive disorders or other maternal complications. Neonatal outcomes, including birthweight and neonatal intensive care unit (NICU) admission, were comparable between groups (all *p* >0.05).

**Conclusion**: Our data suggest that a short IPI following a FGR pregnancy was not associated with an increased risk of adverse maternal or neonatal outcomes in subsequent pregnancies. These results offer reassurance for women considering earlier conception; larger studies are needed to validate these findings.

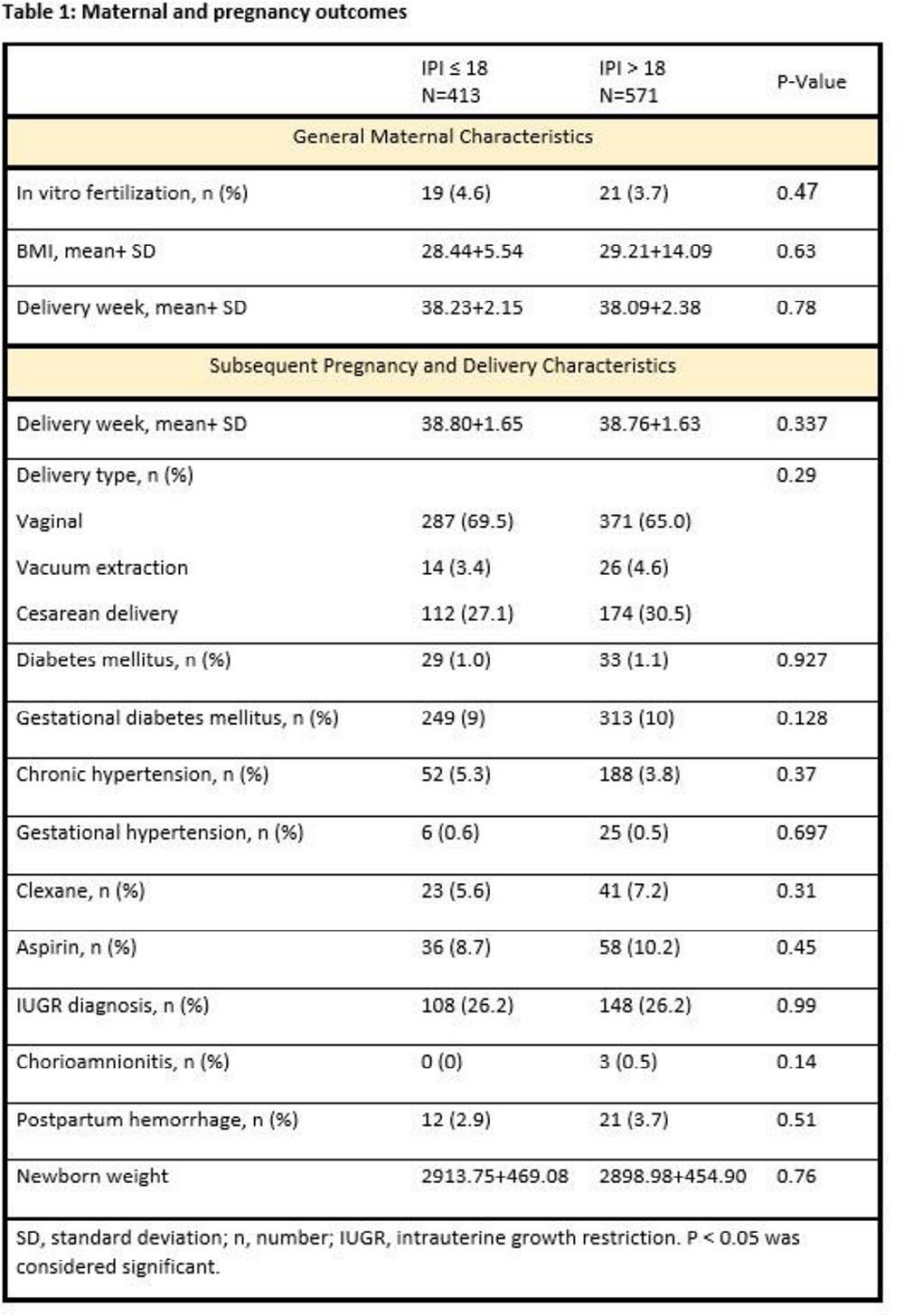

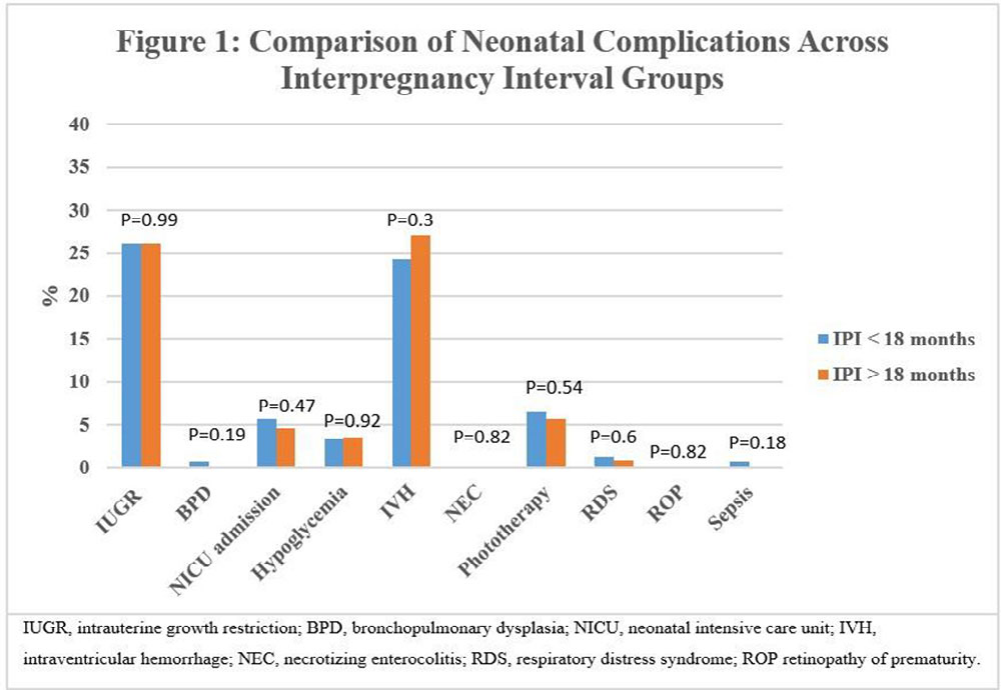

**3:30 PM – 5:00 PM**

## 489 | **Estimated versus Quantitative Blood Loss and Associated Hemorrhagic Complications: A Pre‐ and Post‐Intervention Study**

Gary R. Kersbergen^1^; Cassidy A. O'Sullivan^2^; Suneet P. Chauhan^3^; Matthew K. Hoffman^1^; Jessica C. Fields^4^

^1^Christiana Care Health System, Newark, DE; ^2^Christiana Care Health Services, Newark, DE; ^3^Delaware Center for Maternal‐Fetal Medicine of Christiana Care, Newark, DE; ^4^Delaware Center for Maternal Fetal Medicine, Newark, DE

**Objective**: American College of Obstetrics and Gynecology (ACOG) has recommended using quantitative blood loss (QBL) rather than estimated blood loss (EBL) to accurately measure blood loss during delivery and enable clinicians to promptly identify and treat hemorrhage. Despite the theoretical benefits of ascertaining a more accurate representation of blood loss, there is limited evidence that demonstrates an improvement in clinical outcomes, such as the rate of blood transfusion.

**Study Design**: We performed a pre‐ and post‐ analysis of the use of EBL versus QBL. The primary outcome was to compare the rate of blood transfusion. Secondary outcomes included hospital length of stay, rate of hysterectomy, and maternal death.

**Results**: A total of 46,554 deliveries performed at a single large tertiary hospital were analyzed (Table 1). Of these, 35,255 (75.3%) utilized EBL and 11,541 (24.7%) utilized QBL. The percentage of deliveries by cesarean section was 32.48% (*N* = 11,451) in the EBL group and 33.43% (*N* = 3,858) in the QBL group. Patients in the QBL group were more likely to receive a blood transfusion during their care when compared to the EBL group (3.58% versus 5.94%; OR = 1.70, 95% CI 1.55, 1.87; *p* >0.001). The rate of hysterectomy was 0.03% in both the QBL and EBL groups (*p* = 0.89). The average length of stay in the EBL group was 3.01 days, equal to that of the QBL group. There were no maternal deaths reported.

**Conclusion**: This study calls into question the ACOG recommendation to use QBL over EBL, as the use of QBL appears to increase the likelihood of receiving a blood transfusion without any impact on additional adverse maternal hematologic outcomes or maternal mortality.

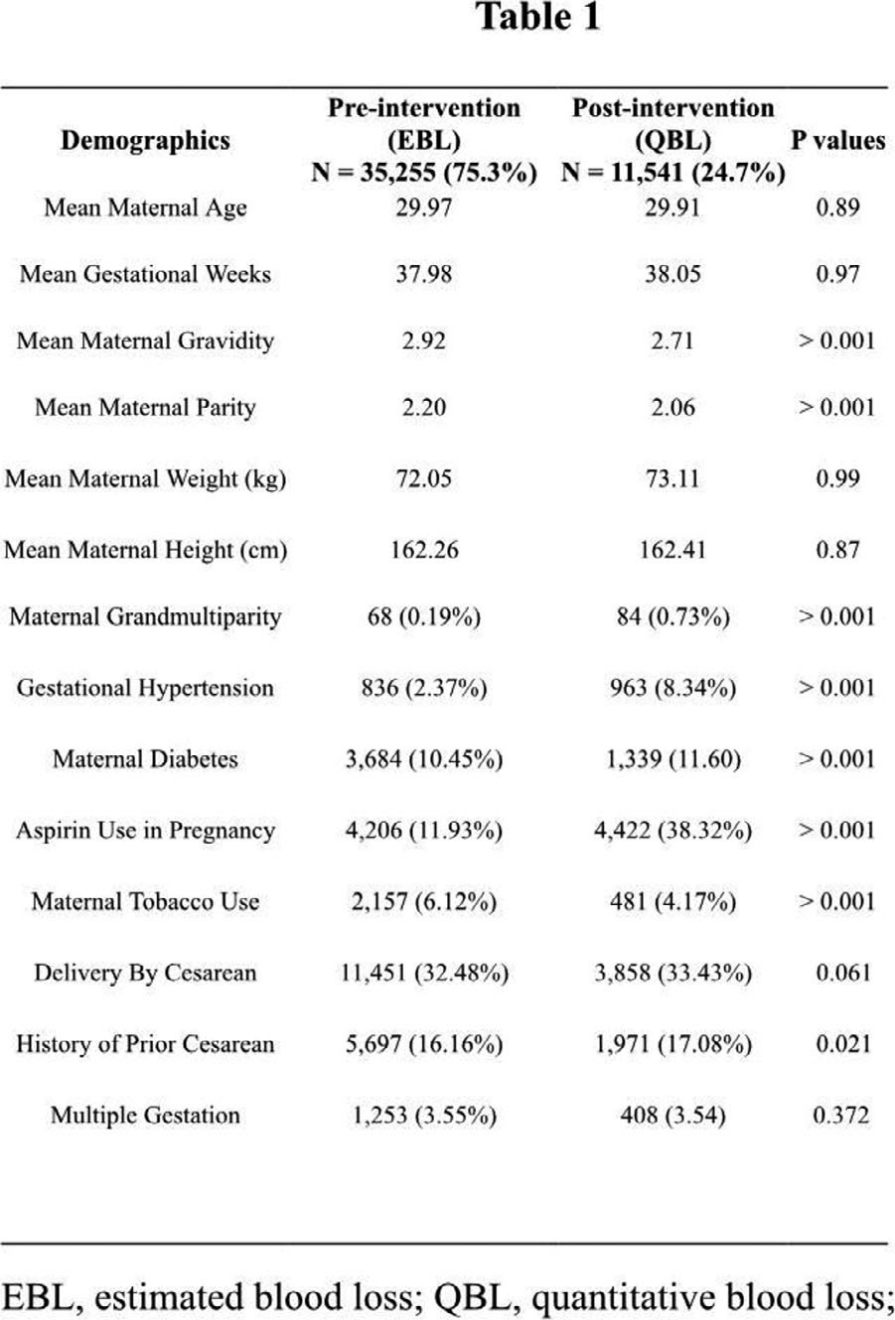

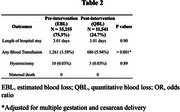

**3:30 PM – 5:00 PM**

## 490 | **Optimal** Continuous **Glucose Monitor “Time in Range” and Neonatal Hypoglycemia in Pregnant Patients With Diabetes**

Genevieve Mazza^1^; Alesandra Rau^1^; Megan C. Oakes^2^

^1^UC Irvine Medical Center, Orange, CA; ^2^MemorialCare Miller Children's and Women's Hospital, Long Beach, CA

**Objective**: Neonatal hypoglycemia is a common and serious complication of sub‐optimally controlled gestational diabetes requiring medication (A2GDM) and type 2 diabetes mellitus (T2DM). The use of a continuous glucose monitor (CGM) has been shown to reduce this risk in patients with T1DM with a target time in range (TIR) >70%. As CGMs are increasingly used for management of A2GDM and T2DM in pregnancy, we aimed to determine the optimal TIR to reduce the risk for neonatal hypoglycemia in this population.

**Study Design**: This was a retrospective cohort study of patients with A2GDM or T2DM utilizing a CGM in the 3^rd^ trimester with an optimal glucose range defined as 70–140mg/dL delivered at a single tertiary care center from Jan 2021‐May 2025. Rates of neonatal hypoglycemia, the primary outcome, were compared between those who had 3^rd^ trimester TIR <70% or >70%. Area under the receiver operative curves (AUROC) were generated to assess TIR as a predictor of avoiding neonatal hypoglycemia. The Youden index identified the optimal TIR cut‐point and the predictive characteristics of the cut‐point to avoid neonatal hypoglycemia.

**Results**: Of the 133 patients included, 87 (65%) achieved TIR >70% and 46 (35%) had TIR <70%. The AUROC of TIR for avoiding neonatal hypoglycemia was 0.64 (95% CI 0.54–0.74). Using the Youden index, the optimal cutoff of third trimester TIR to avoid neonatal hypoglycemia was 76.5%, with a sensitivity, specificity, positive predictive value, and negative predictive value of 66%, 63%, 68% and 61.4%, respectively.

**Conclusion**: A goal CGM TIR >70% is a modest predictor for neonatal hypoglycemia. With the use of CGMs for pregnant patients with T2DM and A2GDM, a TIR target of at least 76% appears to be the optimal target to avoid neonatal hypoglycemia, higher than that which is extrapolated from 70% target used for those with T1DM.

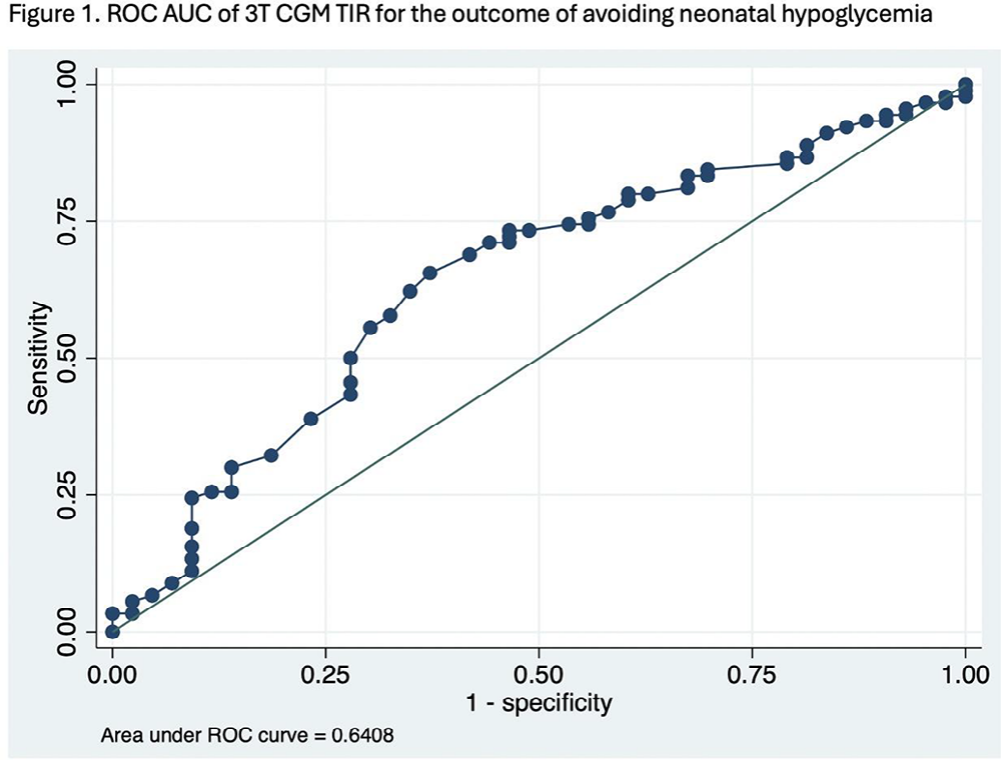

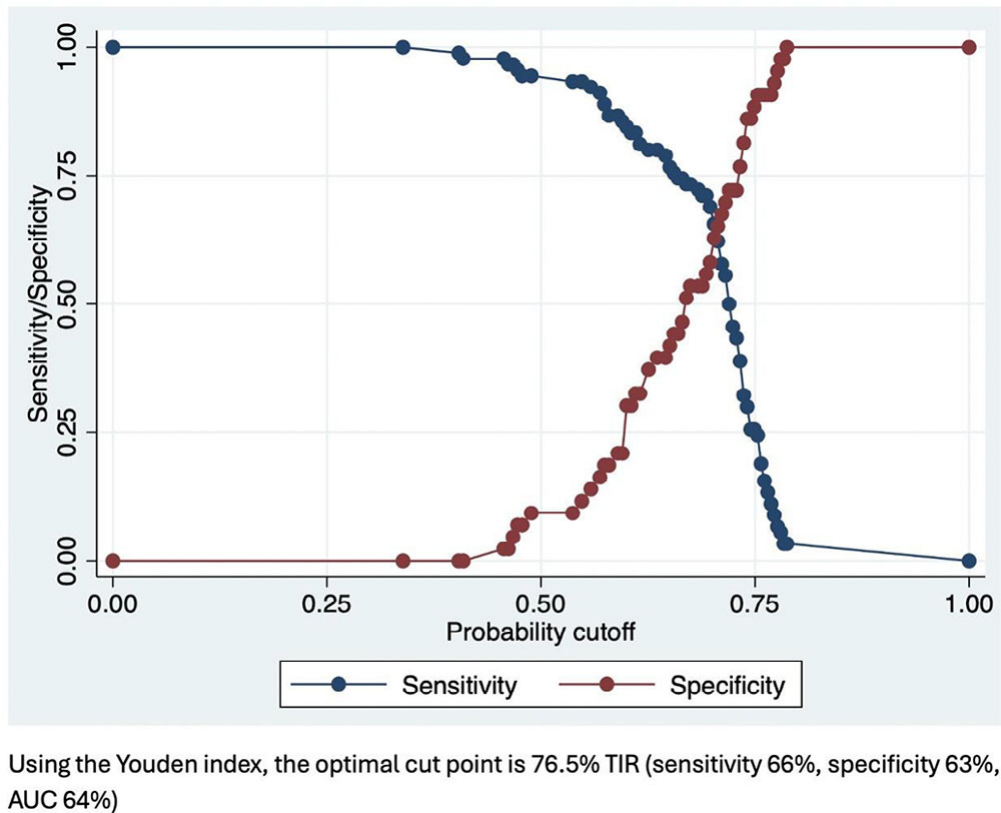

**3:30 PM – 5:00 PM**

## 491 | **Development of Therapeutics for Preterm Premature Rupture of Membranes Using Regenerative Medicine**

Geum Joon Cho^1^; Ho Yeon kim^2^; Yeseon Seok^2^; Heegyeong Song^2^; Jaewon Ju^3^; Hyun‐Joo Seol^2^; Min‐Jeong Oh^2^; Mikyung Shin^3^

^1^Department of Obstetrics and Gynecology, Korea University College of Medicine, Korea, Department of Obstetrics and Gynecology, Korea University College of Medicine, Korea, Seoul‐t'ukpyolsi; ^2^Department of Obstetrics and Gynecology, Korea University College of Medicine, Department of Obstetrics and Gynecology, Korea University College of Medicine, Seoul‐t'ukpyolsi; ^3^Department of Intelligent Precision Healthcare Convergence, Sungkyunkwan University, Department of Intelligent Precision Healthcare Convergence, Sungkyunkwan University, Kyonggi‐do

**Objective**: Preterm premature rupture of membranes (PPROM) is a major cause of spontaneous preterm birth and associated neonatal complications, including cerebral palsy. Currently, no effective treatment exists to restore the integrity of ruptured fetal membranes. This study aimed to develop a novel biomaterial‐based therapy using shape‐adaptive, tissue‐adhesive hydrogels to physically seal and regenerate ruptured membranes, enabling prolonged pregnancy.

**Study Design**: We developed a chitosan‐based hydrogel film enriched with plant‐derived polyphenols to form a biocompatible, adhesive patch. The material was engineered to seal ruptured amniotic membranes and support tissue integration. In vitro experiments assessed patch retention and cellular infiltration. In vivo, a PPROM model was established by rupturing the amniotic membrane in pregnant mice at embryonic day 15. The hydrogel patch was applied to the rupture site, and therapeutic efficacy was evaluated based on membrane healing and pregnancy duration.

**Results**: The hydrogel patch formed a stable adhesive film at room temperature, maintaining attachment for at least seven days and degrading gradually. It adhered securely to the membrane across defects of varying sizes. Histological analysis confirmed cellular infiltration and tissue compatibility. In vivo, patch application restored membrane continuity and significantly prolonged pregnancy to term compared with controls.

**Conclusion**: This study demonstrates the therapeutic potential of a bioadhesive hydrogel patch for treating PPROM. The patch promotes membrane regeneration and prevents preterm birth in a murine model. Future work should explore integrating drug delivery systems to enhance antimicrobial and regenerative effects for clinical application.

**3:30 PM – 5:00 PM**

## 492 | **Exploring the** Link **Between Maternal Chronic Hypertension and Offspring Neurological Health**

Gil Gutvirtz^1^; Tamar Wainstock^2^; Eyal Sheiner^3^

^1^Department of Obstetrics and Gynecology, Soroka University Medical Center, Ben‐Gurion University, Metar, HaDarom; ^2^Department of Epidemiology, Biostatistics and Community Health Sciences, Faculty of Health Sciences, Ben‐Gurion University of the Negev, Beer Sheva, HaDarom; ^3^Soroka University Medical Center, Faculty of Health Sciences, Ben‐Gurion University of the Negev, beer sheva, HaDarom

**Objective**: The prevalence of maternal chronic hypertension has increased in recent decades, mainly due to rising maternal age and obesity. While hypertensive disorders in pregnancy are linked to adverse pregnancy and neonatal outcomes, the long‐term effects on offspring remain understudied. Most studies have focused on preeclampsia‐related outcomes. This study aims to investigate long‐term neurological morbidity in offspring exposed in utero to maternal chronic hypertension, excluding preeclampsia.

**Study Design**: A population‐based retrospective cohort study was conducted using singleton deliveries from 1991–2021 at a tertiary center. Deliveries of women with chronic hypertension (without superimposed preeclampsia) were compared to those without chronic hypertension or other hypertensive disorders of pregnancy. Long‐term neurological morbidity was assessed using ICD‐9 diagnoses from community clinics and hospitalizations of offspring up to 18 years. Kaplan–Meier survival curves compared cumulative morbidity, and a Cox proportional hazards model controlled for confounders.

**Results**: The study included 342,365 singleton deliveries, of which 3097 (0.9%) were from mothers with chronic hypertension. Children exposed to maternal chronic hypertension had a significantly higher rate of neurological morbidity compared to those from pregnancies without hypertensive disorders (Table). Kaplan‐Meier analysis showed a higher cumulative incidence of neurological morbidity in the chronic hypertension group (Figure). A Cox regression model, adjusting for maternal age, fertility treatment, preterm delivery, and cesarean delivery, confirmed that maternal chronic hypertension is an independent risk factor for long‐term neurological morbidity of the offspring (adjusted HR = 1.14, 95% CI 1.05–1.23, *p* < 0.001).

**Conclusion**: Maternal chronic hypertension, unrelated to preeclampsia, is an independent risk factor for long‐term neurological morbidity in offspring. Despite the increased relative risk, the absolute risk remains low. Further research is necessary to explore underlying mechanisms and potential preventive strategies.

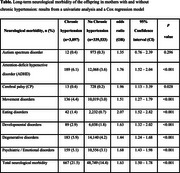

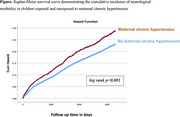

**3:30 PM – 5:00 PM**

## 493 | **Long‐Term** Offspring **Health Following Fertility Treatments in Women Aged 40 and Above**

Gil Gutvirtz^1^; Tamar Wainstock^2^; Eyal Sheiner^3^

^1^Department of Obstetrics and Gynecology, Soroka University Medical Center, Ben‐Gurion University, Metar, HaDarom; ^2^Department of Epidemiology, Biostatistics and Community Health Sciences, Faculty of Health Sciences, Ben‐Gurion University of the Negev, Beer Sheva, HaDarom; ^3^Soroka University Medical Center, Faculty of Health Sciences, Ben‐Gurion University of the Negev, beer sheva, HaDarom

**Objective**: The global rise in pregnancies of women of advanced maternal age (AMA) has been accompanied by a growing reliance on fertility treatments to achieve pregnancy. These pregnancies are associated with increased obstetric risks, which may potentially influence the health trajectories of the offspring. Data on long‐term morbidity in children conceived via fertility treatments, particularly in the context of AMA, remain sparse. We investigated the long‐term health of children conceived via fertility treatments at AMA.

**Study Design**: We conducted a retrospective cohort study of singleton deliveries of primiparas aged ≥40 who delivered between the years 1991–2021 at a tertiary center. We investigated several aspects of long‐term offspring morbidities including cardiac, gastrointestinal, respiratory, endocrine and neurological disorders of children conceived via fertility treatments compared to those from spontaneous pregnancies, using data from both community clinics and hospitalization records. Kaplan–Meier survival curves were used to compare the cumulative incidence of the investigated morbidities and Cox regression models were constructed to control for confounders.

**Results**: A sum of 276 primipara women aged ≥40 were included in the study. Of them, 166 (60.1%) conceived using fertility treatments and 110 (39.9%) had spontaneous pregnancies. The rates of long‐term offspring morbidities investigated in this study (Table) as well as the cumulative incidence over time (Figure) were comparable among children conceived via fertility treatments and spontaneously conceived offspring. The Cox regression model, controlling for maternal and gestational age, diabetes mellitus, hypertensive disorders of pregnancy and cesarean delivery, found that the use of fertility treatments in women aged ≥40 was not a risk factor for adverse long‐term offspring health (Table).

**Conclusion**: The use of fertility treatments in women aged 40 and above did not adversely affect their offspring long‐term health. Our results may provide reassurance to women of advanced maternal age undergoing medically assisted reproduction.

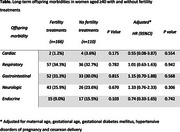

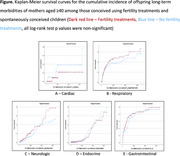

**3:30 PM – 5:00 PM**

## 495 | **Association** Between **Hypertensive Disorders of Pregnancy and Preterm Neonatal Respiratory Outcomes Following Betamethasone Administration**

Grace M. DiGiovanni^1^; Sara E. Edwards^2^; Nicola F. Tavella^3^; Sunidhi Singh^1^; Camila Johanek^1^; Sarriyah Hanif^1^; Gabriele Baptiste^4^; Dayana Mirzaliev^2^; Monica J. Patel^2^; Alexandra N. Mills^1^; Daniel Katz^1^; Chelsea A. DeBolt^2^; Angela T. Bianco^1^

^1^Icahn School of Medicine at Mount Sinai, New York, NY; ^2^Division of Maternal‐Fetal Medicine, Icahn School of Medicine at Mount Sinai, New York, NY; ^3^Division of Maternal‐Fetal Medicine, Icahn School of Medicine, Icahn School of Medicine at Mount Sinai Hospital, New York, NY; ^4^Icahn School of Medicine, Icahn School of Medicine, NY

**Objective**: Betamethasone administration for pregnant patients at risk of preterm delivery has been shown to improve neonatal outcomes. Research also suggests that neonates who develop in a hypertensive environment may clinically differ from those born to patients without hypertension. There are limited studies analyzing how hypertensive disorders of pregnancy (HDP) influence neonatal outcomes after betamethasone administration. We hypothesized that there would be no difference in neonatal respiratory outcomes after betamethasone administration between patients with and without HDP.

**Study Design**: This was a retrospective chart study of all patients delivering at a single academic institution in New York City from January 1, 2013 ‐ December 31, 2023 who received two doses of betamethasone prior to delivery and had a diagnosis of gestational hypertension, preeclampsia with or without severe features, eclampsia, or HELLP. We assessed electronic medical records for clinical and demographic data. Our primary outcome was a neonatal respiratory composite (transient tachypnea of the newborn, respiratory distress syndrome, use of CPAP for >12 hours, oxygen for >24 hours, intubation, and/or surfactant). Multivariable logistic regression models controlling for potential confounders examined associations of HDP with neonatal respiratory outcomes.

**Results**: 2638 patients were identified, of which 675 had HDP (Table 1). Patients with HDP delivered neonates at lower birth weights and earlier gestational ages and were more likely to have other comorbidities (Table 1). After controlling for confounders, neonates born to patients with any HDP had a higher likelihood of requiring CPAP for >12 hours (Table 2). Neonates of patients with pre‐eclampsia with severe features, eclampsia, or HELLP had a higher likelihood of developing RDS and TTN, and requiring CPAP for >12 hours and oxygen for >24 hours (Table 2).

**Conclusion**: Neonates born to patients with HDP, particularly severe forms, experienced worse respiratory outcomes than their counterparts, despite betamethasone administration.

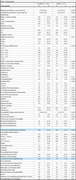

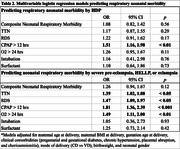

**3:30 PM – 5:00 PM**

## 496 | **National Trends** in **Postpartum Readmission for de Novo Endometritis, 2010–2021**

Grace K. Noonan^1^; Rachel L. Wiley^2^; Alexander M. Friedman^3^; Cynthia Gyamfi‐Bannerman^2^; Timothy Wen^2^

^1^Department of Obstetrics, Gynecology and Reproductive Science, University of California San Diego, San Diego, CA; ^2^Division of Maternal Fetal Medicine, Department of Obstetrics, Gynecology and Reproductive Sciences, University of California San Diego, San Diego, CA; ^3^Division of Maternal‐Fetal Medicine, Department of Obstetrics and Gynecology, Columbia University Irving Medical Center, New York, NY

**Objective**: Over the past decade, changing antibiotic practices have aimed to reduce delivery‐related infections; however, the impact on de novo postpartum endometritis readmissions is unknown. Our aim is to evaluate national trends and risk factors associated with 60‐day postpartum readmission for de novo endometritis during this time period.

**Study Design**: The 2010–2021 Nationwide Readmissions Database was utilized for this cross‐sectional analysis. Delivery hospitalizations (excluding those with delivery‐related infection, shock/sepsis, or hysterectomy) and de novo readmissions within 60 days complicated by endometritis were ascertained. Temporal trends for 60‐day postpartum endometritis readmissions were evaluated utilizing joinpoint regression to estimate average annual percent change (AAPC) and 95% confidence intervals (CI). Unadjusted and adjusted logistic regression models were fit to assess the relationship between clinical, demographic, and hospital factors with odds ratios (OR) to measure associations.

**Results**: Among 35.9 million births included, 69,712 (0.2%) were complicated by a 60‐day readmission complicated by endometritis. Rates of readmissions decreased from 23.8 to 14.5 per 10,000 birth hospitalizations (APC −5.3%, 95% CI ‐8.1%, ‐2.9%), with the largest decline in 2014–2017 (APC −12.9%; 95% CI–16.7%, ‐8.1%) (Figure). Readmission was more likely in younger patients and those with diabetes, obesity, multiple gestations, smoking, or immunosuppression (Table). Operative delivery (aOR 1.28; 95% CI: 1.20–1.36), cesarean delivery (aOR 1.94; 95% CI: 1.88–2.00), postpartum hemorrhage (aOR 2.08; 95% CI: 1.97–2.19) and dilation and curettage (aOR 1.97; 95% CI: 1.76–2.19) were independently associated with readmission.

**Conclusion**: National data show declining endometritis readmissions, suggesting benefits from recent practice changes. Yet, identifiable risk factors remain. Our findings support proactive postpartum care models, including education for symptom recognition, timely follow up and improved outpatient monitoring.

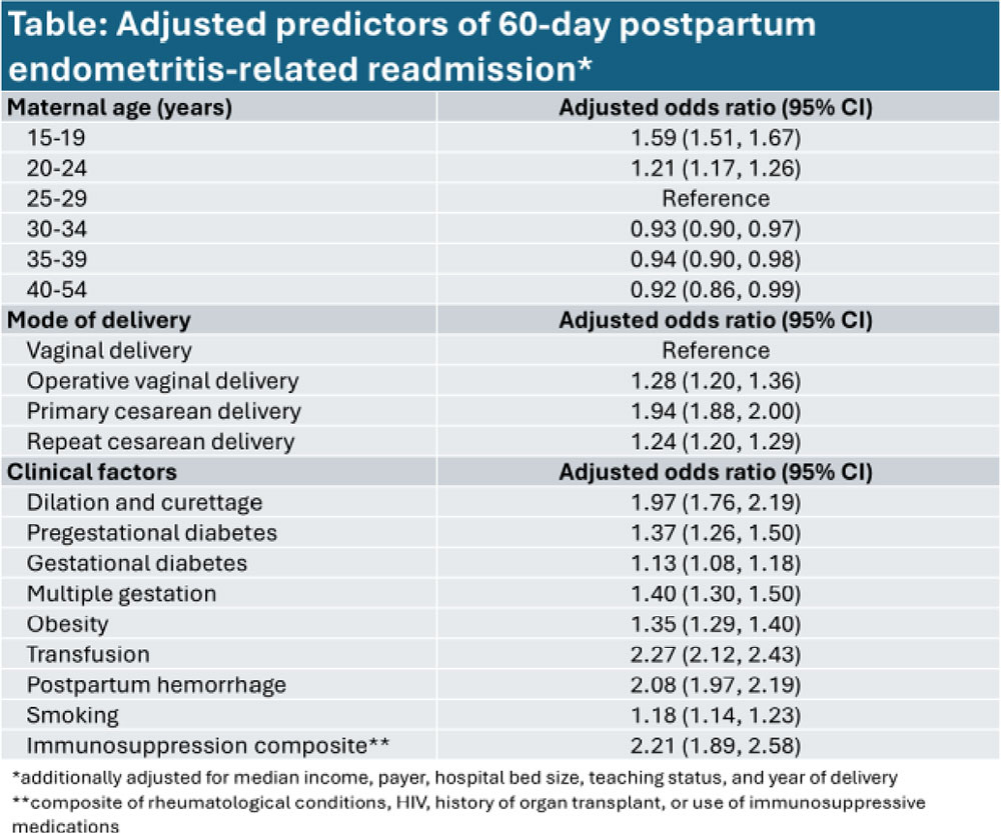

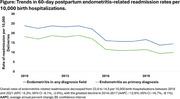

**3:30 PM – 5:00 PM**

## 497 | **Prenatal Mood** Symptoms **Predict Lower Objectively Measured Activity and Altered Perception of Activity Level**

Graciela Caraballo^1^; Hayley E. Miller^2^; Janet Hurtado^3^; Chi‐Hung Shu^4^; Jane Chueh^5^; Brendan Carvalho^3^; Pervez Sultan^3^; Nima Aghaeepour^5^; Maurice L. Druzin^6^; Danielle M. Panelli^3^

^1^Stanford Healthcare, Palo Alto, CA; ^2^University of California San Francisco, Department of Obstetrics, Gynecology and Reproductive Sciences, San Francisco, CA; ^3^Stanford University Healthcare, Palo Alto, CA; ^4^Stanford University, Palo Alto, CA; ^5^Stanford University, Stanford University, CA; ^6^Stanford University, Department of Obstetrics and Gynecology, Maternal Fetal Medicine, Palo Alto, CA

**Objective**: Physical activity promotes healthy pregnancy outcomes, yet many individuals fall short of recommendations. Depressive or anxiety symptoms are linked to reduced activity, but how these symptoms affect objectively measured activity, or influence self‐perception of activity, remains unclear. We examined associations between prenatal mood symptoms, measured physical activity, and discrepancies between perceived and actual activity.

**Study Design**: In this prospective observational study, 40 pregnant outpatients between 16–36 weeks’ gestation completed mental health screening, then wore wrist Actigraph accelerometers continuously for 7 days. Mood symptoms were defined as Edinburgh Postnatal Depression Scale (EPDS) >10 or State‐Trait Anxiety Inventory (STAI) >80. Physical activity metrics included daily steps, metabolic equivalent tasks (METs), moderate‐to‐vigorous physical activity (MVPA), and hourly kilocalorie (kcal) expenditure. Perceived activity was assessed by self‐reported average daily hours of activity during the monitoring period. Generalized estimating equations evaluated associations between mood symptoms and each activity metric, adjusting for confounders. Secondary analysis used Spearman correlations to compare measured vs. perceived activity, stratified by mood status.

**Results**: Nine (23%) of 40 participants screened positive for mood symptoms. Compared to those without symptoms, they recorded fewer daily steps (adjusted effect −1496 steps, 95% CI −2389 to −603), lower MVPA (−30.2 min/week, 95% CI −56.1 to −4.4), and reduced hourly kcal expenditure (−164.1, 95% CI −308.8 to −19.5, Table 1). Among participants with mood symptoms, actual steps and perceived hours of exercise were negatively correlated (*r* =−0.79, *p* = 0.0); no such correlation was seen in those without symptoms (Figure 1).

**Conclusion**: Prenatal mood symptoms were associated with lower objectively measured physical activity and inaccurate self‐perception of activity level. These findings highlight the behavioral and cognitive impact of mood symptoms in pregnancy and support sensor‐based monitoring to guide screening and intervention.

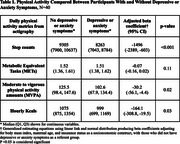

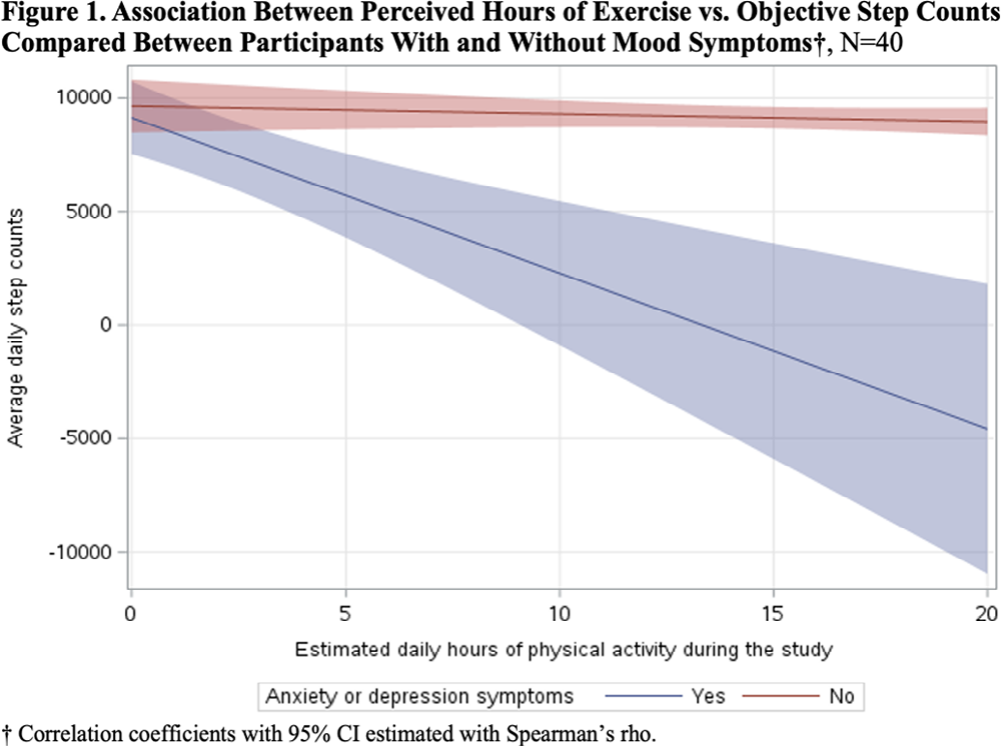

**3:30 PM – 5:00 PM**

## 498 | **Impact of** Specialized **Cardiac Care Proximity on Maternal and Neonatal Outcomes in Fetal Heart Disease**

Guadalupe Martinez^1^; Angela Desmond^2^; Anthony Jusuf^3^; Kara Calkins^3^; Yalda Afshar^4^

^1^University of California, Los Angeles (UCLA), Los Angeles, CA; ^2^CHLA, Los Angeles, CA; ^3^UCLA, Los Angeles, CA; ^4^David Geffen School of Medicine at University of California, Los Angeles (UCLA), Los Angeles, CA

**Objective**: To assess the association between proximity to a specialized cardiac care center and mode of birth, maternal and neonatal length of stay (LOS), and gestational age at birth with prenatally suspected fetal congenital heart disease (CHD), aiming to identify modifiable drivers of health outcomes (Figure 1).

**Study Design**: Prospective cohort study of pregnant people and neonates in California with prenatally suspected and postnatally confirmed fetal CHD at a single institution (2018–2022). Residence zip codes were mapped; and census‐tract social determinants were extracted using CalEnivoScreen. Subjects were grouped by travel time to birth hospital: <30 min, 30–90 min, and >90 min. Pregnancies were risk‐ stratified by a delivery room coordination algorithm based on prenatal CHD diagnosis. Outcomes included mode of birth, maternal and neonatal LOS, and gestational age. Chi‐square, ANOVA, and regression analyses compared outcomes by distance and cardiac severity.

**Results**: Among 214 maternal‐neonatal dyads, CHD severity was categorized as: 21% category 1, 51% category 2, 16% category 3, 10% category 4, and 2% unclassified. Thirteen percent lived <30 min, 66% lived 30–90‐min, and 21% lived >90 min from the hospital. Hispanic or Latino ethnicity was more prevalent among those living farther away (38% vs 11%, *p* = 0.003). Public insurance was more common in the >90 min group (56% vs. 11% for <30 min, *p* = 0.00038) and was associated with a 22‐day longer neonatal LOS (*p* < 0.001). Those farther from care were more likely to have the highest‐risk CHD (20% vs. 7%, *p* = 0.02). No significant differences were observed in planned (*p* = 0.433) or actual (*p* = 0.141) mode of birth by distance.

**Conclusion**: Greater distance from specialized cardiac care centers and public insurance status are associated with higher‐risk CHD and prolonged LOS, highlighting structural barriers that adversely impact outcomes. Addressing geographic and socioeconomic disparities is essential to improve equity and outcomes for this vulnerable population.

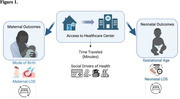

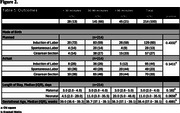

**3:30 PM – 5:00 PM**

## 499 | **Fetal Head** Station **at Second Stage Onset Predicts Delivery Outcomes in Nulliparous Women**

Hadas Zafrir Danieli^1^; Miriam Lopian^2^; Anat Pardo^1^; Or Bercovich^1^; Eran Hadar^1^; Ohad Houri^3^

^1^Rabin Medical Center, Petach Tikva, HaMerkaz; ^2^Helen Schneider Hospital for Women, Rabin Medical Center, Petah Tikva, HaMerkaz; ^3^BCNatal Fetal Medicine Research Center, BARCELONA, Catalonia

**Objective**: To investigate the association of fetal head station at the onset of the second stage of labor with delivery outcomes, specifically the duration of the second stage and the mode of delivery in nulliparous women.

**Study Design**: Retrospective cohort of all nulliparous women with singleton gestations who delivered at a single high‐volume tertiary center between 2012 and 2020. All women delivered at term (>37 gestational weeks) and progressed to the second stage of labor. Fetal head station at the onset of the second stage was categorized as below (S >0), at (S = 0), or above (S <0) the ischial spines. Delivery outcomes, including mode of delivery and second‐stage duration, were compared across these groups.

**Results**: 12,877 nulliparous women were included. Women with the fetal head below the ischial spines (S >0) had a higher rate of vaginal delivery (6019, 77.84%) and shorter second‐stage duration compared to women with the head at (437, 64.17%,) or above (3184, 69.82%) the ischial spines. The likelihood of cesarean delivery was low across all groups (S <0 36 5.29%, S = 0 102 2.29%, S >0 49 0.63%). A significant association was found between fetal head station at second stage onset, and the duration of the shorter second‐stage of labor (*p* < 0.01). The median duration of the second stage was 2.52, 2.17 and 1.32 hours for s <0, s+0 and s >0 respectively.

**Conclusion**: Fetal head station at the onset of the second stage of labor is an independent predictor of delivery outcomes in nulliparous women. A lower fetal head station is associated with a shorter second‐stage duration and a higher likelihood of vaginal delivery.

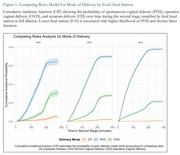

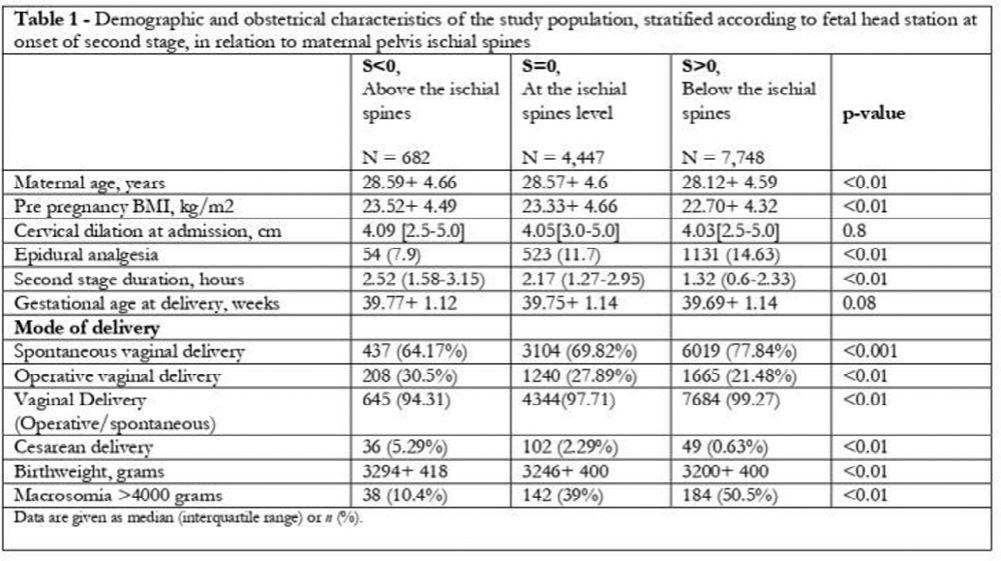

**3:30 PM – 5:00 PM**

## 500 | **Low Titer O+** Whole **Blood in Massive Transfusion of Obstetric Patients: a Retrospective Cohort Study**

Hadley Ross^1^; Alicia Davidson^1^; Donese Henneke^1^; Elisabeth Halm^1^; Victoria Epstein^1^; Terri‐ Jeanne Lui^1^; Emma Tao^1^; Alixandria F. Pfeiffer^2^; Erika Brigmon^1^; Donald Jenkins^1^; Kayla E. Ireland^1^

^1^UT Health San Antonio, San Antonio, TX; ^2^University of Texas Health San Antonio, University of Texas health San Antonio, TX

**Objective**: While whole blood is widely used in trauma, it remains underutilized in obstetrics. We evaluated the impact of low‐titer O+ whole blood (LTOWB) in massive transfusion protocols (MTP) for obstetric hemorrhage.

**Study Design**: We conducted a retrospective cohort study of obstetric patients who received MTP at a tertiary care center from 2016–2024. Patients who received component therapy (2016–2018) were compared to those who received LTOWB after implementation (2020–2024). Cases from the 2019 transition year were excluded. Primary outcome was transfusion volumes. Secondary outcomes included intensive care unit (ICU) admission, hysterectomy, and severe maternal morbidity. Demographics, transfusion volumes, and clinical outcomes were analyzed using t‐test, chi‐square analysis, and Fisher's exact test where appropriate.

**Results**: Seventy‐three patients received component therapy and 56 received LTOWB. Groups were similar in age, body mass index, parity, and Rh status. LTOWB patients had higher rates of diabetes (28% vs. 11%, *p* = 0.04), placenta accreta spectrum (PAS) (62% vs. 38%, *p* = 0.001), and earlier gestational age at delivery (31.7 vs. 34.8 weeks, *p* = 0.005). LTOWB was associated with a significant reduction in total transfusion volume (10.6 vs. 20.8 units, *p* = 0.036), including fewer units of packed red blood cells (2.8 vs. 11, *p* = 0.001), fresh frozen plasma (1.4 vs. 7.6, *p* = 0.006), and platelets (0.51 vs. 1.4, *p* = 0.09). Despite lower transfusion volume, ICU admission (60% vs. 32%, *p* = 0.001) and hysterectomy (71% vs. 41%, *p* = 0.001) were more frequent in the LTOWB group. DIC was less common with LTOWB (18% vs. 36%, *p* = 0.03). No significant differences were observed in maternal death, thromboembolic events, or lactic acid levels.

**Conclusion**: Despite a higher baseline risk in the LTOWB group due to increased PAS prevalence, LTOWB was associated with significantly lower total transfusion volumes. Increased ICU and hysterectomy rates likely reflect underlying severe maternal morbidity. LTOWB represents a more efficient transfusion strategy in obstetric hemorrhage.

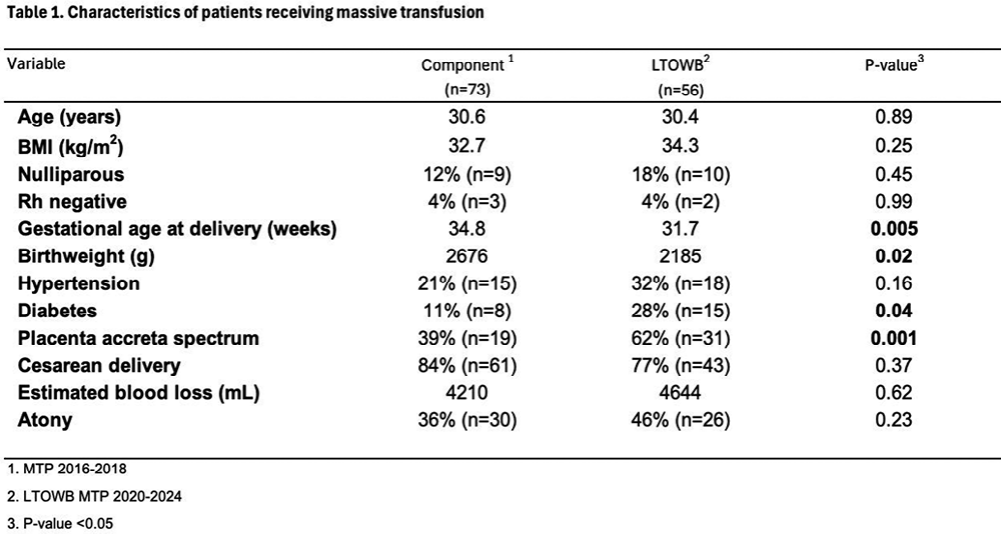

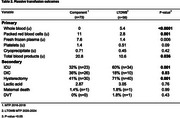

**3:30 PM – 5:00 PM**

## 501 | **Increasing Parity and Long‐term Risk of Maternal Psychiatric Morbidity: A Cohort Study**

Hagar Brami^1^; Gil Gutvirtz^2^; Tamar Wainstock^3^; Eyal Sheiner^4^; Ruslan Sergienko^5^; Roy Kessous^6^

^1^Soroka, 0, HaDarom; ^2^Department of Obstetrics and Gynecology, Soroka University Medical Center, Ben‐Gurion University, Metar, HaDarom; ^3^Department of Epidemiology, Biostatistics and Community Health Sciences, Faculty of Health Sciences, Ben‐Gurion University of the Negev, Beer Sheva, HaDarom; ^4^Soroka University Medical Center, Faculty of Health Sciences, Ben‐Gurion University of the Negev, beer sheva, HaDarom; ^5^Ben‐Gurion University of the Negev, ben gurion, HaDarom; ^6^Soroka, BEER SHEVA, HaDarom

**Objective**: While early postpartum psychiatric disorders are well‐recognized, the long‐term mental health implications of increasing parity remain unclear. Multiparity, particularly grand multiparity (≥5 births), may contribute to chronic psychological stress through repeated caregiving demands, hormonal fluctuations, and socioeconomic strain. This study aimed to investigate whether increasing parity is linked to a higher risk of psychiatric disorders later in life.

**Study Design**: A population‐based study of women who gave birth at a single tertiary hospital between 1991–2021 was conducted. We investigated the risk of long‐term psychiatric morbidity of mothers related to increasing parity. Only women who had concluded childbearing were included. Data for the incidence of psychiatric morbidity of the mother was extracted from maternal community and hospitalization records. Kaplan‐Meier survival curves were constructed to compare the cumulative incidence over time of maternal psychiatric morbidity between the study groups. A Cox proportional hazards model was used to control for possible confounders.

**Results**: A total of 54,346 women were included in the study with varying number of births. Overall psychiatric morbidity rate was lowest for primiparous (1 birth) women and increased with increasing parity, although not in a linear manner (Table). In the survival analysis considering the follow up time, increasing parity correlated with higher cumulative incidence of psychiatric morbidity, with the higher risk observed in women with three or more deliveries (Figure). A Cox proportional hazards model, controlling for the use of fertility treatments and ethnicity, confirmed that parity was an independent risk factor for psychiatric morbidity as compared to primiparas (Table).

**Conclusion**: In our cohort, increasing parity was independently associated with a higher risk of subsequent psychiatric morbidity in women. These findings underscore the need for mental health surveillance for multiparous women to mitigate potential adverse outcomes.

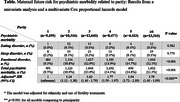

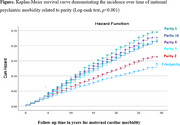

**3:30 PM – 5:00 PM**

## 502 | **Association** Between **Timing of Delivery After Corticosteroids Administration and Their Clinical Indication**

Rémi Micheletti^1^; Hugo Madar^1^; Frédéric Coatleven^2^; Amaury Brot^1^; Aurélien Mattuizzi^1^; Marie Vincienne^1^; Alizee Froeliger^1^; Loïc Sentilhes^1^; Hanane Bouchghoul^1^

^1^Bordeaux University Hospital, Bordeaux Universitary Hospital, Aquitaine; ^2^Bordeaux University Hospital, BORDEAUX, Aquitaine

**Objective**: The objective of this study was to evaluate the association between interval between antenatal corticosteroid administration and delivery across clinical indications.

**Study Design**: This retrospective study included women with singleton pregnancies who received antenatal corticosteroids for one of four clinical indications: preeclampsia, preterm prelabor rupture of membranes (PPROM), fetal growth restriction (FGR), or threatened preterm labor (TPL). The interval between corticosteroid administration and delivery (days) was assessed using Kaplan–Meier survival analysis for each indication, and differences between indications were compared using the log‐rank test. A multivariable Cox proportional hazards regression model was used to adjust for gestational age at corticosteroid administration (categorized), prior preterm birth, maternal age, BMI category, and previous cesarean.

**Results**: A total of 312 patients were included: 71 (22.8%) with preeclampsia, 125 (40.1%) with PPROM, 44 with FGR (14.1%), and 72 with TPL (23.1%). The median interval between corticosteroid administration and delivery significantly differed across the four groups (log‐rank *p* < 0.001), with the shortest interval observed in FGR and preeclampsia, and the longest in TPL. In the multivariable adjusted Cox model, the interval between corticosteroid administration and delivery remained significantly associated with the clinical indication. Compared to preeclampsia (reference group), the hazard of delivery was significantly lower in cases of TPL (HR = 0.47; 95% CI [0.30–0.74], p < 0.001), showed a trend toward being lower in PPROM (HR = 0.68; 95% CI [0.46–1.01], p = 0.054), and was not significantly different in FGR (HR = 0.93; 95% CI [0.59–1.47], p = 0.769).

**Conclusion**: The interval between corticosteroid administration and delivery varies significantly by clinical indication. These findings suggest that timing of corticosteroid administration could consider the expected latency to delivery associated with each indication to optimize neonatal benefit.

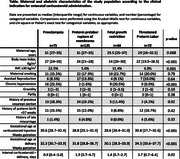

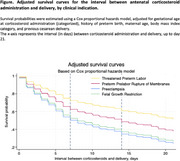

**3:30 PM – 5:00 PM**

## 503 | **Routine** Comprehensive **First Trimester Anatomy Scans: Enhanced Early Detection of Fetal Anomalies**

Hannah E. Bowers^1^; Lisa Gluszik Davis^2^; Kelly Woodmancy^2^; Maribeth Linsky^2^; Alia Grattan^2^; Sarah Graves^2^; Easha Patel^3^; Megan R. Ansbro^1^; Marissa A. Hand^1^; Yamen Ahmed^4^; Edward Chien^2^; Amol Malshe^2^; Ahmed Ahmed^2^

^1^Cleveland Clinic Foundation, Cleveland Clinic Foundation, Cleveland, OH; ^2^Cleveland Clinic Foundation, Cleveland Clinic Foundation, OH; ^3^Cleveland Clinic Foundation, Cleveland, OH; ^4^Case Western Reserve University, Cleveland, OH

**Objective**: The sonographic midsagittal fetal view traditionally used for nuchal translucency (NT) measurements provides limited anatomical information. Although SMFM and AIUM endorse targeted early anatomy assessments, neither mandates comprehensive first trimester fetal evaluations. This study assesses the added diagnostic value of routine comprehensive first trimester anatomy scans compared to standard NT scans for early detection of fetal anomalies.

**Study Design**: In June 2024, our institution adopted a protocol to routinely perform comprehensive first trimester fetal anatomy scans (including NT measurements) between 12 and 13 weeks and 6 days gestation, replacing traditional midsagittal NT sonograms. This retrospective cohort study compares patients who underwent standard NT scans (*N* = 11,010, June 2023–May 2024) with those who received comprehensive first trimester anatomy scans (*N* = 10,954, June 2024–June 2025). Data were extracted from a dedicated ultrasound registry. Scans were classified as abnormal if the NT thickness was >3 mm, structural anomalies were identified, or both. The primary outcome was abnormal scan frequency; secondary outcomes included detection rates of specific anomalies by organ system.

**Results**: Abnormal scans were reported in 0.87% of standard NT scans versus 0.76% of comprehensive first trimester anatomy scans (*p* = 0.34). The standard NT scan showed significantly higher detection of NT‐related abnormalities (increased NT/cystic hygroma; 0.84% vs. 0.36%; *p* < 0.007). Conversely, the comprehensive first trimester anatomy scan identified significantly more structural anomalies in craniofacial (0.17% vs. 0.07%; *p* = 0.036), cardiac (0.24% vs. 0.09%; *p* = 0.030), abdominal (0.23% vs. 0.10%; *p* = 0.048), renal (0.08% vs. 0.00%; *p* = 0.018), and spinal (0.07% vs. 0.00%; *p* = 0.021) systems.

**Conclusion**: Routine comprehensive first trimester anatomy scans significantly enhance early detection of structural fetal anomalies compared to standard midsagittal NT scans, facilitating earlier counseling and diagnostic testing. We recommend comprehensive first trimester anatomy screening for all pregnant women.

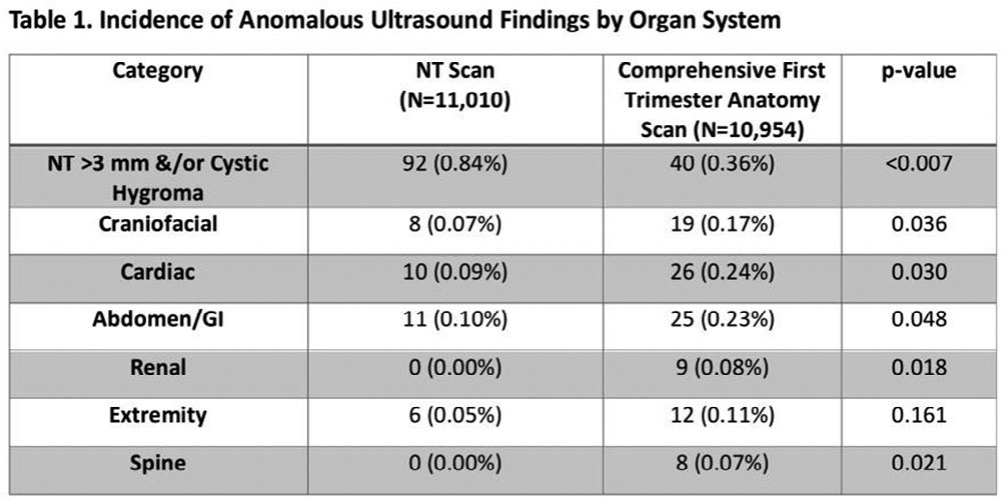

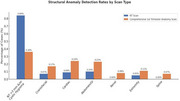

**3:30 PM – 5:00 PM**

## 504 | **Termination of** Pregnancy **Among Patients at Risk for Periviable Delivery**

Hannah Caldwell; Alison Asirwatham; Jessica Kloppenburg; Edesiri Igbuya; Yiming Zhang; Jessica Moszkowicz; Katherine Leung; Katherine Johnson

UMass Chan Medical School, Worcester, MA

**Objective**: Patients at risk of periviable delivery may opt for termination of pregnancy (TOP). We aimed to understand the clinical and demographic factors associated with patients’ decisions to pursue TOP in order to inform patient‐centered counseling and care.

**Study Design**: We retrospectively reviewed 207 patients admitted between 21w0d–24w6d at high risk for periviable delivery, excluding those with a documented plan for TOP prior to admission. We compared demographic and clinical characteristics between patients who underwent TOP (*n* = 22) and those who did not (*n* = 185), including gestational age at admission, estimated fetal weight, maternal age, race/ethnicity, BMI, mental health history, presence of fetal anomaly, and social factors.

**Results**: Overall, 35.3% (54/207) were counseled about TOP and 10.6% (22/207) underwent TOP. Patients who were counseled about the option for TOP were significantly more likely to undergo TOP (38.9% vs. 0.7%, *p* < 0.001). Counseling about TOP was documented in 95.5% (21/22) of patients undergoing TOP and 17.8% (33/185) of those continuing pregnancy. Patients opting for TOP presented at significantly earlier gestational ages (median 22.1 vs. 23.6 weeks, *p* < 0.001) and had lower estimated fetal weights (median 399g vs. 595g, *p* < 0.001). Those undergoing TOP had significantly lower BMI (median 26.1 vs. 30.9, *p* < 0.001). No significant differences were found in maternal age, race/ethnicity, presence of fetal anomalies, mental health disorders, or substance use. Receipt of periviability counseling was similar between groups (86.4% vs. 78.9%, *p* = 0.578).

**Conclusion**: Earlier gestational age, lower fetal weight, and lower maternal BMI were associated with termination of pregnancy in patients at high risk of periviable delivery. These findings highlight key clinical factors influencing patients’ choices and underscore the need for timely, nuanced counseling.

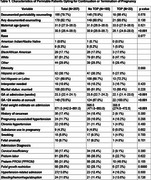

**3:30 PM – 5:00 PM**

## 505 | **Likelihood of** Shoulder **Dystocia in Nondiabetic Patients With Birthweight ≥ 4000 Grams**

Haviva Kobany^1^; Mary B. Janicki^2^; Samantha Pecenka^3^; Julia A. Dietz^4^; Dorothy B. Wakefield^5^

^1^University of New Mexico, University of New Mexico, NM; ^2^Trinity Health Of New England, Saint Francis Hospital, Hartford, CT; ^3^Frank H. Netter MD School of Medicine at Quinnipiac University, Frank H. Netter MD School of Medicine at Quinnipiac University, CT; ^4^Stamford Hospital, Stamford Hospital, Stamford, CT; ^5^Trinity Health Of New England, Trinity Health Of New England / Hartford, CT

**Objective**: To evaluate the likelihood of shoulder dystocia in neonates ≥4,000 grams when combining excessive gestational weight gain (GWG) with a 1‐hour glucose challenge test (GCT) ≥135mg/dL.

**Study Design**: This retrospective cohort study included 344 singleton, vaginal deliveries with birthweight ≥4,000 grams to nondiabetic patients at a single institution from 1/1/19–12/31/22. Patients were stratified into 3 risk groups: High (excessive GWG + GCT ≥ 135), Moderate (excessive GWG or GCT ≥ 135), Low (neither). Excessive GWG was defined as more than the upper limit of weight gain for pre‐pregnancy BMI by Institute of Medicine guidelines. The primary outcome was shoulder dystocia. Chi‐square test and Mantel‐Haenszel trend test were used for statistical analysis.

**Results**: The overall incidence of shoulder dystocia was 9.0% (31/344). There was a significant trend showing less dystocia as risk decreased: High 15.4% (8/52), Moderate 9.3% (20/215), Low 3.9% (3/77), *p* = 0.02. A larger proportion of Black 18.0% (7/39) and Hispanic 15.6% (7/45) patients had shoulder dystocia compared to White 6.8% (17/251) and Asian (0%), *p* = 0.04. More neonates in the dystocia group had Apgar scores (1 & 5 minutes) <7 and had higher initial glucose values. A logistic regression model, including race and risk group, revealed the High‐risk group was 4 times more likely to have shoulder dystocia compared to the Low‐risk group (OR 4.03, 95% CI 1.08–15.00), *p* = 0.04. Compared to White patients, both Black (OR 3.0, 95% CI 1.15–7.61) and Hispanic (OR 2.69, 95% CI 1.06–6.84) patients were almost 3 times as likely to have a shoulder dystocia.

**Conclusion**: Risk stratification based on excessive GWG + GCT≥135mg/dL in nondiabetic patients successfully identified singleton pregnancies with birthweight ≥4,000 grams that had an increased likelihood of shoulder dystocia, with the High‐risk group having over 4‐fold increased odds compared to the Low‐risk group. This model provides a practical tool for counseling and delivery planning in pregnancies complicated by macrosomia. Prospective validation could further optimize risk thresholds for clinical implementation.

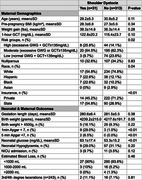

**3:30 PM – 5:00 PM**

## 506 | **Outcomes** Associated **With Hypertensive Disorders of Pregnancy in the Setting of Statewide Quality Improvement**

Haley Grayson^1^; Alexander M. Friedman^1^; Dena Goffman^1^; Mary D'Alton^1^; Maria Andrikopoulou^2^; Timothy Wen^3^

^1^Columbia University, New York, NY; ^2^Division of Maternal‐Fetal Medicine, Department of Obstetrics and Gynecology, Columbia University Irving Medical Center, New York, NY; ^3^UCSD, Irvine, CA

**Objective**: The NY Safe Motherhood Initiative (SMI), a statewide quality improvement effort, was convened in 2013 and included 117 of the 123 hospitals in NY State. A bundle to optimize management of obstetric hypertension was developed and disseminated. The purpose of this study was to evaluate trends in statewide outcomes related to hypertensive disorders of pregnancy (HDP) including gestational hypertension and preeclampsia.

**Study Design**: Delivery hospitalizations in the 2007–2022 NY State Inpatient Database (SID) were analyzed for this repeated cross‐sectional analysis. The NY SID includes all acute care inpatient discharge data for NY State. First, HPD diagnoses among all delivery hospitalizations were trended. Then, among deliveries with HDP, the following outcomes were trended: (i) transfusion, (ii) non‐transfusion severe maternal morbidity (SMM), and (iii) disseminated intravascular coagulation (DIC). Trends analyses were performed with joinpoint regression to determine the average annual percent change (AAPC). Because of small numerators, stroke risk during two 8‐year periods (2007–2014 and 2015–2022) was compared with the chi square test.

**Results**: Among 3,579,336 delivery hospitalizations, HDP increased from 6.1% in 2007 to 15.6% in 2022 (Figure 1). In joinpoint analysis, transfusion among deliveries with PPH increased from 2007 to 2012 but then decreased from 2012 to 2016 (AAPC −4.9%, 95% CI −10.9%, −0.2%) before increasing after 2016 (Figure 2). SMM increased from 2007 to 2014 before decreasing from 2014 to 2017 (AAPC −7.9%, 95% CI −11.5%, −1.4%), before rising again from 2017 to 2022. DIC increased from 2007 to 2013, decreased from 2013 to 2017 (AAPC −13.2%, 95% CI −19.4%, −7.9%) and increased non‐significantly from 2017 on. From 2007 to 2014, 44 cases of HDP‐associated stroke occurred compared to 19 from 2015 to 2022 (3.5 vs 1.0 per 10,000, *p* < 0.01).

**Conclusion**: The initiation of the NY SMI was associated decreased risk for a range of adverse outcomes among deliveries with HDP including stroke. Decreases in risk continued for approximately 3–4 years after initiation of the program.

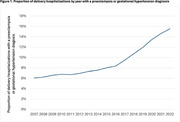

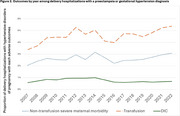

**3:30 PM – 5:00 PM**

## 507 | **Isolated** Abdominal **Circumference <10% in Fetal Growth Restriction: Indicator of Neonatal Outcomes or Overdiagnosis?**

Nikolas Bletnitsky^1^; Helen B. Gomez Slagle^2^; Shai Bejerano^2^; Uma M. Reddy^2^; Maria Andrikopoulou^3^; Olivia F. Schulist^2^

^1^Columbia University Medical Center, Brooklyn, NY; ^2^Columbia University Irving Medical Center, New York, NY; ^3^Division of Maternal‐Fetal Medicine, Department of Obstetrics and Gynecology, Columbia University Irving Medical Center, New York, NY

**Objective**: While abdominal circumference (AC) <10% is included in the definition of fetal growth restriction, its association with neonatal outcomes is unclear. We evaluated the association between isolated AC <10%, estimated fetal weight (EFW) <10%, and neonatal outcomes.

**Study Design**: This was a retrospective cohort of singleton pregnancies at a tertiary care center from 2020–2023. Fetal anomalies, missing data, and EFW >90% were excluded. Study arms were EFW and AC 10–90%, EFW <10 and AC 10–90%, EFW 10–90% and AC <10% (isolated AC <10%), and EFW and AC <10%. The primary outcome was serious composite neonatal morbidity (SCNM): acidosis, NICU admission, or neonatal death. Categorical variables were analyzed using Chi‐squared or Fisher's exact test; continuous variables with ANOVA or Kruskal‐Wallis. Logistic regression adjusted for maternal age, race, hypertension, pregestational diabetes, and pre‐pregnancy BMI.

**Results**: A total of 8870 patients met inclusion criteria. The rate of isolated AC <10% was 3.2%. An EFW <10% was associated with the highest SCNM risk, regardless of AC (33.3% vs 9.6%, *p* < 0.001) (Table 1). Fetuses with an isolated AC <10% had higher SCNM compared to normally grown fetuses (13.2% vs 9.6%, *p* < 0.001). An EFW <10% was also associated with the highest rate of neonatal death (5.6% vs 0.1%, *p* < 0.001). An isolated AC <10% was associated with a higher rate of neonatal death compared to normally grown fetuses (0.4% vs 0.1%, *p* < 0.001). The adjusted composite outcome was higher among EFW <10% regardless of AC (OR 3.93, CI 1.87–8.28, *p* = 0.0003) while isolated AC <10% was not associated with higher rate of SCNM after adjusting for confounders (Table 2).

**Conclusion**: In one of the largest cohorts linking prenatal ultrasound with neonatal outcomes, EFW <10% was strongly associated with SCNM and neonatal death, regardless of AC. Although isolated AC <10% showed increased unadjusted risk, it was not independently associated with adverse outcomes after adjustment. EFW <10% appears to be a more reliable predictor of neonatal risk than isolated AC <10%.

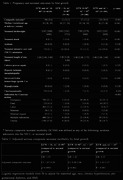

**3:30 PM – 5:00 PM**

## 508 | **Maternal** Nativity **and Preterm Birth Risk Among US Twin Pregnancies: A National Population Study**

Hiba J. Mustafa^1^; Jana Karam^2^; William A. Grobman^3^; Kevin Moss^4^; Jasmine Johnson^5^; Maged M. Costantine^6^; Heather A. Frey^7^; Moti Gulersen^8^; Vincenzo Berghella^9^; Alireza A. Shamshirsaz^10^

^1^Division of Maternal‐Fetal Medicine, Indiana University School of Medicine, Indianapolis, IN; ^2^Lebanese American University School of Medicine, Beirut, Beyrouth; ^3^Brown University, providence, RI; ^4^Indiana University, Indianapolis, IN; ^5^Indiana University Health, Indianapolis, IN; ^6^Ohio State University, Columbus, OH; ^7^The Ohio State University Wexner Medical Center, The Ohio State University Wexner Medical Center, OH; ^8^Division of Maternal Fetal Medicine, Department of Obstetrics, Gynecology and Reproductive Science, Jefferson Health, Philadelphia, PA, Philadelphia, PA; ^9^Thomas Jefferson University, Philadelphia, PA; ^10^Boston Children's Hospital, Harvard Medical School, Boston, MA

**Objective**: To assess whether maternal nativity (US‐born vs. non–US‐born) is associated with preterm birth (PTB) risk among nulliparous individuals with twin pregnancies and if this association varies by race/ethnicity.

**Study Design**: We conducted a cross‐sectional analysis of the US National Center for Health Statistics natality dataset (2016–2023), including nulliparous individuals aged 15–44 who delivered live‐born twins. Singleton, higher‐order multiples, and those with missing key data were excluded. Maternal nativity (US‐born vs. non–US‐born) was analyzed overall and stratified by race/ethnicity. Covariates included age, education, insurance, prenatal care, smoking, pre‐pregnancy BMI, diabetes, hypertension, and year. PTB was defined as <37 weeks, further categorized as extremely preterm (<29 weeks), moderately preterm (29–33 weeks), and late preterm (34–36 weeks). Binary and multinomial logistic regression models were used to estimate adjusted odds ratios (aOR) with 95% confidence intervals (CIs).

**Results**: Among 467,446 twin pregnancies, 39.5% delivered at term (≥37 weeks), while 60.5% were preterm. Of PTBs, 41.1% were late preterm, 14.6% moderately preterm, and 4.8% extremely preterm. Non–US‐born individuals had lower odds of PTB than US‐born peers (aOR 0.89, 95% CI 0.88–0.91). The greatest reduction in PTB risk was observed among non–US‐born Black individuals (aOR 0.65, 95% CI 0.62–0.68). Non–US‐born status was associated with lower odds of extremely preterm (aOR 0.88, 95% CI 0.84–0.91), moderately preterm (aOR 0.85, 95% CI 0.83–0.87), and late preterm birth (aOR 0.91, 95% CI 0.89–0.93).

**Conclusion**: In this national cohort, non–US‐born individuals had consistently lower PTB risk than US‐born counterparts with twin pregnancies across racial and ethnic groups. These findings highlight nativity as an important social determinant of preterm birth among twins. Future research should elucidate mechanisms underlying these differences and consider broader social and structural factors influencing PTB in multiples.

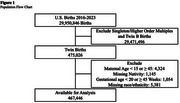

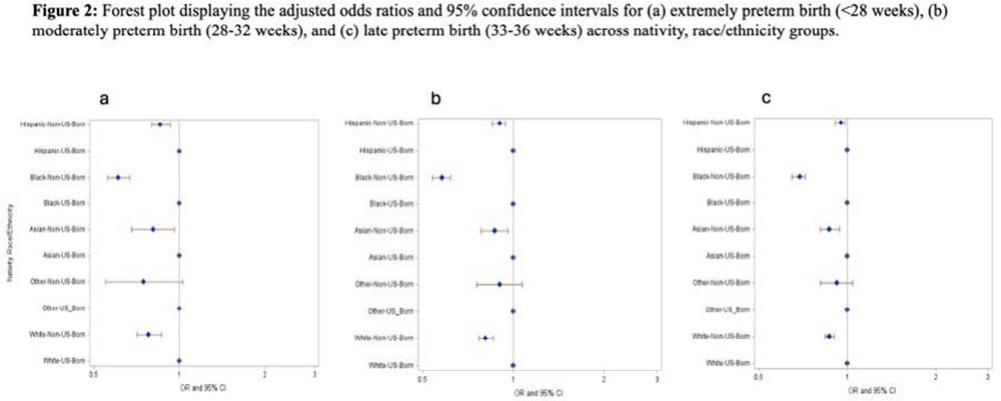

**3:30 PM – 5:00 PM**

## 509 | **Uterine Cavity** Evaluation **after Uterine Preserving Surgery in Placenta Accreta Spectrum: Comparative Surgical Outcomes Analysis**

Hila Lahav Ezra^1^; David Stockheim^2^; Michal Fishel Bartal^1^; Keren Zloto^3^; Shali Mazaki‐Tovi^4^; Eran Kassif^1^; Eyal Sivan^1^; Elias Castel^1^

^1^Division of Maternal‐Fetal Medicine, Department of Obstetrics, Gynecology and Reproductive Sciences, Sheba Medical Center, Tel Aviv University, Israel, Ramat Gan, HaMerkaz; ^2^Department of Obstetrics, Gynecology and Reproductive Sciences, Sheba Medical Center, Tel Aviv University, Israel, Ramat Gan, HaMerkaz; ^3^sheba medical center, Tel Hashomer, Tel Aviv; ^4^Department of Obstetrics and Gynecology, Sheba Medical Center, Ramat Gan, Tel Aviv

**Objective**: Uterine‐preserving surgical techniques are increasingly being developed as alternatives to the traditional approach of hysterectomy for individuals with placenta accreta spectrum (PAS), offering the potential to maintain future fertility. Various methods, including the LSR (Ligation‐Seperation‐Resection) technique, which includes bilateral uterine artery ligation, placenta separation, and resection of the defective uterine segment, have been proposed. The study aims to describe hysteroscopy findings for uterine cavity evaluation in individuals who had uterine preserving surgery.

**Study Design**: This was a retrospective study of all individuals who underwent conservative PAS surgery at a single referral care center (2019–2023) and had a follow‐up diagnostic hysteroscopy at 3 to 6 months. We compared individuals who were managed with the LSR surgical approach (study group) to a control group managed prior to the implementation of this technique (by uterine artery embolization or ligation and approximation sutures). Uterine cavity characteristics, including normal cavity, adhesions, niche, residua, or both, and intra and postoperative outcomes, were assessed.

**Results**: During the study period, 177 patients with PAS underwent surgery, 86 (49%) underwent the LSR technique, and 91(51%) had other interventions. Of them, 43 (50%) and 18 (19.7%), respectively, had follow‐up hysteroscopy to evaluate the uterine cavity. The LSR group had a higher normal uterine cavity rate (53.49% vs. 11.11%, *p* < 0.01) and a lower rate of residua (13.95% vs. 61.11%, *p* < 0.01). However, there was a higher rate of niche (13.95% vs. 5.56%, *p* = 0.34) and adhesions (11.63% vs. 5.56%, *p* = 0.46).

**Conclusion**: The majority of Individuals managed by the LSR surgical approach for conservative management had normal uterine cavities at 3–6 months following surgery, suggesting that this technique might be preferable in patients opting for fertility preservation after uterine preserving surgery.

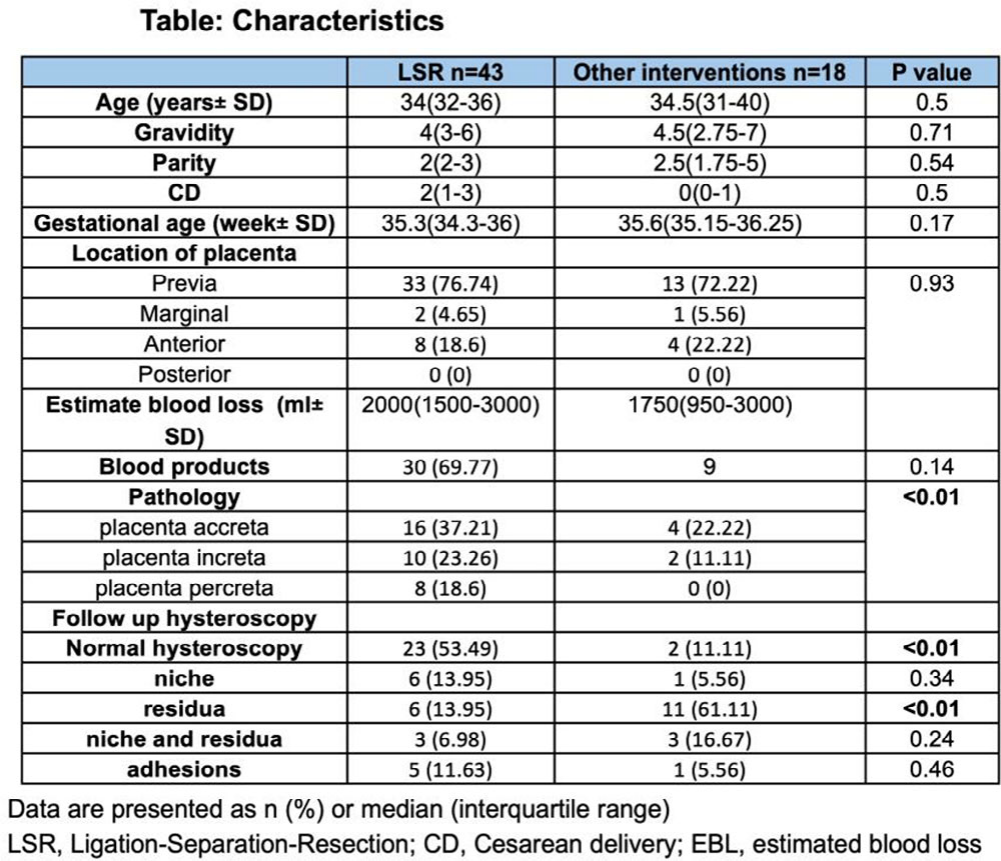

**3:30 PM – 5:00 PM**

## 510 | **Outcomes of** Pregnancies **Complicated by P‐Prom After Mid Trimester Amniocentesis**

Hila Shalev Ram^1^; Yael Rosental^2^; Yael Wolf^3^; Maya Finkelstein^4^; Ron Schonman^5^; Zvi Klein^6^; Eran Hadar^7^; Yariv Yogev^8^; Michal Kovo^9^; Shai Ram^4^; Tal Biron‐Shental^10^

^1^Department of Obstetrics and Gynecology Meir Medical Center, Kfar Saba, HaMerkaz; ^2^'Rabin medical center', Rabin medical center/ Petach tikwa, HaMerkaz; ^3^Faculty of Medical and Health Sciences, Tel Aviv University, Kfar Saba, HaMerkaz; ^4^Department of Obstetrics and Gynecology, Lis Maternity Hospital, Sourasky Medical Center, Tel Aviv, Israel, Tel Aviv, Tel Aviv; ^5^Department of Obstetrics and Gynecology, Meir Medical Center, Kfar Saba, Israel, Kfar Saba, Tel Aviv; ^6^Department of Obstetrics and Gynecology, Meir Medical Center, Kfar Saba, Israel, Kfar Saba, HaMerkaz; ^7^Helen Schneider Hospital for Women, Rabin Medical Center, Petach‐Tikva, Israel, Petach Tikva, HaMerkaz; ^8^Lis Hospital for Women's Health, Tel Aviv Sourasky Medical Center Gray Faculty of Medicine, Tel Aviv University, Israel, Lis Hospital for Women's Health, Tel Aviv Sourasky Medical Center, Tel Aviv; ^9^Shamir Medical Center, Israel; ^10^Meir Medical Center, Kfar Saba, HaMerkaz

**Objective**: P‐PROM after mid‐trimester amniocentesis (AC) is rare but clinically important. Although it is generally thought to have a more favorable course than spontaneous P‐PROM at similar gestational ages, data are limited to small series, and actual prognosis remains uncertain. We aimed to determine pregnancy outcomes and prognostic factors for a live fetus after mid‐trimester AC complicated by P‐PROM.

**Study Design**: Retrospective cohort in three university tertiary centers (2014–2025). Exclusions: multifetal gestation, elective terminations of pregnancy due to genetic abnormalities, clinical chorioamnionitis at admission. Cases were stratified as live vs. no live birth (termination of pregnancy [TOP] or late abortion [<= 22 weeks]). Multivariable logistic regression tested predictors of live birth.

**Results**: The final cohort included 93 women:

- Late abortion in 4 women (4.3 %; mean latency between AC to abortion 1.05 ± 0.77 wk).
- TOP for adverse findings in 38 women (40.9 %; latency between AC to TOP 3.08 ± 2.57 wk): oligohydramnios 24 (63 %), continuous leak 6 (15.8 %), chorioamnionitis 2 (5.3 %), contractions 2 (5.3 %), and single cases (2.6 % each) of pulmonary hypoplasia, placental abruption, IUFD, or unknown indication.
- Overall live births rate was: 51 / 93 (54.8 %). Of them, 37 (72.5 %) at ≥ 37 wk and 13 (25.5 %) prior to 37 wk, of them, 7 [7.5%] <34 wk, 4 [4.3%] <32 wk, 1 [1.1%] <28 wk. Both women with continuous leakage delivered preterm at 32.0 wk and 30.5 wk.

In multivariable analysis, AFI >5 cm at admission was the only independent predictor for live birth (OR 19.5; 95% CI 4.2–90.2; *p* < 0.001). Latency from AC to PPROM, IVF, BMI >30, maternal age, parity, previous abortions, and placental location were not significant.

**Conclusion**: Approximately half of women presenting with P‐PROM after mid‐trimester amniocentesis had a live birth. Most non‐viable outcomes were iatrogenic (TOP for adverse clinical status). AFI >5 cm at admission was the strongest predictor of favorable outcome.

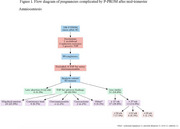

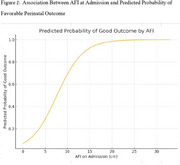

**3:30 PM – 5:00 PM**

## 511 | **Social** Determinants **of Health Associated With de Novo Opioid Use Disorder (OUD)‐Related Postpartum Re‐Admissions**

India Perez‐Urbano^1^; Maria Andrikopoulou^2^; Mary E. D'Alton^2^; Alexander M. Friedman^2^; Timothy Wen^3^

^1^Columbia University ‐ NY Presbyterian, Columbia University ‐ NY Presbyterian, NY; ^2^Division of Maternal‐Fetal Medicine, Department of Obstetrics and Gynecology, Columbia University Irving Medical Center, New York, NY; ^3^UCSD, Irvine, CA

**Objective**: Peripartum opioid‐related mortality has been on the rise. Social determinants of health (SDOH) have been associated with poorer clinical outcomes. To objective of this study was to evaluate risk factors for opioid use‐related 6‐month postpartum readmissions, and to explore associated trends and clinical outcomes.

**Study Design**: Delivery hospitalizations and 180‐day postpartum readmissions with de novo opioid use disorder (OUD) diagnoses were identified from the 2016–2021 Nationwide Readmissions Database. Delivery hospitalizations with pre‐existing OUD were excluded. Primary exposures of interest were SDOH relating to education/employment, physical environment, and social factors identified using ICD‐10 codes. Temporal trends in OUD‐related readmissions were assessed utilizing joinpoint regression to determine average annual percent change (AAPC) with 95% confidence intervals (CI). Unadjusted and adjusted logistic regression models were fit to assess the association between SDOH and 180‐day de novo OUD readmissions, adjusting for demographic and clinical factors.

**Results**: Of 10,285,974 delivery hospitalizations, there were 3659 postpartum readmissions with de novo OUD (3.5 per 10,000). The prevalence of 180‐day OUD‐related postpartum readmission decreased from 4.5 to 2.4 per 10,000 delivery hospitalizations in 2016 to 2022 (AAPC: −10.4%, 95% CI −13.5%, −7.8%). Presence of education/employment (18.2 vs. 3.6 per 10,000), physical environment (73.5 vs. 3.5 per 10,000), and social SDOH (14.8 vs. 3.5 per 10,000) were associated with higher prevalence of OUD‐related readmissions. Adjusted analysis demonstrates physical environment factors were the only SDOH factor associated with OUD‐related readmission (aOR 4.49, 95% CI: 3.04, 6.62).

**Conclusion**: While OUD‐related postpartum readmissions are decreasing, findings suggest that individuals with physical environment SDOH factors remain at disparately higher likelihood. Early identification of socioeconomic vulnerability and linkage to supportive resources has the potential to mitigate risk of opioid‐related adverse outcomes in the postpartum period.

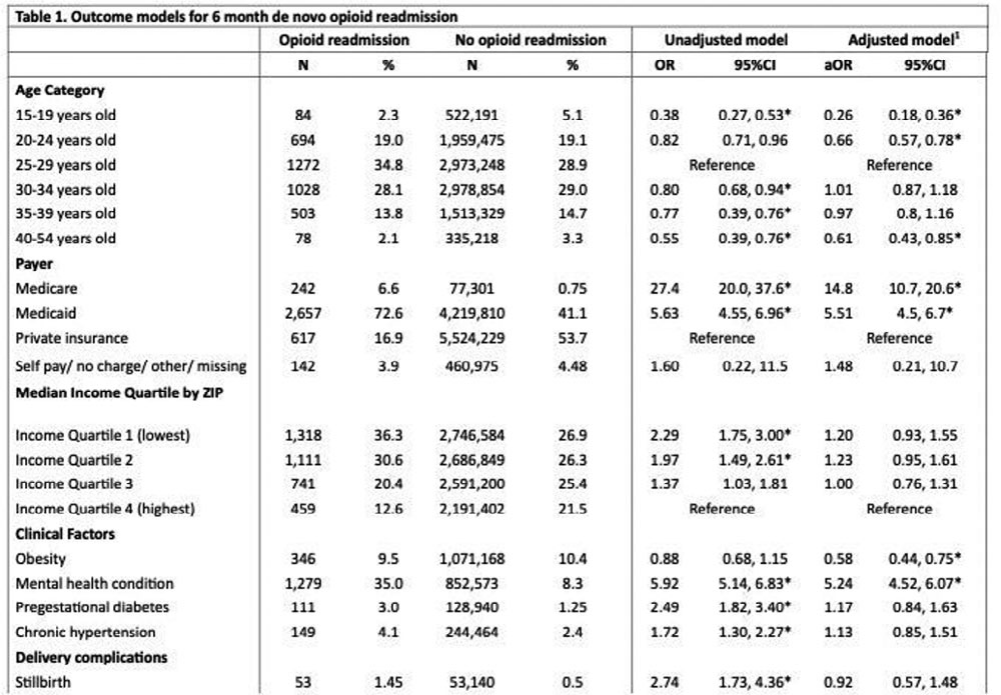

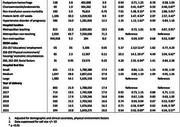

**3:30 PM – 5:00 PM**

## 513 | **Comparison of** Sepsis **Case Definitions Among Obstetric Delivery Encounters**

Isaac Michaels^1^; Nicole Krenitsky^2^; Thomas P. Kishkovich^3^; Astra Toyip^4^; Rachel Herz‐Roiphe^5^; Julia Middlesworth^4^; Lilian Chung^1^; Dahsan Gary^1^; Christian Schettini^1^; Julie Milne^1^; Lejdisa Stanaj^1^; Brianne Genow^1^; Amanda Ragsdale^1^; Janna Lay^1^; Baruch Fertel^1^; Jason Adelman^5^; Benjamin Ranard^5^; Dena Goffman^6^

^1^NewYork‐Presbyterian Hospital, New York, NY; ^2^NYP/Columbia, New York, NY; ^3^Division of Maternal‐Fetal Medicine, Department of Obstetrics and Gynecology, Columbia University Irving Medical Center, New York, NY; ^4^Columbia Vagelos College of Physicians and Surgeons, New York, NY; ^5^Columbia University Irving Medical Center, New York, NY; ^6^Columbia University, New York, NY

**Objective**: Accurate retrospective labeling of obstetric sepsis is critical for performance improvement, reporting and epidemiology. Multiple billing and electronic health record case definitions of sepsis exist for non‐pregnant adults. We sought to compare incidence rates of sepsis across four case definitions.

**Study Design**: A retrospective cohort study of all delivery encounters across a large, academic health system of 8 hospitals from 1/1/2023 to 12/31/2024 that met at least one of four sepsis case definitions: Centers for Disease Control (CDC) clinical definition, and Centers for Medicare & Medicaid (CMS), New York State Department of Health (NYSDOH), or Vizient ICD‐10 diagnosis code definitions. Identified charts were reviewed by obstetricians to determine if sepsis was present. The incidence rate of obstetric sepsis for each case definition was calculated and visualized and the frequency of intersection combinations of case definitions was visualized using an upset graph and Venn diagram.

**Results**: Of 49,083 delivery hospitalizations, 171 met criteria for at least one case definition. Encounter characteristics are summarized in the Table. Sepsis incidence rates varied by case definition: CDC 2.2%, CMS 1.9%, NYSDOH 0.5%, Vizient 2.4%, and clinical review 2.1%. Of the 171 cases, 60.8% were deemed to meet sepsis criteria on clinical chart review. The intersections of case definition combinations are shown in the Figure. There was large variability, with only 18 cases (10.5%) meeting the four case definitions and clinical review.

**Conclusion**: Discrepancies in incidence rates among different sepsis definitions applied to obstetric sepsis cases highlights variability in retrospective sepsis identification and suggests potential under‐ or overestimation of obstetric sepsis incidence. The use of any of the four case definitions may lead to variable identification of obstetric sepsis cases and estimates of incidence. Tailored criteria for obstetric sepsis clinical and coding definitions are warranted.

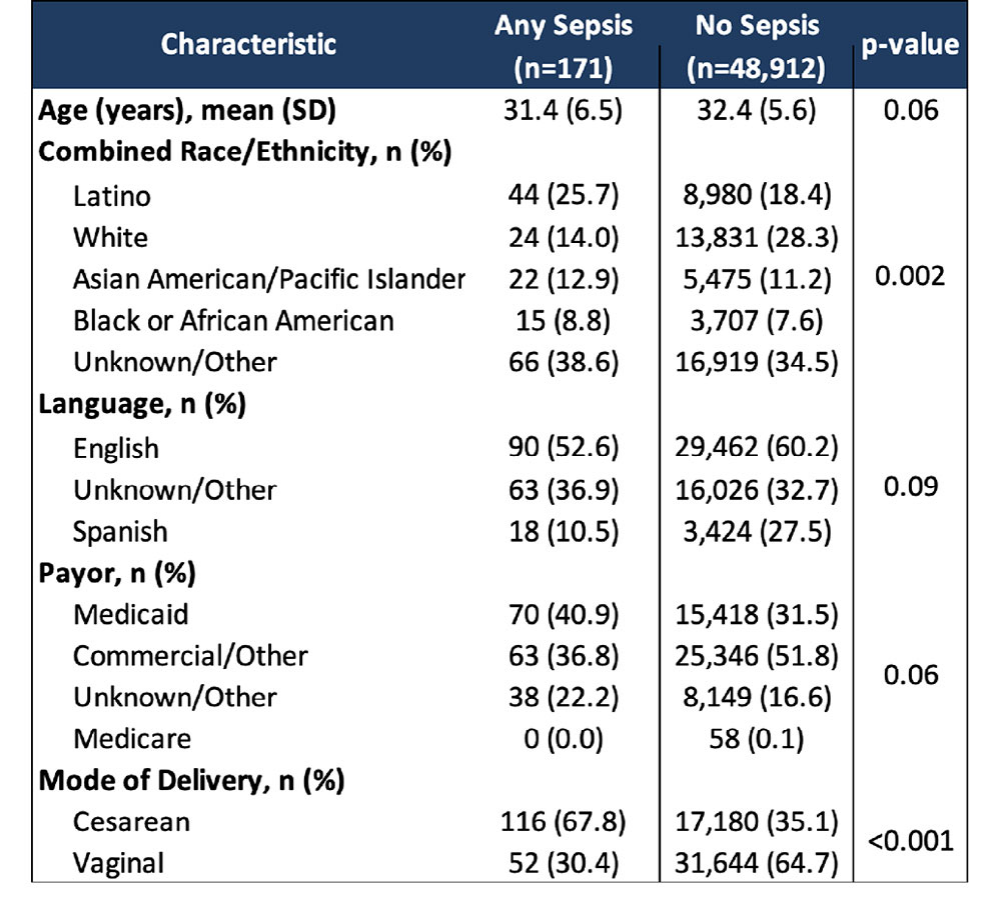

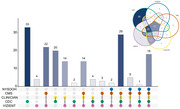

**3:30 PM – 5:00 PM**

## 514 | **Severity of** Excess **Gestational Weight Gain and Dyadic Outcomes in Normal BMI Pregnancies**

Isabella A. Molina^1^; Sarah H. Abelman^1^; Frank I. Jackson^1^; Oladunni Ogundipe^1^; Insaf Kouba^2^; Matthew J. Blitz^1^

^1^Northwell, New Hyde Park, NY; ^2^Johns Hopkins All Childrens, St. Petersburg, FL

**Objective**: To evaluate the association between excess gestational weight gain (EGWG) and maternal‐newborn dyadic outcomes using the desirability of outcome ranking (DOOR) methodology among patients with normal prepregnancy body mass index (BMI).

**Study Design**: This retrospective cross‐sectional study included all deliveries eligible for the Joint Commission's PC‐06 quality metric (term newborns without preexisting conditions) at seven hospitals within a large New York health system (2019–2024). Only each patient's first delivery was analyzed. Among patients with normal BMI, gestational weight gain was categorized as appropriate (≤35 lb per 2009 Institute of Medicine guidelines) or excess (>35 lb), and further classified as mild (36–44 lb), moderate (45–54 lb), or severe (≥55 lb). DOOR methodology grouped outcomes by delivery mode and presence of maternal or neonatal complications: (1) uncomplicated vaginal delivery, (2) uncomplicated cesarean, (3) vaginal delivery with complications, and (4) cesarean with complications. Multinomial logistic regression assessed the association between EGWG and DOOR category, adjusting for race and ethnicity, insurance, language, multiparity, and the obstetric comorbidity index (OB‐CMI).

**Results**: Of 43,502 patients, dyadic outcomes by EGWG category are shown in the Figure; regression results appear in the Table. Compared to those without EGWG, patients with mild, moderate, and severe EGWG had progressively higher adjusted odds of cesarean with complications (aORs 1.17, 1.37, 1.64), and uncomplicated cesarean (aORs 1.27, 1.49, 1.70). Severe EGWG was also associated with higher odds of vaginal delivery with complications (aOR 1.64; 95% CI, 1.27–2.11).

**Conclusion**: In pregnancies with normal BMI, EGWG is an independent predictor of worse maternal‐newborn outcomes. A clear dose‐response pattern was observed, even after adjustment for comorbidities and social determinants.

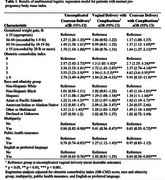

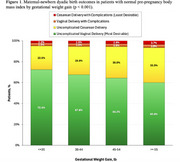

**3:30 PM – 5:00 PM**

## 515 | **Comparing** Severe **Maternal Morbidity Between Somali and Non‐Somali Black Birthing Individuals**

Isahaq Abdullahi^1^; Lensa Toka^2^; Connor Demorest^2^; Katelyn Tessier^2^; Cresta W. Jones^3^; Bethany A. Sabol^1^; Sarah A. Wernimont^1^

^1^University of Minnesota, Minneapolis, MN; ^2^University of Minnesota, University of Minnesota, MN; ^3^University of Minnesota, MInneapolis, MN

**Objective**: While Black pregnant individuals experience worse perinatal outcomes than White counterparts, outcomes within Black minority populations remain understudied. The heterogeneity of Black populations includes both descendants of enslaved Africans and recent immigrants from regions of the Black diaspora, who may have distinct health profiles. To address this gap and identify within‐group differences, we evaluated severe maternal morbidity (SMM) and neonatal outcomes among Somali immigrants—one distinct diaspora subgroup—compared to the broader Black population.

**Study Design**: In a retrospective cohort study from 2016–2024, we identified Somali and non‐Somali Black individuals within an academic‐community birth outcomes database using reported race, primary language (Somali), and country of origin documentation. Clinical characteristics including SMM using CDC's 21‐indicator criteria (excluding blood transfusions) and a neonatal morbidity composite were compared using chi‐square, Fisher's exact, and Student's t‐test as appropriate.

**Results**: Among 7373 births, 2425 (32.9%) were Somali and 4948 (67.1%) non‐Somali Black individuals. Differences in baseline characteristics included older age, higher multiparous rates, higher public insurance rates and lower epidural and C‐section rates among Somali compared to non‐Somali Black individuals (all *p* < 0.001). SMM during birth hospitalization was similar between groups, but significantly lower at 6 weeks postpartum in Somali compared to non‐Somali Black individuals (0.5% vs 1.0%, *p* = 0.021). However, after adjusting for parity, mode of delivery and insurance type, difference in 6‐week postpartum SMM was not statistically significant (aOR 0.57, 95% CI: 0.29–1.05). Composite neonatal outcomes were lower in Somali births compared to non‐Somali Black births (14.3% vs 17.1%, *p* = 0.002).

**Conclusion**: No differences in SMM were noted between Somali and non‐Somali birth populations. However, neonatal morbidity was lower in Somali populations. These findings challenge assumptions about immigrant health disparities within Black populations.

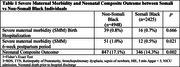

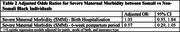

**3:30 PM – 5:00 PM**

## 516 | **Sonographic** Misclassification **of Fetal Growth and its Influence on Delivery Decisions: Time for Improved Accuracy**

Itai Atar^1^; Einat Tako^1^; Itamar Gilboa^2^; Daniel Gabbai^2^; Michael Shenhav^3^; yariv yogev^1^; Emmanuel Attali^2^

^1^Lis Hospital for Women's Health, Tel Aviv Sourasky Medical Center, Tel Aviv, Israel, Tel Aviv, Tel Aviv; ^2^Lis Hospital for Women's Health, Tel Aviv Sourasky Medical Center, Lis Hospital for Women's Health, Tel Aviv Sourasky Medical Center, Tel Aviv; ^3^Lis Hospital for Women's Health, Tel Aviv Sourasky Medical Center, Tel Aviv, Tel Aviv

**Objective**: To evaluate whether an estimated fetal weight (EFW) below the 10th percentile influences mode of delivery.

**Study Design**: A retrospective cohort study of all women who underwent sonographic EFW at 36–37 weeks, within 7 days prior to delivery, at a university‐affiliated tertiary center (2012–2025). Multiple gestations and non‐viable fetuses were excluded. EFW and birth percentiles were calculated using the Hadlock curve. Women were categorized into: (1) Estimated and born appropriate for gestational age (AGA, reference); (2) Estimated small for gestational age (SGA; ≤10th percentile) but born AGA; (3) Estimated AGA but born SGA; and (4) Estimated and born SGA. Outcomes were compared using univariate and multivariable analyses.

**Results**:

1. Of 147,045 deliveries, 21,089 (14.3%) met the study's criteria: 17,044 (80.8%) were estimated and born AGA, 311 (1.5%) estimated SGA but born AGA, 1826 (8.7%) estimated AGA but born SGA, and 1908 (9.0%) estimated and born SGA.
2. The average EFW‐to‐delivery interval ranged from 4.75 to 6.5 days. Sensitivity and specificity for diagnosing SGA were 51.1% and 98.0%, respectively.
3. Compared with the reference group, all other groups had higher induction rates (26.1–38.0% vs. 22.1%), longer postpartum stays (>5 days: 26.7–42.4% vs. 21.1%), and lower rates of gestational diabetes (7.6–14.5% vs. 19.5%) (all *p* < 0.001).
4. Operative vaginal deliveries, elective cesareans (CD), and CDs for non‐reassuring fetal heart rate (NRFHR) were more common in non‐reference groups (p≤0.04). SGA‐born groups had higher intrapartum CD rates (5.7–6.0% vs. 4.6%). Most CDs were due to malpresentation or NRFHR.
5. After adjusting for induction and other factors, none of the non‐reference groups independently predicted vacuum or intrapartum CD.

**Conclusion**: SGA estimation, regardless of actual birth weight, was associated with more inductions, earlier delivery, and CD for NRFHR, but did not independently predict vacuum or intrapartum CD. Overestimation of risk may lead to unnecessary interventions and higher maternal burden.

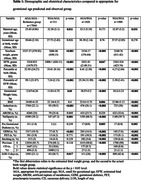

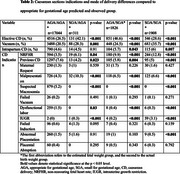

**3:30 PM – 5:00 PM**

## 517 | **Fetal Head** Station **at Second‐Stage Onset in Twin Pregnancies and Delivery Outcomes**

Itamar Gilboa^1^; Daniel Gabbai^1^; Dana Englander^1^; Itai Atar^2^; Yariv Yogev^3^; Emmanuel Attali^1^

^1^Lis Hospital for Women's Health, Tel Aviv Sourasky Medical Center, Lis Hospital for Women's Health, Tel Aviv Sourasky Medical Center, Tel Aviv; ^2^Lis Hospital for Women's Health, Tel Aviv Sourasky Medical Center, Tel Aviv, Israel, Tel Aviv, Tel Aviv; ^3^Lis Hospital for Women's Health, Tel Aviv Sourasky Medical Center Gray Faculty of Medicine, Tel Aviv University, Israel, Lis Hospital for Women's Health, Tel Aviv Sourasky Medical Center, Tel Aviv

**Objective**: Although fetal head station (FHS) at the onset of the second stage is associated with delivery outcomes in singletons, its role in twin gestations—where only the presenting twin's station is assessed—remains unclear. This study aimed to evaluate the association between FHS at second‐stage onset and mode of delivery in twin pregnancies.

**Study Design**: A retrospective cohort study was conducted at a single university‐affiliated center (2013–2024), including women with twin gestations ≥37 weeks, presenting vertex first twin, and planned trial of labor. Only the first twin was analyzed, as fetal head station was documented solely for the presenting fetus. Women were stratified by FHS at second‐stage onset: Group A ‐ above ischial spines (S <0), Group B ‐ at ischial spines (S = 0), and Group C ‐ below ischial spines (S >0).

**Results**:

1. Of 496 eligible twin pregnancies, 249 met inclusion criteria
2. Distribution by fetal head station: Group A–9.6%, Group B–30.1%, Group C–60.2%.
3. Cesarean delivery (CD) rates were significantly higher in Group A (12.5%) vs. Groups B and C (1.3% each, *p* < 0.001). Most CDs were due to arrest of descent (66%).
4. Vacuum‐assisted delivery was more common in Group A (25.0%) than in Groups B (12.0%) and C (9.3%) (*p* = 0.032).
5. Median second‐stage duration increased with higher station: Group C: 0.2 h (0.1–1.1), Group B: 0.5 h (0.2–1.4), Group A: 1.1 h (0.2–3.1) (*p* < 0.001).
6. No significant difference was observed in non‐vertex presentation of the second twin across groups (6.7–10.7%, *p* = 0.315).
7. On multivariable analysis, fetal head station above the ischial spines was independently associated with a reduced likelihood of spontaneous vaginal delivery (aOR 0.21, 95% CI 0.01–0.9; *p* = 0.030), even after adjustment for relevant maternal and labor‐related variables, including second‐stage duration.
8. Among nulliparous women, this association remained significant (aOR 0.10, 95% CI 0.01–0.9; *p* = 0.042).

**Conclusion**: In twin gestations, fetal head station above the ischial spines at the onset of the second stage is associated with prolonged second stage and lower rates of spontaneous vaginal delivery.

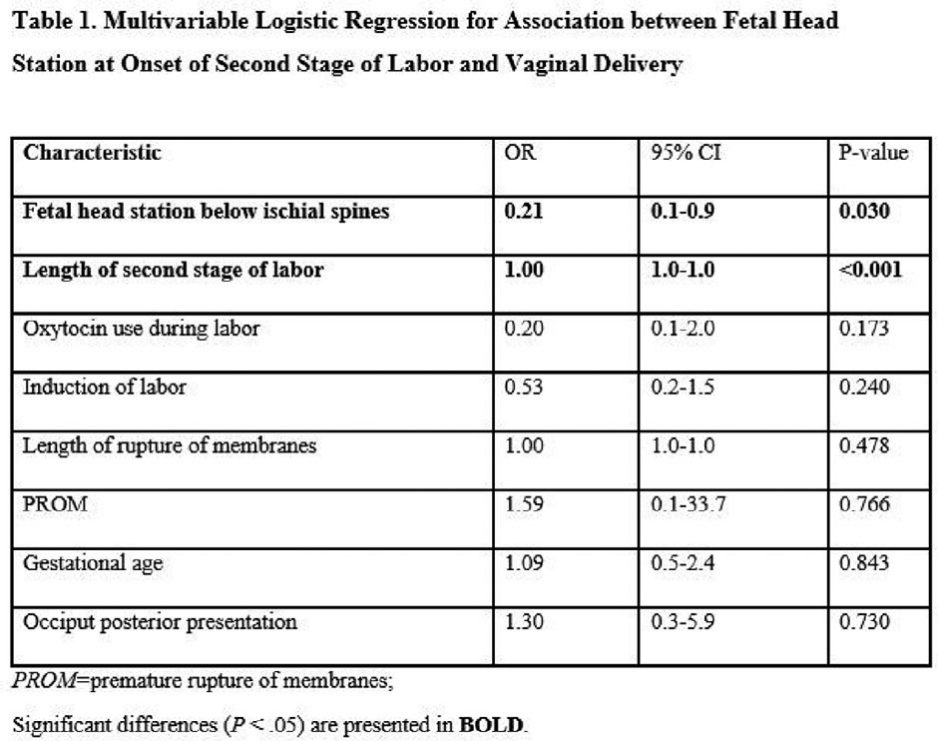

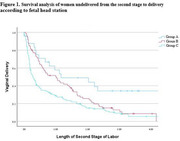

**3:30 PM – 5:00 PM**

## 518 | **Intrapartum** Fingerstick **vs. Continuous Glucose Monitoring in Patients with Type 2 and A2 Gestational Diabetes**

Jacqueline Rice; Ayesha Hasan; Lauren Larocca; Nicole Hsing‐Smith; Quetzal Class

University of Illinois at Chicago, Chicago, IL

**Objective**: Maintaining intrapartum maternal euglycemia decreases adverse neonatal outcomes. Continuous glucose monitoring (CGM) is a new tool that may be employed in lieu of standard fingerstick point‐of‐care (POC) measurements during labor. We examined differences in neonatal outcomes in patients with type 2 diabetes mellitus (T2DM) or medication‐controlled gestational diabetes (A2GDM) who used CGM versus POC glucose measurements for intrapartum monitoring.

**Study Design**: We performed a retrospective chart review of patients with T2DM or A2GDM by ICD‐10 who delivered between 9/2020–7/2024 at an urban academic hospital. Exclusion criteria were diet‐controlled (A1) gestational diabetes, type 1 diabetes, multifetal gestation, antenatal steroid administration within 14 days of delivery, non‐laboring (e.g. scheduled cesarean section), and nonviable pregnancies. We matched the POC group to CGM users by diabetes class, BMI, and age at delivery using randomized matching. Chi‐square and t‐tests were performed to evaluate maternal and fetal outcomes between groups.

**Results**: Of 80 patients who met inclusion criteria, 35% (*n* = 28) used CGM intrapartum while 65% (*n* = 52) were monitored with standard POC measurements. Race, ethnicity, and type of diabetes mellitus did not differ across groups. Mean glucose was higher (109.7 vs. 100.2 mg/dL; U = 970, *p* < 0.05) and time‐in‐range (70–110 mg/dL) lower (59.2; vs 72.1 percent; U = 518.5, *p* < 0.05) in the CGM group. Though neonatal hypoglycemia was rare (3.8%, *n* = 3), occurrences were not significantly different between groups (X2 (1) = 1.56, *p* = 0.21). Similarly, need for insulin drip during labor (X2 (1) = 0.30, *p* = 0.87) and maternal treatment of hypoglycemia (X2 (1) = 0.06, *p* = 0.89) were not significantly different. Neonatal outcomes including gestational age, birthweight, birthweight percentile, initial glucose measurement, and hospital length of stay did not differ by group.

**Conclusion**: Important clinical outcomes did not differ between CGM and POC glucose measurement groups during the intrapartum period for patients with A2GDM or T2DM.

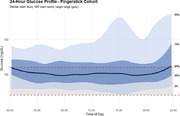

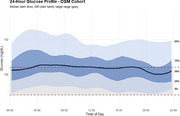

**3:30 PM – 5:00 PM**

## 519 | **Maternal and** Fetal **Adverse Reactions With Iron Infusions**

James Doss^1^; Emily Schwabe^2^; Nandini Raghuraman^3^; Amanda C. Zofkie^3^; Megan L. Lawlor^3^; Antonina I. Frolova^3^; Roxane Rampersad^3^; Jeannie C. Kelly^4^

^1^University of Vermont, University of Vermont, VT; ^2^BJC Healthcare, BJC Healthcare, MO; ^3^Washington University School of Medicine, St. Louis, MO; ^4^Washington University School of Medicine, Washington University School of Medicine, MO

**Objective**: As oral iron is poorly tolerated and inefficient, intravenous (IV) iron has become an increasingly common strategy to manage iron deficiency in pregnancy. While generally considered safe, emerging reports have resulted in FDA warnings regarding fetal bradycardia. We sought to characterize the maternal and fetal effects of adverse reactions observed during IV iron infusions at our institution.

**Study Design**: We reviewed all adverse drug reaction events in pregnancy during IV iron infusions from 2018–2024 using the institutional event reporting system. Data was extracted from electronic medical records and external fetal monitoring (EFM) tracings if performed; institutional policy utilizes continuous EFM during infusions in viable pregnancies. We evaluated the incidence of maternal reactions and compared characteristics between patients who did/did not experience EFM changes.

**Results**: Of 2994 infusions in pregnancy during the study period, 41 (1.4%) adverse maternal reactions occurred. Most occured with iron dextran (93%). The most common maternal symptoms were shortness of breath (59%) and chest pain/tightness (37%); the majority of patients had no prior history of drug or food allergies (61%). EFM decelerations were documented in 19 cases (46%). Between those with and without decelerations, there were no differences in age, race, gestational age, or prior allergies; there were also no differences in iron formulation, pre‐medication or test dose (Table). Most decelerations occurred within 5 minutes of infusion (68%) and all occurred 1–2 minutes after start of maternal symptoms. The median time required for resolution back to fetal baseline was 5 minutes (range: 1 to >10 minutes); 2 patients experienced prolonged fetal bradycardia unresponsive to resuscitation and delivered via emergent cesarean.

**Conclusion**: Maternal adverse events during IV iron transfusion may be associated with concerning EFM changes, regardless of iron formulation and premedication. Further large studies should assess whether continuous EFM during iron infusion is needed.

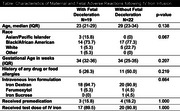

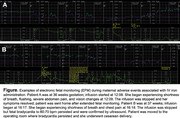

**3:30 PM – 5:00 PM**

## 520 | **Maternal** Vaccination **Behavior as Predictor of Neonatal Vaccine Acceptance**

James D. Toppin^1^; Talia Suner^1^; Brian W. James^1^; Joseph R. Biggio, Jr.^2^; Frank B. Williams^3^

^1^Ochsner Clinic Foundation, New Orleans, LA; ^2^Ochsner Health, New Orleans, LA; ^3^Ochsner Clinic Foundation, Ochsner Clinic Foundation, LA

**Objective**: The COVID‐19 pandemic represented a disruption event in vaccination uptake, with shifting public sentiment on vaccination. We sought to examine the association between maternal Tdap refusal and neonatal hepatitis B vaccine refusal while assessing rural‐urban geographic disparities in vaccination.

**Study Design**: We performed a retrospective analysis of 103,549 mother‐infant pairs from 2016 to 2024 undergoing prenatal and delivery care at a large regional health system in the American South. Multivariable logistic regression was used to assess maternal Tdap refusal as a predictor of neonatal hepatitis B refusal, controlling for insurance, age, delivery year, and COVID‐19 period. Rural‐Urban Commuting Area (RUCA) codes were used to stratify populations into urban, large rural, small rural, and isolated rural categories. Adjusted odds ratios (aOR) with 95% confidence intervals (CI) were generated.

**Results**: The mean patient age was 28.4 years (SD 5.8), 60.3% public insurance, and 41.1% Black. RUCA categorization from urban to most rural was 88.4%, 7.6%, 3.3%, and 0.7%, respectively. Overall maternal Tdap, neonatal hepatitis B, and concordant vaccination rates were 56.4%, 85.9%, and 61.9%, respectively. Maternal Tdap refusal strongly predicted neonatal hepatitis B refusal (aOR 3.13, 95% CI 3.02, 3.25). Substantial rural‐urban vaccination rate disparities were found, with urban areas achieving the highest concordant maternal‐neonatal vaccination rates at 53.7%, while rural areas demonstrated significantly lower rates: large rural 33.9%, small rural 38.7%, and isolated rural 35.6%. Maternal Tdap vaccination rates showed the largest geographic disparities, ranging from 58.8% in urban areas to 37.3% in large rural areas. Neonatal hepatitis B vaccination rates were more geographically consistent, but still showed urban advantages.

**Conclusion**: Maternal vaccination strongly predicts neonatal vaccine acceptance, with pronounced rural‐urban disparities in vaccination coordination. These findings highlight the need for targeted interventions in rural areas to improve both maternal and neonatal vaccination rates.

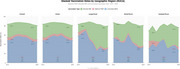

**3:30 PM – 5:00 PM**

## 521 | **Association** Between **Blood Product Refusal and Cesarean Delivery in Nulliparous Term Singleton Vertex Pregnancies**

Jamie Green; Sarah H. Abelman; Frank I. Jackson; Oladunni Ogundipe; Luis A. Bracero; Matthew J. Blitz

Northwell, New Hyde Park, NY

**Objective**: To determine whether there is an association between blood product refusal and cesarean delivery among nulliparous, term, singleton, vertex (NTSV) pregnancies. Obstetricians may have a lower threshold for surgical intervention to avoid hemorrhage risks that cannot be managed with transfusion, potentially leading to higher cesarean delivery rates even in low‐risk pregnancies.

**Study Design**: This retrospective cross‐sectional study included all NTSV deliveries at seven hospitals within a large New York health system from 2019–2024. Exclusion criteria were fetal demise and contraindication to labor. The primary exposure was blood product refusal, documented at admission via a standardized intake question for all patients. The primary outcome was cesarean delivery. Multivariable logistic regression modeled the association between blood product refusal and cesarean delivery, adjusting for race and ethnicity group, public health insurance, preferred language English, and obstetric comorbidity index (OB‐CMI) score, calculated from 24 weighted comorbidities identified via ICD‐10 codes and clinical notes.

**Results**: A total of 55,750 patients were included. Blood product refusal was documented in 358 cases and 115 identified as Jehovah's Witnesses. Non‐Hispanic White patients constituted the largest race and ethnicity group (41.9%), followed by Hispanic (17.1%), Asian or Pacific Islander (15.2%), and Non‐Hispanic Black patients (11.8%). Cesarean delivery occurred in 29.1% of patients who indicated they would decline blood products if needed, compared to 27.9% of patients without such a refusal (aOR 1.01; 95% CI, 0.80–1.28). Among Jehovah's Witnesses, the cesarean rate was 33.9% (aOR 1.16; 95% CI 0.77–1.71).

**Conclusion**: In this cross‐sectional study of NTSV patients, there was no difference in cesarean delivery rates between patients who declined blood products and those that did not.

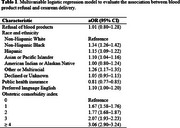

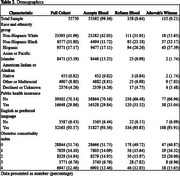

**3:30 PM – 5:00 PM**

## 522 | **Combined Effect of Adverse Pregnancy Outcome and Steatotic Liver Disease on Future Cardiovascular Disease Risk**

Jeesun Lee^1^; In Ae Cho^1^; Jaeyoon Jo^1^; Yujin Lee^1^; Manu Shivakumar^2^; Dokyoon Kim^2^; So Hee Kim^3^; Ji Hoi Kim^3^; Chan‐Wook Park^4^; Joong Shin Park^4^; Won Kim^5^; Seung Mi Lee^6^

^1^Gyeongsang National University Hospital, Jinju‐si, Kyongsang‐namdo; ^2^University of Pennsylvania, Philadelphia, PA; ^3^Seoul National University Hospital, Seoul, Seoul‐t'ukpyolsi; ^4^Department of Obstetrics and Gynecology, Seoul National University Hospital, Seoul National University College of Medicine, Seoul, Republic of Korea, Seoul, Seoul‐t'ukpyolsi; ^5^Seoul National Univeristy, Seoul, Seoul‐t'ukpyolsi; ^6^Department of Obstetrics and Gynecology, Seoul National University College of Medicine, Seoul, Seoul‐t'ukpyolsi

**Objective**: Adverse pregnancy outcome (APO) is an emerging risk factor for cardiovascular disease (CVD) in women. Steatotic liver disease (SLD) is a metabolic disorder that independently increases CVD risk. However, the combined effect of APO and SLD remains unclear, despite their shared metabolic and inflammatory pathways. Evaluating their joint effect may provide important information for targeted prevention strategies.

The goal of this study was to evaluate the combined impact of APO and SLD on CVD risk.

**Study Design**: This study utilized UK Biobank data, a large‐scale prospective cohort. Participants aged 40–69 were recruited between 2006 and 2010 and remain under follow‐up. Parous women without prior CVD were included. APO history was defined as any prior occurrence of preeclampsia, low birthweight, or gestational diabetes mellitus. SLD was defined according to the fatty liver index at enrollment (≥30). Participants were categorized into four groups based on APO history and SLD at enrollment: (1) APO(‐), SLD(‐) (reference), (2) APO(+), SLD(‐), (3) APO(‐), SLD(+), and (4) APO(+), SLD(+). The primary outcome was incident CVD after enrollment, which included conditions such as coronary artery disease, peripheral artery disease, ischemic stroke, heart failure, aortic stenosis, mitral regurgitation, atrial fibrillation, and venous thromboembolism. Adjusted hazard ratios (aHRs) for CVD risk were calculated using Cox models, adjusting for covariates.

**Results**: Each factor—APO history or SLD at enrollment—was independently associated with an increased risk of CVD. Women with both risk factors showed the highest CVD incidence. The combined presence of these exposures suggests a greater overall impact on CVD risk than either factor alone (Figure).

**Conclusion**: APO and SLD each confer increased long‐term CVD risk, and their coexistence further amplifies risk in an additive manner. These findings support incorporating obstetric history and metabolic markers into postpartum cardiovascular risk assessment to identify high‐risk women who may benefit from early intervention.

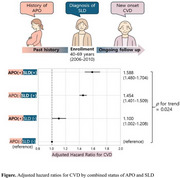

**3:30 PM – 5:00 PM**

## 523 | **Ptsd In** Pregnancy**: Maternal and Neonatal Outcomes**

Jessica Beeghly^1^; Bharti Garg^2^; Katherine Au^3^; Katherine Jorda^1^; Arundhati Headrick^4^; Aaron B. Caughey^2^

^1^Oregon Health & Science University, Portland, OR; ^2^Oregon Health & Science University, Oregon Health & Science University, OR; ^3^Oregon Health and science university, Portland, OR, OR; ^4^OHSU, OHSU, OR

**Objective**: While a diagnosis of post‐traumatic stress disorder (PTSD) has been associated with adverse perinatal mental health outcomes, the association between PTSD and other adverse perinatal outcomes remains less clear. High rates of maternal stress in pregnancy have been found to be associated with increased rates of preterm birth, gestational diabetes, preeclampsia, low birth weight, cesarean deliveries, and infant admission to neonatal intensive care units (NICUs), but data on the association between PTSD and perinatal outcomes remains limited. The objective of this study was to examine the association between a PTSD prior to birth and adverse outcomes in a contemporary cohort.

**Study Design**: This was a retrospective cohort study using linked vital statistics and hospital discharge data for births in California between the years of 2016–2020. We included pregnant individuals with PTSD with a non‐anomalous singleton gestation who delivered between 23 and 42 weeks. The presence of PTSD was identified using F43.10 PTSD, F43.11 PTSD acute, F31.12 PTSD chronic. Chi‐square tests and multivariable Poisson regression models were used to assess the incidence rates of maternal and neonatal outcomes in individuals with and without PTSD.

**Results**: Individuals with a history of PTSD had associations with higher incidences of hypertensive disease in pregnancy (IRR = 1.45; 95% CI = 1.38–1.53), preterm delivery <37 weeks (IRR = 1.29; 95% CI = 1.20–1.39), severe maternal morbidity (IRR = 1.54; 95% CI = 1.33–1.78), placental abruption (IRR = 1.29; 95% CI = 1.07–1.55), postpartum hemorrhage (IRR = 1.74; 95% CI = 1.61–1.89), and chorioamnionitis (IRR = 1.77; 95% CI = 1.61–1.94). Similarly, PTSD‐positive status was also associated with a higher incidence of NICU admission (IRR = 1.20; 95% CI = 1.13–1.28), respiratory distress syndrome (IRR = 1.40; 95% CI = 1.17–1.67), and hypoglycemia (IRR = 1.68; 95% CI = 1.52–1.87).

**Conclusion**: PTSD was associated with a number of adverse perinatal outcomes. These findings can be used both to counsel patients, but also to consider strategies to mitigate these risks.

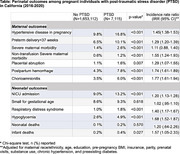

**3:30 PM – 5:00 PM**

## 524 | **Risk Factors for** Readmission **With Delayed Postpartum Preeclampsia: A Retrospective Case‐Control Study**

Jessica B. Hecht^1^; Grace Y. Lee^2^; Rachel Nordlicht^3^; Kavita Vani^4^

^1^Albert Einstein College of Medicine, Bronx, NY; ^2^Washington University School of Medicine, Barnes Jewish Hospital, Saint Louis, MO; ^3^Albert Einstein College of Medicine, The Bronx, NY; ^4^Albert Einstein College of Medicine/Montefiore Medical Center, Bronx, NY

**Objective**: To identify patient characteristics and risk factors for readmission for delayed postpartum preeclampsia (DPP).

**Study Design**: We conducted a retrospective cohort study of patients who delivered at Montefiore Medical Center between June 2018‐May 2022. Cases of DPP, defined as new‐onset preeclampsia diagnosed during a postpartum readmission between 2 days and 6 weeks after delivery in patients without a prior hypertensive disorder of pregnancy (HDP), were identified via ICD‐9/10 codes and verified by chart review. A random sample of 400 patients without HDP and without readmission was selected from the same cohort. Bivariate tests compared baseline characteristics. Multivariable logistic regression assessed factors associated with DPP readmission, adjusting for age, race, body mass index (BMI), insurance status, delivery mode, and delivery timing in relation to the COVID‐19 pandemic.

**Results**: Of 17,711 deliveries, 114 patients (0.64%) were readmitted with DPP. Compared to controls, patients with DPP were more likely to be older (36.9 ± 5.8 vs. 33.3 ± 5.5 years, *p* < 0.01), identify as Black (47.4% vs. 27.8%, *p* < 0.01), undergo cesarean section (48.2% vs. 31.7%, *p* < 0.01), and deliver post‐pandemic (29.8% vs. 18.8% *p* = 0.04). In adjusted analyses, age ≥ 35 years, (aOR 3.79, 95% CI 2.25 ‐ 6.38, *p* < 0.01), Black race (aOR 2.19, 95% CI 1.35–3.56, *p* < 0.01), and post‐pandemic delivery (aOR 2.58 95% CI 1.40–4.77, *p* < 0.01) were significantly associated with readmission for DPP. BMI, insurance status and delivery mode were not associated with DPP readmission.

**Conclusion**: Older age, Black race, and post‐pandemic delivery were independently associated with DPP readmission. Findings highlight the need for targeted discharge education and follow‐up. The post‐pandemic rise in DPP readmissions may reflect improved recognition of DPP due to shifts in care delivery in response to the COVID‐19 pandemic.

**3:30 PM – 5:00 PM**

## 525 | **Evaluating the** Impact **of Threatened ICE Activity on Maternal‐Fetal Medicine Clinic Attendance**

Jessica Montgomery^1^; Anna Madden‐Rusnak^1^; Vaishnavi V. Kankate^1^; Shreyaa Ganesh^1^; Keila Gavia^1^; Jeny Ghartey^1^; Alison G. G. Cahill^2^; Lorie M. Harper^1^

^1^Department of Women's Health, Dell Medical School at the University of Texas at Austin, Austin, TX; ^2^Department of Women's Health, Dell Medical School at the University of Texas at Austin, Department of Women's Health, Dell Medical School at the University of Texas at Austin, TX

**Objective**: The 1/21/25 executive order allowed US Immigration and Customs Enforcement (ICE) raids to occur in sensitive areas, including hospitals, where prior statute largely barred this practice. We aimed to see if this change impacted in‐person attendance in our Maternal‐Fetal Medicine (MFM) clinic.

**Study Design**: This retrospective cohort study included scheduled in‐person MFM visits at a quaternary care center in central Texas for two 60‐day periods: Jan 21‐Mar 20 in 2024 (pre) and 2025 (post). Clinic‐indicated cancellations were excluded. No‐show rates are reported by year, ethnicity (Hispanic/Latino vs. non‐Hispanic/Latino), and primary language (English vs non‐English). A multivariable logistic regression tested associations between no‐show status and year, ethnicity, and language. A post‐hoc logistic regression examined rates of missing demographic data across years.

**Results**: A total of 8040 appointments (show and no‐show) were included. In 2024, 2397 patients across 4442 appointments had a no‐show rate of 15%. In 2025, 2446 patients across 3598 appointments had a no‐show rate of 18%. No‐show rates in 2024 were similar between non‐Hispanic (15%) and Hispanic patients (14%) and odds of no‐show for all ethnicity categories were not significantly different (all *p* > 0.05). In 2025, no‐show rates for Hispanic patients increased by 63% but only 28% for non‐Hispanic, reflecting significantly higher odds of no‐show for Hispanic patients compared to non‐Hispanic in this year (aOR = 1.39, 95% CI: 1.00–1.87, *p* = 0.047). There was no significant difference in no‐show odds for patients with unknown ethnicity compared to those with known in 2025 (*p* = 0.48). The odds of non‐reported ethnicity or primary language increased dramatically in 2025 from 2024 (both *p* < 0.001).

**Conclusion**: There was a significant increase in no‐show rates for MFM appointments after the Executive Order permitting ICE raids to occur in hospitals. These findings highlight how immigration policy impacts healthcare access by introducing barriers to prenatal care, potentially increasing disparities among vulnerable populations.

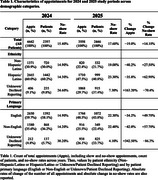

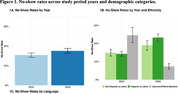

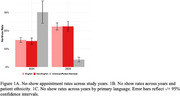

**3:30 PM – 5:00 PM**

## 526 | **Congenital** Syphilis **Risk Following Inadequate Treatment of Late Latent or Unknown Duration Syphilis in Pregnancy**

Jessica Sisco^1^; Shreya Battu^1^; Abigail B. Clark^2^; Heath Yancey^1^; Lorre MacDonald^3^; Allison Kurzeja^1^; Jessica E. Pruszynski^1^; Emily H. Adhikari^1^

^1^University of Texas Southwestern Medical Center, Dallas, TX; ^2^University of Texas Southwestern Medical Center, University of Texas Southwestern Medical Center, TX; ^3^Parkland Health, Dallas, TX

**Objective**: Data are sparse on the actual risk of congenital syphilis (CS) following inadequate treatment of maternal latent syphilis.

**Study Design**: This is a retrospective cohort study of pregnant patients diagnosed with late latent syphilis (late or unknown duration) who received at least one dose of benzathine penicillin G (BPG) before delivery between January 2010 and June 2025. We excluded individuals reporting any treatment prior to pregnancy. Inadequate treatment was defined as (1) fewer than three total BPG doses, (2) receipt of initial BPG dose <30 days before delivery, or (3) lack of at least 2 sequential dosing intervals of <10 days. We compared demographics, syphilis characteristics and CS outcomes among those with inadequate vs adequate treatment of late latent syphilis during pregnancy.

**Results**: Of 362 individuals with late latent syphilis, 51(14.1%) received inadequate treatment and 311(85.9%) received adequate treatment before delivery. Inadequately treated individuals had fewer prenatal visits (4 vs 11, *p* < 0.001) and later EGA at syphilis diagnosis (23 vs.14 weeks, *p* < 0.001). Inadequately treated individuals received the first BPG dose at a later EGA than adequately treated (27 vs. 15.7 weeks, *p* < 0.001). Serologic response (fourfold RPR drop) occurred in 11 (22%) with inadequate vs 110 (35%) with adequate treatment (*p* = 0.05). Neonates born to mothers who received inadequate syphilis treatment delivered earlier (38 vs. 39 weeks, *p* = 0.01). CS was higher among inadequately treated (OR 2.16, 95% CI: 0.87–4.93) but effect was not statistically significant. Neonates born to inadequately treated mothers were significantly more likely to receive 10 days IV penicillin. Stratified by initial RPR titer, CS risk increased with maternal RPR group, but risks were imprecise and not statistically significant.

**Conclusion**: While the great majority of gravidas with latent syphilis are adequately treated, data on congenital syphilis risk following inadequate treatment of late latent syphilis are relatively weak. Refinement of clinical criteria for determining the highest risk neonates could be considered.

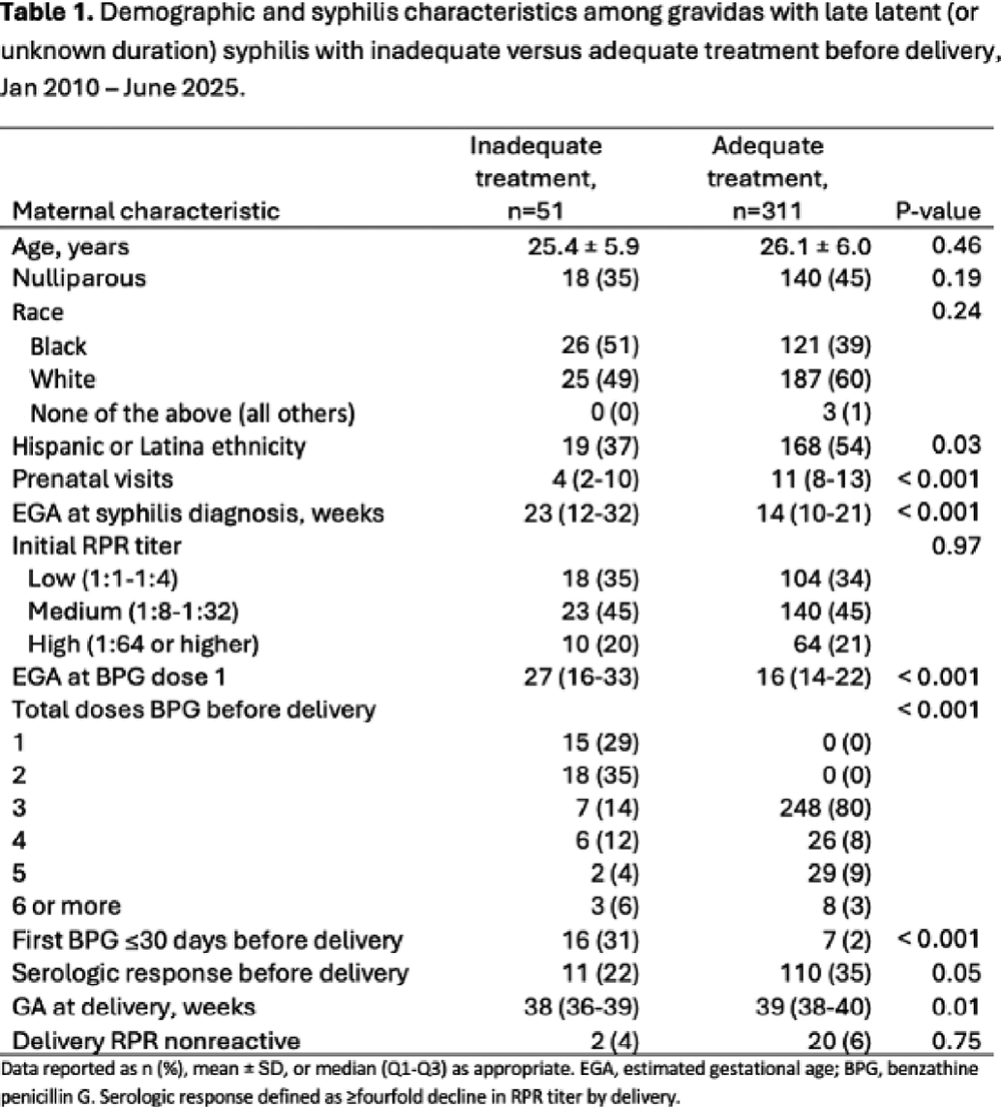

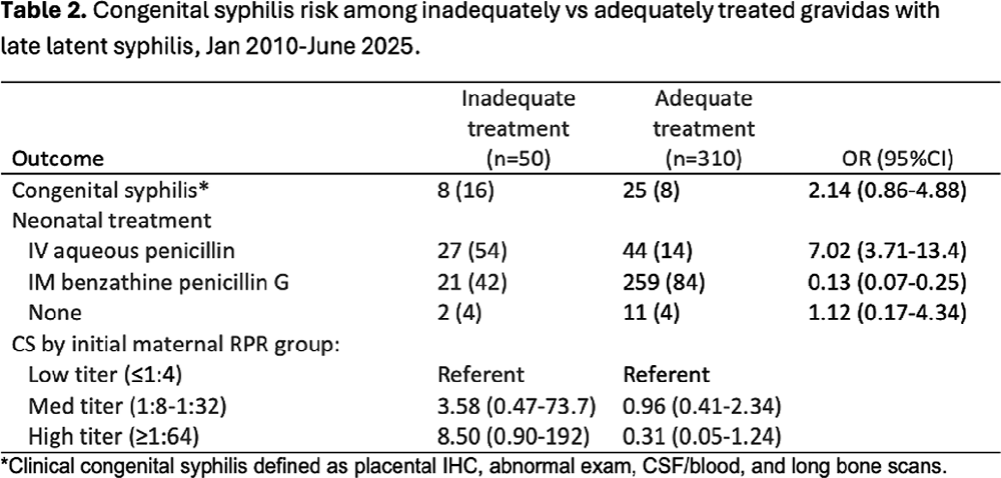

**3:30 PM – 5:00 PM**

## 527 | **Digital Based** Management **System (MomStart) for Diabetes in Pregnancy: Improving Perinatal Outcomes Through Technology‐Based Care**

Elisa Gi Soo Um^1^; Byung Soo Kang^2^; Hyun Sun Ko^2^; Seon Ui Lee^3^; Jin Yu^4^; Jae‐Seung Yun^5^; Kyu‐Ho Kim^5^; Youn‐Ju Lee^6^; Yoon‐Hee Choi^7^

^1^Department of Obstetrics and Gynecology, Seoul St. Mary's Hospital, College of Medicine, The Catholic University of Korea, Seoul, Korea, Seoul, Seoul‐t'ukpyolsi; ^2^Department of Obstetrics and Gynecology, Seoul St. Mary's Hospital, College of Medicine, The Catholic University of Korea, Seoul, Seoul‐t'ukpyolsi; ^3^Department of Obstetrics and Gynecology, Incheon St. Mary's Hospital, College of Medicine, The Catholic University of Korea, Incheon, Inch'on‐jikhalsi; ^4^Department of Endocrinology and Metabolism, Seoul St Mary's Hospital, College of Medicine, The Catholic University of Korea, Seoul, Seoul‐t'ukpyolsi; ^5^Department of Internal Medicine, St Vincent's Hospital, College of Medicine, The Catholic University of Korea, Suwon, Kyonggi‐do; ^6^PH‐DATA Co,, Ltd, Seoul, Korea., Seoul, Seoul‐t'ukpyolsi; ^7^Department of Endocrinology and Metabolism, Seoul St Mary's Hospital, College of Medicine, The Catholic University of Korea, PH‐DATA Co,, Ltd, Seoul, Korea., Seoul, Seoul‐t'ukpyolsi

**Objective**: Diabetes during pregnancy poses a growing public health burden, affecting both perinatal outcomes and long‐term health of mothers and offspring. This study aimed to evaluate the impact of a digital‐based management system—including the *MomStart* mobile application and an educational website—on pregnancy outcomes in women with diabetes.

**Study Design**: We compared outcomes between a prospective digital care cohort and a retrospective traditional care cohort (control group) using 1:3 propensity score matching based on age, parity, and pre‐pregnancy BMI.

**Results**: After matching, 91 women in the digital care group and 273 in the control group were included. The digital care group had higher rates of family history of diabetes, alcohol use before pregnancy, and office‐based employment, compared with control group. However, systolic blood pressure in the second and third trimesters, diastolic blood pressure in the third trimester, and cesarean section rates were significantly lower. Although mean gestational age at delivery was similar, and the digital care group showed higher rates of pre‐existing hypertension and insulin use, it had significantly lower rates of preeclampsia, threatened preterm labor, PPROM, preterm birth, and postpartum hemorrhage. Neonatal outcomes of NICU admission, phototherapy for jaundice, and hypoxic‐ischemic injury were also significantly reduced in the digital care group when compared with control group. Composite neonatal adverse outcomes occurred in 39.6% of the digital care group vs. 59.3% in controls (*p* = 0.001).

**Conclusion**: Digital care in pregnancies with diabetes was associated with improved maternal and neonatal outcomes. This approach may offer an effective strategy for managing high‐risk pregnancies. This study was supported by the National Institute of Health (No. 22ER080800) and registered with Korea's Clinical Research Information Service system (KCT0008483).

**3:30 PM – 5:00 PM**

## 529 | **How Does** ChatGPT **Compare to MFM Specialists in Diagnosing PAS?**

Joe Haydamous^1^; Edgar A. Hernandez‐Andrade^2^; Laura Diab^2^; Baha M. Sibai^2^; Farah H. Amro^2^

^1^Department of Obstetrics, Gynecology and Reproductive Sciences, McGovern Medical School at UTHealth Houston, Department of Obstetrics and Gynecology, McGovern Medical School at UT Health, Houston, TX; ^2^Division of Maternal‐Fetal Medicine, Department of Obstetrics, Gynecology and Reproductive Sciences, McGovern Medical School at UTHealth Houston, Houston, TX

**Objective**: ChatGPT (Generative Pre‐trained Transformer) is a deep‐learning technology with potential healthcare applications. We evaluated its diagnostic performance for placenta accreta spectrum (PAS) on ultrasound (US) versus maternal‐fetal medicine (MFM) specialists, using pathology as the gold standard, and examined performance across transabdominal (TA), transvaginal (TV), and combined (TA+TV) imaging.

**Study Design**: This retrospective study included patients referred for suspected PAS who underwent US at our institution. All non‐PAS cases had placenta previa; PAS cases included previa and non‐previa. US images and video clips were reviewed by MFM specialists and ChatGPT using standardized prompts, blinded to outcome. Interpretations were classified as PAS present (yes/no) and subtype. Pathology was the reference standard. Agreement was assessed with Cohen's kappa (κ). Sensitivity, specificity, PPV, NPV, and accuracy were calculated. McNemar's test compared modalities.

**Results**: Thirty cases were evaluated; 13 (43.3%) were non‐PAS. Among PAS cases (*n* = 17), MFMs showed substantial agreement with pathology (κ = 0.798,*p* < 0.001); ChatGPT showed moderate agreement (κ = 0.609,*p* < 0.001). MFMs and ChatGPT had substantial mutual agreement (κ = 0.627,*p* < 0.001). MFMs sensitivity and accuracy exceeded ChatGPT (88.2% vs. 68.6% and 90.0% vs. 80.0%), though specificity was high for both (>92%). ChatGPT correctly identified 12/13 non‐PAS placenta previa cases (specificity 92.3%) with an NPV of 80.0%, supporting its potential as a rule‐out tool. Sensitivity improved from 47.1% with TA alone to 76.5% with TV and 82.4% with TA+TV, with accuracy rising from 70.0% to 83.3% and 86.7%. No modality differences were statistically significant (all *p* >0.05) but trends favored TA+TV and TV.

**Conclusion**: While MFM specialists outperformed ChatGPT in sensitivity and accuracy, ChatGPT's high specificity and NPV support exploring its possible role to aid in the community setting. While not statistically significant, performance overall improved with the addition of transvaginal imaging, underscoring its value in evaluating the PAS patient.

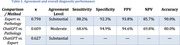

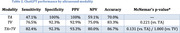

**3:30 PM – 5:00 PM**

## 530 | **Accounting for** Time **From Labor Onset Improves the Detection of Hypoxic‐Ischemic Encephalopathy in Labor**

Johann Vargas‐Calixto^1^; Yvonne Wu^2^; Michael W. Kuzniewicz^3^; Marie‐Coralie Cornet^2^; Aditi Lahiri^3^; Lawrence Gerstley^3^; John Parker^4^; Philip Warrick^4^; Robert Kearney^5^

^1^Emory University, Atlanta, GA; ^2^UCSF, San Francisco, CA; ^3^Kaiser Permanente, Oakland, CA; ^4^Perigen Inc, Cary, NC; ^5^McGill University, Montreal, PQ

**Objective**: Hypoxic‐ischemic encephalopathy (HIE) may result from events during pregnancy and delivery, often resulting in lifelong morbidity or mortality. Early detection of fetuses at risk of intrapartum HIE using cardiotocography (CTG) is still challenging. We aimed to determine if accounting for the time from labor onset (TLO) in a machine learning classifier would improve the early detection of HIE.

**Study Design**: This retrospective study used CTG recordings from a cohort of 37,522 deliveries, including 342 HIE cases. We developed and compared two random forest classifiers: a time‐independent (TI) model using 86 CTG features, and a time‐dependent (TD) model which added the TLO as an additional feature. The CTG features were calculated from 20‐minute CTG segments. Also, both models were tested for the entirety of labor, which could last up to 72 hours. The decision threshold was selected such that the false‐positive rate (FPR) of both models matched the observed emergency Caesarean delivery (ECD) rate in the healthy population.

**Results**: Both models yielded the same FPR equal to the ECD rate in the healthy group (27.3%). The TD model was superior in HIE detection: it detected 3% more HIE cases than the TI model one hour before delivery (55.9% vs 52.9%, *p* = 0.0002); and it also detected more HIE cases than their ECD rate (55.9% vs 44.7%). This advantage was more pronounced for earlier detection; the TD model identified 5.3% more cases than the TI model three hours before delivery (43.5% vs 38.2%, *p* < 0.0001). Also, the TD model had a median detection time of 6.0 h before delivery compared to 5.3 h in the TI model (*p* = 0.0083).

**Conclusion**: Our results demonstrate that the performance of a machine learning classifier for the detection of HIE can be significantly improved by the addition of a single feature: the TLO. This indicates that providing a classifier with temporal context increases the early detection rate of HIE without increasing its FPR. Such early detection could allow timely clinical interventions that have the potential to improve neonatal outcomes.

**3:30 PM – 5:00 PM**

## 531 | **Outcomes of IVF Versus Non‐IVF Conceived Monochorionic‐Diamniotic Twin Gestations**

Johanna A. Suskin^1^; Erica Glaser^1^; Annika Hsu^2^; Morgan Steelman^3^; Lois Brustman^1^; Simi Gupta^1^; Jennifer Lam‐Rachlin^4^; Andrei Rebarber^5^; Nathan S. Fox^1^

^1^Icahn School of Medicine, Mount Sinai West, New York, NY; ^2^Mount Sinai, New York, NY; ^3^Icahn School of Medicine at Mount Sinai Hospital, Icahn School of Medicine at Mount Sinai Hospital, NY; ^4^Icahn School of Medicine at Mount Sinai, Icahn School of Medicine at Mount Sinai, NY; ^5^Icahn School of Medicine, Mount Sinai West, Mount Sinai West, NY

**Objective**: This study aims to compare outcomes of monochorionic‐diamniotic (MD) twin pregnancies conceived with in vitro fertilization (IVF) with those conceived by other means, including spontaneous conception or ovulation induction.

**Study Design**: A single‐center retrospective cohort study was conducted amongst all MD twin pregnancies between 2005 and 2025 in an urban academic medical center. Pregnancies involving twin‐twin transfusion syndrome (TTTS), uterine anomalies, major fetal anomalies, and fetal losses prior to 20 weeks were excluded. For the preeclampsia (PEC) outcome, we excluded patients with chronic hypertension (cHTN). SPSS was used to perform descriptive and comparative statistics. A significance level of 0.05 was used.

**Results**: A total of 83 IVF and 160 non‐IVF MD pregnancies were included. The IVF group was significantly older (37.2 ± 5.9 vs. 31.8 ± 5.8, *p* < 0.001) than the non‐IVF group. There was significantly higher baseline cHTN among the IVF group (4.8% vs. 0.6%, *p* = 0.029). No significant difference was noted in pre‐pregnancy BMI.

On univariate analysis, IVF was significantly associated with an increased rate of PEC spectrum diagnosis (25.3% vs. 11.9%, *p* = 0.009), gestational diabetes (20.5% vs. 6.9%, *p* = 0.002), and cesarean delivery (CD; 78.3% vs. 45.6%, *p* < 0.001). After controlling for maternal age and BMI, IVF was independently associated with PEC (aOR 2.49, 95% CI 1.14, 5.37) and CD (aOR 2.97, 95% CI 1.55, 5.69), but not gestational diabetes (aOR 2.15, 95% CI 0.88, 5.33).

IVF was not significantly associated with gestational age at delivery, preterm birth, placental abruption, birthweight, fetal growth restriction, birthweight discordancy, APGAR scores, NICU admission, or fetal demise.

**Conclusion**: Among MD pregnancies, IVF is significantly and independently associated with increased rates of PEC and CD. This is amongst the largest studies of MD twins examining IVF as a risk factor that excludes TTTS/other major anomalies.

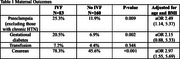

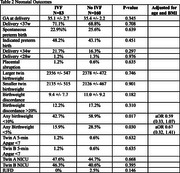

**3:30 PM – 5:00 PM**

## 532 | **It Takes Two: An** Initiative **To Increase Partner Carrier Screening Rates**

Joselle O'Brien^1^; Eliana Wachstock^2^; Emily Schlussel Markovic^1^; Itamar Futterman^3^; Robyn Weiss^1^; Rodney A. McLaren, Jr^1^; Scott Chudnoff^1^; Sandra McCalla^1^; Shoshana Haberman^1^; Tirtza Spiegel Strauss^1^

^1^Maimonides Medical Center, Brooklyn, NY; ^2^Yeshiva University, New York, NY; ^3^Maimonides Medical Center, Maimonides Health, NY

**Objective**: Expanded carrier screening (ECS) has increased due to its performance in diverse populations. However, there are low rates of partner screening in publicly insured patients. We implemented multiple interventions to increase partner follow up. We compared rates of partner screening before and after these interventions.

**Study Design**: This was an IRB approved retrospective cohort study at an academic hospital clinic. From 1/1/2024–2/28/2024, interventions were implemented, specifically, ECS panel was standardized to 113 genes recommended by American College of Medical Genetics, providers were educated, and free concurrent partner screening was offered via blood draw or prepaid saliva kit. The primary outcome was the rate of partner screening in patients who were heterozygous for autosomal recessive (AR) pathogenic variants six months before (7/1/2023–12/31/2023) and six months after (3/1/2024–8/31/2024) the intervention period. Secondary outcomes included percentage of patients who were heterozygous for AR or X‐linked pathogenic variants with standardized ECS, time from patient to partner result, and percentage of couples that carried the same AR pathogenic variant. Descriptive and comparative statistics were used.

**Results**: There were 444 patients; 156 pre‐intervention and 288 post‐intervention (demographics are in Table 1, results are in Table 2). In patients who were heterozygous for AR variants, partner screening rates significantly increased post‐intervention (49% vs 71%, *p* < 0.01). With standardized ECS, 50.0% (144/288) of patients were heterozygous for pathogenic variants. The median time from patient to partner result significantly decreased post‐intervention (32 vs 7 days, *p* < 0.01). Detection of carrier couples increased post‐intervention (0% vs 2.43%, *p* = 0.04).

**Conclusion**: Standardizing ECS panels, educating providers, and offering free concurrent partner screening increased partner screening rates to 71%, reduced time to partner results by 25 days, and detected more carrier couples. These interventions support equitable access to genetic screening in publicly insured populations.

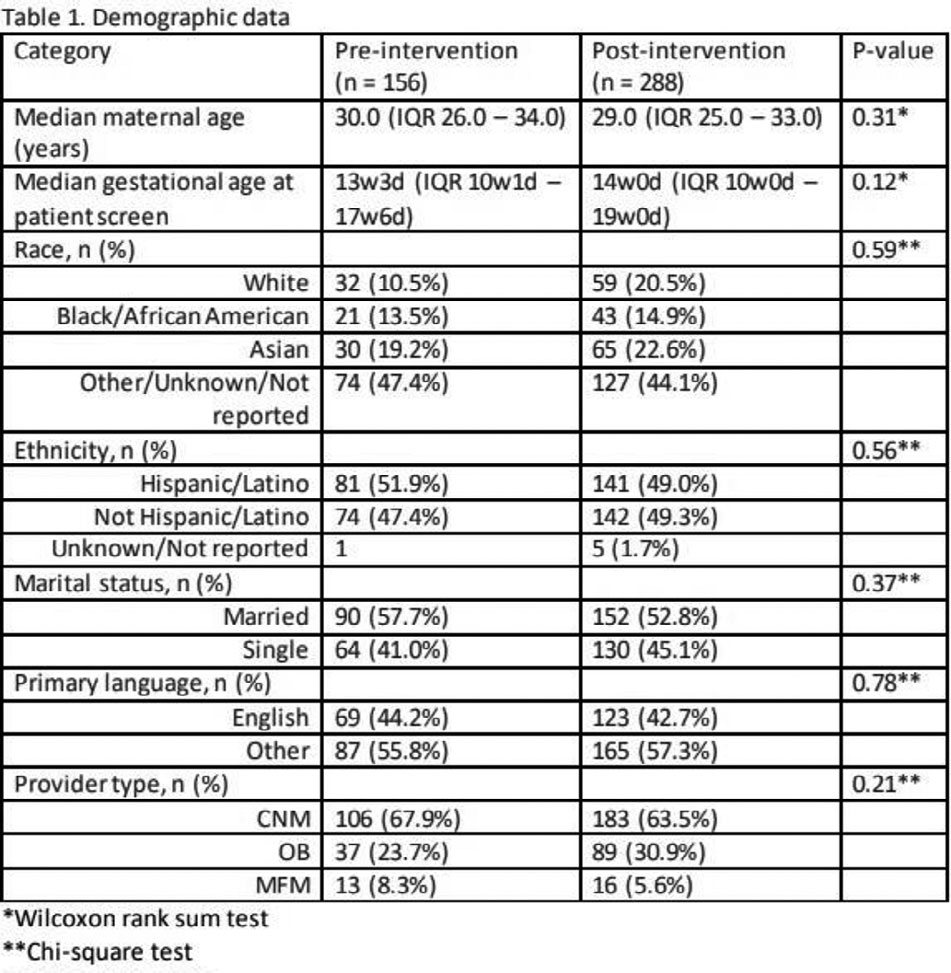

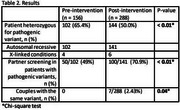

**3:30 PM – 5:00 PM**

## 533 | **Women With** Higher**‐Order Cesareans and Risks of TOLAC (WHOTOLAC)**

Joshua Brunton; Edward H. Springel

Virginia Commonwealth University, Richmond, VA

**Objective**: To evaluate whether the risk of uterine rupture is increased in patients undergoing trial of labor after cesarean (TOLAC) with ≥3 prior cesarean deliveries, and to compare associated maternal and neonatal outcomes to elective repeat cesarean delivery (ERCD).

**Study Design**: We conducted a retrospective cohort study using U.S. Vital Statistics Natality data from 2014 to 2023. We included singleton, nonanomalous live births at 34–42 weeks to women aged 18–50 with a history of ≥3 prior cesareans. Patients were grouped by delivery approach: TOLAC or ERCD.

**Results**: Among 442,049 eligible patients, 20,614 underwent TOLAC and 421,435 underwent ERCD. The TOLAC cohort was matched similarly in age (32.28 vs. 32.33 years; *p* = 0.142) and BMI (32.47 vs. 32.50 kg/m^2^; *p* < 0.001). Gravidity was higher in the TOLAC group (5.54 vs. 5.30; *p* < 0.001), with a similar number of prior cesareans (3.29 vs. 3.30; *p* = 0.865). Gestational age at delivery was slightly earlier (37.84 vs. 37.95 weeks; *p* < 0.001). TOLAC patients were more often non‐Hispanic (71.4% vs. 67.5%; *p* < 0.001). Uterine rupture was more common with TOLAC (*n* = 62 RR 1.75, 95% CI: 1.35–2.27; *p* < 0.001), but transfusion (RR 0.78) and hysterectomy (RR 0.70) were less likely. ICU admission was similar (RR 0.89; *p* = 0.264), while chorioamnionitis was more frequent (RR 3.37; *p* < 0.0001). Neonatal outcomes were worse with TOLAC, including higher rates of NICU admission (RR 1.11), ventilation, antibiotic use (RR 1.34), and seizures (RR 2.19; *p* = 0.0017).

**Conclusion**: Among patients with ≥3 prior cesarean deliveries, TOLAC was associated with a significantly higher risk of uterine rupture compared to ERCD, though overall rates remained low. Interestingly, TOLAC was also associated with lower risks of transfusion and hysterectomy. Despite these maternal findings, TOLAC was linked to higher rates of neonatal morbidity, including NICU admission, respiratory support, and antibiotic use. These data highlight the complex risk‐benefit considerations when counseling patients with higher‐order cesareans on the choice between TOLAC and ERCD.

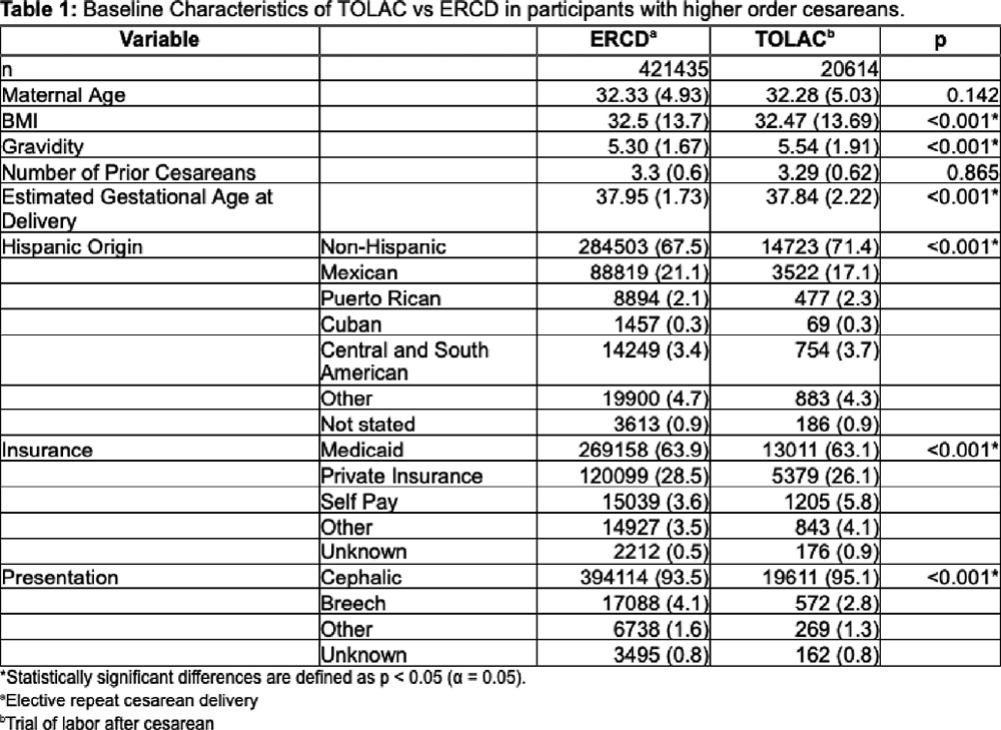

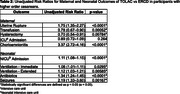

**3:30 PM – 5:00 PM**

## 534 | **Impact of Opioid use Disorder on Oxytocin Required for Labor Induction in Term Pregnancies**

Hansika Kunduru^1^; Judy Jiang^2^; Hannah Hill^3^; Jessica Pippen^4^

^1^Case Western Reserve University, Case Western Reserve University, OH; ^2^Case Western Reserve University/MetroHealth Medical Center, Cleveland, OH; ^3^MetroHealth Medical Center, The MetroHealth System, OH; ^4^MetroHealth Medical Center, Cleveland, OH

**Objective**: Oxytocin is a neuropeptide proven to impact human labor, lactation and social affinity and is of interest in the study of pain and μ‐opioid receptor modulation. Individuals impacted by opioid use disorder (OUD) frequently have μ‐opioid receptor dysregulation. In this study we sought to investigate the effect of OUD and the use of medications for opioid use disorder (MOUD) on the effectiveness of synthetic oxytocin in term inductions of labor.

**Study Design**: Retrospective case‐control study of individuals with term pregnancies undergoing induction of labor with oxytocin from 2020–2024 at an urban safety‐net hospital. The total amount of oxytocin required to achieve vaginal delivery and total induction of labor time were compared between those with OUD and those without. The groups were matched by age, parity, gestational age, month of delivery, and body mass index. Wilcoxon rank‐sum tests were used to compare numeric variables between two groups.

**Results**: 211 (106 without OUD and 105 with OUD) individual vaginal deliveries were evaluated in the study. There was no significant difference in total oxytocin dose used in induction between patients with OUD and those who did not have OUD. However, patients with OUD spent an estimated 66 minutes longer in induction after amniotomy than those without OUD (269 minutes versus 335 minutes (*p* = 0.015)). Patients on MOUD spent 76 minutes longer in induction after amniotomy than those not on MOUD (269 minutes vs 345 minutes (*p* = 0.007)). No statistically significant correlation was found between MOUD dose and oxytocin dose.

**Conclusion**: Individuals with OUD were not found to have significantly different total required oxytocin dose to delivery than those without OUD. However, patients with OUD, particularly those on MOUD, experienced significantly longer induction time following amniotomy compared to those without OUD. Further studies are needed to further examine the impact of OUD on induction of labor with oxytocin.

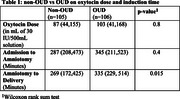

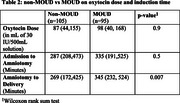

**3:30 PM – 5:00 PM**

## 535 | **Utilization of GPT for Enhanced Safety and Quality Review in Healthcare**

Julia Kim^1^; Isabella Harnick^2^; Karyn Wat^2^; Erika Banks^1^; Genevieve Sicuranza^1^; Hye J. Heo^1^

^1^NYU Grossman Long Island School of Medicine, Mineola, NY; ^2^NYU Langone Hospital ‐ Long Island, Mineola, NY

**Objective**: The purpose of root cause analysis (RCA) in healthcare is to systematically investigate adverse events or near misses and identify root causes. This allows for targeted interventions to prevent future occurrences, improving patient safety and overall care quality. Yet individual review of cases may miss opportunities to identify common themes to help prioritize and direct intervention. The advent of artificial intelligence (AI) technologies, such as Generative Pre‐trained Transformers (GPT), offers a promising avenue. In 2023, NYU Langone launched a licensed, HIPAA compliant GPT‐4. This study evaluated the feasibility and utility of GPT as a tool to enhance safety and quality review processes within healthcare organizations.

**Study Design**: A retrospective analysis evaluated event summaries of RCA cases with delay in timely delivery of category II tracings from 2020–2025 using the NYU GPT ecosystem. The model was tasked with identifying common themes and suggesting risk reduction strategies. The AI‐based analysis was compared with traditional review methods. Fisher's exact test was used with *p* < 0.05.

**Results**: GPT‐4 was given 11 case summaries and 8 prompts, including adjustments to prioritize themes or provide interventions. Approximately 30 minutes was used to prompt and review responses from GPT while >8 hours were spent on manual review. GPT recognized that 1 of 11 cases did not pertain to category II tracings as it was incorrectly included in the initial query. It identified 7 main themes that contributed to delay in delivery in the setting of category II tracings. Comparison of GPT to manual review revealed similarities in outcomes.

**Conclusion**: GPT accurately evaluated a small cohort of RCA cases, identifying recurrent themes and objective areas of improvement, in significantly less time than manual review. Integration of GPT into safety and quality review processes can be a feasible tool to identify and prioritize interventions that can be applied to more common RCA themes within larger datasets and across multiple specialties.

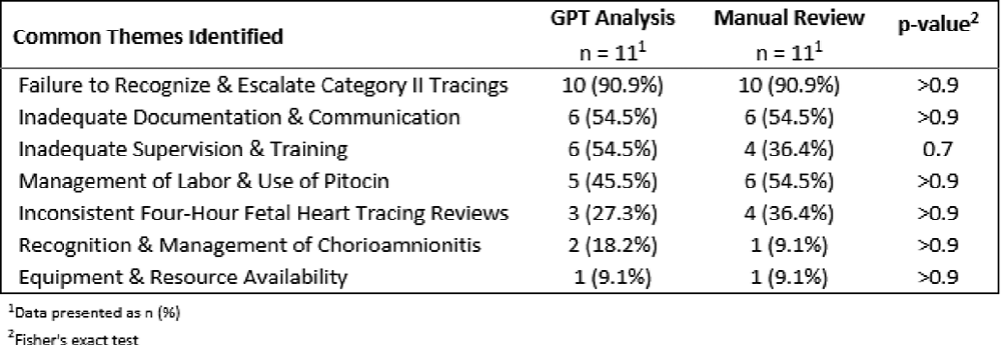

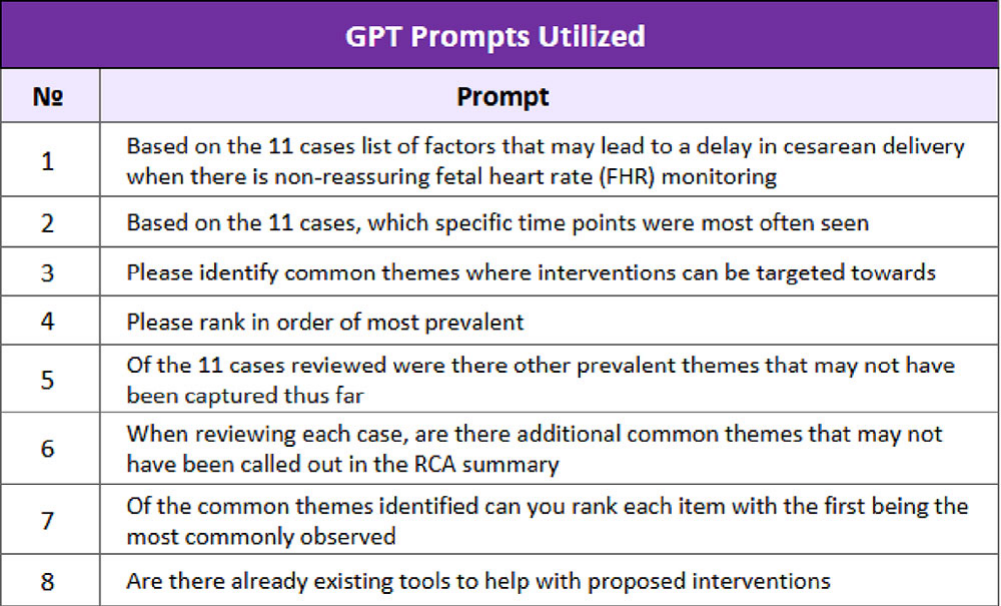

**3:30 PM – 5:00 PM**

## 536 | **Characteristics** and **Outcomes of Selective Fetal Growth Restriction With Intermittent Absent End Diastolic Flow**

Juliana S. Gebb^1^; Alekhya Jampa^2^; Violetta Bakunina^2^; Yunliang Zhao^3^; Kiersten Barr^2^; Federica Bellusi^2^; Desiree Fiorentino^2^; Suzanne E. DeBari^2^; Beverly G. Coleman^4^; Nahla Khalek^2^; Shelly Soni^2^

^1^Richard D. Wood Jr Center for Fetal Diagnosis and Treatment, Children's Hospital of Philadelphia, Children's Hospital of Philadelphia, PA; ^2^Richard D. Wood, Jr Center for Fetal Diagnosis and Treatment, Children's Hospital of Philadelphia, Children's Hospital of Philadelphia, PA; ^3^Richard D. Wood, Jr Center for Fetal Diagnosis and Treatment, Children's Hospital of Philadelphia, Philadelphia, PA; ^4^Richard D. Wood, Jr Center for Fetal Diagnosis and Treatment and Department of Radiology, Children's Hospital of Philadelphia, Children's Hospital of Philadelphia, PA

**Objective**: To describe characteristics of selective fetal growth restriction (sFGR) pregnancies with umbilical artery (UA) intermittent absent end diastolic flow (iAEDF) and compare outcomes in expectantly managed patients with iAEDF vs those with all forward flow (+EDF; Gratacos Type I), no forward flow (A/REDF; Gratacos Type II) and intermittent forward, absent, & reversed flow (iAREDF; Gratacos Type III).

**Study Design**: Retrospective analysis of prospectively collected data from a single center monochorionic (MC) pregnancy registry. Records of patients with MC twins with sFGR evaluated 1/2013–12/2024 were reviewed. Patients with Twin Twin Transfusion Syndrome, Twin Anemia Polycythemia Sequence, & structural malformations were excluded. sFGR was defined by the Delphi criteria. Pregnancies were stratified by the UA of the FGR fetus: 1) +EDF; 2) iAEDF; 3) A/REDF; or 4) iAREDF. Pregnancy characteristics were compared, and outcomes were then compared in expectantly managed patients using Kruskall‐Wallis, Mann‐Whitney U, chi square, and Fisher's exact test. *p* < 0.008 was statistically significant due to multiple comparisons.

**Results**: Of 332 patients with sFGR, 115 (34.6%) had +EDF, 91 (27.4%) had iAEDF, 70 (21.1%) had A/REDF and 56 (16.9%) had iAREDF. Overall, gestational age (GA) at consult was lower in those with A/REDF compared to iAREDF (*p* = 0.007) and fetal intervention was higher in patients with iAEDF, A/REDF and iAREDF compared to those with +EDF (all *p* < 0.001). In expectantly managed patients (*n* = 196), median GA at delivery (weeks) was higher in patients with +EDF (32.5) & iAEDF (32.3) compared to A/REDF (27.4) & iAREDF (29.1) (+EDF vs iAEDF, *p* = 0.160; +EDF vs A/REDF, *p* < 0.001; +EDF vs iAREDF, *p* < 0.001; iAEDF vs A/REDF, *p* = 0.004; iAEDF vs iAREDF, <0.001; A/REDF vs iAREDF, *p* = 0.632). Total survivors to discharge were higher in those with +EDF compared to A/REDF (*p* < 0.001) but not compared to iAEDF (*p* = 0.280) or iAREDF (*p* = 0.150).

**Conclusion**: In our cohort, patients with iAEDF had similar survival to patients with A/REDF and iAREDF but delivered at a later GA that was similar to those with +EDF.

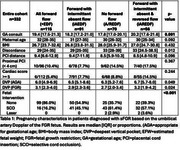

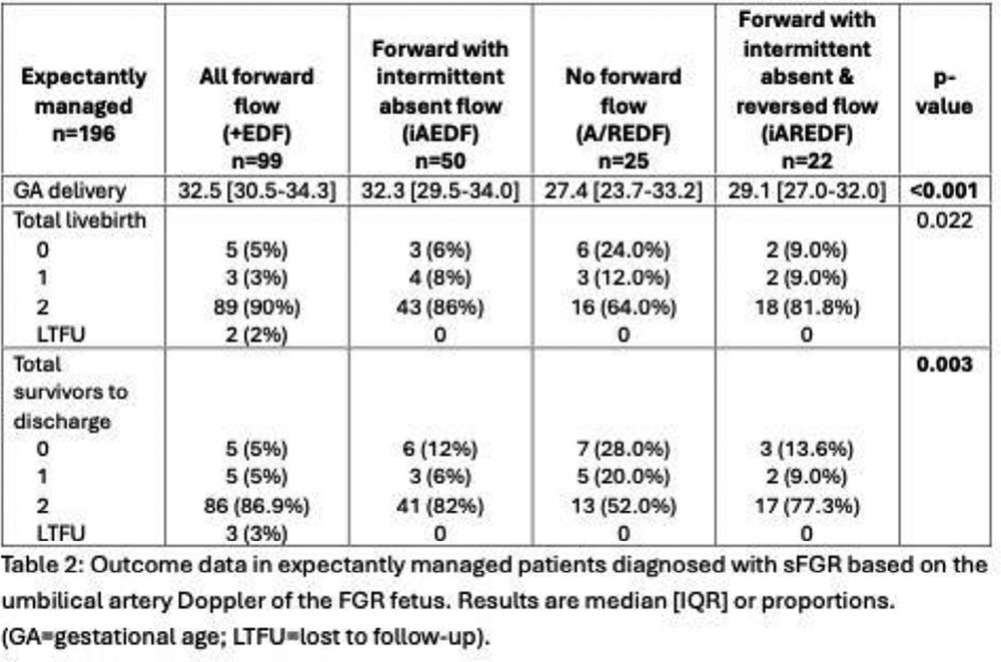

**3:30 PM – 5:00 PM**

## 537 | **Cell‐Free‐DNA** Based **Tests for Prenatal Diagnosis of Fetal Anomalies: What is the Diagnostic Yield?**

Julianna Fischer^1^; Rachel Veazey^1^; Emily Hardisty^1^; Kelly L. Gilmore^1^; Amy Motolla^1^; Ginger Hocutt^1^; Madeline Dyke^1^; Andrea Johnson^1^; Neeta L. Vora^1^; Asha N. Talati^2^

^1^University of North Carolina at Chapel Hill, Chapel Hill, NC; ^2^University of North Carolina at Chapel Hill, University of North Carolina at chapel Hill, NC

**Objective**: To identify diagnostic yield of whole genome cell free DNA (cfDNA) for prenatal diagnosis of fetal anomalies and compare to diagnostic yield of direct DNA testing.

**Study Design**: Retrospective review of pregnancies between 2020 to present that had MaterniTGenome™ sent at a quaternary care referral center. Participants were identified through the institution's LabCorp Portal. All participants received genetic counseling per institutional guidelines. Charts were reviewed for participant demographics, indication for testing including ultrasound findings and type(s) of fetal anomaly, types of test sent and results, and neonatal testing. Primary outcomes include: (1) frequency in which cfDNA based assessment provided a diagnosis, (2) correct diagnostic yield corroborated by direct DNA. Descriptive statistics were performed using STATA18.

**Results**: 108 patients had a MaterniTGenome test (Table 1). Of those, 86 (79.6%) had testing due to an ultrasound detected fetal anomaly, and 49 (45%) had testing for single gene disorders via cfDNA (Vistara™). 8 patients had positive MaterniTGenome™ results and 2 had positive Vistara™ results. Of the 8 with positive results, 4 (50%) had true positives by direct DNA and 1 (12.5%) had a false positive; 3 patients were lost to follow up. Notably, 2 of 4 with true positives had a diagnosis of Trisomy 21. 13 patients had diagnostic testing after a negative MaterniTGenome™: amniocentesis (8), CVS (1), cord blood (2), products of conception (2). Of those, 5 (38.5%) received a genetic diagnosis based on further direct DNA testing, and 8 (61.5%) had testing that confirmed true negative results (Table 2). The sensitivity of MaterniTGenome™ was calculated to be 44.4% compared to diagnostic yield of invasive testing.

**Conclusion**: CfDNA based assays offer an alternative to invasive diagnostic testing in the setting of fetal anomalies, but with significant limitations compared to direct fetal DNA assessment. This should be further evaluated in larger cohorts to determine best practice and counseling for use of cfDNA in these settings.

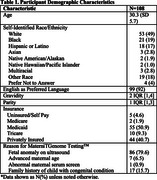

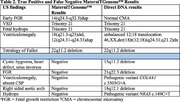

**3:30 PM – 5:00 PM**

## 538 | **Association of** Maternal **and Cord Plasma Leptin Levels With Fetal Adiposity Across Gestation**

Junko Tamai; Satoru Ikenoue; Keisuke Akita; Koki Shigematsu; Naotsugu Ishikawa; Yasuhiko Ogata; Yushi Abe; Yuka Fukuma; Yuya Tanaka; Toshimitsu Otani; Marie Fukutake; Yoshifumi Kasuga; Mamoru Tanaka

Department of Obstetrics and Gynecology, Keio University School of Medicine, Shinjuku‐ku, Tokyo

**Objective**: Fetal fat deposition during gestation has been associated with infant adiposity, which subsequently leads to childhood obesity and metabolic disorders. Maternal and cord leptin (one of the adipokines that influence lipid metabolism) are known to affect birth weight and infant adiposity. However, the association between leptin and fat deposition in the fetal period remains unclear. This study aimed to investigate the association of maternal and cord plasma leptin with fetal adiposity across gestation.

**Study Design**: A prospective study was conducted with 113 singleton pregnancies. Maternal blood sample was obtained at 10, 24 and 36 weeks, and cord blood sample was obtained at delivery. Plasma leptin level was log transformed to be normally distributed. Fetal ultrasonography was performed at 24, 30 and 36 weeks. Estimated fetal adiposity (EFA) was calculated as the average of standardized z scores of arm percentage fat area, thigh percentage fat area and anterior abdominal wall thickness. Multiple linear regression analyses were performed to examine the association between maternal or cord plasma leptin levels and EFA at 24, 30 and 36 weeks, adjusting for confounding factors including maternal age, parity, pre‐gravid BMI, gestational weight gain, and infant sex.

**Results**: Maternal plasma leptin at 10, 24, 36 weeks and cord plasma leptin were 18.3 ± 11.5 (median ± IQR), 19.5 ± 13.5, 21.4 ± 17.7, and 7.9 ± 6.9 ng/ml, respectively. Maternal leptin at 10 weeks was not associated with EFA across gestation. Maternal leptin at 24 and 36 weeks and cord leptin were not associated with EFA at 24 weeks and 30 weeks, but significantly correlated with EFA at 36 weeks. Even after controlling for the confounding factors, maternal leptin at 24 weeks (β = 0.474, *p* < 0.001), at 36 weeks (β = 0.456, *p* < 0.001) and cord leptin (β = 0.408, *p* < 0.001) significantly correlated with EFA at 36 weeks.

**Conclusion**: Maternal plasma leptin at 24 and 36 weeks and cord leptin significantly correlated with EFA at 36 weeks. Maternal leptin levels after 24 weeks could be a marker for identifying increased fetal adiposity in late gestation.

**3:30 PM – 5:00 PM**

## 539 | **Twin‐Twin** Transfusion **Syndrome After Fetoscopic Laser Photocoagulation: Survival Outcomes According to Gestational Age at Delivery**

Kamran Hessami^1^; Brian A. Burnett^1^; Ingmar N. Bastian^1^; Rebecca M. Johnson^1^; Jessian L. Munoz^2^; Cara Buskmiller^3^; Roopali V. Donepudi^4^; Magdalena Sanz Cortes^4^; Michael A. Belfort^1^; Ahmed A. Nassr^4^

^1^Baylor College of Medicine, Houston, TX; ^2^Texas Children's Hospital, Texas Children's Hospital, TX; ^3^Baylor College of Medicine, Austin, TX; ^4^Baylor College of Medicine, Baylor College of Medicine, TX

**Objective**: To evaluate 30‐day neonatal survival across gestational age (GA) at delivery in pregnancies affected by twin‐twin transfusion syndrome (TTTS) treated with fetoscopic laser photocoagulation (FLP).

**Study Design**: We conducted a retrospective cohort study of TTTS pregnancies that underwent FLP between 2012 and 2024 at a tertiary fetal center. Pregnancies were stratified by GA at delivery: 23–25⁶, 26–27⁶, 28–29⁶, 30–31⁶, 32–33⁶, 34–35⁶, and ≥36 weeks. Outcomes included (i) survival at delivery and (ii) 30‐day neonatal survival, limited to liveborn infants. Survival rates were reported per bracket.

**Results**: Among 562 pregnancies, dual survival at delivery occurred in 70.5% and single survival in 19.9% (Figure 1). Among liveborn neonates, 30‐day survival of at least one neonate improved significantly with advancing gestational age—from 60.5% at 23–25⁶ weeks to ≥98% in all brackets ≥28 weeks. Fetal loss was most pronounced at the earliest gestations, with 39.5% of fetuses lost by delivery in the 23–25⁶ week group. In contrast, nearly all fetuses survived to birth beyond 28 weeks. Dual 30‐day survival peaked at 32–33⁶ weeks (85.3%) and declined thereafter. At ≥36 weeks, single survival predominated (64.9%) due to continued gestation in pregnancies with prior single fetal demise (Table 1).

**Conclusion**: In TTTS pregnancies treated with FLP, the highest risk of fetal loss occurs at early gestational ages. Among liveborn infants, 30‐day survival improves substantially with increasing gestational age, reaching near‐universal survival from 28 weeks onward. However, dual survival is highest between 30–33⁶ weeks and declines at ≥36 weeks, likely due to selective continuation of pregnancies after one twin is lost. These findings support the consideration of late‐preterm delivery to optimize outcomes in surviving twins.

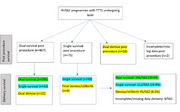

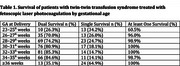

**3:30 PM – 5:00 PM**

## 540 | **Pathogenic and** Likely **Pathogenic Copy Number Variants Detected by Chromosomal Microarray in Isolated Fetal Ventriculomegaly**

Katarzyna B. Gajewska‐Knapik^1^; Alexandros Moraitis^1^; Magdalena Szybka^1^; Ruiling Xu^1^; Fionnuala Mone^2^; Gordon CS Smith^3^

^1^Department of Obstetrics & Gynaecology, Cambridge University Hospitals, Cambridge, UK, Department of Obstetrics & Gynaecology, Cambridge University Hospitals, Cambridge, UK, England; ^2^Centre for Public Health, Queen's University Belfast, Belfast, UK, Centre for Public Health, Queens University Belfast, Belfast, UK, Northern Ireland; ^3^University of Cambridge, Cambridge, England

**Objective**: Rich data exist on genomic changes in fetuses with non‐isolated structural abnormalities. However, for isolated ventriculomegaly (VM) they are still inconsistent and heterogenous, making parental decision difficult regarding further invasive testing.

The aim of this project was to perform qualitative and quantitative assessment of the current evidence on the prevalence of pathogenic and likely pathogenic (P/LP) copy number variants (CNVs) in fetuses with apparently isolated VM and normal karyotype.

**Study Design**: A systematic review of studies reporting on the prevalence of P/LP CNVs detected by chromosomal microarray (CMA) in fetal isolated VM from inception to December 2024 was conducted. Synthesis was performed separately for data on all isolated VM ≥10 mm and for data on non‐severe isolated VM ≥10 mm and ≤15 mm. For quantitative assessment the extracted results were pooled in a meta‐analysis of proportions and presented using forest plots. Heterogeneity was assessed using Higgins’ I2. PROSPERO CRD42021279656

**Results**: Twenty two studies incorporating in total *n* = 2999 cases with isolated VM and normal karyotype (CNVs >10Mb excluded) were included in the final meta‐analyses. For all isolated VM ≥10 mm (*n* = 1967) the incremental yield of P/LP CNVs was 5% (95% CI 3–7%; I2 = 69.06%) (Figure 1.), and for non‐severe isolated VM (≥10 mm and ≤15 mm; *n* = 1561) the incremental yield of P/LP CNVs was 3% (95% CI 1–5%; I2 = 66.5%) (Figure 2). Synthesis was attempted in mild (≥10 mm and ≤12 mm) and severe (>15 mm) VM groups but ultimately not performed due to the lack of adequate data.

**Conclusion**: This study provides the clarity for counselling patients with fetal isolated VM and isolated non‐severe VM. Original separate data is scarce for mild and severe VM groups. While severe VM is likely to have a guarded prognosis even in cases where genetic cause is not found, having more specified data on the prevalence of genetic changes in mild VM (≥10 mm and ≤12 mm) would have the potential to transform the counselling in Fetal Medicine setting. Further original data need to be collected to be able to advise patients accordingly.

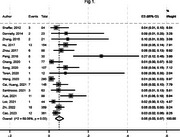

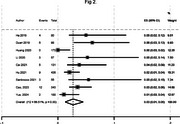

**3:30 PM – 5:00 PM**

## 541 | **School of** POWHER **Training Impact on Traditional Birth Attendant Motivation and Client Empowerment in Kenya**

Kate Tolleson^1^; Calder Hollond^2^; Augustine Kakuko^3^; Saula Abraham^3^; Dorothy Pilakan^3^; Miriam Petakwang^3^; Irene Chemtai^3^; Reham Perry^4^; Jessica Oliveira^5^; Taraneh Shirazian^6^; Sasha Hernandez^7^

^1^New York University Grossman School of Medicine, NYU Grossman School of Medicine, NY; ^2^New York University Grossman School of Medicine, New York, NY; ^3^Saving Mothers, Kapenguria, Western; ^4^Saving Mothers, New York, NY; ^5^Saving Mothers, Miami, FL; ^6^Department of Obstetrics and Gynecology, New York University Grossman School of Medicine, New York, NY; ^7^New York University Grossman School of Medicine, Department of Obstetrics and Gynecology, New York, NY

**Objective**: In rural Kenya, where maternal morbidity and mortality are high, traditional birth attendants (TBAs) often serve as the first point of care. The School of POWHER (SOP), led by a maternal health NGO, is a community‐based training program that aims to strengthen TBAs’ clinical knowledge, referral practices, and client‐centered care. We assessed TBA motivation and client‐reported trust and empowerment using adapted, validated community health worker (CHW) performance tools in two TBA cohorts in West Pokot, Kenya that were trained using the SOP model.

**Study Design**: Quantitative surveys were conducted with SOP‐trained TBAs (*n* = 15) and their clients (*n* = 17). TBAs completed the Multi‐Dimensional Motivation Scale, assessing supervision, value/capacity, peer support, and compensation. Clients completed three validated measures: Trust in CHWs, Client Empowerment in Community Health Systems (CE‐CHS), and CHW Influence on Empowerment. Composite scores were calculated using published protocols.

**Results**: TBAs exhibited moderate to high motivation (mean = 12.6; range:–1.1 to 24), with highest scores in perceived supervision and peer support. Clients reported high levels of trust in TBAs (mean = 3.81/4) and perceived them as positively contributing to their empowerment (mean = 3.74/4), particularly in navigating healthcare and promoting community well‐being. Overall client empowerment was also high, as reflected in the CE‐CHS score (mean = 3.25/4).

**Conclusion**: SOP‐trained TBAs in West Pokot, Kenya are highly trusted and contribute meaningfully to client empowerment and community‐based care. These findings offer evidence of implementation success and highlight motivation, trust, and empowerment as key benchmarks for TBA program evaluation. Insights from this evaluation will guide refinement of the SOP model, including structured peer mentorship, enhanced supervision, and strengthened referral support to better integrate community‐based services with formal health systems. By expanding access and quality in remote settings, this work supports global efforts to reduce maternal morbidity and mortality.

**3:30 PM – 5:00 PM**

## 542 | **Third‐Trimester** Biometric **Discordance Between AC and EFW and Risk of Adverse Outcomes**

Katelyn J. Rittenhouse^1^; Julie Johnson^2^; Yuri Sebastião^1^; Nicole Davis^1^; Rebecca Ritter^1^; Jeffrey S.A. Stringer^1^; Kim Boggess^1^

^1^University of North Carolina at Chapel Hill, Chapel Hill, NC; ^2^University of North Carolina ‐ Chapel Hill, Chapel Hill, NC

**Objective**: To evaluate the association between discordant abdominal circumference (AC) and estimated fetal weight (EFW) in the third trimester and the risk of large‐for‐gestational‐age (LGA) birth and associated complications.

**Study Design**: We assembled a retrospective cohort of singleton pregnancies with a growth ultrasound performed between 2017 and 2024 at a single academic center. We restricted the analysis to the first scan performed between 32w0d and 36w6d. EFW and AC percentiles were calculated using Hadlock standards. Fetuses were stratified into four groups: (1) EFW <90%/AC <90%, (2) EFW ≥90%/AC <90%, (3) EFW <90%/AC ≥ 90%, and (4) EFW ≥ 90%/AC ≥ 90%. Outcomes included LGA (birthweight ≥ 90% by Fenton), macrosomia (≥4000g), primary cesarean, and shoulder dystocia. Multivariable models estimated adjusted relative risks, controlling for maternal age, BMI, parity, and diabetes (pregestational and gestational), using the EFW <90%/AC <90% group as the reference.

**Results**: Among 12,781 pregnancies, 10% (*n* = 1,263) demonstrated discordance between EFW and AC percentiles (only AC or EFW ≥90%), while 15% (*n* = 1,863) had both ≥90%. Discordant biometry was associated with a 4–5‐fold increased risk of LGA and macrosomia and modestly increased risk of primary cesarean and shoulder dystocia. Notably, isolated AC≥90% conferred a >2‐fold higher shoulder dystocia risk, even in the absence of large EFW. Concordant elevation (EFW and AC≥90%) was associated with >10‐fold higher risk of LGA and 5‐fold higher risk of shoulder dystocia. These patterns were consistent among both diabetic and non‐diabetic patients.

**Conclusion**: Third‐trimester discordance between AC and EFW ≥90% is associated with increased risk of adverse perinatal outcomes. While discordance occurred in only 10% of pregnancies, it resulted in more than twice the rate of LGA and shoulder dystocia compared to the concordant normal group. These associations held true regardless of glycemic status. These findings support incorporating AC% into third‐trimester counseling, even when EFW alone does not meet the≥90% threshold.

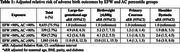

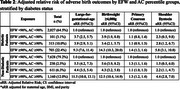

**3:30 PM – 5:00 PM**

## 543 | **Predicting** Gestational **Weight Gain Using Hybrid Weight Sources and Machine Learning**

Kathan Vyas^1^; Timothy Wen^2^; Mia Charifson^3^; Adesh Kadambi^1^; Shreyas Kadambi^1^; Isabel Fulcher^1^; Sara M. Sauer^4^

^1^Delfina Care Inc, San Francisco, CA; ^2^University of California San Diego, San Diego, CA; ^3^Delfina Care Inc, Delfina Care Co., CA; ^4^Delfina Care Inc, Brooklyn, NY

**Objective**: Excessive or inadequate gestational weight gain (GWG) are associated with increased risk of adverse maternal and neonatal outcomes. Our goal was to develop and validate a machine learning model for predicting GWG at term using clinical and self‐reported weight data through 24 weeks gestational age (wga).

**Study Design**: We analyzed pregnant individuals from four community clinics using a digital health platform (2021–2025), excluding preterm births, multiple gestations, and insufficient weight data. We combined electronic health records and self‐reported data to predict GWG category (inadequate, within range, excessive per IOM guidelines) using a gradient boosting classifier, trained with 5‐fold cross‐validation. Predictors included pre‐pregnancy body‐mass index (BMI), weight trajectory calculated as the slope between pre‐pregnancy and 16–24 weeks, maternal age, parity, chronic hypertension, and pregestational diabetes. We assessed model performance with area under the curve (AUC) and compared it across BMI and race categories using DeLong's test with Bonferroni correction.

**Results**: Among the 1088 included pregnancies, 287 (26%), 240 (22%), and 561 (52%) had adequate, inadequate, and excessive GWG, respectively. The final model achieved an overall AUC of 0.85 (95% CI: 0.80–0.87), with class‐specific AUCs of 0.76 (CI: 0.70–0.81) for “Within Range”, 0.90 (CI: 0.86–0.93) for “Inadequate,” and 0.90 (CI: 0.87–0.93) for “Excessive” (Figure 1). Performance was stable across BMI and race groups, with no statistically significant differences in AUC (all Bonferroni‐adjusted *p* >0.05).

**Conclusion**: Machine learning can be leveraged to construct pragmatic and parsimonious models to accurately predict GWG using clinical and self‐reported weight data prior to 24wga with limited bias across BMI or race groups. Early identification of pregnant individuals at risk of either excessive or inadequate GWG enables timely initiation of preventive interventions.

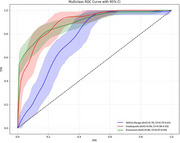

**3:30 PM – 5:00 PM**

## 544 | **The Impact of an Immediate Postpartum LARC Program on Short‐Interval Birth Rates in Tennessee**

Kaylee Ramage^1^; Megan L. Young^2^; Alicia Mastronardi^3^; Sam Havron^3^; Jill M. Maples^4^; Jordan E. Lewis^3^; Mary G. Herndon^3^; Katherine B. Kaak^2^; Nikki B. Zite^3^

^1^University of Tennessee, Knoxville, Knoxville, TN; ^2^University of Tennessee Medical Center, Knoxville, TN; ^3^University of Tennessee Graduate School of Medicine, Knoxville, TN; ^4^University of Tennessee Health Science Center COM Knoxville, Knoxville, TN

**Objective**: In Nov 2017, a Tennessee‐wide policy change (i.e., the immediate postpartum long‐acting reversible contraception (IPP LARC) program) occurred, which allowed reimbursement for IPP LARC among patients receiving Tennessee Medicaid; however, few hospitals adopted this program. To understand its impact, this study assessed rates of short‐interval births (SIB) among institutions who implemented the IPP LARC program compared to those who did not.

**Study Design**: Birth certificate data from Tennessee Medicaid patients who had a live birth between Jan 2015‐Dec 2021 were abstracted, excluding nulliparas. The 3 major hospitals who implemented the IPP LARC program were identified (1 in each of West, Middle, and East TN). We applied an interrupted time‐series design to assess the impact of the IPP LARC program implementation on average (avg) monthly rates of SIB at institutions who did and did not implement the program. The intervention effect was assessed at 9 months post‐implementation (Aug 2018). SIB was classified as a birth interval <24 months.

**Results**: Implementation of the IPP LARC program had differential impacts on SIB among the 3 hospitals who implemented this program. The hospital in West TN had higher than avg monthly SIB rates pre‐implementation (33%), compared to non‐implementing hospitals. They had an avg decrease of 0.16% of births classified as SIB/month (*p* = 0.046) post‐implementation (Figure 1). The pre‐implementation monthly SIB rates in the Middle and East TN implementation hospitals had less than avg SIB rates compared to non‐implementing hospitals (25.8% in Middle TN and 23.1% in East TN vs 28.6% in non‐implementing hospitals). A significant decline in SIB was not seen post‐implementation (Middle TN: avg decline of 0.07% of births classified as SIB/month (*p* = 0.546); East TN: avg increase of 0.06% of births classified as SIB/month (*p* = 0.565) (Figure 2).

**Conclusion**: Implementation of IPP LARC programs may help to reduce rates of SIB. Expansion of IPP LARC programs could increase access to and uptake of IPP LARC, potentially reducing adverse neonatal and maternal outcomes associated with SIB.

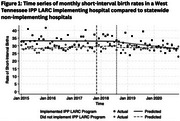

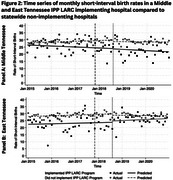

**3:30 PM – 5:00 PM**

## 545 | **Improving Human Milk Education for OB/GYN Residents: A Quality Improvement Initiative**

Kathryne Kostamo^1^; Stephanie Stokes^2^; Raeshmma Rajesh^2^; Emily Wheeler^3^; Gillian Bremer^4^; Kate Tauber^1^

^1^Albany Medical Center, Albany, NY; ^2^Albany Medical Center, Albany Medical Center, NY; ^3^Albany Medical College, Albany, NY; ^4^Albany Medical College, Albany Medical College, NY

**Objective**: Human Milk Feeding (HMF) has well‐documented benefits for infants and lactating individuals. In 2024, our institution's term nursery had a 45% exclusive HMF rate upon discharge. OB/GYN residents reported receiving limited education in providing HMF counseling to patients. In efforts to support both our residents and their patients, a Quality Improvement (QI) initiative to improve resident education on HMF was implemented.

**Study Design**: A QI initiative started with a chart review of prenatal care visits to assess baseline HMF counseling documentation among residents. Next, a structured resident educational curriculum was given over a six‐month period. Residents participated in HMF education with pre‐ and post‐education surveys for didactic knowledge, self‐efficacy, and clinical exposure. Data was analyzed using Wilcoxon signed‐rank and Mann‐Whitney U tests.

**Results**: Baseline documentation of patients who received prenatal care and delivered at our institution showed that only 51% had any documentation of a discussion of HMF during their prenatal care. Sixteen residents participated in the HMF curricula; six completed both pre‐ and post‐education surveys. Residents’ didactic knowledge scores improved, but this was not statistically significant (*p* = 0.16 paired). Residents’ self‐efficacy to adequately address patient concerns about HMF showed statistically significant improvement (*p* = 0.038 paired, *p* = 0.005 unpaired). In post‐education assessment of residents’ opinion on the influence of HMF education on their future patient care, 64% (*n* = 11) considered this to be “neutral” or “not important”.

**Conclusion**: An educational intervention improved OB/GYN resident self‐efficacy and didactic knowledge in HMF counseling. An unexpected and disheartening finding was that 64% of residents considered that HMF education would have a “neutral” or “not important” influence on their future clinical practice. Our next step is to explore residents' attitudes towards HMF to guide future educational interventions to increase resident buy‐in for this important yet undervalued care.

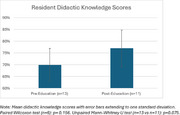

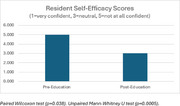

**3:30 PM – 5:00 PM**

## 546 | **Changes in Birth Prevalence of Congenital Anomalies Post‐Dobbs in States With Varying Abortion Policies**

Kavisha Khanuja^1^; Laura Uricoechea Stoker^2^; Brooke E. Ciampaglio^3^; Emily F. Rieger^4^; Kavita Vinekar^2^; Huda B. Al‐Kouatly^5^; Rodney A. McLaren, Jr^6^

^1^Sidney Kimmel Medical College Thomas Jefferson University, Philadelphia, PA; ^2^Thomas Jefferson University, Thomas Jefferson University, PA; ^3^Sidney Kimmel Medical College at Thomas Jefferson Univeristy, Philadelphia, PA; ^4^Department of Obstetrics and Gynecology, Donald and Barbara Zucker School of Medicine at Hofstra/Northwell, Manhasset, NY; ^5^Division of Maternal‐Fetal Medicine, Department of Obstetrics and Gynecology, Sidney Kimmel Medical College at Thomas Jefferson University, Philadelphia, PA, USA, Philadelphia, PA; ^6^Maimonides Medical Center, Brooklyn, NY

**Objective**: The 2022 Dobbs decision shifted reproductive rights from the federal to the state level, leading to variable abortion access, reduced resources, and potential impacts on birth outcomes. The objective of this study is to examine birth rates of neonates with congenital anomalies pre and post Dobbs by restrictive and protective abortion policies.

**Study Design**: This was a population‐based cohort study of all live, singleton births complicated by congenital anomalies using data from the U.S. Natality Vital Statistics Database from 2016–2025. Pre Dobbs included births between 2016 and 2021 and post Dobbs included births in 2023–2025. Births in 2022 (the year of the Dobbs decision) were excluded. Live births were categorized into states with the highest restriction (20 states) and states with most protection (9 states). Differences in the prevalence of live births complicated by congenital anomalies were examined between both groups using univariable analyses.

**Results**: Among congenital anomaly‐affected live births in restrictive states, 27,361 (0.303%) occurred pre‐Dobbs and 11,463 (0.310%) occurred post Dobbs (*p* = 0.067). Of the congenital anomaly‐affected live births in protected states, 17,359 (0.267%) occurred pre Dobbs and 6548 (0.266%) occurred post Dobbs (*p* = 0.707). When live births affected by congenital anomalies in individual states were categorized as restrictive, a statistically significant increase was noted post Dobbs amongst the following states: Florida (0.143% vs 0.178%, *p* = < 0.001), Louisiana (0.351% vs 0.415%, *p* = 0.001), Oklahoma (0.304% vs 0.377%, *p* = 0.001), and Utah (0.790% vs 1.113%, *p* = < 0.001).

**Conclusion**: Following the Dobbs decision, the overall prevalence of congenital anomaly‐affected live births remained stable in both restrictive and protective states. However, several individual restrictive states demonstrated significant increases. Continued surveillance is needed to monitor long‐term trends and inform health system planning in regions with limited abortion access.

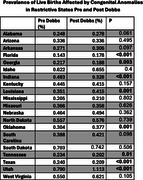

**3:30 PM – 5:00 PM**

## 548 | **Association** Between **Maternal Serum Essential Fatty Acids and Fetal Development: A Prospective Longitudinal Study**

Keisuke Akita; Satoru Ikenoue; Junko Tamai; Yasuhiko Ogata; Yushi Abe; Naotsugu Ishikawa; Yuka Fukuma; Yuya Tanaka; Toshimitsu Otani; Marie Fukutake; Yoshifumi Kasuga; Mamoru Tanaka

Department of Obstetrics and Gynecology, Keio University School of Medicine, Shinjuku‐ku, Tokyo

**Objective**: It has become clear that neonatal body fat percentage is a risk factor for childhood obesity and early onset metabolic syndrome. Fetal fractional limb volume has recently attracted attention as a new indicator of fetal growth evaluation, including fetal adiposity. Especially, fetal fractional arm volume (AVol) is more useful in assessing fetal adiposity than fetal fractional thigh volume (TVol). ω‐6 fatty acids (arachidonic acid [ARA]) and ω‐3 fatty acids (eicosatetraenoic acid [EPA], docosahexaenoic acid [DHA]) are essential fatty acids. However, their effects on fetal growth have not been elucidated. In this study, we examined the effects of ARA, EPA, and DHA in maternal serum on fetal limb volume.

**Study Design**: A prospective study was conducted in a cohort of 60 singleton pregnancies. Maternal blood tests were obtained at 10, 24, and 30 weeks. The percentage of ARA, EPA and DHA in the total fatty acids was calculated. Fetal ultrasonography was performed at 24, 30 and 36 weeks and fractional limb volume was assessed as cylindrical limb volumes based on 50% of the fetal total diaphysis length using 3D ultrasonography with as AVol and TVol depending on past reports. After performing univariate analysis, multivariate analysis was performed considering confounding factors including maternal age, parity, pre‐pregnancy body mass index, gestational weight gain, fetal sex, and gestational age at assessments, to determine the relationship between fatty acid percentage and fetal limb volume.

**Results**: Maternal serum ARA at 10, 24 and 30 weeks were significantly and negatively correlated with AVol at 36 weeks. Multivariate analysis showed a negative correlation between ARA at 10 and 30 weeks and fetal AVol at 36 weeks. No significant correlation was found between ARA and TVol. There were no significant correlations between EPA and DHA and fetal AVol and TVol.

**Conclusion**: ARA showed a significant negative correlation with fetal AVol in late pregnancy. It suggests that maternal serum ARA during pregnancy may be involved in fetal adiposity in late pregnancy.

**3:30 PM – 5:00 PM**

## 549 | **Quantifying the** Evidence **Gap: Assessing the Exclusion of Pregnant and Lactating Persons From Clinical Trials**

Kelsey C. Mumford^1^; Caleigh Propes^2^; Stephanie Morain^3^; Anne Lyerly^4^; Ruth R. Faden^3^; Jeanne Sheffield^1^

^1^Johns Hopkins Hospital, Baltimore, MD; ^2^Johns Hopkins Berman Institute of Bioethics, Baltimore, MD; ^3^Johns Hopkins University, Baltimore, MD; ^4^University of North Carolina, Chapel Hill, Chapel Hill, NC

**Objective**: In 2018, the Task Force on Research Specific to Pregnant Women and Lactating Women (PRGLAC) reported their recommendations to address the gaps in research on safe and effective therapies for pregnant and lactating women. Despite these recommendations, we hypothesized that these populations continue to be unjustifiably excluded from most clinical trials in the United States.

**Study Design**: A search of phase IV clinical trials and observational studies from 2020–2024 related to four common conditions affecting pregnant and lactating persons (hypertension, diabetes mellitus, anxiety/depression, and HIV/AIDS) was performed using ClinicalTrials.gov. Each study that excluded pregnant or lactating persons was independently evaluated by two reviewers and assigned a risk category of minimal risk, substantial risk, or acceptable to include pregnant or lactating persons on a case‐by‐case basis. Statistical analysis included use of Pearson's chi‐square with statistical significance set at *p* < 0.05.

**Results**: 206 phase IV clinical trials and 550 observational studies met inclusion criteria and were reviewed. Pregnant and lactating persons were explicitly excluded from 138 (67%) of phase IV and 256 (47%) of observational studies. Of the exclusionary phase IV studies, 65 (47%) were classified as posing substantial risk to pregnant persons and 26 (27%) to lactating persons. Of the exclusionary observational studies, 28 (12%) posed substantial risk to pregnant persons and 7 (6%) to lactating persons. Risk classifications were found to significantly differ according to chronic disease category (Table 1).

**Conclusion**: Even when looking at phase IV and observational studies for established medications concerning the most common chronic conditions experienced by pregnant and lactating persons, they are still being unnecessarily systematically excluded from clinical trials. These data emphasize that despite the PRGLAC task force's warning about the detrimental effects this has for both these populations and their fetuses/newborns, the dearth of data on medications and therapies is not set to be rectified anytime soon.

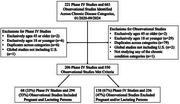

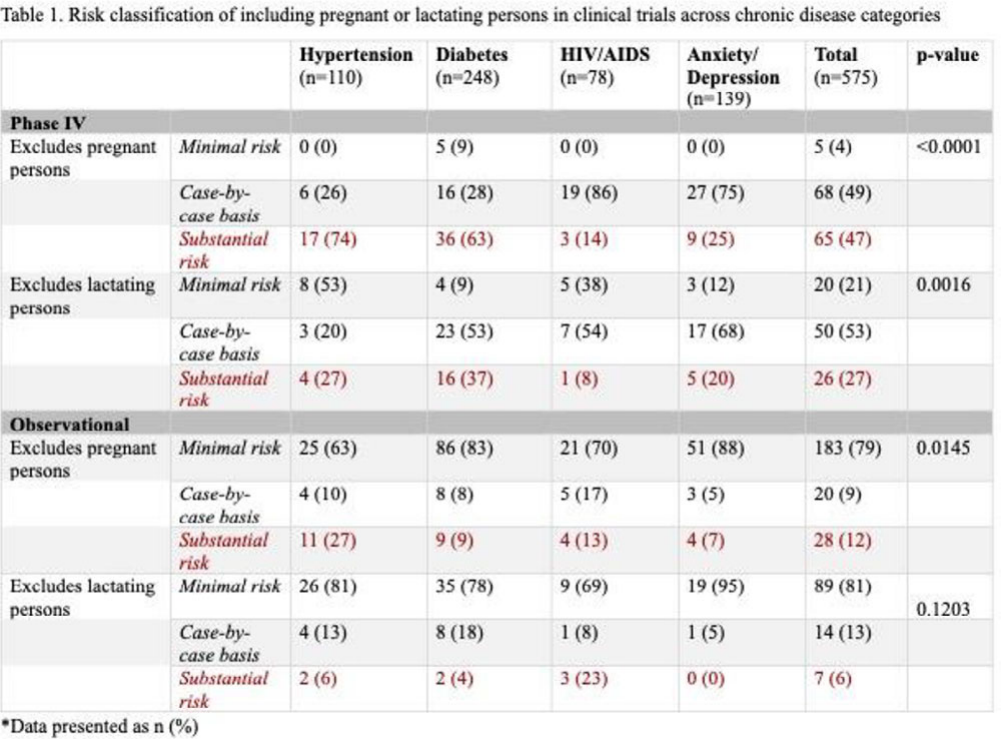

**3:30 PM – 5:00 PM**

## 550 | **Incidence of Fetal Growth and Fluid Abnormalities Diagnosed by Serial Ultrasounds in Patients With Obesity**

Kendall M. Cunningham^1^; Emily A. O'Brien^1^; Kristi Haedrich^1^; Kathryn McMullen^1^; Samantha Allen^1^; Serdar Ural^2^; Jaimey M. Pauli^1^

^1^Penn State Health Milton S. Hershey Medical Center, Hershey, PA; ^2^The Pennsylvania State University College of Medicine, The Pennsylvania State University College of Medicine, PA

**Objective**: Obesity affects nearly 40% of pregnant patients. ACOG recommends periodic growth and fluid evaluation for patients with certain comorbidities. It is unclear whether obesity, in the absence of other comorbidities, is an indication for serial ultrasound assessment. Despite this, many institutions routinely recommend serial ultrasounds for patients with obesity. The primary objective of this study was to determine the incidence of fetal growth and fluid abnormalities in patients who underwent ultrasound assessment for the indication of maternal obesity.

**Study Design**: This is a retrospective cohort study including subjects who delivered between 2019–2023 in an academic medical center where patients with obesity routinely undergo serial ultrasounds. Subjects with a singleton gestation and a pregravid (PG) body mass index (BMI) >30.0 kg/m^2^ were included. Exclusion criteria included coexisting maternal, placental/umbilical cord, and fetal conditions. The results were compared by obesity class as defined by the subject's PG BMI.

**Results**: 4279 subjects with obesity were screened and 1185 were included. There were 566 subjects with Class I, 351 with Class II and 268 with Class III Obesity. Demographic characteristics were similar amongst the three groups. Subjects with Class III Obesity were more likely to have an amniotic fluid abnormality (20.2%) when compared to subjects with Class I (12.3%, OR 1.82, *p* = 0.003) and Class II Obesity (12.3% OR 1.81 *p* = 0.008). Mild polyhydramnios was the most common fluid abnormality amongst all groups. A growth abnormality was diagnosed in 21.9%, 27.9% and 26.9% of subjects with Class I, II and III Obesity, respectively. Suspected large for gestational age fetal weight was more common than fetal growth restriction in all groups.

**Conclusion**: Among patients with uncomplicated obesity, suspected fetal growth and amniotic fluid abnormalities are common. However, when noted, these findings are often transient and resolve prior to delivery. Additional research is needed to determine the optimal frequency and timing of ultrasound assessment for patients with obesity.

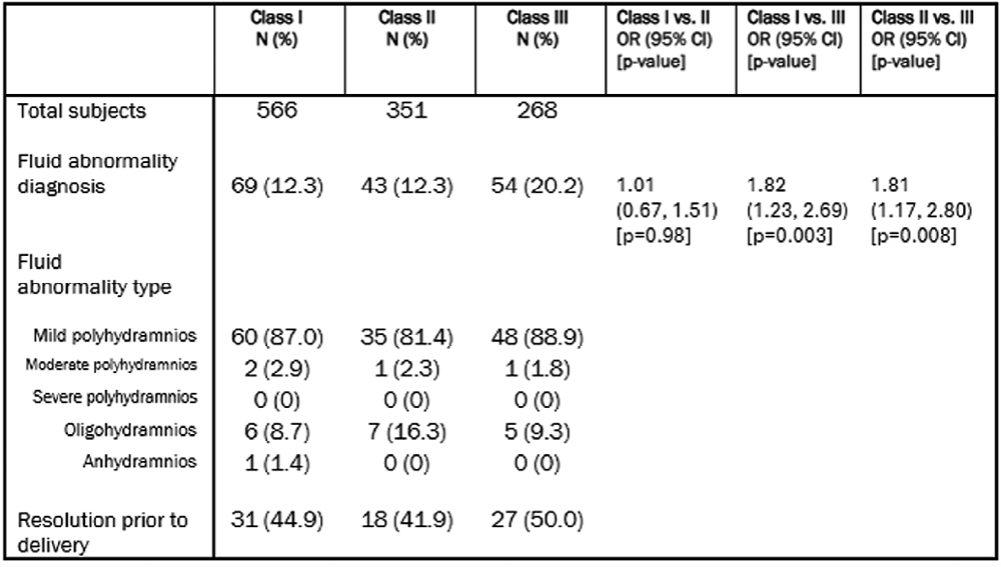

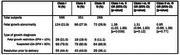

**3:30 PM – 5:00 PM**

## 551 | **Economic** Implications **of Stillbirth to Families and the Health Care System**

Warittakorn Kategeaw^1^; Kennedy Yost^2^; Tsegaselassie Workalemahu^3^; Nathorn Chaiyakunapruk^1^; Robert M. Silver^4^

^1^Department of Pharmacotherapy, University of Utah College of Pharmacy, Department of Pharmacotherapy, University of Utah College of Pharmacy, Salt Lake City,, UT; ^2^University of Utah School of Medicine, University of Utah School of Medicine, UT; ^3^University of Utab, University of Utah, UT; ^4^Department of Obstetrics and Gynecology, University of Utah Health, Salt Lake City, UT

**Objective**: To evaluate the medical health care expenditure and healthcare utilization in pregnancies affected by stillbirth.

**Study Design**: We conducted a population‐based case‐control study using the 1978 to 2021 Utah state fetal death and live birth records linked to the state's All‐Payers Claims Database. Cases were stillbirths (≥20 weeks’ gestation, Apgar 0/0), and controls were live births without prior pregnancy loss. Controls were 2:1 matched to cases by birth/death month and year. Maternal health care expenditure was assessed across antenatal, delivery, and postpartum periods, and stratified by insurance payer vs. out‐of‐pocket (deductible, coinsurance, and copay). Generalized linear models were used to estimate mean differences in per‐person costs adjusted for inflation to reflect 2025 U.S. dollars.

**Results**: Among 1948 stillbirths and 4136 controls with medical claims, the mean total direct medical expenditure reimbursed by payers was $121,359 in stillbirths cases and $83,370 in controls, representing a 45.5% (*p*‐value < 0.001) higher expenditure for cases than controls. In the post‐partum period, the mean expenditure reimbursed by payers was 2.7 times higher in stillbirths group than controls (*p*‐value < 0.001). The mean total out‐of‐pocket expenditure was $18,938 in stillbirths group and $17,429 in controls, representing an 8.7% (*p*‐value = 0.2) higher out‐of‐pocket expenditure in cases than controls. Higher direct medical expenditures associated with stillbirth post‐partum may reflect the complex medical and psychosocial needs for affected families.

**Conclusion**: Stillbirth imposes significant economic implications on both families and the health care system. The observed increased health care expenditures for stillbirth‐affected pregnancies in our data represent the health system's response to higher clinical acuity and care needs during and after a stillbirth. Further investigations are warranted to determine areas where health systems can better support affected individuals through tailored care delivery.

**3:30 PM – 5:00 PM**

## 552 | **More** Complicated **Perioperative Course of Cesarean Section in Patients With Endometriosis**

Keren Zloto^1^; Elad Berkowitz^1^; Yael Lavi^1^; Stav Bar Peled^1^; Miri Shemesh^1^; Chen Berkovitz^2^; Shlomi Toussia‐Cohen^3^; Keren Ofir^4^; Shai Elizur^1^

^1^Sheba Medical Center, Tel Hashomer, Tel Aviv; ^2^Sheba Medical Center, Ramat Gan, HaMerkaz; ^3^The Sheba Medical Center, The Sheba Medical Center, HaMerkaz; ^4^Sheba Medical Center, Sheba Medical Center, HaMerkaz

**Objective**: Recent studies suggest an increased risk of pregnancy complications and a higher incidence of cesarean sections (CS) in individuals with endometriosis. However, information on CS‐specific outcomes in this population remains scarce. This study aimed to assess CS outcomes in patients with endometriosis.

**Study Design**: A case‐control study was conducted at a tertiary care center between 2011 and 2023. The study group included all women with endometriosis who underwent a CS that were matched at a 1:2 ratio based on women's age, date of CS, type of CS (emergency or elective), previous CS and gestational age to control group comprised of those without endometriosis. Individuals with multiple gestations were excluded from the study. Pregnancy, delivery, and perinatal outcomes were evaluated.

**Results**: We compared 108 CS deliveries in the study group to 216 in the control group. Patients with endometriosis were more likely to be nulliparous (65.7% vs. 47.2%, *p* < 0.01), conceived via IVF (53.7% vs. 17.1%, *p* < 0.01), and had more placenta previa (7.4% vs. 2.3%, *p* = 0.03). They also had more surgical adhesions (40.7% vs. 6.9%, *p* < 0.01), greater blood loss (600 mL vs. 500 mL, *p* < 0.01), and a larger drop in hemoglobin (1.36 vs. 1.1 g/dL, *p* = 0.05). The time to fetal extraction was nearly longer (7 vs. 5 minutes, *p* = 0.06). Among the endometriosis group, 63.9% had a history of surgery.

**Conclusion**: Patients with endometriosis are at higher risk of a complicated CS, with increased blood loss and longer extraction times, possibly due to adhesions and placenta previa.

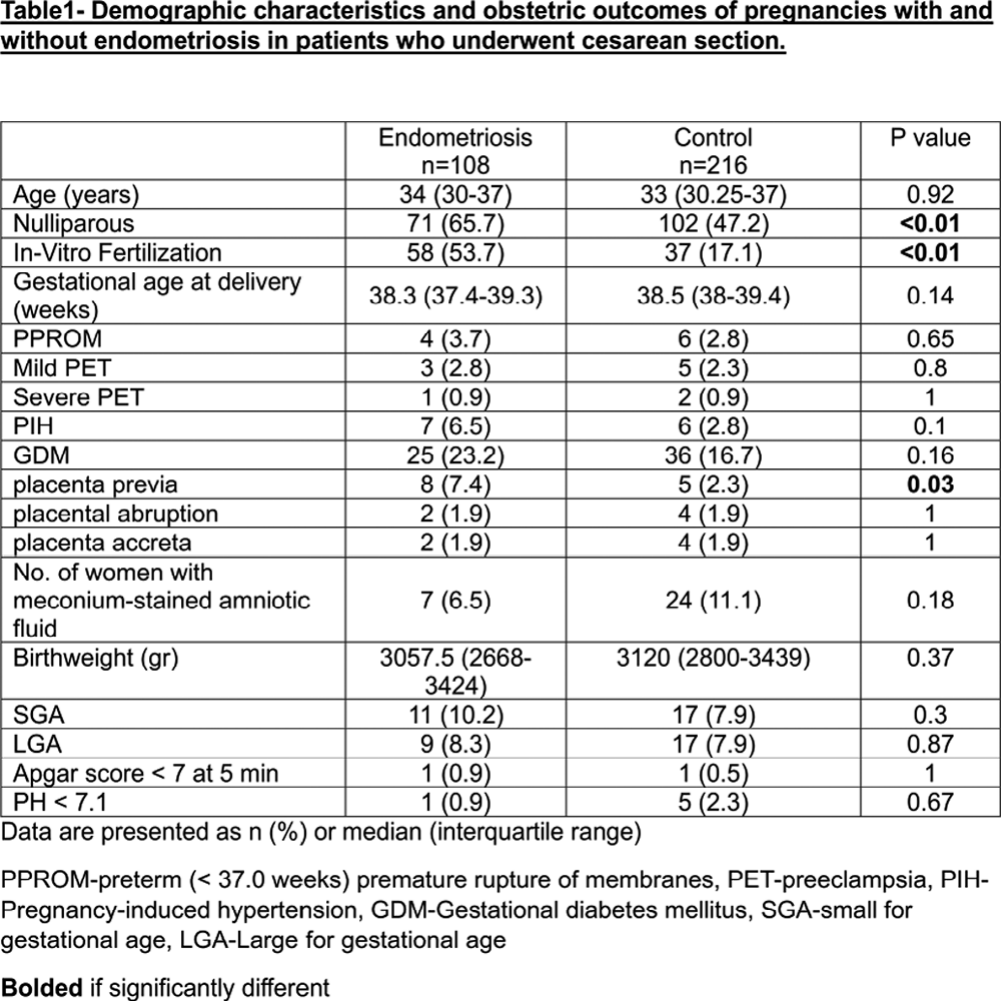

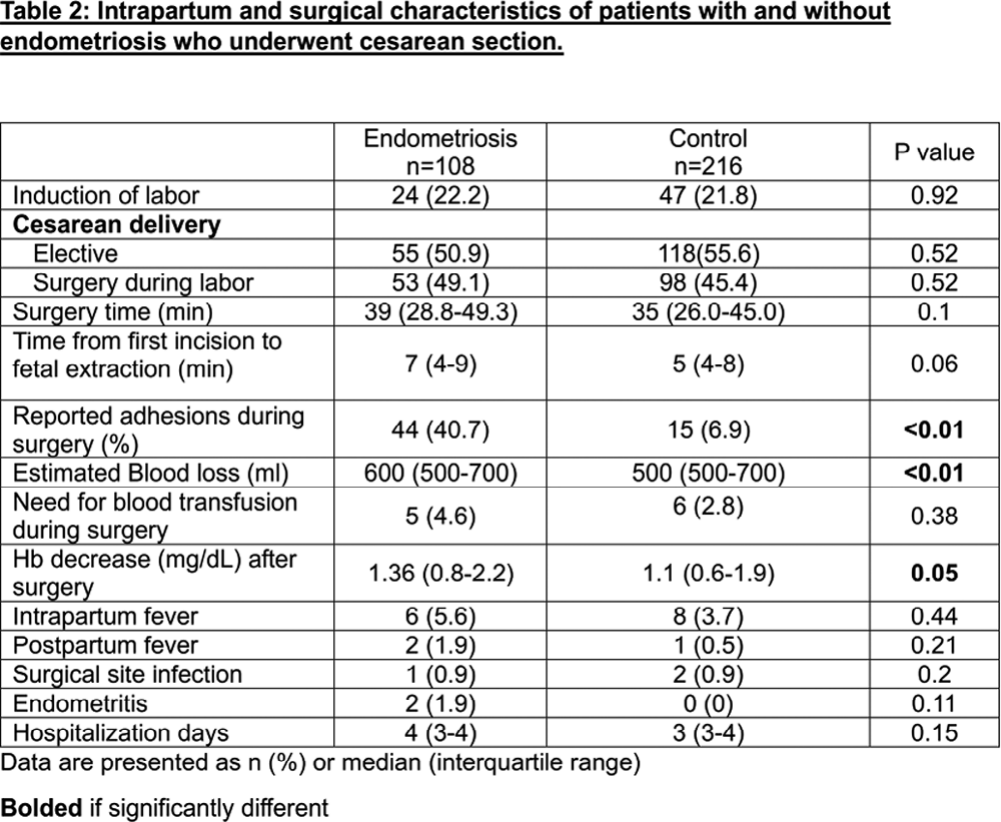

**3:30 PM – 5:00 PM**

## 553 | **Impact of** Induction **Versus Expectant Management on Cesarean Rates in Nulliparous Women With Closed Cervix**

Khalil M. Chahine; Baha M. Sibai

Division of Maternal‐Fetal Medicine, Department of Obstetrics, Gynecology and Reproductive Sciences, McGovern Medical School at UTHealth Houston, Houston, TX

**Objective**: To evaluate whether induction of labor at 39 weeks versus expectant management in low‐risk nulliparous women with a closed cervix on exam at 37–38 weeks is associated with an increased risk of cesarean delivery (CD).

**Study Design**: This is a secondary analysis of a multicenter randomized trial of induction of labor at 39 weeks compared with expectant management in low‐risk nulliparous patients (ARRIVE). Only participants with a cervical dilation ≤0.5 cm within 72 hours before or 24 hours after randomization (37w4d‐38w6d) were included. Primary outcome was rate of CD. Secondary outcomes were maternal (modified Bishop score on L&D, duration of labor, rate of operative vaginal delivery, chorioamnionitis, postpartum infection, postpartum hemorrhage, prolonged postpartum hospitalization) and neonatal (composite of adverse outcomes). Modified Poisson and log‐linear regressions were performed to estimate adjusted relative risks; penalized methods addressed rare events, and Chi‐square and Mann‐Whitney U tests were used for group comparisons.

**Results**: 2399 patients were included: 1205 in induction (IOL) and 1194 in expectant management (EM). Baseline demographics were similar (IOL v EM), except for Hispanics (35.9% v 30.7%, *p* < 0.01), BMI ≥30 (23.3% v 27.6%, *p* < 0.05), and history of loss (21.5% v 26.9%, *p* < 0.01). After adjustment for significant variables, **rate of CD in IOL was significantly lower than in EM (24.9% v 30.0%, aRR 0.85 [95% CI: 0.75–0.97])**. The primary indication for CD were similar (IOL v EM): non‐reassuring fetal status (52.0% v 47.3%, *p* 0.31) and dystocia/maternal exhaustion (39.4% v 46.0%, *p* 0.10). As for secondary outcomes, EM had higher Bishop score on admission, higher rates of SROM, and shorter duration between admission and delivery, without increased risk of adverse maternal or neonatal outcomes (Table).

**Conclusion**: Women with a closed cervix in the early term period have a lower CD rate when induced at 39 weeks. This finding can help counsel patients with a closed cervix in clinic that induction of labor at 39 weeks does not increase CD risk.

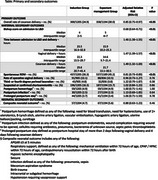

**3:30 PM – 5:00 PM**

## 554 | **Cervical Mucin** Expression **Is Associated With Preterm Birth: Molecular Determinants of Mucus Quality in Pregnancy**

Kristin M. Klohonatz^1^; Zoe Graskin^1^; Rita Leite^1^; Lauren Anton^1^; Rachel F. Ledyard^2^; Michele R. Hacker^3^; Heather H. Burris^2^; Kristin D. Gerson^4^

^1^University of Pennsylvania Perelman School of Medicine, Philadelphia, PA; ^2^Children's Hospital of Philadelphia, Philadelphia, PA; ^3^Beth Israel Deaconess Medical Center, Boston, MA; ^4^University of Pennsylvania, Hospital of the university of Pennsylvania, PA

**Objective**: Cervical mucus serves as a key immune and structural barrier to prevent microbial ascension from the lower genital tract. Mucins are glycoproteins that contribute to the gel‐like consistency of mucus and provide structural scaffolding. We previously showed that *G. vaginalis*, a vaginal anaerobe implicated in the pathophysiology of spontaneous preterm birth (sPTB), induces expression of select mucins and genes regulating mucus hydration in cervical epithelial cells *in vitro*. The impact of mucin profiles on pregnancy outcomes remains under investigated. We examined associations between cervical mucin expression and sPTB in human pregnancy.

**Study Design**: This was a prospective, nested, case‐control study of 23 sPTB cases and 23 term controls from the Boston‐based *Spontaneous Prematurity and Epigenetics of the Cervix (SPEC)* study. Cases were matched on race, parity, and age. Gel‐forming mucins (MUC2, MUC5AC, MUC5B, MUC6) and membrane‐spanning mucins (MUC1, MUC16) were measured by ELISA from second trimester cervical swabs and normalized to total protein. T‐tests were performed to compare groups.

**Results**: Participant characteristics are presented in Table 1. Expression of MUC2 (2.94‐fold), MUC5B (2.44‐fold), MUC6 (1.85‐fold), and MUC16 (3.52‐fold) was higher in sPTB vs term controls (all *p* < 0.05, Figure 1). Similar nonsignificant trends were seen for MUC5AC and MUC1. Total mucin content was 1.56‐fold higher (*p* = 0.006) and total membrane‐spanning mucin content was 1.83‐fold higher (*p* = 0.004) in sPTB vs term. We did not detect a difference in total gel‐forming mucin content across groups (*p* = 0.24).

**Conclusion**: Higher concentrations of gel‐forming and membrane‐spanning cervical mucins are associated with sPTB. The hydrophilic properties of cervical mucins result in increased mucus water content, likely rendering mucus less viscous and more permeable to microorganisms. Modification of mucus properties in pregnancy may be targeted as a future therapeutic strategy to strengthen this biophysical barrier and mitigate microbiota‐associated sPTB risk. Charles H. Hood Foundation (HHB). PennCHOP Microbiome Program (KDG).

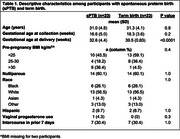

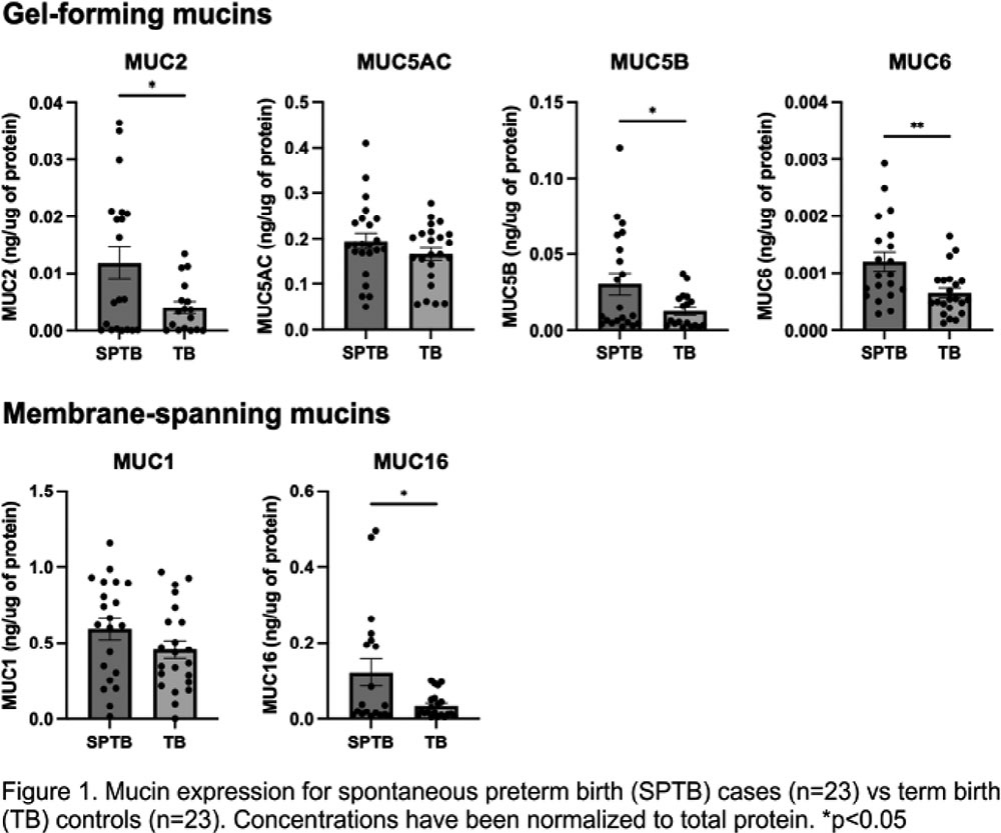

**3:30 PM – 5:00 PM**

## 555 | **Geographic** Differences **In Co‐Occurring Perinatal Mood and Anxiety Disorders and Substance use During Pregnancy**

Kristin C. Prewitt; Erin Nacev; Ann Martinez Acevedo; Megan Fuerst; Maria I. Rodriguez

Oregon Health & Science University, Portland, OR

**Objective**: Substance use disorders (SUD) and perinatal mood and anxiety disorders (PMAD) are common and independently associated with adverse pregnancy outcomes, yet their co‐occurrence is not well understood. This study assesses differences by rural and urban residence in rates of co‐occurring SUD and PMAD during pregnancy.

**Study Design**: We conducted a cross‐sectional study using linked Medicaid claims and birth certificate records from Oregon and South Carolina from 2016 to 2020. We identified SUD and PMAD diagnoses during pregnancy and within 60 days postpartum using International Classification of Disease‐10 codes. We compared those with SUD by diagnosis of any PMAD using chi‐square tests. We used Rural‐Urban Community Area codes to identify rural versus urban residence.

**Results**: Out of 185,809 births, 18,670 (10.0%) were identified as having SUD and 43,759 with PMAD (23.6%). Substance use disorders (SUDs) were significantly more common among individuals with PMAD compared to those without, across both rural and urban settings (*p* < 0.001 for all). Tobacco use was most prevalent, affecting 41.2% of rural and 38.9% of urban individuals with PMAD. Rates of cannabis, opioid, alcohol, sedative, and stimulant use were 2–6 times higher among those with PMAD. Among individuals with PMAD, substance use was generally higher in rural compared to urban settings, with rural cases showing up to 22% higher stimulant use and 14% higher alcohol use.

**Conclusion**: PMADs are associated with SUD during pregnancy, especially in rural areas. Targeted screening and intervention are needed to address this dual burden and improve maternal and neonatal morbidity and mortality.

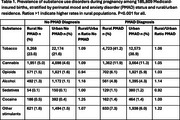

**3:30 PM – 5:00 PM**

## 556 | **A Nationwide** Analysis **of Adverse Delivery Outcomes Among Adolescents Pre‐ and Post‐Dobbs Decision**

LaMani Adkins^1^; Katie Freedy^1^; Shakthi Uunithan^1^; Jonas J. Swartz^1^; Sarah K. Dotters‐Katz^2^; Sarahn M. Wheeler^2^; Rachel L. Wood^3^

^1^Duke University, Duke/Durham, NC; ^2^Division of Maternal Fetal Medicine, Department of Obstetrics and Gynecology, Duke University, Duke University/Durham, NC; ^3^Division of Maternal Fetal Medicine, Department of Obstetrics and Gynecology, Duke University, Durham, NC

**Objective**: Adolescents account for 4% of all pregnancies in the United States and approximately 10% of all elective terminations. In June 2022, the Dobbs decision eliminated federal abortion protections and abortion access is adjudicated by state. We evaluated the impact of the Dobbs decision on delivery outcomes in adolescent births based on state level abortion access.

**Study Design**: We conducted an IRB approved interrupted time series analysis (ITS) of U.S. adolescent (age <20 years) births using birth certificate data in two groups of states: those with abortion protection(SAP)‐ CA, CO, CT, DE, IL, MA, MD, ME, NJ, NV, NY, OR, and VT versus those with trigger bans(TB) restricting abortion access‐ AR, ID, KY, LA, MS, MO, ND, OK, SD, TN, and UT. Primary outcomes were preterm birth(PTB) (<37 weeks) and low birthweight(LBW) (<2500g) across two time periods: pre‐Dobbs (January 1, 2021–May 31, 2022) and post‐Dobbs (December 1, 2022–December 31, 2023). Secondary outcomes included mode of delivery and maternal morbidity.

**Results**: 142,859 births were included: 78,682 in SAP states and 64,177 in TB states. Overall, average maternal age was 18.1+1.2 years, with most births occurring in white individuals with no meaningful differences in BMI, insurance status, or prenatal care between groups. In both the pre‐ and post‐Dobbs periods, rates of PTB (Figure 1), LBW (Figure 2), cesarean section were significantly higher in TB states; however, there were no changes in the rates within or between groups in the pre‐ or post‐Dobbs period. Maternal morbidity significantly increased above expected in TB states in the post‐Dobbs period (RR 1.55, 95% CI 1.11–2.15), there was no significant increase in maternal morbidity in SAP states (RR 1.12, 95% CI 0.85–1.47).

**Conclusion**: The rates of preterm birth, low birthweight, and c‐section were higher in adolescents in states with abortion trigger bans, though there was no change in these trends following the Dobbs decision. Maternal morbidity for adolescents increased post‐Dobbs decision in states with abortion restrictions, aligning with previous findings.

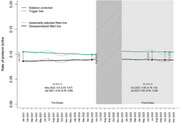

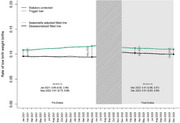

**3:30 PM – 5:00 PM**

## 557 | **Keys to A** Successful **Induction in Patients With BMI >40**

Lauren T. Gilgannon^1^; Enkhbileg Dendev^2^; Allison Gonzalez^3^; Madison Coleman^3^; Shivani Arza^3^; Christopher S. Ennen^3^

^1^Univeristy of Virginia School of Medicine, Charlottesville, VA; ^2^Unvieristy of Virginia School of Medicine, Charlottesville, VA; ^3^University of Virginia School of Medicine, Charlottesville, VA

**Objective**: The objective of this study was to determine factors associated with a successful induction of labor in patients with body mass index (BMI) >40 kg/m^2^.

**Study Design**: This was a retrospective cohort study of 374 patients with BMI >40 kg/m^2^ who underwent induction of labor at a single academic institution from 2011–2021.

**Results**: Induction of labor resulted in a vaginal delivery for 60% (255/374) of the patients. Those having a vaginal delivery had lower BMI at both their initial visit and at delivery, higher parity, rupture of membranes at an advanced cervical dilation, and a shorter length of induction. In contrast, women who had cesarean deliveries were more likely to have preeclampsia severe features, develop chorioamnionitis, use more doses of misoprostol, and reach higher doses of oxytocin. When comparing obesity classes [defined as Class III (BMI 40.0–49.9), Class IV (BMI 50–59.9) and Class V (BMI >60)], those with a lower BMI class had higher parity and shorter length of induction. In contrast, those with a higher BMI class were more likely to have a diagnosis of chronic hypertension and require higher doses of oxytocin.

**Conclusion**: Patients with a lower BMI were more likely to deliver vaginally after an induction of labor. Specifically, higher parity, rupture of membranes at an advanced cervical dilation, and a shorter length of induction were associated with the success of a vaginal delivery in these patients. Comorbidities such as hypertensive disorders of pregnancy may affect the success of an induction in those with higher BMIs.

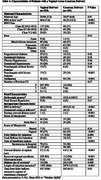

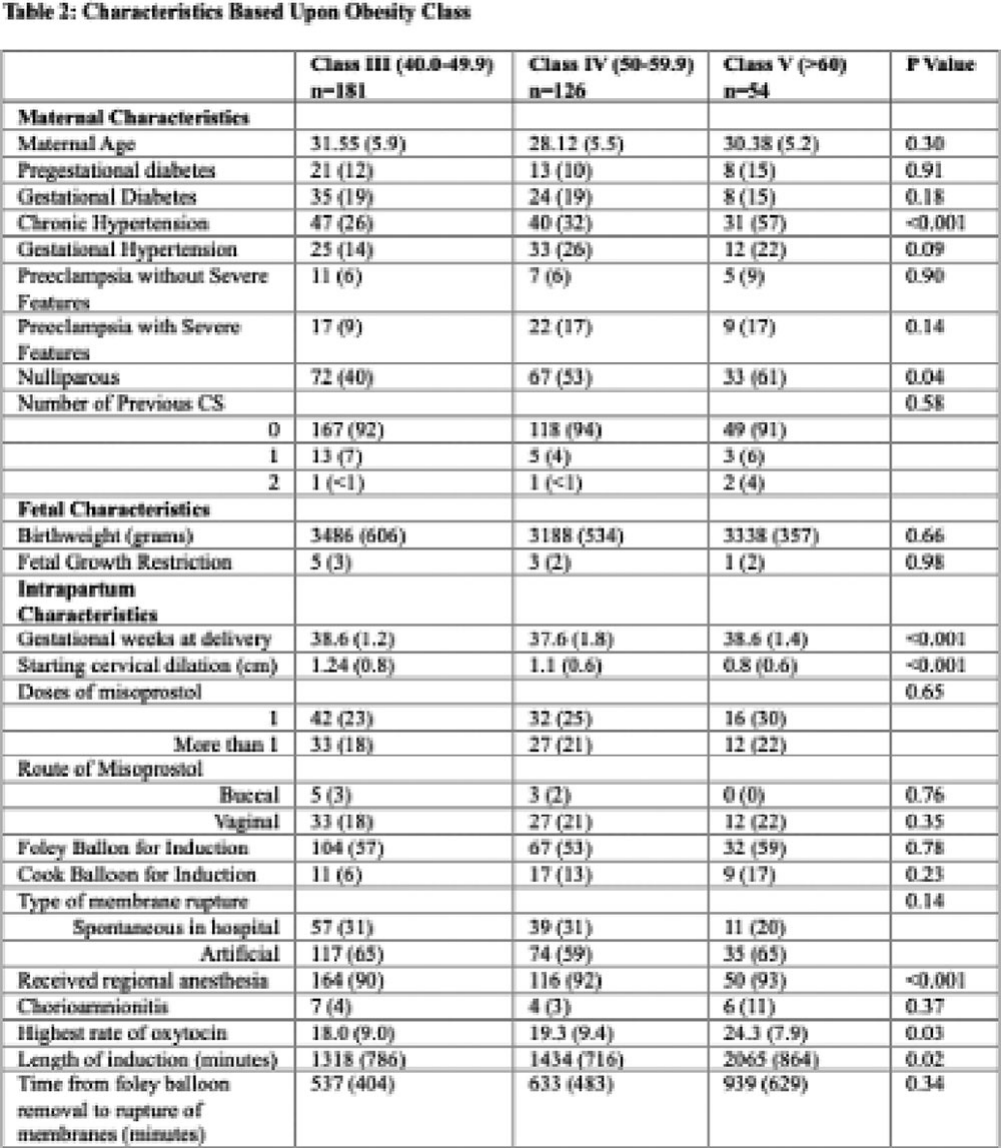

**3:30 PM – 5:00 PM**

## 558 | **Psychosocial** Stressors **and Preterm Birth Among Individuals at Risk for Adverse Pregnancy Outcomes**

Lauren Keenan‐Devlin^1^; Chuhan Wu^1^; Alexa A. Freedman^2^; Linda M. Ernst^1^; Gregory E. Miller^3^; Ann E Borders^4^

^1^Endeavor Health, Evanston, IL; ^2^Northwestern University, Chicago, IL; ^3^Northwestern University, Evanston, IL; ^4^Endeavor Health, Evanston Hospital, Evanston, IL

**Objective**: Psychosocial stress has been linked with elevated risk for adverse pregnancy outcomes (APOs), but whether these stressors compound risk for pregnancies with other APO risk factors (high APO‐risk) is unclear. We investigated whether mid‐pregnancy psychosocial stressors independently increased risk of preterm birth (PTB) for individuals with and without other APO risk factors.

**Study Design**: The prospective Stress, Pregnancy and Health cohort collected self‐reported stress measures in 2^nd^ and 3^rd^ trimesters and clinical outcomes from the medical record for a cohort of 605 singleton pregnancies. High APO‐risk was defined by history of IUGR, antenatal bleeding, prior PTB, PPROM, or IUFD in a prior pregnancy, or past or current hypertensive disorders of pregnancy. Psychosocial factors included loneliness, depressive symptoms, financial hardship and food insecurity and were z‐scored for comparability. PTB was defined as delivery < 37 gestational weeks. Logistic regressions were conducted within each APO‐risk stratum, adjusting for maternal age, race, and ethnicity.

**Results**: The 138 individuals with high APO‐risk were more likely to identify as Black or Hispanic and have lower socioeconomic status. As expected, PTB occurred more frequently in the high APO‐risk group versus the low‐risk group (22.5% versus 6.0%). Among high‐risk pregnancies, each 1‐SD increase in loneliness (OR1.48, 95% CI 1.03–2.14), financial hardship (OR1.53, 1.06–2.21) and food insecurity (OR1.71, 1.20–2.43) was significantly associated with higher odds of PTB, while depressive symptoms were not. These associations were absent in low‐risk pregnancies.

**Conclusion**: Elevated loneliness, financial strain, and food insecurity were independently associated with PTB in pregnancies with high APO‐risk, compounding risk for adverse outcomes among those already more likely to experience them. Our results suggest that psychosocial stress screening and intervention in prenatal care, particularly for those with high APO‐risk, may be a strategy to reduce risk for PTB.

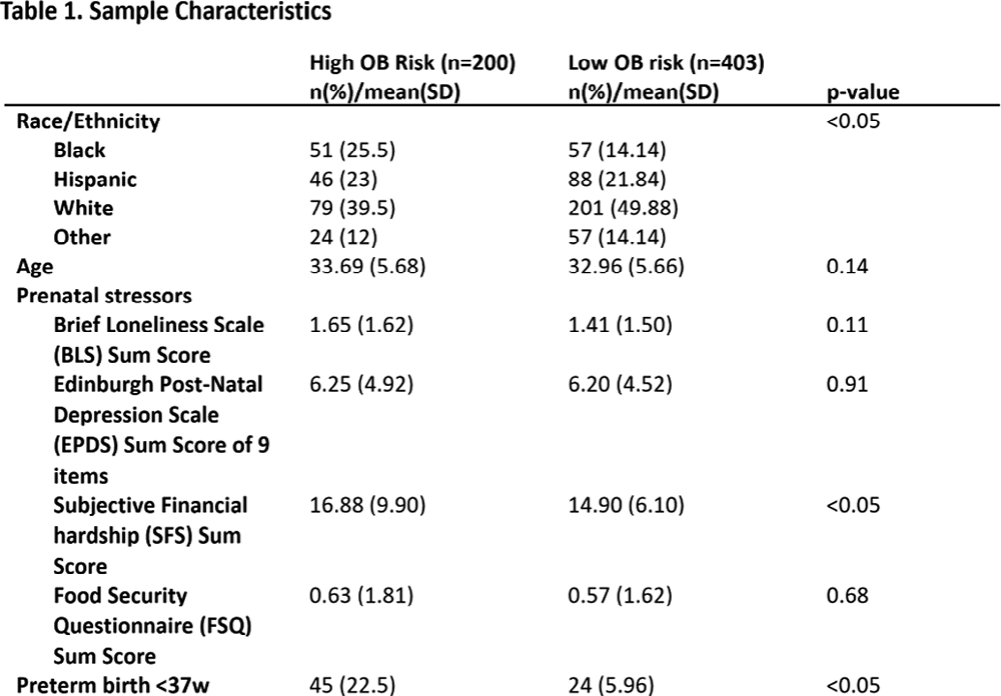

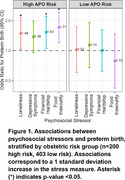

**3:30 PM – 5:00 PM**

## 559 | **Resolved and** Persistent **Fetal Growth Restriction: Associations With Small‐for‐Gestational‐Age Neonates**

Leshae A. Cenac^1^; Mona M. Makhamreh^2^; Lucy M. Vaughn^3^; Emily F. Rieger^4^; Madeline V. Smith^5^; Sam Mauser^6^; Leanna M. Reichert^5^; Huda B. Al‐Kouatly^7^

^1^Division of Maternal‐Fetal Medicine, Department of Obstetrics and Gynecology, Sidney Kimmel Medical College at Thomas Jefferson University, Philadelphia, PA, USA, Philadephia, PA; ^2^Baylor College of Medicine, Houston, TX; ^3^Sidney Kimmel Medical College at Thomas Jefferson Univeristy, Philadelphia, PA; ^4^Department of Obstetrics and Gynecology, Donald and Barbara Zucker School of Medicine at Hofstra/Northwell, Manhasset, NY; ^5^Sidney Kimmel Medical College at Thomas Jefferson University, Philadelphia, PA; ^6^Westchester Medical Center, Philadelphia, PA; ^7^Division of Maternal‐Fetal Medicine, Department of Obstetrics and Gynecology, Sidney Kimmel Medical College at Thomas Jefferson University, Philadelphia, PA, USA, Philadelphia, PA

**Objective**: To compare the odds of delivering a small for gestational age (SGA) neonate in pregnancies complicated by resolved versus persistent fetal growth restriction (FGR), relative to pregnancies with appropriate for gestational age (AGA) fetuses.

**Study Design**: This was a retrospective cohort study of singleton, non‐anomalous pregnancies diagnosed with FGR between 2017 and 2023. FGR was defined as estimated fetal weight (EFW) and/or abdominal circumference (AC) <10^th^ percentile for gestational age. Resolved FGR was defined as a prior diagnosis of FGR with a subsequent EFW and AC ≥ 10^th^ percentile before delivery. Persistent FGR was defined as continued EFW and/or AC <10^th^ percentile through delivery. SGA was defined as birthweight <10^th^ percentile for gestational age at birth. Multivariable logistic regression was used to estimate adjusted odds ratios (aOR) and 95% confidence intervals (CI). Analyses were performed using SPSS v30.

**Results**: A total of 9923 singleton pregnancies were reviewed and stratified into three groups: AGA (*N* = 8858), resolved FGR (*N* = 341), and persistent FGR (*N* = 724). Resolved FGR was associated with over a threefold increased odds of SGA (aOR 3.44; 95% CI: 2.59–4.56) relative to pregnancies without FGR. Persistent FGR conferred the highest risk (aOR 14.83; 95% CI: 12.51–17.58). Maternal factors independently associated with SGA included substance use (aOR 1.52; 95% CI: 1.16–2.00) and a history of FGR in a prior pregnancy (aOR 2.80; 95% CI: 1.48–5.29).

**Conclusion**: In our ultrasound cohort, both resolved and persistent FGR were independently associated with significantly increased odds of delivering a SGA infant, with persistent FGR conferring the highest risk. These findings suggest that FGR, even when resolved, may carry clinical significance and warrant attention due to potential perinatal implications. Further research is needed to better understand the clinical relevance and long‐term outcomes of resolved FGR.

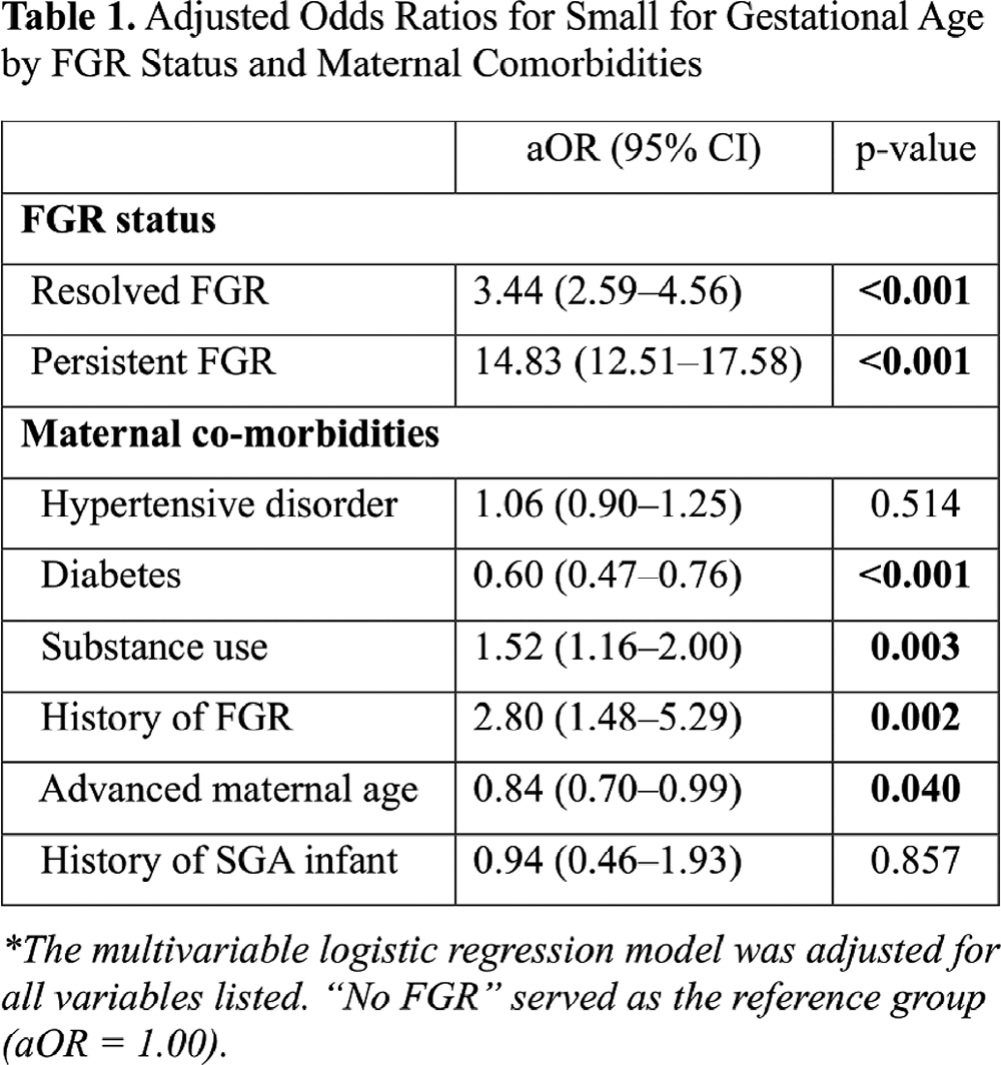

**3:30 PM – 5:00 PM**

## 560 | **Vaginal Birth** After **Cesarean (VBAC): Achieving the Healthy People Nulliparous Term Singleton Vertex (NTSV) Goal**

Leslie C. Omeire^1^; John J. Byrne^2^; Patrick S. Ramsey^3^

^1^University of Texas San Antonio, Long School of Medicine, San Antonio, TX; ^2^University of Texas Health Science Center in San Antonio, San Antonio, TX; ^3^Department of Obstetrics and Gynecology University of Texas Health Science Center in San Antonio, San Antonio, TX

**Objective**: Substantial variation in cesarean births among low‐risk, first‐time parturients emphasizes the need for quality improvement efforts to target safe reduction in NTSV cesarean births. We sought to evaluate whether hospital achievement of the Healthy People 2030 NTSV goal (≤23.6%) was associated with a hospital having a VBAC policy in the United States. **Study Design**: We conducted a cross sectional cohort study analysis using 2024 Leapfrog hospital survey data on NTSV cesarean rates at 1655 participating hospitals. Data was extracted from survey responses using filters for maternity care “services” and achievement of the Healthy People 2030 “standard” to tabulate reported information for further analysis. Each hospital was categorized as “VBAC supportive” or “VBAC restrictive”, and as “meeting the standard” or “not meeting the standard” for the Healthy People 2030 NTSV goal. Achievement of the Healthy People 2030 NTSV goal was compared between the groups. Statistical analysis was performed using Chi‐square test as appropriate. **Results**: Mean NTSV cesarean delivery rate was 25.3% across all reporting maternal care facilities (*n* = 1655), with 84.1% having in place a VBAC supportive policy. 41.1% of all reporting hospitals met the Healthy People NTSV goal. When evaluating progress towards meeting the NTSV goal, categorized by “VBAC supportive” or “VBAC restrictive”, 39.3% of “VBAC supportive” hospitals (*n* = 1392) and 49.1% of “VBAC restrictive” hospitals (*n* = 263) meet the Healthy People 2030 NTSV goal of <23.6% (*p* < .01). **Conclusion**: Hospitals with VBAC‐supportive policies were less likely to meet the target NTSV cesarean rate of ≤23.6%, compared to VBAC restrictive hospitals. These findings suggest that other factors contributing to the practice environment may have a more considerable impact on NTSV outcomes than VBAC policy alone.

**3:30 PM – 5:00 PM**

## 561 | **Severe Maternal Morbidity in Second‐Stage Cesarean Sections**

Leslie Tseng^1^; Olga Grechukhina^2^; Katherine H. Campbell^2^; Lisbet S. Lundsberg^1^; Jennifer F. Culhane^1^; Sara Giuliano^1^; Caitlin Partridge^1^; Colleen Sinnott^2^

^1^Department of Obstetrics, Gynecology, and Reproductive Sciences, Yale School of Medicine, New Haven, CT; ^2^Division of Maternal Fetal Medicine, Yale School of Medicine, New Haven, CT

**Objective**: Second‐stage Cesarean sections (CS), performed after full cervical dilation, are associated with higher maternal and neonatal morbidity compared to CS for other indications. Among patients undergoing 2^nd^‐stage CS, we evaluated risk factors associated with severe maternal morbidity (SMM).

**Study Design**: All nulliparous, term, singleton, vertex gestations delivered via CS in one health system between 2013–2024 were identified in electronic medical records (EMR). Second‐stage CS was defined as cases with “full dilation” recorded. Duration of the 2^nd^ stage was calculated by subtracting the time of full dilation from the time of delivery; all outliers were reviewed. Patient demographics, comorbidities, and labor characteristics were obtained from the EMR. SMM cases were defined as those for which an SMM event per Centers for Disease Control criteria occurred after delivery but during the delivery admission. Bivariate, crude, and adjusted multivariate analyses were performed to assess the relationship between patient or labor characteristics and SMM among patients undergoing 2^nd^ stage CS.

**Results**: Of 2680 total 2^nd^ stage CS, 124 patients (4.6%) experienced SMM (Table 1). For reference, the SMM rate among all pregnancies in this system for the same period was 3.8%. Public insurance (aOR 1.86, 95% CI 1.20–2.87), anemia (aOR 1.86, 95% CI 1.20–2.87), and chronic kidney disease (aOR 12.70, 95% CI 4.75–33.97) was associated with increased odds of SMM, as were labor induction (aOR 1.76, 95% CI 1.18–2.61), chorioamnionitis (aOR 1.70, 95% CI 1.08–2.70), and intravenous magnesium (aOR 2.48, 95% CI 1.10–5.61) (Table 2). The duration of the 2^nd^ stage of labor was ≥ 4 hours for most patients (65.3% of those without SMM; 62.2% of those with SMM), and not statistically associated with SMM.

**Conclusion**: In patients undergoing 2^nd^ stage CS, intrapartum events including labor induction, chorioamnionitis, and intravenous magnesium were associated with increased odds of SMM, while most pre‐existing patient characteristics were not. Duration of the 2^nd^ stage ≥ 4 hours was not associated with increased odds of SMM.

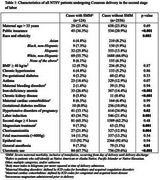

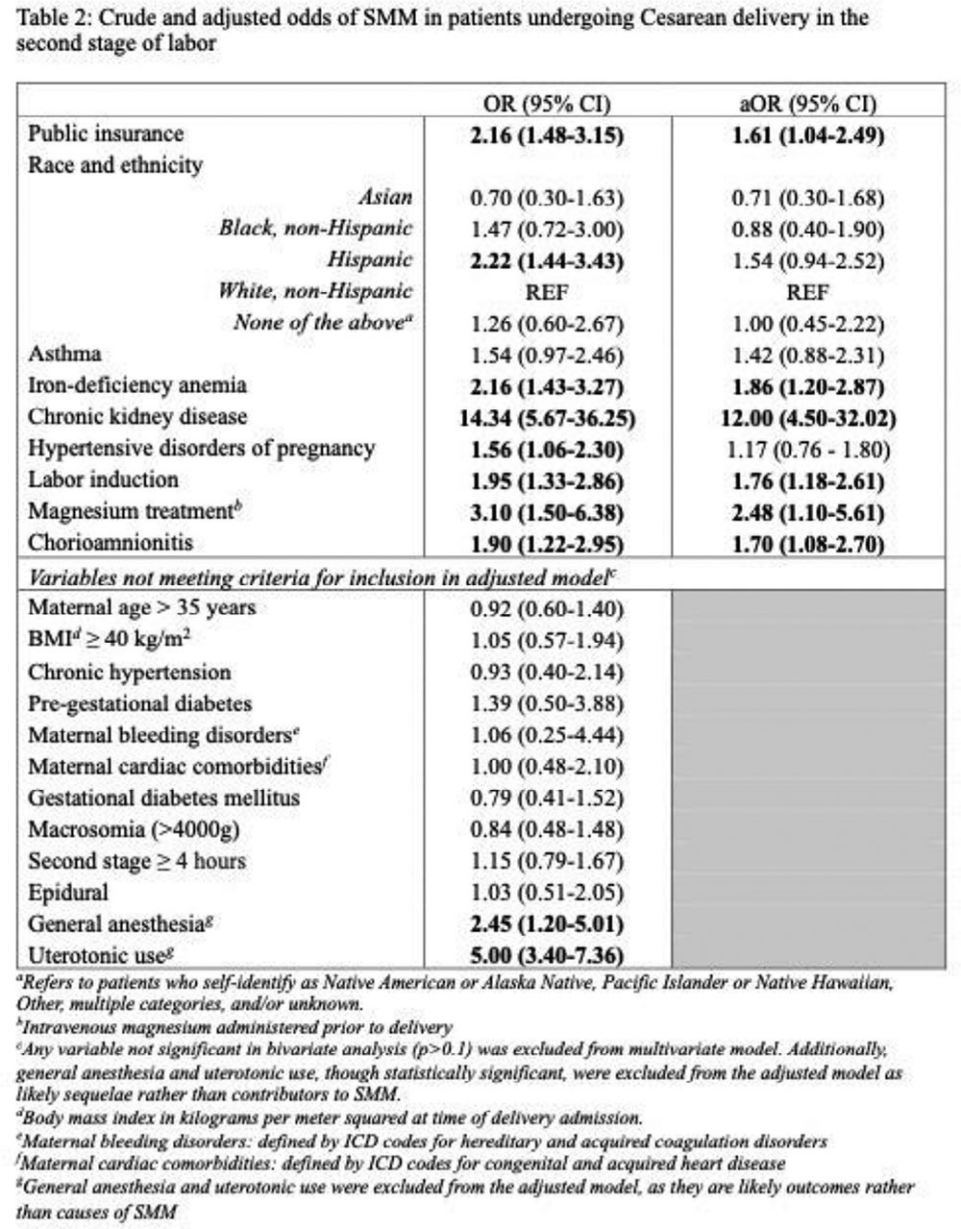

**3:30 PM – 5:00 PM**

## 562 | **Impact of** Interdisciplinary **Clinic on Delivery Mode and Gestational Age in Adult Congenital Heart Disease**

Leslie J. Warren^1^; Renata Mazurek^1^; Elizabeth Cochrane^2^; Marina Schechter^1^; Amare Osei^1^; Raina Kishan^3^; Nicola F. Tavella^4^; Angela T. Bianco^5^; Kali Hopkins^1^; Chelsea A. DeBolt^6^; Ali Zaidi^1^; Lauren A. Ferrara^1^

^1^Icahn School of Medicine at Mount Sinai Hospital, New York, NY; ^2^Hackensack Meridian Health, Hackensack, NJ; ^3^Mount Sinai Hospital, New York, NY; ^4^Division of Maternal‐Fetal Medicine, Icahn School of Medicine, Icahn School of Medicine at Mount Sinai Hospital, New York, NY; ^5^Icahn School of Medicine at Mount Sinai, New York, NY; ^6^Division of Maternal‐Fetal Medicine, Icahn School of Medicine at Mount Sinai, New York, NY

**Objective**: Advances in care have enabled more patients with complex congenital heart disease to reach childbearing age. This study evaluated the impact of an interdisciplinary clinic model on maternal delivery outcomes in this population.

**Study Design**: Retrospective cohort study of patients with adult congenital heart disease (ACHD) who delivered at a single institution before and after the implementation of a collaborative ACHD‐Maternal‐Fetal Medicine (MFM) clinic model in August 2019, focused on interdisciplinary clinic visits and delivery planning. Mode and gestational age of delivery for ACHD patients who delivered before (January 2008‐December 2018) and after (January 2020–December 2024) utilizing the interdisciplinary clinic model were compared.

**Results**: A total of 192 patients with ACHD were identified: 86 delivered before and 106 delivered after the establishment of the interdisciplinary clinic model. All patients (nulliparous and multiparous) were less likely to deliver vaginally prior to the implementation of the joint clinic model [pre/post 42.2% vs 60.0%, OR 0.48 (CI 0.25, 0.89)]. Nulliparous patients alone were also less likely to deliver vaginally prior to implementation of the joint clinic model [pre/post 10.8% vs 22.1%, OR 0.32 (95% CI 0.10, 0.96)] (Table 2). Rate of induction of labor for nulliparous patients was significantly lower prior to the joint clinic model [pre/post OR 0.09 (95% CI 0.02,0.34)]. No significant difference in gestational age of delivery was observed [pre/post 36.3wk vs 37.6wk]. No change in maternal postpartum cardiovascular complications was observed after the joint clinic model, represented as readmission within 6 weeks, postpartum ICU admission, or cardiovascular complication within 12 months.

**Conclusion**: Implementation of an interdisciplinary ACHD‐MFM clinic model was associated with higher likelihood of vaginal delivery among all patients and a higher likelihood of induction of labor for nulliparous patients.

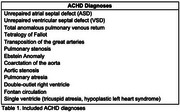

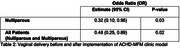

**3:30 PM – 5:00 PM**

## 563 | **Optimal** Antibiotic **Treatment Strategy for GBS‐Negative Individuals Diagnosed With PPROM Prior to 34 Weeks**

Lihi Bakal^1^; Raneen Abu Shqara^2^; Ravit Peretz Machluf^1^; Lital Shaham^3^; Nimrod Dori‐Dayan^4^; Maya Frank Wolf^5^; Shali Mazaki‐Tovi^6^; Michal Fishel Bartal^7^; Noa Gonen^8^

^1^sheba medical center, Ramat Gan, Tel Aviv; ^2^Galilee Medical Center, Nahariya, HaZafon; ^3^Department of Obstetrics and Gynecology, Sheba Medical Center, Sheba Medical Center, HaMerkaz; ^4^Sheba Medical Center, Ramat Gan, HaMerkaz; ^5^Galilee Medical Center, Naharyia, HaZafon; ^6^Department of Obstetrics and Gynecology, Sheba Medical Center, Ramat Gan, Tel Aviv; ^7^Division of Maternal‐Fetal Medicine, Department of Obstetrics, Gynecology and Reproductive Sciences, Sheba Medical Center, Tel Aviv University, Israel, Ramat Gan, HaMerkaz; ^8^Wolfson Medical Center, Holon, HaMerkaz

**Objective**: While broad‐spectrum antibiotics are known to prolong latency in preterm prelabor rupture of membranes (PPROM(up to 34 weeks of gestation, the necessity of 7 days of penicillin prophylaxis in GBS‐negative individuals remains uncertain. We aimed to determine the association between the two regimens and clinical outcomes, including neonatal outcomes, rate of chorioamnionitis, and duration of latency period

**Study Design**: This two‐center retrospective cohort study included all individuals diagnosed with PPROM prior to 34 weeks of gestation and a negative GBS culture between 2018–2024, excluding multiple gestations. Center 1 used the Mercer regimen (2 days IV ampicillin followed by 5 days oral amoxicillin, plus 7 days erythromycin, regardless of GBS status). In contrast, at Center 2, IV ampicillin was given for 2 days and discontinued if the GBS culture was negative, **with erythromycin administered identically in both centers**.

**Results**: The study population included 113 from center 1 and 381 from center 2. Composite neonatal outcome (rates of RDS, TTN, IVH, Neonatal sepsis, Mechanical ventilation and Neonatal death) was more common in center 1 than center 2 (54.9% Vs. 31%, *p* < 0.001), while the rate of chorioamnionitis was significantly lower in center 1 (2.7% Vs. 10.8%, *p* = 0.013) Finally, The median latency period was statistically significantly longer in center 1 compared to center 2 although the difference does not seem to have a clinical importance (median: 3, IQR 2–8 days Vs. median: 3,  IQR 0–10 days *p* = 0.045). The rate of Cesarean section was comparable between the two centers.

**Conclusion**: In the subset of patients with PPROM <34 weeks of gestation and negative GBS culture, a shorter antibiotics treatment regimen was associated with an increased rate of chorioamnionitis and shorter latency period. In contrast, maternal shorter exposure to antibiotics was associated with improved neonatal outcome. Collectively these data suggest that universal implementation of prolonged broad‐spectrum antibiotics requires clinical appraisal.

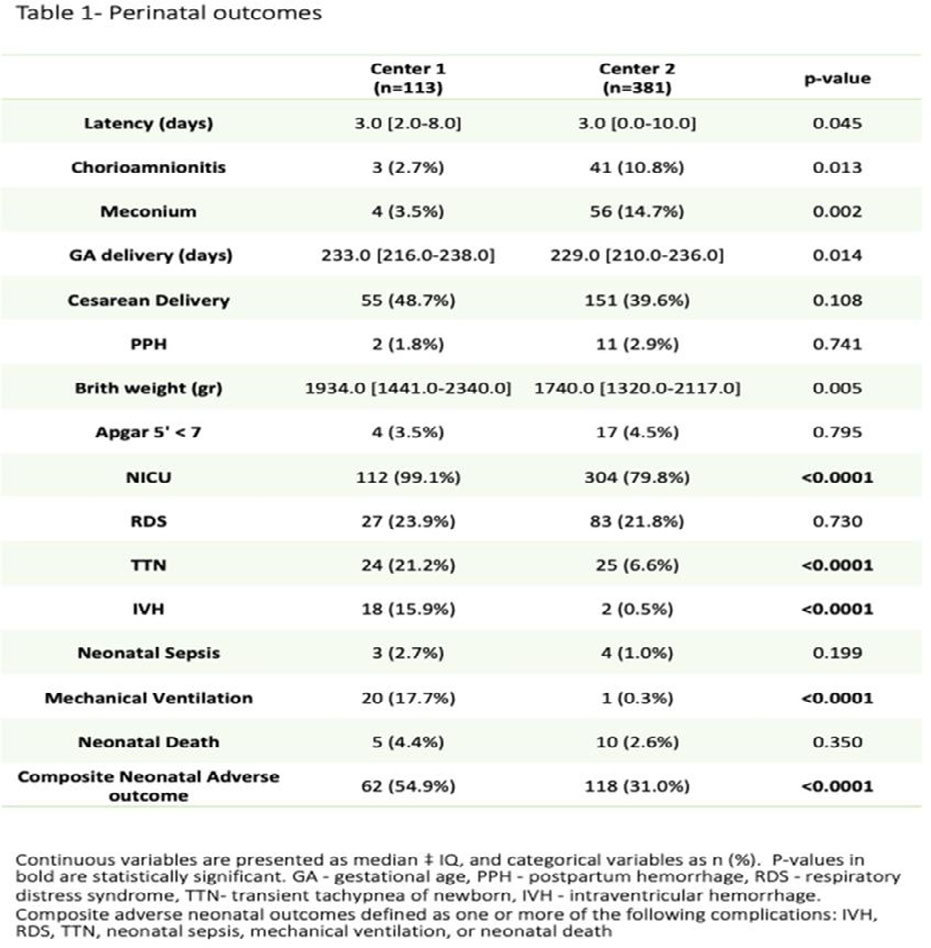

**3:30 PM – 5:00 PM**

## 564 | **Accidental Dural Puncture During Labor–The Impact on Perinatal Outcome**

Lihie Maltz‐Yacobi^1^; Shir Nov^2^; Chaim Greenberger^2^; Tomer Talmi^2^; Anat Lavie^3^; Yariv Yogev^4^; Liran Hiersch^5^; Uri Amikam^6^

^1^Lis Hospital for Women's Health, Tel Aviv Sourasky Medical Center, Tel‐Aviv University, Tel Aviv; ^2^Tel Aviv Sourasky Medical Center, Tel‐Aviv University, Tel Aviv; ^3^Lis Hospital for Women's Health, Tel Aviv Sourasky Medical Center, Lis Hospital for Women's Health, Tel Aviv Sourasky Medical Center, Tel Aviv; ^4^Lis Hospital for Women's Health, Tel Aviv Sourasky Medical Center Gray Faculty of Medicine, Tel Aviv University, Israel, Lis Hospital for Women's Health, Tel Aviv Sourasky Medical Center, Tel Aviv; ^5^Lis Hospital for Women's Health, Tel Aviv Sourasky Medical Center Gray Faculty of Medicine, Tel Aviv University, Israel, ISRAEL, Tel Aviv; ^6^Lis Hospital for Women's Health, Tel Aviv Sourasky Medical Center, Tel Aviv, Tel Aviv

**Objective**: To examine whether accidental dural puncture (ADP) during epidural analgesia in labor is associated with perinatal outcomes.

**Study Design**: A single‐center retrospective cohort study including singleton term (≥37 weeks) vertex, live births with epidural analgesia (2018–2024). The study group included women with ADP. Controls were women with an uneventful epidural anesthesia, performed before and immediately after each case in the study group at a 1:2 ratio. Perinatal outcomes were compared between groups. The primary outcome was composite maternal morbidity, including operative vaginal delivery, cesarean delivery, and postpartum hemorrhage. Secondary outcomes included prolonged second stage of labor (>2 hours for multiparas and >3 hours for nulliparas); urinary retention (need for an indwelling catheter for >24 hours postpartum); hospital readmission within 6 weeks of discharge; and neonatal composite morbidity, including 5‐minute Apgar score <7, arterial pH <7.1, neonatal intensive care unit admission, need for respiratory support, neonatal sepsis, and hypoxic ischemic encephalopathy. Multivariable logistic regression adjusted for confounders. A sub‐analysis was conducted for nulliparas.

**Results**: Overall, 429 patients were included: 143 in the study group and 286 controls. Maternal characteristics were similar between groups (Table), except for a higher nulliparity rate in controls (41.9 vs. 52.8, *p* = 0.03, respectively).

The study group had higher rates of urinary retention (4.2% vs. 0.07%, *p* = 0.01) and readmissions (7.7% vs. 1.7%, *p* < 0.01).

Multivariable logistic regression adjusting for maternal age, pre‐pregnancy BMI, labor induction, assisted reproductive technology use, gestational diabetes, and premature rupture of membranes, demonstrated no association between ADP and composite maternal or neonatal outcomes. Sub‐analysis including only nulliparas showed similar results (Figure).

**Conclusion**: Accidental dural puncture during epidural analgesia in labor was associated with a higher risk of urinary retention and readmission, with similar other maternal and neonatal outcomes.

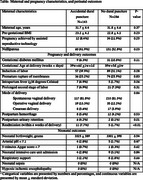

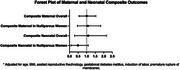

**3:30 PM – 5:00 PM**

## 565 | **Adverse** Pregnancy **Outcomes in Patients With Excessive Gestational Weight Gain versus Pre‐Pregnancy Obesity**

Lily K. Wiedmer^1^; Yuanfan Ye^2^; Moti Gulersen^3^; Ashley N. Battarbee^2^; Ayodeji A. Sanusi^2^

^1^University of Alabama at Birmingham, Birmingham, AL; ^2^Center for Research in Women's Health, University of Alabama at Birmingham, Birmingham, AL; ^3^Division of Maternal Fetal Medicine, Department of Obstetrics, Gynecology and Reproductive Science, Jefferson Health, Philadelphia, PA, Philadelphia, PA

**Objective**: Glucagon‐like peptide‐1 receptor agonists (GLP‐1 RA) are increasingly used to treat obesity but cannot be continued during pregnancy, putting patients at risk of rapid gestational weight gain. Our objective was to model the tradeoffs of GLP‐1 RA by comparing outcomes between individuals with excessive gestational weight gain (GWG) without pre‐pregnancy obesity, versus those with pre‐pregnancy obesity.

**Study Design**: Secondary analysis of a prospective cohort of deliveries at 25 U.S. hospitals (2008–2011). We included pregnant patients with data on pre‐pregnancy/first prenatal visit BMI and GWG. Patients with pregestational diabetes or BMI <18.5 were excluded. The primary outcome was GDM. Baseline characteristics and outcomes were compared between patients with excessive GWG without obesity to those with pre‐pregnancy obesity. Log binomial regression estimated associations between excessive GWG without obesity and outcomes. In sensitivity analyses we 1) restricted the cohort to term deliveries and 2) excluded patients with normal BMI.

**Results**: Of 24,720 included patients, 13,789 (56%) had excessive GWG without obesity and 10,391 (44%) had pre‐pregnancy obesity. Patients with excessive GWG without obesity had mean BMI 24.5 ± 3.0 and GWG 19.2 ± 6.6kg. Patients with pre‐pregnancy obesity had mean BMI 35.9 ± 5.6 and GWG 8.9 ± 9.8kg. After adjusting for covariates that differed between groups, excessive GWG without obesity was associated with a lower risk of GDM (4% vs 12%; aRR 0.36, CI 0.32–0.39), hypertensive disorder, cesarean and maternal ICU admission (Figure & Table). Compared with pre‐pregnancy obesity, excessive GWG without obesity was also associated with lower risk of congenital malformation, preterm birth, LGA, hypoglycemia, NICU admission and severe morbidity (Figure & Table). Findings were consistent in sensitivity analyses.

**Conclusion**: Excessive GWG may be preferable to pre‐pregnancy obesity given lower risk of adverse outcomes. Further research is needed to directly assess the pregnancy benefits of preconception GLP‐1 RA use for treatment of obesity.

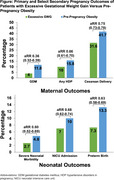

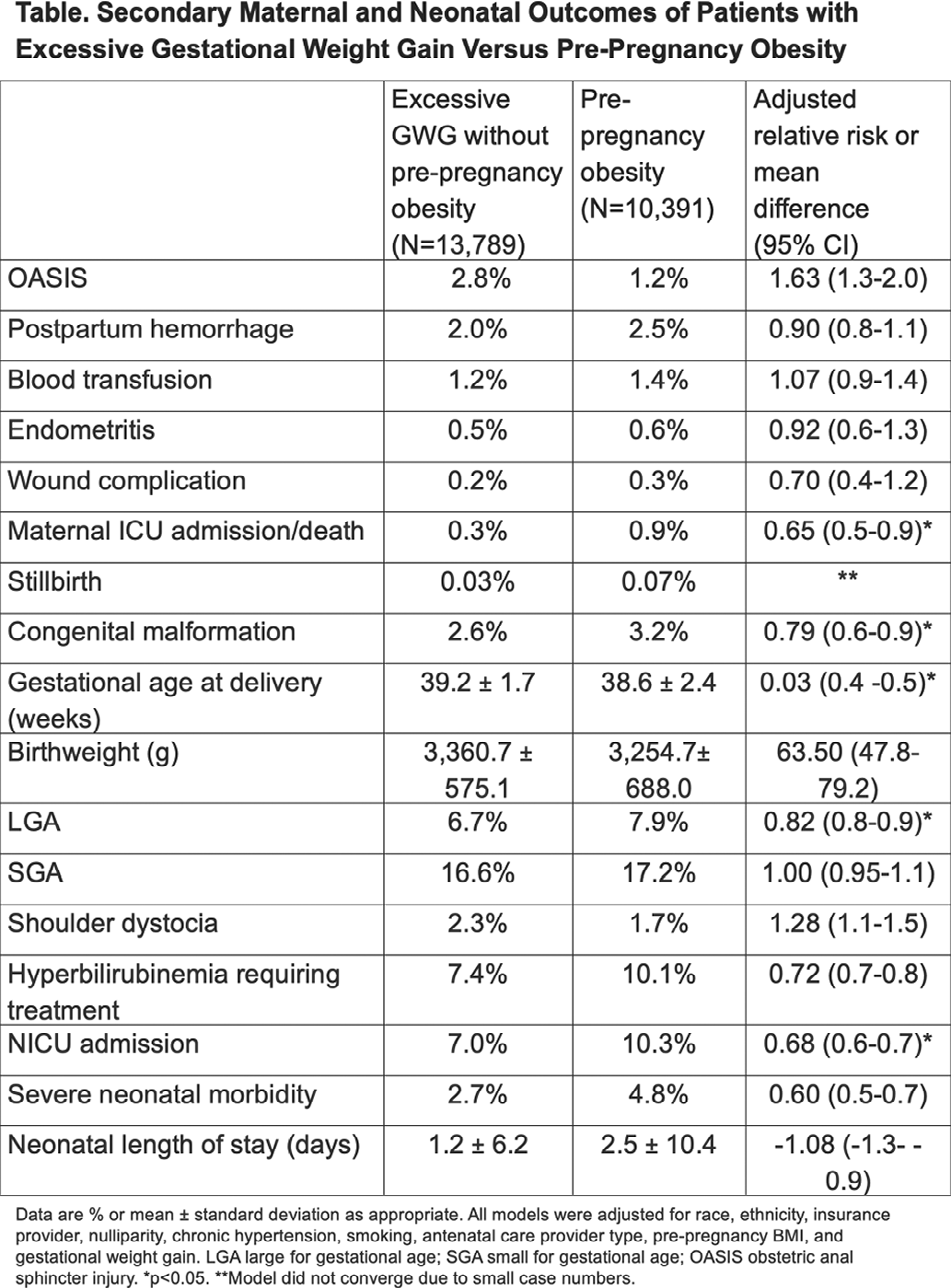

**3:30 PM – 5:00 PM**

## 566 | **Association of** Proteinuria **With Latency in Expectantly Managed Early‐Onset Preeclampsia With Severe Features**

Lina A. Fouad^1^; Yossi Bart^2^; Zakaria Doughan^3^; Joe Haydamous^4^; Ahmed Zaki Moustafa^5^; Baha M. Sibai^2^

^1^Division of Maternal Fetal Medicine, Department of Obstetrics, Gynecology and Reproductive Sciences, McGovern Medical School at UTHealth Houston, Houston, TX; ^2^Division of Maternal‐Fetal Medicine, Department of Obstetrics, Gynecology and Reproductive Sciences, McGovern Medical School at UTHealth Houston, Houston, TX; ^3^Department of Obstetrics and Gynecology, McGovern Medical School at UT Health, Houston, Houston, TX; ^4^Department of Obstetrics, Gynecology and Reproductive Sciences, McGovern Medical School at UTHealth Houston, Department of Obstetrics and Gynecology, McGovern Medical School at UT Health, Houston, TX; ^5^Division of Maternal‐Fetal Medicine, Department of Obstetrics, Gynecology and Reproductive Sciences, McGovern Medical School at UTHealth Houston, University of Texas ‐ Houston, TX

**Objective**: Although the role of proteinuria in the classification and stratification of preeclampsia has declined over time, it is still a marker of kidney dysfunction in those with serum creatinine of <1.1 mg/dl. This study aimed to evaluate if presence of proteinuria (urine protein‐creatinine ratio [UPCr] ≥0.3 or 24‐hour urine protein ≥300 mg) in expectantly managed preeclampsia with severe features before 33 weeks was associated with latency from diagnosis to delivery.

**Study Design**: A single center retrospective cohort study from 2018 to 2025 including singletons diagnosed with preeclampsia with severe features between 23 weeks 0 days and 32 weeks 6 days gestation for whom urine protein was available. Exclusion criteria were chronic hypertension or contraindications to expectant management (placental abruption, intrauterine fetal demise, acute kidney injury, pulmonary edema, HELLP syndrome, eclampsia). The primary outcome was latency (days) between those patients with and without proteinuria. Following adjustment, multivariable linear regression was used to calculate the effect size (B) with 95% confidence intervals (CI).

**Results**: 160 patients were identified; 128 (80%) had proteinuria, and 32 (20%) did not. Those with proteinuria had higher rates of pregestational diabetes (12.5% vs. 0%, *p* = 0.04). Median latency in patients without proteinuria was longer (9.5, IQR 3–20) than in patients with proteinuria (4, IQR 2–9) (*p* = 0.02). Following adjustment for pregestational diabetes, the presence of proteinuria was associated with shorter latency (OR 5.59, 95% CI 1.34–9.83). Additionally, higher UPCr levels were associated with shorter latency in a dose‐dependent manner (*p* = 0.01; Figure). The rate of composite maternal adverse outcomes did not differ between groups (Table).

**Conclusion**: Among expectantly managed patients, the presence of proteinuria was associated with a more than 5‐day shorter latency period. These findings suggest that proteinuria may be a useful predictor of latency and could be considered in the design of clinical trials focused on prolonging pregnancy in this population.

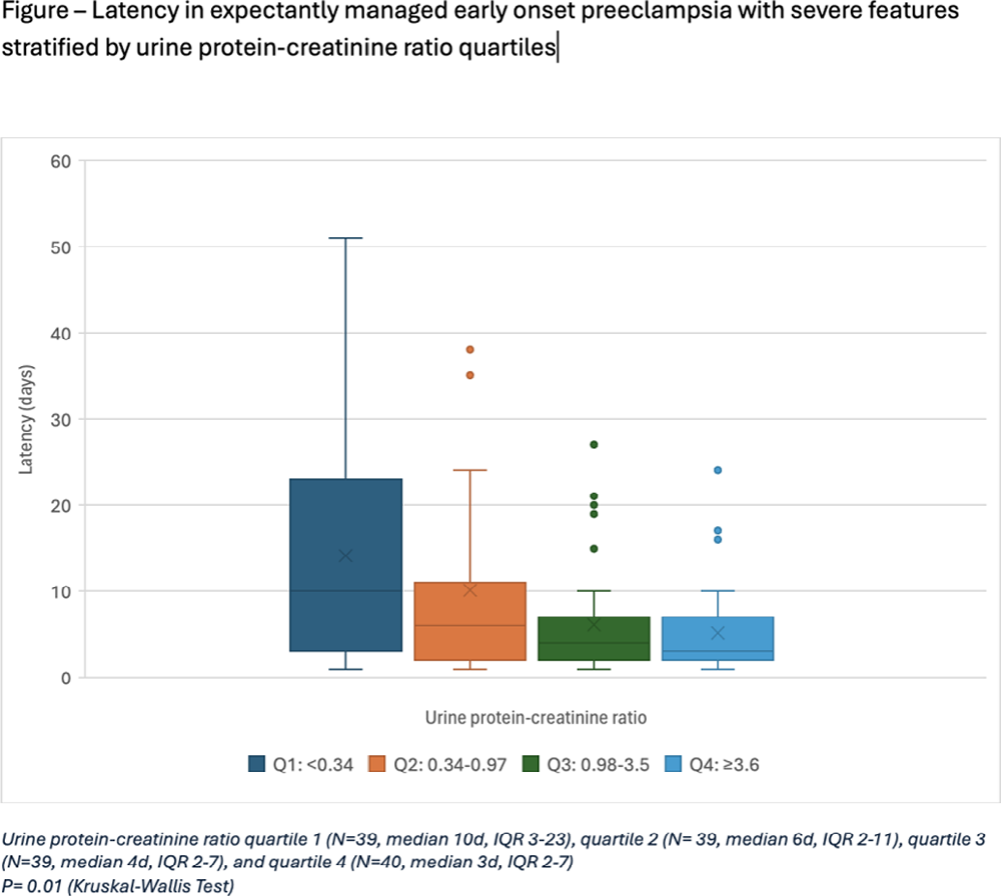

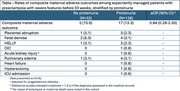

**3:30 PM – 5:00 PM**

## 567 | **Association** Between **Cerebroplacental Ratio and Placenta Regardless of Birthweight: Secondary Analysis of the IMPACT‐BCN Trial**

Lina Youssef^1^; Rommy H. Novoa^1^; Ohad Houri^2^; Giulia Ferrante^1^; Arianna Pizzetti^1^; Roberta Castellani^1^; Leticia Benitez^1^; Irene Casas^1^; Mariona Genero^1^; Francesca Crovetto^3^; Eduard Gratacós^4^; Fatima Crispi^5^

^1^BCNatal–Barcelona Center for Maternal‐Fetal and Neonatal Medicine (Hospital Clínic and Hospital Sant Joan de Deu), IDIBAPS, University of Barcelona, Barcelona, Catalonia; ^2^BCNatal Fetal Medicine Research Center, BARCELONA, Catalonia; ^3^BCNatal–Fetal Medicine Research Center (Hospital Clínic and Hospital Sant Joan de Déu), Institut d'Investigacions Biomèdiques August Pi i Sunyer (IDIBAPS), Barcelona, Catalonia; ^4^Hospital Clínic Barcelona, BARCELONA, Catalonia; ^5^Hospital clinic Barcelona, BARCELONA, Catalonia

**Objective**: To evaluate the association between cerebroplacental ratio (CPR) and placental histopathology, independent of estimated fetal weight (EFW), in fetuses from high‐risk pregnancies.

**Study Design**: This was a secondary analysis of the IMPACT‐BCN randomized clinical trial, including 707 singleton pregnancies at high risk for small‐for‐gestational‐age (SGA) outcomes, defined by an EFW and birthweight below the 10th centile. At 33–34 weeks’ gestation, fetal ultrasound assessments were performed to obtain EFW and CPR, calculated as the ratio of the middle cerebral artery to umbilical artery pulsatility indices. An abnormal CPR was defined as <5th centile. At delivery, placental weight and histopathological lesions were recorded and categorized as vascular (maternal/fetal), immune‐inflammatory, or other, following the 2014 Amsterdam Placental Workshop Group Consensus criteria.

**Results**: Placental characteristics were analyzed across four groups: appropriate‐for‐gestational‐age (AGA) fetuses with normal CPR (*n* = 554) or abnormal CPR (*n* = 21), and SGA fetuses with normal CPR (*n* = 112) or abnormal CPR (*n* = 20). Placental weight was significantly lower in both SGA groups compared to AGA groups, regardless of CPR status (315 ± 83 g and 362 ± 63 g vs. 449 ± 99 g and 462 ± 92 g, respectively). No significant differences were observed among groups for vascular or infectious lesions. However, immune‐inflammatory lesions were significantly more prevalent in SGA fetuses (with and without abnormal CPR) and in AGA fetuses with abnormal CPR, compared to AGA fetuses with normal CPR (25%, 17%, and 28.6% vs. 9.4%, respectively; *p* = 0.002).

**Conclusion**: An abnormal CPR in the third trimester is associated with an increased prevalence of placental immune lesions, independent of fetal growth status. These findings highlight the potential role of CPR as a marker of placental immune dysregulation beyond fetal size assessment.

**3:30 PM – 5:00 PM**

## 568 | **Hindbrain** Herniation **Reversal and CSF Diversion Following Fetoscopic Spina Bifida Repair With Umbilical Cord Patch**

Linoy Batsry, N/A^1^; Rajan Patel^2^; Lovepreet Mann^3^; Clifton O. Brock^4^; Sen Zhu^3^; Elisa Garcia^3^; Ashley Salazar^3^; Jimmy Espinoza^3^; Stephen A. Fletcher^5^; Kuojen Tsao^6^; Peter Yang^1^; Mary Austin^6^; Ramesha Papanna^7^

^1^UTHealth, houston, TX; ^2^TCH, houston, TX; ^3^UTHealth Houston Fetal Institute; Division of Fetal Intervention, Department of Obstetrics, Gynecology and Reproductive Sciences, McGovern Medical School at UTHealth Houston, Houston, TX; ^4^Midwest Fetal Care Center, Children's Minnesota, Minneapolis, Minnesota, minneapolis, MN; ^5^Division of Pediatric Neurosurgery, Department of Pediatric Surgery, McGovern Medical School at UTHealth Houston, Houston, TX; ^6^Division of Pediatric General & Thoracic Surgery, Department of Pediatric Surgery, McGovern Medical School at UTHealth Houston, Houston, TX; ^7^University of Texas Health Science Center in Houston, McGovern Medical School, Houston, TX

**Objective**: In‐utero closure of spina bifida improves hindbrain herniation and reduces the need for cerebrospinal fluid (CSF) diversion (ventriculoperitoneal shunt or endoscopic third ventriculostomy). Fetoscopic spina bifida repair with human umbilical cord patch (fSB‐HUC) uses a patch to create a watertight closure compared to the myofascial closure of open in‐utero repair. This study compares rates of hindbrain herniation reversal (HHR) on MRI and CSF diversion at 12 months following fSB‐HUC versus open in‐utero repair.

**Study Design**: This is a secondary analysis comparing patients from a single‐arm interventional study conducted at a single tertiary center to a prospective cohort and historical control groups of patients who underwent open in‐utero repair at the same institution (oSB), as well as to a de‐identified individual‐level database from the open in‐utero repair group from the MOMS trial (MOMS). Patients eligible for open in‐utero repair were offered fSB‐HUC. Following placode dissection, the HUC patch was sutured circumferentially; skin closure was then performed with or without an additional patch. Preoperative neuroradiological features and 12‐month outcomes were compared between the three groups. The primary outcomes were complete HHR on 12‐month MRI (defined as the obex at or above the level of the foramen magnum) and CSF diversion within 12 months.

**Results**: Of 49 patients who underwent fSB‐HUC, 46 completed 12 months MRI and were included in the analysis (Table 1). The rate of complete HHR was significantly higher in the fSB‐HUC group compared to the MOMS group (*p* = 0.010). Rates of CSF diversion within the first year of life were comparable across the three groups (*p* = 0.592) and were not influenced by HHR status. In the fSB‐HUC group, the pre‐procedure hindbrain herniation improved in 41/46 (89%) compared to 12 months degree of hindbrain herniation on MRI (*p* < 0.001, Figure 1).

**Conclusion**: fSB‐HUC was associated with higher rates of complete HHR compared to MOMS, and comparable CSF diversion rates within 12 months compared to both oSB and MOMS.

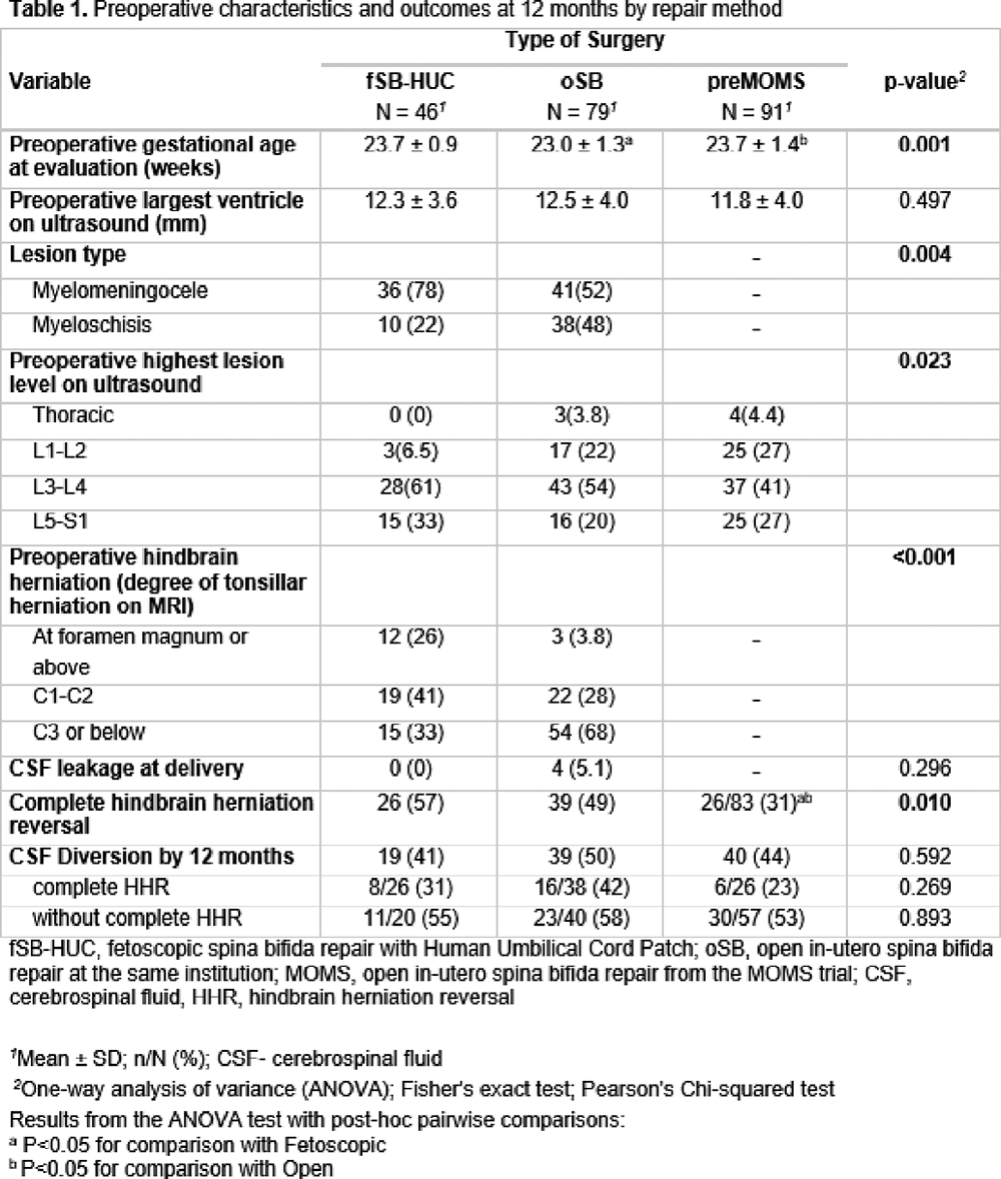

**3:30 PM – 5:00 PM**

## 569 | **Clinical Impact of Fetal Weight Estimation Errors on Maternal and Neonatal Outcomes**

Lior Heresco^1^; Sharon Kronkop^2^; Chaya Ben Yehuda^3^; Hadar Gluska^1^; Tal Biron‐Shental^1^; Omer Weitzner^1^

^1^Meir Medical Center, Kfar Saba, HaMerkaz; ^2^Ben‐Gurion University of the Negev, Beer Sheva, HaDarom; ^3^Clalit Health Services, Tel Aviv, HaMerkaz

**Objective**: Accurate fetal weight estimation (EFW) is essential in obstetric care yet commonly exhibits error margins of 10–15%.Limited data exist regarding the clinical consequences of EFW inaccuracies. This study aimed to assess maternal and neonatal outcomes in relation to EFW error percentage.

**Study Design**: This retrospective cohort study included patients with singleton pregnancies delivered between 2014–2023, with an EFW performed within 14 days prior to delivery. EFW error was calculated as the percentage difference between ultrasound‐estimated and actual birthweight. Patients were categorized into two groups: <10% and ≥10% EFW error. Baseline characteristics and outcomes were compared, and adjusted multivariate regression was used to assess the association between error level and risk of cesarean delivery (CD).

**Results**: A total of 19,338 patients were included; 14,410 (74.5%) had EFW error <10%, and 4820 (25.5%) had error ≥10%. The ≥10% group had higher rates of nulliparity (35% vs. 32%, *p* < 0.001), delivered slightly earlier (39.4 vs. 39.5 weeks, *p* = 0.001), and had lower maternal height (162.8 vs. 163.4 cm, *p* = 0.007).CD was more frequent in the ≥10% group (19.1% vs. 17.1%, *p* = 0.002), both for labor arrest (3.3% vs. 3.2%, *p* = 0.04) and fetal distress (7.6% vs. 6.2%, *p* = 0.04).No differences were observed in CD type (elective vs. urgent).Neonates in the ≥10% group had lower birthweight (3276 vs. 3381 g, *p* = 0.001), with higher rates of macrosomia (>4000g: 9.4% vs. 8.1%, *p* = 0.007) and low birthweight (<2500g: 6.6% vs. 3.3%, *p* < 0.001). In multivariate analysis, ≥10% error was not associated with an increased risk of CD (OR 0.98; 95% CI 0.86–1.12), whereas an error of ≥20% significantly raised the risk of CD (OR 1.68; 95% CI 1.27–2.22).

**Conclusion**: An EFW error of ≥10% was not independently linked to an increased risk of CD, while errors of ≥20% significantly elevated the risk. These findings provide reassurance regarding typical error margins but highlight the importance of caution and repeated expert evaluation in cases at high risk for inaccurate fetal weight assessment.

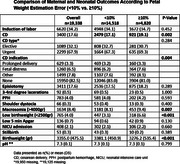

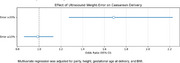

**3:30 PM – 5:00 PM**

## 570 | **Delayed Pushing in Second Stage: Variation in De‐Implementation in Real World Clinical Settings**

Lisa Kane Low^1^; Michelle Moniz^2^; Molly J. Stout^1^; Xilin Chen^1^; Althea Bourdeau^1^; Madonna Ladouceur^3^; Jessi Ems^1^; Eileen Suse^4^; Jourdan E. Triebwasser^1^

^1^University of Michigan, Ann Arbor, MI; ^2^University of Michigan, University of Michigan, MI; ^3^Detroit Medical Center Huron Valley‐Sinai Hospital, Detroit, MI; ^4^Obstetrics Initiative, Ann Arbor,, MI

**Objective**: The American College of Obstetricians and Gynecologists (ACOG) recommends that pushing commence when complete cervical dilation is achieved, rather than delaying pushing or “laboring down,” particularly when an epidural is in place. In a statewide quality collaborative, we assess the use of this evidence‐based practice in a **n**ulliparous **t**erm, **s**ingleton, **v**ertex (NTSV) population

**Study Design**: Retrospective cohort study of NTSV births from 1/1/2020– 12/31/2024 in the clinical registry of the Obstetrics Initiative, a state‐wide quality collaborative supported by Blue Cross Blue Shield of Michigan and Blue Care Network as part of the BCBSM Value Partnerships program. We included births with an epidural in place, preceded by spontaneous or induced labor progressing to the 2nd stage. Our primary exposure was delayed pushing, defined as active pushing initiated at >60 minutes after complete cervical dilation. Descriptive statistics characterized delayed pushing rates over time and across individual hospitals, and generalized linear mixed models evaluated associations between delayed pushing and maternal and neonatal outcomes, controlling for covariates.

**Results**: Among 84,771 births at 72 hospitals, the collaborative‐wide rate of delayed pushing decreased from a high of 16.8% in 2020 to 12.8% (*p* < 0.05) in 2024 (Figure 1, A). Individual hospital rates of delayed pushing still varied widely in 2024 (range: 2.3% to 31.9%; Figure 1, B). After adjusting for covariates, births after delayed vs. immediate pushing had significantly increased odds of multiple maternal complications, including cesarean (OR 1.66, 95% CI 1.56 to 1.77), hemorrhage (OR 1.23, 95% CI 1.14 to 1.33), and operative vaginal birth (OR 1.19, 95% CI 1.11 to 1.27; Table 1), consistent with findings in earlier clinical trials.

**Conclusion**: Delayed pushing remains a common practice associated with harm in real‐world maternity settings. Given the established risks of delayed pushing, quality improvement interventions are urgently needed to increase use of immediate pushing in the second stage.

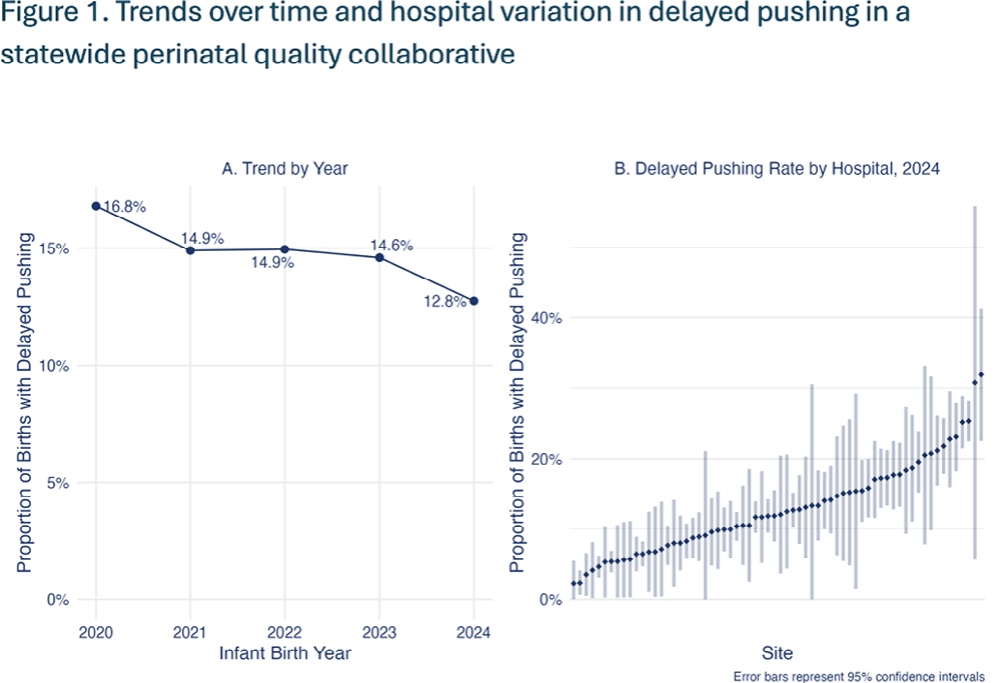

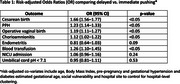

**3:30 PM – 5:00 PM**

## 571 | **Prediction of** Preterm **Birth in Twin Pregnancies Using Cervical Length and Baseline Clinical Variables**

Lorraine Dugoff

On behalf of the Eunice Kennedy Shriver National Institute of Child Health and Human Development (NICHD) Maternal‐Fetal Medicine Units (MFMU) Network, Bethesda, MD

Perelman School of Medicine at the University of Pennsylvania, Philadelphia, PA

**Objective**: To develop a model to predict early delivery in twin pregnancies.

**Study Design**: Secondary analysis of a clinical trial which randomized women carrying twin pregnancies and a cervical length <30 mm to Arabin cervical pessary, vaginal progesterone, or placebo from 16 weeks 0 days through 23 weeks 6 days. We examined various endpoints for early delivery. For the primary analysis, a bootstrapped resampling method was used to develop and internally validate a Cox Proportional Hazards (PH) model for the outcome of gestational age at delivery as a continuous measure. Secondarily, logistic models to predict PTB at <24, <28, and <32 weeks were created. Baseline enrollment factors associated with PTB <32 weeks were assessed in all models. Predictors retained in at least 75% of the 1000 bootstrapped models were selected for the final models. A Kaplan‐Meier curve was created to demonstrate the probability of remaining pregnant across GAs in weeks.

**Results**: A total of 437 pregnancies were included. Variables predicting PTB in the final CoxPH model included cervical length (CL) (HR 0.66, 95% CI 0.54, 0.79), GA when qualifying CL was measured (HR 0.59 95% CI 0.49, 0.72), and the interaction between them (HR 1.02 95% CI 1.01, 1.03). Logistic models with predictors of CL and gestation at the time of CL measurement had moderate to excellent prediction of PTB prior to 24, 28 and 32 weeks (Table). The Kaplan‐Meier curve illustrated the relationship between the covariates and GA at delivery. For example, median delivery GA if CL was <15 mm prior to 20 weeks GA was 23 weeks, but increased to 31 weeks if not less than 15 mm until after 20 weeks (Figure, *p* < 0.001).

**Conclusion**: Cervical length and the gestational age at diagnosis of shortened cervical length accurately predicted GA at delivery for twin pregnancies with a cervix <30 mm, as well as preterm birth at GAs less than 32 weeks. Model performance was excellent when predicting very early preterm births at less than 24 weeks.

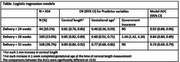

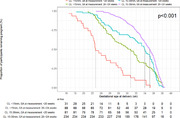

**3:30 PM – 5:00 PM**

## 572 | **Association of** Duration **of Breastfeeding With Child Neurodevelopment at 18 to 30 Months**

Lynn M. Yee

On behalf of the Eunice Kennedy Shriver National Institute of Child Health and Human Development (NICHD) Maternal‐Fetal Medicine Units (MFMU) Network, Bethesda, MD

Northwestern University Feinberg School of Medicine, Chicago, IL

**Objective**: Although breastfeeding is associated with many maternal and infant health benefits, it is unknown whether the duration of breastfeeding is associated with offspring neurodevelopmental outcomes. We evaluated the association of duration of breastfeeding with neurodevelopmental outcomes at 18 to 30 months of age.

**Study Design**: This is a secondary analysis of a multisite, prospective observational study assessing outcomes after SARS‐CoV‐2 vaccination during or up to 30 days before pregnancy. This analysis evaluated neurodevelopmental outcomes of 18–30 month old children born at term as singleton gestations, who initiated breastfeeding, had a known breastfeeding duration, and had complete primary outcome data. At 18–30 months, caregivers completed study visits that included breastfeeding queries and measures of neurodevelopment. The primary outcome was the Ages and Stages Questionnaire version 3 (ASQ) total score. Secondary outcomes included domain‐specific ASQ scores, the Child Behavior Checklist (CBCL), Modified Checklist for Autism in Toddlers (MCHAT), and Early Childhood Behavior Questionnaire (ECBQ). Differences in each outcome were compared by breastfeeding duration (<3 versus ≥3 months), adjusting for maternal age, education, SARS‐CoV‐2 vaccination during pregnancy, and study center.

**Results**: Of 511 participants in the primary study, 428 (84%) were eligible for inclusion. A majority (*N* = 292, 68%) breastfed for ≥3 months. Those who breastfed ≥3 months were older, more likely to have some college education, and to be multiparous. There was no difference in offspring ASQ total score by breastfeeding duration, although those who were breastfed ≥3 months had a reduced risk of a score >2 SDs below mean on the ASQ problem‐solving domain (aRR 0.3, 95% CI 0.1–0.6) (Table 1). No clinically significant differences were noted in total or component scores for the CBCL, MCHAT, or ECBQ (Table 2).

**Conclusion**: Among children born at term who initiated breastfeeding, there was no association between being fed breastmilk for ≥3 months and neurodevelopmental outcomes at 18–30 months of age.

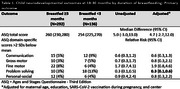

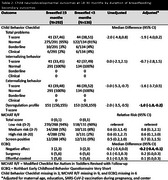

**3:30 PM – 5:00 PM**

## 573 | **Does Metformin Use in Pregnancy Alter Glucose Tolerance Postpartum in Patients With Gestational Diabetes?**

Macy M. Walz^1^; Alissa Prior^2^; Esha Ghosalkar^3^; Christopher Nau^4^

^1^University Hospitals Cleveland Medical Center, University Hospitals/Cleveland, OH; ^2^Metrohealth/Case Western Reserve University Program, Cleveland, OH; ^3^University Hospitals, University Hospitals, OH; ^4^University Hospitals Cleveland Medical Center, Cleveland, OH

**Objective**: Animal studies suggest use of metformin in pregnancy may increase insulin resistance during pregnancy and postpartum. We aimed to investigate the association between treatment of GDM with metformin vs insulin and the incidence of dysglycemia postpartum.

**Study Design**: A retrospective cohort was conducted using the United States Collaborative Network in TriNetX. Patients aged 18 to 50 with a diagnosis of medication controlled GDM after 24 weeks gestation were included. Two groups were defined as GDM with metformin and GDM with insulin. Cohorts were matched for age, race, ethnicity, BMI, and primary hypertension (HTN). The primary outcome of dysglycemia was assessed at 1 to 2 years after pregnancy and defined by laboratory measurements (HbA1C, 2‐hour oral glucose tolerance testing, and fasting glucose) and ICD‐10 codes for diabetes and prediabetes. Other outcomes of interest included HTN, hyperlipidemia, and cardiovascular disease postpartum. Logistic regression was performed to determine odds ratios (OR) and 95% confidence intervals (CI).

**Results**: After matching, there were 10,362 individuals in each group. Individuals with a metformin prescription were more likely to develop prediabetes at 1 to 2 years postpartum based on ICD‐10 codes (4.5% vs 2.2%, OR 2.06 (CI 1.75, 2.42)) and laboratory diagnosis (5.4% vs 3.8%, OR 1.45 (CI 1.27, 1.65)) compared to those prescribed insulin alone (Table 1). The risk of developing diabetes based on ICD‐10 codes (OR 1.04 (CI 0.92, 1.18)) and laboratory diagnosis (OR 0.94 (CI 0.75, 1.16)) was not different between groups. There was also an increased risk of hyperlipidemia (3.8% vs 2.5%, OR 1.5 (CI1.29, 1.78)) among the metformin group, though no difference in diagnosis of HTN.

**Conclusion**: Treatment with metformin during a pregnancy complicated by GDM is associated with an increased risk of developing prediabetes 1 to 2 years after pregnancy. This could suggest metformin use in pregnancy may impact glucose tolerance beyond pregnancy. Further investigation is warranted to explore potential residual confounding and physiologic mechanism.

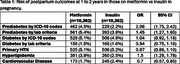

**3:30 PM – 5:00 PM**

## 574 | **Aortopathies and Hypertrophic Cardiomyopathy in Pregnancy**

Madeline P. McKenna^1^; Marisa R. Imbroane^2^; Dana Baraki^3^; Ahmed Ahmed^4^; Amol Malshe^4^

^1^Cleveland Clinic Akron General, Akron, OH; ^2^Case Western Reserve University School of Medicine, Case Western Reserve University School of Medicine/Cleveland, OH; ^3^Cleveland Clinic Foundation, Cleveland, OH; ^4^Cleveland Clinic Foundation, Cleveland Clinic Foundation, OH

**Objective**: To assess obstetric risks in patients with aortic dissection, thoracic/abdominal aneurysms, and hypertrophic obstructive cardiomyopathy (HOCM), compared to matched controls.

**Study Design**: We conducted a retrospective cohort study using the US collaborative network on the TriNetX database, including patients with outcomes of interest from the last 20 years. ICD‐10 codes were used to identify and evaluate pregnancy outcomes in patients with aortopathies (dissection, thoracic, and abdominal aneurysms) and HOCM. Relative risk (RR) and 95% confidence intervals (CI) were calculated to assess the key outcomes of hypertensive disorders, fetal growth restriction (FGR), intrauterine fetal demise (IUFD), and preterm birth.

**Results**: Aortic dissection was associated with a more than twofold increased risk of both preeclampsia (11.9% vs 4.9%) and preterm birth (4.9% vs 2.2%). Thoracic aneurysms significantly increased the risk of gestational hypertension (gHTN) (7.0%) and preterm delivery (3.6%) compared to controls. Abdominal aneurysms presented a striking 2.38‐fold elevated risk of FGR (5.9% vs 2.5%). When combined, thoracic and abdominal aneurysms were associated with increased rates of preeclampsia (6.2%), gHTN (6.6%), FGR (5.2%), placental abruption (1.0%), and preterm birth (3.4%). HOCM was also high risk, associated with over twofold increases in both FGR and preterm delivery compared to controls. All noted findings were statistically significant with *p* < 0.0001.

**Conclusion**: Aortopathies and HOCM confer significant obstetric risk, particularly for hypertensive disease, FGR, and preterm birth. These outcomes likely reflect disrupted maternal cardiovascular adaptation and altered perfusion, implicating hemodynamic compromise as a contributing factor to poor placental function and fetal growth. These findings underscore the importance of tailored multidisciplinary care and close antenatal surveillance for pregnant patients with aortopathies and HOCM to mitigate maternal and fetal risks.

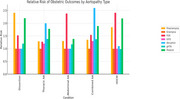

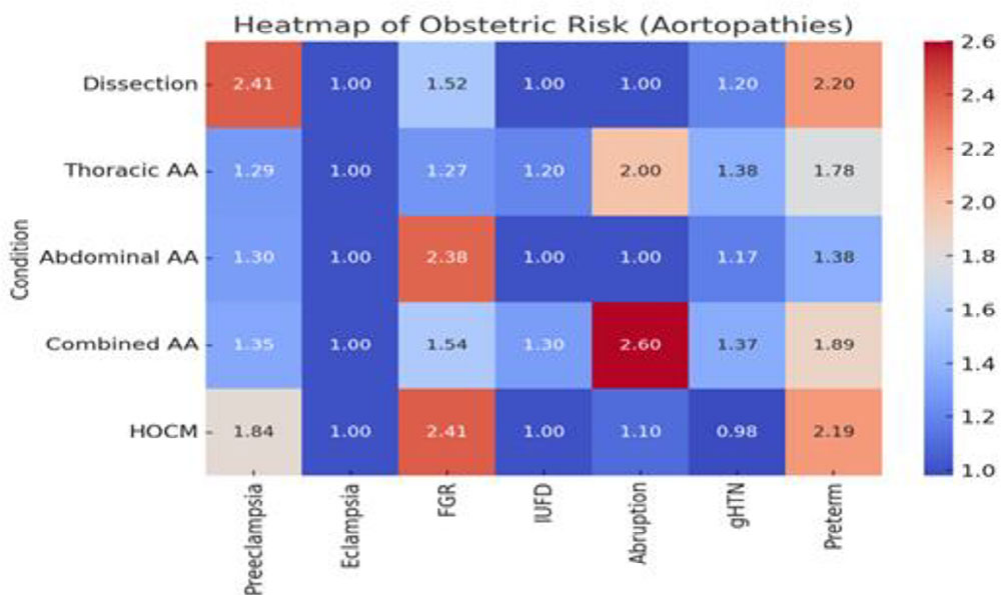

**3:30 PM – 5:00 PM**

## 575 | **Are Women** Enrolled **in a Postpartum Monitoring App More Likely to Receive Lactation Care Postpartum?**

Madison Witherington; Caroline Centeno; Sapna K. Patel; Amie Goodin; Jungjun Bae; Kay Roussos‐Ross

University of Florida College of Medicine, Gainesville, FL

**Objective**: Lactation consultation has been shown to improve both breastfeeding duration and experience. Data is limited on how postpartum monitoring apps affect lactation consult use. MOMitor™ is a post‐partum app that monitors both the mental and physical health of participants. We examined lactation consultation utilization among women using the MOMitor™ app compared to those not using the app within one year postpartum.

**Study Design**: This cross‐sectional, descriptive analysis retrospectively reviewed electronic health records from 2021–2023 to observe postpartum lactation visit frequency between 919 MOMitor™ app users and 627 non‐users. Patients enrolled in the app received weekly questions asking if they were having any breastfeeding complications, if they would like to be contacted by a nurse to discuss concerns, and whether they would like to be referred for lactation care. Bivariate analysis with chi‐squared testing was used to assess differences in healthcare utilization.

**Results**: A total of 1546 charts were reviewed. 919 charts were MOMitor™ participants, and 627 were controls. There were 260 MOMitor™ patients with at least one lactation visit (28.29%) and 54 controls with at least one lactation visit (8.61%). A statistically significantly greater proportion of MOMitor participants, as compared with controls, received lactation consultant care in the postpartum period (*p* < 0.001).

**Conclusion**: Participation in a post‐partum monitoring app significantly increases the use of lactation consultation care in the post‐partum period. This is likely due to the questionnaires and access to referrals through the MOMitor™ app. Further studies could investigate whether breastfeeding durations were influenced by increased lactation support.

**3:30 PM – 5:00 PM**

## 576 | **Beyond** Ultrasound**: MRI for Fetal Neuroanatomy Evaluation Following Laser Surgery for Twin‐Twin Transfusion Syndrome**

Manasa G. Rao^1^; Lynn L. Simpson^2^; Rosalie Ingrassia^3^; Noelle Breslin^4^; Chia‐Ling Nhan‐Chang^5^; Russell S. Miller^6^

^1^Columbia University, New York, NY; ^2^Division of Maternal‐Fetal Medicine, Department of Obstetrics and Gynecology, Columbia University Irving Medical Center, new york, NY; ^3^Columbia University Medical Center, New York, NY; ^4^Northside Hospital, Atlanta, GA; ^5^Division of Maternal Fetal Medicine, Department of Obstetrics, Gynecology and Reproductive Sciences, San Diego, CA; ^6^Division of Maternal‐Fetal Medicine, Department of Obstetrics and Gynecology, Columbia University Irving Medical Center, New York, NY

**Objective**: Fetoscopic laser surgery (FLS) for twin‐twin transfusion syndrome (TTTS) appears to reduce neurologic morbidity in surviving monochorionic diamniotic (MCDA) twins. However, abnormal fetal neuroimaging is reported in 2–6% of cases after FLS, and the utility of fetal MRI as an adjunct to ultrasound (US) is unclear. This study aimed to determine the frequency of abnormal fetal MRI findings post‐FLS and assess associated perinatal factors.

**Study Design**: Retrospective review of MCDA twin pregnancies with TTTS treated with FLS and subsequent fetal MRI at a single center from 2009–24. Univariable and Firth multivariable logistic regression were used to evaluate associations between clinical factors and abnormal MRI findings.

**Results**: Fetal MRI was available for 163 of 298 (54.7%) pregnancies. Mean gestational age (GA) at FLS was 19w6d (+/‐3d) and at MRI was 22w6d (+/‐2d). Twelve patients (7.3%) had abnormal MRIs. Common findings included intraventricular hemorrhage (*n* = 7, 58.3%), ventriculomegaly (*n* = 7, 58.3%), and infarction (*n* = 6, 50%). When correlating with fetal US images, MRI provided additional neurologic findings in 9 (75%) cases. Donor twins had more lesions (11 fetuses, 18 lesions) than recipients (1 fetus, 4 lesions). Neonatal neuroimaging was available for 5 patients. Of those, 3 (60%) had neonatal US/MRI findings correlating to the abnormal fetal MRI. On univariable analysis, factors such as recurrent TTTS, TAPS, pre‐MRI PPROM, growth discordance, co‐twin demise, BMI, age, and nulliparity were directionally associated with abnormal MRI but not statistically significant. Firth multivariable analysis controlling for age, nulliparity, TTTS stage, co‐twin demise and inter‐twin discordance found no independent risk factors.

**Conclusion**: Abnormal fetal MRI findings occurred in 1 in 13 pregnancies after FLS. Most findings were not obvious on post‐FLS surveillance ultrasound. No discrete perinatal risk factors were associated with abnormal MRI. Based on these data, we recommend fetal MRI surveillance for all post‐FLS patients.

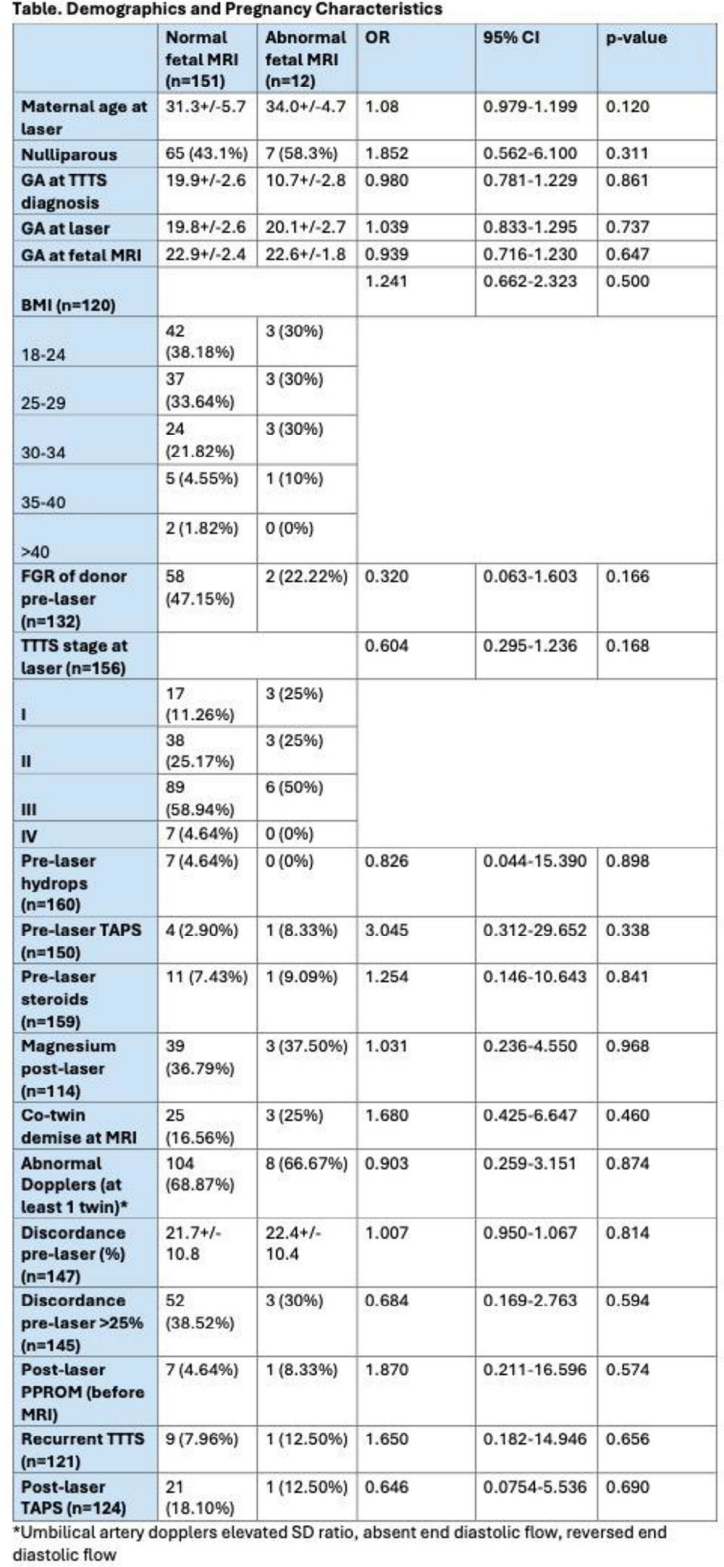

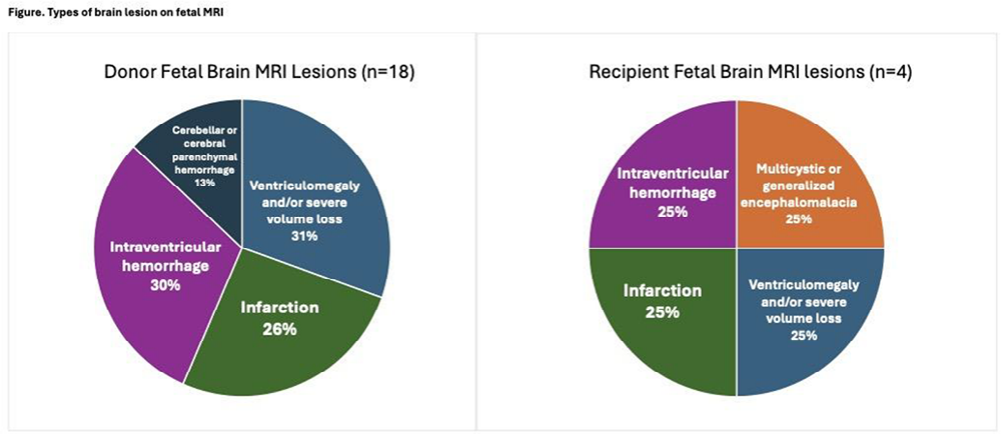

**3:30 PM – 5:00 PM**

## 577 | **An RCT to Assess Impact of Video Education on Decisional Conflict Regarding Aneuploidy Testing**

Margaret M. Thorsen^1^; Rose C. Mahoney^1^; Sayda MoranCordon^1^; Victoria Adewale^1^; Irving Angeles^1^; Paola Jiménez Muñoz^2^; Stephanie Nunez^1^; Cinthya Cruzcolon^1^; Carolyn Slack^1^; Melissa L. Russo^1^

^1^Women & Infants Hospital of Rhode Island / Warren Alpert Medical School of Brown University, Providence, RI; ^2^University of Illinois, Chicago, IL

**Objective**: Informed consent for prenatal aneuploidy testing is challenging due to complexity of counseling and lack of access to genetic counselors. Prior educational interventions have not been available in multiple languages, accessible for those with low educational attainment, or with patient voices incorporated into the design. This RCT sought to assess the impact of an end‐user‐designed video education series on patient decisional conflict scale (DCS) with respect to prenatal genetic testing. Secondary aims assessed testing uptake and risk perception of aneuploidy.

**Study Design**: English or Spanish speaking pregnant individuals were recruited at the dating ultrasound from Jan to July 2025. Participants completed a baseline genetics knowledge and demographics survey. Participants were randomized to video education (viewed prior to new OB visit) or standard care. A follow‐up survey was administered to all participants after their new OB appointment to assess DCS (primary outcome). Thirty‐six participants per group (total *n* = 72 participants) were required for 80% power to detect a decrease in DCS score from 30% to 23% (alpha = 0.05) based on prior literature. Genetic testing uptake was obtained via chart review. Wilcoxon rank sum test, Fisher's exact test, or Pearson's Chi‐squared test were applied as appropriate.

**Results**: A total of 72 participants were consented and enrolled (37 randomized to intervention). There were no baseline demographic or genetics knowledge differences between groups (Table 1). In the video intervention group, most participants (65%) watched the complete video series in‐person. There were no differences in mean DCS, testing uptake, or risk perception between groups (Table 2).

**Conclusion**: A patient‐designed video education series regarding aneuploidy testing options did not impact DCS regarding testing decisions. Compared to prior literature, participants in both groups were certain about their choices (low DCS scores). Uptake of cell free DNA was near universal. Further studies are needed to explore how options are being presented and patient comprehension of their results.

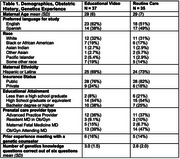

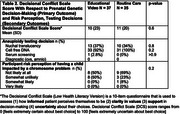

**3:30 PM – 5:00 PM**

## 578 | **Coagulation** Markers **Among Preeclamptic Pregnancies: A Systematic Review and Meta‐Analysis**

Maria Lemos^1^; Marcelo Garrido^2^; Laura Queiroz^3^; Hellen Pereira^4^

^1^Kurah Women's Health Institute, Montverde, FL; ^2^Oeste Paulista University, Presidente Prudente, Sao Paulo; ^3^Santa Casa de Misericórdia de Belo Horizonte, Belo Horizonte, Minas Gerais; ^4^University of Messina, Messina, Sicilia

**Objective**: To assess the alterations in coagulation markers among women with preeclampsia compared to normotensive pregnant women, in order to assess their potential utility in the clinical assessment and management of preeclampsia.

**Study Design**: A comprehensive literature search was conducted across PubMed, Embase and Cochrane Library to identify studies published up to July 2025. Studies were included comparing coagulation parameters, such as antithrombin III, fibrinogen, and D‐dimer, between preeclamptic and normotensive pregnant women. Pooled mean differences and 95% confidence intervals were calculated using a random‐effects model and heterogeneity through I^2^ statistics on RevMan.

**Results**: The primary finding was that Antithrombin III levels were significantly higher in normotensive pregnant women (MD =−10.35; 95% CI: −14.89 to −5.80; *p* < 0.00001; Figure A), reinforcing the notion that preeclampsia is associated with reduced anticoagulant capacity andendothelial dysfunction. We also assessed D‐dimer levels, which were significantly higher in preeclamptic pregnant women (MD = 393.21; 95% CI: 247.44 to 538.99; *p* < 0.00001; Figure B), suggesting a relevant marker of coagulation activation, though not specific to the condition, given their elevation in normotensive women as well. As for fibrinogen, no significant difference was observed between groups (MD = −0.13; 95% CI: −0.54 to 0.27; *p* = 0.52; Figure C), suggesting it may not be a reliable marker to distinguish preeclamptic and normotensive pregnancies.

**Conclusion**: Our findings reveal a distinct prothrombotic profile in preeclamptic pregnancies, marked by elevated D‐dimer and reduced antithrombin III levels compared to normotensive women, features reflecting endothelial dysfunction and increased coagulation activity. These markers enhance our understanding of preeclampsia's hemostatic changes and may serve as accessible, non‐invasive tools for early detection, monitoring, and risk stratification. As research progresses, incorporating coagulation biomarkers into predictive models and therapeutic strategies may enable more precise management of the condition.

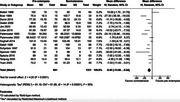

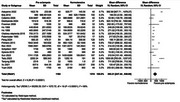

**3:30 PM – 5:00 PM**

## 579 | **Perinatal** Outcomes **Following Multifetal Pregnancy Reduction at a Quaternary Referral Center**

Marie J. Boller^1^; Miranda O'Keefe^1^; Lucy Ward^2^; Sally Segel^3^; John Buckmaster^4^; Catherine Caruso^1^; Anna Zylak^1^; Monica Rincon^5^; Adam Crosland^1^; Annessa S. Kernberg^1^; Neel K. Rana^2^; Leonardo Pereira^5^; Andrew H. Chon^2^

^1^Oregon Health and Science University, Portland, OR; ^2^Oregon Health & Science University, Portland, OR; ^3^Vancouver Clinic, Vancouver, WA; ^4^Legacy Health, Portland, OR; ^5^Oregon Health Sciences University, Portland, OR

**Objective**: The management of multiple gestations is complex and may involve multifetal pregnancy reduction (MFPR). Limited data exist regarding procedure‐related complications and pregnancy outcomes. Our objective was to describe perinatal outcomes of patients undergoing MFPR.

**Study Design**: Retrospective cohort study of patients with multiple gestations who underwent a MFPR procedure at a quaternary referral center between 2003 and 2024. All procedures were performed by Maternal‐Fetal Medicine physicians using potassium chloride injection. Patient characteristics were described, and post‐procedure outcomes were compared between early and late reduction. Descriptive statistics [n (%) and median (range)] were used to report findings.

**Results**: Sixty‐one patients were included: 30 dichorionic diamniotic twins and 31 triplets (25 trichorionic triamniotic and 6 dichorionic triamniotic). Of the triplets, 23 reduced to twins and 8 reduced to a singleton. The gestational age (GA) at MFPR was 12.7 weeks (11.4–21.0). A trainee was involved in 13 (21.0%); the procedure was technically successful in all cases. The placenta was traversed in 21.3% of cases. Incidental septostomy did not occur in any cases. Among the 30 twins, procedural complications and pregnancy outcomes were reported (Table 1). Post‐procedure pregnancy loss occurred in one case. Among those with delivery data available, the interval from MFPR to delivery was 24.6 weeks (16.4–29.0) (Figure 1). The GA at delivery following MFPR was 38.7 weeks (33.1–41.3) for twins reduced to singleton and 34.6 weeks (32.3–37.6) for triplets reduced to twins, and 40.1 weeks (36.4–41.4) for triplets reduced to singletons. Among those with neonatal follow‐up data, neonatal intensive care unit admission was 34.4% and overall neonatal survival was 100.0%.

**Conclusion**: At an experienced center, MFPR was technically achievable with favorable pregnancy outcomes and neonatal survival in both twin and triplet gestations.

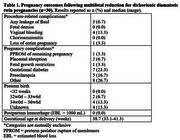

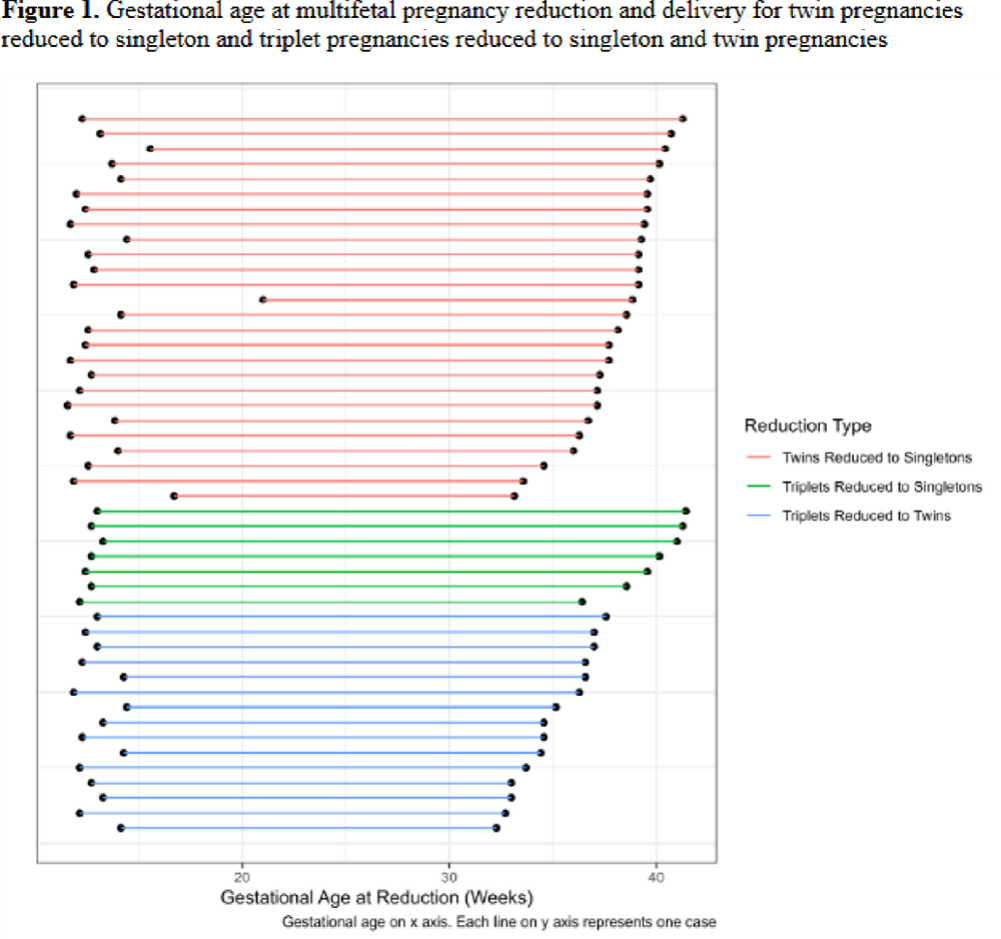

**3:30 PM – 5:00 PM**

## 580 | **The Association** Between **Obesity and Post‐Cesarean Morbidity**

Marissa J. Berry

On behalf of the Eunice Kennedy Shriver National Institute of Child Health and Human Development (NICHD) Maternal‐Fetal Medicine Units (MFMU) Network, Bethesda, MD

The Ohio State University, Columbus, OH

**Objective**: Severe maternal morbidity (SMM) and pregnancy‐related deaths are increasing, which may be affected in part by the rising prevalence of maternal obesity and rates of cesarean delivery (CD). Although each is an independent risk factor for maternal morbidity, obesity is also an established risk factor for CD. The objective of this analysis is to evaluate the relationship between body mass index (BMI) in pregnancy and SMM and mortality among individuals who underwent CD.

**Study Design**: Secondary analysis of a multicenter trial of tranexamic acid (TXA) in individuals who underwent CD. We included those with a documented BMI >18.5 kg/m^2^ prior to 20 weeks’ gestation. The primary exposure was categorical BMI: normal (18.5–24.9 kg/m^2^) [referent], overweight (25–29.9 kg/m^2^), class I/II obesity (30–39.9 kg/m^2^), class III obesity (≥40 kg/m^2^). The primary outcome was a composite of SMM and mortality including blood transfusion, acute kidney injury, hysterectomy, venous thromboembolism, ischemic stroke, myocardial infarction, seizure, sepsis, ICU admission, or death. We also evaluated individual adverse outcomes including postpartum infection, postpartum hemorrhage with interventions, and hospital readmission. Multivariable logistic regression was used to adjust for potential confounding variables.

**Results**: A total of 8864 individuals were included in this analysis. There was no difference in the primary outcome across BMI groups. There were significantly higher odds of the primary outcome excluding blood transfusion (aOR [95% CI]: 1.89 [1.05, 3.40]), postpartum infection (2.05 [1.36, 3.10]) and blood loss >1 liter (1.81 [1.37, 2.40]) with class III obesity, and of hospital readmission with class I/II (1.61 [1.16, 2.23]) and class III obesity (2.29 [1.59, 3.28]).

**Conclusion**: There was not an observed association between BMI and the primary outcome; however, class III obesity was associated with SMM/mortality when blood transfusion was excluded from the composite. The higher odds of important secondary outcomes in class III obesity highlight the risk for maternal morbidity in this cohort.

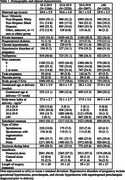

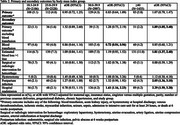

**3:30 PM – 5:00 PM**

## 581 | **Shorter Latency** After **Previable PPROM is Associated With Increased Risk of Subsequent Preterm Birth**

Marissa A. Hand^1^; Hannah Sinks^2^; Amrit Dhanushkoti^3^; Christine Chien^4^; Caroline Pennacchio^5^; Catherine Klammer^6^; Adriana Huelmo^3^; Meng Yao^7^; Carol Wang^3^; Lisa Gray^8^; Dzhamala Gilmandyar^2^; Ahmed Ahmed^7^; Amol Malshe^7^; Craig Pennell^3^

^1^Cleveland Clinic Foundation, Cleveland Clinic Foundation, Cleveland, OH; ^2^Hofstra Northwell School of Medicine, Hofstra Northwell School of Medicine, NY; ^3^University of Newcastle, University of Newcastle, New South Wales; ^4^Carle Foundation Hospital, Urbana, IL; ^5^University of Pittsburgh Medical Center, University of Pittsburgh Medical Center, PA; ^6^University of Pennsylvania, University of Pennsylvania, PA; ^7^Cleveland Clinic Foundation, Cleveland Clinic Foundation, OH; ^8^Carle Foundation Hospital, Carle Foundation Hospital, IL

**Objective**: Women who experience previable preterm premature rupture of membranes (PPROM) are at increased risk for recurrent preterm birth (PTB) in subsequent pregnancies. However, limited data exist regarding factors that may influence this recurrence risk. One potential factor is the time to onset of labor after membrane rupture during the index pregnancy, reflecting underlying inflammatory or structural abnormalities. Length of latency following previable PPROM may help guide counseling and risk stratification for future pregnancies.

**Study Design**: A multinational, retrospective cohort study of pregnancies complicated by previable PPROM (14w0d–23w6d) in the U.S. and Australia from 2010–2023, and subsequent pregnancy outcomes beyond 14 weeks were included. Patients were grouped by latency duration in the index pregnancy: labor onset <24 hours (idiopathic preterm birth) vs. >24 hours. Cases with IUFD preceding PPROM or artificial rupture were excluded. Outcomes in the index pregnancy were stratified as early mid‐trimester loss (MTL) (14w0d–19w6d), late MTL (20w0d–23w6d), and PTB (24w0d–36w6d).

**Results**: Of 111 patients with previable PPROM, 35 spontaneously labored within 24 hours and 76 after 24 hours. No significant differences were observed in aspirin or progesterone use, or hypertensive disorders in the index pregnancy. Patients who experienced idiopathic PTB <24 weeks in the index pregnancy had an increased risk of MTL (11.4% vs. 7.9%, *p* = 0.073) and significantly higher rates of PTB (34.3% vs 17.1%, *p* < 0.001) in the subsequent pregnancy compared to those with ruptured for greater than 24 hours (Table 1).

**Conclusion**: Among patients with previable PPROM, those who labored within 24 hours of membrane rupture were at increased risk of mid‐trimester loss and significantly more likely to experience preterm birth in a subsequent pregnancy. These findings suggest that latency duration may serve as a clinically meaningful marker of underlying biological risk and inform surveillance strategies in future pregnancies.

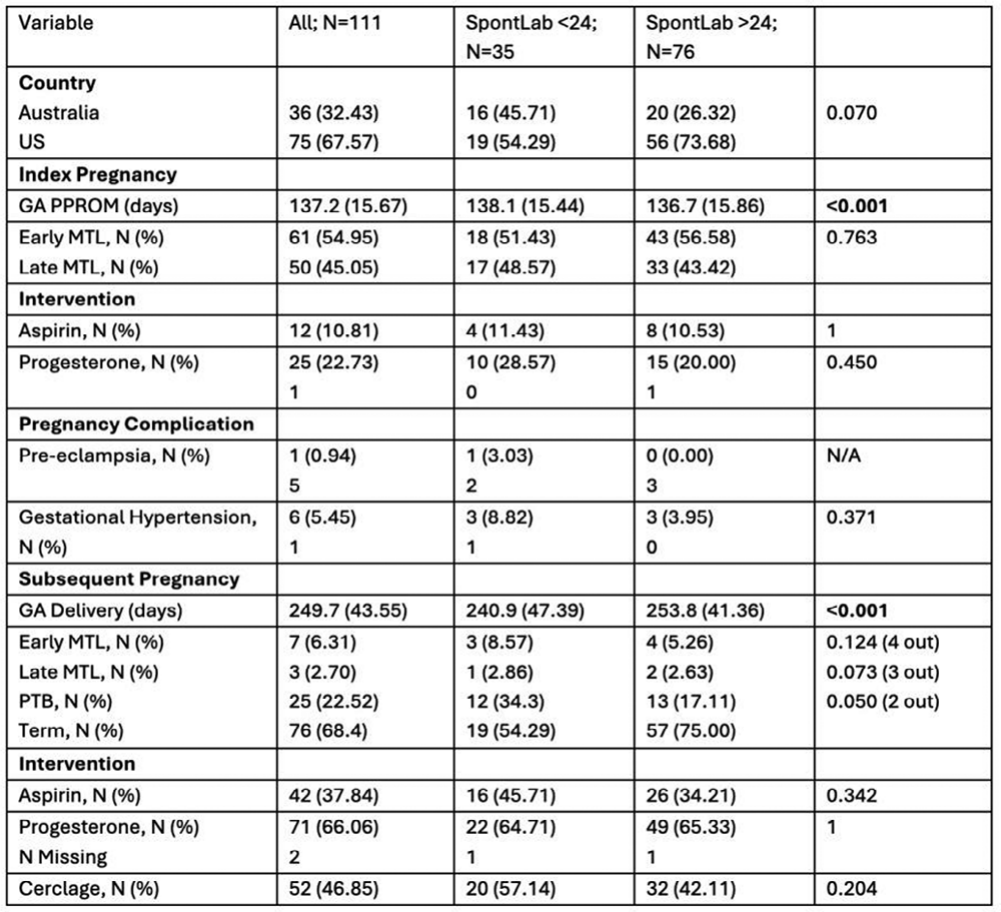

**3:30 PM – 5:00 PM**

## 582 | **Association** Between **Social Determinants of Health and Acute Care Utilization in Pregnancy**

Marissa Platner^1^; Priyanka Mehta^2^; Kaitlyn K. Stanhope^3^

^1^Emory University School of Medicine, Atlanta, GA; ^2^Emory University School of Medicine, Emory University School of Medicine, GA; ^3^Rollins School of Public Health, Rollins School of Public Health, GA

**Objective**: In non‐pregnant populations, social needs, including housing instability, food insecurity, and neighborhood disadvantage, are associated with increased acute care utilization. Our objective is to determine if patients reporting social needs have an increased number of triage visits during pregnancy and the immediate postpartum period compared to those not reporting social needs.

**Study Design**: We conducted a prospective cohort study from October 2019 to July 2021 of 1608 pregnant patients who attended prenatal care at our institution. Social needs within the prior 12 months were evaluated using the CMS/CMMI Accountable Health Communities‐ Health Related Social Needs Screening Tool. Five core domains were evaluated including housing, utilities, transportation, food and interpersonal violence. We examined the association between each domain of social need (present/absent) and the number of triage visits. We used Poisson regression to calculate unadjusted and adjusted risk ratios (aRRs), adjusting for race/ethnicity, parity, prenatal care utilization, and obstetric comorbidity score.

**Results**: Of the 1608 patients who completed the social needs screening 81.2% were non‐Hispanic Black and 87.4% had public insurance. 44.8% (720) of all patients screened reported having any social needs. The most common reported needs were food (19.2%), housing (16.5%), and transportation (14.2%). Patients who reported housing, utilities, or interpersonal violence social needs each had a higher mean number of triage visits than those without those social needs. (Table 1). After adjusting for confounders, the patients who reported housing (aRR 1.20), utilities (aRR 1.25), or interpersonal violence (aRR 1.37) social needs had an increased likelihood of high triage utilization. (Table 2)

**Conclusion**: Patients with housing, utility, and interpersonal violence needs had higher mean number of triage visits. After controlling for confounders, these patients also had significantly higher risk of increased triage utilization.

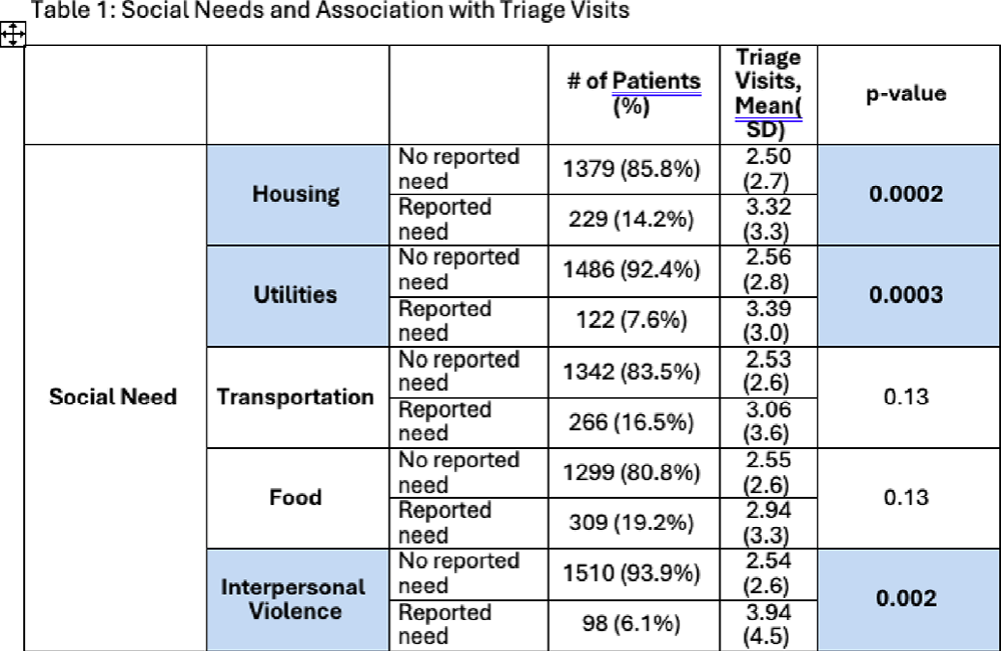

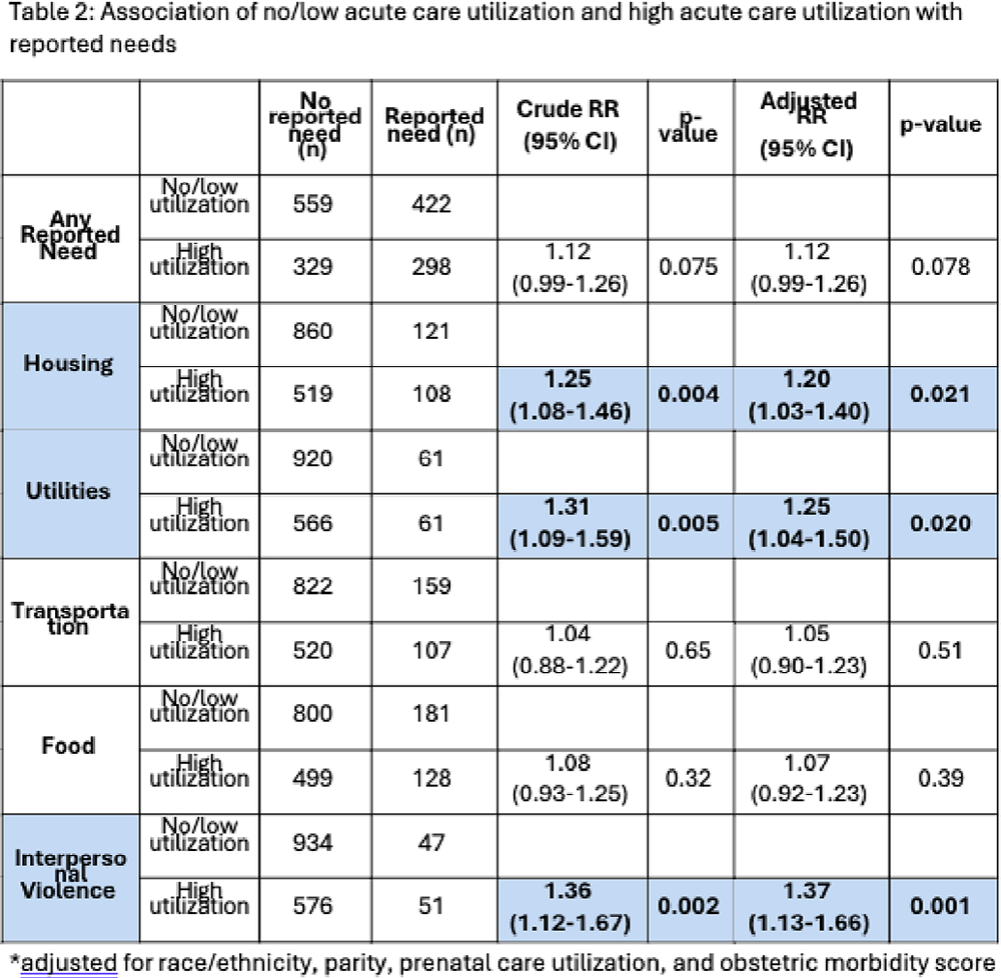

**3:30 PM – 5:00 PM**

## 583 | **Antenatal Fetal** Surveillance **Use and Stillbirth Risk in the US**

Haley K. Sullivan^1^; Anna Sinaiko^2^; Joanne C. Armstrong^3^; Kathe P. Fox^4^; Jessica L. Cohen^5^; Mark A. Clapp^6^

^1^Harvard Interfaculty Initiative in Health Policy, Cambridge, MA; ^2^Harvard T. H. Chan School of Public Health, Boston, MA; ^3^CVS Health, Wellesley, MA; ^4^Health Care Cost Institute, Washington, DC; ^5^Harvard. T.H. Chan School of Public Health, Boston, MA; ^6^Department of Obstetrics and Gynecology, Mass General Brigham, Massachusetts General Hospital, MA

**Objective**: To analyze antenatal fetal surveillance (AFS) among live and stillbirths in a US cohort and describe variation in AFS use for pregnancies with and without indications for testing.

**Study Design**: This study analyzed births from the Health Care Cost Institute (HCCI) commercial health insurance claims. AFS tests were captured with Current Procedural Terminology (CPT) codes for nonstress tests, biophysical profiles, and Doppler artery velocimetry. We analyzed AFS use in pregnancies that delivered at 32 weeks of gestation or later by AFS‐indicated or AFS‐not‐indicated pregnancies and by birth outcome (stillbirth or live birth). “AFS‐indicated” was defined by ICD‐10 codes corresponding to conditions included in the ACOG guidelines on outpatient fetal surveillance.

**Results**: We identified 2,792,701 live and stillbirths among 2,508,195 people. Pregnancies identified with indications for AFS had a significantly higher stillbirth rate than those without indications (7.94 vs. 4.88 stillbirths per 1000 births; *p* < 0.01). 62% of all pregnancies had ≥1 indication for AFS, and 59% of the sample had ≥1 AFS procedure. The distribution of indications for AFS is shown in Figure 1. Among pregnancies that were delivered ≥32 weeks, AFS‐indicated pregnancies were substantially more likely to have AFS than AFS‐not‐indicated pregnancies (65.0 vs 46.5%, *p* < 0.001). Among AFS‐indicated pregnancies, those that ended in stillbirth were less likely to have had AFS than those that ended in live birth (65.5 vs 72.6%, *p* < 0.001; Figure 2). A similar pattern was observed among AFS‐not‐indicated pregnancies (31.9 vs 38.0%, *p* < 0.001). 27.8% of AFS‐indicated pregnancies did not receive any AFS.

**Conclusion**: AFS use was common and associated with lower stillbirth rates in a large US cohort. Nearly 3 in 10 pregnancies with indications did not receive AFS. Further investigation is needed into barriers to receiving guideline‐based care and to inform the design of future efforts improve care quality and reduce stillbirth rates in the US.

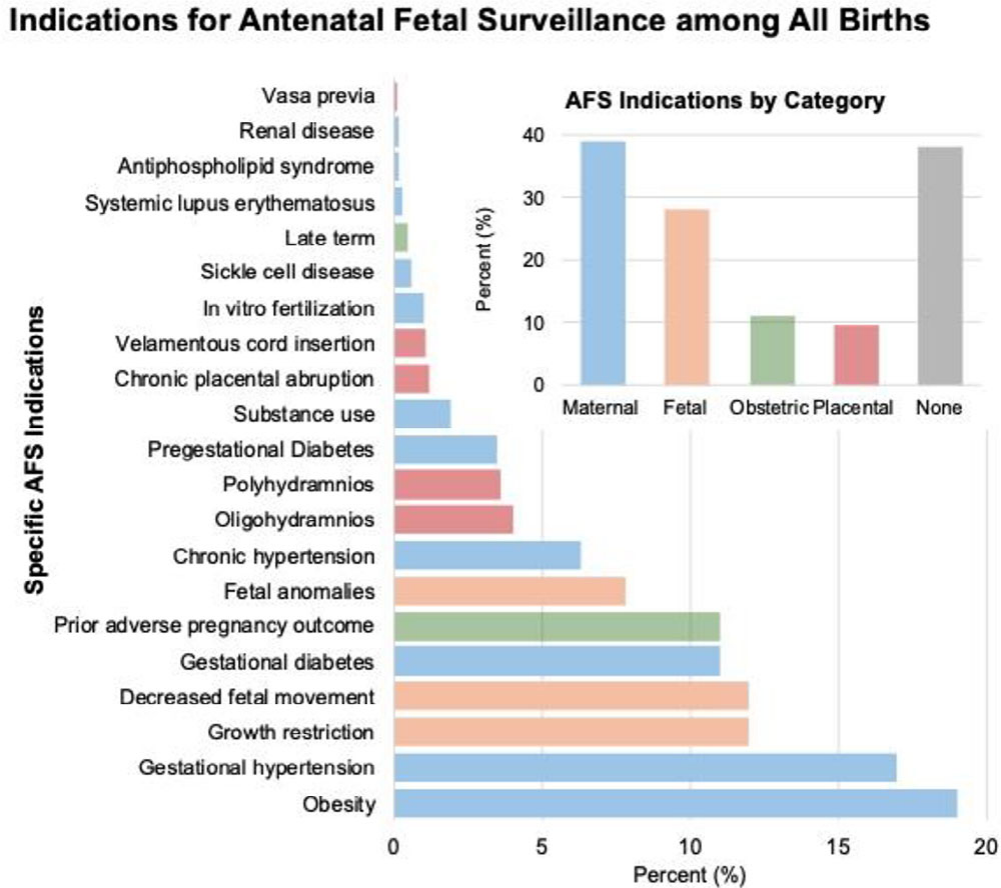

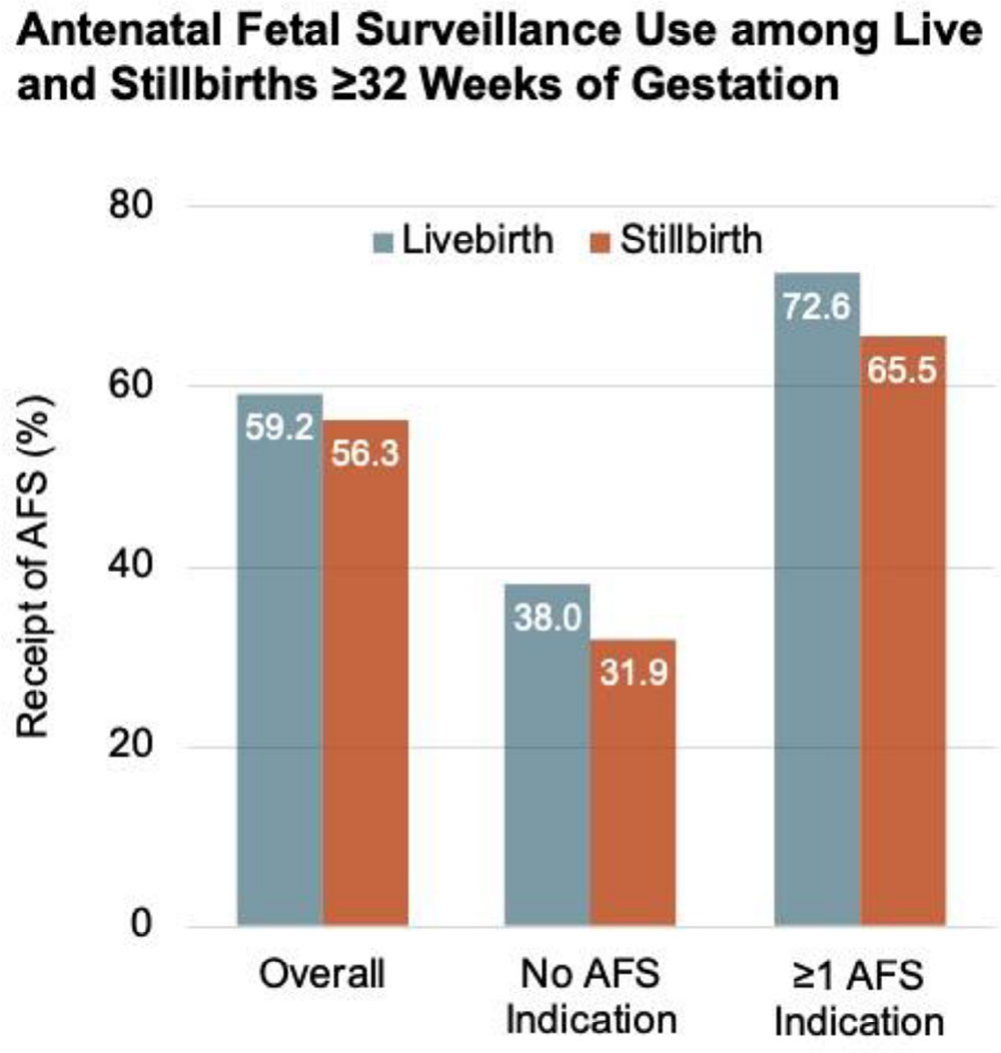

**3:30 PM – 5:00 PM**

## 584 | **Beyond** Comorbidities**: Integrating Real‐Time Clinical Data to Improve Prediction of Severe Maternal Morbidity**

Matthew J. Blitz; Frank I. Jackson; Dimitre Stefanov; Alejandro D. Alvarez; Fernando Suarez; Adriann Combs; Dawnette Lewis; Michael Nimaroff; Burton Rochelson

Northwell, New Hyde Park, NY

**Objective**: To compare the predictive performance of three models for severe maternal morbidity (SMM): (1) the Obstetric Comorbidity Index (OB‐CMI), (2) the California Maternal Quality Care Collaborative (CMQCC) score, and (3) a novel model that integrates OB‐CMI and CMQCC elements with real‐time clinical data. Prediction of SMM without transfusion (SMM‐NT) was assessed as a secondary outcome.

**Study Design**: We conducted a retrospective cohort study including 160,515 deliveries from 132,189 patients across a large New York health system (2019–2024). SMM was defined using CDC criteria. Patients were randomly assigned to training (60%), validation (20%), and test (20%) sets, with all deliveries from a given patient grouped together. Logistic regression models were developed using backward elimination. The novel model incorporated comorbidities, vital signs (heart rate, mean arterial pressure, temperature), laboratory values (hematocrit, white blood cell count, platelets), non‐linear terms, and missingness indicators. Model performance was assessed in the test set using area under the receiver operating characteristic curve (AUC‐ROC), Brier score, and the Spiegelhalter test for calibration.

**Results**: Among 31,943 test‐set deliveries, 6966 (4.3%) met SMM criteria and 1373 (0.9%) met SMM‐NT criteria. The novel model significantly outperformed both OB‐CMI (AUC 0.6726) and CMQCC (AUC 0.7244), achieving an AUC of 0.7630 (*p* < 0.0001 vs. both) and better calibration (Brier 0.039, Spiegelhalter *p* = 0.53). For SMM‐NT, discrimination and calibration were even stronger (AUC 0.7868, Brier 0.0123). Model performance was consistent across racial and insurance subgroups.

**Conclusion**: A novel model incorporating real‐time admission vitals and labs improved prediction of SMM and SMM‐NT compared to OB‐CMI and CMQCC alone. Dynamic data may enhance early risk identification and support targeted maternal care interventions.

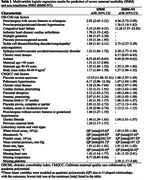

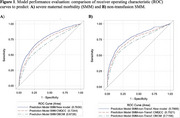

**3:30 PM – 5:00 PM**

## 585 | **Obstetrics** Comorbidities **Index Predicts Severe Maternal Morbidity in Patients With Placenta Accreta Spectrum**

Matthew H. Mossayebi^1^; Logan C. Mauney^2^; Hope Y. Yu^3^; Daniela A. Carusi^1^; Mark A. Clapp^4^; Christina M. Duzyj^1^

^1^Department of Obstetrics and Gynecology, MassGeneral Brigham; Division of Maternal‐Fetal Medicine; Harvard Medical School, Boston, MA; ^2^Tufts Medical Center, Boston, MA; ^3^Department of Obstetrics and Gynecology, MassGeneral Brigham; Division of Maternal‐Fetal Medicine; Harvard Medical School, Bostton, MA; ^4^Department of Obstetrics and Gynecology, Mass General Brigham, Massachusetts General Hospital, MA

**Objective**: The obstetric comorbidity index (OB‐CMI) is a clinical risk‐assessment tool that prospectively identifies patients at risk of severe maternal morbidity (SMM); however, its additive utility in patients with pre‐existing risk due to placenta accreta spectrum (PAS) remains unknown. We assessed the association between OB‐CMI and SMM in deliveries with PAS.

**Study Design**: We conducted a retrospective cohort study of patients with histologically confirmed PAS delivered via cesarean hysterectomy at two major academic medical centers between 2019 and 2024. The primary outcome was number of SMM events based on ACOG/SMFM consensus definition, excluding hysterectomy. We additionally stratified analysis by cesarean urgency and histopathologic PAS grade. OB‐CMI was calculated by the patient's primary nurse. Given the increased risk of SMM in patients with scores >9 in prior studies, we similarly stratified our results to reflect this risk threshold. Chi‐square testing was used to assess the association between OB‐CMI and SMM.

**Results**: A total of 72 cases were identified, of whom 61 (85%) experienced severe maternal morbidity (Table 1). OB‐CMI ranged from 1 to 14 in the study cohort. Those with OB‐CMI scores >9 had higher number of SMM events (median SMM 1 vs 1, interquartile range (IQR) 0–1 vs 1–2, *p* = 0.057), when compared to cases with OB‐CMI <9 (Table 2). A higher number of SMM events continued to be observed in the OB‐CMI score >9 group after removing blood transfusions (median SMM 0 vs 0, IQR 0–0 vs 0–1, *p* = 0.002). With scheduled hysterectomy, OB‐CMI score <9 was associated with less non‐transfusion SMM (median 0 vs 0, IQR 0–0 vs 0.5, *p* = 0.013) compared to those with OB‐CMI >9. Scheduled hysterectomy was not associated with a higher OB‐CMI (7 vs 6, *p* = 0.17). Histopathologic stage of PAS was not associated with occurrence of SMM (*p* = 0.39).

**Conclusion**: Identification of high‐risk pre‐existing conditions as delineated by obstetrics comorbidities index (OB‐CMI) predicts severe maternal morbidity events in patients with placenta accreta spectrum when removing anticipated morbidity of blood transfusions.

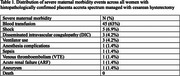

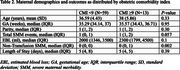

**3:30 PM – 5:00 PM**

## 586 | **Ampicillin–**Gentamicin **Versus Ampicillin Alone for Term PROM in GBS‐Negative Patients: A Randomized Controlled Trial**

Raneen Abu Shqara^1^; Daniel Glikman^2^; Gabriela Goldinfeld^3^; Dunia Hassan^4^; Amal Orabi^4^; Nadir Ganem^5^; Lior Lowenstein^5^; Maya Frank Wolf^5^

^1^Galilee Medical Center, Nahariya, HaZafon; ^2^Azrieli Faculty of Medicine, Bar Ilan University, Safed, Israel, Poria, HaZafon; ^3^assuta ashdod, ashdod, HaDarom; ^4^Galilee Medical Center, Nahariyya, HaZafon; ^5^Galilee Medical Center, Naharyia, HaZafon

**Objective**: To compare maternal and neonatal outcomes between two prophylactic antibiotic regimens—ampicillin plus gentamicin versus ampicillin alone—in patients with term premature rupture of membranes (PROM) and confirmed negative group B Streptococcus (GBS) colonization.

**Study Design**: This single‐center, randomized controlled trial was conducted at a tertiary university‐affiliated hospital between November 2022 and July 2025. Eligible participants were women with singleton term pregnancies, PROM >18 hours, and negative GBS colonization. Patients were randomized in a 1:1 ratio to receive either intravenous ampicillin (2 g every 6 h) plus gentamicin (5 mg/kg every 24 h) or ampicillin alone, starting 18 hours post‐PROM. The primary outcome was the incidence of clinical chorioamnionitis. Secondary outcomes included intrapartum fever, postpartum maternal infections, postpartum stay ≥5 days, and neonatal morbidity. Chorioamniotic cultures were obtained postpartum. Analyses were performed on an intention‐to‐treat basis.

**Results**: A total of 207 women were randomized (103 to ampicillin–gentamicin; 104 to ampicillin alone). Clinical chorioamnionitis occurred less frequently in the combination group (1.9% vs. 10.6%; *p* = 0.019; number needed to treat = 11.5, 95% CI: 7–45). Postpartum infectious morbidity was also reduced in the ampicillin–gentamicin group (1.9% vs. 9.6%; *p* = 0.033), as was the rate of postpartum hospitalization ≥5 days (3.9% vs. 13.5%; *p* = 0.024) (Figure 1). Neonatal outcomes did not differ significantly between groups. Positive chorioamniotic cultures were less frequent in the ampicillin–gentamicin group (20.9% vs. 36.7%; *p* = 0.029), including a lower prevalence of Enterobacteriaceae spp. (12.1% vs. 25.6%; *p* = 0.033) (Figure 2).

**Conclusion**: Among GBS‐negative patients with prolonged term PROM, prophylactic administration of ampicillin plus gentamicin significantly reduced rates of clinical chorioamnionitis and postpartum maternal infectious morbidity compared to ampicillin monotherapy. Broader Gram‐negative coverage may improve maternal outcomes and warrants further evaluation

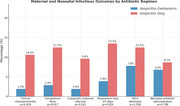

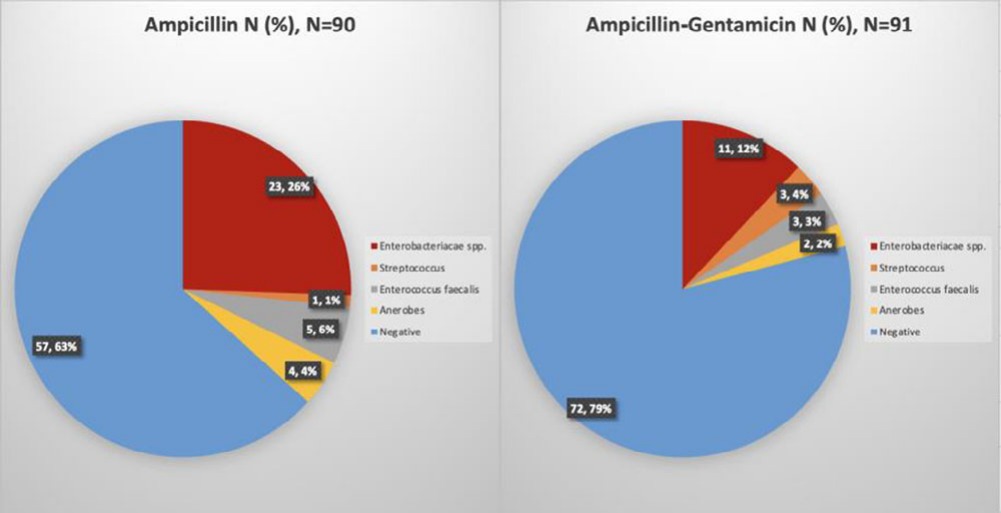

**3:30 PM – 5:00 PM**

## 587 | **The Higher** Preterm **Birth Risk After Conization is Unaltered by Conization‐to‐Pregnancy Interval or Interventions**

Maya Oberman^1^; Orly Weinstein^2^; Oren Barak^3^; Racheli Magnezi^4^; Inbal Avrahami^1^; Amir Kandel^5^; Moran Frid^1^; Omri Mizrachi^1^; Roni Levy^1^; Edi Vaisbuch^1^

^1^Kaplan Medical Center, Kaplan Medical Center, HaMerkaz; ^2^Clalit Health Services, Clalit Health Services, Tel Aviv; ^3^Kaplan Medical Center, Rehovot, HaMerkaz; ^4^Bar Ilan University, Bar Ilan University, HaMerkaz; ^5^Kaplan Medical Center, Kaplan Medical Center, HaZafon

**Objective**: This study aimed to assess the effect of cervical conization on the incidence of preterm birth and to determine whether conization‐to‐pregnancy interval or preventive interventions influence this risk.

**Study Design**: This retrospective cohort study included 7080 primiparous women. The study group included 600 (8.5%) women who had a cervical conization before their first delivery, and the rest served as the control group (6,480 women). Baseline characteristics, pregnancy management, and outcomes were compared between the groups. The study group was further analyzed for the rates of preterm birth (<37, <34, and <32weeks) stratified by the conization‐to‐pregnancy interval (<6, 6–11, and ≥12 months) and preventive interventions, including cervical cerclage (early <16 vs late ≥16 weeks) and progesterone administration

**Results**: During pregnancy, the study group demonstrated a higher prevalence of short cervix (15.5% vs 4.0%, *p* < 0.001), with similar rates of pregnancy complications such as hypertensive disorders (7.0% vs 6.7%, *p* = 0.7) and gestational diabetes (4.5% vs 4.3%, *p* = 0.8) to the control group. The study group had a higher risk of preterm birth <37 weeks (16.2% vs 10.9%; *p* < 0.0001), <34 weeks (6.6% vs 3.4%; *p* < 0.001), and <32 weeks (4.2% vs 1.1%; *p* < 0.001). However, within the conization group, preterm birth rate did not differ by conization‐to‐pregnancy interval, timing of cerclage, or progesterone initiation (Table 1).

**Conclusion**: Prior cervical conization in primiparous women increases the risk of preterm birth, including a two‐ to four‐fold risk at <34 and <32 weeks. However, preventive interventions or a longer conization‐to‐pregnancy interval failed to mitigate this risk. Larger cohorts are warranted to validate this finding further.

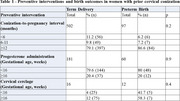

**3:30 PM – 5:00 PM**

## 588 | **Use of Low Dose** Aspirin **and Timing of Preeclampsia With Severe Features Diagnosis and Delivery**

Maya Patel^1^; Madison Calvert^1^; Hannaneh Mirmozaffari^1^; Jaye Boissiere^2^; Sally Kuehn^2^; Brooke Schroeder^2^; Matt Fuller^2^; Marie‐Louise Meng^2^; Johanna Quist‐Nelson^1^; Kim Boggess^3^

^1^University of North Carolina, Chapel Hill, Chapel Hill, NC; ^2^Duke University School of Medicine, Durham, NC; ^3^University of North Carolina at Chapel Hill, Chapel Hill, NC

**Objective**: Low dose aspirin (LDASA) is used to mitigate odds of developing preeclampsia, and some data suggest that it also delays onset of diagnosis. Our objective was to evaluate the association between LDASA and timing of preeclampsia with severe features (PEC‐SF) diagnosis and medically‐indicated preterm delivery.

**Study Design**: This retrospective cohort study included patients diagnosed with antepartum PEC‐SF as defined by ACOG who delivered at two academic medical centers over two years. The exposure of interest was LDASA use during pregnancy. The primary outcome was gestational age <37 weeks at time diagnosis of PEC‐SF. The secondary outcomes were delivery at <37 weeks and <34 weeks, and <34 weeks at time of PEC‐SF diagnosis. Multivariable logistic regression models were used to estimate adjusted odds ratios (ORs) and 95% confidence intervals (CIs) for preterm birth. Kaplan‐Meier curves were generated to examine timing of diagnosis and timing of delivery for those reporting LDASA and those not reporting LDASA.

**Results**: Of 774 patients with PEC‐SF, 186 (24%) reported LDASA and comprise the analysis cohort. Of the 186, 124 (67%) were diagnosed with preterm PEC‐SF at <37 weeks, and 58 (31%) at <34 weeks. Compared to non‐LDASA users, LDASA use was not associated with PEC‐SF at <37 weeks or <34 weeks (Table 1). The Kaplan‐Meier curves comparing the timing of PEC‐SF diagnosis and timing of delivery between LDASA users and non‐users demonstrate no meaningful difference between the two groups (Figure 1).

**Conclusion**: In this diverse population, the modest, non‐significant increase in odds of preterm diagnosis and delivery <37 weeks among ASA users reflects confounding by indication, as patients prescribed LDASA have higher baseline risk of PEC‐SF.

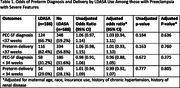

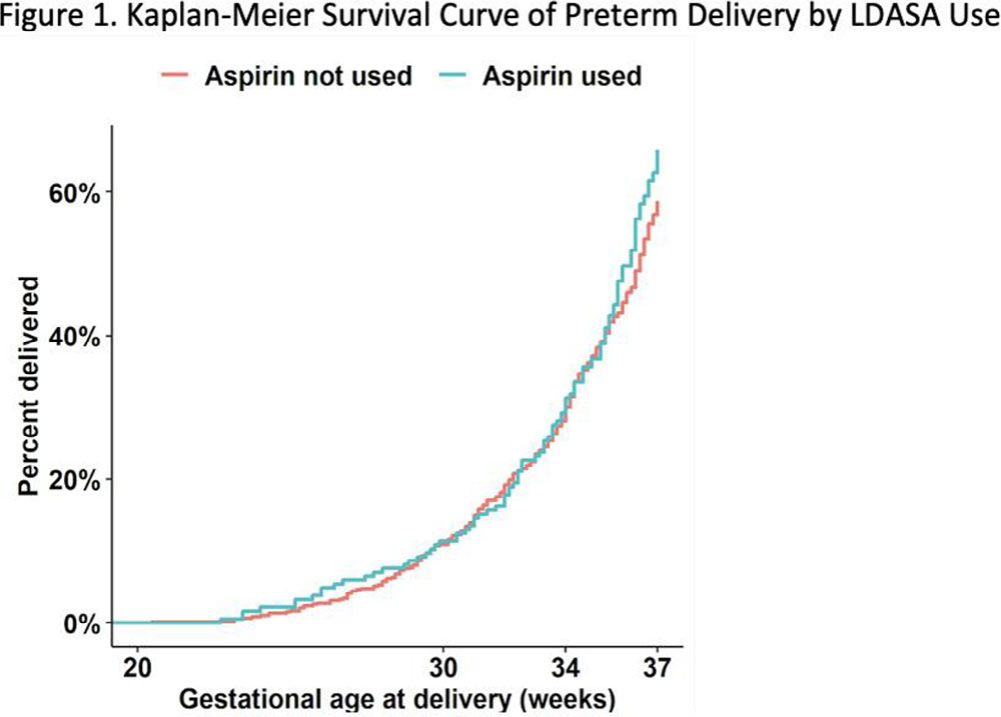

**3:30 PM – 5:00 PM**

## 589 | **The** Development **of Placenta Accreta Spectrum‐Sonographic Assessment for Forecasted Estimated Blood Loss (PAS‐SAFE) Scoring System**

Maya A. Sahtout^1^; Chitra Gotluru^2^; Amberly Diep^3^; Debra Swanson^3^; Herman Hedriana^3^; Lihong Mo^3^

^1^UC Davis School of Medicine, Sacramento, CA; ^2^University of Miami/ Jackson Health System, Miami, FL; ^3^Department of Obstetrics and Gynecology, UC Davis, Sacramento, CA

**Objective**: While the sonographic diagnostic criteria for Placenta Accreta Spectrum (PAS) were well established, they do not reliably predict the degree of intraoperative hemorrhage. The objective of this study is to develop and validate a PAS‐Sonographic Assessment for Forecasted Estimated Blood Loss (PAS‐SAFE) scoring system, thereby enhancing preoperative planning.

**Study Design**: A retrospective cohort of 113 patients confirmed PAS at a single tertiary academic medical center was identified (2015 to 2024). Patients were excluded if data were incomplete, EBL values were outliers, or if pregnancy was terminated. The final cohort included 30 patients, with 17 classified as low EBL (≤ 1000 mL) and 13 as high EBL (>3000 mL). Second‐ or third‐trimester ultrasounds were independently reviewed by four blinded examiners. Each examiner assigned a PAS‐SAFE score. Inter‐rater reliability was assessed using intraclass correlation coefficients. Linear regression assessed the association between PAS‐SAFE score and EBL, with R^2^ and *p*‐value reported.

**Results**: The PAS‐SAFE scoring system consisted of six components (Figure 1) including lacunae, placenta location, previa status, vascularity, bladder involvement, and blood flow. Inter‐rater reliability was 16.67% however the trend was similar between examiners (Figure 1). There was a significant correlation between the PAS‐SAFE score and the EBL at cesarean hysterectomy (Figure 2). Confounding variables including hypertension, gestational age at delivery, antenatal vaginal bleeding episodes, number of prior cesarean sections, and urgency of delivery were found to be similar between groups.

**Conclusion**: The PAS‐SAFE scoring system shows promise as a predictive tool for estimating blood loss at the time of cesarean hysterectomy in patients with PAS. Limitations of this study include the small sample size of both patients and examiners, which may impact generalizability. Future directions include prospective validation of the scoring system in larger, multi‐center cohorts to assess its clinical utility and reproducibility across diverse practice settings.

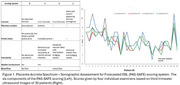

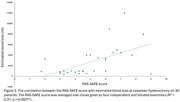

**3:30 PM – 5:00 PM**

## 590 | **Patient, Clinician and Administrator Perspectives on a Home Remote Blood Pressure Monitoring Program**

Mayra Alejandra Shafique^1^; Ashley Williams^2^; Ashley M. Hesson^2^; Elizabeth S. Langen^2^; Joanne M. Bailey^3^; Molly J. Stout^2^; Jourdan E. Triebwasser^2^; Natasha R. Kumar^4^

^1^University of Michigan, OBGYN Department, Ann Arbor, MI; ^2^University of Michigan, Ann Arbor, MI; ^3^University of Michigan, University of Michigan, MI; ^4^University of Michigan, Department of OBGYN, Ann arbor, MI

**Objective**: Remote home blood pressure (BP) monitoring improves postpartum BP acquisition, mitigates racial disparities in follow‐up, reduces urgent postpartum care for individuals with hypertensive disorders of pregnancy (HDP). Successful program implementation relies on engagement by patients, clinicians and administrators, but there is a paucity of data on stakeholder perspectives. We assessed the impact of a 10‐day text message support program including escalation of care via phone calls and a telemedicine visit upon completion.

**Study Design**: Semi‐structured key informant interviews of individuals with HDP (*n* = 10), OB clinicians (*n* = 12) and obstetric hospital administrators (*n* = 4) were performed to assess barriers and facilitators to postpartum transitions to primary care after engagement with remote BP monitoring using the socioecological model at a single academic center. Interviews were coded using grounded theory until thematic saturation was achieved.

**Results**: Patients attributed high levels of self‐efficacy, i.e. sense of control over their health, due to the program's behavioral nudges (Table 1). They experienced increased connection with providers with ongoing and rapid engagement by the clinical team. Clinicians and patients felt that this program improved organizational efficiency with clinical decisions (Table 2). Clinicians and administrators appreciated remote care delivery, which increased appointment availability for other issues (Table 2). However, patients desired opportunities for more frequent touchpoints with clinicians regarding other postpartum issues. Patients’ understanding of long‐term CVD risks beyond program completion was limited. Some patients desired more in‐person care.

**Conclusion**: Home blood pressure monitoring is positively perceived by both providers and patients. However, there are unmet opportunities for improved patient education about long‐term CVD risks outside the immediate postpartum period suggesting a critical gap in immediate postpartum provider to longer‐term primary care provider transitions.

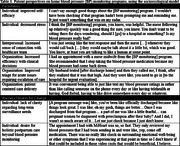

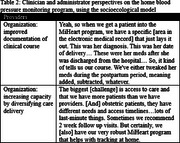

**3:30 PM – 5:00 PM**

## 591 | **Support for** Universal **Third Trimester Ultrasound From Risk Factor‐Based Analysis**

Megan E. Graeff^1^; Suneet P. Chauhan^2^; Aaron W. Roberts^1^

^1^Department of Obstetrics, Gynecology and Reproductive Sciences, McGovern Medical School at UTHealth Houston, Houston, TX; ^2^Delaware Center for Maternal‐Fetal Medicine of Christiana Care, Newark, DE

**Objective**: Abnormalities of amniotic fluid or growth are linked with adverse outcomes; however, in low‐risk pregnancies the majority are unidentified prior to delivery. This study aimed to identify maternal risk factors associated with oligohydramnios, polyhydramnios, fetal growth restriction (FGR), and large for gestational age (LGA) in low‐risk pregnancies without any other indications for third trimester ultrasound.

**Study Design**: This secondary analysis includes 661 low‐risk pregnancies in the intervention arm from a previous study (PMID: 38583714) where all patients had fetal growth and amniotic fluid assessed by ultrasound at 36 weeks. Maternal factors (race, age, height, pre‐pregnancy BMI, nulliparity, and insurance status) were analyzed in relation to the incidence of oligohydramnios, polyhydramnios, FGR, and LGA.

**Results**: Of the patients analyzed, 147/661 (22.2%) had a composite of the four ultrasound findings. No single risk factor was predictive of this abnormal ultrasound composite (Table). When the composite components were analyzed separately, maternal age ≤19 years old (OR 5.49 95% CI: 1.72–17.56, *p* = 0.006) and nulliparity were associated predictors of oligohydramnios (OR 2.35 95% CI: 1.09–5.05, *p* = 0.025). Finally, patients who self‐identified as black had lower rates of LGA at 36 weeks compared to white patients (OR 0.23 95% CI: 0.09–0.58, *p* < 0.001).

**Conclusion**: At 36 weeks ultrasound exam composite of fetal growth or amniotic fluid abnormality was identified in 1 in 5 low‐risk pregnancies, with no significant predictive factors apparent prior to universal screening. This supports implementing a universal 36‐week ultrasound screening protocol for low‐risk pregnancies. The association of oligohydramnios and LGA with maternal age, parity, and race warrants further study. When routinely offered to all patients, third trimester ultrasound facilitates the identification of abnormalities that would otherwise go undetected with current recommended fundal‐height protocols.

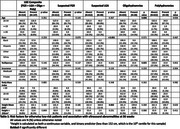

**3:30 PM – 5:00 PM**

## 592 | **What to Expect** of **Expectant Management in Preterm Preeclampsia with Severe Features: Impact of Co‐morbidities**

Megan C. Shepherd^1^; Wissam Akkary^1^; Yossi Bart^1^; Joe Haydamous^2^; Zakaria Doughan^3^; Sean C. Blackwell^1^; Baha M. Sibai^1^; Ahmed Zaki Moustafa^4^

^1^Division of Maternal‐Fetal Medicine, Department of Obstetrics, Gynecology and Reproductive Sciences, McGovern Medical School at UTHealth Houston, Houston, TX; ^2^Department of Obstetrics, Gynecology and Reproductive Sciences, McGovern Medical School at UTHealth Houston, Department of Obstetrics and Gynecology, McGovern Medical School at UT Health, Houston, TX; ^3^Department of Obstetrics and Gynecology, McGovern Medical School at UT Health, Houston, Houston, TX; ^4^Division of Maternal‐Fetal Medicine, Department of Obstetrics, Gynecology and Reproductive Sciences, McGovern Medical School at UTHealth Houston, University of Texas ‐ Houston, TX

**Objective**: Expectant management of preeclampsia with severe features (PSF) before 34 weeks in stable patients is primarily considered for fetal benefit. However, current guidance does not address the presence of additional medical comorbidities. As a result, the safety and impact of expectant management in this population remains unclear. We aimed to evaluate outcomes of expectant management in PSF, comparing patients with and without comorbidities.

**Study Design**: This retrospective cohort study included singleton pregnancies with PSF at <32 weeks at a single tertiary center between 2016–2025. Patients were excluded if enrollment gestational age (GA) was <23 weeks, or if they were delivered within 24 hours of diagnosis. Group 1 included patients without comorbidities, and group 2 included patients with ≥1 comorbidity (chronic hypertension, BMI >40 Kg/m^2^, pregestational diabetes, cardiac disease, renal disease, or autoimmune disease). The primary outcome was median latency (time interval from diagnosis to delivery) in days. Secondary outcomes included a composite of severe maternal morbidity and stillbirth.

**Results**: A total of 304 patients met inclusion criteria, 119 without comorbidities (group 1) and 185 with at least 1 comorbidity (group 2). Patients without comorbidities were more likely to be younger, primiparous, and to have fetal growth restriction (FGR). After adjusting for age and parity, there was no difference in median latency (4 days in group 1 vs 5 days in group 2, *p* = 0.59), or composite adverse outcomes (Table 1). Patients in group 1 were more likely to achieve 34 weeks (13% vs 5.4%, *p* = 0.020) and to be delivered for fetal indications (36% vs 26%, *p* = 0.043) as compared to group 2.

**Conclusion**: Maternal comorbidities did not impact latency or adverse maternal outcomes. However, patients without comorbidities were more likely to reach 34 weeks gestation. They also had higher rates of FGR and delivery for fetal indications. This may reflect more severe placental dysfunction in this group and the importance of individualized fetal surveillance strategies even in the absence of maternal comorbidities.

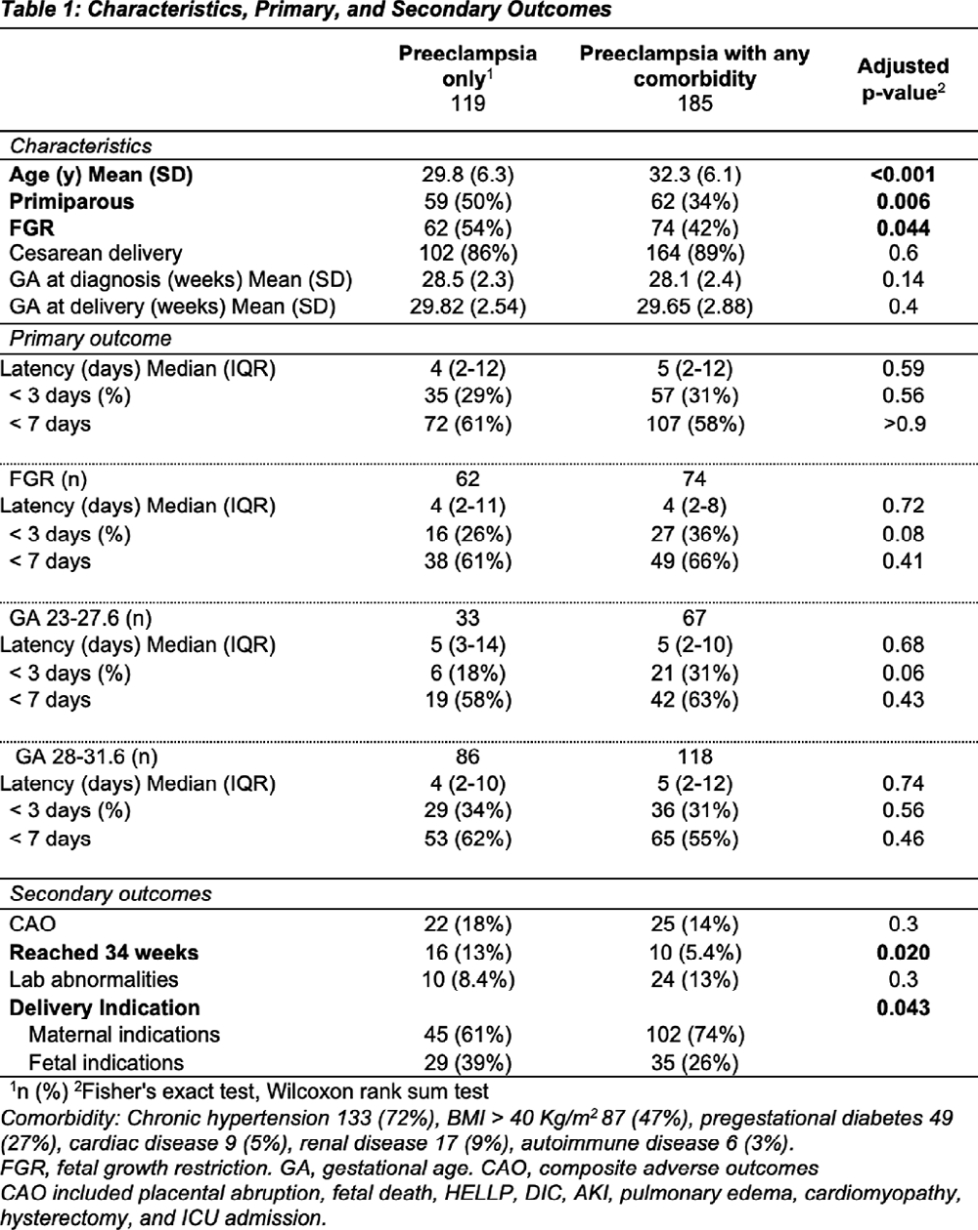

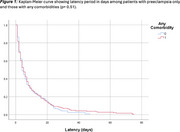

**3:30 PM – 5:00 PM**

## 593 | **Delayed Interval Delivery in Multiple Gestations at Periviability–A Multicenter Retrospective Cohort Study**

Megan Stevenson^1^; Melanie Maykin^2^; Kelly Yamasato^2^; Christine Carlos^3^; Srishti Jayakumar^4^; Danielle LoRe^5^; Mina Sohrabi^6^; Aunum Akhter^7^; Jasmine Soo^8^; Richard Kampanatkosol^9^; Uchenna Anani^10^; Olivia Kammegne‐Simo^10^; Chondraah Holmes^10^; Tiffany Tonismae^11^; Mobolaji Famuyide^12^

On behalf of the Investigating Neonatal Decisions for Extremely Early Deliveries (INDEED) Study Group

^1^Yale School of Medicine, New Haven, CT; ^2^University of Hawaii John A. Burns School of Medicine, Honolulu, HI; ^3^University of Chicago, Chicago, IL; ^4^Johns Hopkins University School of Medicine, Baltimore, MD; ^5^Hackensack University Medical Center, Hackensack, NJ; ^6^Advocate Christ Hospital, Oak Lawn, IL; ^7^University of Iowa Health Care, Iowa City, IA; ^8^University of Colorado, Aurora, CO; ^9^Rush University Medical Center, Chicago, IL; ^10^Vanderbilt University Medical Center, Nashville, TN; ^11^University of Louisville Health, Louisville, KY; ^12^University of Mississippi Medical Center, Jackson, MS

**Objective**: Delayed interval delivery (DID) is a rare strategy for preterm multiple gestations where delivery of the remaining fetus(es) is deferred following the delivery of the first infant, with the goal of improving outcomes for the retained fetuses. Clinical outcome data is limited due to the rarity of this scenario, which adds to the complexity of counseling patients who are considering DID. We sought to summarize contemporary obstetric care and neonatal outcomes in the setting of DID at periviability in a multicenter cohort.

**Study Design**: This multicenter retrospective cohort study analyzed data on periviable births (22w0d‐24w6d) from 13 institutions between 2010–2020. From this cohort, we obtained cases of delayed interval deliveries where data was available for the retained twin. Management and outcome data were compiled for all viable fetuses. Deliveries were then stratified by the viability of Twin A at delivery and neonatal outcomes were compared between the two groups.

**Results**: 13 pregnant persons and 20 infants from 6 institutions were included. All pregnancies were dichorionic‐diamniotic twins who delivered from 2013–2019. The median intertwin delivery interval was 7.5 days (range 29 hours–5 weeks) and was longer when Twin A delivered before 22 weeks compared to after 22 weeks (median 14 days vs. 5 days 10 hours). When Twin A delivered liveborn between 22–24 weeks, survival was 80% (4/5) for Twin A compared to 87.5% (7/8) for Twin B among those with resuscitation. When Twin A delivered prior to 22 weeks and Twin B delivered periviable, there were no Twin B survivors (0/2) among those with resuscitation. The intraamniotic infection rate was 14.3% (1/7) in the Twin A group and 53.8% (7/13) in the Twin B group.

**Conclusion**: In this multi‐institutional, contemporary periviable cohort, DID has the potential to improve neonatal survival in the retained twin but is associated with a higher risk of intraamniotic infection. These findings contribute to the limited data on DID at periviability and may assist clinicians when counseling patients in this complex scenario.

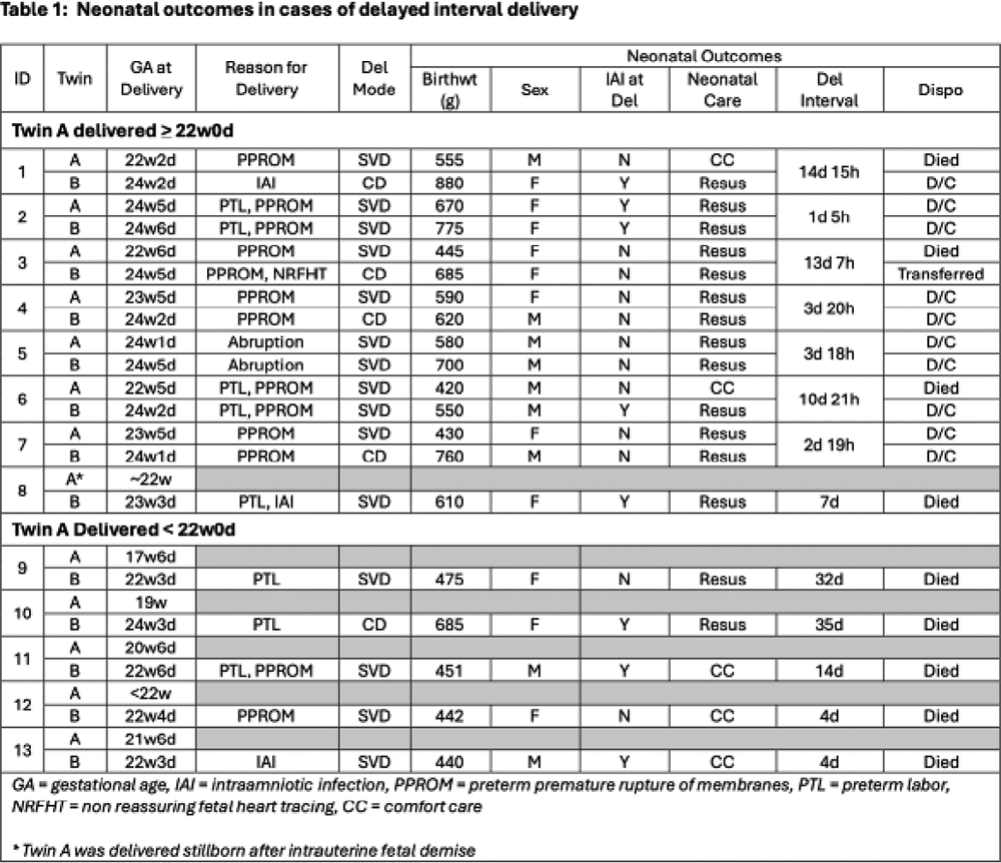

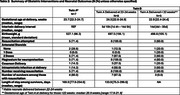

**3:30 PM – 5:00 PM**

## 594 | **Maternal Body** Mass **Index, Treatment for Gestational Diabetes and Delivery Outcomes**

Meghan Taylor^1^; Karina Pone^1^; Linda M. Szymanski^1^; Kylie Cooper^1^; Samyuktha Prabu^2^; Carl H. Rose^1^; Aoife M. Egan^1^

^1^Mayo Clinic, Rochester, MN; ^2^University of Central Florida, Internal Medicine Residency, Orlando, FL

**Objective**: 14% of pregnancies worldwide are affected by gestational diabetes mellitus (GDM). GDM has been associated with an increased risk of adverse delivery outcomes including an increase in cesarean delivery by 30% and prematurity by nearly 70%. Maternal obesity is an additional risk factor for these adverse outcomes, but data on further stratification of GDM‐related risks by maternal BMI category and GDM treatment are limited. We hypothesized that the need for pharmacologic treatment for GDM would compound the risk of adverse outcomes beyond that associated with GDM and maternal obesity alone.

**Study Design**: This is a retrospective cohort study of singleton pregnancies in individuals ≥18 years old diagnosed with GDM receiving prenatal care at Mayo Clinic Rochester, Minnesota and three outlying Mayo Clinic Health System community sites from 2018–2022. Universal GDM screening was employed with a 50 g oral glucose challenge test ± a 100 g oral glucose tolerance test. Maternal demographics, preexisting medical conditions, and selected obstetric and neonatal morbidities were evaluated.

**Results**: 2193 pregnancies in 2110 individuals affected by GDM were identified. 506 (23.1%) of these pregnancies occurred in patients with a normal BMI (18.5–25 kg/m^2^), 596 (27.2%) with overweight BMI (25–30 kg/m^2^), and 1091 (49.7%) with obese BMI (≥30 kg/m^2^). Cesarean delivery was higher in patients with obesity compared to overweight and normal weight patients (*p* < 0.001, table 1), but the requirement for glucose lowering therapy did not further increase this risk. Premature delivery was not significantly higher across maternal BMI categories (*p* = 0.12, table 1), although gestational age at delivery was higher in those with normal BMI. There were no significant differences between those that did and did not require pharmacologic therapy (table 2).

**Conclusion**: Patients with GDM and an obese BMI had increased adverse delivery outcomes when compared to those with GDM in the normal and overweight BMI categories. The need for pharmacotherapy for GDM did not further increase these risks.

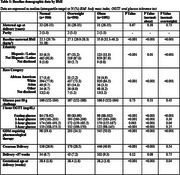

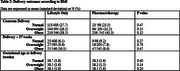

**3:30 PM – 5:00 PM**

## 595 | **Cost‐**Effectiveness **of Oral vs. IV Iron Treatment for Iron Deficiency Anemia in Pregnancy**

Meghana Narahari^1^; Amelia H. Gagliuso^2^; Lily Ben‐Avi^3^; Ashley E. Benson^4^; Aaron B. Caughey^3^

^1^Oregon Health and Science University, Camas, WA; ^2^Oregon Health and Science University, Portland, OR; ^3^Oregon Health & Science University, Oregon Health & Science University, OR; ^4^Oregon Health & Science University, Portland, OR

**Objective**: Iron deficiency anemia (IDA) in pregnancy is a common condition which affects about 23% of all pregnancies. Increasing severity of IDA is associated with progressively worse maternal and neonatal outcomes. A recent randomized trial found that pregnant patients with IDA treated with intravenous (IV) iron had lower rates of mild, moderate and severe anemia compared to those treated with oral iron. This analysis evaluated the cost‐effectiveness of oral ferrous sulfate compared to IV low molecular weight dextran (IV ID) for IDA in pregnancy.

**Study Design**: We constructed a decision‐analytic model using TreeAge to compare outcomes in pregnant patients with IDA who were treated with IV versus oral iron. The modeled cohort consisted of 842,787 individuals and was calculated based on the prevalence of IDA in pregnancy. Clinical outcomes measured in the model included preeclampsia, stillbirth, preterm birth, neonatal death and neurodevelopmental disability. Probabilities, utilities and costs were derived from literature. QALYs were discounted at a rate of 3%.

**Results**: In this theoretical cohort, IV ID was associated with a reduction in 1294 cases of preeclampsia, 1760 cases of stillbirth, 12,016 cases of preterm birth, 101 cases of neonatal death and 103 cases of neurodevelopmental delay. IV ID was the dominant strategy as it saved $680,246,993 and resulted in an additional 439,279 QALYs.

**Conclusion**: We found that the IV ID strategy resulted in savings of $680,246,993 and 439,279 additional QALYs. Considering the significant decrease in morbidity and cost, the results of our model support the use of IV ID over oral ferrous sulfate.

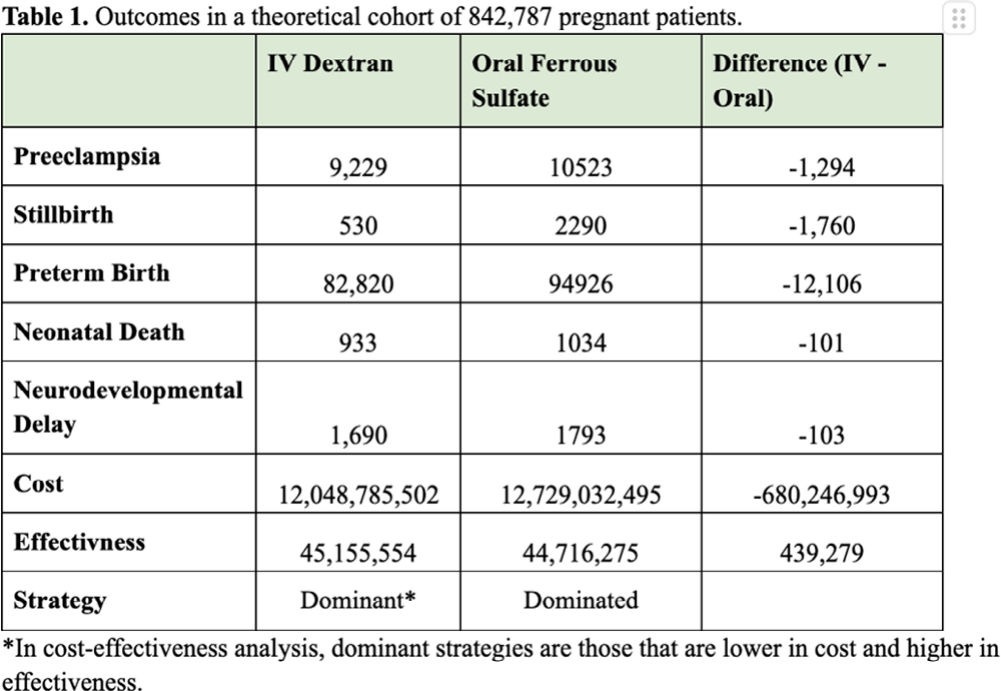

**3:30 PM – 5:00 PM**

## 596 | **Perinatal AT1‐AA Exposure is a Risk Factor for Preeclampsia**

Melissa S. Helmich^1^; Nathan Campbell^1^; Ella Goolsby^1^; Emma Grace Joyner^1^; Alexandra Demesa^1^; Evan Lamarca^1^; Ty Turner^1^; Baoying Zheng^1^; Babbette LaMarca^2^

^1^University of Mississippi Medical Center, Jackson, MS; ^2^The University of Mississippi Medical Center, Jackson, MS

**Objective**: Preeclampsia (PE) is new‐onset hypertension with multi‐organ dysfunction during pregnancy. Female children from PE pregnancies are at risk for developing PE during pregnancy, indicating a familial connection. Agonistic autoantibodies to the angiotensin II type 1 receptor (AT1‐AA) are produced in PE women and cause a PE phenotype in pregnant rats. We have shown that perinatal AT1‐AA exposure causes adult female offspring to have elevated blood pressure. However, the role of perinatal AT1‐AA exposure to increase the risk for a PE phenotype is unknown. Thus, we hypothesize that perinatal AT1‐AA exposure will cause female offspring to develop hypertension and fetal growth restriction during pregnancy.

**Study Design**: We infused AT1‐AA (1:40) from gestational day (GD)14 throughout pregnancy and allowed the dams (F0) to give birth. Birth weight was measured within 12 hours. OS (F1) were aged to 3 months. Control males and females or AT1‐AA females were mated. Pregnancy was confirmed by vaginal smear. On GD18 carotid catheters were inserted. On GD19, mean arterial pressure (MAP), weights, and litter sizes were measured. Placental and renal preproendothelin were measured by RT‐PCR. Immune cells were measured with flow cytometry. A student's t‐test was used for statistical analysis.

**Results**: F1 AT1‐AA had increased MAP and smaller litters compared to F1 NP (117 ± 3 mmHg, *p* < 0.05 vs 105 ± 2 mmHg) (7 ± 2, vs 13 ± 1 pups, *p* < 0.05). Placental T helper cells were significantly increased in F1 AT1‐AA (34 ± 3 % gated) compared to F1 NP (16 ± 3 % gated) with decreased T regulatory cells in F1 AT1‐AA (0.44 ± 0.12 % gated) compared to F1 NP (1.27 ± 0.40 % gated). AT1‐AA female F1 conceived earlier than normal pregnant (NP) female F1 (14 ± 2 weeks, vs 16 ± 3 weeks, *p* < 0.05). F1 AT1‐AA were smaller at gestational day 19 than F1 NP (293 ± 5g, vs 338 ± 7g, *p* < 0.0001). Placental PPET and renal PPET were significantly increased 9.5 ± 2.0 fold; 7.5 ± 2.5 fold in F1 AT1‐AA compared to F1 NP.

**Conclusion**: These data demonstrate that perinatal AT1‐AA exposure predisposes F1 females toward PE phenotype thereby contributing to the familial risk for PE.

**3:30 PM – 5:00 PM**

## 597 | **Enhanced** Recovery **After Surgery Regimens for Cesarean Deliveries complicated by Conditions Contraindicating NSAID and Acetaminophen**

Daniel J. Martingano^1^; Melody Boafo^2^; Andrea Rivera^3^; Crystal Awad^4^; Marwah Al‐Dulaimi^5^; Sarrah Dominique^2^; Samantha Giler^1^; Jaina Diaz‐Kelly^2^; Kelly Mondey^2^; Brenna M. Regan^6^; Kristen Henry^2^; Eddie Santana^7^; Angelo Oduro^1^; Jacqueline Marecheau^2^; Francis X. Martingano^8^

^1^St. John's Episcopal Hospital‐South Shore / Lake Erie College of Osteopathic Medicine, St. John's Episcopal Hospital‐South Shore / Far Rockaway, NY; ^2^St. John's Episcopal Hospital‐South Shore, St. John's Episcopal Hospital‐South Shore / Far Rockaway, NY; ^3^Wyckoff Heights Medical Center, Wyckoff Heights Medical Center / Brooklyn, NY; ^4^Jamaica Hospital Medical Center, Jamaica Hospital Medical Center / Queens, NY; ^5^St. John's Episcopal Hospital‐South Shore, St. John's Episcopal Hospital‐South Shore, NY; ^6^William Carey University College of Osteopathic Medicine, William Carey University / Hattiesburg, MS; ^7^Good Samaritan University Hospital / NYIT, Good Samaritan University Hospital / West Islip, NY; ^8^NYU Langone Medical Center / NYU School of Medicine, NYU Langone Medical Center / New York, NY

**Objective**: Enhanced recovery after surgery for cesarean deliveries (ERAS‐C) protols provide improved outcomes and mitigation of complications. Non‐steroidal anti‐inflammatory medications (NSAID) and acetaminophen (APAP) remain a staple therein, making cases contraindications to NSAID and APAP (NXA) challenging. This study sought to evaluate ERAS‐C protocols utilizing alternative medication regimens in pregnancies complicated by NXA.

**Study Design**: We conducted a multi‐center, prospective observational study from 7/2020 to 7/2025 and included all pregnant women with NXA utilizing ERAS‐C protocols. Regimens included those that substituted NSAIDs for tramadol and APAP for gabapentin (TX1), and those that substituted NSAIDs for cyclobenzaprine and APAP for pregabalin (TX2). Each regimen was compared to the total patients receiving a different regimen. Both protocols otherwise utilized the same medications and procedures. Primary outcomes included length of stay exceeding 3 days postoperative (LOS), postoperative ileus, need for additional medications (MED), addition of opioid medications (OM), and subsequent emergency room visits for pain (EDP), as discrete events. Patients with multi‐fetal gestations, concurrent infectious diseases, diabetes in pregnancy, or intrapartum morphine or butorphanol use were excluded.

**Results**: The study included 333 patients, with 183 receiving TX1 and 150 receiving TX2. Baseline demographic factors were not significantly different. Patients receiving TX1 had lower rates of OM (29.5% v. 34.0% *p* = 0.005) and MED (27.3% v. 32.9%, *p* < 0.001 with an 61% (RR = 0.39, 95% CI 0.17–0.22, *p* = 0.003) and 69% (RR = 0.31, 95% CI 0.19–0.23, *p* = 0.008) decreased risk in confounder‐adjusted models, respectively. Patients receiving TX2 had lower rates of LOS (12.6% v. 16.9% *p* = 0.022) and EDP(10.0% v. 15.3% *p* = 0.040), with a 11% (RR = 0.89, 95% CI 0.77–0.96, *p* = 0.045) and 14% (RR = 0.86, 95% CI 0.81–0.98, *p* = 0.050) decreased risk in adjusted models.

**Conclusion**: Pregabalin, gabapentin, tramadol, and cyclobenzaprine are safe and efficacious alternatives for NSAIDs and APAP, with potential respective benefits for ERAS‐C.

**3:30 PM – 5:00 PM**

## 598 | **Recurrence of** Hypertensive **Disorders of Pregnancy: a Longitudinal Study of Live Births in California, 2016–2021**

Meralis V. Lantigua‐Martinez^1^; Rebecca J. Baer^2^; Scott P. Oltman^2^; Laura Jelliffe‐Pawlowski^3^; Justin S. Brandt^1^

^1^Division of Maternal‐Fetal Medicine, Department of Obstetrics and Gynecology, NYU Grossman School of Medicine, New York, NY; ^2^UCSF, San Francisco, CA; ^3^NYU, New York, NY

**Objective**: To assess the recurrence risk of hypertensive disorders of pregnancy (HDP), identify risk factors associated with recurrent HDP (R‐HDP), and quantify the impact of gestational age of delivery in the para 1 (P1) pregnancy on R‐HDP and associated preterm birth (PTB) in their para 2 (P2) pregnancy.

**Study Design**: We performed a longitudinal study of pregnant people with consecutive liveborn singleton births in California (2016–2021) using birth certificates linked to hospital discharge records. We included subjects who had HDP in their P1 pregnancies. Demographic and obstetric risk factors for R‐HDP were calculated using Poisson regression models as was associated PTB. The risk of R‐HDP was also evaluated by gestational age of delivery in P1 pregnancies. Models were adjusted for demographic and obstetric factors.

**Results**: During the study period, there were 20,166 people with HDP in P1 pregnancies who had subsequent linked P2 pregnancies. The overall recurrence rate of HDP was 30.5%; recurrence varied by type of HDP in P1 pregnancy, ranging from 27% for gestational hypertension to 45% for superimposed preeclampsia. Among people with HDP in P1, the risk of R‐HDP was higher for people with advanced maternal age compared to age 18–34 (relative risk [RR] 1.2, 95% confidence interval [CI] 1.1,1.3), drug or alcohol use compared to none (RR 1.8, 95% CI 1.6,2.0), housing instability compared to stable housing (RR 2.1, 95% CI 1.1,4.1), and mental health conditions compared to none (RR 1.5, 95% CI 1.4,1.6) (Table 1). In the analysis of risk of R‐HDP by gestational age of delivery in P1 pregnancies, we found the highest risk of R‐HDP and PTB <34 weeks occurred in people with PTB prior to 28 weeks in their index pregnancies (adjusted RR 13.3, 95% CI 4.7,37.7) and remained elevated for any history of PTB with HDP (adjusted RR 7.9, 95% CI 5.6,11.1) (Table 2).

**Conclusion**: The recurrence rate of HDP and associated PTB varies by severity of HDP and gestational age at delivery in the index pregnancy. Further prospective studies on mitigating modifiable risk factors are needed to design targeted preventative interventions.

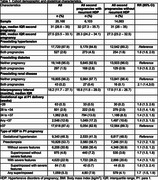

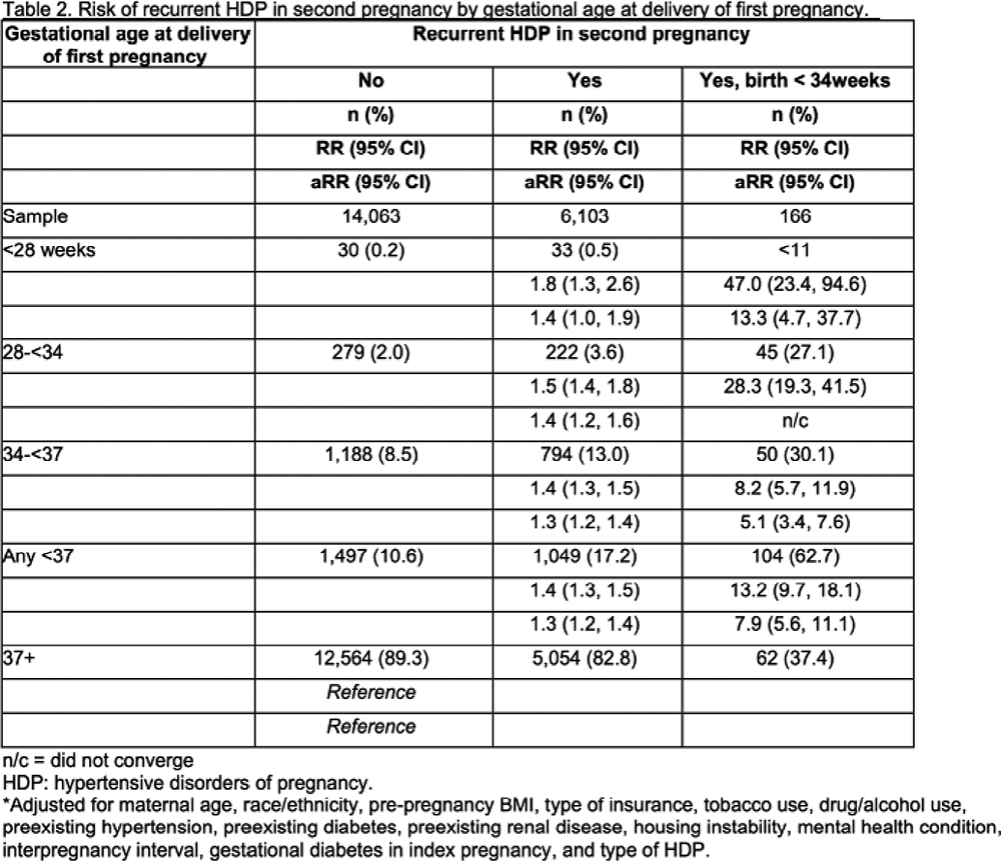

**3:30 PM – 5:00 PM**

## 599 | **Outcomes After** Implementation **of a Hybrid Virtual/In‐Person Prenatal Care Model**

Michael J. Fassett^1^; Emily V. West^2^; Ericka C. Gibson^3^; Linda S. Chong Tim^4^; Chantal C. Avila^5^; Jiaxiao M. Shi^5^; Vicky Y. Chiu^5^; Joanie WL Chung^5^; Erica E. Rudolph^6^; Nehaa Khadka^5^; Christopher J. Jentz^6^; David M. Mosen^7^; Jemma C. Nonog^8^; Jocelyn W. Audelo^9^; Darios Getahun^10^

^1^Department of Obstetrics and Gynecology, Kaiser Permanente West Los Angeles Medical Center, Los Angeles, CA; ^2^Kaiser Permanente Northwest, Clackamas, OR; ^3^Southeast Permanente Medical Group, Atlanta, GA; ^4^Hawaii Permanente Medical Group, Honolulu, HI; ^5^Kaiser Permanente Southern California, Pasadena, CA; ^6^Kaiser Permanente Care Management Institute, Oakland, CA; ^7^Kaiser Permanente Center for Health Research, Portland, OR; ^8^The Permanente Federation, Oakland, CA; ^9^Kaiser Foundation Health Plan, Oakland, CA; ^10^Department of Research & Evaluation, Kaiser Permanente Southern California, Pasadena, CA

**Objective**: Barriers to in‐person care during the COVID‐19 pandemic led to development of a blended virtual/in‐person compared to traditional face‐to‐face prenatal care. We evaluated perinatal outcomes after implementing this blended model in a large health maintenance organization.

**Study Design**: A matched case‐control study was conducted at Kaiser Permanente Northwest and Southern California, comparing the blended virtual/in‐person care model (cases, 7/22/2022–12/31/2024) with the traditional face‐to‐face care model (controls, 1/1/2018–12/31/2019).

**Results**: Among 41,538 patients (473 cases; 41,065 controls), cases were older at delivery (32.0 ± 4.6 vs. 30.3 ± 4.9 y, *p* < .0001), more likely to be non‐Hispanic whites (68.9% vs. 30.0%, *p* < 0.0001), had higher median household income, and higher rates of publicly‐funded coverage (15.9% vs. 11.2%, *p* < .0001). Cases were fewer first‐time mothers (20.9% vs. 39.8%, *p* < .0001), with lower alcohol/substance use but higher smoking rates. Prenatal care initiation in the first trimester was lower (92.6% vs. 94.1%). Cases had fewer in‐person visits (6.2 vs. 13.1, *p* < 0.0001), more virtual visits (3.2 vs. 0.8, *p* < .0001), and fewer total visits (9.4 vs. 13.9, *p* < .0001). Depression screening was more frequent in cases (84.1% vs. 41.9%, *p* < .0001), while gestational diabetes screening was less frequent (32.3% vs. 90.4%, *p* < .0001). Compared to controls, cases had lower odds of preterm PROM (OR 0.23, 95% CI 0.07–0.79) and preterm birth <37 weeks (OR 0.51, 95% CI 0.31–0.85), but higher odds of non‐reassuring fetal heart rate tracing (OR 1.29, 9% CI 1.05–1.59) and macrosomia (OR 4.69, 95% CI 1.10–19.91). No significant differences were observed in placenta previa, chorioamnionitis, cesarean birth, postpartum hemorrhage, or maternal length of stay >2 days.

**Conclusion**: Despite differences in patient characteristics and rates of depression and gestational diabetes screening, perinatal outcomes were similar in patients who received prenatal care through a blended in‐person/virtual model compared to those who received exclusively in‐person care.

**3:30 PM – 5:00 PM**

## 600 | **Transfusion Risk** Among **Pregnant Patients With Prior Metabolic and Bariatric Surgery**

Michael T. Xie^1^; Sarah H. Abelman^2^; Frank I. Jackson^2^; Oladunni Ogundipe^2^; Jamie Green^2^; Esther Miller^3^; Elisheva Miller^3^; Matthew J. Blitz^2^

^1^Donald and Barbara Zucker School of Medicine at Hofstra/Northwell, Bay Shore, NY; ^2^Northwell, New Hyde Park, NY; ^3^New York Medical College, Westchester, NY

**Objective**: This study aimed to assess associations between history of metabolic and bariatric surgery (MBS), surgery type, and the risk of receiving blood transfusion during delivery admission.

**Study Design**: This retrospective cross‐sectional study included all deliveries at ≥23 weeks of gestation across seven hospitals within a large New York health system between 2019–2024. Pregnancies with multiple gestations or intrauterine fetal demise were excluded. The primary exposure was a history of MBS. Multivariable logistic regression analysis was used to evaluate the associations between history of MBS and the primary outcome: the odds of receiving a blood transfusion during their delivery admission, adjusting for anemia, race and ethnicity, insurance, history of preterm birth, and multiparity. To examine associations between the type of MBS and transfusion risk, a subgroup analysis of patients with a history of bariatric surgery was conducted with the type of MBS as the primary exposure grouped as: Roux‐en‐Y gastric bypass, sleeve gastrectomy, gastric banding, or unknown.

**Results**: Among the 156,923 deliveries included, 1815 patients had a history of MBS. The rate of transfusion was 3.0% in patients with no history of bariatric surgery and 5.3% in patients with prior MBS. Post‐MBS pregnancy patients were significantly more likely to receive a transfusion during their delivery admission (aOR 1.5, 95% CI 1.03–2.1). Among those with a history of MBS, 48.5% had a sleeve gastrectomy, 6.8% had gastric banding, 23.2% had a gastric bypass, 9.5% had a revision or conversion, and 21.4% had an unknown MBS. The type of MBS was not associated with an increased risk of blood transfusion.

**Conclusion**: Pregnant patients with a history of MBS were significantly more likely to receive a blood transfusion at their delivery.

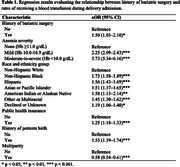

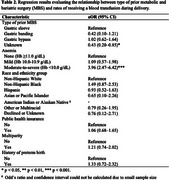

**3:30 PM – 5:00 PM**

## 601 | **External Cephalic Version in Suspected Growth‐Restricted Fetuses: Safety and Success in a Large Cohort**

Michal Axelrod^1^; Shlomi Toussia‐Cohen^2^; Tal Cahan^3^; Ronit Silber^3^; Shir Koren^3^; Maayan Mandelbaum^4^; Chen Berkovitz^3^; Orit Moran^3^

^1^Sheba Medical Center, Sheba Medical Center, HaMerkaz; ^2^The Sheba Medical Center, The Sheba Medical Center, HaMerkaz; ^3^Sheba Medical Center, Ramat Gan, HaMerkaz; ^4^Faculty of Medicine, Tel Aviv University, Israel, Tel Aviv, HaMerkaz

**Objective**: To assess the safety and success rate of external cephalic version (ECV) in pregnancies with suspected small‐for‐gestational‐age (SGA) fetuses, including those with fetal growth restriction (FGR), defined as estimated fetal weight (EFW) ≤ the 3rd percentile.

**Study Design**: A retrospective cohort study was conducted at a large tertiary center between 2011–2024. Singleton pregnancies undergoing ECV with an EFW assessed within two weeks prior to the procedure, based on Hadlock 4 curves, were included. Patients were categorized into SGA (<10th percentile) and appropriate‐for‐gestational‐age (AGA) groups, large‐for‐gestational‐age (LGA) were excluded. Baseline characteristics, ECV success, complications, and perinatal outcomes were compared.

**Results**: Of 1152 women, 195 (16.9%) were classified as SGA, including 31 (2.7%) with FGR. The AGA group included 957 women (83.1%). Baseline characteristics were generally comparable, except for lower BMI in the SGA group (25.8 vs. 27.5, *p* < 0.001). There was a trend toward higher rates of polyhydramnios in AGA cases (*p* = 0.069). ECV success rates were similar between SGA and AGA (54.4% vs. 56.8%, *p* = 0.524), and slightly lower among FGR cases (45.2%). Complications were rare and limited to isolated episodes of variable fetal heart rate decelerations, observed more frequently in SGA (2.1% vs. 0.6%, *p* = 0.072), including one event (3.2%) in the FGR subgroup. Spontaneous reversion to breech following successful ECV occurred at similar rates across groups (2.6% vs. 3.1%, *p* = 0.715). Mode of delivery did not differ significantly. Postnatal birth weights confirmed that not all prenatally suspected to be SGA were truly growth restricted (60.5%).

**Conclusion**: ECV appears safe and effective in pregnancies with suspected SGA, including those with EFW below the 3rd percentile. These findings support the inclusion of select growth‐restricted fetuses in ECV candidates and underscore the limitations of prenatal SGA classification.

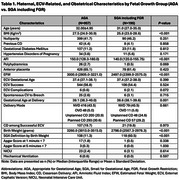

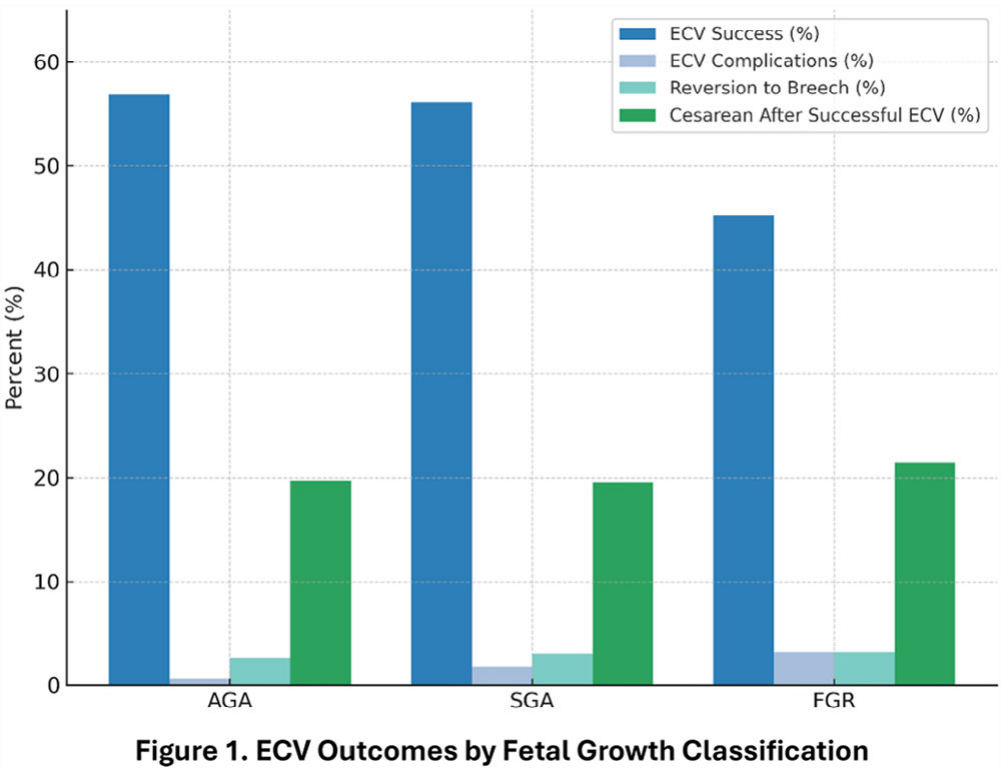

**3:30 PM – 5:00 PM**

## 602 | **Targeting (Pro)**Renin **and Its Receptor: A Potential Biomarker and Therapeutic Approach for Preeclampsia**

Michelle N. Harris^1^; Ashleigh Aubin^2^; Kelsey R. Kelso^1^; Diana Villazana‐Kretzer^3^; Jessica C. Ehrig^4^; Ram R. Kalagiri^5^; Niraj Vora^3^; Mohammad N. Uddin^6^; Ahmed Pantho^7^; Thomas Kuehl^8^

^1^Baylor Scott & White Health ‐ Baylor College of Medicine, Temple, TX; ^2^Baylor Scott and White Health, Temple, TX; ^3^Baylor Scott & White Medical Center, Temple, TX; ^4^Baylor Scott & White Medical Center‐Temple, Temple, TX; ^5^Baylor Scott and White Health, Te, TX; ^6^Texas A&M Health University College of Medicine, Temple, TX; ^7^Orion Institute for Translational Medicine, Temple, TX; ^8^Texas A&M College of Medicine, Bryan, TX

**Objective**: While the etiology of Pre‐eclampsia (PreE) is unclear, the renin‐angiotensin system has been implicated. Previous studies found high circulating levels of (pro)renin ((P)R) and soluble (pro)renin receptor (s(P)RR) in patients with PreE, highlighting potential as biomarkers. Increased placental (P)RR was found in patients with preE and in a rat model of PreE. A peptide mimicking the (P)RR Handle Region (HRP) attenuated preE effects on blood pressure (BP), urinary protein (UP), number of pups, and number of normal pups.

Our goal was to evaluate (P)R and s(P)RR trends in PreE patients to understand biomarker utility and detection timing. We aimed to understand the dose‐dependent effects of HRP fusion protein (HRP‐Fc) on outcomes in PreE model rats.

**Study Design**: Pregnant rats were assigned to three groups: 1) Normal Pregnant (NP), 2) PDS, in which the rats were given saline to drink and injected with desoxycorticosterone acetate (DOCA) to induce hypertension, and 3) PDS‐HRP, DOCA rats given high (HRP‐Fc‐Hi), medium (HRP‐Fc‐Med), or low doses (HRP‐Fc‐Lo) of HRP‐Fc. BP, UP, total pups, and number of anormal pups were measured. Results are shown in Table 1. 76 women were recruited in the first trimester. Serum PR and s(P)RR were analyzed at four time points throughout pregnancy. Serum levels of (P)R and s(P)RR in the 21 patients who developed PreE were compared to levels in those who did not. Results are shown in Table 2.

**Results**: The HRP‐Fc normalized BP, UP, total pups, and number of abnormal pups in the DOCA rats in a dose‐dependent manner, with high‐dose HRP‐Fc most effective. There was a statistically significant increase in (P)R and s(P)RR in PreE women with most significant rise in the second trimester.

**Conclusion**: These data suggest a role of (P)R and the RAS cascade in the development of PreE. Elevated (P)R and s(P)RR in women with PreE suggest its utility as a serum biomarker for the disease. Attenuation of PreE effects by the HRP‐Fc suggests its use as a therapeutic agent, and higher doses were more effective than lower doses.

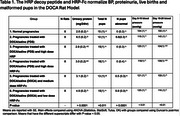

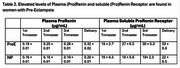

**3:30 PM – 5:00 PM**

## 603 | **Obstetric Anal** Sphincter **Injury Repair Proficiency Among Maternal‐Fetal Medicine Fellow Physicians**

Minhazur R. Sarker^1^; Rachel L. Wiley^1^; Dana R. Canfield^2^; Yalda Afshar^3^; Kelsey Rose^3^; Scott Harvey^4^; E. Nicole Nicole Teal^1^

^1^Division of Maternal Fetal Medicine, Department of Obstetrics, Gynecology and Reproductive Sciences, University of California San Diego, San Diego, CA; ^2^Department of Obstetrics & Gynecology, University of Washington, Seattle, WA, Seattle, WA; ^3^David Geffen School of Medicine at University of California, Los Angeles (UCLA), Los Angeles, CA; ^4^Department of Obstetrics, Gynecology and Reproductive Science, University of California San Diego, San Diego, CA

**Objective**: Suboptimal repair of obstetric anal sphincter injuries (OASIS) is associated with complications such as repair breakdown, infection, incontinence, and worse quality of life. In current practice, obstetrics and gynecology trainees have less exposure to OASIS repairs. This study aims to characterize OASIS repair proficiency among maternal‐fetal medicine (MFM) fellow physicians–a group of physicians obtaining additional training in clinical obstetrics.

**Study Design**: We conducted a cross‐sectional survey of MFM fellows between January to June 2025. The survey was distributed primarily by email to fellows, program directors, and program coordinators. The primary outcome was fellow comfort with OASIS repairs (3^rd^ degree or 4^th^ degree. Secondary outcomes included training characteristics and perception of respective programs’ training adequacy. This study was deemed exempt by the Institutional Review Board.

**Results**: Of the 411 fellows, 162 (39.4%) responded with no differences in fellowship year, region, or type of institution. Among respondents, 93.8% participated in OASIS repair simulations and felt they were helpful (Table 1). More than half of respondents (53.1%) reported repairing more than 10 3^rd^ degree perineal lacerations and feeling comfortable teaching the repair (Table 2). Conversely, only 26.5% of respondents reported repairing 5 or more 4^th^ degree perineal lacerations and less comfort with teaching this skill (Table 2). While 92.6% of respondents felt residency provided adequate 3^rd^ degree repair training, only 62.4% felt similarly for 4^th^ degree repair training (Table 2). Moreover, only 61.7% and 38.3% of respondents felt fellowship provides adequate training for 3^rd^ and 4^th^ degree perineal lacerations, respectively (Table 2).

**Conclusion**: While the majority of MFM fellows are comfortable repairing 3^rd^ degree lacerations, over a quarter did not feel comfortable repairing 4^th^ degree lacerations without supervision. There is a clear need to develop pathways for improvement in OASIS repair such as simulation models or workshops.

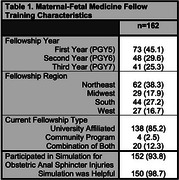

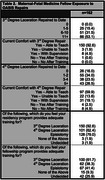

**3:30 PM – 5:00 PM**

## 604 | **Estimating** Individualized **Treatment Effects of Labor Induction at 39 Weeks on Maternal and Neonatal Outcomes**

Misa Hayasaka^1^; George R. Saade^2^; Grace Spencer^3^; Tetsuya Kawakita^1^

^1^Macon & Joan Brock Virginia Health Sciences at Old Dominion University Eastern Virginia Medical School, Norfolk, VA; ^2^Eastern Virginia Medical School at Old Dominion University, Norfolk, VA; ^3^Eastern Virginia Medical School at Old Dominion University, EVMS OBGYN, Virginia Health Sciences at Old Dominion University, VA

**Objective**: To identify baseline characteristics associated with heterogeneity in the effect of elective induction at 39 weeks on maternal and neonatal outcomes, utilizing individual treatment effect (ITE) estimates.

**Study Design**: We conducted a secondary analysis of a randomized controlled trial involving nulliparous women randomized to either elective induction at 39 weeks or expectant management. A Desirability of Outcome Ranking (DOOR) score, ranging from spontaneous vaginal delivery without severe neonatal morbidity (most favorable) to cesarean delivery with perinatal death (least favorable), was assigned on an eight‐level ordinal scale.

We employed a causal forest machine learning model to estimate ITEs for the DOOR score, based on maternal baseline characteristics. Honest splitting was used to divide the dataset into training and validation subsets. Model performance was assessed using the Q‐score. Feature importance was derived from the causal forest model. Multivariable regression was performed to enhance interpretability by examining the direction and strength of associations between baseline factors and predicted ITEs.

**Results**: Among 6096 participants, DOOR scores were skewed toward favorable outcomes, with 69% receiving the highest score. The median predicted ITE was greater than 0, and the Q‐score was 0.54. Mean differences in DOOR scores between the induction and expectant management groups increased across tertiles of predicted ITEs (Figure 1).

Feature importance analysis and multivariable regression showed that weight gain during pregnancy, BMI at randomization, gestational age at the first prenatal visit, maternal age, and Bishop score were the strongest contributors to treatment effect variation (Figure 2).

**Conclusion**: Causal forest modeling, utilizing the DOOR score as a composite and patient‐centered outcome, effectively identifies baseline characteristics associated with differential treatment benefit. This approach may facilitate the tailoring of induction strategies to patients most likely to benefit, thereby supporting shared decision‐making.

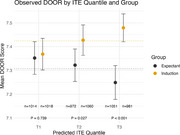

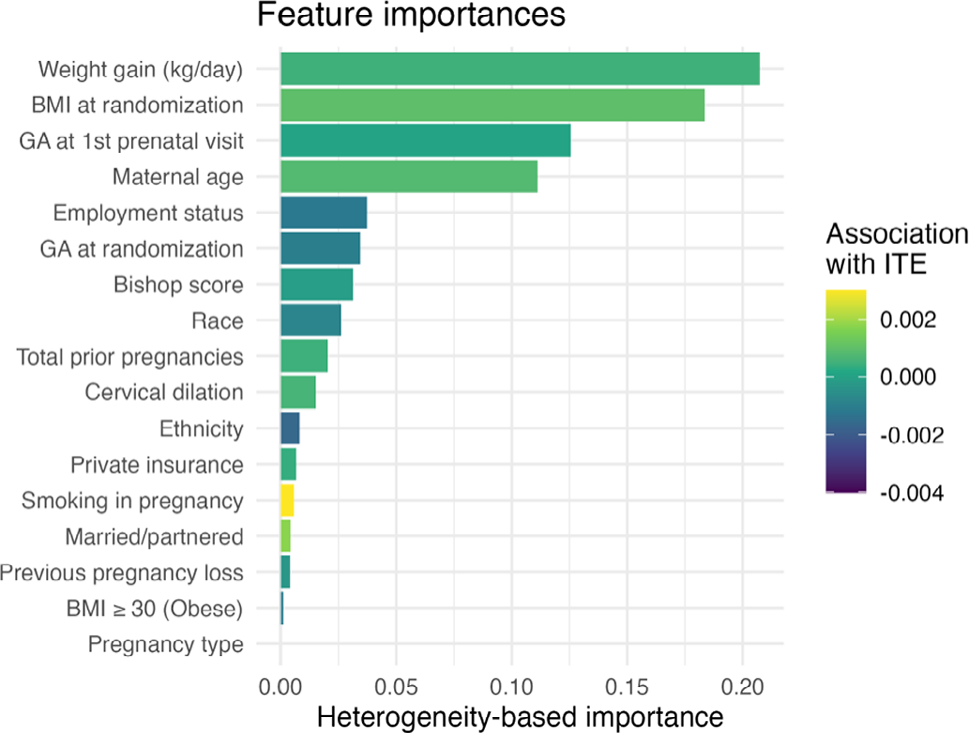

**3:30 PM – 5:00 PM**

## 605 | **Maternal Asthma and Perinatal Outcomes in California**

Molly Reese^1^; Sophia V. Gomez^2^; Amelia H. Gagliuso^2^; Bharti Garg^1^; Aaron B. Caughey^1^

^1^Oregon Health & Science University, Oregon Health & Science University, OR; ^2^Oregon Health and Science University, Portland, OR

**Objective**: Asthma is a common chronic disease in the United States, and often patients see worsening of symptom control during pregnancy. Though improvements have been made in asthma diagnosis and treatment, few large studies have been done to evaluate how the severity of maternal asthma affects pregnancy outcomes. The objective of this study is to evaluate the association between severity of asthma and maternal and neonatal outcomes.

**Study Design**: This is a retrospective cohort study using linked vital statistics and hospital discharge data for births in California (2016–2020). We included pregnant asthmatic individuals with a singleton gestation who delivered between 23 and 42 weeks. Asthma severity was categorized as mild, moderate and severe. Chi‐square tests and multivariable Poisson regression models were used to assess risk of outcomes among mild, moderate and severe asthma patients as compared to patients without asthma (reference group).

**Results**: We included a total of 1,660,227 patients, of which 0.9% had mild asthma, 0.1% had moderate, and 0.01% had severe asthma. The risk of pre‐eclampsia increased from mild (aRR = 1.32; 95% CI: 1.24–1.40) to severe asthma (aRR = 1.68; 95% CI: 1.07–2.64). Similarly, the risk of gestational hypertension, severe maternal morbidity, and preterm delivery <37 weeks increased from mild to moderate to severe asthma. When we compared neonatal outcomes, risk of respiratory distress syndrome increased from mild (aRR = 1.16; 95% CI: 1.09–1.23) to severe asthma (aRR = 1.71; 1.09–2.70). In addition, hypoglycemia, jaundice and small for gestational age also increased with rise in asthma severity.

**Conclusion**: As the severity of asthma increased, risk of maternal and neonatal outcomes increased. Despite advancements in diagnosis and treatment, severe asthma is associated with an increased risk of adverse perinatal outcomes.

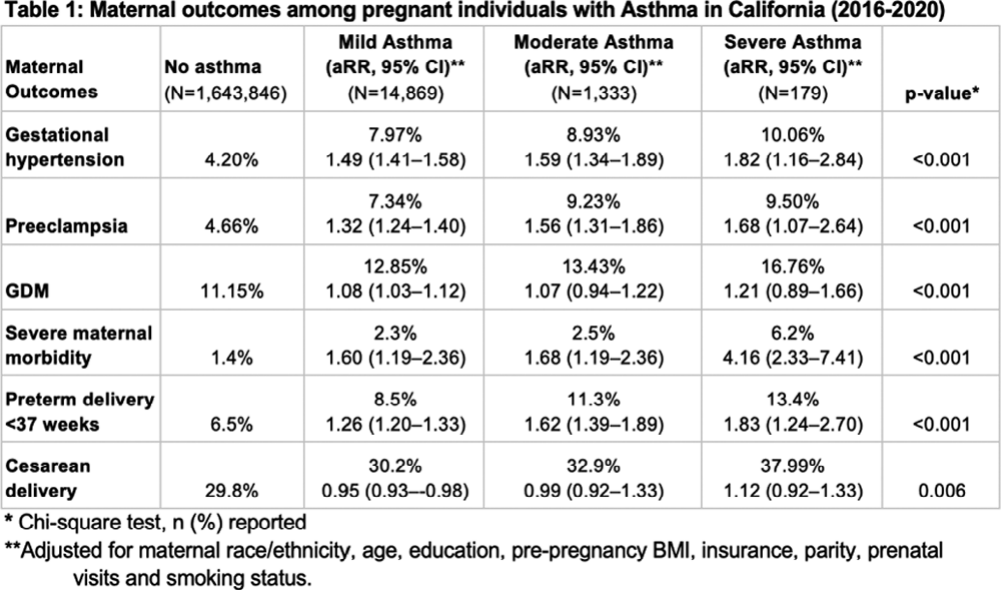

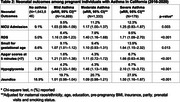

**3:30 PM – 5:00 PM**

## 606 | **Not All Single** OGTT **Abnormalities are Equal: Fasting Abnormal Value is Correlated With GDMA2**

Moran Weiss^1^; Shaked Yarza^2^; Shaked Zeevi^3^; Nofar Bar Noy‐Traub^1^; Tal Biron‐Shental^1^; Omer Weitzner^1^

^1^Meir Medical Center, Kfar Saba, HaMerkaz; ^2^Meir Medical Center, kefar saba, HaMerkaz; ^3^Meir Medical Center, Kefar Saba, HaMerkaz

**Objective**: An isolated abnormal value on the Oral Glucose Tolerance Test (OGTT) is associated with complications similar to those of gestational diabetes mellitus and is often treated the same way. In this study, we aimed to identify which of the four specific tests evaluated in the OGTT is most closely correlated with the requirement for pharmacologic treatment in gestational diabetes (GDMA2).

**Study Design**: This retrospective cohort study included women with one pathological value in OGTT over 8 years. Participants were divided into four groups based on which one of the OGTT measurements was elevated: fasting (0h), 1‐hour, 2‐hour, or 3‐hour. Obstetric and neonatal outcomes were compared across groups. Multivariable logistic regression was performed to assess associations, adjusting for potential confounding variables.

**Results**: A total of 671 women were included: Elevated fasting glucose values were found in 176 women, an increased value at one hour in 364 women, two hours in 112 women, and three hours in 19 women. The highest proportion of GDMA2 was observed among women with isolated abnormal fasting glucose (51%), whereas the rates of GDMA2 were significantly lower among those with isolated post‐load abnormalities at 1‐hour (17%), 2‐hour (19%), and 3‐hour (16%) (p < 0.001). Multivariable logistic regression analysis demonstrated that fasting elevated glucose had the strongest association with GDMA2 compared to post‐load abnormalities. Women with elevated values at 1‐hour (aOR 0.22, 95% CI 0.15–0.33, *p* < 0.001), 2‐hour (aOR 0.22, 95% CI 0.12–0.39, *p* < 0.001), and 3‐hour (aOR 0.17, 95% CI 0.04–0.55, *p* = 0.007) were significantly less likely to require pharmacologic treatment.

**Conclusion**: Among women with a single abnormal OGTT value, a fasting abnormality is associated with a significantly higher risk of GDMA2. These findings suggest that the specific abnormal test in OGTT has predictive potential for the severity of gestational diabetes mellitus.

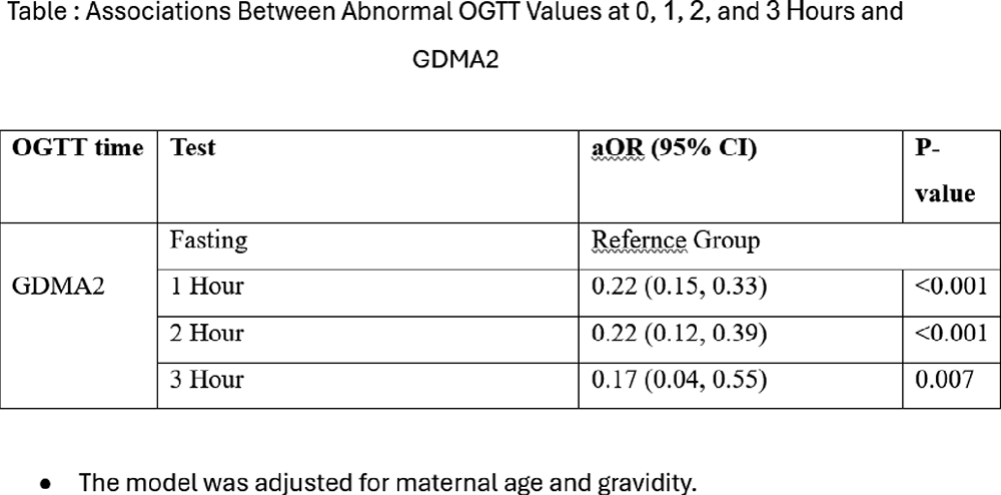

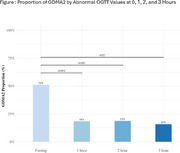

**3:30 PM – 5:00 PM**

## 607 | **Machine Learning Versus Binary Scoring to Predict Adverse Outcomes of Pregnant Patients With Diabetic Ketoacidosis**

Melissa J. Cazzell^1^; Morgan A. Scaglione^1^; Erkan Kalafat^2^; Malgorzata Mlynarczyk^1^; George R. Saade^1^; Grace Spencer^3^; Marwan Ma'ayeh^1^

^1^Eastern Virginia Medical School at Old Dominion University, Norfolk, VA; ^2^Koc University Hospital, Istanbul, Koc University Hospital, Istanbul, Istanbul; ^3^Eastern Virginia Medical School at Old Dominion University, EVMS OBGYN, Virginia Health Sciences at Old Dominion University, VA

**Objective**: To identify factors on admission that are associated with a composite adverse outcome in pregnant patients with diabetic ketoacidosis (DKA), and to determine the predictive performance of a binary scoring system versus a machine learning (ML) model.

**Study Design**: This was a retrospective cohort study of all DKA admissions from 2014–2024. The primary outcome was a composite of: ICU admission, mechanical ventilation, or a prolonged hospital stay (>14 days). We used mixed‐effects logistic regression to identify characteristics present on admission that are independently associated with the composite outcome. We then developed and compared two predictive models: 1) simple binary score based on admission glucose (>350 mg/dL), bicarbonate (<10 mEq/L), and anion gap (>20 mEq/L), and 2) ML model using restricted cubic splines. Both models were validated over 1000 repetitions on a held‐out test set, and their performance was assessed using the Area Under the Curve (AUC).

**Results**: 158 admissions from 107 pregnancies were identified. In the multivariable analysis, admission glucose (aOR 1.72, 95% CI 1.03–2.84; *p* = 0.03), anion gap (aOR 0.29, 95% CI 0.10–0.69; *p* = 0.01), and bicarbonate (aOR 0.18, 95% CI 0.07–0.41; *p* < 0.001) were independently associated with the composite outcome. Conditional density plots revealed a linear relationship for glucose, while anion gap and bicarbonate had non‐linear relationships with the outcome. The ML‐based scoring model, which incorporated these non‐linearities, had better predictive performance than the simple binary scoring system, and a risk calculator based on this model was developed. The median AUC for the ML model was 0.75 (IQR 0.69–0.81), compared to 0.70 (IQR 0.65–0.74) for the binary score, with a mean AUC difference of 0.044 (95% CI 0.039–0.049, *p* < 0.001).

**Conclusion**: Admission glucose, anion gap, and bicarbonate levels are independent predictors of adverse outcomes in pregnant patients with DKA. A ML model incorporating non‐linear relationships of these predictors has improved accuracy for risk stratification compared to a simple threshold‐based scoring system.

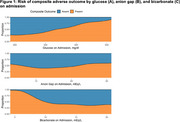

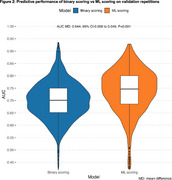

**3:30 PM – 5:00 PM**

## 608 | **Racial and Ethnic Disparities in Birth Location by Abortion Policy Environment in the United States**

Moti Gulersen^1^; Erez Lenchner^2^; Ashley Zimmermann^3^; Marlee Hirsch^4^; Itai Hazan^5^; Eran Bornstein^3^

^1^Thomas Jefferson University, PHILADELPHIA, PA; ^2^New York University Rory Meyers College of Nursing, New York, NY; ^3^Northwell, New Hyde Park, NY; ^4^Northwell, New York, NY; ^5^Ben‐Gurion University of the Negev, Beer Sheva, HaDarom

**Objective**: To assess trends in births by maternal race and ethnicity following the Dobbs v. Jackson Women's Health Organization (Dobbs) decision.

**Study Design**: Retrospective analysis of the Centers for Disease Control and Prevention Natality Live Birth database with detailed geographical data available (2018–2023). States were classified by abortion policy environment based on the Center for Reproductive Rights’ five‐tier framework. For this analysis, states classified as hostile or illegal were grouped as restrictive, while those classified as expanded access or protected access were grouped as protective. We compared rates of births in restrictive versus protective states before and after the Dobbs ruling on June 24, 2022, stratified by maternal race and ethnicity. The period from July to December 2022 was considered transitional and excluded from the analysis. Statistical significance was set at *p* < 0.05.

**Results**: The proportion of births among Hispanic patients in restrictive states increased by 12.8%, while the proportion in protective states increased by 7.6% (Figure, *p* < 0.01). Rates of births among non‐Hispanic White (NHW) and non‐Hispanic Black (NHB) patients in restrictive states decreased by 3.8% and 5.6%, respectively (Figure, *p* < 0.01). Rates of births among NHW and NHB patients in protective states also decreased by 2.4% and 6.4%, respectively (Figure, *p* < 0.01). Changes for Asian/Pacific Islander and other racial groups were small (<1% relative change) (Figure).

**Conclusion**: Following the *Dobbs* decision, birth rates in restrictive states increased most among Hispanic individuals, while non‐Hispanic White and Black populations saw declines. These trends highlight racial and ethnic disparities in the impact of abortion restrictions. Further research is needed to understand the underlying factors driving these differences.

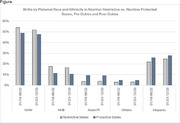

**3:30 PM – 5:00 PM**

## 609 | **Survey of Current Management Practices for Gestational Hypertension and Preeclampsia in the Postpartum Period**

Nadine Sunji^1^; Kathryn E. Flynn^1^; Alisse Hauspurg^2^; Anna Palatnik^3^

^1^Medical College of Wisconsin, Medical College of Wisconsin, WI; ^2^Alpert Medical School of Brown University, Alpert Medical School of Brown University, RI; ^3^Medical College of Wisconsin ‐ Milwaukee, WI, Milwaukee, WI

**Objective**: To assess postpartum management practices for hypertensive disorders of pregnancy (HDP) among maternal‐fetal medicine specialists.

**Study Design**: An 18‐item survey on the clinical management of postpartum HDP (gestational hypertension and preeclampsia) was designed and validated through six cognitive interviews with general OB and MFMs. The revised survey was disseminated to SMFM members via email and SMFM's Surveys of Interest webpage.

**Results**: 379 SMFM members completed the survey, with 95.2% from the U.S. and 77.2% working in academic or mixed academic‐community settings (Table 1). The most common blood pressure (BP) thresholds for initiating antihypertensive therapy postpartum were ≥140 mmHg (54.9%) and ≥150 mmHg (33.2%) for systolic BP, and ≥90 mmHg (57.3%) and ≥100 mmHg (34.6%) for diastolic BP. Most respondents (68.5%) preferred to see at least two elevated BPs before initiating therapy. Respondents used similar BP thresholds for dose escalation. 46.4% reported that their workplace has formal guidelines for postpartum HDP management. 68.3% believed that evidence‐based recommendations for antihypertensive therapy initiation endorsed by a professional society would be “very useful”, and only 24.6% reported being “satisfied” or “highly satisfied” with the available guidance. For postpartum BP monitoring, 40.6% have access to a remote BP monitoring system, and of these 47.3% enroll 75–100% of their patients in the system. 73.4% of those without remote BP monitoring reported prescribing BP cuffs at discharge from the delivery admission. Regarding magnesium sulfate administration during postpartum readmissions, 19.5% reported routinely administering it, and 65.5% indicated that administration depended on the presence of neurologic symptoms, lab abnormalities, or persistent severe‐range BPs. If indicated, 54.2% would administer magnesium sulfate up to two weeks postpartum, while 28.9% would do so up to 6 weeks postpartum (Table 2).

**Conclusion**: This survey highlighted the variation in postpartum HDP management, underscoring the need for standardization of postpartum HDP care.

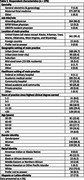

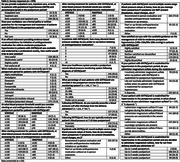

**3:30 PM – 5:00 PM**

## 610 | **Risk of Ischemic** Placental **Disease and Preterm Delivery Among Nulliparous Individuals With Insomnia**

Naima E. Ross^1^; Steven Friedman^2^; Rashmi N. Aurora^3^; Erinn M. Hade^2^; Justin S. Brandt^4^

^1^NYU Langone, NYU Langone, NY; ^2^Division of Biostatistics, Department of Population Health, NYU Grossman School of Medicine, New York, NY; ^3^Department of Medicine, NYU Grossman School of Medicine, New York, NY; ^4^Division of Maternal‐Fetal Medicine, Department of Obstetrics and Gynecology, NYU Grossman School of Medicine, New York, NY

**Objective**: To assess whether moderate/severe insomnia is associated with ischemic placental disease (IPD)—including preeclampsia (PEC), abruption, and fetal growth restriction (FGR)—and with preterm delivery (PTD).

**Study Design**: We conducted a secondary analysis of the Nulliparous Pregnancy Outcomes Study: Monitoring New Mothers‐to‐be (nuMoM2b), a prospective cohort of singleton, nulliparous individuals. We included those who underwent insomnia screening with the Women's Health Initiative Insomnia Rating Scale (WHIRS) at either one or two of the study visits (at 6–14 weeks and/or 22–30 weeks). Moderate/severe insomnia was defined as WHI scores of 15–28 at either study visit. The primary outcome was IPD, defined as a composite of PEC, abruption, or FGR. Secondary outcomes included individual IPD components and preterm delivery <37 weeks. Log‐binomial regression estimated the relative risk between groups and corresponding 95% confidence intervals (CI), adjusted for pre‐pregnancy depression and smoking.

**Results**: Among 8793 participants with WHI data, 5008 (57.0%) had moderate/severe insomnia at either visit. Pregnant people with moderate/severe insomnia were predominantly on average 27.2 years old (SD: 5.7), non‐Hispanic/Latinx, White, and had a Bachelor's degree or higher (Table 1). Additionally, they more often reported tobacco smoking (44.6% vs. 37.8%), gestational diabetes (5.2% vs 3.8%), and pre‐pregnancy depression (13.9% vs. 7.8%). Among those with moderate/severe insomnia, the risk of PTD <37 weeks (aRR 0.69, 95% CI 0.53, 0.91) was decreased. The risks of IPD (aRR 0.88, 95% CI 0.65, 1.24), including PEC (aRR 0.98, 95% CI 0.64, 1.49) and FGR (aRR 0.72, 95% CI 0.45, 1.23) were not increased. Additional outcomes, by insomnia status, are described in Table 2.

**Conclusion**: Moderate/severe insomnia was not associated with increased risk for IPD or preterm birth in this large cohort of nulliparous individuals. These findings provide mechanistic insights, suggesting that insomnia related risks, if present, are not due to placental ischemia.

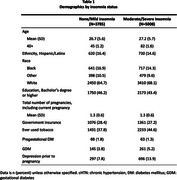

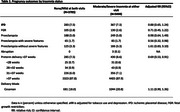

**3:30 PM – 5:00 PM**

## 611 | **From Delivery to Prevention: Building Bridges to Longterm Cardiometabolic Care**

Natalie E. Poliektov^1^; Hannah G. Steffke^2^; Olivia Carter^3^; Kaitlyn K. Stanhope^4^; Vasiliki Michopoulos^5^; Abigail Lott^5^; Elizabeth C. Rhodes^6^; Suchitra Chandrasekaran^1^

^1^Department of Gynecology and Obstetrics, Emory University School of Medicine, Atlanta, GA; ^2^Harvard Medical School, Boston, MA; ^3^Emory University School of Medicine, Atlanta, GA; ^4^Rollins School of Public Health, Rollins School of Public Health, GA; ^5^Department of Psychiatry, Emory University School of Medicine, Atlanta, GA; ^6^Rollins School of Public Health, Emory University, Atlanta, GA

**Objective**: The postpartum (PP) period is a key window to impact long‐term health, especially for those with cardiometabolic diseases in pregnancy who need intensified follow‐up. In 2022, we established a Postpartum Cardiometabolic Clinic (PPMC), offering counseling at 12 weeks PP to address health risks & coordinate referrals to primary (PCP) & specialty health care. While early pilot data showed improved PCP and cardiology follow‐up, we now assess referrals and lab monitoring at 1 year PP.

**Study Design**: We performed a retrospective cohort study of subjects referred to PPMC. Data including follow‐up with providers were compared between those who attended vs did not attend PPMC. Continuous and categorical variables were compared using Wilcoxon Rank Sum Tests and Chi‐Square/Fisher's exact tests as appropriate. Log binomial regressions calculating adjusted risk ratios (ARR) were performed.

**Results**: Of *N* = 382 referrals, 58% (*n* = 220) attended PPMC (Table 1). Those who attended vs did not attend PPMC were more likely to have follow‐up scheduled with PCP (59.6% vs 37.0%, *p* < 0.01), cardiology (56.8% vs. 25.9%, *p* < 0.01), & a trend with mental health providers (14.6% vs. 9.3%, *p* = 0.12) within 1 year PP. Those who attended PPMC were also more likely to have lipids (37.7% vs. 19.1%, *p* < 0.001), hemoglobin A1c (36.8% vs. 22.8%, *p* = 0.003), & 2‐hour glucose tolerance test drawn (9.1% vs. 1.2%, *p* = 0.001) (Table 2). Controlling for age, race, parity, education & insurance, those attending PPMC had a 1.4‐fold higher likelihood of PCP follow‐up (ARR 1.4, 95% CI 1.1–1.7, *p* = 0.006) & 2‐fold higher likelihood of cardiology follow‐up (ARR 2.0, 95% CI 1.5–2.7 *p* < 0.001).

**Conclusion**: Our findings underscore the need to shift the PP care model from a single visit to a longitudinal, multidisciplinary approach. A targeted counseling visit was associated with significant improvements in long‐term follow‐up, highlighting the powerful impact of individualized care in empowering patients & facilitating care transitions. PPMC represents a scalable, patient‐centered intervention to bridge care gaps & optimize long‐term cardiometabolic health.

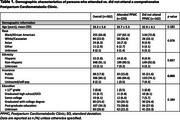

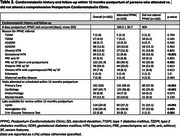

**3:30 PM – 5:00 PM**

## 612 | **Triple Threat: How Obesity, Chronic Hypertension, and Pregestational Diabetes Drive Severe Maternal Morbidity**

Nida Ali^1^; Bo Park^2^; Ashra Tugung^1^; Sergio A. Karageuzian^3^; Havilah Reimche‐Vu^4^; Kriti N. Vedhanayagam^5^; Ilish Gedestad^3^; Neville Tritch^4^; Kevin H. Hu^5^; Ruofan H. Yao^5^

^1^Loma Linda University Children's Health | Perinatal Institute, Loma Linda, CA; ^2^California State University, Fullerton, Fullerton, CA; ^3^Loma Linda University Health, Loma Linda, CA; ^4^Loma Linda University School of Medicine, Loma Linda, CA; ^5^Loma Linda University, Loma Linda, CA

**Objective**: To examine whether obesity, chronic hypertension (HTN), and pregestational diabetes (DM), independently and through interactive effects, are associated with risk of severe maternal morbidity (SMM).

**Study Design**: We conducted a population‐based, retrospective cohort study using the California Office of Statewide Health Planning and Development Linked Birth File with ICD‐9‐CM diagnoses from 2007–2012. Patients were grouped by the presence of obesity, HTN, and DM independently and interactively. Chi‐square tests determined associations with SMM, and cox proportional hazard analysis estimated SMM risk for each condition and their interactions, adjusting for maternal age, parity, prior cesareans, delivery year, and mode of delivery. A cumulative hazard graph illustrated gestational age at SMM occurrence. Hazard ratios (HRs) with 95% confidence intervals (CIs) were reported to indicate the magnitude and reliability of risks.

**Results**: Among 2.9 million deliveries, SMM incidence was 1.47% in women without these conditions. Obesity alone (*n* = 817,034) was not associated with increased risk (HR 0.97 [95% CI: 0.97–1.01]). HTN only (*n* = 6,128) (HR 6.47 [95% CI: 5.85–7.15]) and DM only (*n* = 9,961) (HR 2.36 [95% CI: 2.11–2.64]) were strong independent risk factors. When obesity interacted with HTN or DM, HRs were elevated but lower than HTN or DM alone: obesity+HTN (*n* = 7,648; HR 4.61 [95% CI: 4.16–5.10]) and obesity+DM (*n* = 13,764; HR 1.97 [95% CI: 1.79–2.18]). HTN+DM (*n* = 421) demonstrated the highest risk with an 11‐fold increase (HR 11.02 [95% CI: 8.08–15.02]), while the triple combination (*n* = 1,021) also markedly increased risk (HR 5.86 [95% CI: 4.59–7.48]).

**Conclusion**: Chronic HTN, especially when interacting with obesity or pregestational DM, greatly increases SMM risk, underscoring the need for targeted prenatal care. Obesity alone conferred a slightly lower risk.

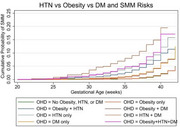

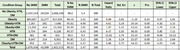

**3:30 PM – 5:00 PM**

## 613 | **Surgery‐to‐**Delivery **Interval: Critical Predictor of School Age Neurodevelopmental Functioning in Prenatally Repaired Myelomeningocele**

Nikan Zargarzadeh^1^; Claudio V. Schenone^2^; Eyal Krispin^2^; Jonathan Castillo^3^; Heidi Castillo^3^; Alireza A. Shamshirsaz^2^; Ashish Premkumar^4^

^1^Boston Children's Hospital, Boston, MA; ^2^Boston Children's Hospital, Harvard Medical School, Boston, MA; ^3^UNMC Division of Developmental Medicine, Omaha, NE; ^4^University of Chicago, Chicago, IL

**Objective**: To evaluate the association between the prenatal surgery‐to‐delivery interval—the time between prenatal fetal myelomeningocele (fMMC) repair and delivery—and long‐term neurodevelopmental outcomes, ascertained at school age.

**Study Design**: This was a secondary analysis of the prenatal fMMC repair arm of the Management of Myelomeningocele Study (MOMS) using data provided by the National Institutes of Health (NIH). The primary exposure was prenatal surgery‐to‐delivery interval, dichotomized as ≤62 days (lowest quartile) vs. >62–110 days. Outcomes included validated neurodevelopmental assessments, need for additional neurosurgical procedures, and independent walking. Bivariate analyses used Fisher's exact and Wilcoxon rank‐sum tests. Linear regression, adjusted for covariates (*p* < 0.05), assessed associations between exposure and Vineland scores, the primary MOMS2 outcome.

**Results**: Of 72 participants, 19 (26.4%) were in the shortest interval group and 53 (73.6%) in the longer interval group. At 10 years, children in the shorter interval group had lower standard scores in Vineland II communication (89.1 vs. 95.6, *p* = 0.03), daily living skills (82.7 vs. 88.7, *p* = 0.03), KBIT verbal (95 vs. 103, *p* = 0.03), reading performance (92.2 vs. 103, *p* = 0.006), and word generation (6.2 vs. 8.6, *p* < 0.001). Independent ambulation was also less frequent (6.7% vs. 35.1%, *p* = 0.05). After adjusting for key covariates and gestational age via residual analysis, the interval was no longer significantly associated with Vineland II scores or ambulation. However, in a model adjusting only for residual gestational age, a longer interval remained significantly associated with higher Vineland Adaptive Behavior Composite standard scores (adjusted coefficient: 0.11 [95% CI: 0.006, 0.22], *p* = 0.04).

**Conclusion**: Shorter intervals between prenatal fMMC repair and delivery are linked to worse cognitive and functional outcomes in certain domains at 10 years. While this association weakens after full covariate adjustment, it remains significant when adjusting only for gestational age—suggesting an independent effect of pregnancy duration.

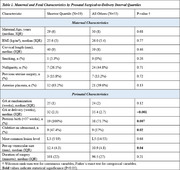

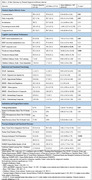

**3:30 PM – 5:00 PM**

## 614 | **Is Household** Income **Associated With 30‐Month Neurological Outcomes After Prenatal Fetal Myelomeningocele Repair?**

Nkechinyelum Ogu^1^; Abhinav Reddy^2^; Henry David^2^; Bahktiar Yamini^2^; Aimen Shaaban^3^; Jessica T. Fry^1^; Ashish Premkumar^2^

^1^Northwestern University Feinberg School of Medicine, Chicago, IL; ^2^University of Chicago, Chicago, IL; ^3^The Chicago Institute for Fetal Health (CIFH), Chicago, IL

**Objective**: To evaluate the relationship between household income at the time of prenatal myelomeningocele (MMC) repair and neurodevelopmental outcomes at 30 months of life.

**Study Design**: This was an unplanned secondary analysis of individuals undergoing prenatal MMC repair in the Management of Myelomeningocele Study (MOMS). Bivariate analyses were performed comparing families with household income above versus below $50,000 at the time of randomization, which corresponded to the median U.S. household income during the study period (2003–2010). The outcomes were: shunt or death by 1 year of life, difference between the motor function and anatomic level, Bayley Scales of Infant Development (BSID) scores, Peabody Developmental Motor Scales, WeeFIM scores, and ability to walk independently. Wilcoxon rank‐sum tests were used for continuous variables and Fisher's exact or chi‐squared tests for categorical variables. A *p*‐value <0.05 indicated statistical significance.

**Results**: Of 91 participants meeting inclusion criteria, 60 (66%) had a household income above $50,000. There were significant differences between groups in multiple sociodemographic markers, though no differences were seen for lesion level, lateral ventricular size, clubfoot, operative duration, and gestational age at delivery (Table 1). At 30 months, children in higher income households had better motor function (*p* = 0.04). They also had higher BSID mental (*p* = 0.03) and psychomotor scores (*p* = 0.01), as well as higher Peabody scores related to locomotion and object manipulation (*p* < 0.001). A similar pattern was noted for WeeFIM scores related to mobility (*p* = 0.03, Table 2).

**Conclusion**: Household income below $50,000 at the time of prenatal MMC repair is associated with worse functional and neurodevelopmental outcomes at 30 months of life despite similar lesion characteristics. Further research is required to develop novel interventions aimed at ameliorating socioeconomic disparities among people undergoing prenatal MMC repair to improve childhood health outcomes.

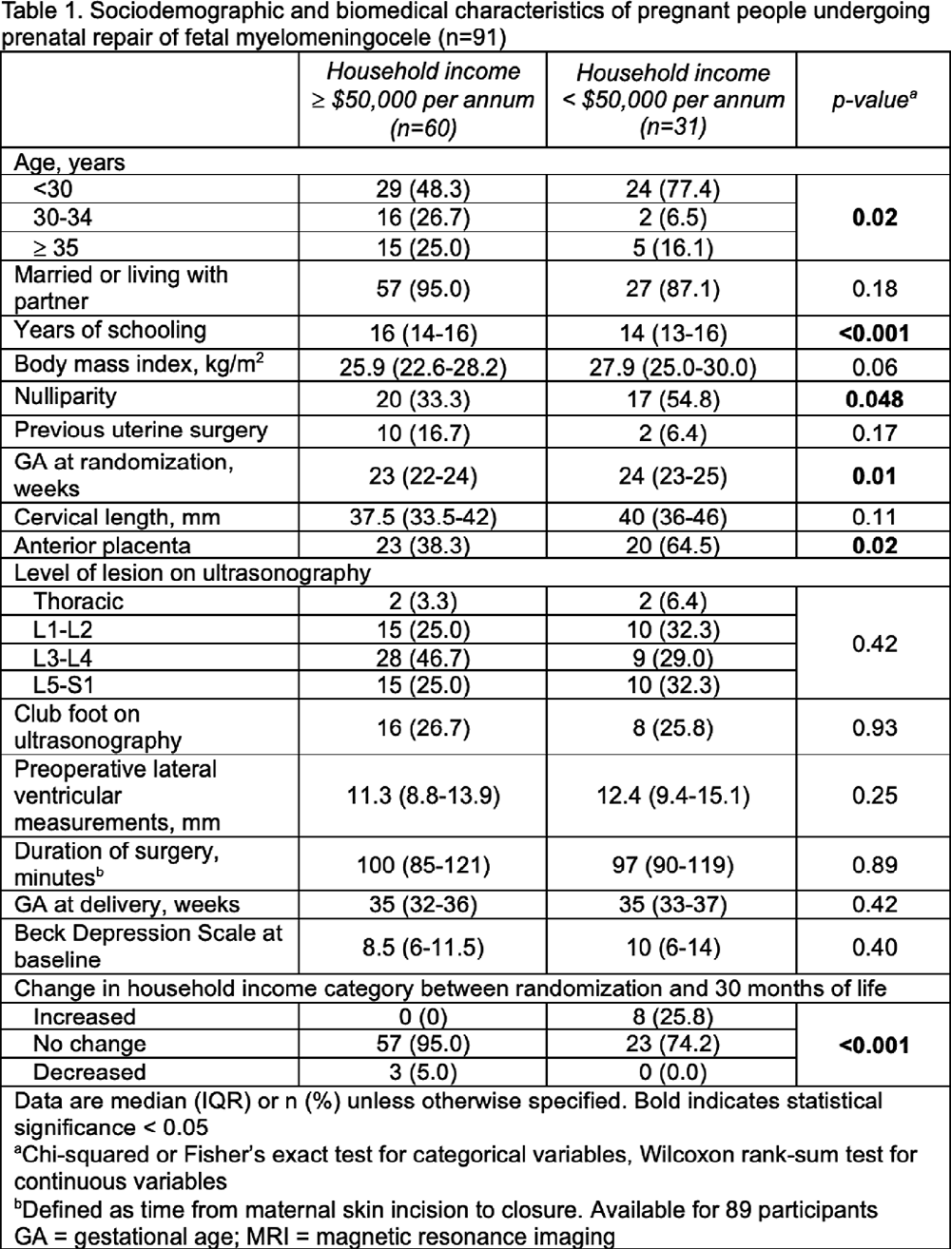

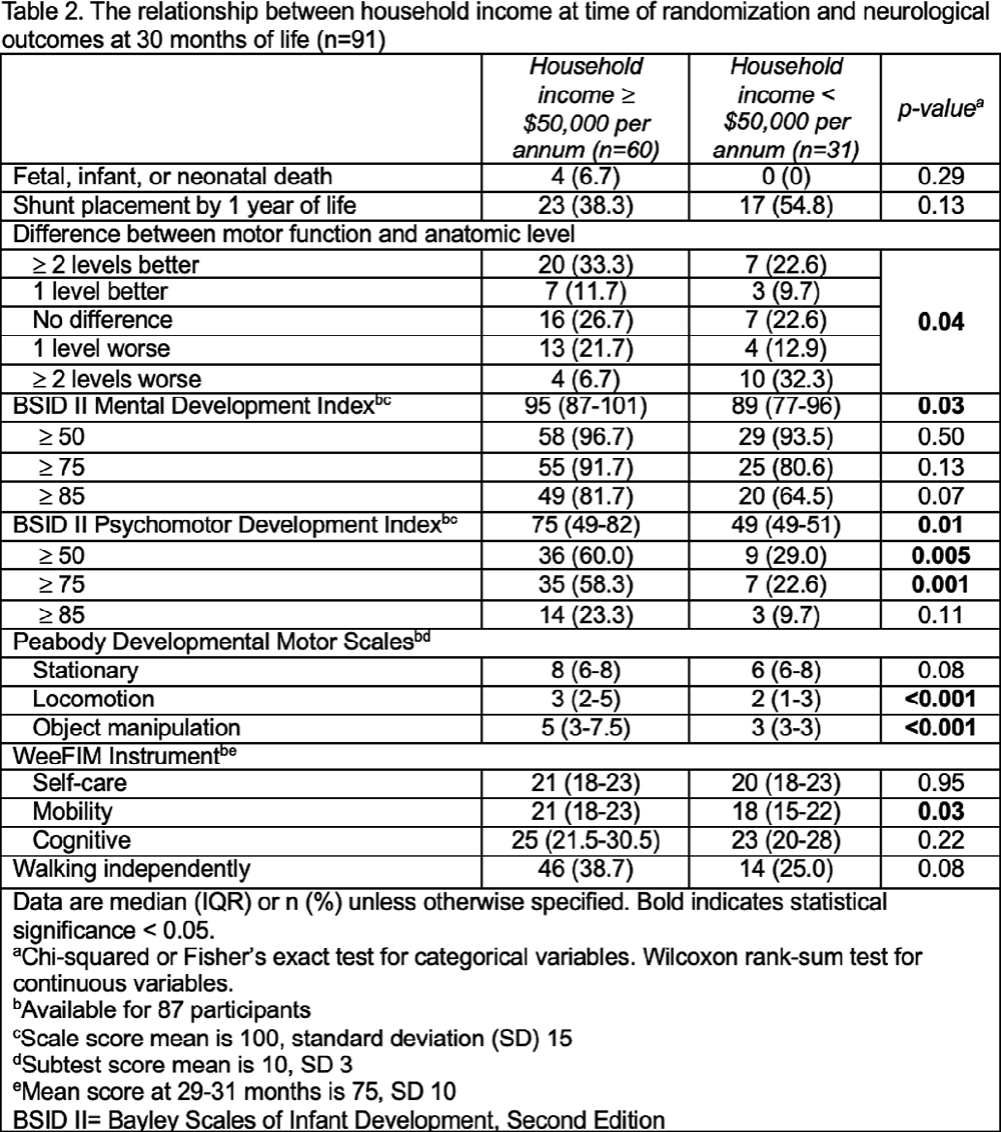

**3:30 PM – 5:00 PM**

## 615 | **Dynamic Fetal T** Cell **Responses to Viral Exposure Uncovered by Single‐cell Systems Immunology**

Noah w. Parker, N/A^1^; Jessian L. Munoz^2^; Myla C. Stanford^1^; Alexandra L. Hammerquist^1^; Michael D. Jochum, Jr.^3^; Brian A. Burnett^3^; Emma Kelling^1^; Daniel Arnaud Dominguez^1^; Luis E. Delgadillo Chabolla^1^; Ahmed A. Nassr^1^; Roopali V. Donepudi^1^; Lori Showalter^1^; Cynthia Shope^1^; Magdalena Sanz Cortes^1^; Enrico R. Barrozo^4^

^1^Baylor College of Medicine, Baylor College of Medicine, TX; ^2^Texas Children's Hospital, Texas Children's Hospital, TX; ^3^Baylor College of Medicine, Houston, TX; ^4^Baylor College of Medicine and Texas Children's Hospital, Houston, TX

**Objective**: Cytomegalovirus (CMV) is the leading cause of congenital infections, yet the timeline and specificity of fetal adaptive immune responses remain undefined. Prior studies have detected fetal T cell activation in CMV as early as 22 weeks, but lacked patient‐level evidence of antigen‐specific memory at early gestation. We hypothesized that clonal, CMV‐specific fetal T cell memory responses can emerge before 22 weeks gestation and sought to test this by leveraging high‐resolution single‐cell analyses.

**Study Design**: We collected fetal specimens from intrauterine transfusions (IUT; *n* = 3) and neural tube defect repair (NTD; *n* = 12) surgeries and used a single‐cell multi‐omics approach to compare 2 transplacental CMV infections with 12 cases lacking exposure histories. Downstream analyses were conducted with Seurat, clusterProfiler, and scRepertoire for T cell receptor (TCR) profiling.

**Results**: Corroborating serological and histopathological clinical diagnoses, CMV gene expression was detected in 26 cells from the two CMV‐infected patients, with 52% from monocyte lineages (Figure 1a). Comparing CMV^+^ and CMV^−^ cells from 166,107 single‐cell transcriptomes to a reference of 1,075,352 cells (CZI CellxGene) identified 2535 differentially expressed genes (DEGs; log2FC >1.4, FDR <0.05). Pathway analysis revealed 720 significant GO enrichment pathways, highlighted by hundreds of genes in innate, myeloid, and leukocyte pathways (Figure 1b‐c; FDR <0.05). A focused set of 16,515 cells with paired VDJ sequences revealed 12 unique clusters, including distinct CD8⁺ T cell populations (Figure 2a). TCR mapping detected clonotypes matching known CMV‐specific epitopes (UL29, pp65, IE1; Figure 2b) and CMV‐specific clonal expansion. Gene expression analysis of CMV‐specific clonotypes showed limited Th1 responses.

**Conclusion**: These findings set a new benchmark for fetal immune memory, revealing antigen‐specific T cell competence at least three weeks earlier than previously recognized and informing earlier intervention strategies for congenital infections.

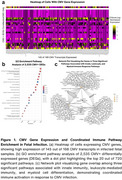

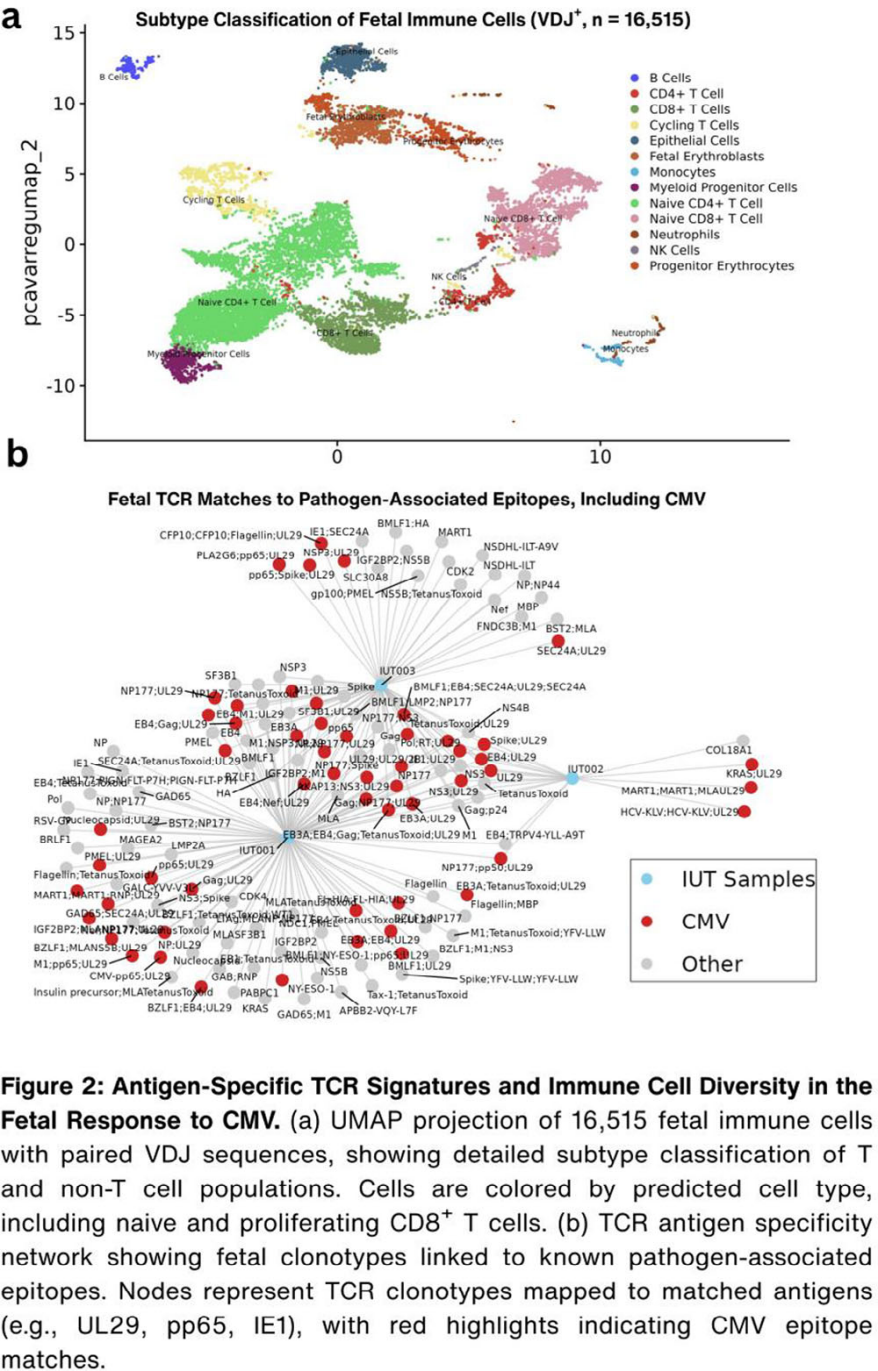

**3:30 PM – 5:00 PM**

## 616 | **Prognostic Value and Progression of Umbilical Artery Doppler in Dichorionic Twin Pregnancies With Early‐Onset FGR**

Noam Pardo^1^; John C. Kingdom, MD^2^; Liran Hiersch^3^; Nir Melamed^4^

^1^Sunnybrook Health Sciences Centre, Toronto, ON; ^2^Maternal Fetal Medicine Division, Department of Obstetrics and Gynaecology, Mount Sinai Hospital, University of Toronto, Toronto, Ontario, Canada, Toronto, ON; ^3^Lis Hospital for Women's Health, Tel Aviv Sourasky Medical Center Gray Faculty of Medicine, Tel Aviv University, Israel, ISRAEL, Tel Aviv; ^4^Sunnybrook Health Sciences Center, Toronto, ON

**Objective**: The mechanisms underlying fetal growth restriction (FGR) in twin pregnancies may differ from those in singletons. These differences may affect the relationship between placental insufficiency and umbilical artery (UA) Doppler. In the current study, we aimed to compare the rate of UA Doppler progression and time to delivery between dichorionic twin and singleton pregnancies with severe early‐onset FGR.

**Study Design**: We conducted a retrospective study of dichorionic twins and singletons with severe early‐onset FGR, defined as FGR diagnosed <32 weeks, accompanied by absent or reversed end‐diastolic flow (AREDF) in the UA. UA Doppler changes were classified as elevated UA‐PI, intermittent or persistent absent end‐diastolic flow (iAEDF or AEDF), and intermittent or persistent reversed end‐diastolic flow (iREDF or REDF).

**Results**: A total of 46 dichorionic twin pregnancies and 241 singleton pregnancies (701 and 1835 ultrasound exams, respectively) were analyzed. Twins were characterized by a higher gestational age at diagnosis with AREDF (28.6 ± 3.7 vs. 27.5 ± 3.2 weeks, *p* = 0.032) and at birth (31.2 ± 2.2 vs. 28.6 ± 3.1 weeks, *p* < 0.001) (Figure 1), and a lower rate of preeclampsia (8.7% vs. 24.1%, *p* = 0.02). Doppler progression was overall slower in twins than in singletons. For example, the mean time (in days) of progression from elevated UA‐PI to iAEDF, iREDF, and REDF was 14 ± 16 vs. 7 ± 8 (*p* = 0.025), 21 ± 20 vs. 9 ± 8 (*p* = 0.007), and 25 ± 3 vs. 8 ± 8 (*p* = 0.007), respectively. Twins were also characterized by a longer interval to delivery compared with singletons following elevated UA‐PI (median 16 [IQR 6–26] vs. 6 [3–12] days, *p* < 0.001) and iAEDF (11 [5–17] vs. 4 [2–11] days, *p* = 0.001) (Figure 2).

**Conclusion**: In pregnancies complicated by severe early‐onset FGR with AREDF, dichorionic pregnancies are characterized by a later onset of AREDF, a slower progression rate of UA Doppler findings, and a longer interval from UA Doppler abnormalities to delivery. This information may be useful for patient counseling and can inform fetal surveillance in these high‐risk pregnancies.

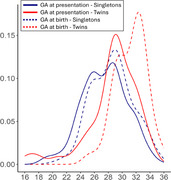

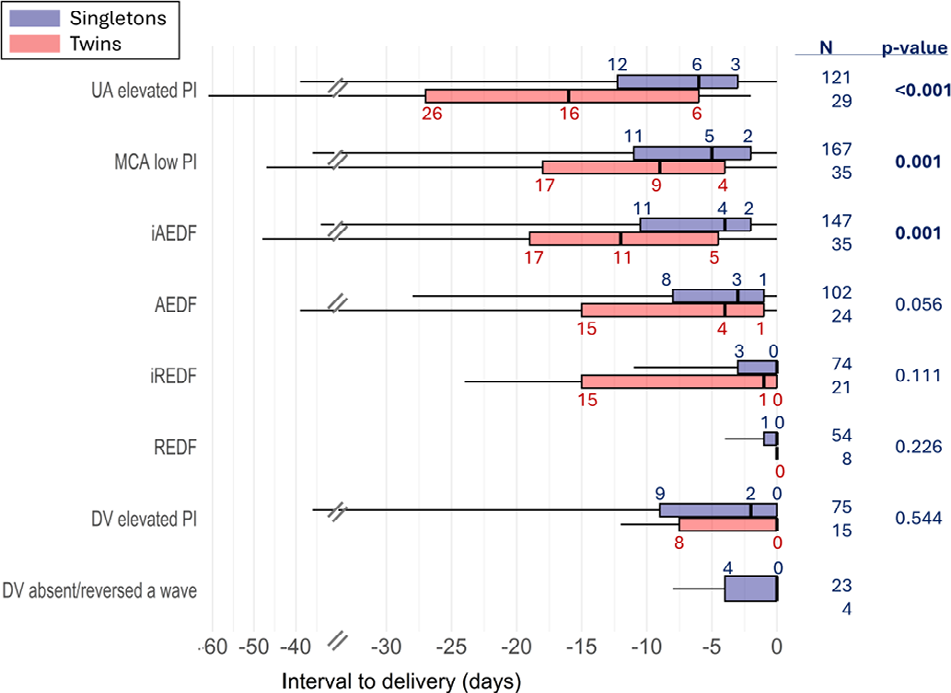

**3:30 PM – 5:00 PM**

## 617 | **Long‐Term** Gastrointestinal **Morbidity in Term‐Born Offspring Following Antenatal Corticosteroids Exposure Before 34 Weeks of Gestation**

Noam Shema^1^; Tamar Wainstock^2^; Eyal Sheiner^3^

^1^Department of Obstetrics and Gynecology, Soroka University Medical Center, Ben‐Gurion University, Beer sheva, HaDarom; ^2^Department of Epidemiology, Biostatistics and Community Health Sciences, Faculty of Health Sciences, Ben‐Gurion University of the Negev, Beer Sheva, HaDarom; ^3^Soroka University Medical Center, Faculty of Health Sciences, Ben‐Gurion University of the Negev, beer sheva, HaDarom

**Objective**: Antenatal corticosteroids (ACS) are routinely administered to women at risk for preterm birth to reduce neonatal morbidity. While their short‐term benefits are well‐established, some exposed pregnancies continue to term, raising concerns about potential long‐term effects in this group. This study aimed to evaluate whether ACS exposure before 34 weeks is associated with long‐term gastrointestinal morbidity in children born at term.

**Study Design**: A population‐based study was conducted at a tertiary medical center, including all singleton term deliveries (≥37 weeks) from 1991 to 2021. Data were linked to community and hospital medical records. Neonates were categorized based on ACS exposure before 34 weeks of gestation. The primary outcome was long‐term gastrointestinal morbidity up to 18 years of age. Time‐to‐event analysis was performed using Kaplan‐Meier curves and Cox proportional hazards models adjusted for confounders.

**Results**: Among 182,625 singleton term births, 2093 (1.1%) were exposed to ACS before 34 weeks of gestation. The incidence of gastrointestinal morbidity was higher in the exposed group (66.2 vs. 35.2 per 1000 person‐years; Table, Kaplan Meier log‐rank *p* < 0.001, Figure). After adjusting for confounders using a Cox regression model, controlling for maternal age, gestational age, ethnicity, diabetes, hypertensive disorders and caesarian delivery, the association remained significant (adjusted HR = 1.13; 95% CI, 1.01–1.26; *p* = 0.029). The results remained significant in a sub‐analysis for early term (adjusted HR = 1.19, *p* < 0.001).

**Conclusion**: Among infants born at term, ACS exposure before 34 weeks of gestation was associated with a modest but statistically significant increase in long‐term gastrointestinal morbidity. Given this observed association, further research is warranted to clarify the potential long‐term risks. These findings support the need for more nuanced counseling with patients at risk of preterm birth, particularly when the likelihood of reaching term remains uncertain.

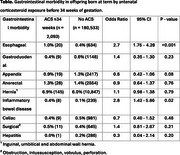

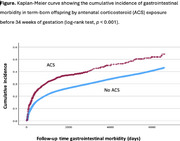

**3:30 PM – 5:00 PM**

## 618 | **Unequal Access, Unequal Outcomes: Food Deserts and Preterm Birth Rates**

Noor K. Al‐Shibli^1^; Elizabeth C. Rhodes^2^; Erin Ferranti^3^; Anne L. Dunlop^4^; Suchitra Chandrasekaran^5^

^1^Emory University School of Medicine, Emory University School of Medicine, GA; ^2^Rollins School of Public Health, Emory University, Atlanta, GA; ^3^Nell Hodgson Woodruff School of Nursing, Dept of Gynecology & Obstetrics, Emory University, Nell Hodgson Woodruff School of Nursing, Dept of Gynecology & Obstetrics, Emory University/ Atlanta, GA; ^4^Department of Gynecology and Obstetrics, Division of Research, Emory University School of Medicine, Dept of Gynecology & Obstetrics, Division of Research, Emory University School of Medicine/ Atlanta, GA; ^5^Department of Gynecology and Obstetrics, Emory University School of Medicine, Atlanta, GA

**Objective**: Social disparities are known contributors to preterm birth (PTB). Food deserts (FD), regions with limited access to nutrition/affordable food, reflect broader social, physical and emotional inequities that may adversely impact maternal and neonatal outcomes. Hence, we sought to assess the relationship between living in a FD and PTB.

**Study Design**: Using our institutional obstetric database, we performed a retrospective cohort study of all deliveries with documented zip‐code and clinical/demographic data. FD status was assigned by zip‐code of primary residence according to the U.S. Department of Agriculture (USDA) definitions. PTB was defined as delivery <37 weeks of gestational age (GA). Kruskal‐Wallis and Chi‐square tests were performed to compare continuous and categorical variables, respectively. A Poisson regression, adjusted for maternal age, race, and body mass index, was used to estimate incidence risk ratios (IRR).

**Results**: Among *N* = 20,925 pregnancies, 7107 (34.0%) lived in a FD. Mean GA at delivery was lower in the FD group vs those not in a FD (37.9 ± 2.8 vs 38.2 ± 2.7, *p* < 0.01). 14.6% (*n* = 3,080) of the cohort experienced a PTB. Those in a FD had significantly higher rates of PTB (15.3% vs 14.2%, *p* = 0.026) and lower neonatal birthweight (3050 ± 621g vs 3110 ± 617g, *p* < 0.001). However, there was no difference in mode of delivery by FD status (Table 1). Our data noted those who live in an FD have a 1.1‐fold higher risk of experiencing a PTB (IRR 1.13, 95% CI 1.02–1.24, *p* < 0.01).

**Conclusion**: Our data innovatively demonstrate the need to understand the impacts of living in an FD on pregnancy outcomes including PTB. These findings highlight the need to consider geographic and nutritional access factors when designing strategies to reduce PTB.

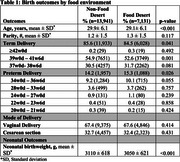

**3:30 PM – 5:00 PM**

## 619 | **Association of** Prolonged **Labor With Infectious Outcomes in Nulliparous Women With Isolated Intrapartum Fever**

Ohad Houri^1^; Liel Mharian^2^; Mor Ginat Shaked^3^; Or Bercovich^4^; Yarin Mash^3^; Asnat Walfisch^5^; Asaf Romano^6^; Yossi Geron^6^; Natav Hendin^7^

^1^BCNatal Fetal Medicine Research Center, BARCELONA, Catalonia; ^2^Meir Medical Center, Kfar Saba, HaMerkaz; ^3^Rabin medical center, Petah Tikva, HaMerkaz; ^4^Rabin Medical Center, petha tiqwa, HaMerkaz; ^5^Rabin Medical Center, Petach Tikva, HaMerkaz; ^6^Helen Schneider Hospital for Women, Rabin Medical Center, Petah Tikva, HaMerkaz; ^7^Rabin Medical Center, Petah Tikva, HaMerkaz

**Objective**: To evaluate the association between total labor duration and maternal or neonatal infectious outcomes among nulliparous women with isolated intrapartum fever.

**Study Design**: We conducted a retrospective cohort study of nulliparous women with singleton gestations and isolated intrapartum fever (≥37.8°C, without clinical chorioamnionitis) who reached the second stage of labor. All women delivered at a single tertiary center between 2012 to 2024. For all patients, urine and blood cultures were obtained at the time of fever diagnosis, and placental cultures were collected at delivery. Blood cultures and pediatric assesment were obtained from all neonates upon deliver. Patients were stratified by total labor duration: <12 hours (*n* = 505), 12–18 hours (*n* = 319), and >18 hours (*n* = 192). Outcomes were compared using chi‐square or Fisher's exact tests for categorical variables and Kruskal‐Wallis test for continuous variables. Composite maternal culture positivity was defined as a positive placental, urine, or blood culture.

**Results**: Out of 1710 deliveries, a total of 1016 women were included. The composite maternal culture positivity rate was similar across groups (11.1% vs. 11.0% vs. 15.6%, *p* = 0.13), as were individual rates of bacteremia (3.2% vs. 2.2% vs. 4.2%, *p* = 0.31). However, urine culture positivity increased with longer labor (4.2% vs. 6.9% vs. 8.3%, *p* = 0.045). Maximum postpartum fever was slightly higher in shorter labor groups (*p* = 0.024), and postpartum stay was unchanged (median 3 days across all groups, *p* = 0.09). Neonatal sepsis, NICU admission, and antibiotic use were not associated with labor duration. In contrast, Apgar scores were lower in the >18 h group: 1‐min Apgar <7 (15.1% vs. 6.5–7.8%, *p* < 0.01) and 5‐min Apgar <7 (6.2% vs. 1.3–2.8%, *p* = 0.04).

**Conclusion**: Prolonged labor in nulliparous women with isolated intrapartum fever was not associated with an increased rate of maternal or neonatal infection. However, it was associated with significantly lower Apgar scores, suggesting potential neonatal compromise in this subgroup.

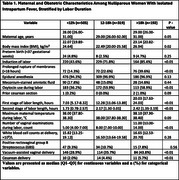

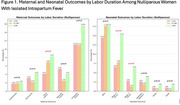

**3:30 PM – 5:00 PM**

## 620 | **Divergent** Maternal**–Fetal Immune Signatures After SARS‐CoV‐2 Vaccination Versus Infection: High‐Dimensional Cytometry Across the Placenta**

Ola Gutzeit^1^; Roee Iluz^2^; Michael G. Ross^3^; Nizar Khatib^4^; Yuval Ginsberg^2^; Zeev Weiner^5^; Ron Beloosesky^6^

^1^Rambam Health Care Campus, Haifa, Hefa; ^2^Rambam, Israel, Hefa; ^3^Harbor‐UCLA Medical Center, The Lundquist Institute at Harbor‐UCLA Medical Center, CA; ^4^Rambam Health Care Campus, haifa, Hefa; ^5^Rambam Health Care campus, Haifa, Hefa; ^6^Rambam Health Care Campus, Rambam Health Care Campus, Hefa

**Objective**: To compare maternal and neonatal immune profiles following SARS‐CoV‐2vaccination versus natural infection during pregnancy using high‐dimensional cytometry.

**Study Design**: We conducted a prospective cohort study at a single tertiary center. Peripheral and cord blood samples were collected at delivery from three groups: unvaccinated controls, pregnant individuals vaccinated with mRNA‐COVID‐19 vaccine, and individuals with resolved mild SARS‐CoV‐2 infection 4–8 weeks prior. Samples were profiled using a 30‐marker CyTOF panel. Immune populations were clustered using FlowSOM to identify 50 metaclusters. Statistical comparisons were performed using ANOVA/Tukey or Kruskal–Wallis/Dunn tests, and UMAP was used for dimensionality reduction.

**Results**: Maternal SARS‐CoV‐2 infection led to broad immune suppression: neutrophils decreased (49%→28%), classical monocytes (28%→3%), Th17‐like CD4⁺ (MC46, *p* < 0.05), and central memory CD4⁺ T cells (MC48, *p* < 0.01) were reduced. CD8⁺ T cells were also affected, with MAIT precursor/memory (MC19, *p* < 0.001) and naïve CD8⁺ T cells (MC29, *p* < 0.01) significantly reduced. Naïve CD4⁺ T cells (MC49) were decreased in both mothers and neonates. Neonates of infected mothers showed neutrophil loss (69%→36%), increased immature NK cells (2%→7%), naïve B cells, and CD8⁺ TEMRA cells (0.11%→0.14%, *p* = 0.01). Naïve B cells (MC8) diverged—decreased in mothers but increased in neonates (5%→14%, *p* = 0.024). In contrast, vaccinated dyads (mothers and neonates) retained immune profiles comparable to uninfected controls across major leukocyte subsets.

**Conclusion**: SARS‐CoV‐2 infection during pregnancy induces systemic maternal immune suppression, particularly affecting monocytes and adaptive T cell subsets, with parallel expansion of naïve and immature lymphoid populations in the fetus. In contrast, mRNA vaccination preserves immune balance across the maternal–fetal interface.

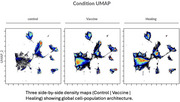

**3:30 PM – 5:00 PM**

## 621 | **Racial and Ethnic Disparities in Maternal‐Newborn Dyadic Birth Outcomes: Insights From Door Methodology**

Oladunni Ogundipe; Frank I. Jackson; Sarah H. Abelman; Matthew J. Blitz

Northwell, New Hyde Park, NY

**Objective**: To evaluate racial and ethnic disparities in maternal‐newborn dyadic outcomes among nulliparous, term, singleton, vertex (NTSV) patients using the desirability of outcome ranking (DOOR) methodology. Specifically, we assessed how the distribution of outcomes, ranked from most to least desirable, differed across racial and ethnic groups.

**Study Design**: This retrospective cross‐sectional study included all NTSV deliveries at seven hospitals within a large New York health system from 2019–2024. Pregnancies that were ineligible for PC‐06 and those with unknown race and ethnicity were excluded. Race and ethnicity were self‐reported by patients using predefined categories. Birth outcomes were classified using DOOR methodology into four groups based on delivery mode and presence or absence of maternal or neonatal complications: (1) uncomplicated vaginal delivery, (2) uncomplicated cesarean delivery, (3) vaginal delivery with complications, and (4) cesarean delivery with complications. Maternal complications were defined using CDC severe maternal morbidity (SMM) indicators, while neonatal complications were defined per the Joint Commission's PC‐06 criteria for unexpected complications in term newborns. Multinomial logistic regression modeled the association between race‐ethnicity group and birth outcome, adjusting for relevant covariates.

**Results**: A total of 47,188 NTSV patients were included. Non‐Hispanic White patients had the highest rate of uncomplicated vaginal delivery (70.4%) and the lowest rate of cesarean delivery with complications (2.4%). In contrast, non‐Hispanic Black patients had the lowest rate of uncomplicated vaginal delivery (62.6%) and the highest rate of cesarean delivery with complications (4.3%). Birth outcomes by group are shown in the Figure. After covariate adjustment, most racial and ethnic groups had higher odds of less desirable outcomes than non‐Hispanic White patients (Table 1).

**Conclusion**: DOOR methodology is a useful tool for describing racial and ethnic disparities in birth outcomes, providing more information than cesarean rates alone.

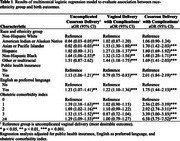

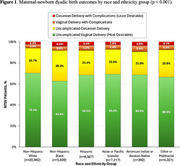

**3:30 PM – 5:00 PM**

## 622 | **Risk Factors** Associated **With Readmission After Cesarean Delivery in the Setting of Intra‐Amniotic Infection**

Osinakachukwu Mbata^1^; Lillian Boettcher^2^; Hannah Kelly^1^; Jaye Boissiere^1^; Sally Kuehn^1^; Jeffrey A. Kuller^1^; Sarah K. Dotters‐Katz^3^

^1^Duke University School of Medicine, Durham, NC; ^2^Department of Obstetrics and Gynecology, Duke University School of Medicine, Duke University School of Medicine/Durham, NC; ^3^Division of Maternal Fetal Medicine, Department of Obstetrics and Gynecology, Duke University, Duke University/Durham, NC

**Objective**: National safety and quality efforts aim to reduce the rates of postpartum readmissions for delivering and postpartum individuals. We sought to further clarify differences in risk factors for readmission as well as identify possible modifiable risk factors to further aid in reaching this goal within our population of interest.

**Study Design**: This was an IRB approved, retrospective cohort study of patients undergoing cesarean with intraamniotic infection (IAI) in a single healthcare system from 4/1/2013 to 11/2/2019. Patients missing data regarding delivery, antibiotic use, or IAI diagnosis were excluded. The primary outcomes of interest were factors associated with readmission among this population. We used bivariate tests and multivariate logistic regression for comparisons.

**Results**: Of the 985 patients who met inclusion criteria, 40 (4.1%) were readmitted. Differences in patient age, race, diabetes status, BMI, penicillin allergy status, and length of membrane rupture were observed (Table 1). Readmission for patients who delivered via cesarean section in the setting of IAI was significantly associated with prolonged rupture of membranes (aOR 2.4; *p* < 0.05) and penicillin allergy (aOR 4.36; *p* < 0.05). Azithromycin administration was protective (aOR 0.30; *p* < 0.05) (Table 2).

**Conclusion**: Differences by race/ethnicity as well as BMI and pre‐existing conditions exist for women delivering via CS in the setting of IAI. Prolonged rupture of membrane and penicillin allergy were significantly associated with readmission. These findings further support the evidence for predelivery optimization with consideration of allergy testing antenatally.

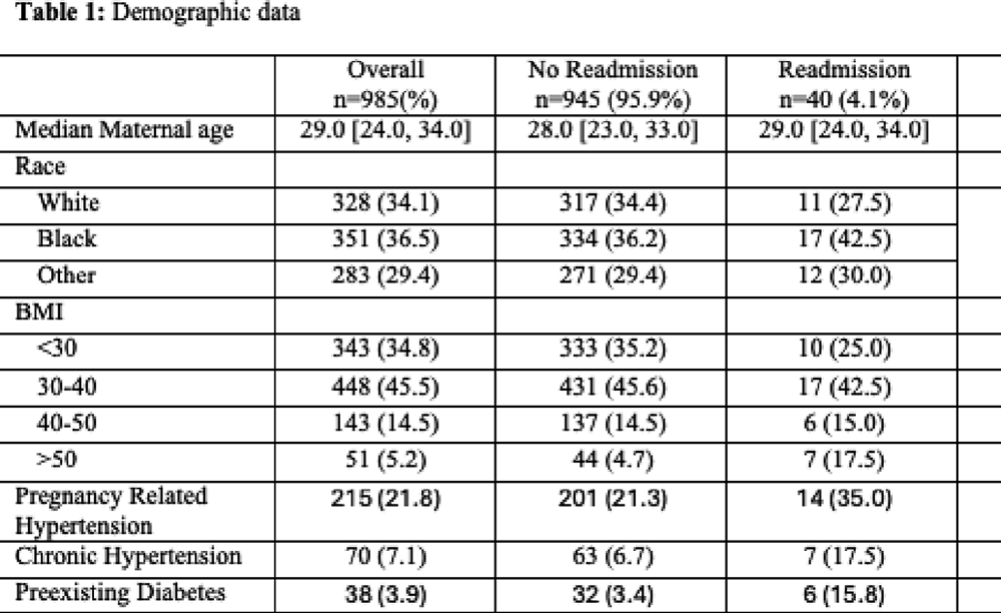

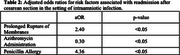

**3:30 PM – 5:00 PM**

## 623 | **Comparative** Effectiveness **of Two Enhanced Prenatal Care Models on Prenatal Care Experience**

Patience A. Afulani^1^; Daisy León‐Martínez^2^; Bethany Simard^3^; Cinthia Blat^4^; Venise Curry^5^; Kristin Carraway^5^; Kimberly Coleman‐Phox^4^; Brittany D. Chambers Butcher^6^; Bridgette Blebu^7^; Jennifer N. Felder^8^; Deborah Karasek^9^; Mary A. Garza^5^; Martha A. Tesfalul^4^; Christopher Downer^10^; Charles E. McCulloch^11^; Miriam Kuppermann^12^

^1^Department of Obstetrics, Gynecology and Reproductive Sciences, University of California, San Francisco, Universiy of California San Francisco, CA; ^2^Division of Maternal‐Fetal Medicine at the University of California San Francisco, University of California San Francisco, CA; ^3^Department of Obstetrics, Gynecology and Reproductive Sciences, University of California, San Francisco, UCSF, CA; ^4^Department of Obstetrics, Gynecology and Reproductive Sciences, University of California, San Francisco, San Francisco, CA; ^5^Central Valley Health Policy Institute at California State University, Fresno, Fresno, CA; ^6^Department of Human Ecology, College of Agricultural and Environmental Sciences, University of California, Davis, Davis, CA; ^7^Lundquist Institute for Biomedical Innovation ‐ Harbor‐UCLA, Los Angeles, CA; ^8^Department of Psychiatry and Behavioral Sciences, University of California, San Francisco, San Francisco, CA; ^9^Oregon Health & Science University‐Portland State University School of Public Health, Portland, OR; ^10^Medical Education Program, University of California, San Francisco, Fresno, Fresno, CA; ^11^University of California, San Francisco, University of California San Francisco, CA; ^12^Department of Obstetrics, Gynecology and Reproductive Sciences, University of California, San Francisco, University of California San Francisco, CA

**Objective**: To compare the effect of enhanced group (eGPC) and enhanced individual prenatal care (eIPC) models on prenatal care experience.

**Study Design**: Engaging Mothers and Babies; Reimagining Antenatal Care for Everyone (EMBRACE) was a pragmatic, two‐arm, randomized trial comparing the effect of eGPC vs eIPC on depression (primary), experience of care (secondary), and preterm birth (exploratory) among Medicaid‐eligible pregnant people in California's San Joaquin Valley. The primary outcome of this analysis was Person‐Centered Prenatal Care (PCPC), measured with the PCPC‐US scale. Secondary outcomes of care experience were measured with the Mothers on Respect index (MORi) and the Prenatal Care Satisfaction (PCS) scale, all completed at 30–34 weeks gestation. Summative scores were generated for all scales and standardized to range from 0 to 100, with higher scores indicating better care experience. We used an intention‐to‐treat approach in the primary analysis; subgroup analyses were conducted by prenatal care attendance (≥1 visit, ≥4 visits) and self‐reported race and ethnicity (Black, Latine). Analyses included mean differences in scores and restricted maximum likelihood mixed linear regression models adjusted for language of interview with random intercepts for provider. The analytic sample includes 526 participants (*n* = 223 eGPC, *n* = 303 eIPC).

**Results**: Most participants identified as Latine (72.2%). Prenatal care experience scores were generally high for both groups, with PCPC scores of 92.8 ± 0.91 for eGPC participants and 90.9 ± 0.81 for eIPC participants. Although eGPC participants had a slightly better experience than eIPC participants, differences did not reach statistical significance, with adjusted mean differences in scores for participants randomized to eGPC versus eIPC at 1.96 (CI:–0.16, 4.08), 0.39 (CI:–1.63, 2.41), 2.68 (CI:–0.72, 6.09) for PCPC, MORi, and PCS, respectively. The subgroup analysis yielded similar findings.

**Conclusion**: The findings suggest that both enhanced prenatal models may result in favorable care experiences for low‐income populations.

**3:30 PM – 5:00 PM**

## 624 | **Maternal** Cannabis **Exposure and Sleep Quality**

Pooja S. Parameshwar^1^; Amanda A. Allshouse^1^; Gwen McMillin^2^; Robert M. Silver^3^; Torri D. Metz^1^

^1^University of Utah, Salt Lake City, UT; ^2^University of Utah, University of Utah, UT; ^3^Department of Obstetrics and Gynecology, University of Utah Health, Salt Lake City, UT

**Objective**: To investigate the relationship between maternal cannabis exposure in pregnancy and measures of sleep quality.

**Study Design**: This was a secondary analysis of Nulliparous Pregnancy Outcomes Study: Monitoring Mothers‐to‐Be (NuMoM2b), a multi‐center prospective observational cohort study of patients enrolled from 2010 to 2013. We included participants who completed the WHI (Women's Health Initiative Insomnia Rating Scale) and had a urine specimen available from the first study visit (6w0d–13w6d). The exposure was cannabis use in early pregnancy, measured by 11‐nor‐9‐carboxy‐tetrahydrocannabinol (THC‐COOH) detected by liquid chromatography mass spectrometry urine assay (detected if ≥15 ng/mL). The primary outcome was clinically significant insomnia, defined as a WHI score greater than or equal to 9. The secondary outcome, excessive daytime sleepiness, was defined as an ESS score (Epworth Sleepiness Scale) greater than or equal to 11. Primary and secondary outcomes were summarized between groups with differences tested using chi‐square analyses. Adjusted odds ratios (aOR) for each outcome with THC exposure (versus without) were reported from multivariable logistic regression models.

**Results**: Of 8163 individuals included for analysis, 482 (5.9%) had cannabis exposure. Cannabis exposure was significantly associated with age younger than 30, obesity, not having daytime employment, low social support, a positive depression screen, moderate to high perceived stress or anxiety, and cotinine from urine toxicology (Table 1). In multivariable modeling, there was no association between cannabis exposure and clinically significant insomnia (50.4 vs 41.0%; aOR 1.12 95% CI 0.91–1.37, Table 2) or excessive daytime sleepiness (26.3 vs 24.3%; aOR 0.94 95% CI 0.75–1.18, Table 2).

**Conclusion**: Maternal cannabis exposure in early pregnancy was not associated with any standardized measures of sleep quality. Previously observed adverse pregnancy outcomes associated with maternal cannabis are likely mediated by mechanisms other than poor sleep quality.

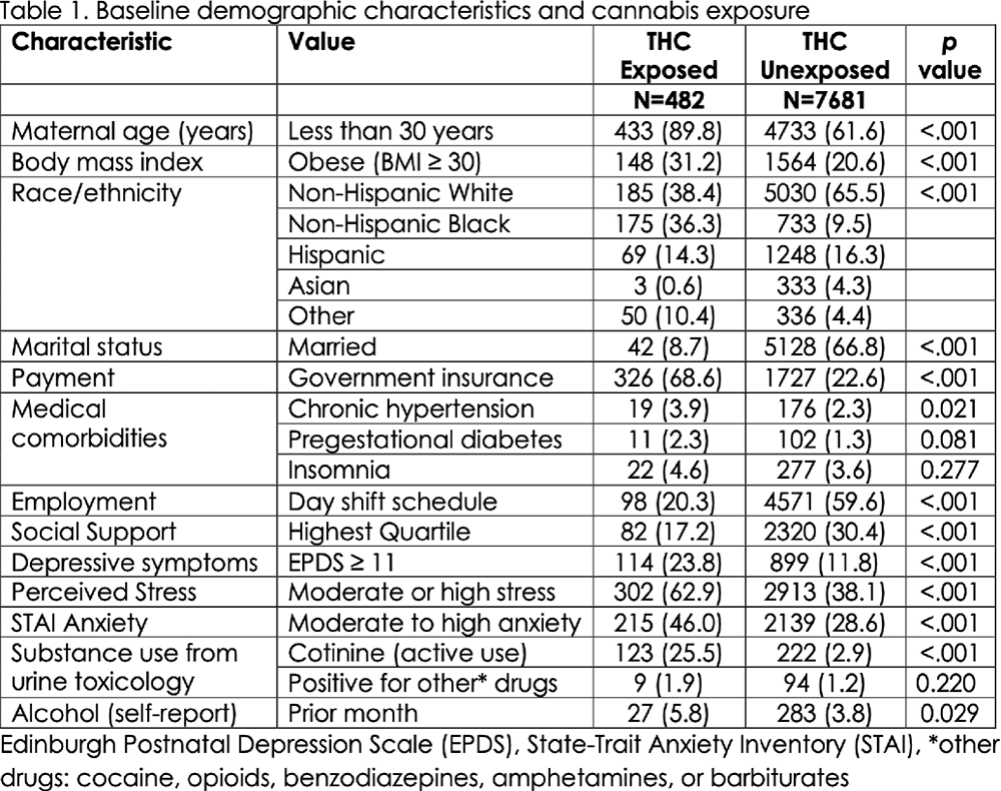

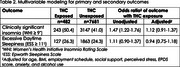

**3:30 PM – 5:00 PM**

## 625 | **Adverse** Perinatal **Outcomes Among Term Pregnancies Complicated by Chorioamnionitis**

Prakrunya Subhasree Badrinarayan^1^; Bharti Garg^2^; Amelia H. Gagliuso^3^; Isha Garg^2^; Irina Cassimatis^2^; Aaron B. Caughey^2^

^1^Oregon Health & Science University, Portland, OR; ^2^Oregon Health & Science University, Oregon Health & Science University, OR; ^3^Oregon Health and Science University, Portland, OR

**Objective**: Chorioamnionitis complicates 1–5% of U.S. births annually and carries substantial perinatal risks. Although timely diagnosis and antibiotic administration have reduced morbidity, chorioamnionitis remains independently associated with adverse outcomes. We evaluated the association between chorioamnionitis and adverse perinatal outcomes among term pregnancies in a modern cohort.

**Study Design**: We conducted a retrospective cohort study using California's linked vital statistics and hospital discharge data for deliveries between 2016 and 2020. We included singleton, non‐anomalous pregnancies delivered between 37 and 42 weeks. We excluded malpresentation, vasa previa, placenta previa, accreta, and planned cesarean deliveries. Chorioamnionitis and perinatal outcomes were identified using ICD‐9 and ICD‐10 codes. We used Chi‐square tests and multivariable Poisson regression models.

**Results**: Among a total 1,296,336 term births, 4.15% (*n* = 53,815) had chorioamnionitis. Compared to those without chorioamnionitis, affected individuals had higher risk of severe maternal morbidity (5.0% vs 1.0% [aRR = 4.14; 95% CI 3.96–4.33]), postpartum hemorrhage (13.1% vs 4.3% [aRR = 2.64; 95% CI 2.57–2.70]), sepsis (1.6% vs 0.1% [aRR = 28.93; 95% CI 28.47–32.86]), and ICU admission (0.4% vs 0.1% [aRR = 4.23; 95% CI 3.55–5.03]). Neonates of individuals with chorioamnionitis had higher risk of NICU admission (17.4% vs 6.3% [aRR = 2.87; 95% CI 2.81–2.93]), respiratory distress syndrome (11.0% vs 3.3% [aRR = 2.78; 95% CI 2.70–2.85]), jaundice (24.8% vs 16.9% [aRR = 1.23; 95% CI 1.22–1.25]), hypoxic ischemic encephalopathy (0.4% vs 0.1% [aRR = 4.53; 95% CI 3.87–5.29]), and Apgar scores <3 at five minutes (0.5% vs 0.1% [aRR = 2.92; 95% CI 2.54–3.36]).

**Conclusion**: Chorioamnionitis was associated with elevated maternal and neonatal risk in term pregnancies even when controlling for potential confounders. How to mitigate this risk deserves both translational works to elucidate causal pathways and clinical trials to investigate prophylaxis or better treatment options.

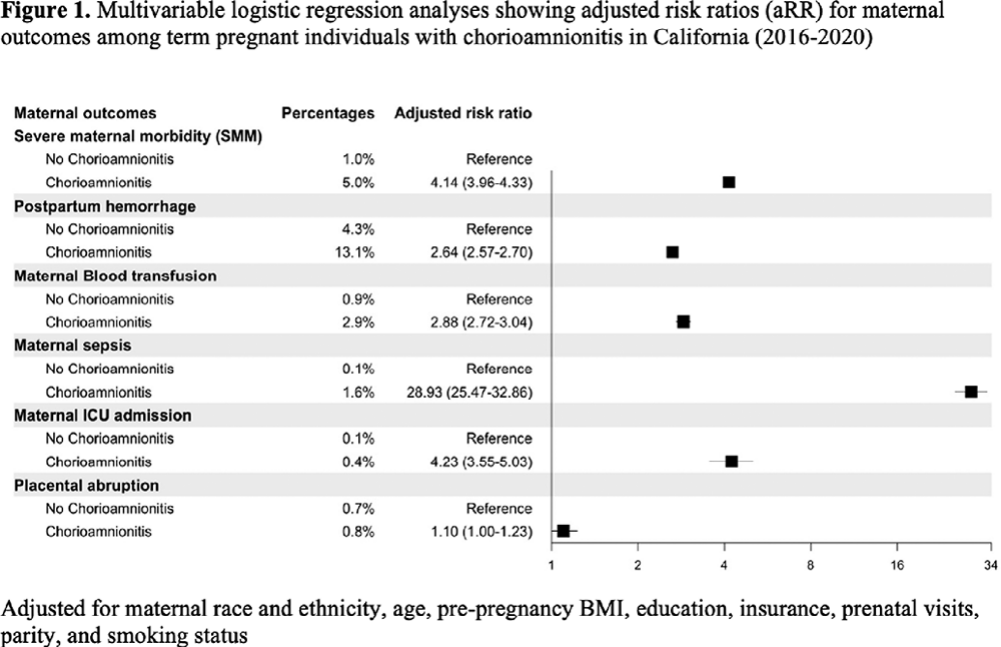

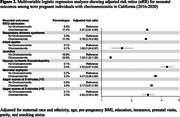

**3:30 PM – 5:00 PM**

## 626 | **Is Cell‐Free Fetal** DNA **Methylation Patterning Different Among Pregnancies Affected With Fetal Growth Restriction?**

Preethi Gopal^1^; Vanya Manthena^2^; Ryan E. Longman^3^; Chuan He^1^; Alana Beadell^1^; Ashish Premkumar^1^

^1^University of Chicago, Chicago, IL; ^2^University of Chicago Medicine, Chicago, IL; ^3^University of Chicago, Biological Sciences Division, Chicago, IL

**Objective**: To assess whether there are significant differences in maternal‐fetal circulating cell‐free DNA 5‐methylcytosine (5mC) patterns between people carrying growth‐restricted fetuses those carrying non‐growth restricted fetuses.

**Study Design**: We conducted a prospective cohort study of patients above the age of 18 with a live, singleton affected with fetal growth restriction (FGR) above 20 0/7 weeks and unaffected singletons, matched by gestational age. After consent, 4 cc of venous blood was obtained by a trained study phlebotomist. Cell‐free fetal DNA was extracted from maternal serum and subjected to fetal whole genome 5mC evaluation via Ultra‐Mild Bisulfite sequencing and Linear Amplification of Bisulfite Sequencing techniques, aimed at improving the yield and specificity of analyses. The primary outcome was the difference in size of maternal‐fetal cfDNA fragments, while the secondary outcome was the numerical difference in upregulated and downregulated genes in cffDNA. High‐throughput sequencing data was analyzed for differentially methylated gene promotors, defined as statistically significant with 20% difference in mean methylation levels between cases and controls.

**Results**: We recruited 10 participants (*n* = 5 with FGR and *n* = 5 unaffected), of whom 9 had sufficient cffDNA for analyses. The median gestational age at diagnosis was 29 weeks. For the primary outcome, cffDNA in the FGR group exhibited more total DNA at sizes below 230 basepairs when compared to unaffected fetuses (Figure 1, sections B‐C). We further identified the promoters of 688 transcripts from 502 different genes to be differentially methylated between participants with FGR and healthy controls. 273 of these promoters were significantly more methylated in participants with FGR; 415 promoters were significantly more methylated in controls (Figure 2).

**Conclusion**: In this pilot study, there are differences in cffDNA 5mC patterns among fetuses affected by growth restriction compared to unaffected fetuses. Future research should evaluate its association with maternal and perinatal outcomes in the setting of FGR.

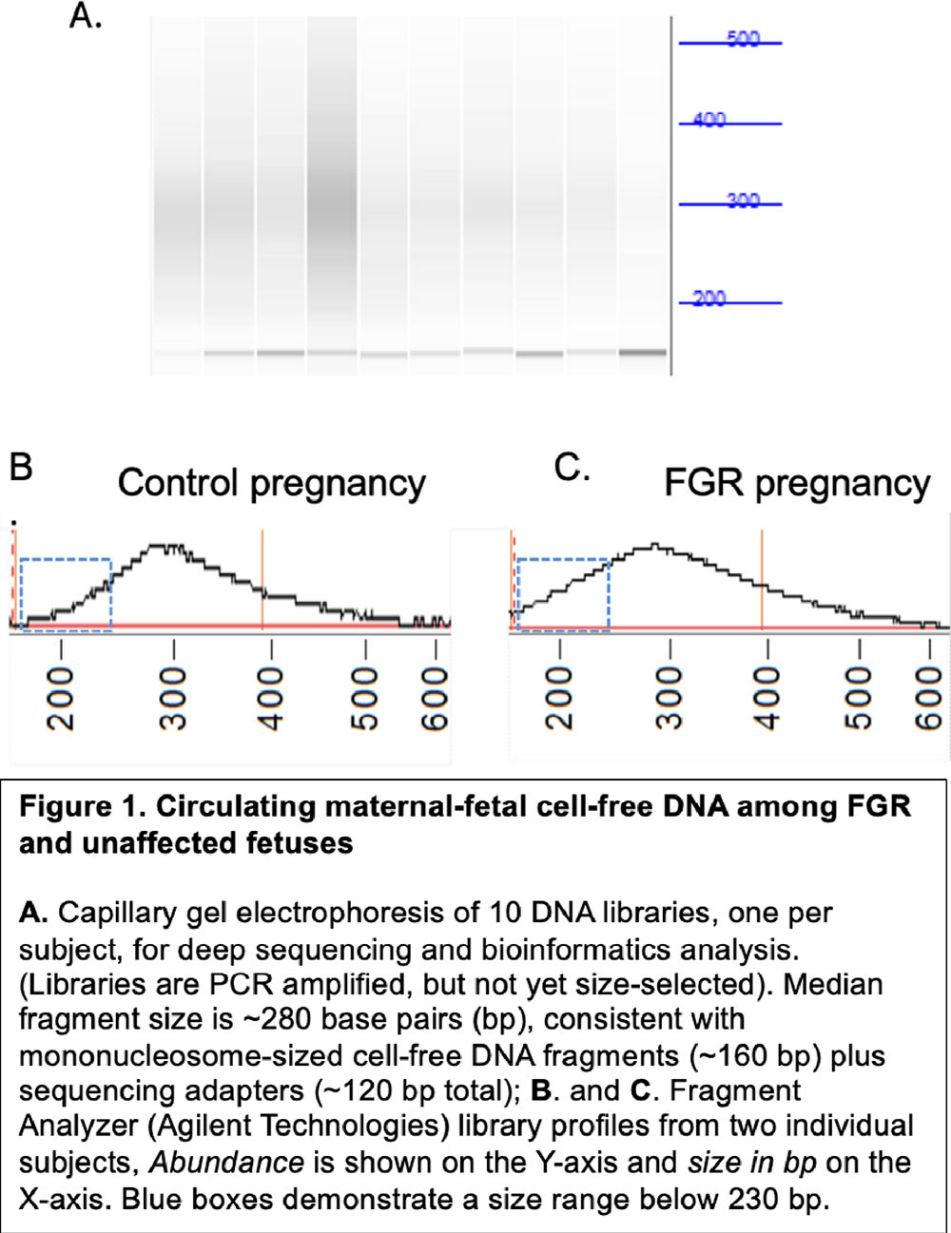

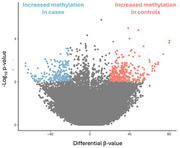

**3:30 PM – 5:00 PM**

## 627 | **Association** Between **Oxytocin Exposure in the Second Stage of Labor and Postpartum Hemorrhage**

Rachel L. Bank^1^; Miriam J. Alvarez^1^; Sindhu K. Srinivas^2^; Aaron B. Caughey^3^; Alan TN Tita^4^; Methodius G. Tuuli^5^; George A. A. Macones^1^; Alison G. G. Cahill^6^

^1^Department of Women's Health, Dell Medical School at the University of Texas at Austin, Austin, TX; ^2^Department of Obstetrics and Gynecology, Perelman School of Medicine, Maternal and Child Health Research Center, Philadelphia, PA; ^3^Oregon Health & Science University, Oregon Health & Science University, OR; ^4^Center for Research in Women's Health, University of Alabama at Birmingham, Birmingham, AL; ^5^Department of Obstetrics and Gynecology, Warren Alpert Medical School at Brown University, and Women & Infants Hospital of Rhode Island, Providence, RI; ^6^Department of Women's Health, Dell Medical School at the University of Texas at Austin, Department of Women's Health, Dell Medical School at the University of Texas at Austin, TX

**Objective**: Oxytocin, when used during labor, is often continued into the second stage, yet its relationship with postpartum hemorrhage risk is not known. We sought to evaluate the relationship between second stage oxytocin exposure and postpartum bleeding outcomes.

**Study Design**: Secondary analysis of a multicenter randomized controlled trial of nulliparous women with term singleton pregnancies comparing immediate vs. delayed pushing in the second stage. In this study, the primary exposure was cumulative second stage oxytocin dose, calculated as the product of the infusion rate at complete cervical dilation (mU/min) and second stage duration (min). Patients were categorized as no, low, or high exposure, based on 50^th^ percentile dose (680 mU; equivalent to a duration of 68 min at 10 mU/min). Exclusions were incomplete data or outlier values. The primary outcome was postpartum hemorrhage (PPH), defined as estimated blood loss ≥500 mL (vaginal) or ≥1000 mL (cesarean). Secondary outcomes included uterotonic use, transfusion, and a composite. Log‐binomial regression estimated risk ratios (RRs) with 95% confidence intervals (CIs). Adjusted models controlled for primary trial group, induction, and prolonged second stage (>3 hrs).

**Results**: Of 2404 patients in the parent trial, 1772 were included. High‐dose oxytocin was not associated with increased risk of PPH (aRR = 1.5; 95% CI: 0.9–2.4; *p* = 0.10), the composite outcome (aRR = 1.5; 95% CI: 0.9–2.4; *p* = 0.11), or uterotonic use (aRR = 1.3; 95% CI: 0.5–3.2; *p* = 0.58) versus no exposure. Low‐dose exposure was not associated with increased risk for any outcome. In sensitivity analyses defining PPH as estimated blood loss ≥1000 mL, results remained consistent with no significant differences observed. Transfusion rates were low and did not differ significantly between groups.

**Conclusion**: Both high and low dose oxytocin exposure in the second stage of labor were not associated with increased risk of maternal blood loss complications. Continuation of oxytocin into the second stage of labor does not appear to be an independent risk factor for postpartum hemorrhage.

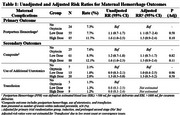

**3:30 PM – 5:00 PM**

## 628 | **Potassium** Chloride **Injection Training and Impacts on Procedural Characteristics**

Rachel Mnuk^1^; Wendy Tian^2^; Emily R. Boniface^3^; Marie J. Boller^3^; Adam Crosland^3^

^1^Oregon Health and Science University, PORTLAND, OR; ^2^Oregon Health & Science University, Portland, OR; ^3^Oregon Health and Science University, Portland, OR

**Objective**: Ultrasound‐guided potassium chloride (KCl) injections to induce fetal asystole is a procedure typically performed by Maternal‐Fetal Medicine (MFM) physicians, requiring specialized training. Data outlining procedural characteristics and necessary curricular components for competence are limited. This study's objective was to describe the procedural characteristics of KCl injections by post‐graduate year (PGY) among MFM fellows.

**Study Design**: This descriptive retrospective study included individuals undergoing pregnancy termination at ≥ 22 weeks at Oregon Health & Science University between June 1, 2021–June 1, 2023. Maternal, fetal, and procedural variables were abstracted from electronic medical records. Procedural variables included duration (with prolonged procedure defined as >10 minutes), number of attempts, KCl dose, anesthetic use, needle gauge, and trainee involvement. We described procedure characteristics, overall and by fellowship year, and compared fellowship years.

**Results**: Of 99 procedures analyzed, 65 (65.7%) involved fellows. Procedures by PGY level were distributed as follows: 19 (PGY5), 33 (PGY6), and 13 (PGY7). The following trends were noted. Fetal anomalies were present in 36.8% of PGY5 procedures, increasing to 63.6% for PGY6 and 61.5% for PGY7. Median estimated fetal weight was 679g (PGY5), increasing to 785g (PGY6) and 730g (PGY7). Median (range) procedure time was similar among groups at 11 (3–41) minutes; procedures were prolonged in 47.4% of PGY5, 54.6% of PGY6, and 46.2% of PGY7 cases. Multiple procedure attempts occurred in approximately 21% of cases for PGY5 and PGY6, decreasing to 15.4% for PGY7. Remaining results in Table 1 & 2.

**Conclusion**: The observed trends suggest that higher rates of prolonged procedures and repeat attempts for PGY6 cases likely reflect increased exposure to complex cases with advancing experience. The reduction in procedure duration and attempts by PGY7 may indicate growing proficiency, reinforcing the value of structured longitudinal training and graduated supervision for procedural competence.

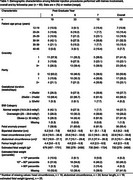

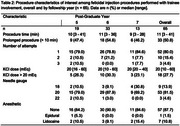

**3:30 PM – 5:00 PM**

## 629 | **Is Isolated Single Umbilical Artery a Risk Factor for Long‐Term Gastrointestinal Morbidity in the Offspring?**

Rachel Rappaport^1^; Gil Gutvirtz^2^; Tamar Wainstock^3^; Eyal Sheiner^4^

^1^Soroka medical center, Soroka medical center, HaDarom; ^2^Department of Obstetrics and Gynecology, Soroka University Medical Center, Ben‐Gurion University, Metar, HaDarom; ^3^Department of Epidemiology, Biostatistics and Community Health Sciences, Faculty of Health Sciences, Ben‐Gurion University of the Negev, Beer Sheva, HaDarom; ^4^Soroka University Medical Center, Faculty of Health Sciences, Ben‐Gurion University of the Negev, beer sheva, HaDarom

**Objective**: Single umbilical artery (SUA) is a relatively common umbilical cord anomaly in pregnancy. While SUA is often considered a benign finding in the absence of other fetal anomalies (isolated SUA), some evidence suggests it may reflect subtle disruptions in fetal development and has been linked with adverse perinatal outcomes. However, the long‐term outcomes in offspring with isolated SUA remain poorly studied. We investigated the long‐term gastrointestinal (GI) morbidity of offspring born with isolated SUA.

**Study Design**: A population‐based cohort study was conducted to evaluate the risk of long‐term GI disease in offspring based upon the presence or absence of SUA at birth. Fetuses with chromosomal abnormalities or congenital malformations were excluded from the study to include only cases of isolated SUA. Data for the incidence of GI morbidity of the offspring was extracted from community and hospitalization records. A Kaplan Meir survival curve was constructed to compare the cumulative incidence over time of long‐term GI morbidity between the study groups. Cox proportional hazards model was used to control for possible confounders.

**Results**: The study included 232,476 singleton newborns of whom 136 (0.1%) were diagnosed with isolated SUA. A positive association was found between SUA and GI morbidity (Table). However, the cumulative incidence over time was not significantly higher in newborns with SUA as compared to children without SUA (Figure). Furthermore, after applying a Cox regression model, adjusting for maternal and gestational age, diabetes mellitus, hypertensive disorders, fetal intrauterine growth restriction and mode of delivery, the association between SUA and GI morbidity was no longer statistically significant (adjusted HR 1.0, 95% CI 0.75–1.33, *p* = 0.988).

**Conclusion**: SUA is not an independent risk factor for GI disorders and the long‐term incidence of GI disorders is comparable between children with or without SUA.

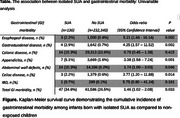

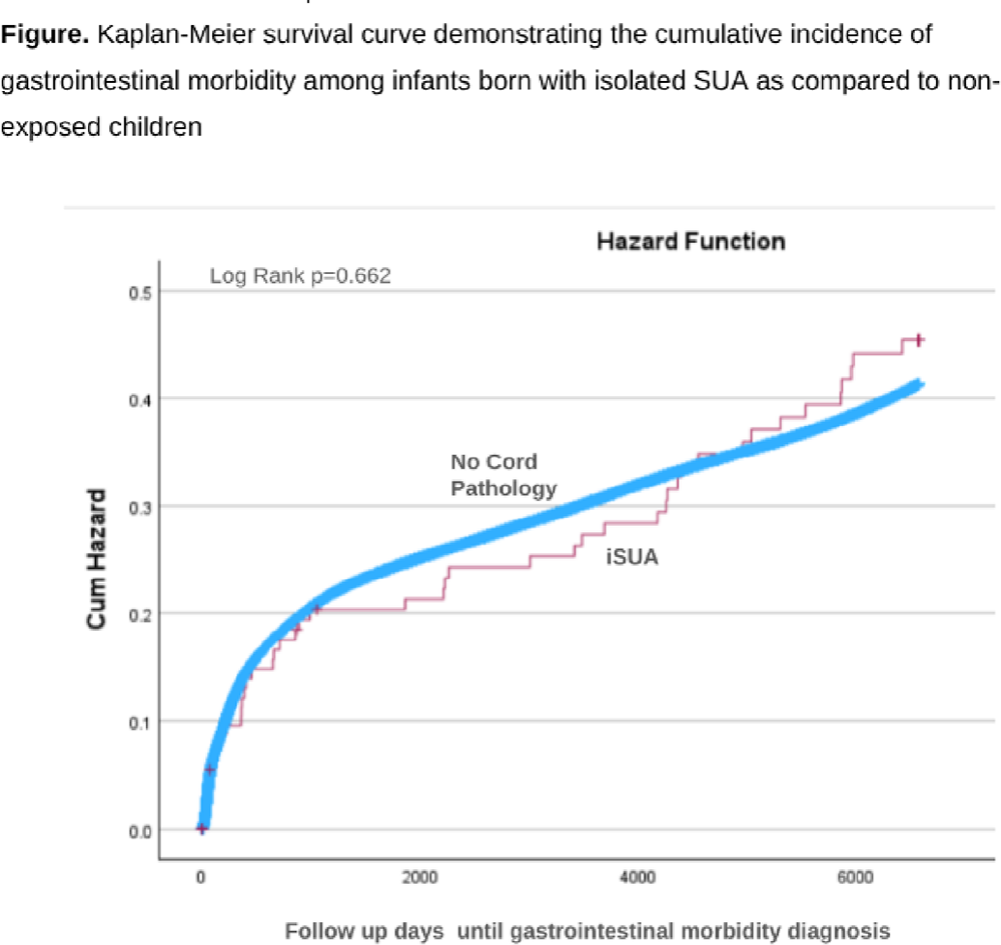

**3:30 PM – 5:00 PM**

## 630 | **Undocumented** Obstetric **Hypertension is Associated With Severe Maternal Morbidity and Unplanned Postpartum Hospital Utilization**

Rachel D. Seaman^1^; Lisbet S. Lundsberg^2^; Caitlin Partridge^2^; Anna Denoble^3^; Alejandra Barreto^4^; Jennifer F. Culhane^2^

^1^Yale School of Medicine, Yale School of Medicine, CT; ^2^Department of Obstetrics, Gynecology, and Reproductive Sciences, Yale School of Medicine, New Haven, CT; ^3^Yale School of Medicine, New Haven, CT; ^4^Yale School of Medicine, Lynwood, CA

**Objective**: Obstetric hypertension (HTN) may not always be recognized and captured by ICD‐10 diagnosis codes in the electronic medical record. However, we propose that even in the absence of an ICD‐10 code for chronic hypertension (cHTN) or hypertensive disorders of pregnancy (HDP), patients with elevated blood pressure (BP) remain at increased risk of adverse peripartum outcomes. We aimed to compare (1) rates of severe maternal morbidity (SMM) at delivery and (2) rates of unplanned hospital utilization within 6 weeks postpartum (PP) amongst normotensive patients and those with undocumented/documented HTN.

**Study Design**: This was a retrospective study of patients delivering within a single system from 2013–2024 with ≥1 prenatal visit and ≥2 recorded BPs. Patients were classified as “normotensive” (no HTN), “BP only” (≥2 BPs of ≥140/90 mmHg from 0–20 weeks’ gestation or 20 weeks‐delivery discharge *without* an ICD‐10 code for cHTN/ HDP), or “ICD‐10” (*with* an ICD‐10 code for cHTN/HDP). Demographic and clinical attributes were compared across groups using chi‐square tests. The primary outcomes were SMM and ED visits or inpatient readmission within 6 weeks PP. These were compared using multivariate logistic regression, adjusted for significant covariates.

**Results**: Of 59734 patients, 33229 (55.6%) were identified as normotensive, 11443 as BP only (19.2%), and 15062 (25.2%) as ICD‐10 (Table 1). The ICD‐10 group experienced the highest rates of SMM (8.1%, aOR 2.4 [95% CI 2.2–2.7]), PP ED visits (10.7%, 2.0 [1.9–2.2]), and readmissions (7.1%, 7.6 [6.6–8.7]) (Table 2). However, the BP only group was also at increased risk of adverse outcomes compared to the normotensive group, including SMM (3.9 vs 2.8%, aOR 1.4 [95% CI 1.3–1.6]) and PP ED visits (5.6 vs 4.8%, 1.2 [1.1–1.3]), although not readmissions [1.0 vs 0.9%, 1.2 [0.9–1.4]).

**Conclusion**: Even in the absence of a formal diagnosis by ICD‐10 coding, elevated peripartum BP is associated with SMM and PP hospital utilization. This further highlights the importance of appropriate recognition and documentation of antenatal HTN.

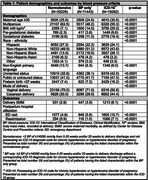

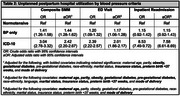

**3:30 PM – 5:00 PM**

## 631 | **Neonatal Fat** Mass **and Hypoglycemia in Infants of Pregnancies Complicated by Diabetes**

Rachel L. Wiley^1^; Claire E. Jensen^2^; Kim Boggess^3^; Marni B. Jacobs^1^; Gladys A. Ramos^4^

^1^Division of Maternal Fetal Medicine, Department of Obstetrics, Gynecology and Reproductive Sciences, University of California San Diego, San Diego, CA; ^2^University of North Carolina, Chapel Hill, NC; ^3^University of North Carolina at Chapel Hill, Chapel Hill, NC; ^4^Division of Maternal Fetal Medicine, Department of Obstetrics, Gynecology and Reproductive Sciences, San Diego, CA

**Objective**: Neonates of pregnancies affected by T2DM are at increased risk for complications, including hypoglycemia and long‐term metabolic sequelae. Neonatal body composition may reflect in utero metabolic effects, but its association with neonatal hypoglycemia is unclear.

**Study Design**: This is a secondary analysis of a multicenter randomized controlled trial investigating metformin use in pregnancies complicated by insulin treated T2DM and diabetes diagnosed in early pregnancy. Singleton, liveborn neonates delivered at >34 weeks with anthropometric measurements collected based on staff availability were included. Fat mass was calculated using skin fold measurements. The primary outcome was neonatal hypoglycemia was defined as an initial glucose <40 mg/dL or requiring IV or oral treatment for hypoglycemia within 72 hours of birth. The association between fat mass and neonatal hypoglycemia was adjusted for gestational age, HbA1c at delivery and pre‐existing diabetes.

**Results**: Of 794 newborns born to participants in the primary trial, 418 (52.6%) newborns met inclusion criteria for this secondary analysis and 167 (39.9%) had hypoglycemia. Maternal characteristics differed between infants with hypoglycemia versus those normoglycemic. (Table 1). In both unadjusted and adjusted analysis, neonatal fat mass was higher in infants with hypoglycemia (525.5 g, 95% CI 453.0–597.0) compared to normoglycemia (429.9 g, 95% CI 362.1–497.6; *p* < 0.01, Figure 1), with significant differences in birthweight, fat free mass, body mass percentage and mid‐upper arm circumference (Table 2).

**Conclusion**: Increased fat mass and body fat percentage is associated with neonatal hypoglycemia. These findings suggest neonatal adiposity may reflect fetal metabolic effects of diabetes during pregnancy and warrant further investigation as a potential marker of future metabolic risk.

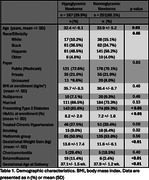

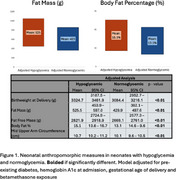

**3:30 PM – 5:00 PM**

## 632 | **Immediate** Induction **Versus Expectant Management in Primiparas Presenting With Decreased Fetal Movements at 39 Weeks**

Raneen Abu Shqara^1^; Kylie Ella Marcovich^2^; Nadir Ganem^3^; Lior Lowenstein^3^; Maya Frank Wolf^3^

^1^Galilee Medical Center, Nahariya, HaZafon; ^2^Bar‐Ilan University, Nahayria, HaZafon; ^3^Galilee Medical Center, Naharyia, HaZafon

**Objective**: To evaluate maternal and neonatal outcomes in primiparous patients presenting with a subjective complaint of decreased fetal movements (DFM), comparing those who underwent immediate labor induction to those who declined and were managed expectantly.

**Study Design**: This retrospective cohort study included nulliparous patients with singleton pregnancies who presented to our obstetric triage unit between 39+0 and 39+6 weeks of gestation with a subjective complaint of DFM, a reassuring fetal assessment, a normal biophysical profile, and an amniotic fluid index (AFI) >50 mm. In accordance with departmental protocol, patients were offered labor induction. Those who agreed formed the induction group, while those who declined and were discharged home comprised the control group. Labor induction was guided by the Bishop score: patients with a score <8 underwent cervical ripening using a balloon catheter or dinoprostone, while those with a score≥8 received oxytocin. Maternal and neonatal outcomes were compared between groups.

**Results**: A total of 413 patients were included: 282 in the induction group and 131 in the expectant management group. Baseline characteristics were comparable. Delivery week was earlier in the induction group (39.4 ± 0.3 vs. 40.1 ± 0.3 weeks, *p* < 0.001). No significant differences were observed in birthweight (*p* = 0.945), cesarean delivery rates (*p* = 0.683) or NICU admission (*p* = 0.696). The induction group had significantly fewer neonates with cord pH <7.15 (2.5% vs. 6.9%, *p* = 0.001), and neonatal hospitalization (days) was slightly shorter (2.0 ± 0.2 vs. 2.3 ± 0.5 days, *p* = 0.034). Total maternal hospitalization duration was significantly longer in the induction group (3.6 ± 0.6 vs. 2.2 ± 0.5 days, *p* < 0.001), though postpartum stay was shorter (2.0 ± 0.2 vs. 2.2 ± 0.1 days, *p* < 0.001).

**Conclusion**: Among primiparous patients presenting with DFM between 39+0 and 39+6 weeks, immediate labor induction was associated with earlier delivery and improved umbilical cord pH without increasing maternal or neonatal complications. Findings support considering induction; randomized trials are warranted.

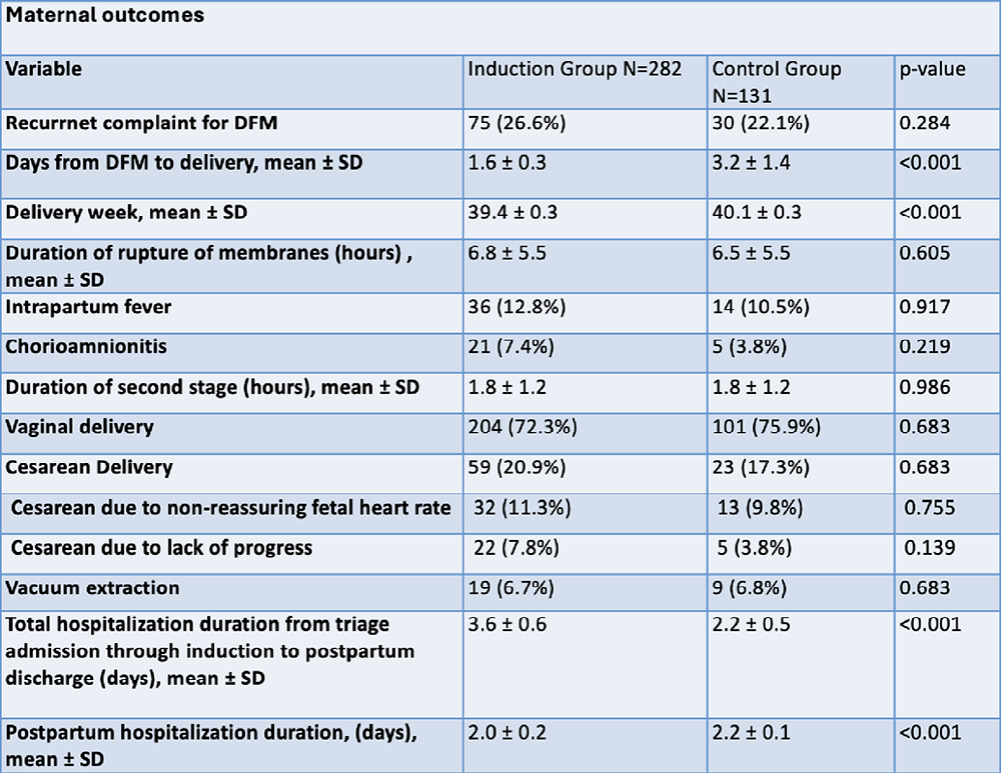

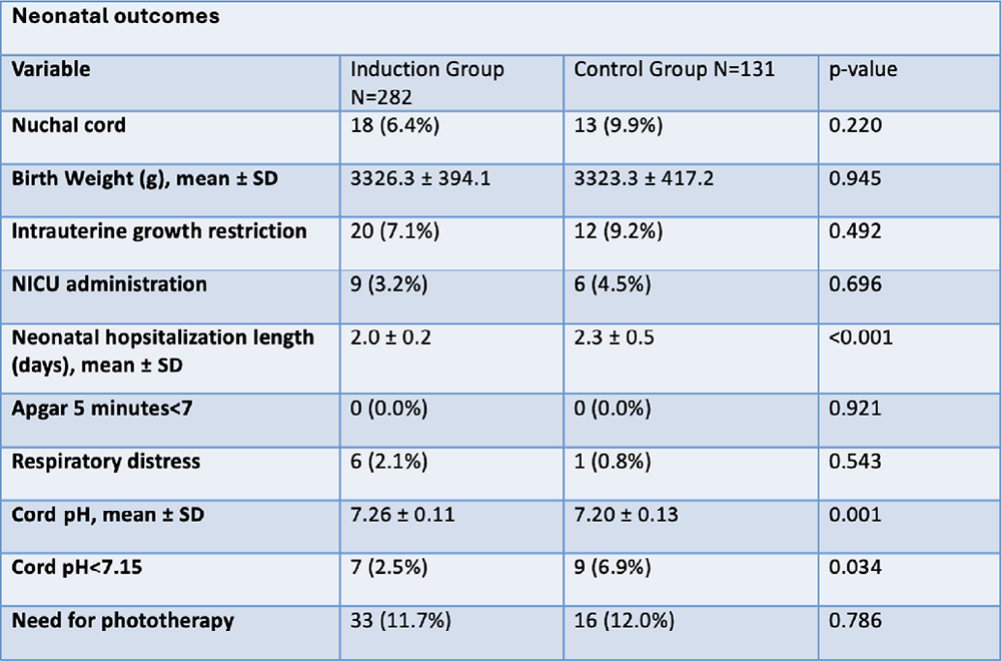

**3:30 PM – 5:00 PM**

## 633 | **Impact of** Placental **sFLT‐1 on Neonatal Bronchopulmonary Dysplasia**

Ravi P. Chokshi^1^; Allen Kunselman^2^; Kathryn McMullen^3^; Noor Banihashem Ahmad^4^; Nayera Guirguis^5^; Serdar Ural^6^; Shaili Amatya^7^

^1^Penn State Health, HUMMELSTOWN, PA; ^2^Pennsylvania State University College of Medicine: Penn State College of Medicine, Hershey, PA; ^3^Penn State Health Milton S. Hershey Medical Center, Hershey, PA; ^4^Penn State College of Medicine, Hershey, PA; ^5^Penn State Hershey, Penn State Hershey, PA; ^6^The Pennsylvania State University College of Medicine, The Pennsylvania State University College of Medicine, PA; ^7^Penn State Hershey Medical Center, Hershey, PA

**Objective**: Bronchopulmonary dysplasia (BPD) is the most frequent complication of preterm birth, impacting about 50% of infants born before 28 weeks of gestation. Babies born to mothers with preeclampsia are at greater risk of developing BPD. The suspected cause is a disruption in the Vascular Endothelial Growth Factor (VEGF) signaling pathway, which affects fetal alveolar angiogenesis. Placental angiogenic factors, particularly soluble fms‐like tyrosine kinase (sFLT‐1), interact with the VEGF pathway, and animal studies have shown that sFLT‐1 can cause direct fetal lung injury.

While previous research has measured sFLT‐1 levels in maternal and cord blood during preeclampsia, no studies have explored whether this biomarker remains in a newborn's blood after birth or its potential effects. This project aims to measure sFLT‐1 levels in neonatal blood samples and investigate whether higher levels are linked to severe BPD.

**Study Design**: Plasma samples from extremely preterm newborns (born before 28 weeks of gestation) were collected during their first week of life and analyzed using validated ELISA kits to measure sFLT‐1 levels. This pilot study tested 35 patients. The primary outcome was the presence or absence of severe BPD, as defined by the NICHD 2019 criteria. The study was approved by our Institutional Review Board.

**Results**: sFLT‐1 was detected in all samples, with levels ranging from 191 to 5375 pg/mL and a median of 943 pg/mL. After adjusting for Fetal Growth Restriction (FGR) and maternal hypertension using binary logistic regression, every 100 pg/mL increase in sFLT‐1 was associated with an 8.5% increased odds for severe BPD. However, this finding was not statistically significant (OR = 1.085, 95% CI: 0.962–1.223, *p*‐value = 0.18).

**Conclusion**: This pilot study is the first to demonstrate that sFLT‐1, a placentally secreted angiogenic factor, is consistently present in the early circulation of extremely preterm newborns. Given its influence on the VEGF signaling pathway, sFLT‐1 may have downstream effects on fetal and early neonatal development, warranting further research.

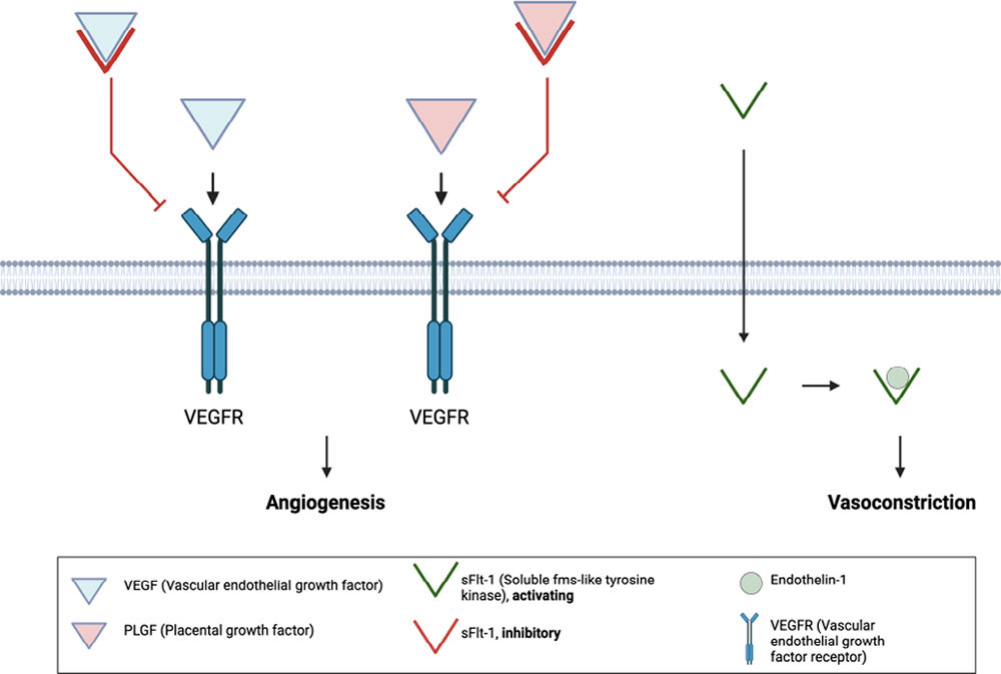

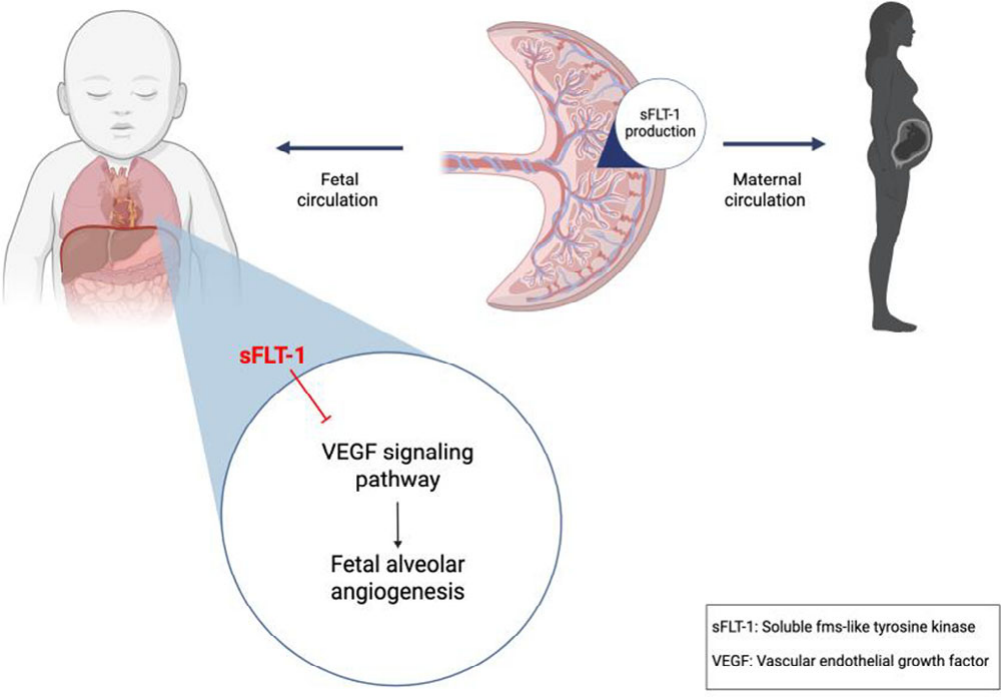

**3:30 PM – 5:00 PM**

## 634 | **Optimizing** Outpatient **Cervical Ripening: A Mixed‐Methods Study Identifying Implementation Drivers During A Multi‐Site, Randomized Trial**

Rebecca F. Hamm^1^; Matthew K. Hoffman^2^; Brett D. Einerson^3^; Anthony C. Sciscione^2^; Robert M. Silver^3^; Miriam J. Alvarez^4^; Alison G. G. Cahill^5^; Lisa D. Levine^1^

^1^Perelman School of Medicine, University of Pennsylvania, Philadelphia, PA; ^2^Christiana Care Health System, Newark, DE; ^3^Department of Obstetrics and Gynecology, University of Utah Health, Salt Lake City, UT; ^4^Department of Women's Health, Dell Medical School at the University of Texas at Austin, Austin, TX; ^5^Department of Women's Health, Dell Medical School at the University of Texas at Austin, Department of Women's Health, Dell Medical School at the University of Texas at Austin, TX

**Objective**: To characterize provider and organizational level determinants of implementation related to outpatient cervical ripening with a Foley catheter.

**Study Design**: This sequential mixed‐methods study (quan‐ >QUAL; 12/2022–1/2025) was embedded into an ongoing 5‐site randomized clinical trial comparing outpatient versus inpatient cervical ripening. Prior to trial initiation, surveys (quan) were distributed to obstetric clinicians at all sites. The survey collected demographics, Foley placement experience, perceived acceptability and feasibility of outpatient Foley via validated measures [Likert; total 4–20]. One year into the trial, clinician focus groups (QUAL) assessed barriers, facilitators, and sustainability of outpatient Foley, guided by the Consolidated Framework for Implementation Research 2.0 and coded by 2 independent coders.

**Results**: 396 clinicians (64% physicians, 22% nurses, 10% CNMs, 4% NPs/PAs) completed the pre‐trial survey; 29.2% had previously used outpatient ripening. Acceptability (19/20 IQR[16–20]) and feasibility (16/20 IQR[15–20]) were high overall but varied by site (*p* = 0.003; *p* = 0.006). Predictors of acceptability and feasibility included female gender and physician/CNM role. As a result, focus groups were divided by site and role to elicit site‐specific barriers and facilitate open discussion among nurses without physicians/CNMs present. Twelve focus groups (*n* = 33) identified facilitators including belief in the evidence/safety and benefit, as well as leadership support. Barriers included concerns about timely hospital return, perceived patient discomfort, and operational constraints (e.g. space and staffing). In response, participants discussed systematic strategies to promote sustainability, several already enacted, such as standardized patient education and workflow solutions (Table).

**Conclusion**: This work identified key determinants influencing implementation of outpatient cervical ripening with a Foley catheter during a multi‐site trial. Findings can inform strategies for future study and to drive success for sites implementing outpatient Foley clinically.

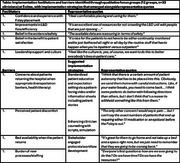

**3:30 PM – 5:00 PM**

## 635 | **Evaluating** Machine **Learning for Recurrent Preterm Birth Prediction: Secondary Analysis of the Omega‐3 Trial**

Rebecca Horgan^1^; Erkan Kalafat^2^; Marwan Ma'ayeh^1^; Maged M. Costantine^3^; George R. Saade^1^

On behalf of the Eunice Kennedy Shriver National Institute of Child Health and Human Development (NICHD) Maternal‐Fetal Medicine Units (MFMU) Network, Bethesda, MD

^1^Eastern Virginia Medical School at Old Dominion University, Norfolk, VA; ^2^Koc University Hospital, Istanbul, Koc University Hospital, Istanbul, Istanbul; ^3^Ohio State University, Columbus, OH

**Objective**: To determine whether recurrent preterm birth (PTB) in women with a prior spontaneous PTB (sPTB) can be predicted using machine learning (ML).

**Study Design**: This is a secondary analysis of a randomized trial of antioxidant for prevention of recurrent PTB amongst patients with a history of prior sPTB (NCT00135902). The primary analysis showed no benefit. For this analysis, patients were categorized by delivery at <32 weeks, 32 to 36 weeks, or ≥37 weeks. We developed a multinomial XGBoost machine learning model to assess prediction of recurrent PTB. The model included baseline demographic and historical obstetric factors that were significantly different between the outcome groups. To assess performance, the dataset was randomly partitioned into training and validation sets over 50 iterations. The mean sensitivity and positive predictive value (PPV) for each outcome category were calculated across all runs.

**Results**: All 841 women enrolled in the trial were included in this analysis (500 in training and 341 in the testing sets). The model's predictive ability was modest. For the prediction of delivery at <32 weeks, mean sensitivity was 24.2% (± 17.9) with a PPV of 5.3% (± 3.7). For delivery between 32 and 36 weeks, mean sensitivity was 42.9% (± 5.8) with a PPV of 23.1% (± 5.3). The model performed best at identifying term deliveries, with a mean sensitivity of 60.8% (± 2.9) and a PPV of 84.4% (± 3.9) (Figure 1). Analysis of feature importance revealed that historical obstetric factors, such as gestational age of the earliest prior preterm birth and the gestational age at the last delivery, were the most significant predictors (Figure 1). Despite the importance of these historical parameters, the overall low to modest predictive performance suggests that these factors alone do not sufficiently explain the timing of recurrent PTB.

**Conclusion**: The ML model demonstrated modest ability to predict recurrent PTB in women with prior sPTB, indicating that baseline demographic and obstetric factors alone are insufficient for accurate risk stratification.

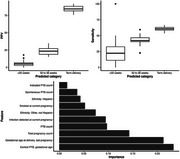

**3:30 PM – 5:00 PM**

## 636 | **First Trimester** Soluble **Fms‐Like Tyrosine Kinase 1 (sFlt‐1) and Its Association With Adverse Neonatal Outcomes**

Rebecca Nunge^1^; Angela J. Johnson^1^; Emily Heideman^1^; Anthony O. Odibo^2^; Jose R. Duncan^1^

^1^University of South Florida, Tampa, FL; ^2^University of Missouri Kansas City, Kansas City, MO

**Objective**: To evaluate the association between first‐trimester angiogenic biomarkers, including of soluble fms‐like tyrosine kinase‐1 (sFlt‐1), vascular endothelial growth factor (VEGF), placental growth factor (PIGF), and sFlt‐1/PIGF ratio, and a composite of adverse neonatal outcomes (CANO).

**Study Design**: This was retrospective case‐control study of pregnancies enrolled in a first‐trimester screening program for adverse neonatal outcomes and the redefinition of fetal growth restriction trial. We included singleton gestations at risk for fetal growth disorders between 26.0 and 36.6 weeks with available first‐trimester measurements of sFlt‐1, VEGF, and PlGF, and sFlt‐1/PIGF ratio. Fetuses with chromosomal or congenital anomalies were excluded. We assessed the association of first‐trimester sFlt‐1, PlGF, and VEGF levels with a CANO using the area under the receiver operating characteristic curve (AUC). CANO included one or more of: neonatal intensive care unit admission, pH <7.1, Apgar score <7 at 5 minutes of life, seizures, respiratory distress syndrome, intraventricular hemorrhage grade III or IV, or neonatal death. Optimal cutoff values were determined, and biomarker levels were compared between pregnancies with and without CANO for statistically significant predictors.

**Results**: Among the 55 pregnancies analyzed, 15 neonates experienced CANO. First‐trimester sFlt‐1 levels were significantly associated with CANO (AUC: 0.70; 95% CI: 0.53–0.86; *p* = 0.018). In contrast, PlGF (AUC: 0.36; 95% CI: 0.21–0.52; *p* = 0.09) and VEGF (AUC: 0.51; 95% CI: 0.34–0.68; *p* = 0.93) were not significantly associated with the outcome (Figure 1). Pregnancies with CANO had significantly higher sFlt‐1 levels compared to those without (Figure 2). An sFlt‐1 threshold of 3588.29 pg/ml was identified as optimal cutoff for predicting CANO.

**Conclusion**: In this cohort of pregnancies at risk for fetal growth disorders, first‐trimester sFlt‐1 was significantly associated with adverse neonatal outcomes. Larger, prospective studies in more diverse populations are warranted to validate these findings.

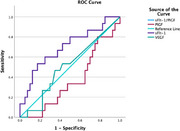

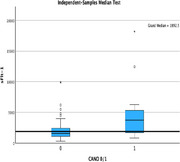

**3:30 PM – 5:00 PM**

## 637 | **Evaluating Acute Renal Failure as an Indicator of Severe Maternal Morbidity**

Rie Maeda^1^; Elizabeth Raiff^2^; Justin R. Lappen^2^; Adina R. Kern‐Goldberger^1^

^1^Cleveland Clinic Lerner College of Medicine, Cleveland, OH; ^2^Cleveland Clinic Foundation, Cleveland, OH

**Objective**: Acute renal failure (ARF) is among the most common indicators of the CDC severe maternal morbidity (SMM) composite. There is a wide range of clinical circumstances that may trigger a diagnosis code for ARF, including transient acute kidney injury (AKI) which typically has minimal implications for a patient's clinical care and does not represent a severely morbid outcome. The purpose of this study was to characterize the etiologies and severity of ARF experienced by patients with this SMM indicator during the delivery admission.

**Study Design**: This cross‐sectional study conducted in a single tertiary hospital included all patients delivered 1/1/2022–6/30/2024 >22 weeks with ARF as defined by the CDC SMM composite. Patient demographics, clinical histories, obstetric outcomes, and the details of their acute renal condition were derived from the electronic health record via detailed chart review and evaluated descriptively. They were also compared in bivariable analysis among patients with and without concurrent hypertensive disorders of pregnancy (HDP).

**Results**: 88 total patients were included (out of 13,685 total deliveries during the study period; 0.64%). Of these, only 40 (45%) had a clear suspected etiology of ARF described in the chart (Figure 1). The leading suspected etiology was acute blood loss, followed by obstructive uropathy. The majority of patients had HDP (76%) though there were no differences in the examined characteristics among those with and without HDP other than gestational age at delivery (Table 1). Median highest serum creatinine was 1.2 mg/dL in both groups (IQR 1.1–1.4) and 18 patients (20.5%) had a creatinine above 1.5 mg/dL at any point during the hospitalization. A minority of patients required higher level care for ARF including nephrology consult or dialysis or had additional SMM indicators.

**Conclusion**: These data highlight that the majority of patients with ARF according to the CDC SMM criteria have minor AKI without other renal morbidity. Including ARF as an indicator for SMM may distort this composite outcome.

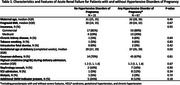

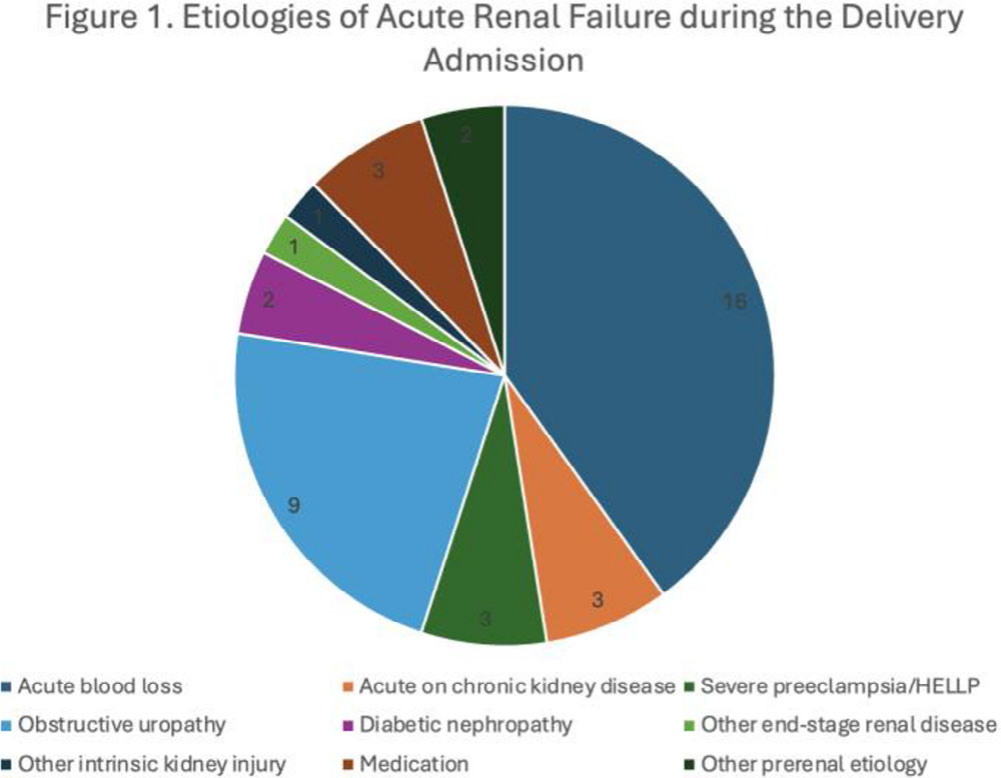

**3:30 PM – 5:00 PM**

## 639 | **Two Antenatal** Corticosteroid **Doses Are Necessary to Decrease Neonatal Mortality in VLBW Infants**

Risa Arai^1^; Takashi Shimokawa^2^; Katsufumi Otsuki^3^; Yoshio Matsuda^4^

^1^Showa medical university koto toyosu hospital / Obestetrics and Gynecology, Koto‐Ku, Tokyo; ^2^Showa Medical University Koto Toyosu Hospital, Koto‐ku, Tokyo; ^3^Perinatal Center, Department of Obstetrics and Gynecology / Showa Medical University Koto Toyosu Hospital, Koto‐ku, Tokyo; ^4^Tokyo Healthcare University / obestetrics and gyneclology, Tokyo Healthcare University / Shinagawa, Tokyo

**Objective**: To investigate the impact of the number of antenatal corticosteroid (ACS) administrations on short‐ and long‐term outcomes in very‐low‐birth‐weight (VLBW, 400–1,500 g) infants using a large multicenter cohort from Japan.

**Study Design**: This secondary analysis used data of 3317 infants from 40 perinatal centers nationwide, originally compiled for a study by Nishida et al. in 2024. After excluding twins, major congenital or chromosomal anomalies, gestational age ≥34 weeks, unknown ACS use, placenta previa, and placental abruption, 1866 infants remained. Outcomes included NICU mortality, short‐term morbidities—respiratory distress syndrome (RDS), patent ductus arteriosus (PDA), sepsis, chronic lung disease (CLD), intraventricular hemorrhage (IVH), necrotizing enterocolitis (NEC)—and neurological impairment at three years. Univariate analyses identified perinatal factors associated with mortality, which were adjusted in multivariate logistic regression assessing the association between ACS doses and outcomes.

**Results**: NICU mortality was significantly associated with gestational age, birth weight, premature rupture of membranes, clinical chorioamnionitis, ACS use, and pregnancy‐induced hypertension, but not with umbilical artery pH, non‐reassuring fetal status, gestational diabetes, maternal age, or infant sex. A single ACS dose significantly reduced short‐term morbidities except NEC. However, mortality did not differ between no ACS and a single dose, whereas two doses were required for a significant mortality reduction. No association was observed between ACS doses and neurological impairment at three years.

**Conclusion**: In VLBW infants, one ACS dose reduced neonatal complications, while two were necessary to lower mortality. ACS did not improve long‐term neurodevelopment, possibly reflecting environmental influences. These findings may help balance effective ACS use with minimizing unnecessary exposure.

**3:30 PM – 5:00 PM**

## 640 | **U.S. State‐Level** Periconception **GLP‐1 Receptor Agonist Prescription Prevalence in 2023 and 2024**

Robert E. Jones; Ethan Litman; Chloe Zera; Taylor S. Freret

Beth Israel Deaconess Medical Center, Boston, MA

**Objective**: Glucagon‐like peptide 1 receptor agonist (GLP‐1RA) use in the U.S. is increasing. The impact on exposure risk during pregnancy is uncharacterized. We sought to examine recent state‐level prevalence and changes in periconception GLP‐1RA prescriptions.

**Study Design**: This was a retrospective cohort study using the Epic Cosmos platform, a nationwide database of individuals receiving care at sites that use the Epic electronic health record. Inclusion criteria were births at >20 weeks in 2023 and 2024. Periconceptional GLP‐1RA prescriptions, ordered from 120 days before or after estimated conception, were identified. The prevalence of a GLP‐1RA prescription by state was calculated, as was the relative change in prevalence from 2023 to 2024. The number of births recorded in Cosmos was compared to U.S. natality records to determine the state sampling rate. The national prescription rate was estimated by calculating a weighted average of state‐level exposure rates, accounting for both the number of births in each state and sampling coverage.

**Results**: Of the ∼7.2 million U.S. births during the study, Cosmos reported ∼2.2 million (31.1%). Sampling rates varied by state (mean 31.2%, standard deviation 15.0%). In total, 15,025 (0.65%) had a periconception GLP‐1RA prescription. The national weighted average was 0.67%. Prescription rates varied geographically (Figure 1), as did the rate of change from 2023 to 2024 (Figure 2). Indiana, Kansas, West Virginia, and Kentucky had the highest prevalence (1.02–1.41%). Only one state (Delaware) reported a lower prescription rate in 2024 than in 2023. Tennessee, Pennsylvania, Arkansas, Missouri, Louisiana, Maryland, and South Dakota all had more than 2.5‐fold increases in prevalence.

**Conclusion**: Periconception GLP‐1RA prescriptions are becoming more common, with variation at the state level. This study provides estimates potential population‐level exposure risk at the local level that may inform approach to preconception counseling. Further study is needed to understand drivers of state‐level variability.

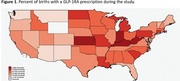

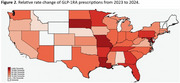

**3:30 PM – 5:00 PM**

## 641 | **Natural Onset Of** Labor **In Twin Pregnancies: A Real‐World Cohort Study**

Roie Alter^1^; Inbal Akavian^1^; Naama Lessans^2^; yossef Ezra^3^; Doron Kabiri^4^

^1^Hadassah Ein‐karem hospital, Obstetrics and gynecology department, Jerusalem, Jerusalem, Yerushalayim; ^2^Department of Obstetrics and Gynecology, Hadassah Ein Kerem Medical Center, Faculty of Medicine, Hebrew University of Jerusalem, Jerusalem, Yerushalayim; ^3^Hadassah Medical Center and Hebrew University of Jerusalem, Jerusalem, Israel, Hadassah Medical Center and Hebrew University of Jerusalem, Jerusalem, Israel, Yerushalayim; ^4^Hadassah Medical Center and Hebrew University of Jerusalem, Jerusalem, Israel, Hadassah Medical Center and Hebrew University of Jerusalem, Jerusalem, Israel, Yerushalayim

**Objective**: The optimal timing for elective cesarean delivery in twin pregnancies remains controversial, with guidelines varying between 37+0–37+6 weeks and 38+0–38+6 weeks. This study aimed to determine gestational age distribution of spontaneous labor onset in twin pregnancies and evaluate whether findings support scheduling elective cesarean delivery at 37 rather than 38 weeks

**Study Design**: This retrospective cohort study included all live twin births ≥23+0 weeks between 1/2019 and 3/2025 at a high‐volume tertiary center. Only pregnancies with spontaneous labor onset were included. Cases involving labor induction, prelabor cesarean delivery, or intrauterine fetal demise were excluded. Data were extracted from electronic medical records. The primary outcome was gestational age at spontaneous labor onset.

**Results**: Among 1396 twin pregnancies with spontaneous labor onset, the mean gestational age at delivery was 35.9 ± 2.6 (median 36.6) weeks. The majority of spontaneous deliveries occurred before traditional elective timing: 812(58.2%) pregnancies delivered before 37+0 weeks, and 1175(84.2%) pregnancies delivered before 38+0 weeks. Only 221(15.8%) pregnancies were delivered at or after 38+0 weeks. Distribution by gestational age categories showed: 201(14.4%) births before 34 weeks, 611(43.8%) births at 34+0–36+6 weeks, 363(26.0%) births at 37+0–37+6 weeks, 175(12.5%) births at 38+0–38+6 weeks, and 46(3.3%) births at 39+ weeks. The peak incidence of spontaneous labor occurred between 35–37 weeks of gestation.

**Conclusion**: The vast majority(84.2%) of twin pregnancies with spontaneous labor onset deliver before 38+0 weeks, providing compelling evidence supporting elective cesarean delivery at 37+0 rather than 38+0 weeks. Scheduling at 37 weeks would prevent emergency interventions in 26.0% of pregnancies while affecting only 41.8% of the cohort. Waiting until 38 weeks would benefit only 15.8% while placing 84.2% at risk for unplanned delivery. This strongly supports earlier delivery timing and may reduce emergency rates and maternal‐fetal complications.

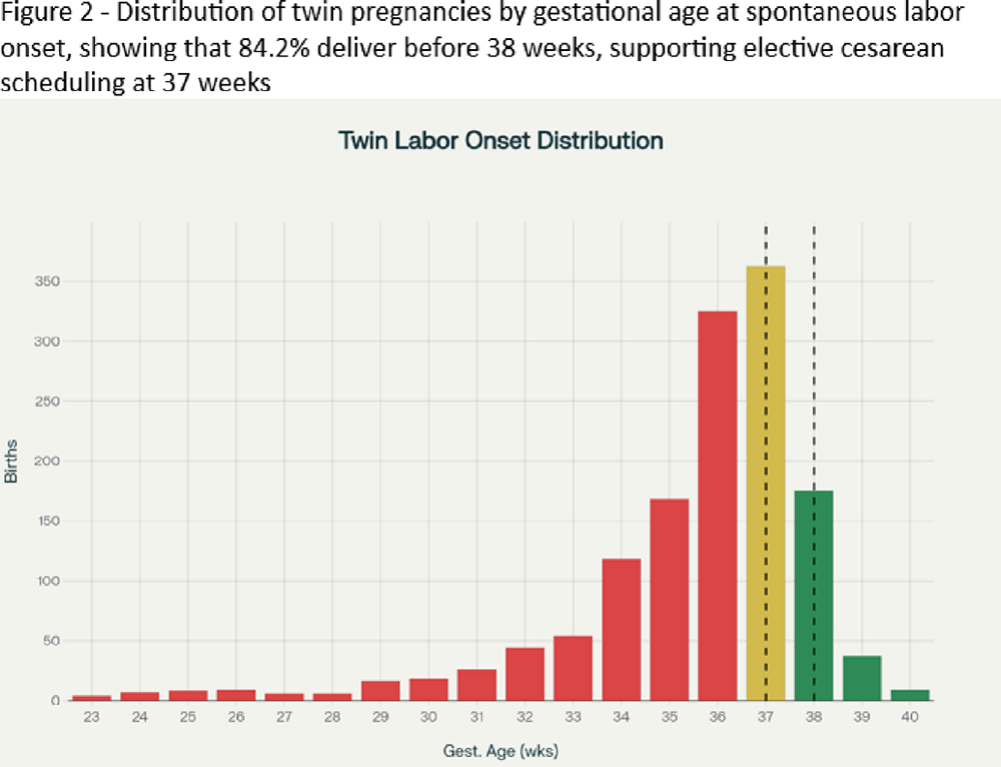

**3:30 PM – 5:00 PM**

## 642 | **Anesthetic** Experience **of Patients With Undergoing Cesarean Hysterectomy for Placenta Accreta Spectrum**

Rosa J. Speranza^1^; Luke A. Gatta^2^; Brett D. Einerson^3^; Jennifer B. Gilner^4^; Jaxon Olsen^5^; Amanda A. Allshouse^1^; Christine M. Warrick^1^; Laura Sorabella^2^; Ashraf S. Habib^5^; Naomi O. Riches^1^

^1^University of Utah, Salt Lake City, UT; ^2^Vanderbilt University, Nashville, TN; ^3^Department of Obstetrics and Gynecology, University of Utah Health, Salt Lake City, UT; ^4^Duke University School of Medicine, Durham, NC; ^5^Duke University, Durham, NC

**Objective**: To understand the patient experience with the three most common anesthetic modalities during cesarean hysterectomy for placenta accreta spectrum (PAS).

**Study Design**: We conducted a multi‐center qualitative study of English‐speaking participants with an antepartum concern for PAS managed by cesarean hysterectomy within the last 3 years. Participants were recruited into categories based on three anesthetic approaches: general anesthesia (GA) for both cesarean and hysterectomy; neuraxial anesthesia (NA) for cesarean followed by GA for hysterectomy; and NA for both cesarean and hysterectomy. Enrollment continued until thematic saturation was reached within each anesthetic cohort. Participants were interviewed by two trained researchers using a semi‐structured guide. Interviews were audio‐recorded, transcribed, and uploaded into qualitative software for coding. The study utilized a modified grounded theory and phenomenology approach to identify emergent themes and guide the analysis. Interviews were coded using a structured approach by two coders with high inter‐rate reliability (k >0.8). Patient demographics were compared by anesthetic modality.

**Results**: 22 patient interviews were conducted between 11/2022–7/2025. All patients were multiparous and had at least 1 prior cesarean section. Two cases were excluded from analysis due to an emergent delivery requiring GA. All other cases utilized their antepartum planned anesthetic modality. Of the analyzed cohort (20), 25% (5) had GA, 40% (8) had the combination of NA followed by GA, and 35% (7) had NA only. Baseline demographics were similar between anesthetic modalities (Table 1). Emergent themes focused on psychological feelings of control, normalization of the experience, birth preferences as well as physical concerns for the neonate's safety, duration of the procedure, and partner's participation (Table 2).

**Conclusion**: Understanding the lived experience with anesthesia of birthing people undergoing cesarean hysterectomy for PAS will help care teams provide more comprehensive counseling to guide shared decision making with regards to anesthesia modality.

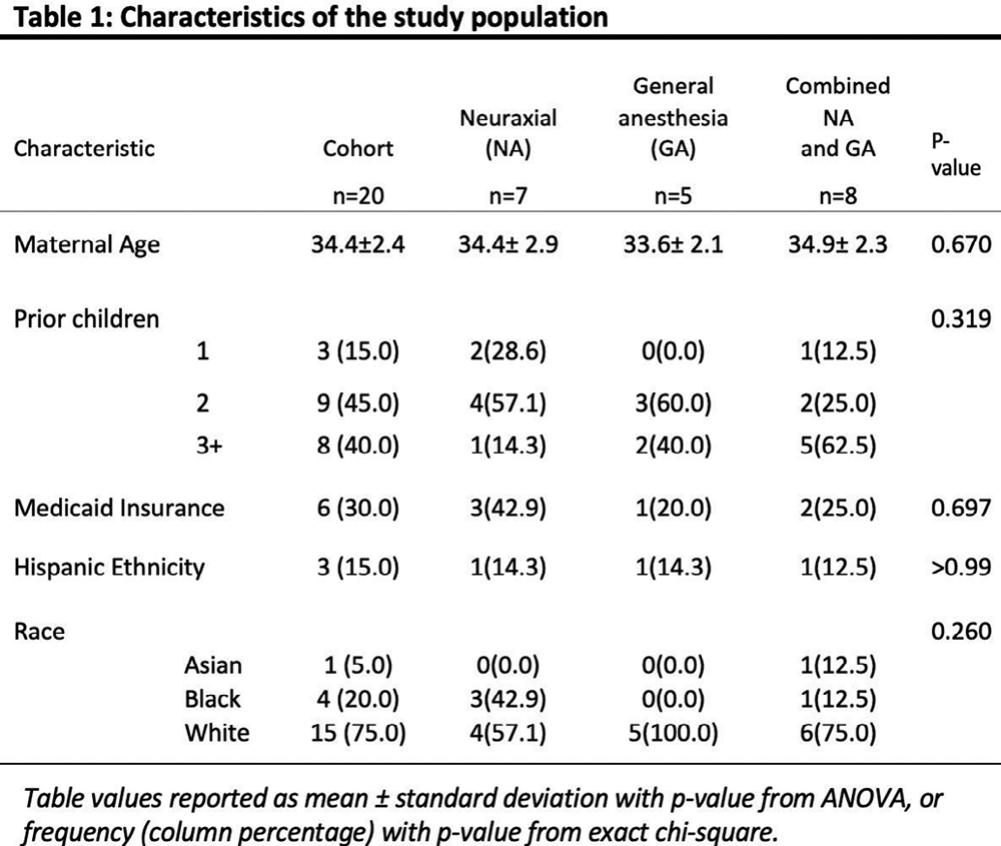

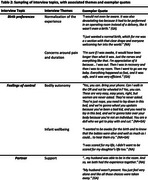

**3:30 PM – 5:00 PM**

## 643 | **Obstetric** Outcomes **Among Women Evacuated from Conflict Zones: A Multicenter Retrospective Cohort Study**

Roy Bitan^1^; Racheli Magnezi^2^; Uri Amikam^3^

^1^Lis Hospital for Women's Health, Tel Aviv, Tel Aviv; ^2^Department of Management, Health Systems Management Program, Bar Ilan University, Ramat Gan, Israel, Ramat Gan, HaMerkaz; ^3^Lis Hospital for Women's Health, Tel Aviv Sourasky Medical Center, Tel Aviv, Tel Aviv

**Objective**: To assess the impact of evacuation from conflict zones on obstetric and neonatal outcomes during a large‐scale armed conflict that commenced in Israel in October 2023.

**Study Design**: A retrospective cohort study was conducted using data from nine university‐affiliated hospitals in Israel. Deliveries between October 7, 2023, and April 7, 2024, were included. Women residing in government‐designated evacuation zones formed the study group, while women from non‐evacuated regions served as controls. Electronic medical records were reviewed, including maternal demographics, obstetric history, comorbidities, and obstetric and neonatal outcomes. Propensity score matching (1:3) was performed based on maternal age, socioeconomic status, parity, and the presence of chronic comorbidities.

**Results**: The total cohort included 14,017 deliveries, of which 321 (2.3%) were from evacuated zones. In the unmatched cohort, evacuated women had higher rates of pregestational diabetes mellitus and malignancy, and they were more likely to belong to lower socioeconomic groups and to identify as Jewish. Preterm birth (PTB) was more common in the evacuated group (7.2% vs. 4.9%, *p* = 0.058, respectively), approaching statistical significance. Postpartum hemorrhage (PPH) was significantly lower in the evacuated group (1.6% vs. 4.4%, *p* = 0.013, respectively). Other obstetric and neonatal outcomes did not differ significantly between the two groups. Following matching (288 evacuated vs. 864 controls), baseline characteristics were well‐balanced. The trend toward increased PTB in the evacuated group persisted (7.3% vs. 4.6%, *p* = 0.081, respectively). The incidence of PPH remained significantly less frequent in the evacuated group (1.4% vs. 4.5%, *p* = 0.015, respectively).

**Conclusion**: Evacuation during conflict was associated with a non‐significant trend toward an increased incidence of PTB and a significantly lower incidence of PPH. These findings emphasize the need for nuanced evaluation of maternal stress and health system adaptations during wartime displacement.

**3:30 PM – 5:00 PM**

## 644 | **Impact of** Azithromycin **Dose on Amniotic Fluid Cytokines in Preterm Premature Rupture of Membranes**

Rupsa C. Boelig^1^; Matthew K. Hoffman^2^; Amanda Roman^3^; Walter Kraft^3^

^1^Sidney Kimmel Medical College, Thomas Jefferson University, Philadelphia, PA; ^2^Christiana Care Health System, Newark, DE; ^3^Thomas Jefferson University, Philadelphia, PA

**Objective**: Azithromycin is an antibiotic with both bacteriostatic and immunomodulatory effect. It is used in various obstetric settings including preterm premature rupture of membranes (PPROM). The impact of azithromycin dosing regimen on amniotic fluid inflammation in the setting of PPROM is unclear. We aimed to compare inflammatory cytokines in amniotic fluid in patients with PPROM who received 1g once vs 500mg dailyx7 days azithromycin for PPROM.

**Study Design**: This is a planned secondary analysis of a prospective study of singletons with PPROM at two sites who received either 1g once or 500mg dailyx7d of azithromycin per site protocol. Amniotic fluid (AF) was collected opportunistically over a seven‐day period from admission, non‐invasively by extracting amniotic fluid from sanitary pads. ELISA assay was used to quantify pro‐inflammatory cytokines IL‐1a, IL‐1b, IL‐6, IL‐8, and TNFa, and anti‐inflammatory IL‐10. Log‐corrected values used for analysis; generalized estimated equations linear model was developed examining each cytokine, time from first dose, and dosing group. Two‐sided alpha = 0.05 used for all analyses.

**Results**: 16 (*N* = 7 with 1g once and *N* = 9 with 500 mg daily) participants with 109 amniotic fluid samples were included. Gestational age at enrollment was similar (28.2 ± 3.1 vs 29.9 ± 2.1 wks, *p* = 0.22). There were significantly higher levels of IL‐1a and TNFa (Figure) in those who had received 1g once azithromycin compared to 500mg daily, with similar trend observed for IL‐1b and IL‐6 (Table).

**Conclusion**: In patients admitted with PPROM, treatment with 1g azithromycin once was associated with higher inflammatory cytokines in amniotic fluid compared to those who received 500mg daily. Prospective randomized trial is warranted to evaluate the impact of azithromycin dosing regimen on infectious/inflammatory outcomes in the setting of PPROM.

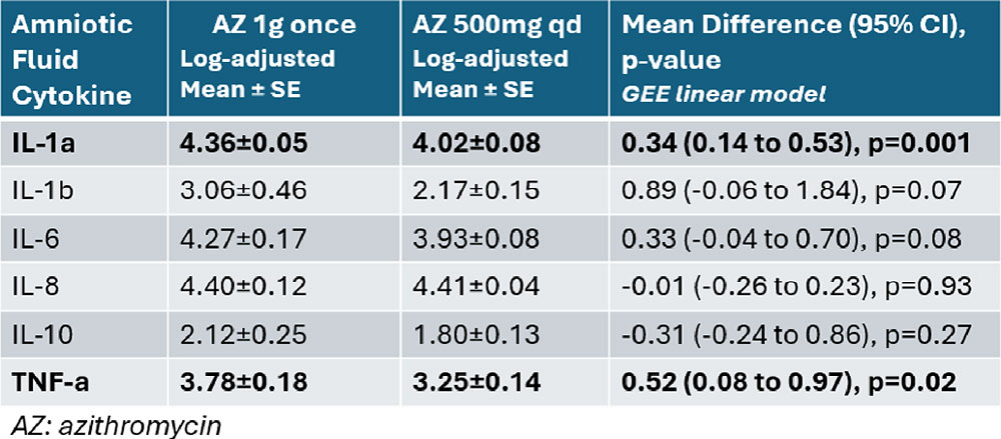

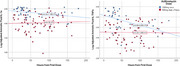

**3:30 PM – 5:00 PM**

## 645 | **Associations** Between **Social Disadvantage Indices and Hypertension in Pregnancy in the United States**

Ruth Esi Akyere Turkson^1^; Jill Coster^2^; Fredrick Dapaah‐Siakwan^3^

^1^St. Louis University, Southwest Illinois Family Medicine Residency, swansea, IL; ^2^St. Louis University, Southwest Illinois Family Medicine Residency, O'Fallon, IL; ^3^Valley Children's Hospital, Madera, CA

**Objective**: While individual‐level social determinants of health have been linked to hypertensive disorders in pregnancy, less is known about the relationship between community‐level social disadvantage and gestational hypertension.

To evaluate the association between four county‐level social disadvantage indices and gestational hypertension rates among U.S. live births. We hypothesized that higher levels of social disadvantage would correlate with higher rates of gestational hypertension.

**Study Design**: This was a retrospective, cross‐sectional study using county‐level gestational hypertension data from the CDC WONDER database (2018 to 2022). Four indices were evaluated: the Social Vulnerability Index (SVI), Social Vulnerability Metric (SVM), Social Deprivation Index (SDI), and Maternal Vulnerability Index (MVI). Each index captures dimensions of socioeconomic disadvantage using varying combinations of demographic, economic, housing, and health‐related indicators. Counties were grouped into quartiles from least to most disadvantaged for each index. Gestational hypertension rates per 1000 live births were compared between the most and least disadvantaged quartiles. Differences were considered statistically significant if 95% confidence intervals (CIs) did not overlap.

**Results**: Data from 570 counties were included, representing 77.7% of all U.S. live births. For all four indices (SVI, SVM, SDI, MVI), there were no statistically significant differences in gestational hypertension rates between the most and least disadvantaged quartiles. Confidence intervals overlapped across all comparisons.

**Conclusion**: Contrary to our hypothesis, county‐level social disadvantage was not associated with an increased rate of gestational hypertension.

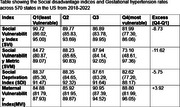

**3:30 PM – 5:00 PM**

## 646 | **Distance to** Extracorporeal **Membrane Oxygenation Access and Outcomes for Severe COVID‐19 in Pregnant/Peripartum Patients**

Ryan W. Wong^1^; Rachel G. Sinkey^1^; Amir A. Shamshirsaz^2^; Mark A. Turrentine^3^; Alison G. G. Cahill^4^; Joe Eid^5^; Caroline E. Rouse^6^; Sohum Shah^7^; Nandini Raghuraman^8^; Mariam Naqvi^9^; Yasser Y. El‐Sayed^10^; Martina Badell^11^; CeCe Cheng^12^; Robert M. Rossi^13^; Emily H. Adhikari^14^; Jennifer L. Thompson^15^; Erika O'Neil^16^; Emily Naoum^17^; Patrick S. Ramsey^18^; John J. Byrne^19^

^1^Center for Women's Reproductive Health Department of Obstetrics and Gynecology University of Alabama at Birmingham, Birmingham, AL; ^2^Division of Maternal‐Fetal Medicine, Department of Obstetrics and Gynecology, Baylor College of Medicine, Houston, TX; ^3^Department of Obstetrics and Gynecology Baylor College of Medicine, Houston, TX; ^4^Department of Women's Health, Dell Medical School at the University of Texas at Austin, Department of Women's Health, Dell Medical School at the University of Texas at Austin, TX; ^5^Department of Obstetrics and Gynecology The Ohio State University College of Medicine, Columbus, OH; ^6^Department of Obstetrics and Gynecology Division of Maternal‐Fetal Medicine Indiana University School of Medicine, Indianapolis, IN; ^7^Division of Maternal Fetal Medicine, Department of Obstetrics and Gynecology, David Geffen School of Medicine at University of California, Los Angeles (UCLA), Los Angeles, CA; ^8^Washington University School of Medicine, St. Louis, MO; ^9^Department of Obstetrics and Gynecology, Cedars‐Sinai Medical Center, Los Angeles, CA; ^10^Department of Obstetrics and Gynecology Division of Maternal‐Fetal Medicine and Obstetrics Stanford University, Palo Alto, CA; ^11^Department of Obstetrics and Gynecology Division of Maternal‐Fetal Medicine Emory University School of Medicine, Atlanta, GA; ^12^Department of Obstetrics and Gynecology Methodist Hospital, San Antonio, TX; ^13^Department of Obstetrics and Gynecology Division of Maternal‐Fetal Medicine University of Cincinnati College of Medicine, Cincinnati, OH; ^14^University of Texas Southwestern Medical Center, Dallas, TX; ^15^Vanderbilt University Medical Center, Nashville, TN; ^16^Department of Pediatrics Brooke Army Medical Center, San Antonio, TX; ^17^Department of Anesthesia Critical Care and Pain Medicine Massachusetts General Hospital, Boston, MA; ^18^Department of Obstetrics and Gynecology University of Texas Health Science Center in San Antonio, San Antonio, TX; ^19^University of Texas Health Science Center in San Antonio, San Antonio, TX

**Objective**: Increased driving distance to maternity hospitals has been associated with higher risk of adverse maternal/perinatal outcomes; however, the association between distance and outcomes among pregnant patients requiring ECMO is unknown. We evaluated the association between distance from patients’ homes to the tertiary center from which they received ECMO to investigate whether distance was associated with adverse outcomes.

**Study Design**: Secondary analysis of a national 25‐center COVID‐19 ECMO consortium, 3/1/20–10/1/22. All cohort subjects, *N* = 100, were included. Exposure was the distance between patients’ homes & their tertiary centers, based on zip codes. The primary outcome was a serious maternal morbidity composite that included: death, venous thromboembolism (VTE), ischemic stroke or intracranial hemorrhage, acute kidney injury, gastrointestinal bleeding, liver failure, non‐cardiac/non‐intracranial ischemic injuries, and any one of several possible cardiac complications. Secondarily, we examined components of the composite and selected obstetric and neonatal outcomes. Logistic regression modeled the association between distance and the odds of selected outcomes.

**Results**: Patient characteristics are shown in Table 1. ECMO was initiated during pregnancy, within 24 hours postpartum, and 24 hours –6 weeks postpartum among 47%, 21% and 32% of patients. Mean ± SD (median) distance from home to the ECMO center was 65 ± 113 (31) miles. The primary composite outcome occurred in 76%, and there was a null association (*p* = 0.70) between distance and composite morbidity. Maternal death (16%) was also not associated with distance (*p* = 0.22). The most common component of the composite was VTE, 39%. Associations of distance with individual components of the composite are in Table 2.

**Conclusion**: In a multicenter study of severe COVID‐19 cases in pregnancy/postpartum, distance from a patient's home to the ECMO center was not associated with increased odds of selected adverse outcomes. Available resources appeared to have overcome geographic obstacles in patients who received ECMO for severe COVID‐19.

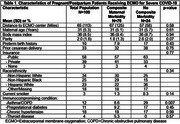

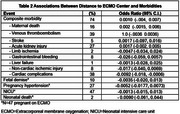

**3:30 PM – 5:00 PM**

## 647 | **Impact of Sickle** Cell **Disease Genotype on Severe Maternal Morbidity and Readmission**

Salimah Navaz Gangji^1^; Grace Spencer^2^; Tetsuya Kawakita^3^

^1^Eastern Virginia Medial School at Old Dominion University, Norfolk, VA; ^2^Eastern Virginia Medical School at Old Dominion University, EVMS OBGYN, Virginia Health Sciences at Old Dominion University, VA; ^3^Eastern Virginia Medical School Department of Obstetrics and Gynecology, Macon & Joan Brock Virginia Health Sciences at Old Dominion University, Norfolk, VA

**Objective**: To examine the maternal outcomes according to sickle cell disease (SCD) genotypes.

**Study Design**: This was a retrospective cohort study using the Healthcare Cost and Utilization Project (HCUP) National Readmissions Database from 2018 to 2022. Patients were categorized by genotype: no SCD, sickle cell trait, hemoglobin SS (SS) disease, hemoglobin SC (SC) disease, and sickle‐thalassemia. The primary outcome was CDC‐defined severe maternal morbidity (SMM), excluding sickle cell crisis and transfusion. Secondary outcomes included postpartum readmission within 42 days, transfusion, and sickle cell crisis. Modified Poisson regression was used to calculate adjusted relative risks (aRR) with 95% confidence intervals (CI), adjusting for relevant confounders.

**Results**: Among 7,990,355 patients, 55,863 (0.7%) had sickle cell trait, 4958 (0.06%) had SS disease, 585 (0.01%) had SC disease, and 760 (0.01%) had sickle‐thalassemia. Maternal demographics, comorbidities, and pregnancy complications varied across genotypes (Table 1). Compared to patients without SCD (0.9%), rates of SMM were higher in those with sickle cell trait (1.6%; aRR 1.44, 95% CI 1.35–1.54), SS disease (6.7%; aRR 5.86, 95% CI 5.28–6.51), SC disease (7.9%; aRR 5.58, 95% CI 4.19–7.45), and sickle‐thalassemia (4.1%; aRR 3.77, 95% CI 2.67–5.33) (Table 2). Similar trends were observed for readmission and transfusion. Compared to SS disease (16.5%), the risk of sickle cell crisis was higher in SC disease (21.7%; aRR 1.29, 95% CI 1.10–1.52) and lower in sickle‐thalassemia (10.4%; aRR 0.68, 95% CI 0.55–0.85).

**Conclusion**: Maternal outcomes vary significantly by SCD genotype. While all genotypes were associated with increased risk of SMM, patients with SC disease had the highest risk despite historically being considered less severe. Sickle cell trait, though often viewed as benign, was also associated with excess risk. These findings underscore the need for genotype‐specific risk counseling, close monitoring, and individualized care pathways during pregnancy.

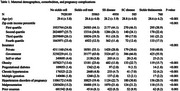

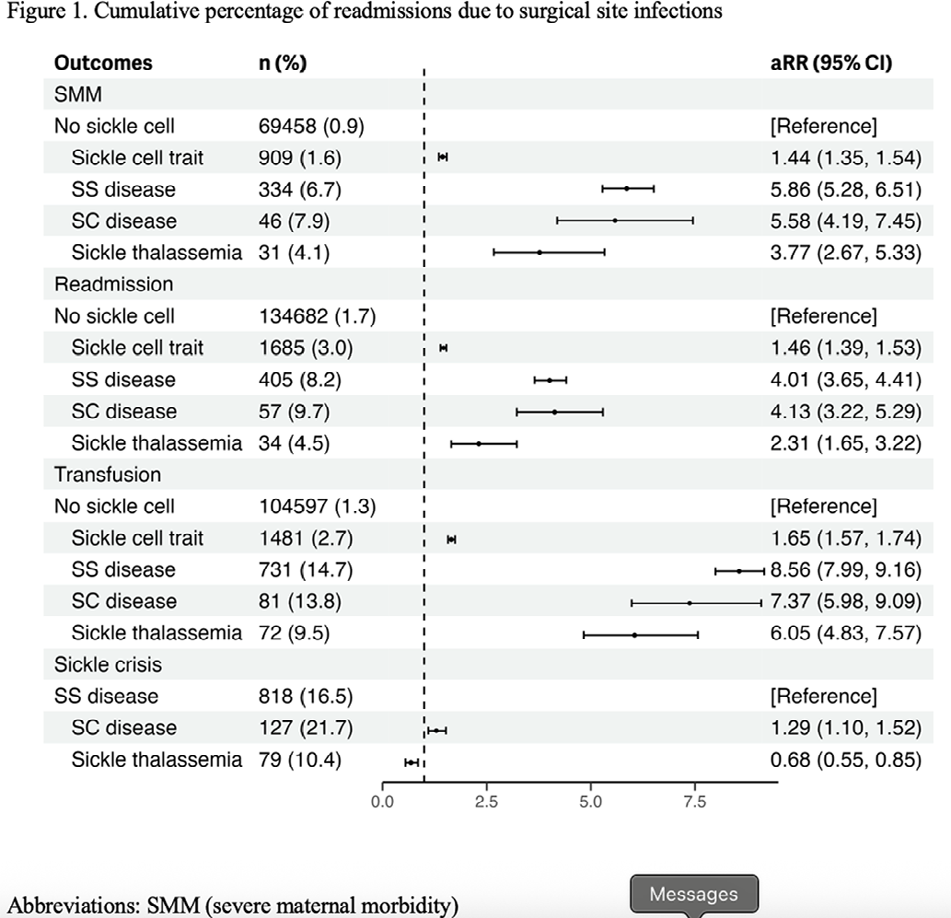

**3:30 PM – 5:00 PM**

## 648 | **Device‐Based** Measures **Of Postprandial Physical Activity and Glucose in Individuals With Pregnancy Hyperglycemia**

Samantha F. Ehrlich^1^; Jordan Lewis^1^; Sara Yousefi^1^; Bethany Hallenbeck^2^; Hollie Raynor^1^; Scott Crouter^1^; Izaak Miller^1^; Nikki B. Zite^3^; Walter Schoutko^4^; Kimberly B. Fortner^5^; Jill M. Maples^6^

^1^The University of Tennessee, Knoxville, Knoxville, TN; ^2^Kaiser Permanente Northern California, Pleasanton, CA; ^3^University of Tennessee Graduate School of Medicine, Knoxville, TN; ^4^The University of Tennessee Graduate School of Medicine, Knoxville, TN; ^5^University of Tennessee College of Medicine‐ Knoxville, Knoxville, TN; ^6^University of Tennessee Health Science Center COM Knoxville, Knoxville, TN

**Objective**: Physical activity (PA) following meals is recommended for individuals with pregnancy hyperglycemia as a non‐pharmacological strategy to manage postprandial glucose levels. The objective of the current study is to investigate this association specifically at the meal level, under free‐living conditions and using repeated, device‐based measures of both PA and glucose.

**Study Design**: Fourteen subjects with gestational glucose intolerance (GGI) and 5 with gestational diabetes (GDM, not requiring insulin) participating in the Time to Move Randomized Crossover Trial (ClinicalTrials.gov #NCT06125704) provided data on 478 meals. Participants uploaded before and after photos of all eating and drinking episodes during the study period, designating them as a meal or snack; the timestamps on the before photos were used to identify the start times of all meals. Participants also wore Dexcom continuous glucose monitoring (CGM) devices and Actigraph CentrePoint Insight Watch PA monitoring devices. The Two‐Regressions R package provided minute‐by‐minute estimates of the metabolic equivalent of task (MET). The associations of PA accumulated during the 1‐hr postprandial period [i.e., total MET‐minutes, dichotomized at the 75^th^ percentile] with 2‐hr postprandial glucose metrics [i.e., mean and area under‐the‐curve (AUC)] were estimated with Proc Mixed in SAS v9.4, adjusting for GGI versus GDM.

**Results**: PA accumulated during the 1‐hr postprandial period at or above the 75^th^ percentile was associated with 3.6 mg/dl (SE 1.6) lower 2‐hr postprandial mean glucose (*p* = .04), and 34787 (SE 12133) lower 2‐hr postprandial glucose AUC (*p* = .01; results of the repeated measures analyses are presented in the Table). The 75^th^ percentile for PA accumulated during the 1‐hr postprandial period was 111.15 MET‐minutes, which corresponds to a threshold of approximately 37 minutes of moderate intensity walking.

**Conclusion**: PA accumulated during the 1‐hr postprandial period modestly reduces CGM assessed 2‐hr postprandial glucose in individuals with pregnancy hyperglycemia.

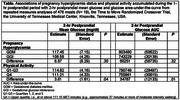

**3:30 PM – 5:00 PM**

## 649 | **Geopolitical** Variation **in MFM Fellowship Website Diversity, Equity, and Inclusion (DEI) Elements**

Samantha Gobioff^1^; Zenobia Gonsalves^2^; Kimberly Herrera^3^; Cassandra Heiselman^3^

^1^Stony Brook University Hospital, NY; ^2^Stony Brook University Hospital, Great Neck, NY; ^3^Stony Brook University Hospital, Stony Brook, NY

**Objective**: To determine if DEI elements represented on MFM fellowship websites differ based on state diversity and legislation.

**Study Design**: This was a cross‐sectional analysis in July of 2025 of DEI represented on ACGME accredited MFM fellowship websites. Programs that had only initial accreditation and military programs were excluded, leaving 100 evaluable. 11 Diversity Elements (DEs), as defined by Winget et al. (AJOG, 2023), were collected from the individual program websites. To indirectly assess population diversity, state Diversity Index (DI) and Diffusion Score (DS) were collected from the 2020 U.S. Census Bureau data. Higher DI and DS (scale 0–100) scores represent higher population diversity and less concentration in the top three racial/ethnic groups, respectively. The presence of anti‐DEI laws (aDEI‐L) in each state was collected. Represented DEs were compared according to corresponding DI, DS, and aDEI‐L, using Chi‐square, Fisher's exact, Kruskal‐Wallis, Mann‐Whitney U test, and Spearman's correlations, with a *p*‐value <0.05.

**Results**: The total number of DEs on websites was not correlated with DI (*r* = 0.095, *p* = 0.35), DS (*r* = 0.121, *p* = 0.23), or aDEI‐L (Mdn[IQR]: 4[2,5] vs. 4[3,5], *p* = 0.44). There was no difference in DI scores with any specific DE or with having ≥6 total elements (Figure 1). DS scores were only associated with the DE “Diversity Specific Language” (10.8[7.9, 14.3] vs. 8.6[7.0, 10.8], *p* = 0.015). When compared to states without aDEI‐L, states with active aDEI‐L had significantly lower representation of “Diversity Specific Language” at 22.2% vs. 47.9% (*p* = 0.02) and “Diversity Inclusion Message” at 14.8% vs. 35.6% (*p* = 0.04). States with aDEI‐L had a higher rate of “Diversity Publications” (51.9%) compared to those states without aDEI‐L (27.4%, *p* = 0.02) (Table 1).

**Conclusion**: Most DEI element representation did not differ among MFM fellowship websites based on state diversity score variations, however the presence of state anti‐DEI laws did affect the presence of specific DEI elements.

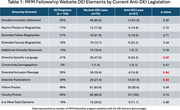

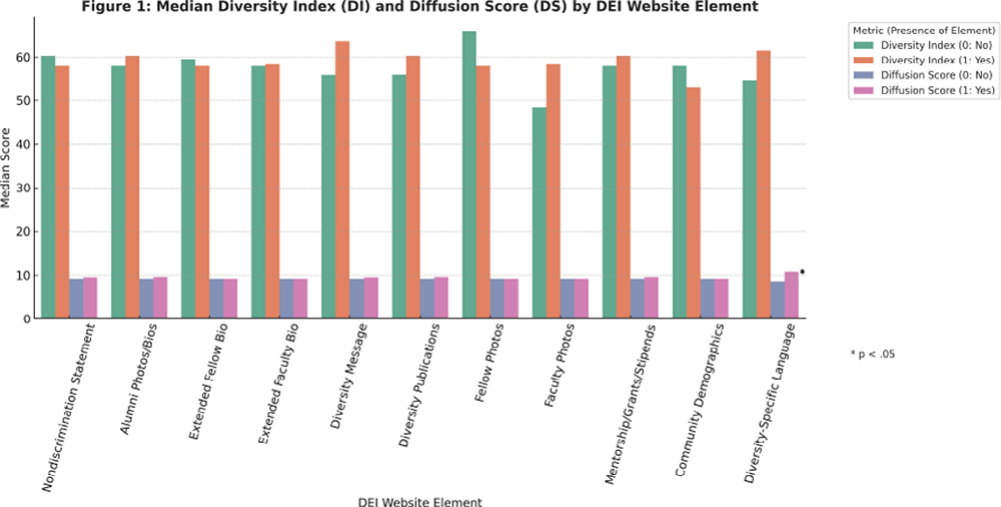

**3:30 PM – 5:00 PM**

## 650 | **Are Social** Determinants **of Health Associated With Vaccine Uptake during Pregnancy?**

Sandhya Chandrasekaran^1^; Stephanie Schreiber‐Gonzalez^2^; Ashish Premkumar^1^; Beth Plunkett^3^; Rachel Ruderman^4^

^1^University of Chicago, Chicago, IL; ^2^Ascension Healthcare, Clarendon Hills, IL; ^3^Endeavor Health Evanston, Evanston, IL; ^4^Endeavor Health, Chicago, IL

**Objective**: Social determinants of health (SDOH) are increasingly recognized as critical to vaccine uptake. Therefore, we sought to evaluate the association of socioeconomic deprivation, measured through area deprivation indices (ADIs), with the uptake of the four recommended vaccinations in pregnancy: tetanus‐diptheria‐acellular pertussis (Tdap), Influenza, coronavirus‐19 (COVID), and respiratory syncytial virus (RSV) vaccines.

**Study Design**: This was a retrospective observational study among pregnant patients in a large, urban medical system. Patients were included if they were between 32 and 36 weeks’ gestation during the study period–hence eligible for all vaccinations. Exclusion criteria included documented allergy to prior vaccination or pregnancy affected by a lethal fetal anomaly. Relative socioeconomic conditions of patients were defined using ADI, whereby, on a scale of 1 to 10, a higher score indicates a more deprived area, and a lower score indicates a more affluent area. Vaccination rates and ADI were abstracted from the electronic medical record and quality checked for accuracy. Kruskal‐Wallis testing with post‐hoc analyses (Dunn's test with Bonferroni correction) was performed to determine statistical significance among groups.

**Results**: 545 individuals met inclusion criteria. The median age of patients included was 33.9 (30.4 to 37.2). 38.7% were obese, and 44% self‐identified as non‐Hispanic Black (Table 1). The median ADI across the study population was 4. Statistically significant differences were observed across all combinations of vaccine uptake (*p* < 0.001, ***), with post‐hoc analyses most notable for a higher ADI in patients receiving Tdap as compared to patients accepting all four vaccines (*p* < 0.001, ***; Figure 1).

**Conclusion**: ADI is significantly associated with the types and numbers of vaccines patients receive in pregnancy, with a general trend toward higher ADI in groups receiving no vaccines and lower ADI in groups receiving all or most vaccines. Complementary qualitative or mixed methods studies will further elucidate barriers to vaccine uptake and motivate appropriate interventions.

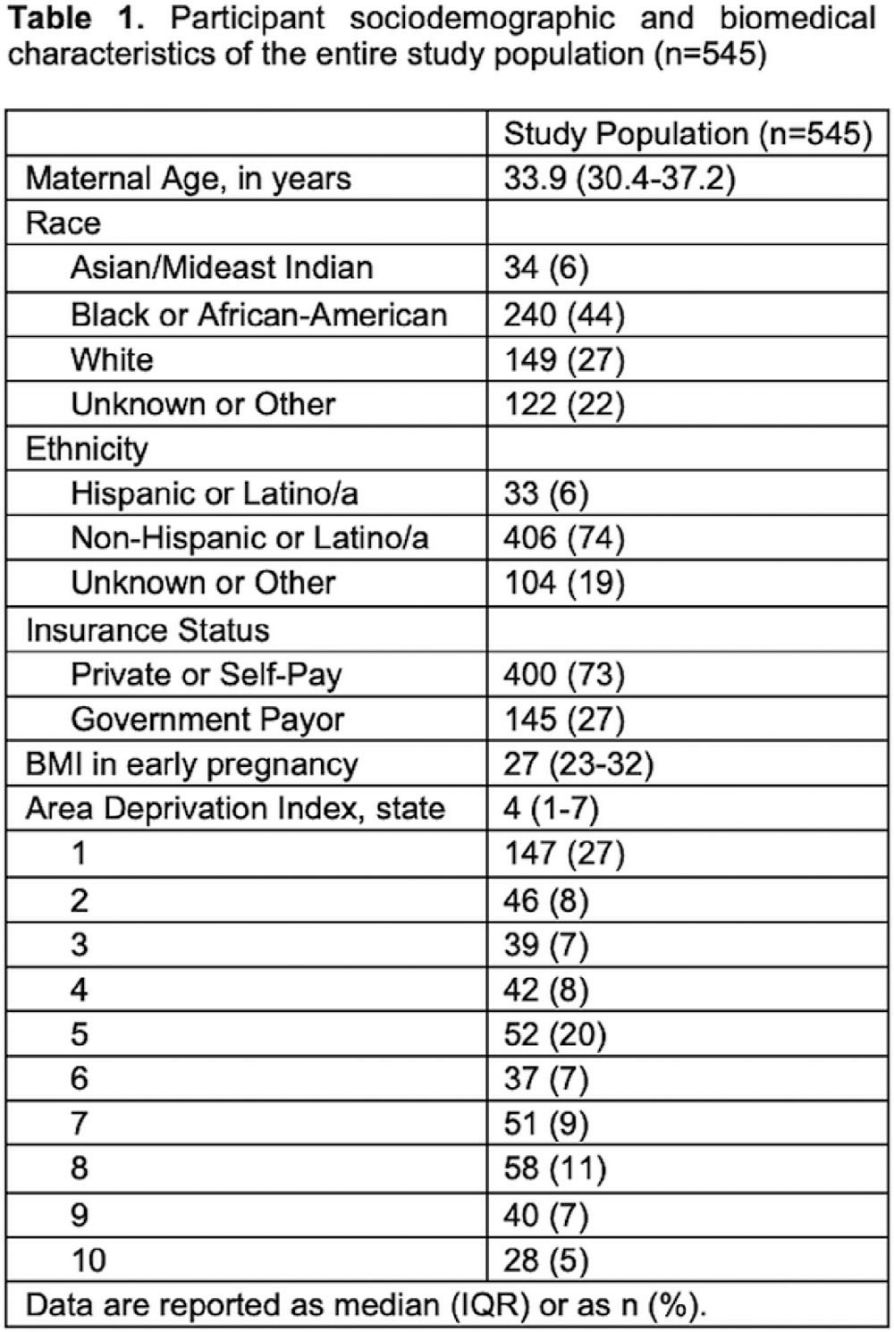

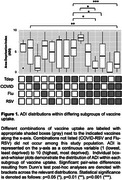

**3:30 PM – 5:00 PM**

## 651 | **Maternal Asthma and Childhood Respiratory Risk: Evidence from a Longitudinal Cohort Study**

Sapir Ellouk^1^; Tamar Wainstock^2^; Eyal Sheiner^3^

^1^Department of Obstetrics and Gynecology, Soroka University Medical Center, Ben‐Gurion University, HaDarom; ^2^Department of Epidemiology, Biostatistics and Community Health Sciences, Faculty of Health Sciences, Ben‐Gurion University of the Negev, Beer Sheva, HaDarom; ^3^Soroka University Medical Center, Faculty of Health Sciences, Ben‐Gurion University of the Negev, beer sheva, HaDarom

**Objective**: Maternal asthma has been linked to a heightened risk of various obstetric complications. Growing evidence indicates that the impact of maternal asthma may extend beyond pregnancy, potentially influencing long‐term health outcomes in children. This study aimed to investigate whether offspring who were born to mothers with asthma are at an increased risk of long‐term respiratory morbidity.

**Study Design**: A population‐based cohort study evaluated long‐term respiratory morbidity in offspring of mothers with and without asthma, using data from community clinics and a tertiary medical center between 1991 and 2021. Multiple gestations and congenital malformations were excluded. Kaplan–Meier analysis estimated cumulative incidence, and Cox proportional hazards model was used to adjust for confounders.

**Results**: The analysis included 232,094 pregnancies, 2875 (1.2%) involving mothers diagnosed with asthma. Children born to mothers with asthma exhibited a significantly higher rate of respiratory morbidity compared to those born to non‐asthmatic mothers (48.7% vs. 34.3%; OR = 1.8, *p* < 0.001). Increased incidences of asthma, obstructive sleep apnea (OSA), and bronchiectasis were particularly notable in the exposed group (Table). Kaplan‐Meier survival analysis revealed a significantly higher cumulative incidence of respiratory morbidity among offspring of asthmatic mothers (log‐rank *p* < 0.001; Figure). Even after adjusting for potential confounders—including maternal age, gestational diabetes, cesarean delivery, hypertension, and gestational age—maternal asthma remained an independent risk factor for offspring respiratory morbidity (adjusted HR = 1.5; 95% CI, 1.42–1.58; *p* < 0.001).

**Conclusion**: Maternal asthma independently contributes to an increased risk of long‐term respiratory morbidity in offspring. Further investigation is needed to clarify the biological mechanisms through which maternal asthma may influence fetal development and long‐term respiratory health.

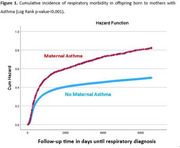

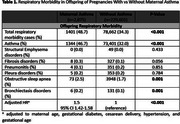

**3:30 PM – 5:00 PM**

## 652 | **Impact of Major** Local **Natural Disasters on the Prevalence of Perinatal Sexually Transmitted Infections**

Sara I. Jones^1^; Lillian Boettcher^2^; Hannah Kelly^1^; Chun Xu^3^; Tracy Truong^3^; Elizabeth Livingston^3^; Sarah K. Dotters‐Katz^4^

^1^Duke University School of Medicine, Durham, NC; ^2^Department of Obstetrics and Gynecology, Duke University School of Medicine, Duke University School of Medicine/Durham, NC; ^3^Duke University Medical Center, Durham, NC; ^4^Division of Maternal Fetal Medicine, Department of Obstetrics and Gynecology, Duke University, Duke University/Durham, NC

**Objective**: To identify the impact of local natural disasters on the prevalence of sexually transmitted infections in pregnant patients at delivery.

**Study Design**: We performed an interrupted time series analysis (ITS) of patients who delivered between 2010 and 2019 using the Centers for Disease Control and Prevention Natality Database. Patients were included if they resided in a state with at least one county affected by a Major Disaster Declaration of a hurricane, earthquake, or fire for at least 1 week, as recorded by the Federal Emergency Management Agency with public assistance requested. We used an ITS quasi‐Poisson regression model to estimate the monthly prevalence of active syphilis, chlamydia, and gonorrhea infection at delivery and assess trends over the year before and after each disaster type. The primary outcome was difference between observed and counterfactual (projected assuming the disaster had not occurred) rates at 1 month post‐disaster for each infection. Models were adjusted for maternal age, categorical BMI, race, ethnicity, insurance, education status, marital status, and United States region.

**Results**: 15,448,550 deliveries were evaluated from 25 states experiencing a Major Disaster Declaration for a hurricane, earthquake, or fire between 2010 and 2019. At 1 month following the disaster, syphilis prevalence was higher than expected after a major fire (adjusted rate ratio [aRR] 1.16; 95% CI:1.05,1.29; *p* = 0.006) and hurricane (aRR 1.10; 95% CI:1.00,1.21; *p* = 0.047) (Figure 1). At the same time point, the observed prevalence of chlamydia was also higher than expected following a major fire (aRR 1.04; 95% CI 1.01–1.06; *p* = 0.015) (Figure 2). No statistically significant differences were seen for rates of gonorrhea following any disaster type.

**Conclusion**: The prevalence of new perinatal sexually transmitted infection increases following local natural disasters, exceeding rates expected had the disaster not occurred. This information may be useful to guide post‐disaster resource allocation, screening, and advocacy for climate action.

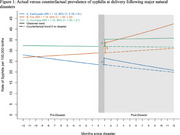

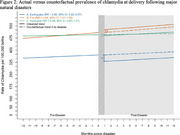

**3:30 PM – 5:00 PM**

## 653 | **Association** Between **Oxytocin Rest and Mode of Delivery in Low‐Risk Nulliparas at Term**

Sara E. Post; Amanda A. Allshouse; Torri D. Metz; Ann M. Bruno

University of Utah, Salt Lake City, UT

**Objective**: “Oxytocin rest” is a practice of temporarily discontinuing oxytocin during labor when a regular contraction pattern has not been achieved to allow for myometrial oxytocin receptor re‐sensitization. Data are lacking on the clinical utility of this practice. We aimed to evaluate the association between oxytocin rest and mode of delivery and maternal outcomes.

**Study Design**: This was a secondary analysis of the MFMU ARRIVE trial, a multi‐center randomized controlled trial of induction versus expectant management in low‐risk term nulliparas. Participants who received oxytocin in labor were included in this analysis. The exposure was oxytocin rest, defined as a stop in oxytocin during labor. The primary outcome was mode of delivery. Secondary outcomes were chorioamnionitis, maximum cervical dilation achieved, and a composite maternal morbidity. Multivariable modeling estimated the association between oxytocin rest and outcomes with odds ratios and 95% confidence intervals reported. A sensitivity analysis excluded those with cesarean for non‐reassuring fetal status.

**Results**: Of 3257 participants included, 365 (11%) experienced an oxytocin rest with a median duration of 1.4 hours (interquartile range 0.7–3.0 hours) and most (61%) occurring in the latent phase (*n* = 224). Baseline characteristics differed by oxytocin rest (Table 1). Oxytocin rest was associated with higher odds of cesarean delivery (37% vs. 22.6%; aOR 1.83, 95% CI 1.44–2.32), and decreased odds of reaching 5 or 10 cm dilation (Table 2). Oxytocin rest was not associated with maternal morbidity. Findings were similar in the sensitivity analysis.

**Conclusion**: In low‐risk nulliparas who received oxytocin, undergoing an oxytocin rest was associated with cesarean delivery, and decreased odds of achieving active labor or complete dilation. Findings may reflect that oxytocin rest was used more often in those with dysfunctional labor without improving chances of vaginal delivery. Prospective study is needed to better elucidate potential benefits or risks of oxytocin rest.

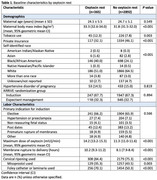

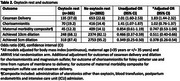

**3:30 PM – 5:00 PM**

## 654 | **Anemia and** Maternal**‐Newborn Dyadic Birth Outcomes: A Desirability of Outcome Ranking (DOOR) Analysis**

Sarah H. Abelman; Frank I. Jackson; Jamie Green; Oladunni Ogundipe; Matthew J. Blitz

Northwell, New Hyde Park, NY

**Objective**: To evaluate the association between maternal anemia and maternal‐newborn dyadic outcomes using the desirability of outcome ranking (DOOR) methodology, which orders outcomes by clinical desirability.

**Study Design**: This retrospective cross‐sectional study included all deliveries eligible for the Joint Commission's PC‐06 metric (term newborns without preexisting conditions) across seven hospitals in a New York health system (2019–2024). Only each patient's first delivery was included. Anemia was categorized as none (Hb ≥11.0 g/dL), mild (10.0–10.9), or moderate‐to‐severe (<10.0). Outcomes were classified into four DOOR categories: (1) uncomplicated vaginal delivery, (2) uncomplicated cesarean, (3) vaginal delivery with complications, and (4) cesarean with complications. Maternal and neonatal complications were defined by CDC severe maternal morbidity and PC‐06 criteria. Multinomial logistic regression assessed associations between anemia and DOOR, adjusting for obstetric comorbidity index (OB‐CMI) score, race and ethnicity, multiparity, insurance, and language.

**Results**: A total of 105,156 deliveries were included. Birth outcomes by anemia severity are shown in the Figure; regression results are presented in the Table. Worsening anemia severity was strongly associated with less desirable outcomes. Compared to no anemia, mild anemia was associated with higher odds of vaginal delivery with complications (aOR 1.49; 95% CI, 1.34–1.65) and cesarean with complications (aOR 2.13; 95% CI, 1.93–2.34). Risks were greater for moderate‐to‐severe anemia: 3.49 times higher odds of vaginal delivery with complications (95% CI, 3.10–3.92) and 5.86 times higher odds of cesarean with complications (95% CI, 5.30–6.49). A dose‐response pattern was observed across all categories.

**Conclusion**: Maternal anemia is a strong, independent predictor of adverse maternal‐newborn outcomes. Increasing severity is linked to significantly worse DOOR outcomes, underscoring the need for early detection and intervention.

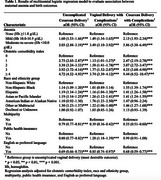

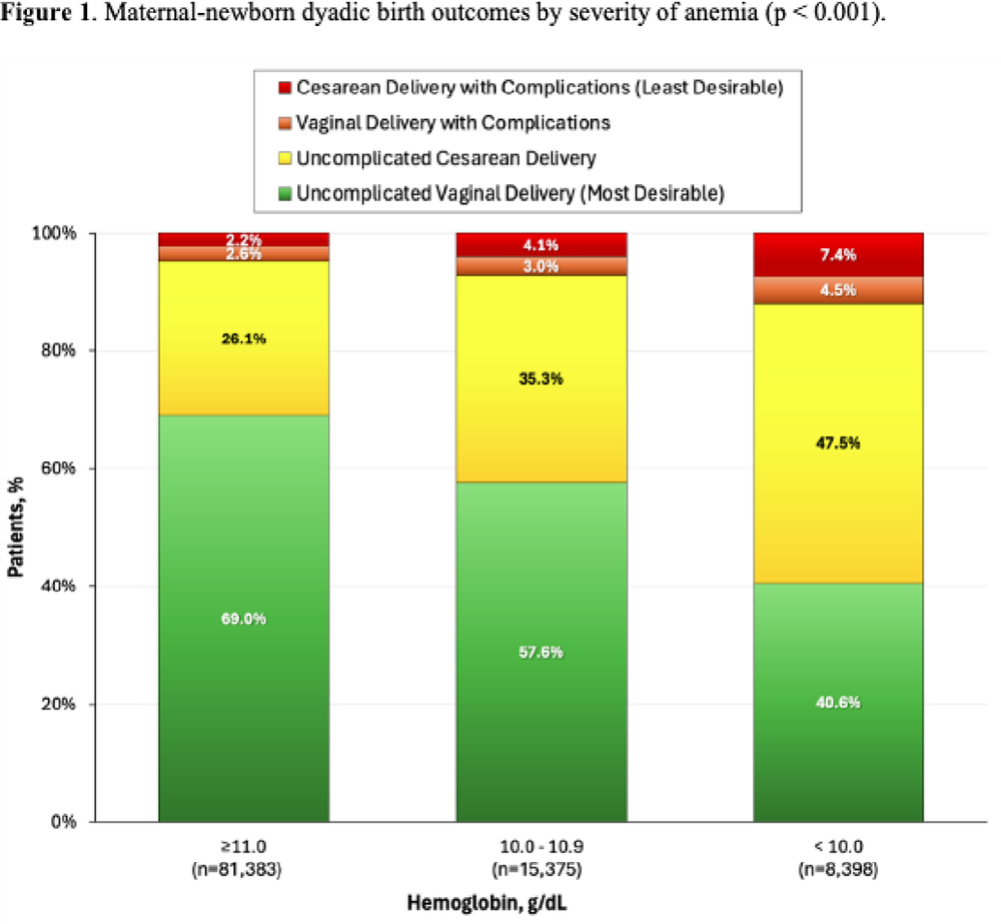

**3:30 PM – 5:00 PM**

## 655 | **Enhancing** Obstetric **Team Readiness for Maternal Cardiac Arrest Through Simulation‐Based, Longitudinal Defibrillator Education**

Sarah E. Detlefs^1^; Tara Barrick^2^

^1^Baylor College of Medicine, Baylor College of Medicine, TX; ^2^Texas Children's Hospital, Texas Children's Hospital, TX

**Objective**: Maternal cardiac arrest is a rare but critical emergency requiring rapid recognition and coordinated response to optimize maternal and fetal survival. Despite widespread Basic Life Support (BLS) certification, institutional preparedness varies. This quality improvement project aimed to improve obstetric team competency in defibrillator use through a structured educational intervention.

**Study Design**: This was a longitudinal IRB‐approved project from June 2023 to May 2025. The educational intervention included: (1) online defibrillator training; (2) hands‐on skill stations covering defibrillator and code cart use; and (3) team‐based simulation emphasizing high‐quality CPR, defibrillator pad placement, and delivery of a shock for a shockable rhythm. Staff completed an objective BLS skills assessment, with responses scored using a 5‐point Likert scale. Assessments were completed prior to initial training in 2023 and prior to annual education in 2025 to evaluate baseline knowledge and retention over time. Performance in each measure was classified as “Proficient” (scores 4–5) or “Not Proficient” (scores 1–3); proportions were compared using chi‐square analysis.

**Results**: Among 272 staff surveyed (58 in 2023, 214 in 2025), no significant differences were observed in assessing responsiveness, breathing, pulses; performing compressions; or providing rescue breaths (Table 1). Significant improvements occurred in defibrillator‐specific skills: correct pad placement (37.9% vs. 77.1%, *p* < 0.001), clearing before shock (74.1% vs. 92.5%, *p* < 0.0001), and safe shock delivery (81.0% vs. 93.9%, *p* = 0.007).

**Conclusion**: A structured educational intervention significantly improved staff readiness for maternal cardiac arrest and defibrillator use. Nonetheless, 22% of staff in 2025 remained unable to correctly place defibrillator pads, suggesting knowledge decay and the impact of staff turnover. These findings underscore the need for ongoing, repetitive training to maintain competency in managing high‐acuity, low‐frequency events such as maternal cardiac arrest.

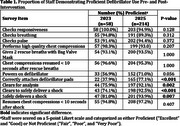

**3:30 PM – 5:00 PM**

## 656 | **Utility of Routine Hemoglobin Assessment Following Vaginal Delivery**

Sarah M. Fujiwara^1^; Andrea Siu^2^; Kelly Yamasato^3^

^1^Hawaii Pacific Health, Honolulu, HI; ^2^Hawaii Pacific Health, Hawaii Pacific Health, HI; ^3^Hawaii Pacific Health Medical Group, Hawaii Pacific Health Medical Group, HI

**Objective**: Universal postpartum (PP) hemoglobin (hgb) testing is standard in many hospitals with the intent of identifying clinically significant anemia. However, evidence for this is lacking. We assessed the utility of PP hgb following vaginal delivery with normal blood loss through frequency of blood and intravenous (IV) iron administrations in this population.

**Study Design**: This retrospective cohort study included vaginal deliveries at a tertiary hospital between January 2020–May 2025. Exclusions were (1) blood loss (estimated or quantitative) >500 mL or unknown, (2) preeclampsia/eclampsia, and (3) hemorrhage diagnosis. Predelivery and PP hgb and blood and IV iron transfusions were collected. Multivariable logistic regression was used to identify predictors of blood transfusion. Manual chart review was done for those receiving blood or IV iron to identify anemia symptoms and other comorbidities.

**Results**: Of 20,993 vaginal deliveries, 11,180 were included. Of these 10,075 (90.1%) had a postpartum hgb with a mean hgb decrease of 1.35 g/dL (SD 0.91) pre‐ to postdelivery. Among the 11,180 deliveries, 19 (0.2%) received blood and 82 (0.7%) received IV iron (Figure 1). Lower predelivery hgb was associated with blood transfusion (OR: 0.27 [0.19, 0.38]) (Figure 2). Among the 10,954 deliveries with predelivery hgb >9 g/dL, 6 received blood transfusions. Five had symptoms (*n* = 1) or comorbidities (sepsis (*n* = 2), hematoma (*n* = 2)). The interaction between age and predelivery hgb predicted risk for IV iron (OR: 0.96, [0.92, 0.99]). Thirty‐nine patients with a predelivery hgb >9 g/dL received IV iron, of whom 13 had symptoms/comorbidities. Patients with predelivery hgb ≥9 g/dL, normal blood loss, and no symptoms/comorbidities had a 0.23% risk of either intervention (1 blood and 26 IV iron transfusions). At this institution, selective testing would result in an annual cost savings of $109,202.

**Conclusion**: In this large cohort of vaginal deliveries with normal blood loss, blood and IV iron transfusion was rare. Selective PP hgb testing based on predelivery hgb and symptoms/comorbidities appears safe with potential cost savings.

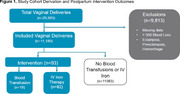

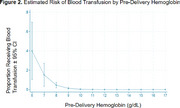

**3:30 PM – 5:00 PM**

## 657 | **Peripartum** Cardiac **Stress Markers Among Pregnancies With and Without Hypertensive Disorder of Pregnancy**

Sarah C. Graves^1^; Uma Perni^1^; Stanley Hazen^2^; Jodi Meridieth^1^; Megan R. Ansbro^3^; Xinmin Li^1^; Deepthi Mallela^4^; Madeline Joquico^1^; Cara D. Dolin^2^

^1^VCU School of Medicine, VCU Health, Richmond, VA; ^2^Cleveland Clinic, Cleveland, OH; ^3^Cleveland Clinic Foundation, Cleveland, OH; ^4^Cleveland Clinic Lerner Research Institute, Cleveland Clinic, OH

**Objective**: To evaluate maternal serum cardiac stress markers, NTproBNP and high‐sensitivity troponin T (hsTropT) at time of delivery in patients with hypertensive disorders of pregnancy (HDP).

**Study Design**: Prospective cohort study of patients ≥18 years with singleton pregnancy. Maternal blood was collected at time of delivery admission. Clinical variables including demographics, medical/obstetric history, pregnancy outcomes, and cardiovascular parameters including blood pressure and baseline labs were collected. NTproBNP and hsTropT were quantified using the Roche analyzer with electrochemiluminescence immunoassay. Levels were compared between those who developed HDP and those who did not. Measures of association with HDP were assessed using multivariable logistic regression and reported as OR and adjusted OR, adjusting for potential confounders.

**Results**: Baseline characteristics were similar between groups. Time of delivery NTproBNP was increased in those with HDP vs no HDP (51.5 [18.0–107.0] vs 38.0 [18.0–60.0], *p* = 0.01). HsTropT was also elevated in the HDP group vs no HDP group (3.0 [3.0–7.11] vs 3.0 [3.0–3.0], *p* < 0.001). Both hsTropT and NTproBNP were significantly associated with risk of HDP (OR [95% CI] 4.62 [2.25–9.88] and 2.42 [1.20–5.01], respectively).

**Conclusion**: Elevated maternal serum NTproBNP and hsTropT at time of delivery were significantly associated with HDP, suggesting peripartum cardiac stress and subclinical myocardial injury may be more pronounced in individuals with HDP, even in the absence of overt cardiovascular disease. Measurement of NTproBNP and hsTropT in the peripartum period may offer insight into maternal cardiovascular strain and could represent potential biomarkers for identifying those at increased risk for HDP. Further research is warranted to assess their utility in risk stratification and their relationship to long‐term cardiovascular outcomes.

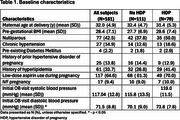

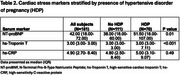

**3:30 PM – 5:00 PM**

## 658 | **Third Trimester** Perceived **Stress is Associated With Adverse Birth Outcomes in Rural Ethiopia**

Sarah KG Jensen, N/A^1^; Firehiwot Workneh^2^; Kalkidan Yibeltal^2^; Theresa Chin^3^; Krysten North^4^; Nebiyou Fasil^2^; Blair J. Wylie^5^; Sara Cherkerzian^4^; Yemane Berhane^2^; Anne CC Lee^3^

^1^Boston Children's Hospital, Boston, MA; ^2^Addis Continental Institute of Public Health, Addis Ababa, Adis Abeba; ^3^Warren Alpert Medical School, Providence, RI; ^4^Brigham and Women's Hospital, Boston, MA; ^5^Beth Israel Deaconess Medical Center, Boston, MA

**Objective**: Low birthweight affects 1 in 7 babies globally, with Ethiopia experiencing 300,000–600,000 annual cases. While maternal psychological stress may represent an added risk factor for adverse birth outcomes in vulnerable populations, most studies of the effects of maternal prenatal stress have been conducted in high‐income countries. We examined the associations of prenatal perceived stress with birth weight‐for‐age, birth length‐for‐age, preterm birth, and stillbirth in the ‘Enhancing Nutrition and Antenatal Infection Treatment for Maternal and Child Health’ (ENAT) cohort in rural Ethiopia.

**Study Design**: The ENAT study is a registered cluster randomized trial (ISRCTN15116516) that enrolled pregnant women (<24 weeks gestation) from August 2020 to December 2021. Socio‐demographic, nutritional, and antenatal history data were collected at enrollment. Perceived stress data were collected at ANC visits using the Cohen's Perceived Stress Scale (PSS), a tool previously validated in pregnant women in Ethiopia. We used regression models with cluster‐robust standard errors and adjusted for potential confounders and women's intervention status.

**Results**: Of *N* = 2,399 women enrolled in ENAT, *n* = 1,103 provided stress data for both second and third trimesters. Second‐trimester stress was not associated with birth outcomes. Third‐trimester stress was not associated with birth weight‐for‐age or preterm birth. However, higher third‐trimester stress predicted lower birth length‐for‐age (coef. = −0.024, 95% CI = −0.038, −0.009, *p* = 0.007) and was associated with a 14% increased stillbirth risk per one‐unit increase in the stress score (OR = 1.14, 95% CI = 1.01, 1.29, *p* < 0.001).

**Conclusion**: Prenatal perceived stress was associated with lower birth length‐for‐age and increased stillbirth risk in our cohort of socioeconomically vulnerable women in rural Ethiopia. These findings suggest that stress screening may help identify women at risk for poor birth outcomes. Future work will examine if these associations differ by nutrition intervention status.

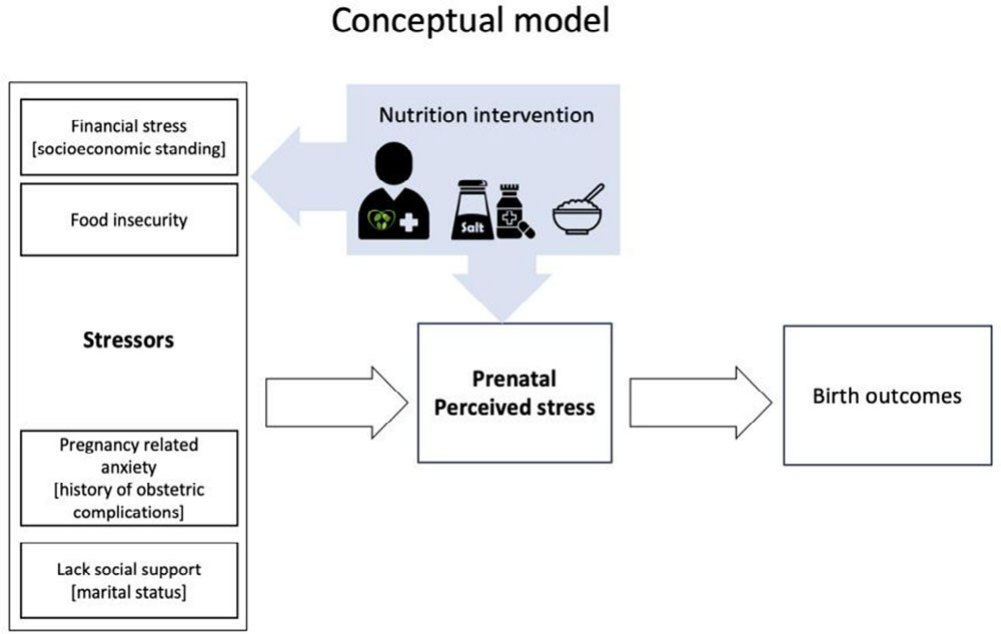

**3:30 PM – 5:00 PM**

## 659 | **Clinicians** Perceptions **of Second Stage Cesarean Delivery Education: A Qualitative Study of Gaps and Opportunities**

Sarah T. McMahon^1^; Erica Marion^1^; Janayah McClellan^1^; Anna Palatnik^2^; Brock E. Polnaszek^1^

^1^Medical College of Wisconsin, Milwaukee, WI; ^2^Medical College of Wisconsin ‐ Milwaukee, WI, Milwaukee, WI

**Objective**: Second stage cesarean delivery is among the most technically challenging and morbid cesarean deliveries, yet educational approaches have remained stagnant. The purpose of this study was to explore clinician perceptions of medical education and training for second stage cesarean delivery management.

**Study Design**: Clinicians were invited to participate in a semi‐structured interview after completing a national survey on second stage cesarean delivery. Semi‐structured interviews were audio recorded, transcribed verbatim, and analyzed using content thematic analysis. Content of the semi‐structured interviews was focused on clinician's knowledge and perceptions of second stage cesarean techniques, educations and training, and real‐world application in the context of perinatal outcomes.

**Results**: A total of (*n* = 41) clinicians participated in semi‐structured interviews, representing all four U.S. geographic regions and a range of training levels (resident, fellow, attending) and specialties (e.g. family medicine, general ob/gyns, maternal fetal medicine). Three major themes emerged: 1) Training variability undermines clinician confidence and competency, particularly among both trainees and attendings; 2) Limited access to simulation and hands‐on training constraints skill development during and post‐residency; 3) Training must be team‐based and institution‐specific, involving interdisciplinary coordination and multidisciplinary care across obstetric teams. (Table 1).

**Conclusion**: Clinicians identified a critical need for structured, standardized education on second stage cesarean delivery techniques, regardless of training level or specialty. Given its recognition as a high‐risk procedure associated with avoidable harm and rising litigation, consistent, multidisciplinary training may better equip clinicians with a reliable surgical toolbox to manage complex second stage scenarios and improve maternal and neonatal outcomes.

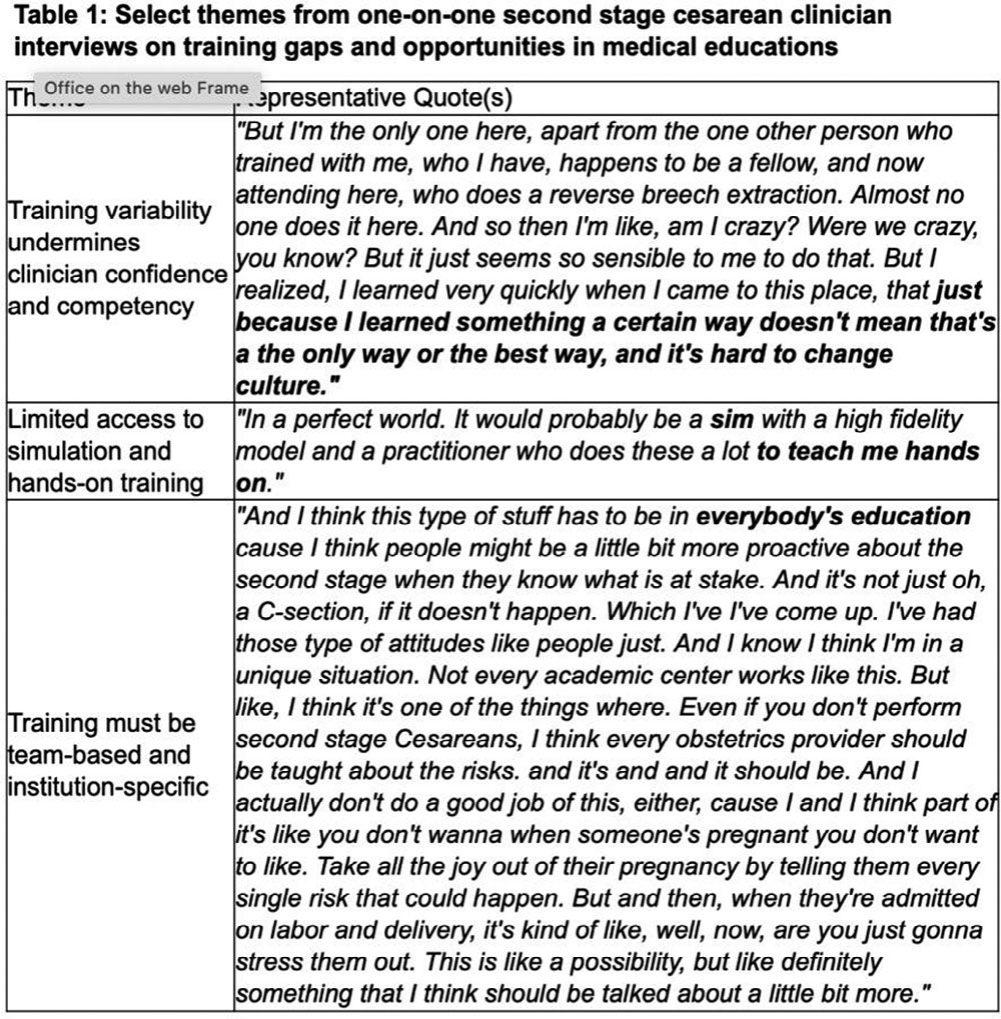

**3:30 PM – 5:00 PM**

## 660 | **Postpartum** Blood **Pressure Trends and Time in Target Range During Remote Blood Pressure Monitoring**

Sarah Shim^1^; Connor Demorest^2^; Katelyn M. Tessier^3^; Martina S. Burn^2^; Tiffany Corlin^2^; Sarah A. Wernimont^2^; Bethany A. Sabol^2^

^1^University of Minnesota Medical Center, Minneapolis, MN; ^2^University of Minnesota, Minneapolis, MN; ^3^University of Minnesota Masonic Cancer Center, University of Minnesota, Minneapolis MN, MN

**Objective**: Remote blood pressure monitoring (RBPM) is a key part of postpartum hypertensive disorders of pregnancy (HDP) care, but data comparing blood pressure (BP) control across antihypertensive regimens during participation remain limited. Here, we evaluate how standardized BP management in our 6‐week RBPM program impacted postpartum BP trends and time in target range (TTR), stratified by discharge medication.

**Study Design**: This is a retrospective cohort study of all individuals with HDP who enrolled in a six‐week postpartum RBPM from November 2023‐December 2024 at a single institution. This program uses a nurse‐driven standardized protocol for antihypertensive medication initiation and titration to achieve a goal BP of ≤140/≤90 mmHg. Based on the standardized protocol utilized, we defined target range as a SBP 100–139 mmHg and DBP 60–89 mmHg. The primary outcome was BP trend and TTR throughout program participation stratified by antihypertensive medication at hospital discharge.

**Results**: Of the 465 individuals in the cohort, 146 (31.4%) were discharged without antihypertensive medications, 40 (8.6%) on Labetalol only, 176 (37.8%) on Nifedipine only, and 93 (20%) on two or more medications. Ten used other antihypertensive medications. Mean BPs were <140/90 mmHg for all groups for each program week. The steepest BP decline was observed within the first week followed by a plateau in both systolic and diastolic BP by week 4 of the program (Figure 1). The majority of participants were within target range throughout (TTR by group varied between 80.3–90.4%). Episodes of severe hypertension and hypotension were rare and not significantly different between groups (Figure 2).

**Conclusion**: Postpartum RBPM programs have emerged as an important tool for postpartum care. Using an RN‐driven standardized protocol for antihypertensive medication initiation and titration, our program achieves optimal BP control irrespective of medication regimen at discharge.

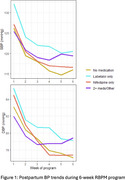

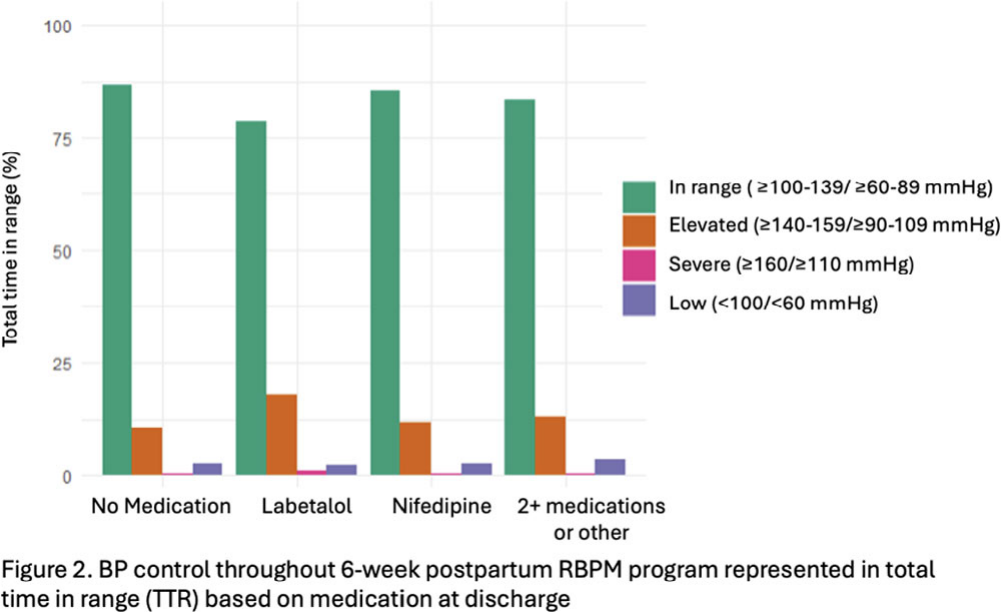

**3:30 PM – 5:00 PM**

## 661 | **Maternal‐**Newborn **Dyadic Outcomes by Severity of Weight Gain in Obese Pregnancies**

Sarah Z. Wu; Frank I. Jackson; Sarah H. Abelman; Oladunni Ogundipe; Matthew J. Blitz

Northwell, New Hyde Park, NY

**Objective**: To evaluate the association between excess gestational weight gain (EGWG) and maternal‐newborn dyadic outcomes using the desirability of outcome ranking (DOOR) methodology among patients with pre‐pregnancy obesity.

**Study Design**: This retrospective cross‐sectional study included all deliveries eligible for the Joint Commission's PC‐06 quality metric (term newborns without preexisting conditions) at seven hospitals within a large New York health system (2019–2024). Only each patient's first delivery was analyzed. Among patients with pre‐pregnancy obesity, gestational weight gain was categorized as appropriate (≤20 lb per 2009 Institute of Medicine guidelines) or excess (>20 lb), and further classified as mild (21–29 lb), moderate (30–39 lb), or severe (≥40 lb). DOOR methodology grouped outcomes by delivery mode and presence of maternal or neonatal complications: (1) uncomplicated vaginal delivery, (2) uncomplicated cesarean, (3) vaginal delivery with complications, and (4) cesarean with complications. Multinomial logistic regression assessed the association between EGWG and DOOR category, adjusting for race and ethnicity, insurance, language, multiparity, and the obstetric comorbidity index (OB‐CMI).

**Results**: Among 18,907 patients, DOOR outcomes by EGWG group are shown in the Figure; regression results are in the Table. Compared to appropriate GWG, mild, moderate, and severe EGWG were each associated with increased odds of uncomplicated cesarean delivery (aORs 1.16, 1.17, and 1.18, respectively). No EGWG group showed a significant increase in the risk of cesarean with complications or vaginal delivery with complications. Risk of adverse outcomes was more strongly associated with OB‐CMI and certain racial and ethnic groups.

**Conclusion**: In patients with obesity, EGWG was linked to higher cesarean delivery rates but not to increased complications. These findings suggest that while EGWG may influence delivery mode, comorbidities and social determinants are more predictive of adverse dyadic outcomes.

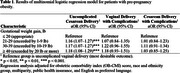

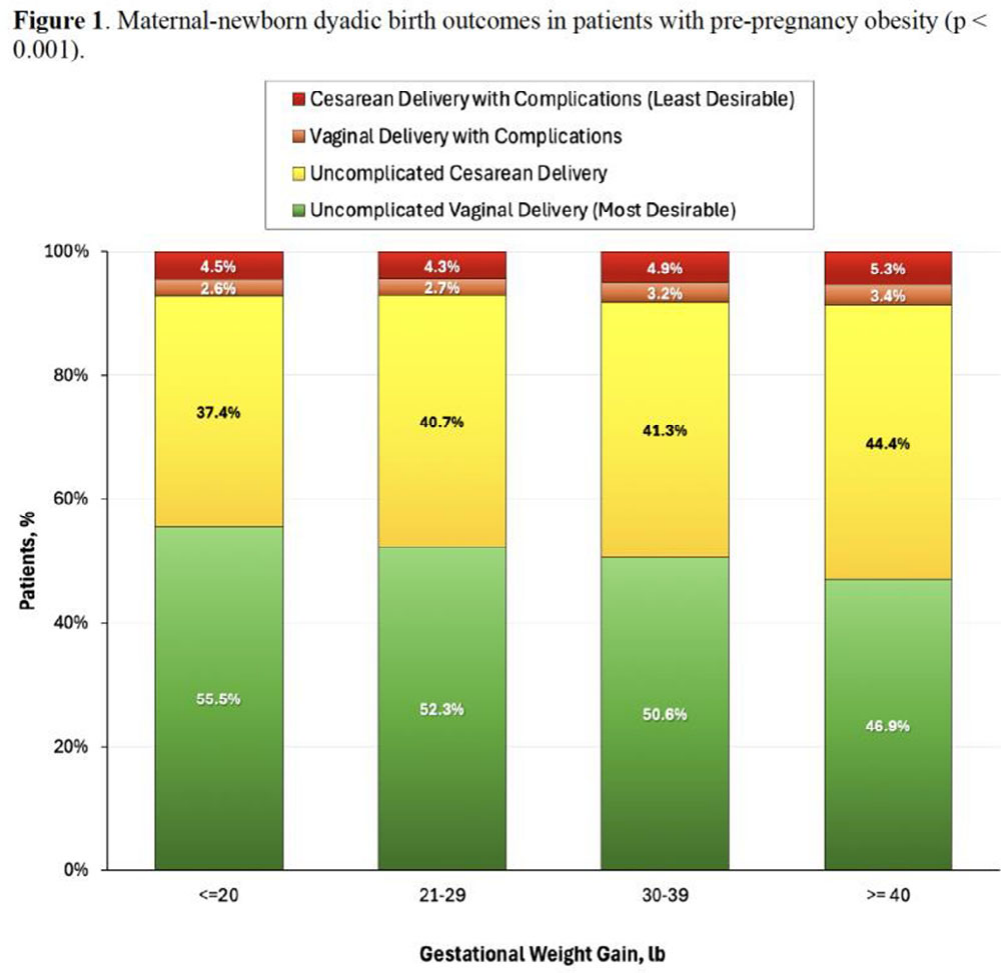

**3:30 PM – 5:00 PM**

## 662 | **Transvaginal** Cervical **Length Assessment in Comparing Single Balloon Versus Double Balloon Cervical Ripening**

Satjeet K. Deol Chauhan^1^; Faro Revital^1^; Rodney A. McLaren, Jr^2^; Fouad Atallah^3^

^1^Bronxcare Health System/Icahn School of Medicine at Mount Sinai, Bronx, NY; ^2^Maimonides Medical Center, Brooklyn, NY; ^3^NYP Brooklyn Methodist Hospital, New York, NY

**Objective**: The aim of our study was to evaluate the feasibility of performing a transvaginal cervical length (TVCL) assessment as a more objective measure of cervical effacement in comparing single balloon versus double balloon cervical ripening during induction of labor.

**Study Design**: This was a pilot prospective study involving patients undergoing labor induction. After informed consent, the patients underwent either a single balloon foley catheter or double balloon Cook catheter placement. TVCL measurement was performed before the placement of cervical ripening balloon and after balloon expulsion. The primary outcomes were number of patients recruited, change in digital Bishops score, difference in cervical length pre and post cervical balloon ripening, and total cervical ripening balloon time. Secondary outcomes were induction to active labor interval, induction to delivery interval, intrapartum complications, and cesarean delivery. Univariable analyses were performed, and data was presented as median (interquartile range).

**Results**: Of the 105 patients approached, 30 (28.6%) were consented. Baseline characteristics did not differ between both the groups. Patients in the double balloon cook catheter group had a higher Bishop score after ripening (Foley: 7 IQR(7,7) vs. Cook: 8 IQR(8, 9); *p* < 0.001) and TVCL difference (Foley: 1.12 IQR (0.91, 1.3) cm vs. Cook: 1.7 IQR(1.38, 2.19) cm; *p* < 0.001) than those in the Foley group. However, time to active labor (Foley: 8 IQR(6, 14) hr vs. Cook: 9 IQR (6,12) hr; *p* = 0.945), time to delivery (Foley: 11 IQR(7.5, 21) hr vs. Cook: 15 IQR (10, 20) hr; *p* = 0.575), and cesarean delivery (Foley: 20% vs Cook: 13.3%; *p* > 0.999) did not differ (Table 1).

**Conclusion**: TVCL measurement was feasible and may be used as an objective measure of effacement during intrapartum ultrasound assessment. Cervical ripening with double balloon cook catheter resulted in greater effacement, more cervical shortening compared to single balloon foley catheter without significant difference in induction to delivery interval.

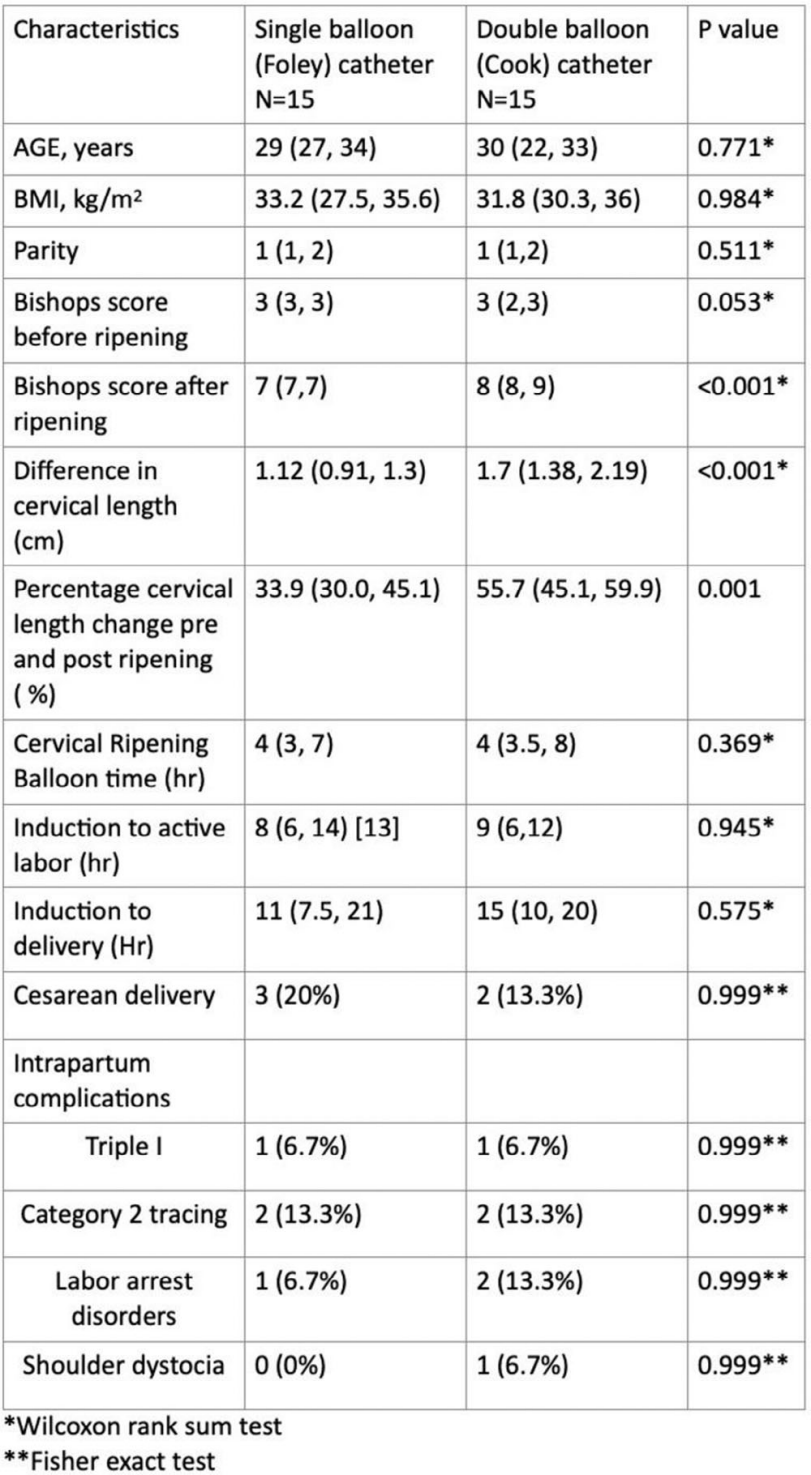

**3:30 PM – 5:00 PM**

## 663 | **County‐Level** Associations **Between Maternal Care Access and Nulliparous, Term, Singleton, Vertex Cesarean Delivery Rates**

Sebastian Z. Ramos^1^; Zeinab Kassem^1^; Phinnara Has^2^; Stephen M. Wagner^3^

^1^Tufts University School of Medicine, Boston, MA; ^2^Brown University Health BERD Core, Providence, RI; ^3^Beth Israel Deaconess Medical Center, Boston, MA

**Objective**: The nulliparous, term, singleton, vertex (NTSV) cesarean birth rate is a widely accepted obstetric quality metric used to assess care patterns and compare performance across hospitals and regions. Safe reduction of primary cesarean births is critical to minimizing long‐term maternal morbidity. It remains unclear how regional access to obstetric care affects NTSV cesarean delivery and related outcomes. This study examines the relationship between county‐level maternal care access and NTSV cesarean delivery rates.

**Study Design**: This is a cross‐sectional study using restricted‐access CDC birth certificate data from 2019–2021. Nulliparous, vertex, singleton, non‐anomalous pregnancies between 37–42 weeks were included. Counties were categorized into four levels of maternal care access: desert, low, moderate, and full access based on March of Dimes data. The primary outcome was rate of NTSV cesarean delivery. Mixed‐effects logistic regression models estimated associations between maternal care access and NTSV cesarean delivery, adjusting for individual‐level clinical factors and county‐level social vulnerability using the CDC Social Vulnerability Index.

**Results**: There were 3,441,704 births in 3137 counties, with 3.6% occurring in maternal care deserts. Patients in maternal care deserts were younger, more likely to be Non‐Hispanic White, and more likely to undergo labor induction. Operative vaginal delivery rates were 19% higher in maternal care deserts compared to full access counties (6.35% vs. 5.64%, *p* < 0.001). The overall NTSV cesarean rate was 25.9%. After adjusting for confounders, residing in a maternal care desert was associated with increased odds of NTSV cesarean delivery compared to full access areas (aOR 1.08, 95% CI: 1.04–1.11, *p* < 0.001).

**Conclusion**: Geographic disparities in access to maternity care are associated with variation in NTSV cesarean delivery. These findings suggest that structural access to obstetric services may influence clinical decision‐making. Further research should explore drivers of this variation and evaluate interventions to support equitable, patient‐centered care.

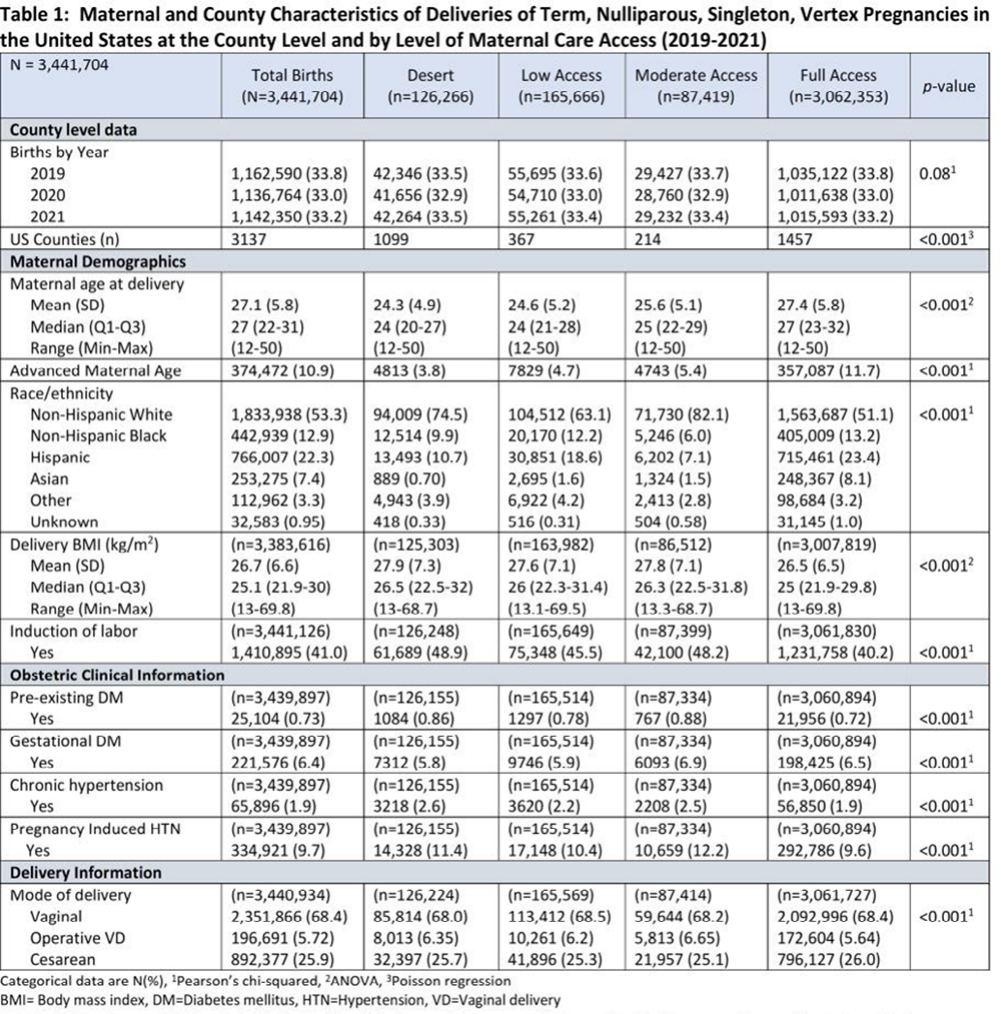

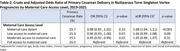

**3:30 PM – 5:00 PM**

## 664 | **Empty Plates,** Smaller **Weights: Exploring the Association Between Food Insecurity and Fetal Growth Restriction**

Shahd Karrar^1^; Hwsoo Shin^2^; Qiong Zhang^2^; Muniza Hossain^2^; Leah Hong^2^; D'Angela S. Pitts^2^; Raminder Khangura^2^; Gregory Goyert^2^

^1^Henry Ford Health ‐ Maternal Fetal Medicine, Royal Oak, MI; ^2^Henry Ford Health, Henry Ford Health ‐ Detroit, MI

**Objective**: Fetal growth restriction (FGR) is a marker of placental insufficiency and adverse perinatal outcomes. The relationship between neighborhood‐level food insecurity and FGR remains underexplored. We examined whether residence in USDA‐defined areas of food insecurity is associated with FGR to understand the impact of unmet nutritional needs on fetal growth.

**Study Design**: We conducted a retrospective cohort study of singleton pregnancies receiving prenatal care and delivering ≥24 weeks at Henry Ford Health System (2015–2023). Residential addresses were geocoded and linked to USDA Food Access Research Atlas data of food insecurity, defined by low income (LI), low access (LA), and low vehicle access. FGR was defined as estimated fetal weight (EFW) or abdominal circumference <10th percentile by ultrasound. Multivariable logistic regression assessed associations between residence in areas of food insecurity and FGR, adjusting for maternal age, and urban status. Analyses were performed using R 4.4.0 with α = 0.05.

**Results**: Among 38,597 pregnancies, 3074 (8.0%) were diagnosed with FGR. Univariate analysis showed those with FGR were younger (27.9 vs 29.1 years, *p* < 0.001) and resided in LI tracts (63.8% vs 56.1%, *p* < 0.001) and areas with low vehicle access (27.6% vs 24.6%, *p* < 0.001). In multivariable analysis, FGR was associated with urban residence (aOR 1.48, 95% CI: 1.13–1.94), LI tract residence (aOR 1.20, 95% CI: 1.10–1.32), and younger age (aOR 0.97, 95% CI: 0.965–0.976). LA alone was no longer significant (*p* = 0.051).

**Conclusion**: Residence in areas of food insecurity, particularly those with low income or urban designation, is significantly associated with FGR. Notably, low access alone was not a significant predictor. These findings suggest that financial hardship and urban structural inequities may be more impactful than proximity to food alone. Targeted interventions must address the economic and systemic barriers contributing to fetal growth disparities.

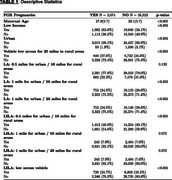

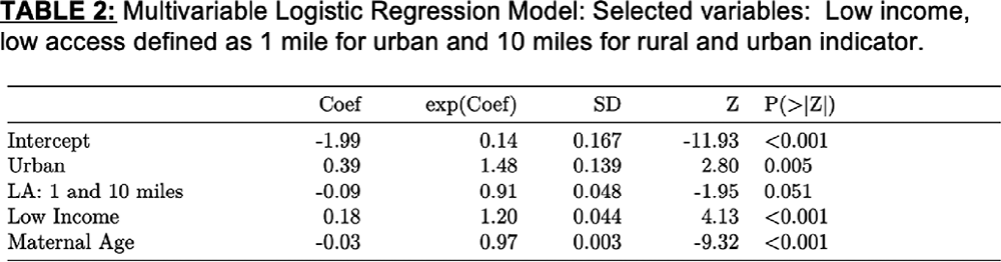

**3:30 PM – 5:00 PM**

## 665 | **Prolonged Second Stage in the Presence of Meconium‐Stained Amniotic Fluid: Is it Worth the Wait?**

Shani Abramov^1^; Omri Dominsky^2^; Roza Shperling‐Berkovitz^3^; Anat Lavie^2^; Yariv Yogev^4^; Liran Hiersch^5^; Uri Amikam^6^

^1^Lis Hospital for Women's Health, Tel Aviv Sourasky Medical Center, Tel‐Aviv, Tel Aviv; ^2^Lis Hospital for Women's Health, Tel Aviv Sourasky Medical Center, ISRAEL, Tel Aviv; ^3^Lis Hospital for Women's Health, Tel Aviv Sourasky Medical Center, ichilov, HaMerkaz; ^4^Lis Hospital for Women's Health, Tel Aviv Sourasky Medical Center Gray Faculty of Medicine, Tel Aviv University, Israel, Lis Hospital for Women's Health, Tel Aviv Sourasky Medical Center, Tel Aviv; ^5^Lis Hospital for Women's Health, Tel Aviv Sourasky Medical Center Gray Faculty of Medicine, Tel Aviv University, Israel, ISRAEL, Tel Aviv; ^6^Lis Hospital for Women's Health, Tel Aviv Sourasky Medical Center, Tel Aviv, Tel Aviv

**Objective**: To determine whether meconium‐stained amniotic fluid (MSAF) during prolonged second stage (PSS) of labor is associated with increased perinatal morbidity.

**Study Design**: A retrospective cohort study including term singleton vertex live births with PSS at a single center (2020–2025). PSS was defined as >2 or >3 hours in nulliparas and >1 or >2 hours in multiparas, with or without epidural analgesia, respectively. Deliveries with MSAF were compared to those with clear fluid. Multivariable logistic regression adjusted for confounders. A sub‐analysis was conducted for nulliparous women. Two composite outcomes were evaluated: a maternal composite including intrapartum fever, postpartum hemorrhage (PPH), obstetric anal sphincter injury (OASI), blood transfusion, or shoulder dystocia; and a neonatal composite including 5‐minute Apgar <7, umbilical cord pH <7.1, or NICU admission.

**Results**: Overall, out of 54,525 deliveries, 3665 (6.7%) met PSS criteria; among these, 660 (18%) had MSAF and 3005 (82%) had clear amniotic fluid.

The MSAF group had higher rates of vacuum extraction (55.6% vs. 48.5%, *p* = 0.001), OASI (3.0% vs. 0.9%, *p* = 0.001), PPH (11.2% vs. 8.1%, *p* = 0.011), and blood transfusion (5.3% vs. 3.4%, *p* = 0.022).

Utilizing multivariable logistic regression adjusting for maternal age >35 years, pre‐gestational BMI, induction of labor, prelabor rupture of membranes, epidural analgesia, gestational age, and birthweight, composite maternal morbidity was significantly higher in the MSAF group (20.0% vs. 15.6%, aOR 1.3, CI(1.036–1.627), *p* = 0.006), with comparable composite neonatal morbidity (5.8% vs. 4.7%, aOR 1.14, CI(0.78–1.69), *p* = 0.231).

The sub‐analysis including only nulliparas (*n* = 2,749) demonstrated similar results (Figure).

**Conclusion**: In the setting of a prolonged second stage, the presence of meconium‐stained amniotic fluid is associated with increased maternal morbidity, with comparable neonatal morbidity.

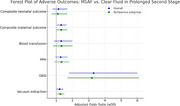

**3:30 PM – 5:00 PM**

## 666 | **Parity and Prior** VBAC **in Relation to Uterine Rupture Risk During TOLAC**

Shanny Kolp‐Asis^1^; keren Nicole Hamisha^2^; limor Vaknin Geron^3^; Ariel Many^4^

^1^Ma'ayanei Ha'yeshua Medical Center, bnei brak, HaMerkaz; ^2^Department of Obstetrics and Gynecology, Mayaney Hayeshua Medical Center, Bnei Barak; Israel, bnei brak, HaMerkaz; ^3^Department of OB&GYN, Mayaney Hayeshua Medical Center., bnei brak, HaMerkaz; ^4^Department of OB&GYN, Mayaney Hayeshua Medical Center, bney brak, HaMerkaz

**Objective**: To evaluate the independent effects of parity and prior vaginal birth after cesarean (VBAC) on the risk of uterine rupture among women undergoing trial of labor after cesarean (TOLAC).

**Study Design**: We conducted a retrospective cohort study of 9597 women with a single prior cesarean delivery who attempted TOLAC at a tertiary medical center between 2011–2023. Multivariable logistic regression models were used to assess the association between increasing parity, prior VBAC, and uterine rupture risk, adjusting for induction of labor, birthweight, maternal age, and other potential confounders.

**Results**: Overall, 38 uterine ruptures occurred (0.4%). Increasing parity was significantly associated with lower odds of uterine rupture (adjusted OR 0.78, 95% CI 0.62–0.97, *p* = 0.026). Similarly, a history of prior VBAC was protective when assessed individually (unadjusted OR 0.67, 95% CI 0.52–0.86, *p* < 0.001). However, when parity and prior VBAC were included in the same multivariable model, only parity remained statistically significant (adjusted OR 0.78, 95% CI 0.62–0.97), while prior VBAC was no longer independently associated with reduced rupture risk (adjusted OR 0.76, 95% CI 0.55–1.05, *p* = 0.093). Induction of labor was independently associated with a higher rupture risk (adjusted OR 3.12, 95% CI 1.60–6.08). Neither birthweight nor maternal age were significantly associated with uterine rupture.

**Conclusion**: Increasing parity is independently associated with a reduced risk of uterine rupture during TOLAC, whereas prior VBAC, although protective in unadjusted analyses, does not retain independent significance after adjustment. These findings suggest that the protective effect of prior vaginal delivery is largely explained by higher parity rather than VBAC status alone, which may improve counseling and risk stratification for women planning TOLAC.

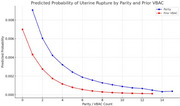

**3:30 PM – 5:00 PM**

## 667 | **Infectious** Morbidity **in Term‐Born Infants Following Antenatal Corticosteroid Exposure Before 34 Weeks: an Observational Study**

Shayna Miodownik^1^; Tamar Wainstock^2^; Eyal Sheiner^3^

^1^Soroka University Medical Center, Soroka University Medical Center, HaDarom; ^2^Department of Epidemiology, Biostatistics and Community Health Sciences, Faculty of Health Sciences, Ben‐Gurion University of the Negev, Beer Sheva, HaDarom; ^3^Soroka University Medical Center, Faculty of Health Sciences, Ben‐Gurion University of the Negev, beer sheva, HaDarom

**Objective**: Antenatal corticosteroid (ACS) administration in anticipation of preterm birth is a well‐established intervention, shown to significantly improve neonatal outcomes. However, many exposed fetuses are delivered at term, raising concerns about potential long‐term effects. This study assessed long‐term infectious morbidity in term‐born offspring exposed to ACS before 34 weeks’ gestation, compared to unexposed controls.

**Study Design**: A population‐based cohort study was conducted. Offspring born at term were categorized based on ACS exposure prior to 34 weeks gestation. Data were collected from ambulatory and hospitalization records and the incidence of infectious morbidity in offspring up to age 18 years was compared. A Kaplan–Meier survival analysis assessed cumulative infectious incidence, and a Cox proportional hazards model adjusted for potential confounders.

**Results**: Among 182,626 neonates born at term, 2093 were exposed to ACS prior to 34 weeks gestation. Term‐born offspring exposed to ACS demonstrated a higher rate of long‐term infectious morbidity (324 vs. 187 cases per 1000 follow‐up years; *p* < 0.001), particularly neonatal infections, viral infections, bronchiolitis, and ear, nose and throat (ENT) infections (Table). Kaplan–Meier analysis demonstrated a significantly increased cumulative incidence in the ACS‐exposed group (*p*‐value <0.001, Figure). In a Cox regression analysis, which controlled for maternal and gestational age, diabetes mellitus, hypertensive disorders and cesarean delivery, ACS exposure was independently associated with infectious morbidity (aHR = 1.116, 95% CI 1.08–1.14, *p*‐value <0.001). In a subgroup analysis for early term, the results remained significant.

**Conclusion**: ACS exposure is associated with a significantly increased risk of long‐term infectious morbidity in term‐born infants. These findings underscore the importance of ongoing evaluation of ACS use and highlight the need for careful patient selection and adherence to clear indications when considering ACS, particularly in pregnancies that may reach term.

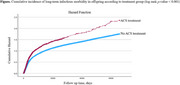

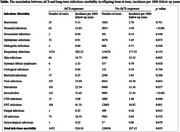

**3:30 PM – 5:00 PM**

## 668 | **Nulliparity at** Advanced **Maternal age and Long‐Term Morbidity in Offspring: A Population‐Based Cohort Study**

Shira Gat^1^; Tamar Wainstock^2^; Gali Pariente^3^; Eyal Sheiner^4^; Gil Gutvirtz^5^

^1^Department of Obstetrics and Gynecology, Soroka University Medical Center, Ben‐Gurion University, Beer‐Sheva, HaDarom; ^2^Department of Epidemiology, Biostatistics and Community Health Sciences, Faculty of Health Sciences, Ben‐Gurion University of the Negev, Beer Sheva, HaDarom; ^3^Department of Obstetrics and Gynecology, Soroka University Medical Center, Ben‐Gurion University, beer sheva, HaDarom; ^4^Soroka University Medical Center, Faculty of Health Sciences, Ben‐Gurion University of the Negev, beer sheva, HaDarom; ^5^Department of Obstetrics and Gynecology, Soroka University Medical Center, Ben‐Gurion University, Metar, HaDarom

**Objective**: Birth rates among women aged 30 and above have climbed to record highs, influenced by evolving social and economic dynamics, including greater focus on professional advancement, prolonged educational pursuits, and increased access to fertility treatments. The aim of the study is to assess whether nulliparity at advanced maternal age (AMA) is associated with increased long‐term morbidity in offspring.

**Study Design**: A population‐based retrospective cohort study was conducted at a single tertiary medical center between 1991 and 2021. The cohort included all nulliparous women aged ≥30 years who delivered a live singleton. Participants were stratified into three age groups: 30–35, 36–40, and >40. Offspring were followed until 18 years of age for the occurrence of long‐term cardiovascular, respiratory, endocrine, neurologic, gastrointestinal, or infectious morbidities, based on community‐based or hospitalizations. Kaplan–Meier survival analyses and Cox proportional hazards models were used to evaluate cumulative incidence and adjusted hazard ratios for each morbidity category.

**Results**: The study included 4951 women: 3729 (75.3%) aged 30–35, 946 (19.1%) aged 36–40, and 276 (5.6%) aged >40. Increasing maternal age was associated with higher rates of hypertensive disorders, gestational diabetes, preterm birth, and cesarean delivery (*p* < 0.001). However, no significant differences were observed in cumulative incidence of long‐term morbidity among offspring across the maternal age groups (Figure). In multivariable analysis adjusted for fertility treatment, ethnicity, cesarean delivery, gestational age, gestational diabetes, and hypertensive disorders, AMA was not independently associated with increased long‐term morbidity in any diagnostic category (Table).

**Conclusion**: Although AMA is associated with adverse pregnancy outcomes, nulliparity at AMA is not associated with long‐term morbidity in offspring.

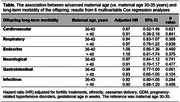

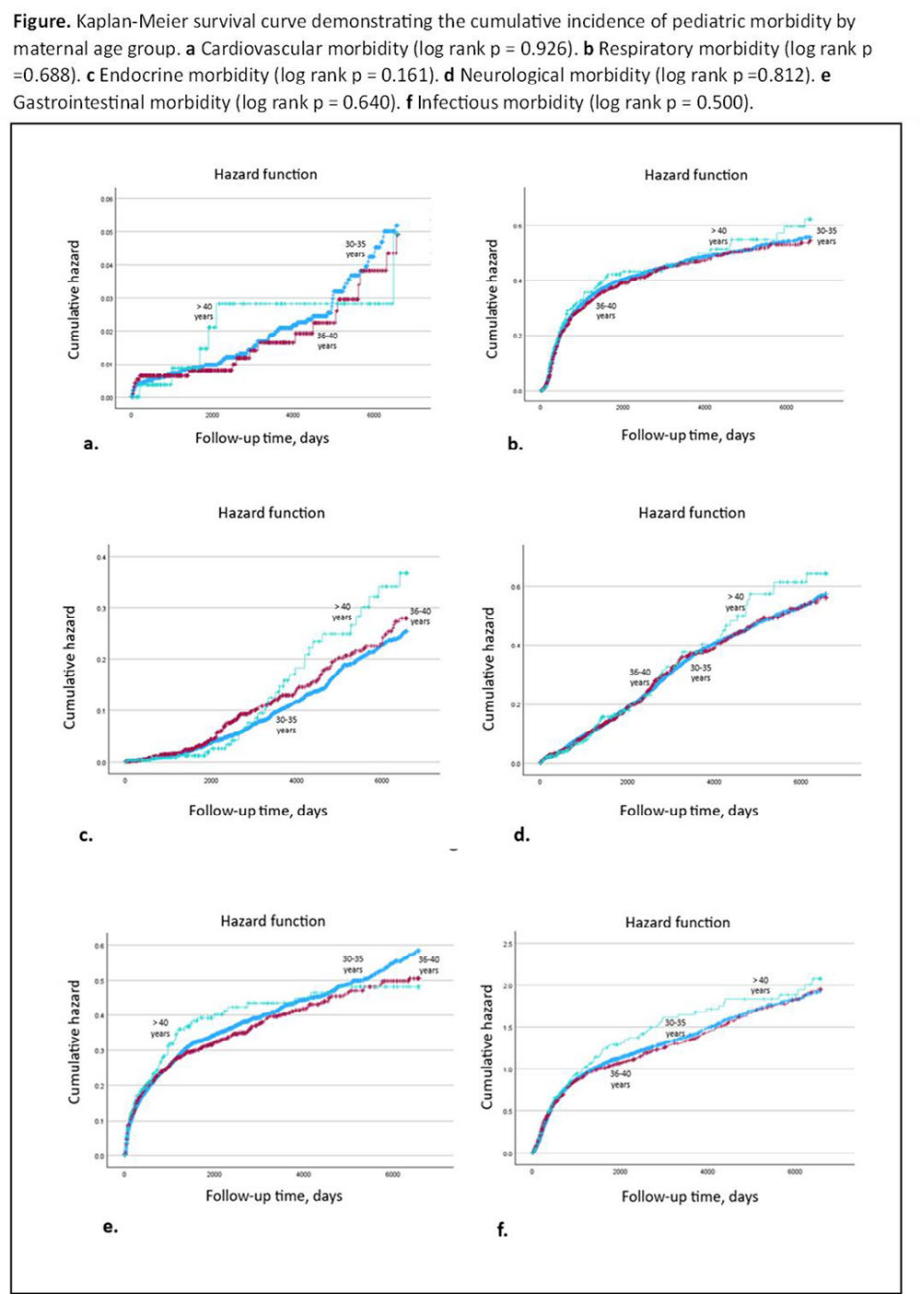

**3:30 PM – 5:00 PM**

## 669 | **Rh Testing And** Immunoglobulin **Administration in Early Pregnancy Among United States Family Planning Providers**

Shirley Qian^1^; Ann Frisse^2^; Jennifer Salcedo^2^; Timothy Ryntz^2^; Bianca Stifani^2^

^1^Westchester Medical Center, White Plains, NY; ^2^Westchester Medical Center, Valhalla, NY

**Objective**: New guidelines from some professional societies recommend foregoing routine Rh testing and Rh immunoglobulin (RhIg) administration for abortion or miscarriage before 12 weeks. We sought whether US Complex Family Planning (CFP) providers practice accordingly, and to describe the de‐implementation process for routine Rh testing and RhIg administration.

**Study Design**: Using the CFP e‐mail listserv, we invited US CFP providers to complete an anonymous online survey hosted on Qualtrics. We offered a raffle for five 100 USD gift cards as a participation incentive. To examine predictors of following new guidelines, we conducted a relative risk regression using a modified Poisson approach with robust error variances, including covariates with *p* < 0.1 on bivariate analysis. We used Stata SE 16.1 for analyses, setting the level of significance at 5%.

**Results**: Of 575 listserv members, 241 (41.9%) responded to the survey. Of these, 152 (63.1%) initiated Rh testing and 179 (74.3%) administered RhIg only after 12 weeks. Providers in the Midwest and West were more likely than those in the Northeast to follow new guidelines (RR 1.81 [1.23–2.66], and RR 2.25 [1.62–3.13], respectively). Those practicing at Planned Parenthood or independent clinics were more likely to follow new guidelines than those at academic medical centers (RR 1.39 [1.11–1.74]). Most respondents reported a deliberate effort to change practice at their facility (161/203, 79.3%). Some (78/203, 38.4%) reported disagreements among clinicians, but most said patients rarely (134/203, 66.0%) or never (35/203, 17.2%) disagreed.

**Conclusion**: A large majority of participants de‐implemented Rh testing or RhIg administration prior to 12 weeks gestation, aligning with the 2022 SFP guidelines. The two most influential factors were the practice region and the practice setting, perhaps most likely driven by state policies as described in the qualitative analysis of the study, in addition to expertise driven by maternal‐fetal medicine specialists at those respective sites.

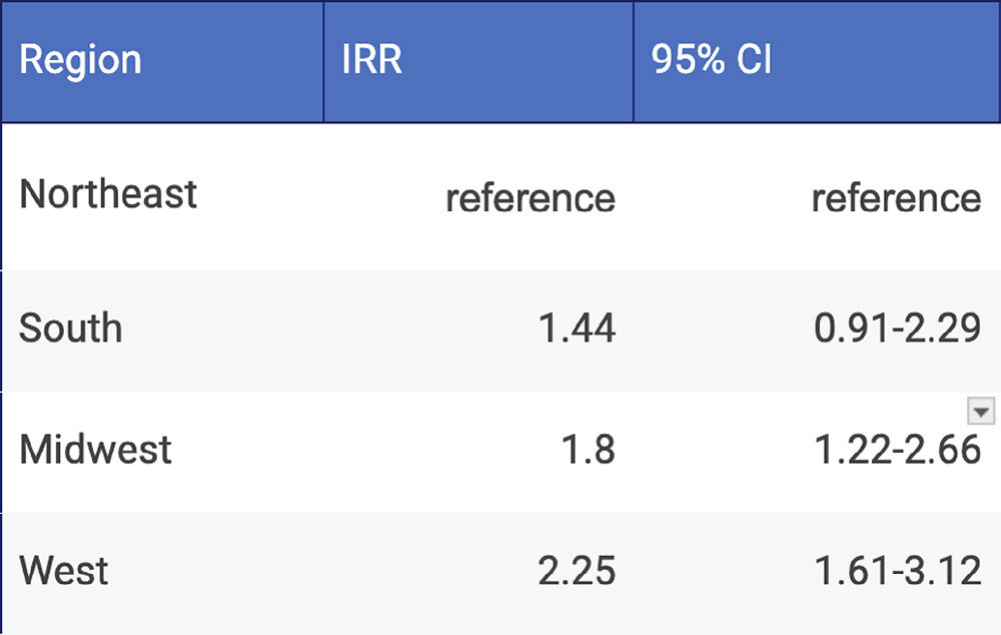

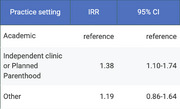

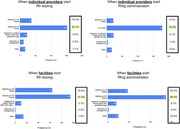

**3:30 PM – 5:00 PM**

## 670 | **Feasibility Trial** of **Viral Mitigation Measures to Reduce Preterm Birth in High‐risk Pregnant Women**

Shivadharshini Sridhar^1^; Ben W. Mol^2^; Ryan Hodges^3^; Kirsten R. Palmer^3^; Suresh Sundram^3^; Rodolfo C. de Carvalho Pacagnella^4^; Renato Souza^4^; Francisco Barbosa‐Junior, Jr.^5^; Daniel L. Rolnik^3^; Atul Malhotra^3^

^1^Monash Health, BURWOOD EAST, Victoria; ^2^Monash Health, Monash University, Victoria; ^3^Monash Health, Melbourne, Victoria; ^4^University of Campinas, Campinas, Sao Paulo; ^5^University of São Paulo, Ribeirão Preto, Sao Paulo

**Objective**: During the COVID‐19 pandemic, reduction in preterm birth (PTB) rates in women exposed to viral mitigation measures was reported, specifically, in women with prior PTB. This pilot study aimed to establish the feasibility of a lifestyle intervention mimicking viral mitigation measures in high‐risk pregnant women.

**Study Design**: We conducted a two‐arm open‐label feasibility randomized controlled clinical trial in a maternity center in Melbourne, Australia. Pregnant women with previous PTB <34 weeks were eligible. The intervention group was asked to comply with lifestyle measures including changes to hygiene measures, social contacts and daily activities. The control group underwent standard pregnancy care. The primary outcome was feasibility, measured as participant recruitment rate, compliance rate (to lifestyle interventions) and data completion rate. Secondary outcomes included incidence of preterm birth <34 weeks, pregnancy and neonatal outcomes. Analysis was by intention to treat. Trial registration: ACTRN12622000753752.

**Results**: From June 2022 to April 2025, 201 eligible women were screened, of which 50 (24.8%) consented and 47 were randomized. Median maternal age and gestation on the first day of the trial were comparable (Table 1). 19/23 (83%) participants randomized to intervention group completed the lifestyle intervention, with 18/23 (78%) in the intervention group and 19/24 (79%) in the control group completing the final survey. The intervention group demonstrated good compliance with lifestyle measures (Table 2), meeting the target of 75%. Incidence of PTB <34 weeks was comparable (Table 1).

**Conclusion**: While a pregnancy lifestyle intervention mimicking viral mitigation measures aimed at reducing PTB in high‐risk women was difficult from a recruitment standpoint, participants who were motivated to enroll, demonstrated low dropout rates, good compliance, and high data completion.

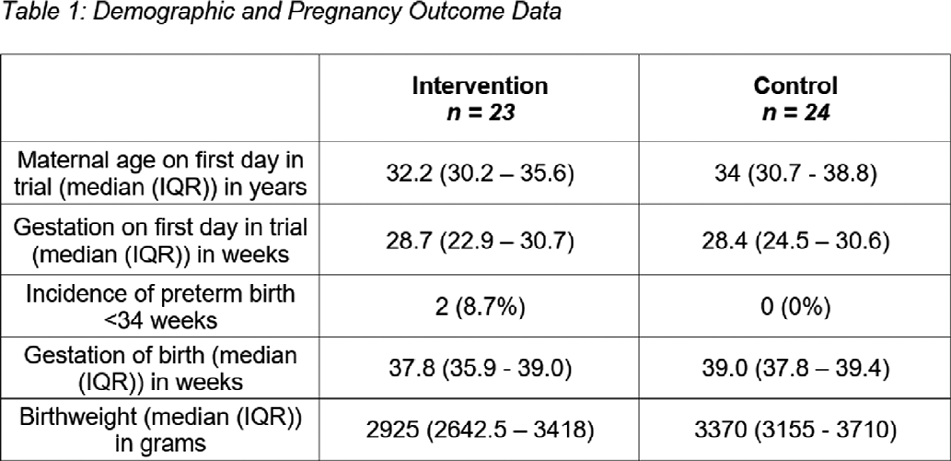

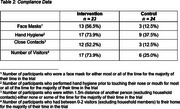

**3:30 PM – 5:00 PM**

## 671 | **Patient** Perspectives **About Universal Inpatient Postpartum Depression Screening**

Shobha Jagannatham^1^; Anna Cavallino^2^; Marlee Madora^1^; Rubiahna L. Vaughn^1^; Kavita Vani^1^

^1^Albert Einstein College of Medicine/Montefiore Medical Center, Bronx, NY; ^2^Albert Einstein College of Medicine/Montefiore Medical Center, Brooklyn, NY

**Objective**: To explore patient perspectives on postpartum depression (PPD) screening during the delivery admission.

**Study Design**: In April 2024, we implemented universal PPD screening using the Edinburgh Postnatal Depression Scale (EPDS) during delivery hospitalization. Scores ≥10 prompted referral to social work and reproductive psychiatry. We conducted semi‐structured interviews with postpartum patients to assess screening experiences. The interview guide was informed by Consolidated Framework for Implementation Research (CFIR) domains: intervention characteristics, inner and outer settings, characteristics of individuals, and process. A purposeful sample was selected based on psychiatric history, parity, and delivery course until thematic saturation was reached. After informed consent, interviews were audio‐recorded, transcribed verbatim, and analyzed using thematic analysis. Two coders iteratively developed a codebook using inductive and deductive approaches.

**Results**: We interviewed 20 patients with varied psychiatric and obstetric histories. Most were familiar with PPD (95%, *n* = 19); 75% (*n* = 15) experienced a pregnancy complication. Median EPDS score was 4 [IQR 1–6]. Themes aligned with CFIR domains and constructs:

- **Intervention Characteristics–Design Quality & Complexity**: *Screening Reactions* ranged from validating to intrusive. *Administration Preferences* (paper vs. verbal screening) were shaped by literacy, privacy concerns, and engagement with staff.
- **Inner Setting–Compatibility & Culture**: *Preferred Timing* of screening varied; while many found screening during hospitalization acceptable, others preferred later screening after recovery and adjustment.
- **Outer Setting–Patient Needs**: *Birth Experience Emotions* like trauma and exhaustion shaped receptivity to screening.
- **Characteristics of Individuals–Knowledge and Beliefs**: *Understanding of PPD* varied; some recognized symptoms in hindsight, while others normalized them.

**Conclusion**: Universal PPD screening was generally acceptable, but varied receptivity underscores the need for flexible, patient‐centered approaches to support postpartum mental health needs.

**3:30 PM – 5:00 PM**

## 672 | **Adverse** Outcomes **in Cesarean Scar Ectopic Pregnancies Continuing Into Mid‐ to Late‐Trimesters: A Meta Analysis**

Shuai Zeng; Jiangxue Qu; Huifeng Shi; Hai Jiang; Jie Yan; Yiwen Chong; Yuanyuan Wang; Lian Chen; Yangyu Zhao

Peking University Third Hospital, Beijing, Beijing

**Objective**: Current guidelines recommend early termination for cesarean scar ectopic pregnancy (CSEP). However, some patients express strong preferences for pregnancy continuation. Existing studies on mid‐ to late‐trimester outcomes remain sparse, heterogeneous, and derived from small cohorts. Crucially, the association between first‐trimester ultrasonographic classifications and subsequent adverse outcomes has not been systematically evaluated. This study aims to comprehensively and quantitatively evaluate mid‐ to late‐trimester outcomes for different subtypes of CSEP.

**Study Design**: Comprehensive searches were performed in PubMed, Embase, Cochrane library, CNKI, Yiigle databases up to February 12, 2025. Eligible studies reporting outcomes of CSEP continued beyond 14 weeks’ gestation were included. Data were synthesized using pooled incidence rates and odds ratios (OR) with 95% confidence interval (CI).

**Results**: 61 studies were included, of which 20 studies provided data suitable for meta‐analysis (Figure 1). The pooled incidences for placenta accreta spectrum (PAS) and hysterectomy were 0.90 (95% CI 0.81–0.96) and 0.39 (95% CI 0.22–0.57), respectively. 85% (95% CI 0.73–0.94) of pregnancies continued beyond 28 weeks, but only 19% (95% CI 0.10–0.30) beyond 37 weeks (Figure 2). Risk analysis indicated significantly elevated risks of invasive PAS and hysterectomy in type III CSEP compared to type I (OR 8.81, 95% CI 1.53–50.70) and II (OR 9.92, 95% CI 1.96–50.29). Exogenous CSEP also posed significantly greater risks for invasive PAS (OR 7.94, 95% CI 1.51–41.72) and hysterectomy (OR 9.22, 95% CI 2.66–31.98), compared to endogenous CSEP. Residual myometrial thickness (RMT) ≤3 mm substantially increased the risk of invasive PAS risk (OR 7.24, 95% CI 2.47–21.25).

**Conclusion**: Ultrasonographic classification and early measurement of RMT proved valuable for risk stratification in continuing CSEP into mid‐ to late‐trimesters. Pregnancies classified as type I, endogenous CSEP or RMT ≤ 3 mm showed more favorable outcomes, while type III, exogenous, or RMT >3 mm cases carried higher risks of adverse events.

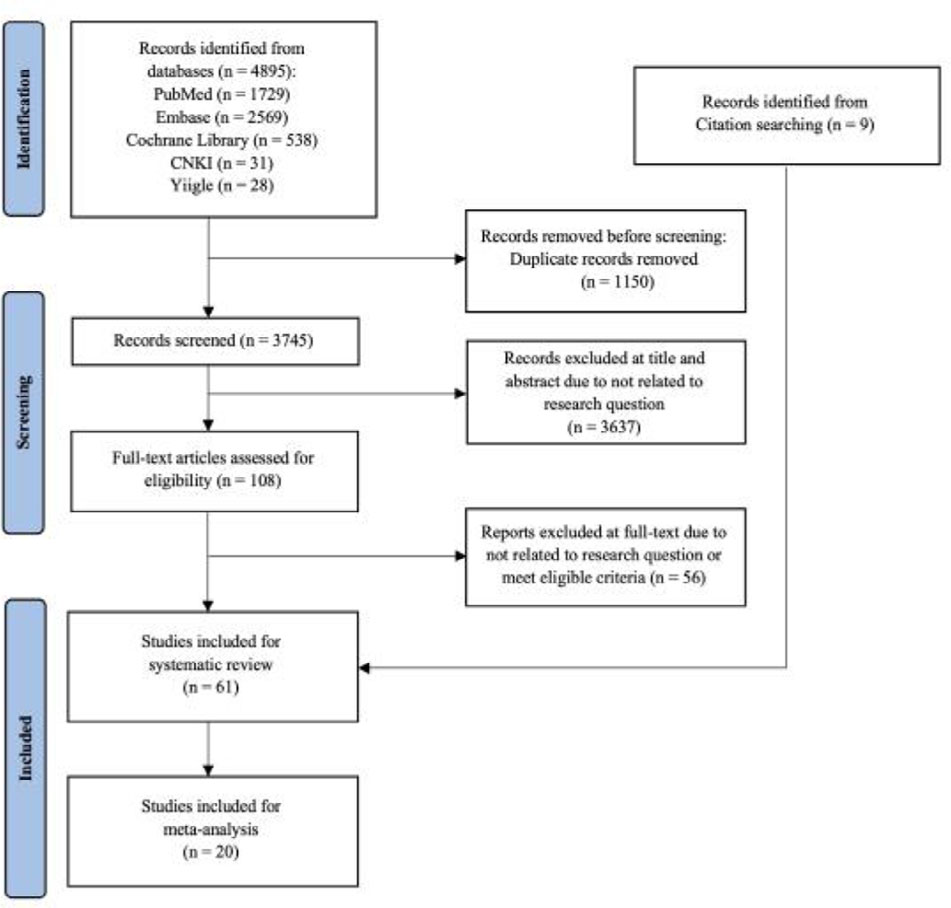

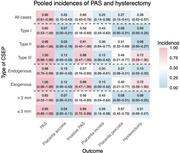

**3:30 PM – 5:00 PM**

## 673 | **Long‐Term** Outcomes **of Small‐For‐Gestational‐Age (Sga) Offspring Born to Mothers With and Without Uterine Malformations**

Sivan Chocron^1^; Polina Schwarzman^2^; Tamar Wainstock^3^; Eyal Sheiner^4^

^1^Department of Obstetrics and Gynecology, Soroka University Medical Center, Ben‐Gurion University, Beer Sheva, HaDarom; ^2^Soroka University Medical Center, Soroka University Medical Center, HaDarom; ^3^Department of Epidemiology, Biostatistics and Community Health Sciences, Faculty of Health Sciences, Ben‐Gurion University of the Negev, Beer Sheva, HaDarom; ^4^Soroka University Medical Center, Faculty of Health Sciences, Ben‐Gurion University of the Negev, beer sheva, HaDarom

**Objective**: Small‐for‐gestational‐age (SGA) neonates are known to be at increased risk for long‐term morbidities. Given that uterine malformations, a recognized risk factor for fetal growth restriction, may further compromise fetal development through impaired uteroplacental perfusion, we aimed to evaluate whether these neonates experience higher long‐term morbidities compared to SGA neonates born to mothers with normal uterine anatomy.

**Study Design**: This retrospective population‐based cohort study included all SGA neonates born between 1991 and 2021 at a single tertiary medical center. Multiple gestations and chromosomal abnormalities were excluded. SGA neonates were categorized based on maternal uterine anatomy: malformed versus normal. Long‐term cardiovascular, respiratory, neurologic, infectious, and gastrointestinal (GI) morbidities were defined based on recorded hospitalization files. Kaplan‐Meier survival curves and Cox proportional hazards models (controlling for maternal age, gestational age and mode of delivery) were used to analyze cumulative incidence and adjusted associations, respectively.

**Results**: Among 16,210 SGA neonates, 99 (0.61%) were born to mothers with uterine malformations. Uterine malformations were associated with increased risk of infectious morbidity (OR 1.70; 95% CI 1.10–2.50; *p* = .002) and GI morbidity (OR 1.70; 95% CI 1.10–2.70; *p* = .003) (Table). However, using a Cox regression model, only GI morbidity remained significantly associated with uterine malformations (adjusted HR 1.70; 95% CI 1.06–2.72; *p* = .028), whereas the association with infectious morbidity was no longer significant (adjusted HR 1.62; 95% CI 0.96–2.74; *p* = .070) (Figure).

**Conclusion**: Small for gestational age neonates born to mothers with uterine malformations are at an increased risk for long‐term gastrointestinal morbidity, suggesting a potential link between abnormal uterine anatomy and altered fetal gastrointestinal development.

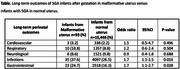

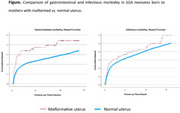

**3:30 PM – 5:00 PM**

## 674 | **Postpartum** Home **Blood Pressure Monitoring: Impact on Post‐Discharge Morbidity Among Individuals on Antihypertensive Medication**

Sonya Fabricant^1^; Emily Seet^2^; Natalie A. Bello^3^; Mariam Naqvi^2^; Sarah Kilpatrick^2^

^1^Cedars‐Sinai Medical Center, Los Angeles, CA; ^2^Department of Obstetrics and Gynecology, Cedars‐Sinai Medical Center, Los Angeles, CA; ^3^Department of Cardiology, Cedars‐Sinai Medical Center, Los Angeles, CA

**Objective**: To evaluate the impact of Postpartum Hypertension Program (PHP) implementation on morbidity following delivery discharge among individuals on antihypertensive medication.

**Study Design**: This was a prospective cohort study of individuals who delivered at a quaternary care center and were discharged on antihypertensive medication. Pre‐implementation controls (delivery discharge 1/1/21–1/20/24) were compared to post‐implementation cases (delivery discharge 3/21/24–3/31/25). The PHP included 1) twice‐daily home blood pressure (BP) monitoring and 2) MD‐guided antihypertensive titration for 2 weeks post‐discharge. The primary outcome was a composite of new‐onset morbidity within 30 days of discharge, including preeclampsia lab abnormalities, HELLP syndrome, eclampsia, pulmonary edema/acute heart failure, cerebrovascular disorders, myocardial infarction/cardiac arrest, liver hematoma, acute renal failure and ICU admission. Secondary outcomes included hospital return within 30 days of discharge, hospital return length of stay, and maximum BP during hospital return. Bivariate analysis compared cases and controls using chi‐square and t‐test as appropriate.

**Results**: Of 717 eligible individuals, there were 218 cases and 468 controls. Cases were more likely to be diagnosed with gestational hypertension, less likely to be diagnosed with severe preeclampsia, and had lower systolic BP in the 24 hours prior to delivery discharge (Table 1). PHP implementation was associated with a reduction in post‐discharge morbidity (0% vs 1.9%, *p* = 0.04), driven by lower rates of new‐onset pulmonary edema/acute heart failure and preeclampsia lab abnormalities. There was no difference in rate of hospital return between cases and controls (5.1% vs 4.9%, *p* = 0.94) (Table 2). Remaining secondary outcomes were similar between groups.

**Conclusion**: Among individuals on antihypertensive medication, PHP implementation was associated with a significant reduction in post‐discharge morbidity. This finding was driven by lower rates of new‐onset pulmonary edema/acute heart failure and preeclampsia lab abnormalities.

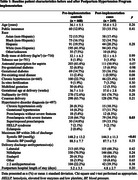

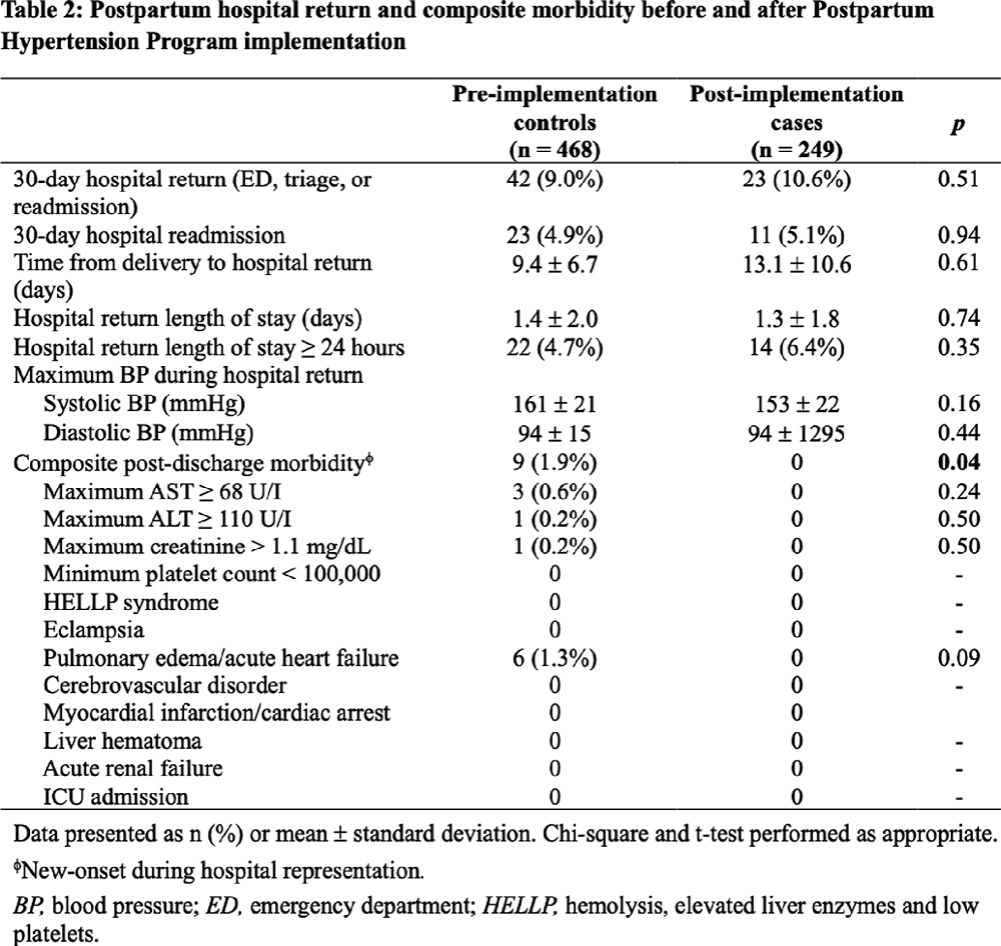

**3:30 PM – 5:00 PM**

## 675 | **Change In** Interpregnancy **Bmi and Perinatal Outcomes Among Overweight Individuals During Their First Pregnancy**

Stacia C. Hickey^1^; Sydney Bethers^2^; Bharti Garg^3^; Aaron B. Caughey^3^

^1^Oregon Health and Science University, Portland, OR; ^2^Oregon Health & Science University, Portland, OR; ^3^Oregon Health & Science University, Oregon Health & Science University, OR

**Objective**: As with the general U.S. population, BMI and obesity rates have risen among women of reproductive age. While it is understood that maternal overweight and obesity are associated with adverse perinatal outcomes, less data focus on the impact of interpregnancy BMI changes on subsequent pregnancies. This study compares the clinical outcomes of second pregnancies among women classified as overweight in their first pregnancy, examining differences between those who increased or decreased their BMI category and those who remained in the overweight category.

**Study Design**: This retrospective cohort study used linked birth certificate‐hospital discharge data for births in California between 2008–2020. We included two consecutive singleton, non‐anomalous births between 23–42 weeks’ gestation and required an overweight BMI category for the first pregnancy. We used chi‐square tests and multivariable Poisson regression models for adverse perinatal outcomes and adjusted risk ratios (aRR) were estimated.

**Results**: A total of 200,297 pregnancies met inclusion criteria. Of these cases, 53.1% remained in the same BMI category, 15.2% reduced BMI category, and 31.7%increased BMI category between pregnancies (Table 1). On multivariate regression, increased interpregnancy BMI was associated with increased risk of gestational diabetes (aRR = 1.39, 95% CI 1.35, 1.42), recurrent preeclampsia (aRR = 1.41, 95% CI 1.35, 1.47), cesarean delivery (aRR = 1.14, 95% CI 1.13, 1.16), postpartum hemorrhage (aRR = 1.09, 95% CI 1.03, 1.15), neonatal hypoglycemia (aRR = 1.15, 95% CI 1.07, 1.24) and large for gestational age newborns (aRR = 1.20 95% CI 1.18, 1.23). An increased interpregnancy BMI was also associated with a decreased risk of placental abruption (aRR = 0.85, CI 0.76, 0.94).

**Conclusion**: Changes in interpregnancy BMI are associated with differences in perinatal outcomes, with increases in BMI generally linked to worse outcomes. Further research is needed to understand these associations and guide weight management between pregnancies to reduce adverse outcomes.

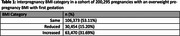

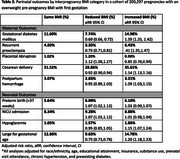

**3:30 PM – 5:00 PM**

## 676 | **Does Metformin Use in Pregnancy Decrease Infection Risk?: A Secondary Analysis of the MOMPOD Trial**

Steffany Conyers^1^; Olivia F. Schulist^1^; Kim Boggess^2^; Noelia Zork^1^

On behalf of the Eunice Kennedy Shriver National Institute of Child Health and Human Development (NICHD) Maternal‐Fetal Medicine Units (MFMU) Network, Bethesda, MD

^1^Columbia University Irving Medical Center, New York, NY; ^2^University of North Carolina at Chapel Hill, Chapel Hill, NC

**Objective**: Outside of pregnancy, metformin use has been variably associated with lower risk of infections. It is unknown whether this association is true in the pregnant population. Pregnancy is associated with an adaptive immunophenotype and thus may have more severe infections. We sought to assess if metformin use in pregnancy decreased rates of infection.

**Study Design**: This is a secondary analysis from the MOMPOD randomized trial comparing the addition of metformin versus placebo to the insulin regimen of pregnant people with early gestational or pre‐existing Type 2 diabetes <23 weeks’ gestation. We included those aged 18–46 who took at least one dose of study drug (metformin or placebo). The primary outcome was a composite of any infection during pregnancy or postpartum (chorioamnionitis, postpartum endometritis, mastitis, wound infections, COVID‐19, pyelonephritis, pneumonia, urinary tract infection, and septic pelvic thrombophlebitis). Secondary outcomes were infection‐related maternal and neonatal complications (neonatal sepsis, postpartum hemorrhage, wound complications). Categorical variables were analyzed using Pearson Chi square and Fisher exact test.

**Results**: *N* = 808 participants were included in this analysis (*n* = 404 on metformin and *n* = 404 placebo). The majority (77.1%) of participants had diabetes prior to pregnancy. Of those diagnosed in early pregnancy, the majority were either diagnosed using the 2‐step method (27.6%) or with an HbA1c ≥ 6.5% (28.1%). There were no significant differences in participant demographics (Table 1). Overall, no significant difference in any infections during pregnancy were observed between the metformin and placebo groups [RR: 0.94 (95% CI: 0.71, 1.24, *p* = 0.764)]. No cases of mastitis, pyelonephritis, or septic pelvic thrombophlebitis were observed. There were also no differences in the secondary outcomes (Table 2).

**Conclusion**: Metformin use in pregnancy does not appear to decrease the rate of infections or related complications during pregnancy (neither antenatal or intrapartum) or in the postpartum period.

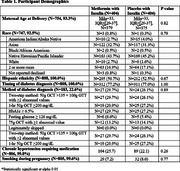

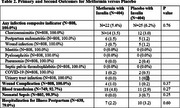

**3:30 PM – 5:00 PM**

## 677 | **Trends in** Attempted **Vaginal Delivery Among Triplet Gestations in the United States, 2014–2023**

Sweet Hope Mapatano^1^; Sarah Heerboth^2^; Annie Dude^3^; Tracy A. Manuck^4^

^1^University of North Carolina ‐ Chapel Hill, Chapel Hill, NC; ^2^University of North Carolina ‐ Chapel Hill, UNC Chapel Hill, Chapel Hill, NC; ^3^University of North Carolina, Chapel Hill, NC; ^4^Division of Maternal Fetal Medicine, Department of Obstetrics, Gynecology and Reproductive Science, University of North Carolina, Chapel Hill, NC

**Objective**: We sought to evaluate national trends in attempted vaginal delivery (VD) among triplet pregnancies in the US from 2014–2023.

**Study Design**: Using National Vital Statistics System data 2014–2023, we included triplet gestations ≥23 weeks. To ensure unique gestation‐level representation, only the first‐born infant of each set was analyzed. Trial of labor was defined as VD or trial of labor prior to cesarean delivery (CD). Trends in attempted VD during the study period were assessed using Joinpoint regression; measures of association were expressed as annual average percent change (AAPC) with 95% CI.

**Results**: Of 32,772 triplet infants, 10,716 were first‐born and had complete delivery data. Of these, 778 (7.3%) underwent trial of labor, with vaginal birth achieved in 385 (49.5%). Compared to those delivered by planned CD, those attempting VD were more likely to be publicly insured, identify as Black, and have higher education, greater parity, fewer co‐morbidities, and earlier gestational age, Table 1. The rate of attempted vaginal delivery for triplet A increased over the study period, from 5.7% in 2014 to 8.4% in 2023 (AAPC 6.97%, 95% CI 1.3–12.8%), Figure 1.

**Conclusion**: Attempted VD in triplet gestations increased significantly over the past decade. Further research is needed to determine whether maternal and neonatal outcomes vary by intended mode of delivery in this high‐risk population.

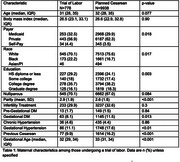

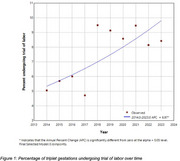

**3:30 PM – 5:00 PM**

## 678 | **Impact of** Simulation**‐Based Training on OB/GYN Residents’ Competence in Managing Second‐Stage Obstructive Labor Complications**

Tamar Shyldkrot^1^; Liliya Tamayev^1^; Lior Kashani Ligumsky^1^; Ann Dekalo^1^; Shiran Rona^2^; Ayala Shevach Alon^1^; Ilia Kleiner^2^; Daniel Tairy^1^; Laura Guzy^3^; offra Engel^1^; Emilie Ben Ezry^4^; Eran Weiner^2^; Yael Ganor Paz^3^

^1^Wolfson Medical Center, Department of Obstetrics and Gynecology, Wolfson Medical Center, HaMerkaz; ^2^Wolfson Medical Center, Department of Obstetrics and Gynecology, Holon, HaMerkaz; ^3^Wolfson Medical Center, Holon, HaMerkaz; ^4^Wolfson Medical Center, Department of Obstetrics and Gynecology, Wolfson, HaMerkaz

**Objective**: To evaluate the impact of weekly multidisciplinary simulation‐based training on residents’ knowledge and skills in managing second‐stage obstructive labor complications, including shoulder dystocia and impacted fetal head during cesarean delivery.

**Study Design**: This interventional study was conducted from November 2024 to August 2025. Weekly two‐hour simulation sessions were held in the labor ward, combining hands‐on practice with obstetric trainer manikins and communication/teamwork exercises involving residents and midwives. Senior physicians participated in each session—one portraying the patient and another evaluating resident performance. Scenarios were tailored by a multidisciplinary team. Residents’ knowledge and technical skills were assessed before and after the intervention using a written questionnaire and the Objective Structured Assessment of Technical Skills (OSATS), through both self‐assessment and senior physician observation.

**Results**: Thirty residents participated in 62 simulation sessions. Knowledge scores improved from 44.2 ± 15.9 to 65.0 ± 17.7 (*p* < 0.001), and OSATS scores increased from 38.3 ± 7.3 pre and post intervention respectively to 43.4 ± 10.5 (*p* = 0.007). Notably, a statistically significant difference was found between the residents’ self‐assessed OSATS scores after simulation (43.4 ± 10.5) and the evaluations by senior physicians (49.6 ± 9.0), highlighting improvement and the added value of expert feedback (*p* < 0.001).

**Conclusion**: Weekly simulation‐based training significantly improved residents’ knowledge and skills in managing second‐stage obstructive labor complications. These findings support incorporating simulation into obstetrics residency curricula to enhance clinical preparedness. Further research is needed to assess the effect on maternal and neonatal outcomes.

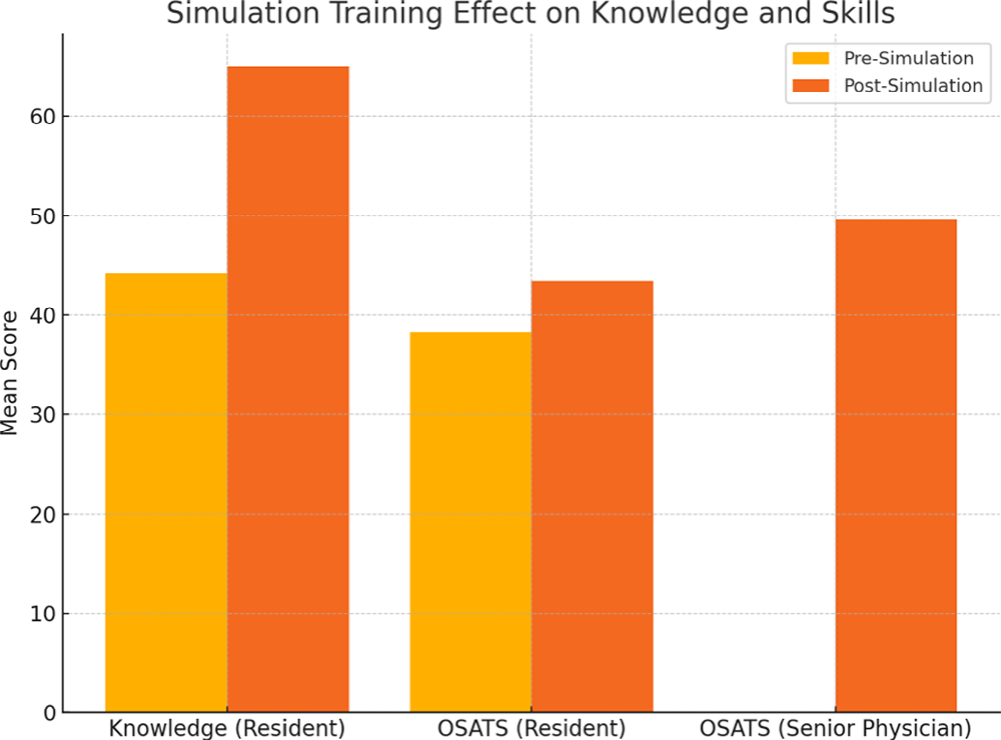

**3:30 PM – 5:00 PM**

## 679 | **Timing Of** Adjunctive **Azithromycin Administration and Postpartum Infection**

Taylor S. Freret^1^; Ethan Litman^2^; Mark A. Clapp^3^; Sarah E. Little^4^

^1^Beth Israel Deaconess Medical Center, Brookline, MA; ^2^Beth Israel Deaconess Medical Center, Boston, MA; ^3^Department of Obstetrics and Gynecology, Mass General Brigham, Massachusetts General Hospital, MA; ^4^Beth Israel Deaconess Medical Center, Newton, MA

**Objective**: The C/SOAP trial (2016) showed that perioperative azithromycin for labored cesarean birth reduces postpartum infection. Optimal administration timing, such as the interval before delivery or relation to umbilical cord clamping, is unknown. Our objective was to analyze whether infectious complication rates vary by timing of administration.

**Study Design**: This was a retrospective cross‐sectional study (2019–2025) using the nationwide Epic Cosmos platform. Laboring persons who gave birth to a liveborn singleton by cesarean delivery at 24–43 weeks were included. Exclusion criteria included no documented antibiotic administration. Initiation time of azithromycin was categorized as: no administration (reference), before birth (>60 min, 41–60 min, 21–40 min, 0–20 min) or after birth (1–20 min, >20 min). The primary outcome, adapted from the C/SOAP trial, was a composite of endometritis, wound infection, or other infection within 6 weeks, identified by ICD codes. Poisson regression with fixed effects for delivery department and calendar year was used to estimate relative risks, adjusting for maternal age, delivery BMI, parity, diabetes, prior cesarean birth, reaching the second stage, rupture of membranes ≥18 hours, and gestational age at delivery.

**Results**: The cohort included 141,528 individuals; azithromycin was administered to 66,951 (47.3%). Timing of azithromycin administration varied widely (Figure 1). The median administration time was 18 min before birth [IQR: 9–33 min before birth]. In the adjusted model, compared to no administration, azithromycin initiated from 0 to 60 minutes before birth was associated with decreased infection (Figure 2). Administration after delivery (and thus cord clamping) was not associated with reduced infection, although this may be confounded by urgency of delivery.

**Conclusion**: The timing of azithromycin administration for labored cesarean birth varies widely nationwide. Our findings suggest the rate of infectious complications also varies by timing and is lower when given in the hour before delivery.

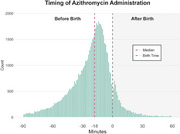

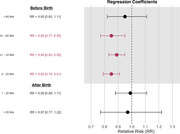

**3:30 PM – 5:00 PM**

## 680 | **From** Hysterectomy **to Preservation: Impact of a Multidisciplinary REBOA Protocol in Placenta Accreta**

Tehilla Stamler‐Yakobi^1^; Shay Porat^2^; Yehuda Ginosar^3^; Shlomo Yahalomy^2^; Doron Kabiri^4^; Jonathan Lober Lorver^5^; David Shweiki^2^; Aharon Tevet^2^

^1^Hadassah Medical Center and Hebrew University of Jerusalem, Jerusalem, Israel, Hadassah Medical Center and Hebrew University of Jerusalem, Jerusalem, Israel, Yerushalayim; ^2^Department of Obstetrics and Gynecology, Hadassah Hebrew University Medical Center, Ein Kerem, Jerusalem, Yerushalayim; ^3^Department of Anesthesiology, Hadassah Hebrew University Medical Center, Ein Kerem, Jerusalem, Yerushalayim; ^4^Hadassah Ein‐karem hospital, Obstetrics and gynecology department, Jerusalem, Jerusalem, Yerushalayim; ^5^Department of Vascular Surgery, Hadassah Hebrew University Medical Center, Ein Kerem, Jerusalem, Yerushalayim

**Objective**: To compare maternal and fetal outcomes in patients undergoing cesarean delivery (CD) for placenta accreta before and after the introduction of a designated multidisciplinary team incorporating REBOA (Resuscitative Endovascular Balloon Occlusion of the Aorta).

**Study Design**: A retrospective cohort study of all patients undergoing CD for placenta accreta in a tertiary care center between 2015–2025. We compared outcomes before (group A) and after (group B) the introduction of a multidisciplinary team including obstetrics, pelvic surgery, anesthesia, radiology, and vascular surgery. Data were retrieved from medical records and included maternal and neonatal outcomes. The primary outcomes were the need for hysterectomy and the number of packed red blood cell units transfused. Secondary outcomes included operating time, estimated blood loss, transfusion of fresh frozen plasma, incidence of bladder injury, need for secondary intervention (relaparotomy or interventional angiography), length of ICU stay >24 hours, type of anesthesia used, duration of post‐operative hospitalization, and the 5‐minute Apgar score.

**Results**: Among 81,512 deliveries, 56 patients had placenta accreta: 29 in group A and 27 in group B. Background characteristics‐maternal age, gestational age at delivery, parity, and accreta type‐were similar, though group B had more prior CDs (2.28 vs. 3.85, *p* = 0.00185). Only one patient in group B required hysterectomy, with concomitant uterine rupture, vs 79.3% in group A (*p* < .001). Packed cell and FFP transfusions were significantly lower (*p* < .001), as were blood loss (1590 vs. 2330 mL, *p* = 0.021) and general anesthesia (25.9% vs. 93.1%, *p* < .001). Fewer patients in group B had ICU stay >24 h (11.1% vs. 41.4%, *p* = 0.0047), and no postoperative infections were noted (0% vs. 13.8%, *p* = 0.0425). Apgar at 5 minutes was significantly higher (*p* = 0.0425). No significant differences were observed in rates of bladder injury or post‐op stay. Reintervention showed a non‐significant trend.

**Conclusion**: Implementing a multidisciplinary team with REBOA improved maternal outcomes and nearly obviated the need for hysterectomy.

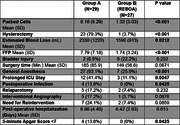

**3:30 PM – 5:00 PM**

## 681 | **Neonatal** Acidemia **And Severe Neonatal Outcomes Among Term Births According to Apgar Score**

Martina Benuzzi^1^; Grace Spencer^2^; Tetsuya Kawakita^3^

^1^Obstetrics and Gynecology Unit, Department of Medical and Surgical Sciences for Mothers, Children and Adults, University of Modena and Reggio Emilia, Azienda Ospedaliero‐Universitaria Policlinico, Modena, Italy., Modena, Emilia‐Romagna; ^2^Eastern Virginia Medical School at Old Dominion University, EVMS OBGYN, Virginia Health Sciences at Old Dominion University, VA; ^3^EVMS Department of Obstetrics and Gynecology, Macon and Joan Brock Virginia Health Sciences at Old Dominion University, Norfolk, VA, United States, Norfolk, VA

**Objective**: A normal Apgar score does not exclude neonatal acidemia, which is linked to serious morbidity. We evaluated the association between umbilical arterial acidemia at birth and severe neonatal outcomes, stratified by 5‐minute Apgar score.

**Study Design**: We performed a secondary analysis of two multicenter cohorts (Assessment of Perinatal Excellence [APEX] and Consortium on Safe Labor [CSL]). We included term neonates (≥37 weeks) with both 5‐minute Apgar scores and umbilical arterial pH (UApH). Acidemia was defined a priori as UApH <7.10; an abnormal Apgar score as <7 at 5 minutes. Neonates were categorized into four groups: (1) normal Apgar/no acidemia, (2) acidemia only, (3) abnormal Apgar only, and (4) both abnormal Apgar and acidemia. The primary outcome was a composite of severe neonatal outcomes (neonatal death, seizures, or suspected hypoxic‐ischemic encephalopathy [HIE]). We used modified Poisson regression with robust variance to estimate adjusted relative risks (aRRs) and 95% confidence intervals (CIs), adjusting for maternal age, body mass index, chronic hypertension, preeclampsia, pregestational diabetes, and a dataset‐level random effect.

**Results**: Among 51,014 term births, 47,790 (93.7%) had normal Apgar/no acidemia, 519 (1.0%) had acidemia only, 2403 (4.7%) had abnormal Apgar only, and 302 (0.6%) had both. Maternal demographic and clinical characteristics differed across groups (Table 1). Compared with normal Apgar/no acidemia, risk of the severe composite increased stepwise: abnormal Apgar+acidemia (13.9%; aRR 221.11, 95% CI 133.84–365.28), acidemia only (3.5%; aRR 54.15, 95% CI 29.24–100.27), and abnormal Apgar only (0.7%; aRR 11.62, 95% CI 6.28–21.53) (Table 2). Component outcomes showed parallel patterns; however, several estimates were imprecise because events were rare.

**Conclusion**: Umbilical arterial acidemia at birth is a strong, independent predictor of severe neonatal morbidity and mortality, even when the 5‐minute Apgar score is normal. These findings support consideration of cord blood gas analysis at term delivery to inform early neonatal management.

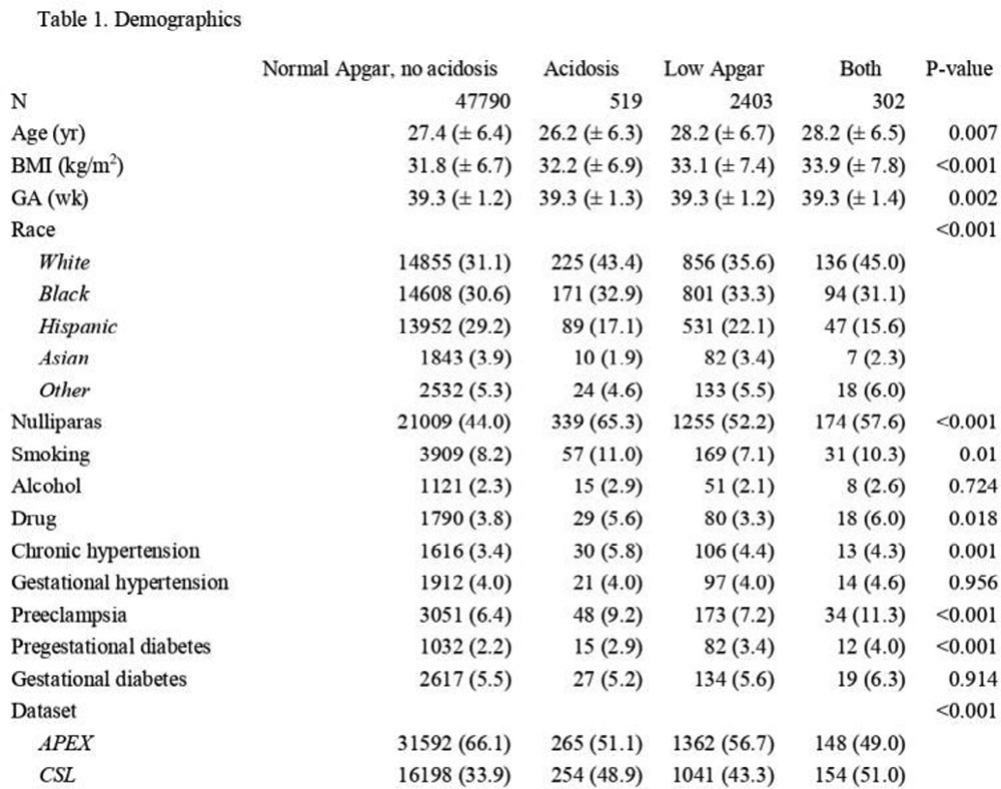

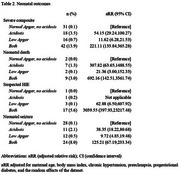

**3:30 PM – 5:00 PM**

## 682 | **Induction of** Labor **at 39 Weeks and Cesarean Delivery Rates: A Nulliparous Cohort Study**

Thibaud Quibel^1^; Anne‐Sophie Boucherie^2^; Anne Rousseau^3^; Camille Bouyer^4^; Patrick Rozenberg^5^

^1^Universite Paris Saclay, UVSQ, INSERM UE1018 Epidemiologie Clinique CESP, Universite Paris Saclay UVSQ INSERM UE1018 Epidemiologie Clinique CESP, Ile‐de‐France; ^2^Maternity of Poissy Saint Germain en Laye, Ile de France, France, Maternity of Poissy Saint germain en Laye, Ile‐de‐France; ^3^UVSQ, university of Versailles Saint Quentine en Yvelines, Ile‐de‐France; ^4^MYPA, MYPA, Ile‐de‐France; ^5^American Hospital of Paris, American Hospital of Paris, Ile‐de‐France

**Objective**: To analyze the trend of induction at 39 weeks of gestation (WG) since 2017 and to investigate the association between induction at 39 WG and cesarean delivery (CD) rates among nulliparous women at 39 WG.

**Study Design**: Nullipara with singleton pregnancies, cephalic presentation, who delivered from 39 WG+0d, within a French perinatal network, between January 1, 2017 and December 31, 2024, were included. Prelabor CD performed prior to 39 WG were excluded.

Monthly rates of induction at 39 WG, and CD rates were modelled over time. The association between induction of labor at 39 WG and CD was evaluated using a multivariate mixed‐effects model (accounting for maternity centers), adjusted for maternal age (>35 years), birth weight (>4000 g), and year of delivery. A sensitivity analysis was conducted excluding spontaneous births at 39 WG.

**Results**: A total of 34,143 nulliparous women were included. During the study period, the induction rate at 39 WG was 9.8% (3,342/34,143), with a significant yearly increase over time. (1,1%, 95% CI [0,8%‐1,4%],*p* < 0,001. The overall CD rate was 20.9% (7,150/34,143), without significant temporal change (+0,1% per year, *p* = 0,26). Induction at 39 WG, maternal age >35 years, and birth weight >4000 g were significantly associated with an increased CD risk: Adjusted OR (aOR) = 1.71 [1.57–1.85] for induction of labor, aOR = 1.70 [1.58–1.82] for maternal age >35 years, and aOR = 2.73 [2.48–3.00] for birth weight >4000 g. The sensitivity analysis yielded similar results.

**Conclusion**: Induction rate at 39 WG increased during the study period and was significantly associated with an increased risk of CD. However, its low incidence did not significantly impact the overall CD in nulliparous women at 39 WG. The discrepancy between our results and previous publication are possibly explained by different organizational factors which may influence the CD risk and which require important future investigations.

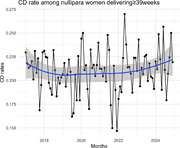

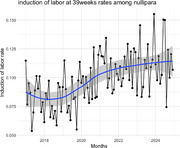

**3:30 PM – 5:00 PM**

## 683 | **Obstetrical** Outcomes **Following Preconception to Early Second Trimester Tumor Necrosis Factor‐Alpha Inhibitor Use**

Tina Hsieh; Shaza Shah; Audrey Mutoni; Ai‐ris Y. Collier

Beth Israel Deaconess Medical Center, Boston, MA

**Objective**: There is conflicting evidence for pregnancy outcomes associated with TNF‐α inhibitor (TNFαi) use among women with autoimmune indications, limiting guidance to this high‐risk population. We aimed to characterize adverse pregnancy outcomes for TNFαi use from preconception to early second trimester.

**Study Design**: The TriNetX U.S Collaborative Network was queried for deliveries from 11/6/2012‐ 12/3/2024 among psoriasis, inflammatory bowel disease, rheumatoid arthritis, ankylosing spondylitis, or juvenile arthritis patients. TNFαi group was defined as having ≥ 1 prescription of etanercept, adalimumab, infliximab, certolizumab, or golimumab during 1‐year preconception to early second trimester. We defined two controls: 1) non‐users; 2) active comparator users (hydroxychloroquine, sulfasalazine, azathioprine, mesalamine). TNFαi and controls were 1:1 propensity score matched (PSM) on demographics, comorbidities, healthcare use, BMI, and medications. Index exposure date was gestational age 18–24 weeks (ICD‐10 codes Z3A.18–24). Hazard ratios (HR) and 95% confidence intervals (CI) of outcomes including preeclampsia, eclampsia, small & large for gestational age infant, stillbirth, gestational diabetes, placental abruption, cesarean section, ante‐, intra‐, and postpartum hemorrhage, preterm premature rupture of membranes, venous thromboembolism, oligohydramnios, chorioamnionitis, and major adverse cardiovascular events (MACE), were calculated with cox proportional hazards model. The groups were followed up from index date until 1) outcome; 2) loss to follow‐up; 3) 6 months.

**Results**: 1667 TNFαi users were PSM‐matched to non‐users, and 1165 to active comparators. TNFαi use significantly reduced risk of placental abruption (HR 0.49, 95% CI 0.28–0.84) and MACE (HR 0.42, 95% CI 0.21–0.85), but not other outcomes compared to non‐users. No differences in any outcomes were found between TNFαi users and active comparators.

**Conclusion**: Preconception to early second trimester TNFαi use was not associated with adverse pregnancy outcome risks, supporting obstetric safety profile.

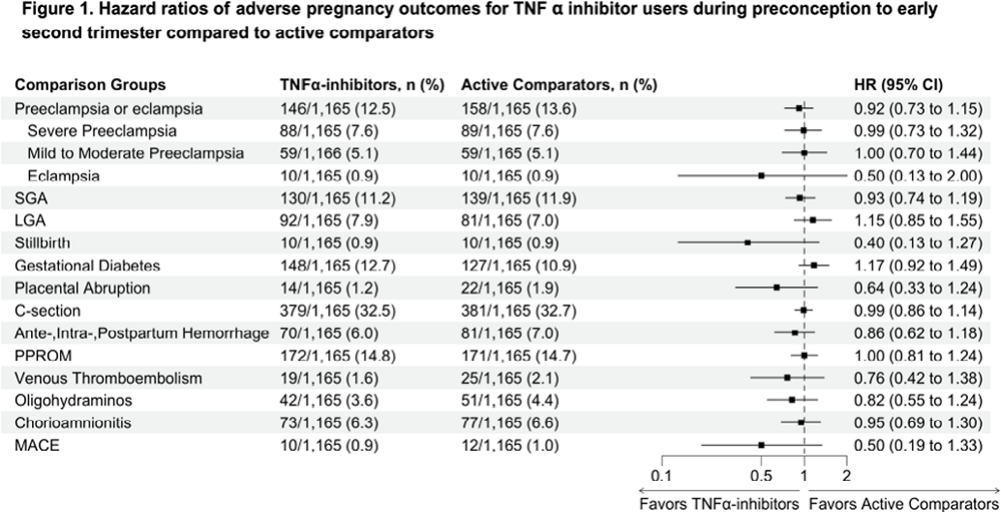

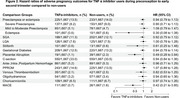

**3:30 PM – 5:00 PM**

## 684 | **Association of** Psychosocial **Stress and Discrimination With Perceptions of Respectful Prenatal Care and Autonomy**

Tiwadeye I. Lawal; Thanuja Suram; Rhea Sharma; Lynn M. Yee; Stephanie A. Fisher

Northwestern University Feinberg School of Medicine, Chicago, IL

**Objective**: To evaluate the association of patient‐reported psychosocial stress and discrimination in pregnant people with perceived respect and autonomy during prenatal care.

**Study Design**: Secondary analysis of data from a prospective cohort study that enrolled publicly insured singleton gestations to evaluate sociobiologic factors associated with adverse pregnancy outcomes. For this analysis, exposures were self‐completed psychosocial scales assessing stress, depressive symptoms, anxiety, discrimination, and social support (Table), conducted at 12–30 (Visit 1) and 24–36 (Visit 2) weeks’ gestation, at least 8 weeks apart. Co‐primary outcomes were Mothers on Respect Index (MORI) and Mother's Autonomy in Decision Making (MADM) survey scores (Visit 2). Exposures and outcomes were analyzed as continuous measures via generalized estimating equations, with models adjusted for relevant covariates.

**Results**: Of 180 participants in the parent study, the MORI and MADM were administered to the final 60 participants (7/2024–5/2025) and completed by 57 (95%), of whom 57% identified as Black and 39% as Hispanic. At Visit 1, each 1‐point higher PSS‐10 score was associated with a 1.19 point higher MORI score (95% CI 0.36, 2.03), indicating greater perceived stress was associated with perceptions of more respectful prenatal care. In contrast, higher PHQ‐9 (adj. ß −0.62; 95% CI −0.09, ‐1.16), GAD‐7 (adj. ß −0.38; 95% CI −0.05, −0.72), and EDS (adj. ß −0.43; 95% CI −0.12, −0.74) scores at Visit 1 were inversely associated with the MADM, indicating greater depressive/anxiety symptoms and discrimination were associated with lower perceived autonomy. At Visit 2, each 1‐point higher MDS score, indicative of greater social support, was associated with higher MADM scores (adj. ß 0.22; 95% CI 0.09, 0.35).

**Conclusion**: Among low‐income pregnant people, patient‐reported psychosocial stress and discrimination are associated with differences in perceptions of respect and autonomy in prenatal care. Addressing these psychosocial determinants is essential for socially conscious patient‐centered care.

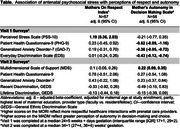

**3:30 PM – 5:00 PM**

## 685 | **Mid‐Pregnancy** Quantification **of Sub‐Clinical Inflammation by Blood‐Based and Vaginal‐Derived Cytokines and the Prediction of PTB**

Tracy A. Manuck^1^; Emily Gascoigne^1^; Sarah Heerboth^2^; Claire E. Jensen^1^; Hadley Hartwell^1^; Mandy Bush^1^; Matthew K. Hoffman^3^; William H. Goodnight^1^; Carmen Beamon^4^; Rebecca Fry^1^

^1^University of North Carolina, Chapel Hill, NC; ^2^University of North Carolina ‐ Chapel Hill, UNC Chapel Hill, Chapel Hill, NC; ^3^Christiana Care Health System, Newark, DE; ^4^WakeMed, Raleigh, NC

**Objective**: PTB risk assessment is imprecise, relying largely on clinical factors. Whether early pregnancy inflammatory biomarkers improve PTB prediction in asymptomatic patients is unknown.

**Study Design**: Primary analysis of a prospective, longitudinal cohort enriched for spontaneous PTB risk. Asymptomatic multiparous patients with singleton gestations were recruited <22 wks. Up to 2 blood and 2 vag samples per patient were collected 12–27+6 wks. 12 cytokines per sample were quantified via Luminex. We evaluated blood and vag cytokines collected early (12–19+6), later (20–27+6), and overall (12–27+6); we analyzed the earliest sample collected per GA epoch for each patient. Outcomes: primary = PTB <35 wks; Secondary = delivery GA, PTB <32 wks. Cytokines were classified as ‘high’ or ‘low’ using cut points optimizing sensitivity & specificity for PTB <35 wks. A pro‐inflammatory cytokine score was calculated per sample: +1 point per ***high* pro‐**inflammatory (IL‐1𝛂, IL‐1β, IL‐2, IL‐6, IL‐8, GM‐CSF, TNF‐𝛂, VEGF) & +1 point per ***low* anti‐**inflammatory (IL‐1RA, IL‐4, IL‐10, G‐CSF) cytokine. Logistic regression, Kaplan‐Meier, and AUC evaluated clinical, blood, vag, and blood+vag cytokine models.

**Results**: 437 patients were included; 15% delivered <35 wks, 8% <32 wks; median delivery GA = 38.1 wks (IQR 36.7, 39.3). Blood (*n* = 200 early, *n* = 239 later) was collected at a median 19.3 (IQR 16.3, 21.3) wks overall; blood cytokine scores = higher in those with PTB <35 (mean 3.7 ± 1.5) and <32 (mean 3.9 ± 1.4) vs. ≥ 37 wks (mean 3.2 ± 1.1), *p* < 0.001. Vag samples (*n* = 314 early, *n* = 255 later) were collected at a median 16.7 (IQR 15.9–19.1; *n* = 375) wks; vag cytokine scores did not differ by outcome, *p* >0.05. Blood & blood+vag models improved PTB prediction vs. clinical‐only models (Table) and were associated with delivery GA (Figure).

**Conclusion**: Subclinical systemic inflammation in maternal blood, particularly <20 weeks, is associated with PTB. Adding blood‐based cytokines to clinical data modestly improves PTB prediction and risk stratification.

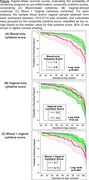

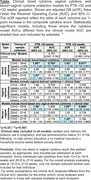

**3:30 PM – 5:00 PM**

## 686 | **Implementation of Universal Syphilis Screening in Patients Presenting for Birth Hospitalization: A Year in Review**

Tyler Lueck; Audrey Mutoni; Kathleen Clarke; Taylor S. Freret; Rebecca Zash; Ai‐ris Y. Collier

Beth Israel Deaconess Medical Center, Boston, MA

**Objective**: In April 2024, ACOG recommended syphilis screening at the time of delivery in response to increasing rates of congenital infection. We aimed to 1) describe rates and uptake of universal syphilis screening at birth hospitalization after implementation of quality improvement operational and educational interventions, and 2) describe the incidence of positive and equivocal screening results.

**Study Design**: We included all birthing persons admitted for delivery at a single urban academic hospital from June 1, 2024, to May 31, 2025. Interventions to improve screening included automatic inclusion of the Treponemal antibody (TP Ab) lab in the standard L&D admission order set in June 2024, a lecture about syphilis in pregnancy for trainees in Sept 2024, and an educational reminder at L&D team meetings with signage for all L&D staff initiated in Jan 2025. Process measures included rate of birthing patients per month that had a TP Ab result within 7 days of birth. Positive and equivocal TP Ab results were further evaluated using a reverse sequence algorithm and medical record review to assess if falsely positive or if indicative of a new or past infection.

**Results**: During the study, 4871 pregnant persons were admitted, of which 3580 (73%) were screened for syphilis at delivery. In June 2024, 21% of birthing persons were screened, and this increased to 94% by May 2025 (Figure 1). The median screening rates increased following trainee education from 57% to 77% and further increased to 93% after staff education. Twenty‐six patients had positive or equivocal TP Ab results: 1 new infection, 14 known, previously treated infections, and 11 false positive results (Figure 2).

**Conclusion**: Syphilis screening of birthing persons presenting for delivery improved significantly following our interventions. Overall, the incidence of new diagnoses of syphilis in birthing persons at our institution was lower than the hospital reported rate amongst all patients. Future efforts to assess the characteristics of patients still missing screening are needed.

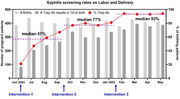

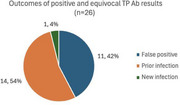

**3:30 PM – 5:00 PM**

## 687 | **Impact of** Hypoglycemia **During Glucose Testing in Pregnancy on Maternal and Neonatal Outcomes**

Tzuria Peled^1^; Tali Ben‐Ami^2^; Sarit Helman^3^; Maayan Bas Lando^3^; Sorina Grisaru‐Granovsky^4^; Misgav Rottenstreich^5^

^1^Shaare Zedek Medical Center, Jerusalem, Yerushalayim; ^2^Shaare Zedek Medical Center, Faculty of Medicine, Hebrew University of Jerusalem, Jerusalem, Israel., Yerushalayim; ^3^Shaare Zedek Medical Center, shaare zedek medical center, Yerushalayim; ^4^Shaare Zedek Medical Centre affiliated with The Hebrew University, Jerusalem, Yerushalayim; ^5^Hebrew University of Jerusalem, Jerusalem, Yerushalayim

**Objective**: Hypoglycemia in pregnancy, defined as a blood glucose level <60 mg/dL, has been inconsistently linked to adverse obstetrical outcomes, and available data remains scant. our study aimed to assess the association between hypoglycemia during the glucose challenge test (GCT, 50 g) and the oral glucose tolerance test (OGTT, 100 g) in pregnancy, and the risk of adverse maternal and neonatal outcomes.

**Study Design**: A multicenter retrospective cohort study was conducted, while including pregnant women who experienced hypoglycemia during either the GCT or OGTT. Women with hypoglycemia on each test were compared to those with normal glucose levels. Analyses included univariate comparisons followed by multivariable logistic regression.

**Results**: Of the cohort, 2.2% (*n* = 2,921/145,978) had hypoglycemia during the GCT, and 16.4% (*n* = 3,441/30,264) had at least one hypoglycemic value during the OGTT. In multivariable analysis, hypoglycemia during GCT was independently associated with lower rates of delivering a large‐for‐gestational‐age (LGA) neonate (adjusted odds ratio [aOR] 0.53, 95% CI 0.39–0.73), but with higher rates of a small‐for‐gestational‐age (SGA) neonate (aOR 1.56, 95% CI 1.24–1.97). No independent association was found between hypoglycemia on either test and overall adverse neonatal outcomes after adjustment.

**Conclusion**: In this large multicenter cohort, hypoglycemia during the GCT was associated with an increased risk of SGA but not with a higher risk of composite adverse neonatal outcomes. These findings may provide reassurance to clinicians and patients and help avoid unnecessary interventions.

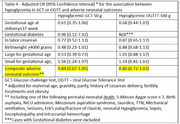

**3:30 PM – 5:00 PM**

## 688 | **Effect of Oral Loop Diuretics on Postpartum Blood Pressure Trajectories: A Randomized‐Controlled Trial Secondary Analysis**

Ukachi N. Emeruwa^1^; Minhazur Sarker^1^; Timothy Wen^2^; Natalie A. Bello^3^; Russell S. Miller^4^; Cynthia Gyamfi‐Bannerman^2^

^1^University of California San Diego, San Diego, CA; ^2^Division of Maternal Fetal Medicine, Department of Obstetrics, Gynecology and Reproductive Sciences, University of California San Diego, San Diego, CA; ^3^Department of Cardiology, Cedars‐Sinai Medical Center, Los Angeles, CA; ^4^Division of Maternal‐Fetal Medicine, Department of Obstetrics and Gynecology, Columbia University Irving Medical Center, New York, NY

**Objective**: In patients at risk for postpartum hypertension (PP HTN), physiologic volume shifts after delivery may contribute to hypertensive episodes. Diuretic therapy is proposed to limit this response without robust data to support efficacy. In this secondary analysis of a negative randomized trial (NCT04752475), we examined the effect of furosemide on longitudinal PP blood pressure (BP) patterns in patients at risk for PP HTN.

**Study Design**: We analyzed data of normotensive patients at high risk for de novo PP HTN (dnPPHTN) randomized to daily furosemide or placebo from PP day 1–5. BP was monitored every 4–8 hours while inpatient, then by remote monitoring twice‐daily for 6 weeks. The primary objective of this secondary analysis was comparison of BP trajectories between exposure groups. Secondary goals included exploring differential effects of furosemide on BP trends between those with or without dnPPHTN. Trends were assessed using local polynomial regression fitting. Linear mixed‐effects models were used to examine temporal BP trajectories.

**Results**: We analyzed 80 participants contributing 4129 PP BP readings. The furosemide group reached peak SBP earlier than the placebo group (PP day 8: 127.4 ± 14.9mmHg vs day 10: 120.7 ± 13.4mmHg) and reached peak DBP earlier (day 10: 83.1 ± 10.6mmHg vs day 12: 81.0 ± 10.3mmHg, Figure 1). Participants who developed dnPPHTN had more pronounced acceleration in BP peak when randomized to furosemide vs placebo (SBP: day 8 vs 9; DBP: day 10 vs 14), whereas normotensive participants had no SBP difference (day 9, both groups) and a smaller DBP shift (day 10 vs 12, Figure 2). Though there was no significant difference in the rate of dnPPHTN, median time to diagnosis was earlier in the furosemide group (PP day 3, IQR 3–6 vs day 5, IQR 5–5.5).

**Conclusion**: In normotensive patients with PP HTN risk factors, administration of a 5‐day course of oral furosemide led to earlier peak BP and earlier dnPPHTN detection. However, furosemide exposure did not influence dnPPHTN incidence, suggesting that PP fluid shifts are not the sole mechanism for pathophysiological BP changes in this population.

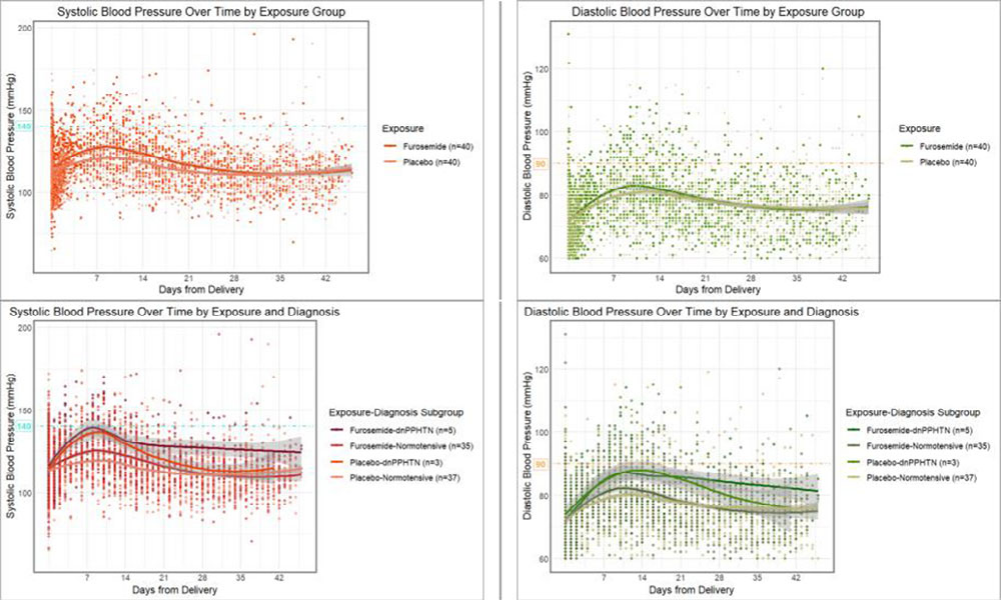

**3:30 PM – 5:00 PM**

## 689 | **Development of Pre‐Pregnancy Diet Quality Index and its Association With Adverse Pregnancy Outcomes**

Ummayhany Bharmal^1^; Grace Spencer^2^; Tetsuya Kawakita^3^

^1^Medical College Baroda, Vadodara, Gujarat; ^2^Eastern Virginia Medical School at Old Dominion University, EVMS OBGYN, Virginia Health Sciences at Old Dominion University, VA; ^3^Eastern Virginia Medical School Department of Obstetrics and Gynecology, Macon & Joan Brock Virginia Health Sciences at Old Dominion University, Norfolk, VA

**Objective**: We developed a Pre‐Pregnancy Diet Quality Index (PPDQI) and evaluated its association with adverse pregnancy outcomes (APO).

**Study Design**: This was a secondary analysis of data from the Nulliparous Pregnancy Outcomes Study: Mothers‐to‐Be (nuMoM2b), which assessed preconception dietary habits using food frequency questionnaires. PPDQI was developed using Dietary Guidelines for Americans, 2020–2025, and included 22 adequacy components and 4 moderation components, each scored from 0–5. Adequacy components received higher scores for greater intake; moderation components for lower intake. The total score was weighted 60% for adequacy and 40% for moderation. The primary outcome was a composite of APOs: preterm births <37 weeks, hypertensive disorders of pregnancy, small for gestational age (<5th percentile), gestational diabetes mellitus, and stillbirths. Modified Poisson regression was used to estimate adjusted relative risks (aRR) and 95% confidence intervals (CI) for APOs across PPDQI quartiles and per interquartile range (IQR) increase, adjusting for confounders.

**Results**: Of the 7,551,1819 (24%) had APO. The mean PPDQI score was 66.03 ± 9.44 (range:36.56–91.00). Higher PPDQI quartiles were associated with older age, non‐Black race, higher education, income, body mass index, and energy intake (Table 1). Compared to Q1, individuals in Q4 had a lower risk of APO (aRR: 0.77; 95% CI: 0.66–0.89), preterm births (aRR:0.75; 95% CI: 0.56–0.99), and hypertensive disorders of pregnancy (aRR: 0.77; 95% CI: 0.63–0.95) (Table 2). One‐IQR increase in PPDQI was associated with lower risk of APO (aRR: 0.89; 95% CI: 0.83–0.95), hypertensive disorders of pregnancy (aRR:0.89; 95% CI:0.80–0.98), preterm births (aRR: 0.86; 95% CI: 0.74–1.00), and stillbirths (aRR: 0.43; 95% 0.20–0.94).

**Conclusion**: Higher PPDQI scores were associated with lower risk of APOs, particularly preterm birth and hypertensive disorders. This supports incorporating dietary assessment and counseling into preconception care.

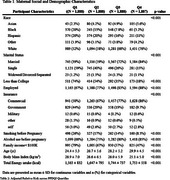

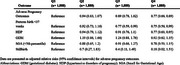

**3:30 PM – 5:00 PM**

## 690 | **Determining** Ethnic **Disparities Inherent to Tier 3 Versus Tier 4 Carrier Screening**

Vaishali Mehta; Emily B. Rosenfeld; Julia Geltch; Elena Ashkinadze

Rutgers Robert Wood Johnson Medical School, New Brunswick, NJ

**Objective**: The American College of Medical Genetics and Genomics (ACMG) defines carrier screening tiers by carrier frequency, recommending Tier 3 for the general population, and Tier 4 for consanguineous couples or those with a family history of genetic disease. The objective is to evaluate ethnic disparities in carrier detection between Tier 3 and Tier 4 panels, and to assess the utility of Tier 4 screening in an ethnically diverse cohort.

**Study Design**: This IRB‐approved retrospective chart review included patients ≥ 18 years who opted for a carrier screening panel between 1/2020–4/2024. Self‐reported ethnicity was mapped to WHO global regions. We determined how many carriers would have been missed if screening was limited to Tier 3 across ethnicities. Outcomes included prevalence of Tier 4‐only gene carriers and the number needed to screen (NNS) to detect them, stratified by region.

**Results**: Among 2000 patients, 1265 (65.7%) carried at least one Tier 4‐only condition, with regional rates ranging from 45–100%, and most populations showing ≥ 60% prevalence (Figure 1). Highest Tier 4‐only carrier rates included: Africa (76%), Caribbean (73%), Eastern Europe (71%), Western Europe (69%), and Southeast Asia (64%). NNS to detect one Tier 4‐only variant ranged from 1.0 to 2.2, with greatest efficiency in Africa, Southeast Asia, and Eastern Europe. Carrier couples of Tier 4 conditions were identified in regions including the Mediterranean (*n* = 10), Caribbean (*n* = 9), and Eastern Europe (*n* = 8). NNS to detect Tier 4‐only carrier couples ranged from 7.3 to 58.1, with the lowest values in Eastern Europe (7.3), the Caribbean (7.3), and the Mediterranean (9.7) (Table 1).

**Conclusion**: Tier 3 panels, which rely on carrier frequency thresholds largely derived from European populations, fail to identify carriers in underrepresented ethnic groups. Tier 4 provides greater carrier ascertainment across diverse populations and yields lower NNS, particularly in non‐European groups. Our data supports the utility of Tier 4 carrier screening in ethnically diverse populations.

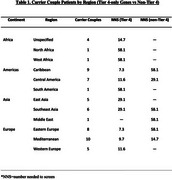

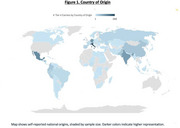

**3:30 PM – 5:00 PM**

## 691 | **Placenta Accreta Spectrum and Postpartum Mental Health: A Global Study**

Vanya Karabadji^1^; Mario Keko^1^; Tina Hsieh^1^; Uma S. Deshmukh^1^; Scott A. Shainker^2^; Anna M. Modest^1^

^1^Beth Israel Deaconess Medical Center, Boston, MA; ^2^Department of Obstetrics and Gynecology, Beth Israel Deaconess Medical Center, Harvard Medical School, Boston, MA

**Objective**: Placenta accreta spectrum (PAS) is associated with an increased risk of perinatal mental health disorders (MHD). However, the lasting MHD for patients with PAS, specifically compared with other high‐risk obstetric populations, remains unknown. The objective of the study was to determine the MHD toll on patients with PAS one year after delivery compared to patients at high risk of postpartum hemorrhage without PAS.

**Study Design**: The study analyzed 2016–2024 data from the TriNetX Global Collaborative cohort which includes electronic health record data from 148 health care organizations. We included patients with prior cesarean delivery and placenta previa in the index pregnancy and dichotomized them to PAS and no PAS based on ICD‐10 codes. Both groups were propensity score matched (PSM) on demographics, socioeconomic indicators and prior medical history including prior MHD. We assessed postpartum MHD diagnosis including anxiety, obsessive compulsive disorder, depression/other major depressive disorders, or stress disorders (including post‐traumatic stress disorder) up to one year post‐delivery. We used Cox regression to compare MHD between groups, reporting hazard ratios (HRs), and 95% confidence intervals (CIs).

**Results**: After PSM, 2706 PAS patients matched 1:1 to those without PAS. The groups demonstrated a mean age of 32.8 ± 5.4 (PAS) and 32.7± 5.6 no PAS), the majority being white (PAS vs no PAS: 55% vs 54%) and non‐Hispanic (57% vs 54%, respectively). Those with PAS were more likely to have a postpartum MHD diagnosis (HR 1.21, 95% CI 1.08–1.35) compared to those without PAS. PAS patients without a pre‐existing MHD diagnosis were more likely to have a postpartum MHD diagnosis (HR 1.61, 95% CI 1.34–1.94) in the year after delivery when compared to those without PAS.

**Conclusion**: Among a cohort of high‐risk pregnancies, individuals with PAS were more likely to have a postpartum MHD diagnosis in the year after delivery when compared to those without PAS. Future research on potential intervention strategies is needed.

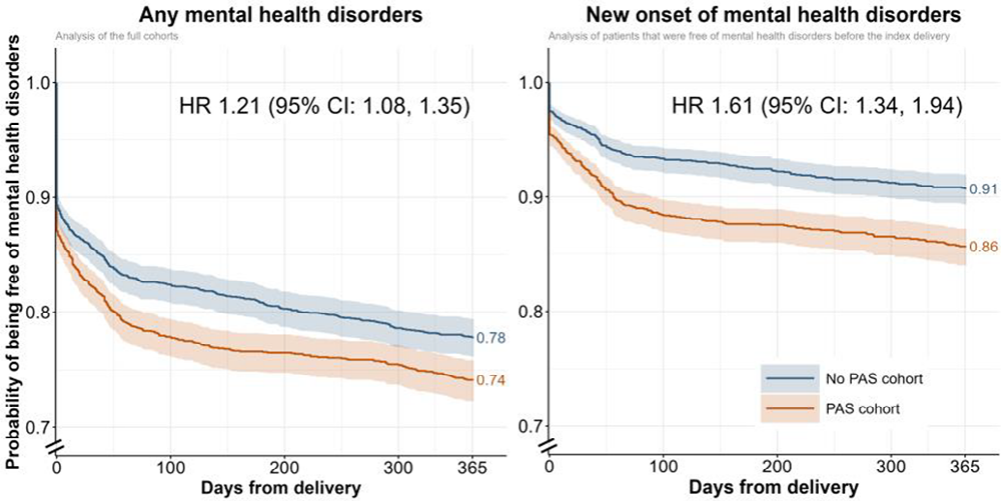

**3:30 PM – 5:00 PM**

## 692 | **What's the** Significance **of Nuchal Translucencies <3 mm But >95th Percentile in the Era of NIPT?**

Xiteng Yan^1^; Johanna A. Suskin^1^; Isaiah Smolar^1^; Thomas Owens^2^; Mia Heiligenstein^3^; Shelly Thai^1^; Ceyda Oner^4^; Jo Hsuan Lee^5^; Guillaume Stoffels^6^; Lois Brustman^1^; Zainab Al‐Ibraheemi^7^

^1^Icahn School of Medicine, Mount Sinai West, New York, NY; ^2^Icahn School of Medicine at Mount Sinai West, New York, NY; ^3^Icahn School of Medicine, Mount Sinai West, New York City, NY; ^4^Division of Maternal‐Fetal Medicine, Icahn School of Medicine at Mount Sinai West, New York, NY; ^5^Icahn School of Medicine at Mount Sinai, Icahn School of Medicine at Mount Sinai, NY; ^6^Icahn School of Medicine at Mount Sinai, New York, NY; ^7^Mount Sinai West, New York City, NY

**Objective**: An increased nuchal translucency (NT) is a well‐known marker for genetic and structural anomalies. Increased NT is typically defined either as a measurement >3 mm or by values >95th percentile. However, there is limited data on the significance of NT >95th percentile but <3 mm. We hypothesize that using the 95th percentile as a threshold for evaluation may enhance detection of anomalies in pregnancies with normal non‐invasive prenatal testing (NIPT).

**Study Design**: This retrospective study included patients who received prenatal ultrasounds at a large, academic center between 2018 and 2023. Participants with an NT <3mm, normal NIPT and a completed early or comprehensive anatomy ultrasound were included. Patients were divided into two groups‐ increased (>95th percentile) vs normal (<95th percentile) NT. Other data collected included invasive genetic testing, fetal echocardiography, and perinatal outcomes. Analysis was performed via Student's t‐test, Mann Whitney's U test, Chi square or Fisher's Exact test.

**Results**: A total of 176 cases were included: 58 (33.0%) had an increased NT and 118 (67.0%) had a normal NT. Results are in Table 1. Although odds of detecting anomalies were 78% higher with an increased NT, this was not significant (OR 1.78, 95% CI 0.54–5.84). No association was seen between an increased NT and anomaly detection on either early (*p* = 0.82) or full anatomy (*p* = 0.53). Patients with increased NT were more likely to get invasive genetic testing (*p* = 0.0004) and fetal echocardiograms (*p* = <0.0001). Increased testing did not improve detection of genetic (*p* = 1.00) or cardiac (*p* = 1.00) anomalies. Of note, patients with increased NT were more likely to be Asian/Pacific Islander or White (*p* < 0.0001) and have a male fetus (*p* = 0.011).

**Conclusion**: In patients with normal NIPT, an increased NT >95th percentile but <3mm was not linked to a higher rate of anomaly detection. Patients were more likely to pursue additional testing despite similar outcomes. Thus, an increased NT >95th percentile but <3mm may have limited clinical utility and could contribute to unnecessary testing and patient anxiety.

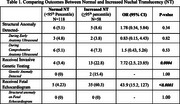

**3:30 PM – 5:00 PM**

## 693 | **Abdominal‐Pelvic CT For Postpartum Maternal Infectious Morbidities: Clinical Utility and Predictive Factors for Intervention**

Yonatan Dror^1^; Tal Israeli^2^; Ariel Rotem^3^; Omer Nir^4^; Michal Axelrod^5^; Yuval Dellaricha Vaisman, N/A^6^; Maor Givon^7^; Roy Mashiach^8^; Michal J. Simchen^9^; Shali Mazaki‐Tovi^10^; Michal Fishel Bartal^11^

^1^Sheba Medical Center, Sheba medical center, HaMerkaz; ^2^Sheba Medical Center, Ramat Gan, HaMerkaz; ^3^Google, New York City, NY; ^4^Department of Obstetrics and Gynecology, Sheba Medical Center, Tel Hashomer, Sheba Medical Center, HaMerkaz; ^5^Sheba Medical Center, Sheba Medical Center, HaMerkaz; ^6^Sheba medical center, Ganey Tikva, Tel Aviv; ^7^Medical University of silesia, Rosh haayin, HaMerkaz; ^8^The Department of Obstetrics and Gynecology, The Chaim Sheba Medical Center, Ramat‐Gan, Israel, Ramat Gan, Tel Aviv; ^9^Department of Obstetrics and Gynecology, Sheba Medical Center, Sheba Medical Center, HaMerkaz; ^10^Department of Obstetrics and Gynecology, Sheba Medical Center, Ramat Gan, Tel Aviv; ^11^Division of Maternal‐Fetal Medicine, Department of Obstetrics, Gynecology and Reproductive Sciences, Sheba Medical Center, Tel Aviv University, Israel, Ramat Gan, HaMerkaz

**Objective**: To determine the yield of abdominal‐pelvic computed tomography (CT) in the management of postpartum infectious complications unresponsive to empiric antibiotic therapy, and to identify maternal and labor characteristics associated with the need for interventional treatment.

**Study Design**: This was a retrospective cohort study conducted at a single tertiary medical center between March 2011 and May 2025.

We included postpartum patients who underwent abdominal‐pelvic CT scans for unresolved postpartum infection. Per protocol, our clinical practice is to perform abdominal‐pelvic CT scans for persistent postpartum fever and/or elevated inflammatory markers and/or presence of ultrasound findings suggesting intra‐abdominal abscess.

Patients with missing delivery data were excluded.

We compared clinical, laboratory, and radiological maternal characteristics between patients who required pelvic abscess drainage ‐ via either surgical (laparoscopy or laparotomy) or CT‐guided percutaneous intervention ‐ and those managed conservatively.

**Results**: During the study period there were 150,331 deliveries, 829 (0.55%) underwent abdominal‐pelvic CT, of whom 713 (86%) met inclusion criteria. Pelvic drainage was required in 78 (10.9%) patients.

Patients who required pelvic drainage had a longer median duration of Cesarean delivery (CD) and of ruptured membranes, higher rates of CD‐ particularly unplanned, and higher rates of intra‐operative use of hemostatic agents (Table 1). In the postpartum period they also experienced higher rate of fever, elevated inflammatory markers and pelvic abscess >5 cm compared with conservatively managed patients (Table 2).

**Conclusion**: Abdominal‐pelvic CT is a valuable diagnostic tool in the evaluation of postpartum infections unresponsive to empiric antibiotic treatment. Some clinical and laboratory features are associated with a higher likelihood of requiring interventional drainage and may aid in early identification of those individuals. Our findings supports the role of CT imaging in guiding timely and targeted postpartum management.

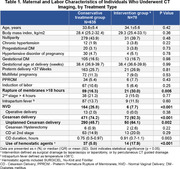

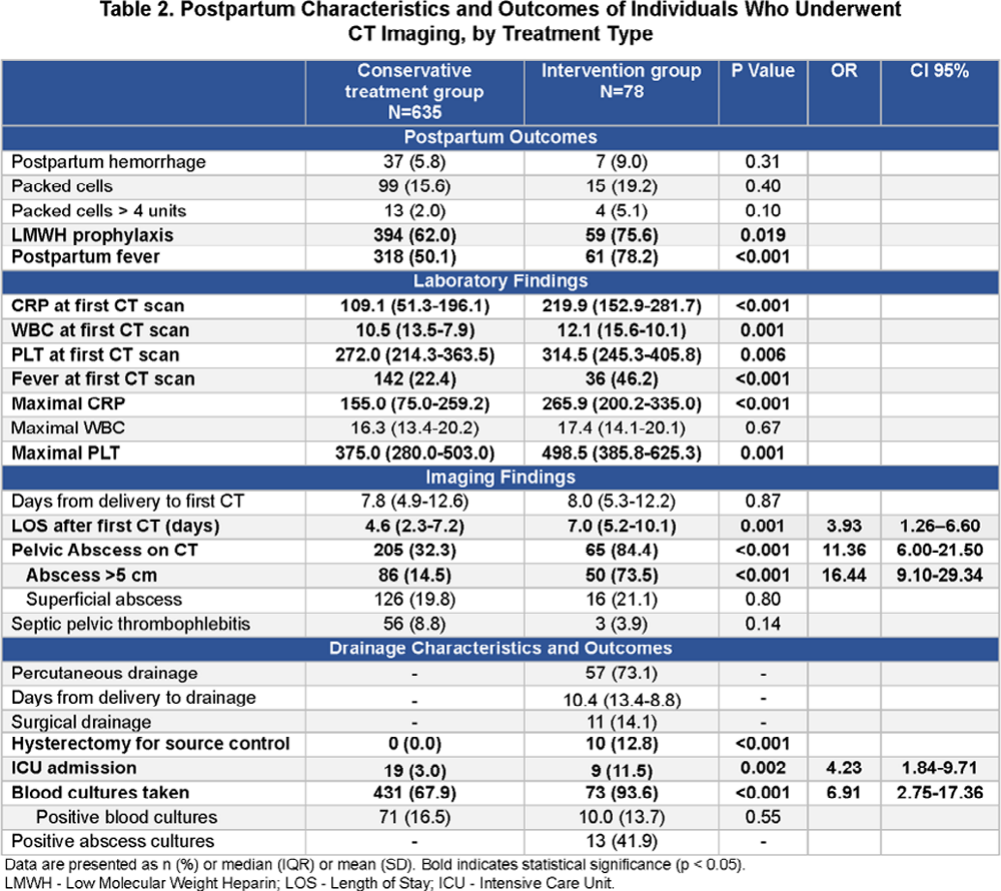

**3:30 PM – 5:00 PM**

## 694 | **Machine** Learning **Approach for Risk Prediction Among Expectantly Managed Early‐Onset Severe Preeclampsia**

Yossi Bart^1^; Zakaria Doughan^2^; Devangi Mahtani^1^; Joe Haydamous^3^; Ahmed Zaki Moustafa^4^; Sean C. Blackwell^1^; Baha M. Sibai^1^

^1^Division of Maternal‐Fetal Medicine, Department of Obstetrics, Gynecology and Reproductive Sciences, McGovern Medical School at UTHealth Houston, Houston, TX; ^2^Department of Obstetrics and Gynecology, McGovern Medical School at UT Health, Houston, Houston, TX; ^3^Department of Obstetrics, Gynecology and Reproductive Sciences, McGovern Medical School at UTHealth Houston, Department of Obstetrics and Gynecology, McGovern Medical School at UT Health, Houston, TX; ^4^Division of Maternal‐Fetal Medicine, Department of Obstetrics, Gynecology and Reproductive Sciences, McGovern Medical School at UTHealth Houston, University of Texas ‐ Houston, TX

**Objective**: During expectant management of preeclampsia with severe features <34 weeks, delivery is indicated when maternal or fetal condition deteriorates. This study aimed to develop machine learning models using data available at admission to predict short latency (delivery ≤3 days of diagnosis) and major maternal or fetal complications among patients managed expectantly.

**Study Design**: A retrospective cohort study at a Level IV referral center, including patients with preeclampsia with severe features between 23w0d and 33w3d (2016–2025). Patients who delivered within 1 day or presented with contraindications to expectant management were excluded. Using 87 baseline clinical, laboratory, and sonographic features available at admission, we compared patients with short vs. longer latency, and those with and without the composite adverse outcome (CAO; Figure 1). Four models (logistic regression, support vector machines, random forest, and eXtreme Gradient Boosting) were trained using five‐fold cross‐validation, grid search, and 100 repeated randomized train‐test splits. Undersampling was used to balance classes and minimize overfitting. The top predictors per outcome were used in the final models.

**Results**: Overall, 370 patients met inclusion criteria; 116 (31%) had a short latency, while 52 (14%) ultimately had the CAO. The best‐performing prediction model achieved an AUC of 0.70 (95% CI 0.61–0.79; Figure 1) for short latency with sensitivity of 0.72 (95% CI 0.55–0.89) and specificity of 0.69 (95% CI 0.52–0.86). For the CAO development, the AUC was 0.70 (95% CI 0.63–0.77) with 0.67 sensitivity (953% CI 0.50–0.84) and 0.72 specificity (95% CI 0.53–0.91). The predictors with highest mean importance for both models were chronic hypertension, maternal age, and urine protein‐creatinine ratio (Figure 2).

**Conclusion**: Machine learning models using admission data showed modest ability to predict early delivery and complications in expectantly managed preeclampsia with severe features. Integrating machine learning into clinical decision support systems may enhance early risk stratification and improve resource allocation.

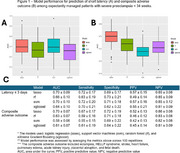

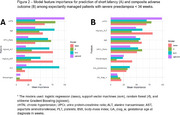

**3:30 PM – 5:00 PM**

## 696 | **Integrating** Complementary **Medicine in Labor: A Prospective Observational Study on Birth Experience and Outcomes**

Yuval Neeman^1^; Hadas Zafrir Danieli^1^; Irit Perez^2^; Rony Chen^1^; Asnat Walfisch^1^

^1^Rabin Medical Center, Petach Tikva, HaMerkaz; ^2^Ben‐Gurion University of the Negev, Be'er‐Sheva, HaDarom

**Objective**: To examine the association between the integration of certified complementary medicine (CM) practitioners in the labor room and the maternal birth experience—specifically satisfaction, perceived control and pain intensity. Secondary objectives included exploring associations between CM use and labor outcomes, and between specific CM modalities (massage, shiatsu, reflexology) and birth experience.

**Study Design**: This prospective observational cohort study was conducted at Rabin Medical Center (Beilinson Hospital) between July 2023 and September 2024. Eligible participants were women aged 18+ undergoing routine vaginal birth. Exclusion criteria included elective cesarean or stillbirth. Data were collected via postpartum digital questionnaires and medical records. Of 672 participants, 138 (20.5%) received CM therapy during labor. Statistical analysis included Mann‐Whitney U, chi‐square/Fisher's exact tests (p≤0.05), and logistic/linear regression models to adjust for confounders.

**Results**: Demographics and clinical characteristics were similar between groups. Women who received CM reported significantly lower average pain scores (6.55 vs. 7.19, *p* = 0.013) and fewer reports of severe pain (54.7% vs. 67.2%, *p* = 0.009). They also reported greater involvement in decision‐making (88.3% vs. 80.0%, *p* = 0.034) and higher responsiveness to concerns (92.0% vs. 81.5%, *p* = 0.004). Satisfaction was higher in the CM group (mean 9.09 vs. 8.81, *p* = 0.077). After adjusting for confounders, CM integration remained associated with reduced pain severity and improved perceived control. Among modalities, massage was significantly associated with increased control (*p* = 0.020), and shiatsu with reduced pain (*p* = 0.013). No statistically significant differences were found between groups in key labor outcomes, including mode of delivery, complications, or neonatal measures.

**Conclusion**: Integrating CM practitioners in labor may enhance women's birth experience by reducing pain intensity and improving perceived control. Specific modalities may offer targeted benefits.

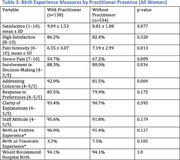

**3:30 PM – 5:00 PM**

## 697 | **Identifying Signs of Congenital Infections With Artificial Intelligence**

Remi Besson^1^; Yves Ville^2^; Charles Egloff^3^; Asma Khalil^4^; Yinka Oyelese^5^; Nikola Matevski^6^; Vianney Debavelaere^7^; Nicolas Fries^8^; Julien Stirnemann^9^; Olivier Picone^3^

^1^Sonio, Sonio, Paris, Ile‐de‐France; ^2^Hôpital Necker‐Enfants Malades, Paris, Ile‐de‐France; ^3^Hôpital Louis Mourier, Hôpital Louis Mourier, Ile‐de‐France; ^4^Kings College of Medicine, London, England; ^5^Harvard Medical School, Harvard Medical School, MA; ^6^Sonio, Sonio, Ile‐de‐France; ^7^Sonio, Paris, Ile‐de‐France; ^8^Imagyn'Echo, Imagyn'Echo, Languedoc‐Roussillon; ^9^Hôpital Necker Enfants Malades, Hôpital Necker Enfants Malades, Ile‐de‐France

**Objective**: Periventricular and subependymal cysts are frequent sonographic findings, often overlooked but which may be one of the rare manifestations of Cytomegalovirus (CMV) infection. An Artificial Intelligence (AI) able to automatically recognize these findings could serve as a safety net, prompting practitioners to conduct further investigations when necessary.

**Study Design**: This study evaluated an AI model (not yet approved) for detecting subtle cerebral findings, such as images of periventricular (PVC) or subependymal cysts (SC) and periventricular (PVN) or subependymal nodules (SN). Trained on 13797 images from 74 centers, including 1180 images of cysts or nodules from 70 CMV‐seroconverted pregnancies and 143 fetuses with isolated brain cyst. The model was evaluated on a test set from a center entirely excluded from the training set where experts identified 84 pathological axial brain images (59 PVC, 8 SC, 12 PVN, 5 SN) and 159 pathological coronal brain images (81 PVC, 21 SC, 19 PVN, 38 SN). The specificity was evaluated on 118 normal axial brain images and 14 coronal brain images from this test center, adding 126 normal coronal brain images not used for training but coming from the 74 training centers.

**Results**: The overall sensitivity was 84 %. The sensitivity was notably higher on coronal view 86,2%, than on axial views 78,6%. The system also exhibits a higher sensitivity on SC, 93,1%, or SN (79,1%) compared to PVC 86,4% or PVN (71%) where ultrasound artefacts are frequent on the anterior horns of the ventricle. The specificity of the system was 94,1% on axial brain images, 97,1% on coronal brain images.

**Conclusion**: AI has the potential to improve ultrasound screening for congenital brain cysts and, consequently, the detection of certain congenital infections. Further work is needed to strengthen the sensitivity of the model on nodules and on periventricular cysts of the anterior horn of the ventricle in axial views without significantly compromising specificity. In addition, learning the other signs of congenital infection should enable AI to greatly improve the relevance of the highlighted fetus.